# Dissecting the Cytochrome P450 OleP Substrate Specificity: Evidence for a Preferential Substrate

**DOI:** 10.3390/biom10101411

**Published:** 2020-10-06

**Authors:** Giacomo Parisi, Ida Freda, Cécile Exertier, Cristina Cecchetti, Elena Gugole, Gabriele Cerutti, Lucia D’Auria, Alberto Macone, Beatrice Vallone, Carmelinda Savino, Linda Celeste Montemiglio

**Affiliations:** 1 Istituto Pasteur-Fondazione Cenci Bolognetti and Department of Biochemical Sciences “Alessandro Rossi Fanelli”, Sapienza, University of Rome, P. le Aldo Moro, 5, 00185 Rome, Italy; giacomo.parisi@iit.it (G.P.); ida.freda@uniroma1.it (I.F.); cecile.exertier@uniroma1.it (C.E.); c.cecchetti17@imperial.ac.uk (C.C.); elena.gugole@uniroma1.it (E.G.); gc2695@columbia.edu (G.C.); beatrice.vallone@uniroma1.it (B.V.); 2 Current affiliation: Center for Life Nano Science @ Sapienza, Istituto Italiano di Tecnologia, Viale Regina Elena, 291, 00161 Rome, Italy; 3 Current affiliation: Department of Life Sciences Imperial College London, South Kensington Campus, London SW7 2AZ, UK; 4 Current affiliation: Zuckerman Mind Brain Behavior Institute, Columbia University, 3227 Broadway, New York, NY 10027, USA; 5 Department of Biochemical Sciences “Alessandro Rossi Fanelli”, Sapienza, University of Rome, P. le Aldo Moro, 5, 00185 Rome, Italy; dauria.1702926@studenti.uniroma1.it (L.D.); alberto.macone@uniroma1.it (A.M.); 6 Institute of Molecular Biology and Pathology c/o Department of Biochemical Sciences “Alessandro Rossi Fanelli”, Sapienza, University of Rome, National Research Council, P.le Aldo Moro, 5, 00185 Rome, Italy

**Keywords:** cytochrome P450, CYP107D1, OleP, 8.8a-deoxyoleandolide, oleandomycin, preferential substrate, X-ray crystallography, molecular docking

## Abstract

The cytochrome P450 OleP catalyzes the epoxidation of aliphatic carbons on both the aglycone 8.8a-deoxyoleandolide (DEO) and the monoglycosylated L-olivosyl-8.8a-deoxyoleandolide (L-O-DEO) intermediates of oleandomycin biosynthesis. We investigated the substrate versatility of the enzyme. X-ray and equilibrium binding data show that the aglycone DEO loosely fits the OleP active site, triggering the closure that prepares it for catalysis only on a minor population of enzyme. The open-to-closed state transition allows solvent molecules to accumulate in a cavity that forms upon closure, mediating protein–substrate interactions. *In silico* docking of the monoglycosylated L-O-DEO in the closed OleP–DEO structure shows that the L-olivosyl moiety can be hosted in the same cavity, replacing solvent molecules and directly contacting structural elements involved in the transition. X-ray structures of aglycone-bound OleP in the presence of L-rhamnose confirm the cavity as a potential site for sugar binding. All considered, we propose L-O-DEO as the optimal substrate of OleP, the L-olivosyl moiety possibly representing the molecular wedge that triggers a more efficient structural response upon substrate binding, favoring and stabilizing the enzyme closure before catalysis. OleP substrate versatility is supported by structural solvent molecules that compensate for the absence of a glycosyl unit when the aglycone is bound.

## 1. Introduction

Cytochrome P450s (P450s) are heme-containing enzymes virtually distributed in all living systems [1,2]. The members of this superfamily are considered the most versatile redox proteins known, since they are involved in a variety of physiological processes and in the biotransformation of drugs and xenobiotics [3,4]. This is even more remarkable given their conserved fold that achieves substrate specificity through diverse active sites yet conserves a general mechanism of catalysis and preventing unregulated solvent access and diffusion of radicals. Because of their versatility and the unique oxygen chemistry they catalyze, many studies are focused on developing strategies to exploit P450s as biocatalysts for biotechnological and biomedical applications [5,6,7]. 

**Figure 1:**
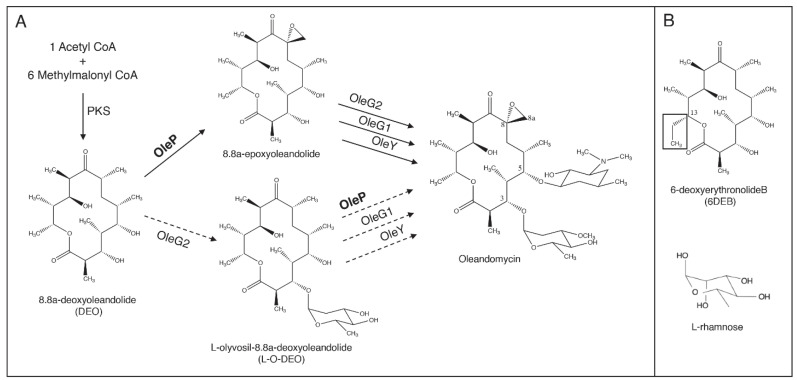
The biosynthetic pathway of oleandomycin. (**A**) OleP is active during the final steps of the biosynthesis of the antibiotic oleandomycin, where it catalyzes a reaction of epoxidation at the C8-C8a bond of the macrolactone ring. According to Gaisser et al. [12], OleP may introduce the epoxide function both to the aglycone and to the C3-monoglycosylated intermediates, splitting the process into two parallel pathways. In the figure, the pathways are distinguished by continuous and dotted arrows, respectively representing the epoxidation occurring on the aglycone intermediate DEO and the one performed on the monoglycosylated L-O-DEO. (**B**) The chemical structure of 6DEB and L-rhamnose are reported. A black square indicates the different substituent group at C13 of 6DEB with respect to DEO.

The bacterial P450 OleP, from *Streptomyces antibioticus*, is an epoxidase involved in the tailoring steps of the biosynthetic pathway of the antibiotic oleandomycin [8,9]. More specifically, OleP catalyzes a regio- and stereospecific reaction of epoxidation at the C8-C8a of the macrolactone ring (Figure 1). Among the known macrolide epoxygenases from actinomycetes, OleP presents a unique epoxidation chemistry since it targets a non-activated C-C bond, which is a reaction not easily achievable through conventional chemical synthesis protocols. This makes OleP a valuable target for protein engineering aimed at redirecting its epoxidase reactivity [10,11]. However, many aspects of the mechanism behind its enzymatic reaction are still unclear. Experimental evidence suggests that it proceeds through a desaturation step with the accumulation of an olefinic intermediate, which is converted into the final epoxide product, thus hypothesizing a double function of OleP [12]. In addition, discordant findings have been reported concerning the timing of OleP activity during the tailoring phase of oleandomycin biosynthesis, showing that OleP is either active on the aglycone intermediate, 8.8a-deoxyoleandolide (DEO), or on the monoglycosylated one, L-olivosyl-8.8a-deoxyoleandolide (L-O-DEO, Figure 1A) [13,14,15]. Both metabolites are commercially unavailable and represent challenging targets for synthesis, given the presence of multiple chiral sites. In 2002, the hypothesis of parallel pathways in oleandomycin biosynthesis was proposed by Gaisser et al. based on the analysis of the products of erythromycin intermediates and derivatives obtained in vivo by transplanting the OleP gene in *Streptomyces erythraeus* strains blocked in erythromycin biosynthesis. They concluded that OleP is potentially able to catalyze the epoxidation at C8-C8a of the macrolactone ring both on the aglycone and on the C3-monoglycosylated intermediates, without considering the possibility of a preferential substrate, a widespread feature of several macrolide P450s [12,16,17,18]. The recent availability of the OleP three-dimensional structure suggested that the active site of the enzyme could host both substrates [19]. Structural and kinetic studies performed with the aglycone substrate analog 6-deoxyerythronolide B (6DEB), from *S. erythraeus*, that only differs from DEO for the presence of an ethyl moiety at carbon 13 instead of a methyl unit (Figure 1B), revealed that substrate recognition in OleP is regulated by a complex mechanism that involves a large open-to-closed structural transition triggered by substrate binding, allowing the enzyme to adopt the catalytically competent conformation [20]. Notably, in the presence of 6DEB, only a minor population of the enzyme closes, showing that binding of this substrate does not fully favor the open-to-closed transition.

In this work, the substrate flexibility of OleP has been investigated by means of structural and functional studies performed on the putative natural aglycone substrate DEO and of *in silico* analysis using the monoglycosylated L-O-DEO, the other proposed substrate. The three-dimensional structure of the OleP–DEO complex revealed that it behaves similarly to the analog and non-physiological substrate 6DEB; binding of DEO to OleP does not lead to transition of the full population of the enzyme into the closed and catalytically competent state. Solvent molecules accumulate in a small cavity that forms over the active site when the enzyme closes, mediating the interactions with DEO. Docking of L-O-DEO into the active site of the OleP–DEO closed structure revealed that the sugar moiety can be accommodated in this small cavity, establishing direct hydrogen bonds with residues at the N-terminus of the internal helix I. In support of the *in silico* analysis and with the aim to experimentally simulate the binding of the substrate L-O-DEO, we determined the structure of OleP–DEO bound to L-rhamnose (Figure 1B), an analog of L-oleandrose, confirming that the small cavity can bind a glycosyl moiety. 

In light of these data, we propose that the monoglycosylated intermediate constitutes the preferential substrate of OleP; productive binding that drives and locks its closure might be more efficiently achieved thanks to direct interactions between the substrate and the N-terminus of the helix I of the enzyme when a glycosyl moiety is present. OleP substrate versatility is assisted by structural solvent molecules that compensate for the absence of a glycosyl unit when the aglycone oleandolide is bound.

## 2. Materials and Methods

### 2.1. Chemicals

Dimethyl sulfoxide (DMSO) and L-rhamnose were purchased by Millipore Sigma (Burlington, MA, USA). The 8.8a-deoxyoleandolide (DEO) was kindly provided by José A. Salas and Carmen Méndez (University of Oviedo, Spain), and 6-deoxyerythronolide B (6DEB) by Barrie Wilkinson and Rachel Lill (Biotica Technology Ltd., Cambridge, UK).

### 2.2. OleP Expression and Purification 

The gene encoding for OleP was cloned into the pET28b(+) vector and the enzyme was expressed in *E. coli* BL21 (DE3) and purified as previously described [19]. Homogeneity and monodispersion were assessed by SDS-PAGE and gel filtration chromatography using a BioFox 17/1200 SEC (Knauer, Berlin, GE) in 20 mM Tris·HCl and 200 mM NaCl, pH 8.0. In this condition, OleP elutes as a monomer [19]. 

### 2.3. Equilibrium Binding Analysis

Binding affinity to DEO and to 6DEB was determined at 298 K by titration of the wild type enzyme at a concentration of 2 μM in a final volume of 750 μL of 50 mM Hepes and 200 mM NaCl, pH 7.5. The stock solutions of DEO and 6DEB were prepared at a concentration of 26 mM in DMSO. Ligand concentrations ranged between 0 and 90 µM. The final percentage of DMSO was always maintained below 1%. The absorption shift of the Soret peak was followed by collecting UV–visible spectra (200–800 nm) after each substrate addition, and the appropriate blank was subtracted. Substrate binding was monitored by following the typical *Type I* absorption shift of the heme γ-Soret peak of OleP from 417 to 382–388 nm [21]. The dissociation constant (*K_D_*) was estimated using the Kaleidagraph software package. A nonlinear regression analysis was applied using hyperbolic equation ΔAUobs = ΔAUmax [L]/(*K_D_* + [L]), where ΔAUobs is the absorbance difference, ΔAUmax is the maximum absorbance difference extrapolated to infinite ligand concentration, and [L] is the ligand analytical concentration. All data were also globally fitted with the program Prism (GraphPad). 

To monitor the effect of the ionic strength on the spin-state equilibrium in OleP bound to DEO and to 6DEB, 2 μM OleP in the presence of 150 μM of DEO and 6DEB was titrated with increasing concentration of sodium formate, ranging from 0 to 3.5 M, in a final volume of 750 μL of 50 mM Hepes and 200 mM NaCl, pH 7.5, at 298 K. The absorption shift of the Soret peak was followed by collecting UV–visible spectra (200–800 nm) after each substrate addition, and the appropriate blank was subtracted.

### 2.4. Crystallization, X-Ray Data Collection, and Analysis

Co-crystallization trials were carried out with purified OleP at a concentration of 19 mg/mL (0.45 mM) in 20 mM Tris·HCl and 200 mM NaCl, pH 8.0, in the presence of saturating concentration of DEO (2 mM) using the hanging drop vapor-diffusion method at 294 K. High quality X-ray diffraction data were obtained from single crystals grown in different conditions (low salt, OleP–DEO_LS_ and high salt, OleP–DEO_HS_), as detailed in Table 1. OleP–DEO_LS_ crystals were obtained by applying the streak seeding technique on a drop containing the same amount of protein and ligand, with the precipitant solution reported in Table 1. Rapid soaking in mother liquor containing 10% glycerol was performed to cryoprotect crystals grown in high salt conditions before flash-cooling in liquid nitrogen. 

Co-crystallization of OleP (19 mg/mL) in the presence of DEO or 6DEB (1 mM) and L-rhamnose (2 mM) was performed in high salt conditions screening sodium formate from 4.0 to 4.4 M using the hanging drop vapor-diffusion method at 294 K (Table 1). Single crystals of OleP–DEO-rhamnose obtained at 4.4 M sodium formate were directly frozen in liquid nitrogen. Single crystals of OleP–6DEB–rhamnose obtained at 4.2 M sodium formate were cryoprotected in mother liquor containing 20% glycerol before flash freezing in liquid nitrogen.

Diffraction data were collected at 100 K at the European Synchrotron Radiation Facility (beamline ID23-2, Grenoble, France), and at ELETTRA (beamline XRD2, Trieste, Italy) with a Pilatus detector (Dectris, Baden-Dättwil, Switzerland). All diffraction data were processed by using the XDS package [22] and Aimless [23,24]. 

Crystallization conditions, data collection, and refinement statistics are summarized in Table 1.

### 2.5. Structure Determination and Refinement 

For OleP–DEO_LS_ and OleP–DEO_HS_, initial phases were calculated by molecular replacement using MOLREP [25] from the CCP4 package (version 7.0). The coordinates of a single open monomer of OleP in complex with 6DEB in low salt conditions (pdb 5MNV) were used as model for OleP–DEO_LS_ [20]; the atomic model of a single closed monomer of OleP-6DEB in high salt conditions (pdb 5MNS) was used as a template for OleP–DEO_HS_ [20]. Thanks to space group isomorphism, the atomic models of OleP–DEO-rhamnose and of OleP–6DEB–rhamnose were determined by the Fourier synthesis method using respectively as templates the atomic coordinates of OleP–DEO_HS_ and OleP-6DEB (PDB code 5MNS, [20]).

Nine monomers (A–I) and six monomers (A–F) were found in one asymmetric unit of OleP–DEO_LS_ and OleP–DEO_HS_, respectively. Six monomers (A–F) were identified in the asymmetric unit of OleP–DEO-rhamnose and OleP–6DEB–rhamnose. Cycles of model building and refinement of the structures were performed with Coot 0.8.9.1 [26,27] using an F_observed_–F_calculated_ (F_o_–F_c_) map contoured at 3 σ and the 2F_o_–F_c_ at 1 σ and Refmac5 [28] in the CCP4 suite. Five percent of the reflections were excluded from refinement and used for the R_free_ calculation [29]. Lack of electron density in OleP structures prevented the reconstruction of the first 5–13 N-terminal residues in all monomers and of segments 177–186 in monomer E, and 179–186 and 220–228 in monomer F of the OleP–DEO_LS_ model. Monomer I in the OleP–DEO_LS_ structure displays weak electron density accounting for only the 60% of the protein sequence. The similarity among open monomers and among closed ones is very high. For the open monomers, the overall rmsd on Cα ranges from a minimum of 0.35 Å for monomer G to a maximum of 0.56 Å for monomer E using monomer A as a reference in OleP–DEO_LS_; for the closed monomers, the overall rmsd on Cα ranges from a minimum of 0.29 Å of monomer A in the structure of OleP–DEO_HS_ to a maximum of 0.95 Å for monomer I in the structure of OleP–DEO_LS_ using monomer C in OleP–DEO_HS_ as a reference. Major differences between the open conformers (rmsd > 2 Å) are localized at segments 178–186 on the FG loop and 220-224 on the HI loop, with a maximum rmsd value of 4.5 Å on Leu221 (Figure S1A); two segments display a rmsd value exceeding 2.0 Å among the closed conformers, residues 208–210 at the C-terminus of the helix G, and 224–228 on the HI loop (Figure S1B). These regions are characterized by weak or absent electron densities given their natural flexibility, which is preserved within the lattice.

Given the overall similarity among all the open conformers and all the closed ones, for the structural analysis herein discussed, we chose the better-defined molecules A from OleP–DEO_LS_ and C from OleP–DEO_HS_ as representative, respectively, of the open and the closed conformations of the complex. We checked that all structural features described in this work are present in all monomers, when allowed by the quality of the corresponding electron density map. All the final statistics of the data collection and model refinement are displayed in Table 1. Real space correlation coefficients (RSCC) were estimated using Molprobity from the PHENIX suite and are reported in Table 1 [30]^,^ [31]. Figures were produced using Chimera [32]. The atomic coordinates and structure factors of OleP–DEO_LS_, OleP–DEO_HS_, OleP–DEO-rhamnose, and OleP–6DEB–rhamnose have been deposited in the Protein Data Bank (accession numbers: 6ZI2, 6ZHZ, 6ZI7, and 6ZI3, respectively). 

### 2.6. Molecular Docking 

The monoglycosylated intermediate L-O-DEO was docked into the active site of the OleP–DEO_HS_ structure in the closed conformation using Autodock Vina 1.1.2 [33]. All crystallographic waters were removed from the OleP–DEO_HS_ model prior to docking simulations. The three-dimensional structure of L-O-DEO in PDB format was generated and optimized using the PRODRG2 web server [34]. The correct number of rotatable bonds in the ligand was confirmed by manual inspection in AutoDockTools 1.5.6 and it was limited to the O-glycosidic bond. Polar hydrogens and Gasteiger charges were also added. The protein was kept rigid during docking calculations. The simulation cell consisted of a grid box, centered at the heme iron, and covering the whole distal heme pocket (x: 38 Å; y: 50 Å; z: 40 Å). The exhaustiveness parameter was default (= 8). The resulting binding pose obtained by a clustered docking was analyzed for binding energy and distances between the target oxidation bond of the ligand and the heme-iron. Tolerance (rmstol) for the cluster analysis was set to 3.0 Å. Amino acid residues contributing to substrate binding were imaged and analyzed using Chimera [32]. 

## 3. Results and Discussion

### 3.1. Binding Properties of OleP to the Aglycone Substrate

**Figure 2:**
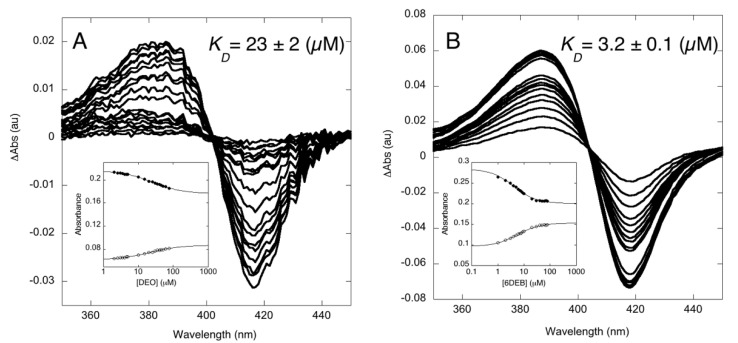
Equilibrium titration of OleP with DEO (**A**) and 6DEB (**B**) at 298 K. Difference spectra and absorbance intensities (relative inset) of OleP as function of total DEO and 6DEB concentration are reported. Data refer to the absorbance monitored at 417 nm (full dots) and at 382 nm for DEO and 388 nm for 6DEB (empty dots) at a constant concentration of OleP in 50 mM Hepes and 200 mM NaCl, pH 7.5. Lines are the best fit to hyperbolic functions.

The binding properties of OleP towards the physiological aglycone substrate DEO were assessed by equilibrium binding experiments. The UV–visible absorbance variation of the heme spectral properties of the enzyme at fixed concentration was followed upon increasing DEO concentration at 298 K. Binding of DEO to OleP produces the typical *Type I* spectral changes, with a peak at 382 nm and trough at 417 nm (Figure 2A), indicating the effects of substrate binding on the equilibrium of the heme iron spin-state. The observed binding curve follows a simple hyperbolic saturation function, returning a *K_D_* value of 23 ± 2 µM as estimated from a global analysis of 40 different wavelengths (ΔG = −6.32 ± 0.05 kcal/mol). The comparison with the binding properties of OleP with the substrate analog 6DEB in the same experimental conditions (*K_D_* = 3.2 ± 0.1 µM, ΔG = −7.45 ± 0.02 kcal/mol; Figure 2B) showed that DEO induces a less pronounced spin-state shift of the heme iron (ΔAbs_417nm_~0.03 compared to ΔAbs_417nm_~0.07 at 80 µM substrate concentration) and that OleP displays about 10 times lower affinity for DEO, which corresponds to a difference in free energy of the order of 1.2 kcal/mol. In an effort to provide a structural explanation for this behavior and to further investigate the molecular basis of the OleP versatility in substrate recognition and binding, we determined the crystallographic structure of OleP in complex with DEO.

### 3.2. The Structure of OleP Bound to the Oleandolide, DEO

**Figure 3:**
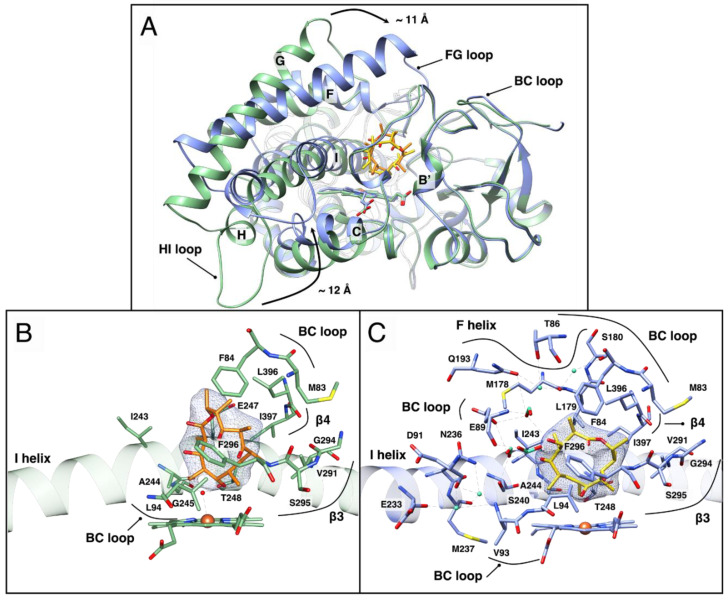
OleP–DEO structure. (**A**). Secondary structure superposition of the open (green) and closed (blue) conformations of OleP–DEO complex. The structures are displayed in a nonstandard orientation for P450s to enable the visualization of the structural transition. DEO molecules in the open and in the closed states are in orange and in yellow sticks, respectively. (**B**,**C**) Close up views of the active site of open (**B**, green) and closed (**C**, blue) OleP in complex with DEO. Red sphere: sixth coordinating water molecule; dashed lines: hydrogen bonds. In panel C, water and formate ions that mediate interactions between protein and substrate are represented as aquamarine spheres and sticks, respectively. Secondary structural elements and amino acids involved in OleP–DEO interactions are labeled. In both panels, the electron density map (2F_o_–F_c_) contoured at 1 σ around DEO is shown as a blue mesh.

Crystals of OleP bound to DEO were obtained in two crystallization conditions, namely low salt (OleP–DEO_LS_) and high salt (OleP–DEO_HS_) (see Table 1), that showed lattice isomorphism with the crystals obtained for OleP in complex with 6DEB in low salt (OleP-6DEB_LS_, pdb 5MNV) and high salt (OleP-6DEB_HS_, pdb 5MNS) conditions, respectively [20]. As for OleP-6DEB_LS_, the crystal of OleP–DEO_LS_ (ionic strength, I = 0.2 M) contains nine monomers in the asymmetric unit arranged in six open conformers and three closed ones. As observed for OleP-6DEB_HS_, high ionic strength conditions (I = 4 M) induced the crystallization of six copies of the closed OleP–DEO complex in the asymmetric unit. Therefore, in the presence of the physiological aglycone intermediate DEO, OleP can assume two conformational states, an open one (unproductive binding) and a closed one (productive binding) [20]. A pronounced conformational reorganization, that mainly involves the F and G helices, the FG, HI, and the BC loops, closes OleP over the substrate, blocking the access channel to the active site (Figure 3A). The same structural transition was observed when OleP binds 6DEB [20]. Both the open and the closed conformations of OleP–DEO closely resemble the structure of the enzyme bound to 6DEB. Indeed, the superposition of their secondary structure confirms a strong similarity (Figure S2). The rmsd, calculated by superposing the open structure of OleP–DEO with the open OleP-6DEB, yields a mean value of 0.51 Å. Only the 183–186 segment on the FG loop displays a rmsd value exceeding 2.0 Å (with a maximum at 4.3 Å for A186) (Figure S2). The rmsd, calculated by superposing the closed structure of OleP–DEO with the closed OleP-6DEB, yields a mean value of 0.17 Å, with a maximum of 0.58 Å for D224 on the HI loop (Figure S2). 

The initial F_o_–F_c_ omit map, calculated upon omission of DEO from the model, reveals the presence of a well-defined electron density in the active site of all OleP monomers in both crystal forms corresponding to one molecule of oleandolide. Similar to 6DEB, in both the open and closed states of OleP, DEO binds the active site while adopting an orthogonal orientation with respect to the porphyrin macrocycle (Figure 3); most of the contacts previously identified in 6DEB binding also contribute to the recognition and binding of DEO. However, the absence in DEO of the one-carbon extended aliphatic chain at C13 typical of 6DEB allows increased freedom of movement of the aglycone within both the open and closed OleP active sites, as detailed below. 

In the open active site, DEO assumes different orientations and conformations, but none of the six molecules of DEO expose the C8-C8a bond, which is the target of the OleP epoxidation, to the heme iron (Figure S3A). The residues of the open OleP interacting with DEO are located on the BC loop (M83, F84, L94), on the N-terminus of the I helix (I243, A244, G245, E247, T248), on β-hairpin β_3_ (V291, G294, S295, F296) and on β-hairpin β_4_ (L396, I397) (Figure 3B). The functional groups that decorate the DEO macrolactone ring alternatively contact residues of the abovementioned set, depending on the position, the orientation, and the conformation adopted by DEO in the active site of each open monomer. Electron density accounting for the sixth coordinate water ligand, typical of a low spin enzyme, was observed in four out of six total open monomers. It is placed at about 2–2.5 Å distance from the heme iron and the substrate ligand, forming hydrogen bonds with the backbone carbonyl of A244 on helix I and with the aglycon substrate, involving the C1-carbonyl or the C3-OH or the C5-OH groups, depending on the monomer (Figure S3A). Some extent of conformational flexibility of the substrate was also described for 6DEB in the open OleP-6DEB structure, but in all the monomers, the macrolide adopts a unique orientation, exposing the C8-C8a bond to the heme iron, with the carbonyl at C9 of the macrolactone ring forming a hydrogen bond with the sixth coordinating water molecule [20]. Structural comparison between open OleP–DEO and OleP-6DEB shows that the ethyl group at C13 in the place of a methyl group reduces the mobility of 6DEB with respect to DEO, since it is hosted in a hydrophobic niche formed by L396 on β-hairpin β_4_, M83 and F84 on the BC loop, and V291 on the β-hairpin β_3_ (Figure S4A and [20]). The interaction with this hydrophobic pocket constrains the conformation of 6DEB at the early stage of binding, when OleP is still in an open state, favoring an overall position in which it exposes the target C8-C8a bond to the heme iron, differently to DEO in open OleP.

In closed OleP, the mobility of DEO in the active site is strongly reduced: the closure of the FG helical region over the heme pocket decreases the volume of the active site chamber, extending the number of contacts formed by the substrate and leading to the structuring of solvent molecules into a small cavity lined by the I helix, the BC loop, and the substrate itself that also contribute to DEO binding and positioning (Figure S3B); we will refer to this small pocket as the solvent cavity. 

In the closed state, the substrate displaces the sixth axial water ligand, with the macrolactone ring adopting the correct orientation where the C8-C8a bond is parallel and exposed to the heme plane, at a distance of about 4–5 Å from the iron, which is compatible with an initial attack of C8-C8a by activated oxygen species formed at the heme iron (Figure 3C and Figure S3B). In this position, DEO induces the formation of the helix I cleft, showing the conserved catalytical T248 lining a water channel that might enable proton delivery during OleP catalysis (Figure S5). A critical contribution to the correct positioning of DEO is provided by carbonyl–aromatic interactions that involve the carbonyl at C1 sandwiched between F296 on β-hairpin β_3_ and F84 on the BC loop. Substrate binding is also mediated by van der Waals interactions established with residues on the BC loop (M83, F84, V93, L94), on the C-terminus of helix F (M178, L179) and on the N-terminus of helix I (S240, I243, A244, T248), on β-hairpin β_3_ (V291, G294, S295, F296) and on β-hairpin β_4_ (L396, I397) (Figure 3C). Remarkably, while 6DEB displays a unique conformation in closed OleP regardless of the crystallization conditions, DEO adopts slightly different conformations in the closed monomers, yet all correctly oriented, in low salt conditions (Figure S3B). Only higher ionic strength conditions enable a unique positioning of DEO in the active site. In the latter conditions, a network of hydrogen bonds that forms between the C3-OH and the C5-OH of DEO and solvent molecules trapped in the small cavity, the solvent cavity, of the active site, constrains the substrate position and bridges its contacts with the enzyme on regions directly involved in the open-to-closed transition (Figure 3B). This structural feature involves formate ions and/or water molecules depending on the monomer, that accumulate in the cavity lined by the I helix, the BC loop, and the substrate aglycone, mediating the interaction with OleP residues at the BC loop (E89), helix F (M178), helix G (Q193), and helix I (N236, S240). These five residues represent contact points located on key regions of structural elements that are crucial for the conformational transition, such as the BC loop, the F and the G helices and the N-terminus of helix I (Figure 3A). The network of hydrogen bonds extends from the solvent cavity towards residues E233 and M237 on the helix I and the BC loop (T86, D91, V93), the C-terminus of helix F (M178, S180). Although not determinant for triggering the closure, these water/formate-mediated interactions contribute to the stabilization of the closed state. Notably, the position adopted by the solvent molecules in the closed OleP–DEO approximates the ones adopted by water detected in the same area of the closed OleP-6DEB structure, highlighting their structural significance (Figure S6). 

The structural data herein described provide an explanation for the smaller absorbance shift and the lower affinity observed in the equilibrium binding experiments of DEO with respect to 6DEB. The difference in structure between 6DEB and DEO, although limited to just one additional methyl group at the C13 substituent of the macrolactone ring of 6DEB, has a double effect. On one side, the hydrophobic constrain at C13 of the macrolide facilitates the correct positioning of 6DEB when it enters OleP in the open state. The absence of this constrain in DEO increases its freedom of movement within the active site of open OleP that might result in an overall minor effect of this *Type I* ligand to the spin-state shift of the heme iron, consequently reducing the magnitude of the spectral changes observed. Our previous study demonstrated that the entrance of the substrate in the OleP active site alters the spin-state equilibrium, even when the enzyme is in the open state with the sixth coordinating water molecule still bound to the heme iron, resulting in an initial blue spectral shift of the Soret band that completes when the enzyme closes [20]. Therefore, the higher flexibility of DEO might be the cause of the difference in the absorbance shift observed when binding with DEO and 6DEB is compared. On the other side, the establishment of additional and/or closer van der Waals interactions at C13 of 6DEB in both the open and closed conformers of OleP might contribute to the ~1.2 kcal/mol stabilization of the OleP–6DEB complex with respect to one formed with DEO (Figure S4), with every single van der Waals contact contributing for about 0.5–1 kcal/mol to the overall free energy, depending on the distance between atoms [35].

A further interesting aspect that emerges from the structural analysis of OleP–DEO is that even the physiological aglycone substrate does not induce the closure of the OleP active site in the full population of the enzyme, at least in the OleP–DEO_LS_ crystal form. This observation, combined with the crystallographic study reported for OleP bound to 6DEB [20], leads to the more general conclusion that an aglycone substrate is not sufficient to shift predominantly the open-closed equilibrium of OleP towards the closed and catalytically competent state, when the ionic strength approximates the physiological one (I = 0.15–0.2 M). Higher ionic strength (I = 4 M) shifts the equilibrium towards the closed state in crystallo. In an effort to investigate such effect in solution, we performed equilibrium binding experiments by titrating the enzyme bound to DEO and to 6DEB with increasing concentration of sodium formate. We observed that increasing the ionic strength determines in both cases an increase in the absorbance at 382–388 nm and a decrease at 417 nm (Figure S7). This result indicates that in solution, an increase in ionic strength affects the equilibrium between low- and high-spin populations of OleP bound to DEO or 6DEB, possibly by enhancing OleP closure which allows the aglycone substrate to displace the sixth coordinating water molecule, therefore stabilizing the high-spin-state, as observed in crystallo (Figure 3C).

### 3.3. Docking of L-O-DEO and Crystallographic Structure of OleP Bound to DEO and L-Rhamnose

**Figure 4:**
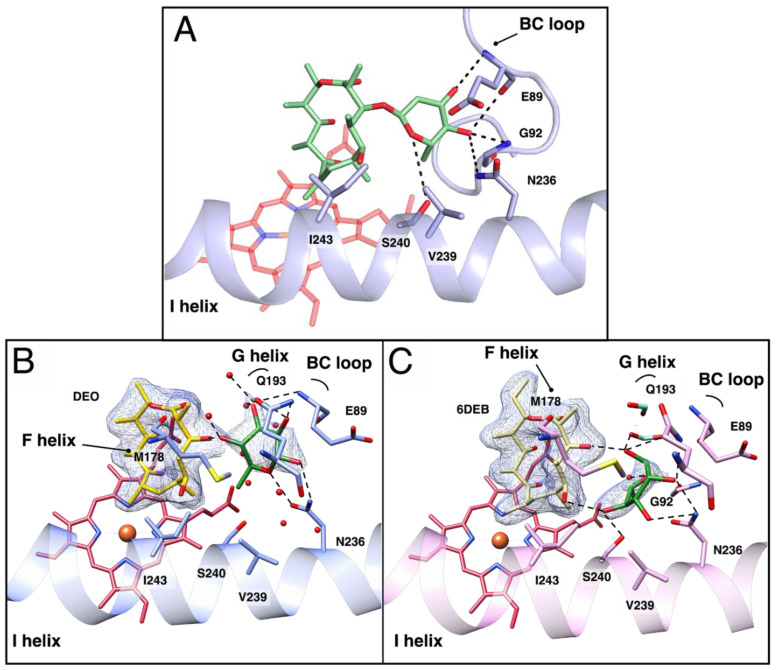
*In silico* docking of L-O-DEO and structures of OleP-aglycones bound to L-rhamnose. (**A**). Zoom on the active site of closed OleP (blue) into which L-O-DEO (lime green sticks) was computationally docked using AutoDock Vina 1.1.2. Residues forming direct hydrogen bonds (dashed lines) with the olivosyl moiety of L-O-DEO, placed at a distance ranging between 2.4 and 4 Å, are labeled. (**B**,**C**). Close up views of the active site OleP–DEO-rhamnose (panel **B**, blue) and OleP–6DEB–rhamnose (panel **C**, pink). Residues and solvent molecules (water, red spheres; formate ions, aquamarine sticks) within 5 Å from L-rhamnose are displayed. Dashed lines represent hydrogen bonds. Secondary structural elements and amino acids are labeled. In both panels, the electron density map (2F_o_–F_c_) contoured at 1 σ around L-rhamnose (green sticks) and DEO (yellow sticks, panel **B**) or 6DEB (khaki sticks, panel **C**) is shown as a blue mesh.

The analysis of the structural and functional data discussed above questions the hypothesis that DEO might be the optimal substrate for OleP: i) equilibrium binding experiments revealed the lower capability of OleP to bind DEO, one of the two natural substrates of the enzyme, with respect to 6DEB, a macrolide that in nature belongs to another streptomyces organism and that is produced during a different biosynthetic pathway; ii) the crystallographic analysis showed the limited capability of the aglycon intermediate of shifting the conformational equilibrium of OleP to the closed and catalytically competent state. An interesting feature that emerges from the crystallographic analysis of the OleP–DEO complex in the closed state is the solvent cavity that forms over the active site where a cluster of structured solvent molecules accumulates and mediates substrate–protein contacts, involving, among others, residues N236 and S240, where the largest displacement of the helix I occurs upon OleP closure (Figure 3A). The hydrophilic nature of the solvent cavity that is formed along the way to the open-to-closed transition supports the possibility that this site might host the glycosyl unit of L-O-DEO, the other substrate of OleP that is a monoglycosylated intermediate of oleandomycin biosynthesis. Since L-O-DEO is not commercially available and extremely complex to produce or synthesize, we performed a computational analysis to understand if it may represent a better substrate for OleP. In this analysis, L-O-DEO was docked into the active site of the closed OleP–DEO_HS_ structure using Autodock Vina 1.1.2 [33]. The energetically and sterically favored binding pose (ΔG = −10.6 kcal/mol) resulting from the clustered analysis shows L-O-DEO accommodating the L-olivosyl sugar moiety at the C3-OH of the ring in the solvent cavity. In this position, the glycosyl unit forms hydrogen bonds with residues of the BC loop (E89, G92) and at the N-terminus of helix I (N236, S240) (Figure 4A), that are only indirectly contacted by DEO through bridging waters and formate ions, as observed in crystallo. I243 and V239 on the I helix also contribute to the interaction by means of van der Waals contacts. Therefore, we hypothesize that a similar scenario might also occur in solution: when L-O-DEO binds the heme pocket of OleP, the macrolactone ring is arranged in a position and a conformation similar to the ones adopted by DEO and 6DEB [20] in the structure of OleP closed state, placing the L-olivosyl moiety within the cavity that is occupied by solvent molecules when the aglycone is bound to the active site. These findings support the idea that the monoglycosylated intermediate L-O-DEO would represent a better substrate for OleP, providing additional chemical and structural elements to interact with the active site of the enzyme.

As an experimental support of these computational findings and in an effort to recreate an OleP-bound-to-L-O-DEO scenario, we solved the crystal structure of an OleP ternary complex with either DEO or 6DEB and L-rhamnose obtained in high salt conditions, where OleP crystallizes in the closed form. We have chosen this sugar for two reasons: (i) it is chemically and stereochemically similar to the L-oleandrose sugar, differing only by the presence of a hydroxyl group at C4 (Figure 1B); (ii) previous studies showed OleP to be capable of catalyzing its reaction on a chimeric compound formed by the aglycone 6DEB attached at the C3 to a L-rhamnose unit, namely 3-O-rhamnosyl-6-deoxyerithronolide B [12]. Therefore, the concomitant binding of an aglycone substrate (DEO or 6DEB) and L-rhamnose to the active site of OleP might mimic the binding of L-O-DEO.

The omit maps of the ternary complex structures clearly revealed the presence of a bulky, partially defined, electron density within the solvent cavity of most of the OleP monomers in the asymmetric unit of both crystals. More in detail, positive density was found in monomer A, B, and D of OleP bound to DEO (Figure S8) and in monomers A, B, C, D, and E of OleP bound to 6DEB (Figure S9). Since this density was absent in the omit maps of the corresponding aglycone-bound OleP crystals where the L-rhamnose was not added, we interpreted it as a molecule of the sugar. Several attempts were made during the refinement to define the position and the occupancy of each rhamnose molecule in the monomers. However, the freedom of movement of the sugar molecule inside the cavity, which is only limited by sterically prohibited contacts, resulted in poorly defined electron densities of the ligand. Successful refinement gave an average of occupancy of 60% for each identified L-rhamnose molecule. Due to its flexibility, to the absence of chemical linkage to the aglycone and to the available room in the solvent cavity, L-rhamnose adopts different positions, orientations, and conformations inside each monomer, contacting via hydrogen bonds and van der Waals interactions: (i) OleP residues present in the cavity, (ii) the aglycone moiety bound to the active site at C3-OH and C5-OH, and (iii) solvent molecules (Figure 4B,C, Figures S10 and S11). Most contacts established in the crystal structures by L-rhamnose with OleP correspond to the ones observed *in silico* for the glycosyl moiety of docked L-O-DEO. These contacts include E89 and G92 on the BC loop, and N236, V239, S240, and I243 on the I helix. Moreover, the hydrogen bonds established with the sugar hydroxyl groups or the oxygen heteroatom by N236 and S240 were correctly predicted. L-rhamnose in the ternary complex establishes contacts with M178 on the F helix and Q193 on the G helix, which were absent in the docking simulation. All the abovementioned residues are located on secondary structural regions that participate in the open-to-closed structural transition.

## 4. Conclusions

The molecular basis of substrate binding and specificity in P450s constitutes the key tool to rationally modify and control their catalysis, expanding their repertoire of reactivities. In this work, the broad substrate specificity of the cytochrome P450 OleP was investigated. Structural and functional data of OleP in complex with the physiological aglycone substrate DEO reveal that this compound only loosely fits the active site of the enzyme. As a consequence, efficient binding of DEO that induces the structural changes required to close OleP and to shift the iron-spin equilibrium to the high state, preparing it for catalysis, occurs on a small fraction of enzyme molecules as supported by having only one-third of the protein copies in the asymmetric unit of the OleP–DEO_LS_ crystal form in the closed conformation. This leads us to hypothesize that although OleP is capable of catalyzing the reaction on the aglycone intermediate [12,20], the latter might not constitute the optimal substrate. Conversely, docking *in silico* of the alternative substrate L-O-DEO into the closed form of OleP shows that the monoglycosylated intermediate fully occupies the active site. The glycosyl unit at C3 could therefore be hosted in a cavity lined by the structural elements that mostly experience the open-to-closed conformational transition, forming direct hydrogen bonds with E89 and G92 (BC loop), and N236 and S240 (N-terminus of the internal helix I). However, in the absence of the sugar, this cavity is occupied by a cluster of structural solvent molecules that bridges the contact between the aglycone substrate and the enzyme together.

The structure of OleP bound to DEO and L-rhamnose, an analog of L-oleandrose, confirms that the cavity enables accommodation of a sugar and that most of the residues predicted by molecular docking interact with L-rhamnose, in compliance with the steric hindrance of the system. Moreover, the position experimentally observed for L-rhamnose is consistent with the glycosidic bond between L-oleandrose and the aglycone moiety. Therefore, we propose that optimal substrate binding to OleP could be achieved through the establishment of direct interactions between the N-terminus of the helix I and the sugar moiety of the monoglycosylated substrate. The substitution of dynamic solvent molecules with a rigid structural element covalently bound to the macrolactone ring could better stabilize the closed state, also preventing unregulated solvent access to the active site that would produce unintended reactions; the enzyme would be locked in the high spin-state conformation, competent for catalysis, maintained until the epoxidation reaction is complete.

Overall, our data show that the broad substrate specificity of OleP is supported by structural solvent molecules that compensate for the absence of a glycosyl unit when the oleandolide is bound. The role of ordered solvent molecules, in the active site of ligand-bound P450s, has been largely discussed in Conner et al. [36]: it is described as a mechanism adopted by multifunctional P450s to adapt the active site to non-specific ligands, increasing their versatility. However, in OleP, the glycosyl unit attached to the macrolide substrate may drive its specificity toward one ligand with respect to the other.

In light of the data presented in this work, one may then speculate that during oleandomycin biosynthesis, once the oleandolide is released, the low affinity and the catalytic efficiency of OleP towards DEO may favor its attack by OleG2, and therefore, epoxidation would occur more frequently after the first glycosylation step rather than before, making L-O-DEO a preferential substrate (Figure 1A).

In light of redirecting the epoxidase activity of OleP toward a broader range of molecules as a biocatalyst for the production of new chemicals and drugs [10,11], it is of interest to underline how an additional methyl group at the C13 substituent of the aglycone substrate, as in 6DEB, is sufficient to increases the binding affinity of OleP by one order of magnitude. This observation, together with the structural information herein reported, may provide guidance in the choice of new target molecules and/or in the rational engineering of OleP to redirect its activity towards alternative substrates.

## Figures and Tables

**Table 1:** Data collection, refinements, statistics, and validation. Highest-resolution shell is shown in parentheses.

Data Collection	OleP–DEO_LS_	OleP–DEO_HS_	OleP–DEO-Rhamnose	OleP–6DEB–Rhamnose
PDB ID	6ZI2	6ZHZ	6ZI7	6ZI3
Crystallization Conditions	0.2 M NaCl 0.1 M Tris·HCl pH 7.4 25% PEG 3350 (seeding)	4 M HCOONa (10% glycerol)	4.4 M HCOONa	4.2 M HCOONa (20% glycerol)
Space group	P1	C2	C2	C2
Unit cell (Å, °)	a = 112.07, b = 116.64, c = 125.17, α = 104.43, β = 104.25, γ = 113.91	a = 247.47, b = 111.22, c = 159.20, β = 129.4	a = 247.53, b = 110.68, c = 159.28, β = 129.5	a = 247.38, b = 111.15, c = 159.14, β = 129.4
Resolution (å)	39.89–2.87 (3.05–2.87)	37.7–2.20 (2.33–2.20)	50.0–2.11 (2.24–2.11)	50.0–1.96 (2.08–1.96)
Total measurements	411,472	574,285	1,262,482	1,251,578
Unique reflections	115,590	168,218	373,419	462,663
Completeness (%)	98.2 (95.9)	98.9 (97.3)	99.1 (99.0)	97.7 (96.7)
Redundancy	3.56 (3.64)	3.41 (3.37)	3.38 (3.30)	2.70 (2.54)
R_merge_^a^ (%)	16.5 (92.4)	5.6 (112.7)	7.2 (147.1)	6.4 (163.6)
cc/2 (%)	99.0 (68.8)	99.8 (51.4)	99.8 (48.4)	99.7 (36.2)
I/σ (I)	7.29 (1.36)	12.10 (1.05)	10.52 (0.72)	7.80 (0.52)
Wilson B-value (Å2)	44.1	44.9	56.7	53.5
**Refinement**				
Molecules per asymmetric unit	9	6	6	6
Resolution Range (Å)	39.89–2.93	37.73–2.2	47.92–2.28	48.09–2.08
R_work_^/^R_free_^b^ (%)	24.0/29.1	18.7/24.3	18.4/24.7	17.2/22.5
**Deviations from ideal geometry**				
Bond (Å)	0.011	0.012	0.013	0.011
Angles (°)	1.78	2.16	2.16	1.75
Ramachandran (%) Favored/allowed/outliers *	96.6/3.4/0.0	96.1/3.9/0.0	96.6/3.4/0.0	97.0/3.0/0.0
**Mean B-factors (Å^2^)**				
Protein	56.9	45.2	59.5	59.6
HEM/DEO/6DEB/RAM	51.3/53.5/-/-	31.7/44.7/-/-	43.1/46.8/-/54.6	42.8/-/47.2/75.7
H2O/Na/FMT/GOL/TRS	13.5/-/-/-/-	62.2/58.5/87.5/81.6/64.4	59.6/56.1/76.4/-	63.9/86.7/55.9/83.3/87.6
**RSCC (per monomer in the asymmetric unit)**				
DEO	0.88/0.96/0.95/0.91/0.88/0.88/0.88/0.88/0.80	0.97/0.96/0.97/0.95/0.95/0.95	0.97/0.97/0.97/0.97/0.96/0.96	
6DEB				0.98/0.93/0.96/0.93/0.97/0.97
RAM (bound to the solvent cavity)			0.91;0.86/0.81/-/0.85/-/-	0.87/0.80/0.86/0.87/0.85/-
**No of Atoms**				
Protein	26350	18824	19903	20575
HEM/DEO/6DEB/RAM	387/234/-/-	258/156/-/-	258/156/-/33	258/-/162/77^+^
H2O/Na^+^/FMT/GOL/TRS	96/-/-/-/-	763/5/249/90/24	1369/6/642/-/-	1145/7/609/36/24

^a^$R_{merge}=\underset{i}{\sum }\underset{j}{\sum }|\;I_{i,j}-\langle I_{j}\rangle |/\underset{i}{\sum }\underset{j}{\sum }I_{i,j}$, where *i* runs over multiple observations of the same intensity, and *j* runs over all crystallographically unique intensities. ^b^ $R_{work}=\sum^{\text{​}}\left|\left|F_{obs}\right|-\left|F_{calc}\right|\right|/\sum^{\text{​}}\left|F_{obs}\right|$, where |F_obs_| > 0. R_free_ is based on 5% of the data randomly selected and is not used in the refinement. * No prolines and glycines. ^+^ In OleP–6DEB–rhamnose, two out of seven total L-rhamnose molecules (RAM) found are bound to the external surface.

## Supplementary Materials

The following are available online at https://www.mdpi.com/2218-273X/10/10/1411/s1, Figure S1: OleP–DEO open and closed conformers, Figure S2: OleP–DEO vs. OleP-6DEB: structural comparison, Figure S3: DEO conformations in open and closed OleP, Figure S4: 6DEB vs. DEO in OleP, Figure S5: Helix I cleft, Figure S6: Solvent cavity in closed OleP–DEO and closed OleP-6DEB complexes: comparison, Figure S7: Effect of the ionic strength on the spin-state equilibrium in OleP bound to DEO and to 6DEB. Equilibrium binding experiments, Figure S8: Structure of OleP–DEO bound to L-rhamnose: omit maps, Figure S9: Structure of OleP-6DEB bound to L-rhamnose: omit maps, Figure S10: Structure of OleP–DEO bound to L-rhamnose, Figure S11: Structure of OleP-6DEB bound to L-rhamnose.
