# Supplementary material for: Dissecting the Cytochrome P450 OleP Substrate Specificity: Evidence for a Preferential Substrate
Source: Biomolecules. 2020 Oct 6;10(10):1411. doi: 10.3390/biom10101411 (PMC7600006; doi:10.3390/biom10101411)
Supplement: Supplementary file 1 [file biomolecules-10-01411-s001.zip › SupplMat_&_ValRep/6ZI7_D_1292109610_val-report-full_P1.pdf]

# Full wwPDB X-ray Structure Validation Report (i)

Jul 28, 2020 – 05:49 PM BST

| | | |
|---|---|---|
| PDB ID | : | 6ZI7 |
| Title | : | Crystal structure of OleP-oleandolide(DEO) bound to L-rhamnose |
| Deposited on | : | 2020-06-25 |
| Resolution | : | 2.28 Å(reported) |

This is a Full wwPDB X-ray Structure Validation Report.

This report is produced by the wwPDB biocuration pipeline after annotation of the structure.

We welcome your comments at *validation@mail.wwpdb.org*
A user guide is available at
https://www.wwpdb.org/validation/2017/XrayValidationReportHelp
with specific help available everywhere you see the (i) symbol.

CONFIDENTIAL VALIDATION REPORT

---

The following versions of software and data (see references (i)) were used in the production of this report:

| | | |
|---|---|---|
| MolProbity | : | 4.02b-467 |
| Mogul | : | 1.8.5 (274361), CSD as541be (2020) |
| Xtriage (Phenix) | : | 1.13 |
| EDS | : | 2.13 |
| buster-report | : | 1.1.7 (2018) |
| Percentile statistics | : | 20191225.v01 (using entries in the PDB archive December 25th 2019) |
| Refmac | : | 5.8.0158 |
| CCP4 | : | 7.0.044 (Gargrove) |
| Ideal geometry (proteins) | : | Engh & Huber (2001) |
| Ideal geometry (DNA, RNA) | : | Parkinson et al. (1996) |
| Validation Pipeline (wwPDB-VP) | : | 2.13 |

# 1 Overall quality at a glance (i)

The following experimental techniques were used to determine the structure:
*X-RAY DIFFRACTION*

The reported resolution of this entry is 2.28 Å.

Percentile scores (ranging between 0-100) for global validation metrics of the entry are shown in the following graphic. The table shows the number of entries on which the scores are based.

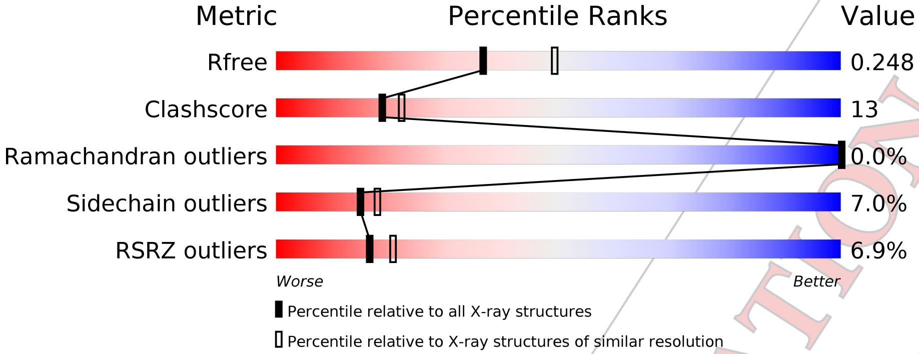


| Metric | Whole archive (#Entries) | Similar resolution (#Entries, resolution range(Å)) |
|---|---|---|
| $R_{free}$ | 130704 | 6980 (2.30-2.26) |
| Clashscore | 141614 | 7711 (2.30-2.26) |
| Ramachandran outliers | 138981 | 7597 (2.30-2.26) |
| Sidechain outliers | 138945 | 7598 (2.30-2.26) |
| RSRZ outliers | 127900 | 6849 (2.30-2.26) |

The table below summarises the geometric issues observed across the polymeric chains and their fit to the electron density. The red, orange, yellow and green segments on the lower bar indicate the fraction of residues that contain outliers for >=3, 2, 1 and 0 types of geometric quality criteria respectively. A grey segment represents the fraction of residues that are not modelled. The numeric value for each fraction is indicated below the corresponding segment, with a dot representing fractions <=5% The upper red bar (where present) indicates the fraction of residues that have poor fit to the electron density. The numeric value is given above the bar.

| Mol | Chain | Length | Quality of chain |
|---|---|---|---|
| 1 | A | 407 | % / 75% 21% • • |
| 1 | B | 407 | % / 79% 18% • • |
| 1 | C | 407 | % / 81% 14% • • |
| 1 | D | 407 | 6% / 74% 21% • • |
| 1 | E | 407 | 12% / 73% 21% • • |
| 1 | F | 407 | 20% / 69% 25% • • |

CONFIDENTIAL VALIDATION REPORT

The following table lists non-polymeric compounds, carbohydrate monomers and non-standard residues in protein, DNA, RNA chains that are outliers for geometric or electron-density-fit criteria:

| Mol | Type | Chain | Res | Chirality | Geometry | Clashes | Electron density |
|---|---|---|---|---|---|---|---|
| 4 | RAM | A | 503[A] | - | - | X | X |
| 4 | RAM | A | 503[B] | - | - | X | X |
| 4 | RAM | B | 513 | - | - | X | - |
| 4 | RAM | D | 503 | - | - | X | - |
| 5 | FMT | A | 510 | - | - | X | - |
| 5 | FMT | A | 511 | - | - | X | - |
| 5 | FMT | A | 517 | - | - | - | X |
| 5 | FMT | A | 529 | - | - | - | X |
| 5 | FMT | A | 531 | - | - | X | - |
| 5 | FMT | A | 546 | - | - | - | X |
| 5 | FMT | A | 549 | - | - | - | X |
| 5 | FMT | A | 550 | - | - | X | - |
| 5 | FMT | A | 563 | - | - | - | X |
| 5 | FMT | A | 565 | - | - | X | - |
| 5 | FMT | A | 574 | - | - | - | X |
| 5 | FMT | B | 503 | - | - | X | - |
| 5 | FMT | B | 511 | - | - | X | - |
| 5 | FMT | B | 522 | - | - | - | X |
| 5 | FMT | B | 534 | - | - | X | - |
| 5 | FMT | B | 538 | - | - | - | X |
| 5 | FMT | B | 539 | - | - | - | X |
| 5 | FMT | C | 507 | - | - | X | - |
| 5 | FMT | C | 528 | - | - | - | X |
| 5 | FMT | C | 539 | - | - | - | X |
| 5 | FMT | C | 543 | - | - | - | X |
| 5 | FMT | C | 544 | - | - | - | X |
| 5 | FMT | C | 545 | - | - | - | X |
| 5 | FMT | C | 551 | - | - | - | X |
| 5 | FMT | C | 552 | - | - | X | - |
| 5 | FMT | D | 504 | - | - | X | - |
| 5 | FMT | D | 517 | - | - | - | X |
| 5 | FMT | D | 527 | - | - | - | X |
| 5 | FMT | D | 528 | - | - | X | - |
| 5 | FMT | D | 530 | - | - | - | X |
| 5 | FMT | D | 536 | - | - | - | X |
| 5 | FMT | E | 501 | - | - | X | - |
| 5 | FMT | E | 509 | - | - | - | X |
| 5 | FMT | E | 511 | - | - | - | X |
| 5 | FMT | E | 517 | - | - | - | X |
| 5 | FMT | F | 505 | - | - | X | - |

*Continued on next page...*

CONFIDENTIAL VALIDATION REPORT

*Continued from previous page...*

| Mol | Type | Chain | Res | Chirality | Geometry | Clashes | Electron density |
|---|---|---|---|---|---|---|---|
| 5 | FMT | F | 509 | - | - | X | - |
| 5 | FMT | F | 514 | - | - | X | - |

CONFIDENTIAL VALIDATION REPORT

## 2 Entry composition (i)

There are 7 unique types of molecules in this entry. The entry contains 22307 atoms, of which 0 are hydrogens and 0 are deuteriums.

In the tables below, the ZeroOcc column contains the number of atoms modelled with zero occupancy, the AltConf column contains the number of residues with at least one atom in alternate conformation and the Trace column contains the number of residues modelled with at most 2 atoms.

- Molecule 1 is a protein called Cytochrome P-450.

| Mol | Chain | Residues | Atoms | | | | | ZeroOcc | AltConf | Trace |
|---|---|---|---|---|---|---|---|---|---|---|
| 1 | A | 396 | Total 3303 | C 2099 | N 587 | O 599 | S 18 | 0 | 36 | 0 |
| 1 | B | 400 | Total 3249 | C 2052 | N 574 | O 606 | S 17 | 0 | 22 | 0 |
| 1 | C | 400 | Total 3248 | C 2053 | N 577 | O 603 | S 15 | 0 | 22 | 0 |
| 1 | D | 400 | Total 3372 | C 2142 | N 603 | O 612 | S 15 | 0 | 42 | 0 |
| 1 | E | 397 | Total 3316 | C 2107 | N 591 | O 601 | S 17 | 0 | 36 | 0 |
| 1 | F | 398 | Total 3358 | C 2149 | N 588 | O 606 | S 15 | 0 | 46 | 0 |

- Molecule 2 is PROTOPORPHYRIN IX CONTAINING FE (three-letter code: HEM) (formula: $C_{34}H_{32}FeN_4O_4$).

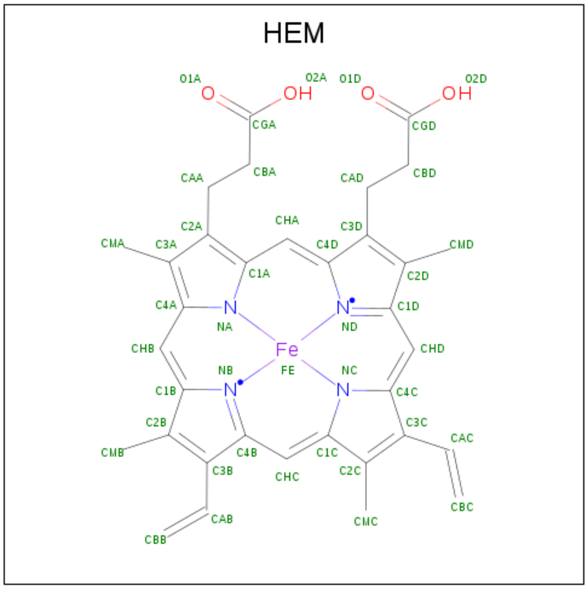


VALIDATION REPORT

| Mol | Chain | Residues | Atoms | ZeroOcc | AltConf |
|---|---|---|---|---|---|
| 2 | A | 1 | Total C Fe N O<br>43 34 1 4 4 | 0 | 0 |
| 2 | B | 1 | Total C Fe N O<br>43 34 1 4 4 | 0 | 0 |
| 2 | C | 1 | Total C Fe N O<br>43 34 1 4 4 | 0 | 0 |
| 2 | D | 1 | Total C Fe N O<br>43 34 1 4 4 | 0 | 0 |
| 2 | E | 1 | Total C Fe N O<br>43 34 1 4 4 | 0 | 0 |
| 2 | F | 1 | Total C Fe N O<br>43 34 1 4 4 | 0 | 0 |

- Molecule 3 is (3 {R},4 {S},5 {R},6 {S},7 {S},9 {S},11 {R},12 {S},13 {R},14 {R})-3,5,7,9 ,11,13,14-heptamethyl-4,6,12-tris(oxidanyl)-1-oxacyclotetradecane-2,10-dione (three-letter code: QR8) (formula: $C_{20}H_{36}O_6$) (labeled as "Ligand of Interest" by author).

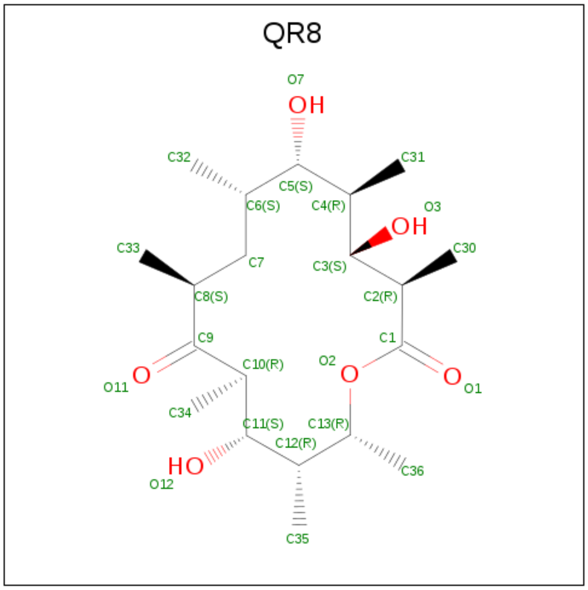


| Mol | Chain | Residues | Atoms | ZeroOcc | AltConf |
|---|---|---|---|---|---|
| 3 | A | 1 | Total C O<br>26 20 6 | 0 | 0 |
| 3 | B | 1 | Total C O<br>26 20 6 | 0 | 0 |
| 3 | C | 1 | Total C O<br>26 20 6 | 0 | 0 |
| 3 | D | 1 | Total C O<br>26 20 6 | 0 | 0 |
| 3 | E | 1 | Total C O<br>26 20 6 | 0 | 0 |

*Continued on next page...*

CONFIDENTIAL - VALIDATION REPORT

*Continued from previous page...*

| Mol | Chain | Residues | Atoms | ZeroOcc | AltConf |
|---|---|---|---|---|---|
| 3 | F | 1 | Total C O<br>26 20 6 | 0 | 0 |

- Molecule 4 is alpha-L-rhamnopyranose (three-letter code: RAM) (formula: $C_6H_{12}O_5$).

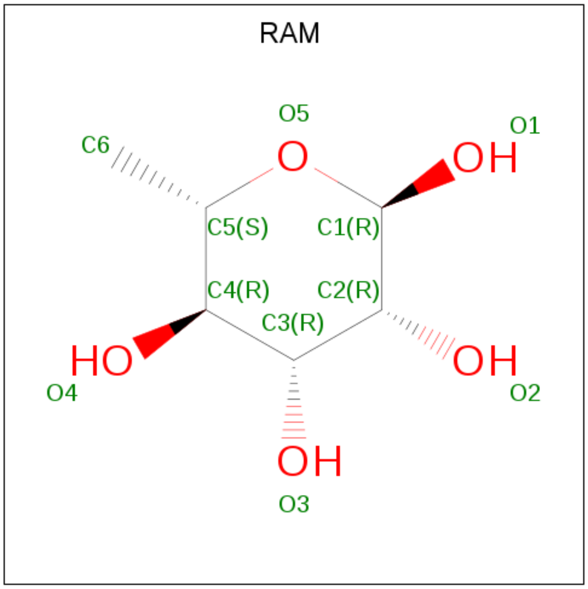


| Mol | Chain | Residues | Atoms | ZeroOcc | AltConf |
|---|---|---|---|---|---|
| 4 | A | 1 | Total C O<br>22 12 10 | 0 | 1 |
| 4 | B | 1 | Total C O<br>11 6 5 | 0 | 0 |
| 4 | D | 1 | Total C O<br>11 6 5 | 0 | 0 |

- Molecule 5 is FORMIC ACID (three-letter code: FMT) (formula: $CH_2O_2$).

CONFIDENTIAL VALIDATION REPORT

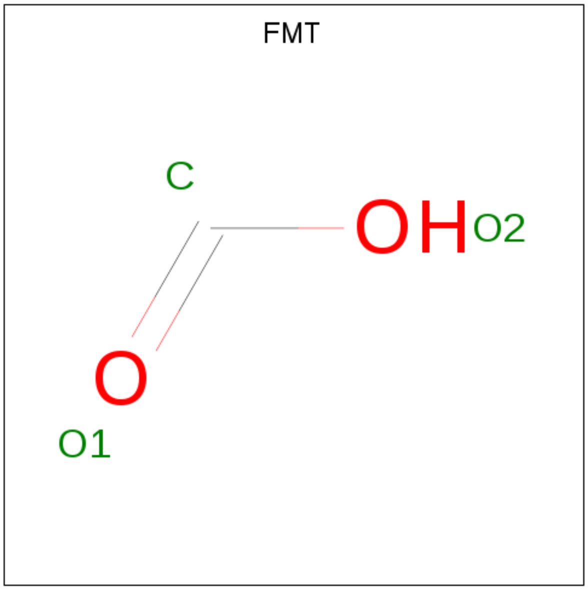


| Mol | Chain | Residues | Atoms | ZeroOcc | AltConf |
|---|---|---|---|---|---|
| 5 | A | 1 | Total C O<br>3 1 2 | 0 | 0 |
| 5 | A | 1 | Total C O<br>3 1 2 | 0 | 0 |
| 5 | A | 1 | Total C O<br>3 1 2 | 0 | 0 |
| 5 | A | 1 | Total C O<br>3 1 2 | 0 | 0 |
| 5 | A | 1 | Total C O<br>3 1 2 | 0 | 0 |
| 5 | A | 1 | Total C O<br>3 1 2 | 0 | 0 |
| 5 | A | 1 | Total C O<br>3 1 2 | 0 | 0 |
| 5 | A | 1 | Total C O<br>3 1 2 | 0 | 0 |
| 5 | A | 1 | Total C O<br>3 1 2 | 0 | 0 |
| 5 | A | 1 | Total C O<br>3 1 2 | 0 | 0 |
| 5 | A | 1 | Total C O<br>3 1 2 | 0 | 0 |
| 5 | A | 1 | Total C O<br>3 1 2 | 0 | 0 |
| 5 | A | 1 | Total C O<br>3 1 2 | 0 | 0 |
| 5 | A | 1 | Total C O<br>3 1 2 | 0 | 0 |

*Continued on next page...*

CONFIDENTIAL VALIDATION REPORT

*Continued from previous page...*

| Mol | Chain | Residues | Atoms | ZeroOcc | AltConf |
|---|---|---|---|---|---|
| 5 | A | 1 | Total C O<br>3 1 2 | 0 | 0 |
| 5 | A | 1 | Total C O<br>3 1 2 | 0 | 0 |
| 5 | A | 1 | Total C O<br>3 1 2 | 0 | 0 |
| 5 | A | 1 | Total C O<br>3 1 2 | 0 | 0 |
| 5 | A | 1 | Total C O<br>3 1 2 | 0 | 0 |
| 5 | A | 1 | Total C O<br>3 1 2 | 0 | 0 |
| 5 | A | 1 | Total C O<br>3 1 2 | 0 | 0 |
| 5 | A | 1 | Total C O<br>3 1 2 | 0 | 0 |
| 5 | A | 1 | Total C O<br>3 1 2 | 0 | 0 |
| 5 | A | 1 | Total C O<br>3 1 2 | 0 | 0 |
| 5 | A | 1 | Total C O<br>3 1 2 | 0 | 0 |
| 5 | A | 1 | Total C O<br>3 1 2 | 0 | 0 |
| 5 | A | 1 | Total C O<br>3 1 2 | 0 | 0 |
| 5 | A | 1 | Total C O<br>3 1 2 | 0 | 0 |
| 5 | A | 1 | Total C O<br>3 1 2 | 0 | 0 |
| 5 | A | 1 | Total C O<br>3 1 2 | 0 | 0 |
| 5 | A | 1 | Total C O<br>3 1 2 | 0 | 0 |
| 5 | A | 1 | Total C O<br>3 1 2 | 0 | 0 |
| 5 | A | 1 | Total C O<br>3 1 2 | 0 | 0 |
| 5 | A | 1 | Total C O<br>3 1 2 | 0 | 0 |
| 5 | A | 1 | Total C O<br>3 1 2 | 0 | 0 |

*Continued on next page...*

CONFIDENTIAL VALIDATION REPORT

*Continued from previous page...*

| Mol | Chain | Residues | Atoms | ZeroOcc | AltConf |
|---|---|---|---|---|---|
| 5 | A | 1 | Total C O<br>3 1 2 | 0 | 0 |
| 5 | A | 1 | Total C O<br>3 1 2 | 0 | 0 |
| 5 | A | 1 | Total C O<br>3 1 2 | 0 | 0 |
| 5 | A | 1 | Total C O<br>3 1 2 | 0 | 0 |
| 5 | A | 1 | Total C O<br>3 1 2 | 0 | 0 |
| 5 | A | 1 | Total C O<br>3 1 2 | 0 | 0 |
| 5 | A | 1 | Total C O<br>3 1 2 | 0 | 0 |
| 5 | A | 1 | Total C O<br>3 1 2 | 0 | 0 |
| 5 | A | 1 | Total C O<br>3 1 2 | 0 | 0 |
| 5 | A | 1 | Total C O<br>3 1 2 | 0 | 0 |
| 5 | A | 1 | Total C O<br>3 1 2 | 0 | 0 |
| 5 | A | 1 | Total C O<br>3 1 2 | 0 | 0 |
| 5 | A | 1 | Total C O<br>3 1 2 | 0 | 0 |
| 5 | A | 1 | Total C O<br>3 1 2 | 0 | 0 |
| 5 | A | 1 | Total C O<br>3 1 2 | 0 | 0 |
| 5 | A | 1 | Total C O<br>3 1 2 | 0 | 0 |
| 5 | A | 1 | Total C O<br>3 1 2 | 0 | 0 |
| 5 | A | 1 | Total C O<br>3 1 2 | 0 | 0 |
| 5 | A | 1 | Total C O<br>3 1 2 | 0 | 0 |
| 5 | A | 1 | Total C O<br>3 1 2 | 0 | 0 |
| 5 | A | 1 | Total C O<br>3 1 2 | 0 | 0 |

*Continued on next page...*

CONFIDENTIAL VALIDATION REPORT

*Continued from previous page...*

| Mol | Chain | Residues | Atoms | ZeroOcc | AltConf |
|---|---|---|---|---|---|
| 5 | A | 1 | Total C O<br>3 1 2 | 0 | 0 |
| 5 | A | 1 | Total C O<br>3 1 2 | 0 | 0 |
| 5 | A | 1 | Total C O<br>3 1 2 | 0 | 0 |
| 5 | A | 1 | Total C O<br>3 1 2 | 0 | 0 |
| 5 | A | 1 | Total C O<br>3 1 2 | 0 | 0 |
| 5 | A | 1 | Total C O<br>3 1 2 | 0 | 0 |
| 5 | A | 1 | Total C O<br>3 1 2 | 0 | 0 |
| 5 | A | 1 | Total C O<br>3 1 2 | 0 | 0 |
| 5 | A | 1 | Total C O<br>3 1 2 | 0 | 0 |
| 5 | A | 1 | Total C O<br>3 1 2 | 0 | 0 |
| 5 | A | 1 | Total C O<br>3 1 2 | 0 | 0 |
| 5 | A | 1 | Total C O<br>3 1 2 | 0 | 0 |
| 5 | A | 1 | Total C O<br>3 1 2 | 0 | 0 |
| 5 | A | 1 | Total C O<br>3 1 2 | 0 | 0 |
| 5 | B | 1 | Total C O<br>3 1 2 | 0 | 0 |
| 5 | B | 1 | Total C O<br>3 1 2 | 0 | 0 |
| 5 | B | 1 | Total C O<br>3 1 2 | 0 | 0 |
| 5 | B | 1 | Total C O<br>3 1 2 | 0 | 0 |
| 5 | B | 1 | Total C O<br>3 1 2 | 0 | 0 |
| 5 | B | 1 | Total C O<br>3 1 2 | 0 | 0 |
| 5 | B | 1 | Total C O<br>3 1 2 | 0 | 0 |

*Continued on next page...*

CONFIDENTIAL VALIDATION REPORT

*Continued from previous page...*

| Mol | Chain | Residues | Atoms | ZeroOcc | AltConf |
|---|---|---|---|---|---|
| 5 | B | 1 | Total C O<br>3 1 2 | 0 | 0 |
| 5 | B | 1 | Total C O<br>3 1 2 | 0 | 0 |
| 5 | B | 1 | Total C O<br>3 1 2 | 0 | 0 |
| 5 | B | 1 | Total C O<br>3 1 2 | 0 | 0 |
| 5 | B | 1 | Total C O<br>3 1 2 | 0 | 0 |
| 5 | B | 1 | Total C O<br>3 1 2 | 0 | 0 |
| 5 | B | 1 | Total C O<br>3 1 2 | 0 | 0 |
| 5 | B | 1 | Total C O<br>3 1 2 | 0 | 0 |
| 5 | B | 1 | Total C O<br>3 1 2 | 0 | 0 |
| 5 | B | 1 | Total C O<br>3 1 2 | 0 | 0 |
| 5 | B | 1 | Total C O<br>3 1 2 | 0 | 0 |
| 5 | B | 1 | Total C O<br>3 1 2 | 0 | 0 |
| 5 | B | 1 | Total C O<br>3 1 2 | 0 | 0 |
| 5 | B | 1 | Total C O<br>3 1 2 | 0 | 0 |
| 5 | B | 1 | Total C O<br>3 1 2 | 0 | 0 |
| 5 | B | 1 | Total C O<br>3 1 2 | 0 | 0 |
| 5 | B | 1 | Total C O<br>3 1 2 | 0 | 0 |
| 5 | B | 1 | Total C O<br>3 1 2 | 0 | 0 |
| 5 | B | 1 | Total C O<br>3 1 2 | 0 | 0 |
| 5 | B | 1 | Total C O<br>3 1 2 | 0 | 0 |
| 5 | B | 1 | Total C O<br>3 1 2 | 0 | 0 |

*Continued on next page...*

CONFIDENTIAL VALIDATION REPORT

*Continued from previous page...*

| Mol | Chain | Residues | Atoms | ZeroOcc | AltConf |
|---|---|---|---|---|---|
| 5 | B | 1 | Total C O<br>3 1 2 | 0 | 0 |
| 5 | B | 1 | Total C O<br>3 1 2 | 0 | 0 |
| 5 | B | 1 | Total C O<br>3 1 2 | 0 | 0 |
| 5 | B | 1 | Total C O<br>3 1 2 | 0 | 0 |
| 5 | B | 1 | Total C O<br>3 1 2 | 0 | 0 |
| 5 | B | 1 | Total C O<br>3 1 2 | 0 | 0 |
| 5 | B | 1 | Total C O<br>3 1 2 | 0 | 0 |
| 5 | B | 1 | Total C O<br>3 1 2 | 0 | 0 |
| 5 | C | 1 | Total C O<br>3 1 2 | 0 | 0 |
| 5 | C | 1 | Total C O<br>3 1 2 | 0 | 0 |
| 5 | C | 1 | Total C O<br>3 1 2 | 0 | 0 |
| 5 | C | 1 | Total C O<br>3 1 2 | 0 | 0 |
| 5 | C | 1 | Total C O<br>3 1 2 | 0 | 0 |
| 5 | C | 1 | Total C O<br>3 1 2 | 0 | 0 |
| 5 | C | 1 | Total C O<br>3 1 2 | 0 | 0 |
| 5 | C | 1 | Total C O<br>3 1 2 | 0 | 0 |
| 5 | C | 1 | Total C O<br>3 1 2 | 0 | 0 |
| 5 | C | 1 | Total C O<br>3 1 2 | 0 | 0 |
| 5 | C | 1 | Total C O<br>3 1 2 | 0 | 0 |
| 5 | C | 1 | Total C O<br>3 1 2 | 0 | 0 |
| 5 | C | 1 | Total C O<br>3 1 2 | 0 | 0 |

*Continued on next page...*

CONFIDENTIAL VALIDATION REPORT

*Continued from previous page...*

| Mol | Chain | Residues | Atoms | ZeroOcc | AltConf |
|---|---|---|---|---|---|
| 5 | C | 1 | Total C O<br>3 1 2 | 0 | 0 |
| 5 | C | 1 | Total C O<br>3 1 2 | 0 | 0 |
| 5 | C | 1 | Total C O<br>3 1 2 | 0 | 0 |
| 5 | C | 1 | Total C O<br>3 1 2 | 0 | 0 |
| 5 | C | 1 | Total C O<br>3 1 2 | 0 | 0 |
| 5 | C | 1 | Total C O<br>3 1 2 | 0 | 0 |
| 5 | C | 1 | Total C O<br>3 1 2 | 0 | 0 |
| 5 | C | 1 | Total C O<br>3 1 2 | 0 | 0 |
| 5 | C | 1 | Total C O<br>3 1 2 | 0 | 0 |
| 5 | C | 1 | Total C O<br>3 1 2 | 0 | 0 |
| 5 | C | 1 | Total C O<br>3 1 2 | 0 | 0 |
| 5 | C | 1 | Total C O<br>3 1 2 | 0 | 0 |
| 5 | C | 1 | Total C O<br>3 1 2 | 0 | 0 |
| 5 | C | 1 | Total C O<br>3 1 2 | 0 | 0 |
| 5 | C | 1 | Total C O<br>3 1 2 | 0 | 0 |
| 5 | C | 1 | Total C O<br>3 1 2 | 0 | 0 |
| 5 | C | 1 | Total C O<br>3 1 2 | 0 | 0 |
| 5 | C | 1 | Total C O<br>3 1 2 | 0 | 0 |
| 5 | C | 1 | Total C O<br>3 1 2 | 0 | 0 |
| 5 | C | 1 | Total C O<br>3 1 2 | 0 | 0 |
| 5 | C | 1 | Total C O<br>3 1 2 | 0 | 0 |

*Continued on next page...*

CONFIDENTIAL VALIDATION REPORT

*Continued from previous page...*

| Mol | Chain | Residues | Atoms | ZeroOcc | AltConf |
|---|---|---|---|---|---|
| 5 | C | 1 | Total C O<br>3 1 2 | 0 | 0 |
| 5 | C | 1 | Total C O<br>3 1 2 | 0 | 0 |
| 5 | C | 1 | Total C O<br>3 1 2 | 0 | 0 |
| 5 | C | 1 | Total C O<br>3 1 2 | 0 | 0 |
| 5 | C | 1 | Total C O<br>3 1 2 | 0 | 0 |
| 5 | C | 1 | Total C O<br>3 1 2 | 0 | 0 |
| 5 | C | 1 | Total C O<br>3 1 2 | 0 | 0 |
| 5 | C | 1 | Total C O<br>3 1 2 | 0 | 0 |
| 5 | C | 1 | Total C O<br>3 1 2 | 0 | 0 |
| 5 | C | 1 | Total C O<br>3 1 2 | 0 | 0 |
| 5 | C | 1 | Total C O<br>3 1 2 | 0 | 0 |
| 5 | C | 1 | Total C O<br>3 1 2 | 0 | 0 |
| 5 | C | 1 | Total C O<br>3 1 2 | 0 | 0 |
| 5 | C | 1 | Total C O<br>3 1 2 | 0 | 0 |
| 5 | C | 1 | Total C O<br>3 1 2 | 0 | 0 |
| 5 | D | 1 | Total C O<br>3 1 2 | 0 | 0 |
| 5 | D | 1 | Total C O<br>3 1 2 | 0 | 0 |
| 5 | D | 1 | Total C O<br>3 1 2 | 0 | 0 |
| 5 | D | 1 | Total C O<br>3 1 2 | 0 | 0 |
| 5 | D | 1 | Total C O<br>3 1 2 | 0 | 0 |
| 5 | D | 1 | Total C O<br>3 1 2 | 0 | 0 |

*Continued on next page...*

CONFIDENTIAL VALIDATION REPORT

*Continued from previous page...*

| Mol | Chain | Residues | Atoms | ZeroOcc | AltConf |
|---|---|---|---|---|---|
| 5 | D | 1 | Total C O<br>3 1 2 | 0 | 0 |
| 5 | D | 1 | Total C O<br>3 1 2 | 0 | 0 |
| 5 | D | 1 | Total C O<br>3 1 2 | 0 | 0 |
| 5 | D | 1 | Total C O<br>3 1 2 | 0 | 0 |
| 5 | D | 1 | Total C O<br>3 1 2 | 0 | 0 |
| 5 | D | 1 | Total C O<br>3 1 2 | 0 | 0 |
| 5 | D | 1 | Total C O<br>3 1 2 | 0 | 0 |
| 5 | D | 1 | Total C O<br>3 1 2 | 0 | 0 |
| 5 | D | 1 | Total C O<br>3 1 2 | 0 | 0 |
| 5 | D | 1 | Total C O<br>3 1 2 | 0 | 0 |
| 5 | D | 1 | Total C O<br>3 1 2 | 0 | 0 |
| 5 | D | 1 | Total C O<br>3 1 2 | 0 | 0 |
| 5 | D | 1 | Total C O<br>3 1 2 | 0 | 0 |
| 5 | D | 1 | Total C O<br>3 1 2 | 0 | 0 |
| 5 | D | 1 | Total C O<br>3 1 2 | 0 | 0 |
| 5 | D | 1 | Total C O<br>3 1 2 | 0 | 0 |
| 5 | D | 1 | Total C O<br>3 1 2 | 0 | 0 |
| 5 | D | 1 | Total C O<br>3 1 2 | 0 | 0 |
| 5 | D | 1 | Total C O<br>3 1 2 | 0 | 0 |
| 5 | D | 1 | Total C O<br>3 1 2 | 0 | 0 |
| 5 | D | 1 | Total C O<br>3 1 2 | 0 | 0 |

*Continued on next page...*

CONFIDENTIAL VALIDATION REPORT

*Continued from previous page...*

| Mol | Chain | Residues | Atoms | ZeroOcc | AltConf |
|---|---|---|---|---|---|
| 5 | D | 1 | Total C O<br>3 1 2 | 0 | 0 |
| 5 | D | 1 | Total C O<br>3 1 2 | 0 | 0 |
| 5 | D | 1 | Total C O<br>3 1 2 | 0 | 0 |
| 5 | D | 1 | Total C O<br>3 1 2 | 0 | 0 |
| 5 | D | 1 | Total C O<br>3 1 2 | 0 | 0 |
| 5 | D | 1 | Total C O<br>3 1 2 | 0 | 0 |
| 5 | E | 1 | Total C O<br>3 1 2 | 0 | 0 |
| 5 | E | 1 | Total C O<br>3 1 2 | 0 | 0 |
| 5 | E | 1 | Total C O<br>3 1 2 | 0 | 0 |
| 5 | E | 1 | Total C O<br>3 1 2 | 0 | 0 |
| 5 | E | 1 | Total C O<br>3 1 2 | 0 | 0 |
| 5 | E | 1 | Total C O<br>3 1 2 | 0 | 0 |
| 5 | E | 1 | Total C O<br>3 1 2 | 0 | 0 |
| 5 | E | 1 | Total C O<br>3 1 2 | 0 | 0 |
| 5 | E | 1 | Total C O<br>3 1 2 | 0 | 0 |
| 5 | E | 1 | Total C O<br>3 1 2 | 0 | 0 |
| 5 | E | 1 | Total C O<br>3 1 2 | 0 | 0 |
| 5 | E | 1 | Total C O<br>3 1 2 | 0 | 0 |
| 5 | E | 1 | Total C O<br>3 1 2 | 0 | 0 |
| 5 | E | 1 | Total C O<br>3 1 2 | 0 | 0 |
| 5 | F | 1 | Total C O<br>3 1 2 | 0 | 0 |

*Continued on next page...*

CONFIDENTIAL VALIDATION REPORT

*Continued from previous page...*

| Mol | Chain | Residues | Atoms | ZeroOcc | AltConf |
|---|---|---|---|---|---|
| 5 | F | 1 | Total C O<br>3 1 2 | 0 | 0 |
| 5 | F | 1 | Total C O<br>3 1 2 | 0 | 0 |
| 5 | F | 1 | Total C O<br>3 1 2 | 0 | 0 |
| 5 | F | 1 | Total C O<br>3 1 2 | 0 | 0 |
| 5 | F | 1 | Total C O<br>3 1 2 | 0 | 0 |
| 5 | F | 1 | Total C O<br>3 1 2 | 0 | 0 |
| 5 | F | 1 | Total C O<br>3 1 2 | 0 | 0 |
| 5 | F | 1 | Total C O<br>3 1 2 | 0 | 0 |
| 5 | F | 1 | Total C O<br>3 1 2 | 0 | 0 |
| 5 | F | 1 | Total C O<br>3 1 2 | 0 | 0 |
| 5 | F | 1 | Total C O<br>3 1 2 | 0 | 0 |

- Molecule 6 is SODIUM ION (three-letter code: NA) (formula: Na).

| Mol | Chain | Residues | Atoms | ZeroOcc | AltConf |
|---|---|---|---|---|---|
| 6 | D | 1 | Total Na<br>1 1 | 0 | 0 |
| 6 | E | 1 | Total Na<br>1 1 | 0 | 0 |
| 6 | B | 1 | Total Na<br>1 1 | 0 | 0 |
| 6 | C | 1 | Total Na<br>1 1 | 0 | 0 |
| 6 | A | 1 | Total Na<br>1 1 | 0 | 0 |
| 6 | F | 1 | Total Na<br>1 1 | 0 | 0 |

- Molecule 7 is water.

CONFIDENTIAL VALIDATION REPORT

| Mol | Chain | Residues | Atoms | | ZeroOcc | AltConf |
|---|---|---|---|---|---|---|
| 7 | A | 260 | Total 260 | O 260 | 0 | 1 |
| 7 | B | 281 | Total 281 | O 281 | 0 | 3 |
| 7 | C | 384 | Total 384 | O 384 | 0 | 3 |
| 7 | D | 158 | Total 158 | O 158 | 0 | 2 |
| 7 | E | 137 | Total 137 | O 137 | 0 | 4 |
| 7 | F | 135 | Total 135 | O 135 | 0 | 2 |

CONFIDENTIAL VALIDATION REPORT

# 3 Residue-property plots (i)

These plots are drawn for all protein, RNA, DNA and oligosaccharide chains in the entry. The first graphic for a chain summarises the proportions of the various outlier classes displayed in the second graphic. The second graphic shows the sequence view annotated by issues in geometry and electron density. Residues are color-coded according to the number of geometric quality criteria for which they contain at least one outlier: green = 0, yellow = 1, orange = 2 and red = 3 or more. A red dot above a residue indicates a poor fit to the electron density (RSRZ > 2). Stretches of 2 or more consecutive residues without any outlier are shown as a green connector. Residues present in the sample, but not in the model, are shown in grey.

- Molecule 1: Cytochrome P-450

Chain A:

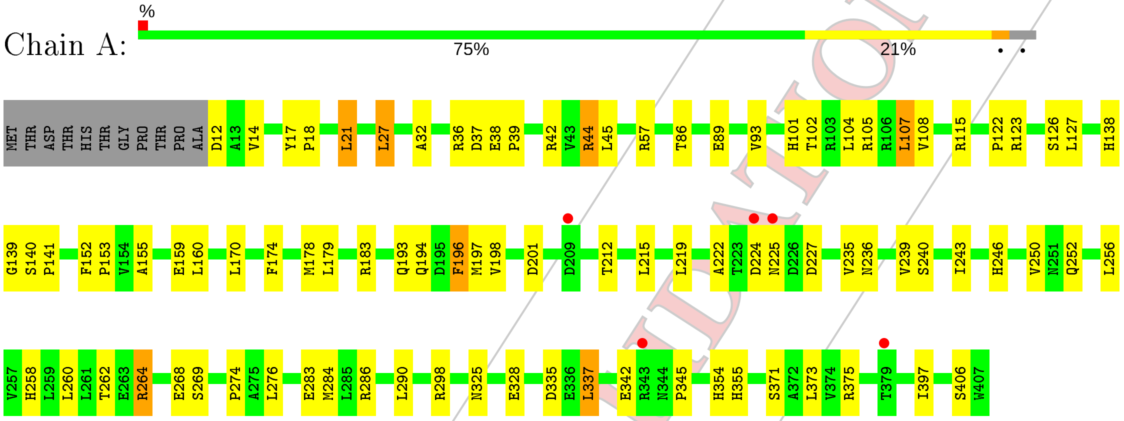


- Molecule 1: Cytochrome P-450

Chain B:

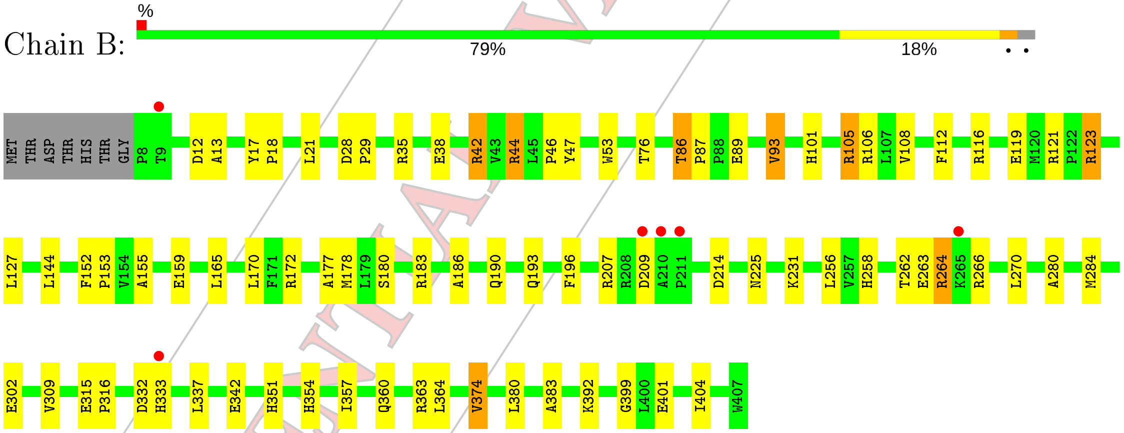


- Molecule 1: Cytochrome P-450

Chain C:

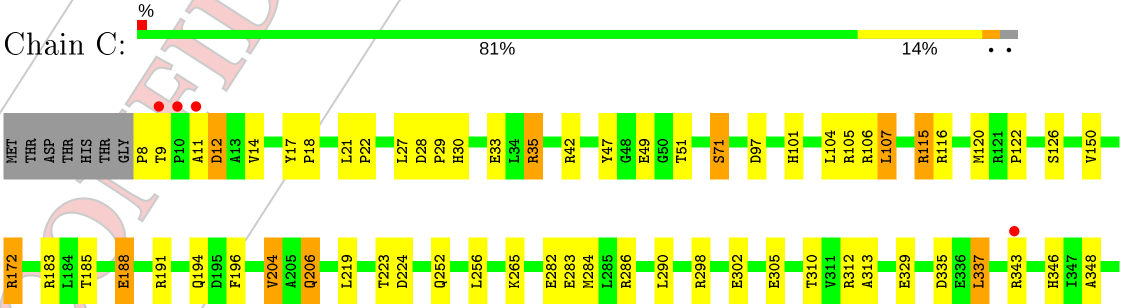


CONFIDENTIAL VALIDATION REPORT

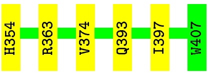


- Molecule 1: Cytochrome P-450

Chain D:

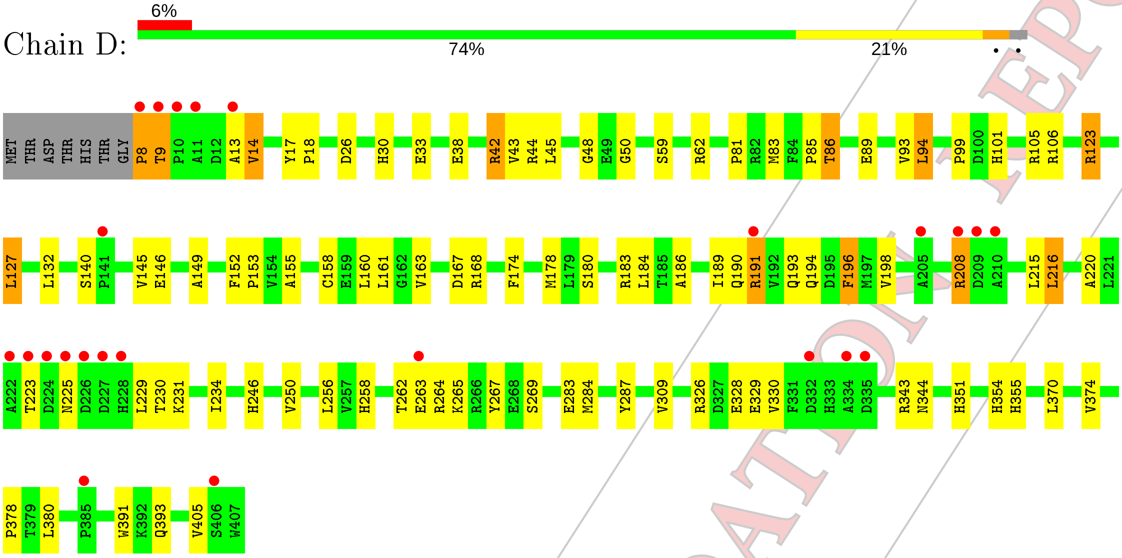


- Molecule 1: Cytochrome P-450

Chain E:

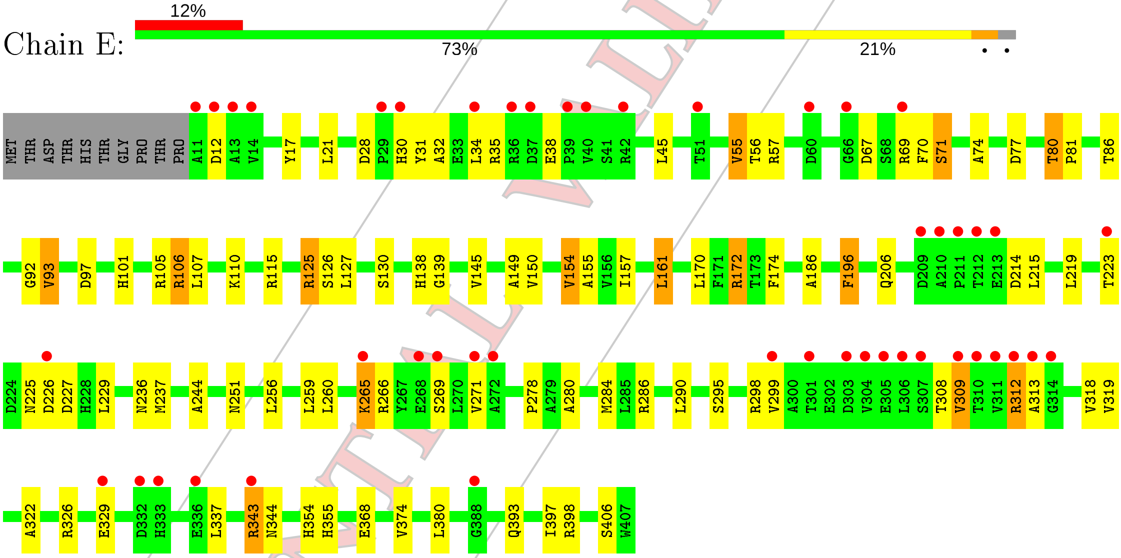


- Molecule 1: Cytochrome P-450

Chain F:

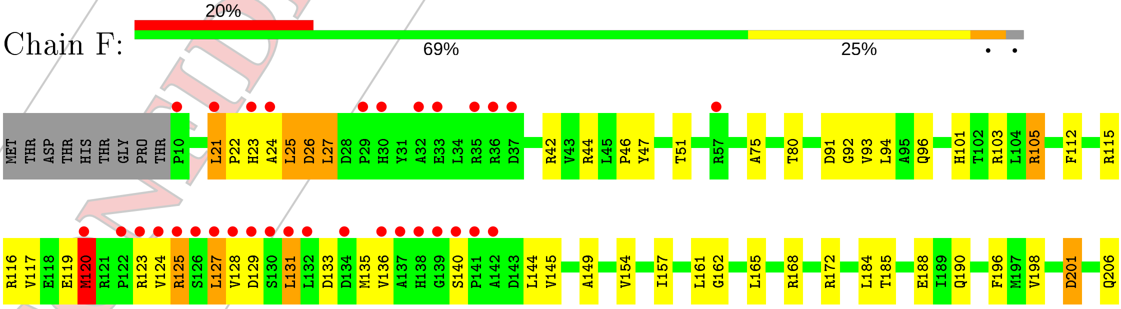


CONFIDENTIAL VALIDATION REPORT

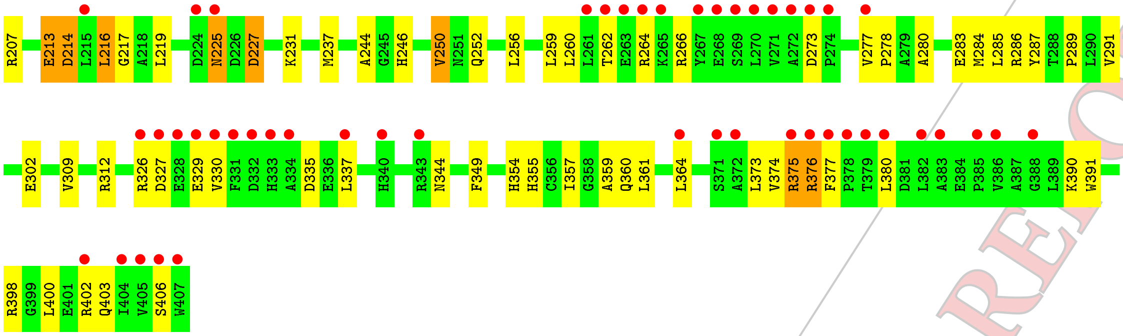


CONFIDENTIAL VALIDATION REPORT

# 4 Data and refinement statistics (i)

| Property | Value | Source |
|---|---|---|
| Space group | C 1 2 1 | Depositor |
| Cell constants<br>a, b, c, $\alpha$, $\beta$, $\gamma$ | 247.53Å 110.68Å 159.28Å<br>90.00° 129.46° 90.00° | Depositor |
| Resolution (Å) | 47.92 – 2.28<br>47.87 – 1.86 | Depositor<br>EDS |
| % Data completeness<br>(in resolution range) | 99.9 (47.92-2.28)<br>94.7 (47.87-1.86) | Depositor<br>EDS |
| $R_{merge}$ | 0.07 | Depositor |
| $R_{sym}$ | (Not available) | Depositor |
| $< I/\sigma(I) >$ [1] | 1.30 (at 1.87Å) | Xtriage |
| Refinement program | REFMAC 5.8.0238 | Depositor |
| R, $R_{free}$ | 0.184 , 0.247<br>0.190 , 0.248 | Depositor<br>DCC |
| $R_{free}$ test set | 13326 reflections (5.09%) | wwPDB-VP |
| Wilson B-factor (Å$^2$) | 56.7 | Xtriage |
| Anisotropy | 0.188 | Xtriage |
| Bulk solvent $k_{sol}$(e/Å$^3$), $B_{sol}$(Å$^2$) | 0.33 , 43.2 | EDS |
| L-test for twinning[2] | $< \|L\| >$ = 0.50, $< L^2 >$ = 0.33 | Xtriage |
| Estimated twinning fraction | 0.010 for -h-2*l,-k,l | Xtriage |
| $F_o$,$F_c$ correlation | 0.97 | EDS |
| Total number of atoms | 22307 | wwPDB-VP |
| Average B, all atoms (Å$^2$) | 60.0 | wwPDB-VP |

Xtriage's analysis on translational NCS is as follows: *The analyses of the Patterson function reveals a significant off-origin peak that is 40.86 % of the origin peak, indicating pseudo-translational symmetry. The chance of finding a peak of this or larger height randomly in a structure without pseudo-translational symmetry is equal to 2.5720e-04. The detected translational NCS is most likely also responsible for the elevated intensity ratio.*

[1] Intensities estimated from amplitudes.

[2] Theoretical values of $< |L| >$, $< L^2 >$ for acentric reflections are 0.5, 0.333 respectively for untwinned datasets, and 0.375, 0.2 for perfectly twinned datasets.

CONFIDENTIAL VALIDATION REPORT

# 5 Model quality (i)

## 5.1 Standard geometry (i)

Bond lengths and bond angles in the following residue types are not validated in this section: QR8, NA, FMT, RAM, HEM

The Z score for a bond length (or angle) is the number of standard deviations the observed value is removed from the expected value. A bond length (or angle) with $|Z| > 5$ is considered an outlier worth inspection. RMSZ is the root-mean-square of all Z scores of the bond lengths (or angles).

| Mol | Chain | Bond lengths | | Bond angles | |
|---|---|---|---|---|---|
| | | RMSZ | #\|Z\| >5 | RMSZ | #\|Z\| >5 |
| 1 | A | 0.86 | 0/3483 | 1.02 | 3/4732 (0.1%) |
| 1 | B | 0.89 | 1/3385 (0.0%) | 1.05 | 7/4606 (0.2%) |
| 1 | C | 0.90 | 3/3385 (0.1%) | 1.08 | 9/4606 (0.2%) |
| 1 | D | 0.84 | 0/3572 | 1.01 | 2/4852 (0.0%) |
| 1 | E | 0.81 | 0/3489 | 0.96 | 1/4744 (0.0%) |
| 1 | F | 0.85 | 2/3561 (0.1%) | 1.06 | 13/4843 (0.3%) |
| All | All | 0.86 | 6/20875 (0.0%) | 1.03 | 35/28383 (0.1%) |

Chiral center outliers are detected by calculating the chiral volume of a chiral center and verifying if the center is modelled as a planar moiety or with the opposite hand.A planarity outlier is detected by checking planarity of atoms in a peptide group, atoms in a mainchain group or atoms of a sidechain that are expected to be planar.

| Mol | Chain | #Chirality outliers | #Planarity outliers |
|---|---|---|---|
| 1 | F | 0 | 3 |

All (6) bond length outliers are listed below:

| Mol | Chain | Res | Type | Atoms | Z | Observed(Å) | Ideal(Å) |
|---|---|---|---|---|---|---|---|
| 1 | F | 26[A] | ASP | CA-C | 8.04 | 1.73 | 1.52 |
| 1 | F | 26[B] | ASP | CA-C | 8.04 | 1.73 | 1.52 |
| 1 | C | 188 | GLU | CD-OE1 | 5.81 | 1.32 | 1.25 |
| 1 | B | 119 | GLU | CD-OE1 | 5.61 | 1.31 | 1.25 |
| 1 | C | 71 | SER | CA-CB | -5.35 | 1.45 | 1.52 |
| 1 | C | 188 | GLU | CD-OE2 | 5.18 | 1.31 | 1.25 |

All (35) bond angle outliers are listed below:

| Mol | Chain | Res | Type | Atoms | Z | Observed($^o$) | Ideal($^o$) |
|---|---|---|---|---|---|---|---|
| 1 | F | 120[A] | MET | N-CA-CB | -14.58 | 84.36 | 110.60 |

*Continued on next page...*

CONFIDENTIAL VALIDATION REPORT

*Continued from previous page...*

| Mol | Chain | Res | Type | Atoms | Z | Observed(°) | Ideal(°) |
|---|---|---|---|---|---|---|---|
| 1 | F | 120[B] | MET | N-CA-CB | -14.58 | 84.36 | 110.60 |
| 1 | F | 26[A] | ASP | CA-C-O | -11.16 | 96.66 | 120.10 |
| 1 | F | 26[B] | ASP | CA-C-O | -11.16 | 96.66 | 120.10 |
| 1 | C | 35 | ARG | NE-CZ-NH2 | -10.81 | 114.89 | 120.30 |
| 1 | F | 26[A] | ASP | CB-CA-C | -8.36 | 93.68 | 110.40 |
| 1 | F | 26[B] | ASP | CB-CA-C | -8.36 | 93.68 | 110.40 |
| 1 | A | 105 | ARG | NE-CZ-NH2 | -8.25 | 116.18 | 120.30 |
| 1 | C | 363 | ARG | NE-CZ-NH1 | 7.97 | 124.28 | 120.30 |
| 1 | B | 363 | ARG | NE-CZ-NH1 | 7.51 | 124.06 | 120.30 |
| 1 | F | 25 | LEU | C-N-CA | -7.37 | 103.27 | 121.70 |
| 1 | F | 120[A] | MET | N-CA-C | 7.28 | 130.65 | 111.00 |
| 1 | F | 120[B] | MET | N-CA-C | 7.28 | 130.65 | 111.00 |
| 1 | C | 35 | ARG | NE-CZ-NH1 | 7.24 | 123.92 | 120.30 |
| 1 | E | 214 | ASP | CB-CA-C | -7.00 | 96.39 | 110.40 |
| 1 | B | 363 | ARG | NE-CZ-NH2 | -6.54 | 117.03 | 120.30 |
| 1 | F | 119 | GLU | C-N-CA | -6.47 | 105.52 | 121.70 |
| 1 | D | 106 | ARG | CB-CA-C | 6.46 | 123.33 | 110.40 |
| 1 | C | 172 | ARG | NE-CZ-NH2 | 6.42 | 123.51 | 120.30 |
| 1 | F | 26[A] | ASP | N-CA-CB | -6.28 | 99.29 | 110.60 |
| 1 | F | 26[B] | ASP | N-CA-CB | -6.28 | 99.29 | 110.60 |
| 1 | F | 105 | ARG | NE-CZ-NH2 | -6.01 | 117.30 | 120.30 |
| 1 | B | 93 | VAL | CB-CA-C | -6.00 | 100.00 | 111.40 |
| 1 | A | 375 | ARG | CG-CD-NE | -5.84 | 99.54 | 111.80 |
| 1 | C | 105 | ARG | NE-CZ-NH2 | -5.83 | 117.38 | 120.30 |
| 1 | C | 298 | ARG | NE-CZ-NH1 | 5.70 | 123.15 | 120.30 |
| 1 | D | 8 | PRO | N-CA-CB | 5.64 | 110.07 | 103.30 |
| 1 | C | 286 | ARG | NE-CZ-NH2 | -5.63 | 117.48 | 120.30 |
| 1 | C | 363 | ARG | NE-CZ-NH2 | -5.53 | 117.53 | 120.30 |
| 1 | C | 47 | TYR | CB-CG-CD1 | 5.50 | 124.30 | 121.00 |
| 1 | B | 183 | ARG | CB-CA-C | -5.42 | 99.55 | 110.40 |
| 1 | B | 105 | ARG | CB-CG-CD | -5.40 | 97.56 | 111.60 |
| 1 | B | 302 | GLU | CB-CA-C | -5.32 | 99.75 | 110.40 |
| 1 | B | 44 | ARG | NE-CZ-NH2 | 5.31 | 122.96 | 120.30 |
| 1 | A | 375 | ARG | NE-CZ-NH1 | -5.23 | 117.68 | 120.30 |

There are no chirality outliers.

All (3) planarity outliers are listed below:

| Mol | Chain | Res | Type | Group |
|---|---|---|---|---|
| 1 | F | 213 | GLU | Peptide |
| 1 | F | 26[A] | ASP | Mainchain |
| 1 | F | 26[B] | ASP | Mainchain |

CONFIDENTIAL VALIDATION REPORT

## 5.2 Too-close contacts (i)

In the following table, the Non-H and H(model) columns list the number of non-hydrogen atoms and hydrogen atoms in the chain respectively. The H(added) column lists the number of hydrogen atoms added and optimized by MolProbity. The Clashes column lists the number of clashes within the asymmetric unit, whereas Symm-Clashes lists symmetry related clashes.

| Mol | Chain | Non-H | H(model) | H(added) | Clashes | Symm-Clashes |
|---|---|---|---|---|---|---|
| 1 | A | 3303 | 0 | 3379 | 104 | 0 |
| 1 | B | 3249 | 0 | 3256 | 61 | 1 |
| 1 | C | 3248 | 0 | 3258 | 59 | 0 |
| 1 | D | 3372 | 0 | 3458 | 98 | 0 |
| 1 | E | 3316 | 0 | 3380 | 74 | 0 |
| 1 | F | 3358 | 0 | 3479 | 115 | 0 |
| 2 | A | 43 | 0 | 30 | 4 | 0 |
| 2 | B | 43 | 0 | 30 | 7 | 0 |
| 2 | C | 43 | 0 | 30 | 3 | 0 |
| 2 | D | 43 | 0 | 30 | 4 | 0 |
| 2 | E | 43 | 0 | 30 | 3 | 0 |
| 2 | F | 43 | 0 | 30 | 4 | 0 |
| 3 | A | 26 | 0 | 0 | 3 | 0 |
| 3 | B | 26 | 0 | 0 | 1 | 0 |
| 3 | C | 26 | 0 | 0 | 0 | 0 |
| 3 | D | 26 | 0 | 0 | 2 | 0 |
| 3 | E | 26 | 0 | 0 | 0 | 0 |
| 3 | F | 26 | 0 | 0 | 0 | 0 |
| 4 | A | 22 | 0 | 23 | 34 | 0 |
| 4 | B | 11 | 0 | 12 | 11 | 0 |
| 4 | D | 11 | 0 | 12 | 14 | 0 |
| 5 | A | 210 | 0 | 71 | 21 | 1 |
| 5 | B | 108 | 0 | 36 | 9 | 0 |
| 5 | C | 147 | 0 | 49 | 9 | 0 |
| 5 | D | 99 | 0 | 33 | 8 | 0 |
| 5 | E | 42 | 0 | 14 | 2 | 0 |
| 5 | F | 36 | 0 | 12 | 10 | 0 |
| 6 | A | 1 | 0 | 0 | 0 | 0 |
| 6 | B | 1 | 0 | 0 | 0 | 0 |
| 6 | C | 1 | 0 | 0 | 0 | 0 |
| 6 | D | 1 | 0 | 0 | 0 | 0 |
| 6 | E | 1 | 0 | 0 | 0 | 0 |
| 6 | F | 1 | 0 | 0 | 0 | 0 |
| 7 | A | 260 | 0 | 0 | 9 | 0 |
| 7 | B | 281 | 0 | 0 | 14 | 0 |
| 7 | C | 384 | 0 | 0 | 17 | 0 |
| 7 | D | 158 | 0 | 0 | 6 | 0 |

*Continued on next page...*

CONFIDENTIAL VALIDATION REPORT

*Continued from previous page...*

| Mol | Chain | Non-H | H(model) | H(added) | Clashes | Symm-Clashes |
|---|---|---|---|---|---|---|
| 7 | E | 137 | 0 | 0 | 1 | 0 |
| 7 | F | 135 | 0 | 0 | 9 | 0 |
| All | All | 22307 | 0 | 20652 | 544 | 1 |

The all-atom clashscore is defined as the number of clashes found per 1000 atoms (including hydrogen atoms). The all-atom clashscore for this structure is 13.

All (544) close contacts within the same asymmetric unit are listed below, sorted by their clash magnitude.

| Atom-1 | Atom-2 | Interatomic distance (Å) | Clash overlap (Å) |
|---|---|---|---|
| 1:E:93[A]:VAL:HG11 | 1:E:237[A]:MET:CE | 1.54 | 1.37 |
| 1:F:120[A]:MET:CE | 1:F:361[A]:LEU:HD21 | 1.69 | 1.22 |
| 1:A:197[A]:MET:CE | 1:A:235[A]:VAL:HG23 | 1.73 | 1.19 |
| 1:B:89:GLU:CB | 4:B:513:RAM:H2 | 1.71 | 1.17 |
| 1:A:197[A]:MET:CE | 1:A:235[A]:VAL:CG2 | 2.23 | 1.17 |
| 7:A:715:HOH:O | 1:D:13:ALA:HB3 | 1.41 | 1.16 |
| 1:B:89:GLU:HB3 | 4:B:513:RAM:H2 | 1.25 | 1.11 |
| 1:A:197[A]:MET:HE1 | 1:A:235[A]:VAL:CG2 | 1.82 | 1.09 |
| 1:D:89:GLU:HB2 | 4:D:503:RAM:H62 | 1.30 | 1.08 |
| 1:E:343[A]:ARG:HG2 | 1:E:343[A]:ARG:HH11 | 0.95 | 1.08 |
| 1:F:120[A]:MET:HE2 | 1:F:361[A]:LEU:HD21 | 1.17 | 1.08 |
| 1:F:185[A]:THR:OG1 | 7:F:601[A]:HOH:O | 1.72 | 1.06 |
| 1:C:30[A]:HIS:ND1 | 1:C:33[A]:GLU:OE2 | 1.90 | 1.04 |
| 1:D:208[A]:ARG:HH11 | 1:D:208[A]:ARG:CB | 1.72 | 1.03 |
| 1:A:197[A]:MET:HE2 | 1:A:235[A]:VAL:CG2 | 1.87 | 1.03 |
| 1:E:93[A]:VAL:HG11 | 1:E:237[A]:MET:HE1 | 1.05 | 1.02 |
| 1:A:193:GLN:HE21 | 4:A:503[A]:RAM:H1 | 1.23 | 1.00 |
| 1:B:42[B]:ARG:HG2 | 1:B:42[B]:ARG:HH21 | 1.23 | 0.99 |
| 1:E:343[A]:ARG:HG2 | 1:E:343[A]:ARG:NH1 | 1.74 | 0.98 |
| 1:F:120[A]:MET:HE1 | 1:F:361[A]:LEU:HD11 | 1.43 | 0.98 |
| 1:E:172:ARG:NH1 | 7:E:601:HOH:O | 1.96 | 0.97 |
| 1:E:93[A]:VAL:CG1 | 1:E:237[A]:MET:CE | 2.41 | 0.96 |
| 1:F:21:LEU:HD13 | 1:F:23[B]:HIS:CE1 | 2.01 | 0.95 |
| 1:E:280:ALA:O | 1:E:284[A]:MET:HG3 | 1.68 | 0.94 |
| 1:A:197[A]:MET:HE2 | 1:A:235[A]:VAL:HG23 | 1.47 | 0.93 |
| 1:C:335:ASP:HB2 | 5:C:552:FMT:O2 | 1.69 | 0.93 |
| 1:D:123[A]:ARG:HH11 | 1:D:123[A]:ARG:HG3 | 1.34 | 0.92 |
| 1:D:30[B]:HIS:ND1 | 1:D:33[B]:GLU:OE2 | 2.03 | 0.92 |
| 1:B:89:GLU:HB2 | 4:B:513:RAM:H2 | 1.51 | 0.92 |
| 1:E:93[A]:VAL:CG1 | 1:E:237[A]:MET:SD | 2.57 | 0.92 |
| 1:A:86:THR:OG1 | 4:A:503[A]:RAM:H63 | 1.68 | 0.91 |

*Continued on next page...*

CONFIDENTIAL VALIDATION REPORT

*Continued from previous page...*

| Atom-1 | Atom-2 | Interatomic distance (Å) | Clash overlap (Å) |
|---|---|---|---|
| 1:D:193:GLN:CD | 4:D:503:RAM:H63 | 1.90 | 0.91 |
| 1:E:93[A]:VAL:HG11 | 1:E:237[A]:MET:SD | 2.11 | 0.91 |
| 1:D:123[A]:ARG:CG | 1:D:123[A]:ARG:HH11 | 1.83 | 0.90 |
| 1:B:42[B]:ARG:CG | 1:B:42[B]:ARG:HH21 | 1.85 | 0.90 |
| 4:A:503[B]:RAM:O2 | 5:A:565:FMT:C | 2.20 | 0.89 |
| 1:A:193:GLN:NE2 | 4:A:503[A]:RAM:H1 | 1.86 | 0.89 |
| 1:D:105:ARG:NH1 | 1:D:355:HIS:O | 2.06 | 0.88 |
| 1:C:101[B]:HIS:CE1 | 1:C:354[B]:HIS:CD2 | 2.62 | 0.87 |
| 1:E:172:ARG:HH11 | 1:E:172:ARG:HG2 | 1.35 | 0.87 |
| 1:F:21:LEU:HD23 | 1:F:22:PRO:HD2 | 1.56 | 0.87 |
| 1:C:256:LEU:HD22 | 1:C:284:MET:HB3 | 1.57 | 0.87 |
| 1:C:185[B]:THR:HG22 | 7:C:617:HOH:O | 1.75 | 0.86 |
| 1:F:237[B]:MET:HA | 1:F:237[B]:MET:HE3 | 1.58 | 0.86 |
| 1:A:345:PRO:HB3 | 1:D:8:PRO:HA | 1.56 | 0.86 |
| 1:B:89:GLU:HB2 | 4:B:513:RAM:C2 | 2.06 | 0.85 |
| 1:E:93[A]:VAL:CG1 | 1:E:237[A]:MET:HE1 | 2.00 | 0.85 |
| 1:A:86:THR:OG1 | 4:A:503[A]:RAM:C6 | 2.24 | 0.85 |
| 1:D:89:GLU:HB2 | 4:D:503:RAM:C6 | 2.06 | 0.84 |
| 1:E:343[A]:ARG:HH11 | 1:E:343[A]:ARG:CG | 1.85 | 0.84 |
| 1:A:197[A]:MET:HE2 | 1:A:235[A]:VAL:HG21 | 1.60 | 0.84 |
| 1:A:355:HIS:NE2 | 5:A:504:FMT:O1 | 2.11 | 0.84 |
| 1:A:197[A]:MET:HE1 | 1:A:235[A]:VAL:CB | 2.07 | 0.83 |
| 1:C:30[A]:HIS:CE1 | 1:C:33[A]:GLU:OE2 | 2.31 | 0.83 |
| 1:A:335[B]:ASP:OD1 | 7:A:601:HOH:O | 1.96 | 0.83 |
| 1:A:42:ARG:NH2 | 7:A:603:HOH:O | 2.11 | 0.83 |
| 1:A:197[A]:MET:CE | 1:A:235[A]:VAL:HG21 | 2.09 | 0.82 |
| 1:A:39:PRO:CB | 1:A:57[B]:ARG:HD3 | 2.09 | 0.82 |
| 1:F:237[B]:MET:HA | 1:F:237[B]:MET:CE | 2.09 | 0.82 |
| 1:F:120[A]:MET:HE2 | 1:F:361[A]:LEU:CD2 | 2.07 | 0.82 |
| 1:D:178:MET:HE2 | 1:D:193:GLN:HA | 1.62 | 0.82 |
| 1:C:42[B]:ARG:HH11 | 1:C:42[B]:ARG:HG2 | 1.45 | 0.82 |
| 1:F:21:LEU:HD13 | 1:F:23[B]:HIS:HE1 | 1.41 | 0.81 |
| 3:B:502:QR8:O3 | 4:B:513:RAM:O4 | 1.99 | 0.81 |
| 1:F:188[A]:GLU:OE1 | 7:F:601[A]:HOH:O | 1.97 | 0.81 |
| 1:C:106[B]:ARG:NH2 | 7:C:604:HOH:O | 2.12 | 0.81 |
| 1:F:256:LEU:O | 1:F:284:MET:HE1 | 1.80 | 0.81 |
| 1:A:89:GLU:OE2 | 4:A:503[A]:RAM:O3 | 1.98 | 0.80 |
| 1:A:39:PRO:HB2 | 1:A:57[B]:ARG:HD3 | 1.62 | 0.80 |
| 1:C:188:GLU:HG3 | 7:C:749:HOH:O | 1.82 | 0.80 |
| 3:A:502:QR8:O3 | 4:A:503[B]:RAM:H2 | 1.82 | 0.79 |
| 5:A:550:FMT:O2 | 7:A:602:HOH:O | 2.00 | 0.79 |

*Continued on next page...*

CONFIDENTIAL VALIDATION REPORT

Continued from previous page...

| Atom-1 | Atom-2 | Interatomic distance (Å) | Clash overlap (Å) |
|---|---|---|---|
| 1:A:197[A]:MET:HE1 | 1:A:235[A]:VAL:HB | 1.63 | 0.79 |
| 1:C:185[B]:THR:CG2 | 7:C:617:HOH:O | 2.31 | 0.79 |
| 1:A:193:GLN:HE21 | 4:A:503[A]:RAM:C1 | 1.97 | 0.78 |
| 1:E:93[A]:VAL:HG13 | 1:E:237[A]:MET:SD | 2.21 | 0.78 |
| 1:D:45:LEU:HD12 | 1:D:81:PRO:HB2 | 1.66 | 0.78 |
| 1:B:266:ARG:HD3 | 7:B:693:HOH:O | 1.84 | 0.77 |
| 1:C:120[A]:MET:CE | 7:C:773:HOH:O | 2.33 | 0.76 |
| 1:D:208[A]:ARG:CG | 1:D:208[A]:ARG:HH11 | 1.98 | 0.76 |
| 1:B:86[B]:THR:OG1 | 7:B:602:HOH:O | 2.02 | 0.76 |
| 1:F:120[A]:MET:HE1 | 1:F:361[A]:LEU:HD21 | 1.68 | 0.76 |
| 1:A:236:ASN:HD21 | 4:A:503[B]:RAM:H4 | 1.50 | 0.75 |
| 1:C:33[A]:GLU:OE1 | 7:C:601:HOH:O | 2.05 | 0.75 |
| 1:F:277:VAL:HG23 | 1:F:278:PRO:HD3 | 1.69 | 0.75 |
| 1:B:309:VAL:HB | 1:C:122:PRO:HB3 | 1.69 | 0.75 |
| 1:E:125:ARG:HD3 | 1:E:368:GLU:OE2 | 1.86 | 0.75 |
| 1:E:256:LEU:HD22 | 1:E:284[B]:MET:HB3 | 1.70 | 0.74 |
| 4:A:503[A]:RAM:O4 | 5:A:565:FMT:C | 2.35 | 0.74 |
| 1:A:101[B]:HIS:CE1 | 1:A:354[B]:HIS:CD2 | 2.75 | 0.74 |
| 1:F:27:LEU:HD23 | 1:F:326[B]:ARG:NE | 2.03 | 0.74 |
| 1:F:93[B]:VAL:HG13 | 1:F:237[B]:MET:SD | 2.27 | 0.74 |
| 4:B:513:RAM:H5 | 7:B:635:HOH:O | 1.88 | 0.73 |
| 1:D:208[A]:ARG:HH11 | 1:D:208[A]:ARG:HB2 | 1.53 | 0.73 |
| 4:A:503[A]:RAM:O4 | 5:A:565:FMT:H | 1.88 | 0.72 |
| 1:F:188[A]:GLU:HB2 | 7:F:601[A]:HOH:O | 1.88 | 0.72 |
| 2:E:502:HEM:HHC | 2:E:502:HEM:HBB2 | 1.72 | 0.72 |
| 1:F:120[A]:MET:HE1 | 1:F:361[A]:LEU:CD1 | 2.20 | 0.72 |
| 1:D:123[A]:ARG:HG3 | 1:D:123[A]:ARG:NH1 | 2.01 | 0.71 |
| 1:A:197[A]:MET:HE1 | 1:A:235[A]:VAL:HG23 | 1.48 | 0.71 |
| 1:B:42[B]:ARG:HG2 | 1:B:42[B]:ARG:NH2 | 1.90 | 0.71 |
| 1:C:126[B]:SER:OG | 7:C:603:HOH:O | 2.09 | 0.70 |
| 5:C:521:FMT:O1 | 7:C:602:HOH:O | 2.08 | 0.70 |
| 1:D:193:GLN:OE1 | 4:D:503:RAM:H63 | 1.91 | 0.70 |
| 1:C:354[B]:HIS:HE1 | 7:C:636:HOH:O | 1.72 | 0.70 |
| 1:D:183[A]:ARG:HD2 | 1:D:184:LEU:CD1 | 2.22 | 0.70 |
| 1:B:89:GLU:CB | 4:B:513:RAM:C2 | 2.57 | 0.69 |
| 1:B:354[B]:HIS:HD2 | 2:B:501:HEM:O1D | 1.74 | 0.69 |
| 1:F:120[A]:MET:CE | 1:F:361[A]:LEU:CD2 | 2.61 | 0.69 |
| 1:A:178:MET:O | 4:A:503[A]:RAM:O1 | 2.11 | 0.69 |
| 1:B:89:GLU:HB2 | 4:B:513:RAM:O2 | 1.91 | 0.69 |
| 1:A:298:ARG:HE | 5:A:514:FMT:C | 2.06 | 0.69 |
| 1:D:220:ALA:O | 1:D:223:THR:OG1 | 2.09 | 0.69 |

Continued on next page...

CONFIDENTIAL VALIDATION REPORT

*Continued from previous page...*

| Atom-1 | Atom-2 | Interatomic distance (Å) | Clash overlap (Å) |
|---|---|---|---|
| 1:A:123[B]:ARG:HE | 5:A:510:FMT:C | 2.06 | 0.69 |
| 1:C:283:GLU:HG3 | 1:C:337:LEU:HD22 | 1.75 | 0.68 |
| 1:D:256[A]:LEU:HD22 | 1:D:284:MET:HB3 | 1.76 | 0.68 |
| 2:A:501:HEM:HMB2 | 2:A:501:HEM:HBB2 | 1.75 | 0.68 |
| 1:B:170:LEU:HB2 | 5:F:509:FMT:H | 1.76 | 0.67 |
| 1:D:178:MET:CE | 1:D:193:GLN:HA | 2.24 | 0.67 |
| 1:C:35:ARG:HH11 | 5:C:504:FMT:C | 2.08 | 0.67 |
| 1:B:13:ALA:HB1 | 1:C:115:ARG:HG2 | 1.76 | 0.67 |
| 1:A:138[A]:HIS:ND1 | 1:A:139:GLY:O | 2.28 | 0.67 |
| 1:F:188[A]:GLU:CD | 7:F:601[A]:HOH:O | 2.33 | 0.67 |
| 1:D:48:GLY:O | 7:D:601:HOH:O | 2.13 | 0.66 |
| 1:C:30[A]:HIS:NE2 | 7:C:612:HOH:O | 2.29 | 0.66 |
| 1:D:355:HIS:NE2 | 5:D:508:FMT:O1 | 2.26 | 0.65 |
| 1:F:93[B]:VAL:CG1 | 1:F:237[B]:MET:SD | 2.84 | 0.65 |
| 1:B:266:ARG:HD2 | 1:B:337:LEU:HD23 | 1.79 | 0.65 |
| 1:A:39:PRO:HB3 | 1:A:57[B]:ARG:HD3 | 1.79 | 0.65 |
| 2:A:501:HEM:CMB | 2:A:501:HEM:HBB2 | 2.27 | 0.65 |
| 1:D:230:THR:O | 1:D:234:ILE:HD12 | 1.97 | 0.65 |
| 1:F:24:ALA:HB2 | 1:F:391:TRP:NE1 | 2.12 | 0.64 |
| 1:F:133:ASP:O | 1:F:136[B]:VAL:HG12 | 1.96 | 0.64 |
| 5:B:503:FMT:H | 7:B:735:HOH:O | 1.97 | 0.63 |
| 1:F:24:ALA:HB2 | 1:F:391:TRP:CD1 | 2.34 | 0.63 |
| 1:B:392:LYS:HG2 | 1:B:401[A]:GLU:HG3 | 1.80 | 0.63 |
| 1:E:17:TYR:HE1 | 1:E:31:TYR:HH | 1.45 | 0.63 |
| 1:A:256:LEU:HD22 | 1:A:284[A]:MET:HB3 | 1.81 | 0.62 |
| 1:D:89:GLU:HB3 | 4:D:503:RAM:H5 | 1.82 | 0.62 |
| 1:F:390[A]:LYS:NZ | 1:F:402[A]:ARG:HE | 1.97 | 0.62 |
| 1:C:101[B]:HIS:CE1 | 1:C:354[B]:HIS:HD2 | 2.18 | 0.62 |
| 1:B:13:ALA:HB2 | 5:B:507:FMT:O1 | 2.00 | 0.61 |
| 1:A:240:SER:HA | 4:A:503[B]:RAM:C6 | 2.31 | 0.61 |
| 1:A:138[A]:HIS:HE1 | 1:A:141:PRO:O | 1.84 | 0.61 |
| 5:D:528:FMT:C | 7:D:619:HOH:O | 2.47 | 0.61 |
| 1:F:277:VAL:CG2 | 1:F:278:PRO:HD3 | 2.30 | 0.60 |
| 1:F:91:ASP:O | 5:F:514:FMT:O1 | 2.19 | 0.60 |
| 1:E:150:VAL:O | 1:E:154:VAL:HG13 | 2.01 | 0.60 |
| 1:F:201:ASP:OD1 | 7:F:602:HOH:O | 2.17 | 0.60 |
| 1:A:243:ILE:HD12 | 4:A:503[B]:RAM:C6 | 2.32 | 0.60 |
| 1:B:123:ARG:HD3 | 5:B:534:FMT:O1 | 2.01 | 0.60 |
| 3:D:502:QR8:O3 | 4:D:503:RAM:O1 | 2.18 | 0.60 |
| 1:D:101[B]:HIS:NE2 | 1:D:354[B]:HIS:CD2 | 2.70 | 0.60 |
| 1:D:145:VAL:HA | 1:D:149:ALA:HB3 | 1.84 | 0.60 |

*Continued on next page...*

CONFIDENTIAL VALIDATION REPORT

*Continued from previous page...*

| Atom-1 | Atom-2 | Interatomic distance (Å) | Clash overlap (Å) |
|---|---|---|---|
| 1:A:174:PHE:HB3 | 1:A:196:PHE:CD2 | 2.37 | 0.60 |
| 1:F:354[B]:HIS:HE1 | 7:F:626:HOH:O | 1.83 | 0.59 |
| 1:F:133:ASP:OD1 | 1:F:376[A]:ARG:NH1 | 2.34 | 0.59 |
| 1:E:172:ARG:HH11 | 1:E:172:ARG:CG | 2.08 | 0.59 |
| 1:F:103:ARG:HE | 5:F:509:FMT:C | 2.16 | 0.59 |
| 1:B:86[A]:THR:HG23 | 7:B:602:HOH:O | 2.02 | 0.59 |
| 1:D:127:LEU:HD11 | 1:D:155:ALA:HB3 | 1.84 | 0.59 |
| 1:E:125:ARG:CD | 1:E:368:GLU:OE2 | 2.51 | 0.59 |
| 1:F:112:PHE:HB3 | 1:F:357:ILE:O | 2.02 | 0.59 |
| 1:D:193:GLN:NE2 | 4:D:503:RAM:H63 | 2.18 | 0.59 |
| 1:E:290:LEU:O | 1:E:397:ILE:HA | 2.03 | 0.58 |
| 1:A:12:ASP:HB2 | 7:A:722:HOH:O | 2.03 | 0.58 |
| 1:A:240:SER:HA | 4:A:503[B]:RAM:H63 | 1.86 | 0.58 |
| 1:F:21:LEU:HB3 | 1:F:23[B]:HIS:NE2 | 2.19 | 0.58 |
| 1:F:93[A]:VAL:HG23 | 5:F:514:FMT:C | 2.33 | 0.58 |
| 1:A:115[A]:ARG:HD3 | 7:A:770:HOH:O | 2.04 | 0.57 |
| 1:E:322:ALA:O | 1:E:326:ARG:HG2 | 2.04 | 0.57 |
| 1:D:101[B]:HIS:CD2 | 1:D:354[B]:HIS:CD2 | 2.93 | 0.57 |
| 1:C:335:ASP:HB2 | 5:C:552:FMT:C | 2.33 | 0.57 |
| 1:F:125:ARG:O | 1:F:128[B]:VAL:HG12 | 2.03 | 0.57 |
| 1:D:17:TYR:CD1 | 1:D:18:PRO:HA | 2.39 | 0.56 |
| 1:D:208[A]:ARG:HH11 | 1:D:208[A]:ARG:HB3 | 1.67 | 0.56 |
| 1:D:287:TYR:O | 1:D:287:TYR:CD1 | 2.57 | 0.56 |
| 1:C:343[A]:ARG:NH2 | 7:C:619:HOH:O | 2.38 | 0.56 |
| 1:E:312[B]:ARG:HH11 | 1:E:312[B]:ARG:HG2 | 1.70 | 0.56 |
| 1:F:244:ALA:HB1 | 2:F:502:HEM:CHD | 2.35 | 0.56 |
| 1:A:243:ILE:HD12 | 4:A:503[B]:RAM:H61 | 1.86 | 0.56 |
| 1:F:101[A]:HIS:CE1 | 1:F:354[A]:HIS:CE1 | 2.94 | 0.56 |
| 1:C:393[B]:GLN:OE1 | 7:C:605:HOH:O | 2.18 | 0.56 |
| 1:D:208[A]:ARG:CG | 1:D:208[A]:ARG:NH1 | 2.64 | 0.56 |
| 1:F:266:ARG:NH2 | 1:F:337[B]:LEU:HD12 | 2.21 | 0.56 |
| 1:C:172:ARG:HH12 | 5:C:507:FMT:C | 2.19 | 0.55 |
| 1:E:74:ALA:HB3 | 1:E:299:VAL:HB | 1.88 | 0.55 |
| 1:F:349:PHE:CE1 | 1:F:359:ALA:HA | 2.40 | 0.55 |
| 1:A:86:THR:OG1 | 4:A:503[A]:RAM:H61 | 2.05 | 0.55 |
| 1:D:152:PHE:HB3 | 1:D:153:PRO:HD3 | 1.88 | 0.55 |
| 1:D:183[A]:ARG:HD2 | 1:D:184:LEU:HD11 | 1.87 | 0.55 |
| 1:D:160:LEU:HG | 1:D:215[B]:LEU:HD22 | 1.88 | 0.55 |
| 1:F:161:LEU:O | 1:F:216:LEU:HB2 | 2.05 | 0.55 |
| 1:E:286:ARG:HD3 | 1:E:344:ASN:OD1 | 2.06 | 0.55 |
| 1:A:115[B]:ARG:HG3 | 1:A:115[B]:ARG:HH21 | 1.72 | 0.55 |

*Continued on next page...*

CONFIDENTIAL VALIDATION REPORT

Continued from previous page...

| Atom-1 | Atom-2 | Interatomic distance (Å) | Clash overlap (Å) |
|---|---|---|---|
| 2:A:501:HEM:HBC2 | 2:A:501:HEM:HMC2 | 1.89 | 0.55 |
| 2:B:501:HEM:HBB2 | 2:B:501:HEM:HMB2 | 1.87 | 0.55 |
| 1:A:159:GLU:HG2 | 5:A:510:FMT:H | 1.89 | 0.55 |
| 1:C:305[B]:GLU:OE1 | 1:C:310:THR:OG1 | 2.21 | 0.55 |
| 1:E:115:ARG:HB3 | 5:E:501:FMT:C | 2.37 | 0.54 |
| 1:D:178:MET:HE3 | 1:D:196:PHE:HD2 | 1.72 | 0.54 |
| 1:A:283:GLU:HG3 | 1:A:337:LEU:HD22 | 1.89 | 0.54 |
| 1:B:105:ARG:HD3 | 1:B:357:ILE:HD12 | 1.88 | 0.54 |
| 1:B:264:ARG:NH1 | 1:B:380:LEU:O | 2.40 | 0.54 |
| 1:E:77:ASP:HB3 | 1:E:80:THR:CG2 | 2.37 | 0.54 |
| 1:F:286[A]:ARG:HD3 | 1:F:344:ASN:OD1 | 2.08 | 0.54 |
| 1:D:178:MET:HE2 | 1:D:193:GLN:CA | 2.37 | 0.54 |
| 1:A:36[B]:ARG:HG2 | 1:A:37:ASP:OD1 | 2.08 | 0.54 |
| 1:D:158:CYS:HB3 | 1:D:163:VAL:O | 2.08 | 0.54 |
| 1:B:207:ARG:NH2 | 1:B:214:ASP:OD1 | 2.40 | 0.53 |
| 1:A:260:LEU:HG | 1:A:284[A]:MET:HE1 | 1.90 | 0.53 |
| 1:D:326:ARG:HG3 | 5:D:507:FMT:H | 1.89 | 0.53 |
| 1:E:92:GLY:HA2 | 1:E:236:ASN:ND2 | 2.23 | 0.53 |
| 1:F:244:ALA:HB1 | 2:F:502:HEM:C4C | 2.43 | 0.53 |
| 1:E:256:LEU:O | 1:E:284[A]:MET:HE1 | 2.09 | 0.53 |
| 1:F:145[B]:VAL:HA | 1:F:149:ALA:HB3 | 1.90 | 0.53 |
| 1:A:274:PRO:O | 5:A:550:FMT:O1 | 2.26 | 0.53 |
| 4:A:503[A]:RAM:HO4 | 5:A:565:FMT:C | 2.18 | 0.53 |
| 1:B:38:GLU:OE1 | 5:B:510:FMT:O2 | 2.26 | 0.53 |
| 1:E:266:ARG:HD3 | 1:E:337:LEU:HD23 | 1.89 | 0.53 |
| 1:F:145[A]:VAL:HA | 1:F:149:ALA:HB3 | 1.90 | 0.53 |
| 1:F:326[B]:ARG:NH2 | 1:F:335[B]:ASP:OD2 | 2.40 | 0.53 |
| 1:A:38:GLU:HG3 | 5:A:531:FMT:O2 | 2.09 | 0.53 |
| 1:A:38:GLU:HG3 | 5:A:531:FMT:C | 2.38 | 0.53 |
| 1:D:89:GLU:CB | 4:D:503:RAM:C6 | 2.84 | 0.53 |
| 1:A:239:VAL:HG12 | 4:A:503[B]:RAM:H62 | 1.90 | 0.53 |
| 4:A:503[B]:RAM:O2 | 5:A:565:FMT:O1 | 2.25 | 0.53 |
| 1:A:371:SER:HB3 | 5:A:506:FMT:C | 2.39 | 0.52 |
| 1:F:375[B]:ARG:HG2 | 1:F:376[B]:ARG:HG3 | 1.89 | 0.52 |
| 1:D:208[A]:ARG:NH1 | 1:D:208[A]:ARG:HB2 | 2.23 | 0.52 |
| 1:E:256:LEU:HD22 | 1:E:284[B]:MET:CB | 2.39 | 0.52 |
| 1:B:383:ALA:HB3 | 1:B:404:ILE:HG22 | 1.91 | 0.52 |
| 1:C:21:LEU:HD12 | 1:C:22:PRO:HD2 | 1.91 | 0.52 |
| 1:E:101[B]:HIS:CD2 | 1:E:354[B]:HIS:CD2 | 2.97 | 0.52 |
| 1:F:327:ASP:OD1 | 1:F:329:GLU:OE1 | 2.26 | 0.52 |
| 1:F:127[B]:LEU:HD13 | 7:F:629[B]:HOH:O | 2.08 | 0.52 |

Continued on next page...

CONFIDENTIAL VALIDATION REPORT

*Continued from previous page...*

| Atom-1 | Atom-2 | Interatomic distance (Å) | Clash overlap (Å) |
|---|---|---|---|
| 1:B:35:ARG:NH2 | 7:B:611:HOH:O | 2.43 | 0.52 |
| 1:B:17:TYR:HA | 1:B:18:PRO:C | 2.29 | 0.52 |
| 1:F:24:ALA:CB | 1:F:391:TRP:NE1 | 2.73 | 0.52 |
| 1:F:289:PRO:HA | 5:F:505:FMT:H | 1.92 | 0.52 |
| 1:A:127:LEU:HD21 | 1:A:155:ALA:HB3 | 1.92 | 0.51 |
| 1:B:101[B]:HIS:HE1 | 2:B:501:HEM:O2D | 1.92 | 0.51 |
| 1:E:256:LEU:HD22 | 1:E:284[A]:MET:HB3 | 1.91 | 0.51 |
| 1:F:287:TYR:CD1 | 1:F:337[B]:LEU:HG | 2.44 | 0.51 |
| 1:C:28:ASP:OD1 | 1:C:29:PRO:HD2 | 2.11 | 0.51 |
| 1:D:89:GLU:CB | 4:D:503:RAM:H62 | 2.21 | 0.51 |
| 1:E:32:ALA:HA | 1:E:35[B]:ARG:NH1 | 2.26 | 0.51 |
| 1:F:168[B]:ARG:CG | 1:F:168[B]:ARG:HH11 | 2.22 | 0.51 |
| 1:F:21:LEU:HD23 | 1:F:22:PRO:CD | 2.37 | 0.51 |
| 1:C:30[A]:HIS:HD1 | 1:C:33[A]:GLU:CD | 2.08 | 0.51 |
| 1:F:162:GLY:HA3 | 1:F:214:ASP:OD2 | 2.11 | 0.51 |
| 1:C:9:THR:CG2 | 1:C:11:ALA:HB3 | 2.41 | 0.51 |
| 1:E:145:VAL:HA | 1:E:149:ALA:HB3 | 1.92 | 0.51 |
| 3:A:502:QR8:O3 | 4:A:503[A]:RAM:H3 | 2.11 | 0.50 |
| 1:E:101[B]:HIS:CD2 | 1:E:354[B]:HIS:NE2 | 2.79 | 0.50 |
| 1:E:170:LEU:HD22 | 1:E:174:PHE:CZ | 2.46 | 0.50 |
| 1:C:120[A]:MET:HE2 | 7:C:773:HOH:O | 2.02 | 0.50 |
| 1:E:256:LEU:HD22 | 1:E:284[A]:MET:CB | 2.39 | 0.50 |
| 1:A:44[B]:ARG:HD2 | 5:A:511:FMT:C | 2.41 | 0.50 |
| 1:E:35[A]:ARG:HG2 | 1:E:57:ARG:HG2 | 1.92 | 0.50 |
| 1:A:160:LEU:HG | 1:A:215[A]:LEU:HD22 | 1.92 | 0.50 |
| 1:B:172:ARG:HD2 | 7:B:670:HOH:O | 2.10 | 0.50 |
| 2:B:501:HEM:HBC2 | 2:B:501:HEM:CMC | 2.41 | 0.50 |
| 2:D:501:HEM:HMC2 | 2:D:501:HEM:HBC2 | 1.93 | 0.50 |
| 1:D:9:THR:HG22 | 1:D:9:THR:O | 2.12 | 0.50 |
| 1:A:240:SER:CA | 4:A:503[B]:RAM:H63 | 2.42 | 0.50 |
| 2:C:501:HEM:HBB2 | 2:C:501:HEM:HMB2 | 1.94 | 0.50 |
| 1:D:193:GLN:HG2 | 4:D:503:RAM:H4 | 1.93 | 0.50 |
| 1:D:258[B]:HIS:NE2 | 1:D:391:TRP:HZ2 | 2.10 | 0.50 |
| 1:E:251:ASN:HD22 | 1:E:397:ILE:HD12 | 1.76 | 0.50 |
| 1:A:32:ALA:HB2 | 5:A:530:FMT:H | 1.94 | 0.50 |
| 1:A:258:HIS:O | 1:A:262:THR:HG23 | 2.12 | 0.50 |
| 1:F:168[A]:ARG:HG2 | 1:F:172:ARG:HD3 | 1.93 | 0.49 |
| 1:F:46:PRO:HB2 | 1:F:47:TYR:CD1 | 2.47 | 0.49 |
| 1:F:291:VAL:H | 5:F:505:FMT:C | 2.25 | 0.49 |
| 1:F:42:ARG:HD2 | 1:F:51:THR:HG23 | 1.93 | 0.49 |
| 1:D:123[A]:ARG:CG | 1:D:123[A]:ARG:NH1 | 2.54 | 0.49 |

*Continued on next page...*

CONFIDENTIAL VALIDATION REPORT

*Continued from previous page...*

| Atom-1 | Atom-2 | Interatomic distance (Å) | Clash overlap (Å) |
|---|---|---|---|
| 1:C:42[A]:ARG:NH1 | 5:C:523:FMT:O2 | 2.45 | 0.49 |
| 1:B:401[A]:GLU:OE2 | 7:B:603[A]:HOH:O | 2.19 | 0.49 |
| 1:F:21:LEU:HD22 | 1:F:23[B]:HIS:CE1 | 2.47 | 0.49 |
| 1:D:189:ILE:HG21 | 5:D:504:FMT:C | 2.43 | 0.49 |
| 1:E:28:ASP:OD1 | 1:E:30:HIS:HB2 | 2.12 | 0.49 |
| 1:B:256:LEU:HD22 | 1:B:284[B]:MET:HB3 | 1.94 | 0.49 |
| 1:C:14:VAL:HG21 | 1:C:51[B]:THR:HG23 | 1.94 | 0.49 |
| 1:F:237[B]:MET:HA | 5:F:514:FMT:H | 1.95 | 0.49 |
| 1:A:122:PRO:HB3 | 1:D:309:VAL:HB | 1.94 | 0.48 |
| 1:A:240:SER:OG | 4:A:503[B]:RAM:C1 | 2.61 | 0.48 |
| 1:F:252:GLN:OE1 | 1:F:252:GLN:HA | 2.12 | 0.48 |
| 1:D:178:MET:CE | 1:D:196:PHE:HB2 | 2.42 | 0.48 |
| 1:E:251:ASN:HB3 | 1:E:398:ARG:O | 2.12 | 0.48 |
| 1:F:172:ARG:HG2 | 1:F:172:ARG:HH11 | 1.78 | 0.48 |
| 1:C:71:SER:OG | 1:C:97:ASP:OD2 | 2.22 | 0.48 |
| 1:B:280:ALA:O | 1:B:284[A]:MET:HG3 | 2.14 | 0.48 |
| 1:A:328:GLU:HB2 | 5:A:505:FMT:C | 2.44 | 0.48 |
| 1:E:86:THR:HG22 | 1:E:186:ALA:HA | 1.95 | 0.48 |
| 1:E:70:PHE:HB3 | 1:E:298:ARG:HB3 | 1.94 | 0.48 |
| 1:E:35[A]:ARG:HG2 | 1:E:57:ARG:CG | 2.43 | 0.48 |
| 1:F:259:LEU:HB2 | 1:F:284:MET:CE | 2.43 | 0.48 |
| 1:F:24:ALA:CB | 1:F:391:TRP:CE2 | 2.97 | 0.48 |
| 1:A:115[B]:ARG:HH21 | 1:A:115[B]:ARG:CG | 2.26 | 0.47 |
| 1:A:44[B]:ARG:HD2 | 5:A:511:FMT:O2 | 2.14 | 0.47 |
| 1:D:89:GLU:CB | 4:D:503:RAM:H5 | 2.43 | 0.47 |
| 1:D:101[B]:HIS:NE2 | 1:D:354[B]:HIS:HD2 | 2.11 | 0.47 |
| 1:A:89:GLU:HB2 | 4:A:503[B]:RAM:O3 | 2.13 | 0.47 |
| 2:B:501:HEM:HMC2 | 2:B:501:HEM:HBC2 | 1.96 | 0.47 |
| 1:D:208[A]:ARG:NH1 | 1:D:208[A]:ARG:CB | 2.57 | 0.47 |
| 4:A:503[A]:RAM:O4 | 5:A:565:FMT:O2 | 2.27 | 0.47 |
| 1:B:177:ALA:O | 1:B:180:SER:HB3 | 2.14 | 0.47 |
| 5:B:503:FMT:C | 7:B:735:HOH:O | 2.59 | 0.47 |
| 1:E:271:VAL:HA | 1:E:374:VAL:HG13 | 1.95 | 0.47 |
| 1:A:107[A]:LEU:HD13 | 1:A:219:LEU:CD2 | 2.44 | 0.47 |
| 1:D:256[A]:LEU:HD22 | 1:D:284:MET:CB | 2.44 | 0.47 |
| 1:E:380:LEU:HA | 1:E:406:SER:O | 2.15 | 0.47 |
| 1:E:312[B]:ARG:HH11 | 1:E:312[B]:ARG:CG | 2.28 | 0.47 |
| 1:F:117:VAL:O | 1:F:120[A]:MET:HG2 | 2.15 | 0.47 |
| 1:F:206[B]:GLN:HG2 | 7:F:657:HOH:O | 2.15 | 0.47 |
| 1:B:392:LYS:HG3 | 1:B:399:GLY:O | 2.15 | 0.47 |
| 1:C:42[B]:ARG:HH11 | 1:C:42[B]:ARG:CG | 2.23 | 0.47 |

*Continued on next page...*

CONFIDENTIAL VALIDATION REPORT

Continued from previous page...

| Atom-1 | Atom-2 | Interatomic distance (Å) | Clash overlap (Å) |
|---|---|---|---|
| 1:B:193:GLN:CD | 4:B:513:RAM:O2 | 2.53 | 0.47 |
| 1:B:159:GLU:HG2 | 5:B:534:FMT:O1 | 2.15 | 0.47 |
| 1:C:185[B]:THR:HG23 | 1:C:188:GLU:HB3 | 1.97 | 0.47 |
| 1:D:83:MET:O | 7:D:602:HOH:O | 2.21 | 0.47 |
| 1:F:27:LEU:HD23 | 1:F:326[B]:ARG:HE | 1.79 | 0.47 |
| 1:D:101[B]:HIS:CD2 | 1:D:354[B]:HIS:NE2 | 2.83 | 0.46 |
| 1:F:27:LEU:HD23 | 1:F:326[B]:ARG:CZ | 2.44 | 0.46 |
| 1:A:39:PRO:HB3 | 1:A:57[B]:ARG:CD | 2.43 | 0.46 |
| 1:F:168[B]:ARG:NH1 | 1:F:168[B]:ARG:CG | 2.79 | 0.46 |
| 1:A:240:SER:OG | 4:A:503[B]:RAM:H1 | 2.15 | 0.46 |
| 1:B:152:PHE:HB3 | 1:B:153:PRO:HD3 | 1.96 | 0.46 |
| 1:D:178:MET:HE3 | 1:D:196:PHE:HB2 | 1.96 | 0.46 |
| 1:B:101[B]:HIS:CE1 | 1:B:354[B]:HIS:CD2 | 3.03 | 0.46 |
| 1:D:86[A]:THR:HG22 | 1:D:186:ALA:HA | 1.98 | 0.46 |
| 1:C:219:LEU:O | 1:C:223:THR:HG23 | 2.15 | 0.46 |
| 7:A:759:HOH:O | 1:D:38:GLU:HG3 | 2.15 | 0.46 |
| 1:E:115:ARG:HB3 | 5:E:501:FMT:H | 1.97 | 0.46 |
| 1:A:264:ARG:HH11 | 1:A:264:ARG:CG | 2.29 | 0.46 |
| 1:C:206:GLN:HE22 | 5:C:514:FMT:H | 1.81 | 0.46 |
| 1:A:193:GLN:HG2 | 4:A:503[A]:RAM:O2 | 2.16 | 0.46 |
| 1:B:12[B]:ASP:CG | 1:B:44:ARG:HH21 | 2.19 | 0.46 |
| 1:F:144[B]:LEU:HD23 | 1:F:144[B]:LEU:HA | 1.72 | 0.46 |
| 1:F:93[B]:VAL:HG11 | 1:F:237[B]:MET:SD | 2.57 | 0.46 |
| 1:F:287:TYR:CE1 | 1:F:337[B]:LEU:HG | 2.51 | 0.46 |
| 1:D:167:ASP:O | 1:D:168:ARG:C | 2.54 | 0.45 |
| 1:E:30:HIS:O | 1:E:34[A]:LEU:HG | 2.16 | 0.45 |
| 1:D:190[B]:GLN:HA | 1:D:190[B]:GLN:HE21 | 1.80 | 0.45 |
| 1:E:172:ARG:NH1 | 1:E:172:ARG:CG | 2.73 | 0.45 |
| 1:E:295:SER:H | 1:E:318[A]:VAL:CG2 | 2.29 | 0.45 |
| 1:F:260[B]:LEU:HD12 | 1:F:260[B]:LEU:HA | 1.79 | 0.45 |
| 1:F:361[A]:LEU:HD23 | 1:F:361[A]:LEU:HA | 1.83 | 0.45 |
| 1:F:373:LEU:HD22 | 1:F:380[B]:LEU:HD11 | 1.98 | 0.45 |
| 1:F:92:GLY:O | 1:F:96:GLN:HG2 | 2.17 | 0.45 |
| 1:A:152:PHE:HB3 | 1:A:153:PRO:HD3 | 1.99 | 0.45 |
| 1:D:123[B]:ARG:HG3 | 7:D:720:HOH:O | 2.17 | 0.45 |
| 1:E:101[B]:HIS:NE2 | 1:E:354[B]:HIS:CD2 | 2.84 | 0.45 |
| 1:F:237[B]:MET:CA | 1:F:237[B]:MET:CE | 2.84 | 0.45 |
| 1:F:237[A]:MET:HA | 5:F:514:FMT:H | 1.98 | 0.45 |
| 5:B:511:FMT:C | 7:B:618:HOH:O | 2.63 | 0.45 |
| 1:E:106:ARG:CG | 1:E:106:ARG:HH11 | 2.29 | 0.45 |
| 1:E:138[B]:HIS:HD2 | 1:E:139:GLY:O | 2.00 | 0.45 |

Continued on next page...

CONFIDENTIAL VALIDATION REPORT

*Continued from previous page...*

| Atom-1 | Atom-2 | Interatomic distance (Å) | Clash overlap (Å) |
|---|---|---|---|
| 1:B:178:MET:HE3 | 4:B:513:RAM:H4 | 1.97 | 0.45 |
| 1:B:270:LEU:HB2 | 1:B:374:VAL:HG21 | 1.98 | 0.45 |
| 2:C:501:HEM:HMC1 | 2:C:501:HEM:HBC2 | 1.98 | 0.45 |
| 1:D:94:LEU:HD21 | 3:D:502:QR8:O12 | 2.17 | 0.45 |
| 1:A:101[B]:HIS:CE1 | 1:A:354[B]:HIS:HD2 | 2.33 | 0.45 |
| 1:A:264:ARG:HG2 | 1:A:268:GLU:OE2 | 2.16 | 0.45 |
| 1:C:120[A]:MET:HE3 | 7:C:773:HOH:O | 2.09 | 0.45 |
| 1:C:393[B]:GLN:CD | 7:C:605:HOH:O | 2.54 | 0.45 |
| 1:A:12:ASP:N | 7:A:615:HOH:O | 2.49 | 0.45 |
| 1:A:240:SER:HA | 4:A:503[B]:RAM:H61 | 1.98 | 0.45 |
| 1:A:290:LEU:O | 1:A:397:ILE:HA | 2.17 | 0.45 |
| 3:A:502:QR8:C5 | 4:A:503[B]:RAM:O5 | 2.65 | 0.45 |
| 1:C:283:GLU:HG3 | 1:C:337:LEU:CD2 | 2.46 | 0.45 |
| 1:C:290:LEU:O | 1:C:397:ILE:HA | 2.16 | 0.45 |
| 1:D:93:VAL:N | 5:D:528:FMT:O2 | 2.50 | 0.45 |
| 1:A:178:MET:CE | 4:A:503[A]:RAM:O2 | 2.65 | 0.44 |
| 1:D:123[A]:ARG:HD2 | 5:D:512:FMT:O2 | 2.17 | 0.44 |
| 1:F:280:ALA:O | 1:F:284:MET:HG3 | 2.17 | 0.44 |
| 1:A:107[B]:LEU:HD21 | 1:A:222:ALA:CB | 2.47 | 0.44 |
| 1:A:243:ILE:HD12 | 4:A:503[B]:RAM:H62 | 2.00 | 0.44 |
| 1:C:312:ARG:O | 1:C:313:ALA:C | 2.54 | 0.44 |
| 1:C:12[A]:ASP:HB2 | 1:C:51[A]:THR:CG2 | 2.47 | 0.44 |
| 1:E:35[A]:ARG:HG3 | 1:E:56:THR:HB | 2.00 | 0.44 |
| 1:F:120[A]:MET:HE1 | 1:F:361[A]:LEU:CD2 | 2.37 | 0.44 |
| 1:F:46:PRO:HB2 | 1:F:47:TYR:CE1 | 2.53 | 0.44 |
| 1:A:140:SER:OG | 1:A:406:SER:HA | 2.17 | 0.44 |
| 1:D:146:GLU:O | 7:D:603:HOH:O | 2.21 | 0.44 |
| 1:D:178:MET:SD | 4:D:503:RAM:O3 | 2.76 | 0.44 |
| 1:D:62:ARG:HH11 | 1:D:351[A]:HIS:CD2 | 2.35 | 0.44 |
| 1:E:259:LEU:HB2 | 1:E:284[A]:MET:CE | 2.47 | 0.44 |
| 1:E:312[B]:ARG:HG3 | 1:E:313:ALA:N | 2.32 | 0.44 |
| 1:F:184:LEU:HD22 | 1:F:188[A]:GLU:OE1 | 2.18 | 0.44 |
| 1:B:315:GLU:HA | 1:B:316:PRO:HD3 | 1.83 | 0.44 |
| 1:F:131[A]:LEU:HA | 1:F:131[A]:LEU:HD13 | 1.74 | 0.44 |
| 1:F:400:LEU:HD13 | 1:F:403:GLN:CG | 2.48 | 0.44 |
| 1:A:138[A]:HIS:CE1 | 1:A:139:GLY:O | 2.71 | 0.44 |
| 1:D:101[B]:HIS:HE2 | 1:D:354[B]:HIS:HD2 | 1.66 | 0.44 |
| 1:D:246:HIS:O | 1:D:250:VAL:HG23 | 2.18 | 0.44 |
| 5:F:504:FMT:C | 7:F:628:HOH:O | 2.65 | 0.44 |
| 1:B:144:LEU:HA | 1:B:144:LEU:HD12 | 1.89 | 0.44 |
| 1:D:94:LEU:HA | 1:D:354[A]:HIS:CE1 | 2.52 | 0.44 |

*Continued on next page...*

CONFIDENTIAL VALIDATION REPORT

*Continued from previous page...*

| Atom-1 | Atom-2 | Interatomic distance (Å) | Clash overlap (Å) |
|---|---|---|---|
| 4:A:503[B]:RAM:H4 | 4:A:503[B]:RAM:H1 | 1.63 | 0.44 |
| 5:B:511:FMT:H | 7:B:618:HOH:O | 2.18 | 0.44 |
| 1:C:256:LEU:HD22 | 1:C:284:MET:CB | 2.37 | 0.44 |
| 1:D:174:PHE:HB3 | 1:D:196:PHE:CD2 | 2.53 | 0.44 |
| 1:F:376[A]:ARG:NH2 | 1:F:377[A]:PHE:CZ | 2.86 | 0.44 |
| 2:B:501:HEM:HBB2 | 2:B:501:HEM:CMB | 2.48 | 0.44 |
| 1:E:127[B]:LEU:HD21 | 1:E:155:ALA:HB3 | 1.99 | 0.43 |
| 1:B:354[B]:HIS:HE1 | 7:B:713:HOH:O | 2.00 | 0.43 |
| 1:D:178:MET:HE3 | 1:D:196:PHE:CD2 | 2.52 | 0.43 |
| 1:F:129[A]:ASP:OD1 | 1:F:376[A]:ARG:HD2 | 2.18 | 0.43 |
| 1:F:244:ALA:CB | 2:F:502:HEM:CHD | 2.96 | 0.43 |
| 1:F:25:LEU:N | 1:F:25:LEU:HD23 | 2.33 | 0.43 |
| 1:A:246:HIS:O | 1:A:250:VAL:HG23 | 2.18 | 0.43 |
| 1:C:101[B]:HIS:HE1 | 2:C:501:HEM:O2D | 2.01 | 0.43 |
| 1:C:346[A]:HIS:HD2 | 1:C:348:ALA:H | 1.66 | 0.43 |
| 1:A:258:HIS:CE1 | 1:A:262:THR:HG21 | 2.53 | 0.43 |
| 1:D:283:GLU:HA | 1:D:344:ASN:HD21 | 1.82 | 0.43 |
| 1:F:214:ASP:HB3 | 1:F:217:GLY:H | 1.82 | 0.43 |
| 1:F:285:LEU:HA | 1:F:285:LEU:HD23 | 1.90 | 0.43 |
| 1:B:263:GLU:O | 1:B:266:ARG:HG3 | 2.18 | 0.43 |
| 1:D:193:GLN:CG | 4:D:503:RAM:H4 | 2.48 | 0.43 |
| 1:D:256[B]:LEU:HD23 | 1:D:370:LEU:HD11 | 1.99 | 0.43 |
| 1:F:168[B]:ARG:HG2 | 1:F:168[B]:ARG:HH11 | 1.83 | 0.43 |
| 1:A:256:LEU:HD22 | 1:A:284[B]:MET:HB3 | 2.00 | 0.43 |
| 1:B:101[B]:HIS:CE1 | 2:B:501:HEM:O2D | 2.71 | 0.43 |
| 1:B:193:GLN:HG2 | 4:B:513:RAM:H1 | 2.00 | 0.43 |
| 1:D:85:PRO:HD2 | 5:D:504:FMT:O1 | 2.19 | 0.43 |
| 1:E:55:VAL:HG22 | 1:E:319:VAL:HG22 | 2.00 | 0.43 |
| 1:F:283:GLU:HG3 | 1:F:337[A]:LEU:HD12 | 2.01 | 0.43 |
| 2:F:502:HEM:HMB2 | 2:F:502:HEM:HBB2 | 2.01 | 0.43 |
| 1:B:17:TYR:O | 1:B:46:PRO:HD3 | 2.19 | 0.43 |
| 1:C:8:PRO:N | 7:C:639:HOH:O | 2.51 | 0.43 |
| 1:D:264[A]:ARG:NH2 | 1:D:378:PRO:O | 2.51 | 0.43 |
| 1:D:393[B]:GLN:NE2 | 5:D:518:FMT:O2 | 2.46 | 0.43 |
| 1:F:101[A]:HIS:CE1 | 1:F:354[A]:HIS:ND1 | 2.87 | 0.43 |
| 1:F:123:ARG:CZ | 1:F:127[B]:LEU:HD11 | 2.48 | 0.43 |
| 1:D:180:SER:HB2 | 1:D:189:ILE:HD11 | 2.01 | 0.43 |
| 1:D:258[B]:HIS:CD2 | 1:D:391:TRP:CZ2 | 3.07 | 0.43 |
| 1:E:215:LEU:HD23 | 1:E:215:LEU:HA | 1.75 | 0.43 |
| 1:B:121:ARG:HG3 | 1:B:364:LEU:HD11 | 2.01 | 0.42 |
| 1:A:104:LEU:O | 1:A:107[A]:LEU:HB2 | 2.18 | 0.42 |

*Continued on next page...*

CONFIDENTIAL VALIDATION REPORT

*Continued from previous page...*

| Atom-1 | Atom-2 | Interatomic distance (Å) | Clash overlap (Å) |
|---|---|---|---|
| 1:F:117:VAL:HG11 | 1:F:360:GLN:C | 2.40 | 0.42 |
| 1:B:127:LEU:HD21 | 1:B:155:ALA:HB3 | 2.01 | 0.42 |
| 1:B:86[A]:THR:HG22 | 1:B:186:ALA:HA | 2.01 | 0.42 |
| 1:D:44[B]:ARG:HD2 | 1:D:50:GLY:O | 2.19 | 0.42 |
| 1:E:308:THR:O | 1:E:309:VAL:HG23 | 2.19 | 0.42 |
| 1:F:277:VAL:HG23 | 1:F:278:PRO:CD | 2.44 | 0.42 |
| 1:E:157:ILE:HG13 | 1:E:161:LEU:HD22 | 2.00 | 0.42 |
| 1:F:283:GLU:OE1 | 1:F:344:ASN:ND2 | 2.52 | 0.42 |
| 1:B:46:PRO:HB2 | 1:B:47:TYR:CD1 | 2.54 | 0.42 |
| 1:D:380:LEU:HD11 | 1:D:405:VAL:HG21 | 2.02 | 0.42 |
| 2:D:501:HEM:HHC | 2:D:501:HEM:HAB | 1.66 | 0.42 |
| 1:E:226:ASP:HB2 | 1:E:229:LEU:O | 2.19 | 0.42 |
| 1:E:265[A]:LYS:NZ | 1:E:265[A]:LYS:HB2 | 2.35 | 0.42 |
| 1:A:179:LEU:HA | 1:A:179:LEU:HD23 | 1.86 | 0.42 |
| 1:D:89:GLU:HB2 | 4:D:503:RAM:C5 | 2.49 | 0.42 |
| 2:E:502:HEM:HMC2 | 2:E:502:HEM:HBC2 | 2.02 | 0.42 |
| 1:F:105:ARG:NH2 | 1:F:355:HIS:O | 2.38 | 0.42 |
| 1:B:351[A]:HIS:HE1 | 7:B:809:HOH:O | 2.03 | 0.42 |
| 1:C:150:VAL:CG2 | 5:C:507:FMT:H | 2.49 | 0.42 |
| 1:D:14:VAL:O | 1:D:44[A]:ARG:NH1 | 2.44 | 0.42 |
| 1:F:225:ASN:ND2 | 1:F:227:ASP:OD1 | 2.53 | 0.42 |
| 1:A:101[B]:HIS:HE1 | 2:A:501:HEM:O2D | 2.01 | 0.42 |
| 1:A:193:GLN:HE22 | 4:A:503[A]:RAM:H63 | 1.85 | 0.42 |
| 1:B:42[B]:ARG:HD2 | 1:B:53:TRP:CH2 | 2.54 | 0.42 |
| 1:E:174:PHE:HB3 | 1:E:196:PHE:CD2 | 2.54 | 0.42 |
| 1:A:212:THR:O | 5:A:546:FMT:O1 | 2.38 | 0.42 |
| 1:A:354[B]:HIS:HE1 | 7:A:680:HOH:O | 2.03 | 0.42 |
| 1:A:21:LEU:HD12 | 5:A:524:FMT:C | 2.50 | 0.42 |
| 1:D:161:LEU:O | 1:D:216:LEU:HB2 | 2.19 | 0.42 |
| 1:D:99:PRO:HD2 | 7:D:651:HOH:O | 2.20 | 0.42 |
| 1:D:190[B]:GLN:HE21 | 1:D:190[B]:GLN:CA | 2.32 | 0.42 |
| 1:F:94:LEU:HA | 1:F:94:LEU:HD12 | 1.94 | 0.42 |
| 1:A:21:LEU:HA | 1:A:21:LEU:HD12 | 1.92 | 0.41 |
| 1:F:120[A]:MET:HE1 | 1:F:361[A]:LEU:CG | 2.50 | 0.41 |
| 1:A:27:LEU:HD12 | 1:A:27:LEU:HA | 1.86 | 0.41 |
| 1:B:28:ASP:HA | 1:B:29:PRO:HD3 | 1.92 | 0.41 |
| 1:C:194:GLN:O | 5:C:512:FMT:O1 | 2.38 | 0.41 |
| 1:D:123[A]:ARG:HH11 | 1:D:123[A]:ARG:HG2 | 1.79 | 0.41 |
| 1:D:42:ARG:O | 1:D:43:VAL:CG1 | 2.68 | 0.41 |
| 1:F:154:VAL:HG11 | 1:F:168[A]:ARG:HE | 1.84 | 0.41 |
| 1:A:373[B]:LEU:HD12 | 1:A:373[B]:LEU:HA | 1.85 | 0.41 |

*Continued on next page...*

CONFIDENTIAL VALIDATION REPORT

*Continued from previous page...*

| Atom-1 | Atom-2 | Interatomic distance (Å) | Clash overlap (Å) |
|---|---|---|---|
| 1:C:17:TYR:HA | 1:C:18:PRO:C | 2.41 | 0.41 |
| 2:D:501:HEM:HMB2 | 2:D:501:HEM:HBB2 | 2.02 | 0.41 |
| 1:E:219:LEU:O | 1:E:223[B]:THR:HG23 | 2.20 | 0.41 |
| 1:F:75:ALA:HA | 1:F:80:THR:HG21 | 2.01 | 0.41 |
| 1:A:107[B]:LEU:HD21 | 1:A:222:ALA:HB1 | 2.02 | 0.41 |
| 1:B:258:HIS:CE1 | 1:B:262:THR:HG21 | 2.55 | 0.41 |
| 1:F:24:ALA:HB1 | 1:F:391:TRP:CE2 | 2.55 | 0.41 |
| 1:F:140:SER:HB2 | 1:F:406:SER:HA | 2.02 | 0.41 |
| 1:A:283:GLU:HG3 | 1:A:337:LEU:CD2 | 2.49 | 0.41 |
| 1:C:107:LEU:HD12 | 1:C:107:LEU:HA | 1.80 | 0.41 |
| 1:C:252:GLN:HA | 1:C:252:GLN:OE1 | 2.19 | 0.41 |
| 1:E:45:LEU:HD22 | 1:E:81:PRO:CB | 2.51 | 0.41 |
| 1:F:144[A]:LEU:HD12 | 1:F:144[A]:LEU:HA | 1.92 | 0.41 |
| 1:F:246:HIS:O | 1:F:250:VAL:HG12 | 2.21 | 0.41 |
| 1:A:36[B]:ARG:NE | 1:A:37:ASP:OD1 | 2.54 | 0.41 |
| 1:B:76:THR:OG1 | 5:B:538:FMT:O2 | 2.36 | 0.41 |
| 1:D:191[A]:ARG:HA | 1:D:191[A]:ARG:HD3 | 1.58 | 0.41 |
| 1:F:22:PRO:HB3 | 1:F:398:ARG:NH2 | 2.36 | 0.41 |
| 1:F:93[B]:VAL:H | 5:F:514:FMT:C | 2.34 | 0.41 |
| 1:D:101[A]:HIS:HE1 | 2:D:501:HEM:O2D | 2.03 | 0.41 |
| 1:E:260:LEU:HG | 1:E:284[A]:MET:CE | 2.51 | 0.41 |
| 1:A:170:LEU:HD23 | 1:A:170:LEU:C | 2.40 | 0.41 |
| 1:A:286:ARG:HG2 | 1:A:325:ASN:HB3 | 2.03 | 0.41 |
| 1:B:332:ASP:C | 1:B:333[A]:HIS:CG | 2.94 | 0.41 |
| 1:E:71:SER:HB2 | 1:E:97:ASP:OD2 | 2.20 | 0.41 |
| 1:A:17:TYR:HA | 1:A:18:PRO:C | 2.41 | 0.41 |
| 1:C:12[A]:ASP:N | 1:C:12[A]:ASP:OD1 | 2.53 | 0.41 |
| 1:C:185[B]:THR:HG23 | 1:C:188:GLU:CB | 2.51 | 0.41 |
| 1:C:282:GLU:OE1 | 1:C:346[A]:HIS:HE1 | 2.04 | 0.41 |
| 1:B:108:VAL:HG13 | 1:B:112:PHE:CE2 | 2.56 | 0.40 |
| 1:E:244:ALA:CB | 2:E:502:HEM:CHD | 2.98 | 0.40 |
| 1:E:67:ASP:OD1 | 1:E:69:ARG:HG3 | 2.21 | 0.40 |
| 1:F:157:ILE:HD12 | 1:F:157:ILE:HA | 1.90 | 0.40 |
| 1:F:42:ARG:HD2 | 1:F:51:THR:CG2 | 2.51 | 0.40 |
| 1:C:104:LEU:O | 1:C:107:LEU:HB2 | 2.20 | 0.40 |
| 1:A:252:GLN:O | 1:A:256:LEU:HG | 2.21 | 0.40 |
| 1:C:204:VAL:HG22 | 7:C:777:HOH:O | 2.20 | 0.40 |
| 1:F:124:VAL:HG21 | 1:F:364:LEU:HD21 | 2.03 | 0.40 |
| 1:A:256:LEU:HB3 | 1:A:284[A]:MET:SD | 2.62 | 0.40 |
| 1:B:165:LEU:HA | 1:B:165:LEU:HD23 | 1.91 | 0.40 |
| 1:E:77:ASP:HB3 | 1:E:80:THR:HG23 | 2.04 | 0.40 |

*Continued on next page...*

CONFIDENTIAL VALIDATION REPORT

*Continued from previous page...*

| Atom-1 | Atom-2 | Interatomic distance (Å) | Clash overlap (Å) |
|---|---|---|---|
| 1:A:337:LEU:HD23 | 1:A:337:LEU:HA | 1.90 | 0.40 |
| 1:A:178:MET:HE3 | 4:A:503[A]:RAM:O2 | 2.21 | 0.40 |
| 1:C:12[A]:ASP:CB | 1:C:51[A]:THR:HG21 | 2.52 | 0.40 |
| 1:F:237[B]:MET:CA | 1:F:237[B]:MET:HE2 | 2.52 | 0.40 |

All (1) symmetry-related close contacts are listed below. The label for Atom-2 includes the symmetry operator and encoded unit-cell translations to be applied.

| Atom-1 | Atom-2 | Interatomic distance (Å) | Clash overlap (Å) |
|---|---|---|---|
| 1:B:360[B]:GLN:NE2 | 5:A:567:FMT:O2[3_555] | 2.18 | 0.02 |

## 5.3 Torsion angles (i)

### 5.3.1 Protein backbone (i)

In the following table, the Percentiles column shows the percent Ramachandran outliers of the chain as a percentile score with respect to all X-ray entries followed by that with respect to entries of similar resolution.

The Analysed column shows the number of residues for which the backbone conformation was analysed, and the total number of residues.

| Mol | Chain | Analysed | Favoured | Allowed | Outliers | Percentiles | |
|---|---|---|---|---|---|---|---|
| 1 | A | 430/407 (106%) | 418 (97%) | 12 (3%) | 0 | 100 | 100 |
| 1 | B | 421/407 (103%) | 407 (97%) | 14 (3%) | 0 | 100 | 100 |
| 1 | C | 420/407 (103%) | 406 (97%) | 14 (3%) | 0 | 100 | 100 |
| 1 | D | 440/407 (108%) | 409 (93%) | 31 (7%) | 0 | 100 | 100 |
| 1 | E | 431/407 (106%) | 410 (95%) | 21 (5%) | 0 | 100 | 100 |
| 1 | F | 442/407 (109%) | 413 (93%) | 28 (6%) | 1 (0%) | 47 | 57 |
| All | All | 2584/2442 (106%) | 2463 (95%) | 120 (5%) | 1 (0%) | 100 | 100 |

All (1) Ramachandran outliers are listed below:

| Mol | Chain | Res | Type |
|---|---|---|---|
| 1 | F | 273 | ASP |

CONFIDENTIAL VALIDATION REPORT

### 5.3.2 Protein sidechains (i)

In the following table, the Percentiles column shows the percent sidechain outliers of the chain as a percentile score with respect to all X-ray entries followed by that with respect to entries of similar resolution.

The Analysed column shows the number of residues for which the sidechain conformation was analysed, and the total number of residues.

| Mol | Chain | Analysed | Rotameric | Outliers | Percentiles | |
|---|---|---|---|---|---|---|
| 1 | A | 368/341 (108%) | 340 (92%) | 28 (8%) | 13 | 15 |
| 1 | B | 358/341 (105%) | 337 (94%) | 21 (6%) | 19 | 24 |
| 1 | C | 357/341 (105%) | 338 (95%) | 19 (5%) | 22 | 29 |
| 1 | D | 376/341 (110%) | 341 (91%) | 35 (9%) | 9 | 9 |
| 1 | E | 368/341 (108%) | 332 (90%) | 36 (10%) | 8 | 8 |
| 1 | F | 377/341 (111%) | 338 (90%) | 39 (10%) | 7 | 7 |
| All | All | 2204/2046 (108%) | 2026 (92%) | 178 (8%) | 15 | 13 |

All (178) residues with a non-rotameric sidechain are listed below:

| Mol | Chain | Res | Type |
|---|---|---|---|
| 1 | A | 14 | VAL |
| 1 | A | 21 | LEU |
| 1 | A | 27 | LEU |
| 1 | A | 44[A] | ARG |
| 1 | A | 44[B] | ARG |
| 1 | A | 45 | LEU |
| 1 | A | 93 | VAL |
| 1 | A | 102 | THR |
| 1 | A | 107[A] | LEU |
| 1 | A | 107[B] | LEU |
| 1 | A | 108[A] | VAL |
| 1 | A | 108[B] | VAL |
| 1 | A | 126 | SER |
| 1 | A | 183 | ARG |
| 1 | A | 194[A] | GLN |
| 1 | A | 194[B] | GLN |
| 1 | A | 196 | PHE |
| 1 | A | 198 | VAL |
| 1 | A | 201 | ASP |
| 1 | A | 225[A] | ASN |
| 1 | A | 225[B] | ASN |
| 1 | A | 227 | ASP |

*Continued on next page...*

CONFIDENTIAL VALIDATION REPORT

Continued from previous page...

| Mol | Chain | Res | Type |
|---|---|---|---|
| 1 | A | 264 | ARG |
| 1 | A | 269 | SER |
| 1 | A | 276 | LEU |
| 1 | A | 337 | LEU |
| 1 | A | 342[A] | GLU |
| 1 | A | 342[B] | GLU |
| 1 | B | 21 | LEU |
| 1 | B | 42[A] | ARG |
| 1 | B | 42[B] | ARG |
| 1 | B | 86[A] | THR |
| 1 | B | 86[B] | THR |
| 1 | B | 93 | VAL |
| 1 | B | 106 | ARG |
| 1 | B | 116 | ARG |
| 1 | B | 123 | ARG |
| 1 | B | 190[A] | GLN |
| 1 | B | 190[B] | GLN |
| 1 | B | 196 | PHE |
| 1 | B | 209[A] | ASP |
| 1 | B | 209[B] | ASP |
| 1 | B | 209[C] | ASP |
| 1 | B | 225 | ASN |
| 1 | B | 231 | LYS |
| 1 | B | 264 | ARG |
| 1 | B | 342[A] | GLU |
| 1 | B | 342[B] | GLU |
| 1 | B | 374 | VAL |
| 1 | C | 12[A] | ASP |
| 1 | C | 12[B] | ASP |
| 1 | C | 27 | LEU |
| 1 | C | 49[A] | GLU |
| 1 | C | 49[B] | GLU |
| 1 | C | 107 | LEU |
| 1 | C | 115 | ARG |
| 1 | C | 116 | ARG |
| 1 | C | 183 | ARG |
| 1 | C | 191 | ARG |
| 1 | C | 196 | PHE |
| 1 | C | 204 | VAL |
| 1 | C | 206 | GLN |
| 1 | C | 265[A] | LYS |
| 1 | C | 265[B] | LYS |

Continued on next page...

CONFIDENTIAL VALIDATION REPORT

Continued from previous page...

| Mol | Chain | Res | Type |
|---|---|---|---|
| 1 | C | 302 | GLU |
| 1 | C | 329 | GLU |
| 1 | C | 337 | LEU |
| 1 | C | 374 | VAL |
| 1 | D | 9 | THR |
| 1 | D | 14 | VAL |
| 1 | D | 26 | ASP |
| 1 | D | 42 | ARG |
| 1 | D | 59 | SER |
| 1 | D | 86[A] | THR |
| 1 | D | 86[B] | THR |
| 1 | D | 94 | LEU |
| 1 | D | 123[A] | ARG |
| 1 | D | 123[B] | ARG |
| 1 | D | 127 | LEU |
| 1 | D | 132 | LEU |
| 1 | D | 140 | SER |
| 1 | D | 191[A] | ARG |
| 1 | D | 191[B] | ARG |
| 1 | D | 196 | PHE |
| 1 | D | 208[A] | ARG |
| 1 | D | 208[B] | ARG |
| 1 | D | 216 | LEU |
| 1 | D | 225 | ASN |
| 1 | D | 229[A] | LEU |
| 1 | D | 229[B] | LEU |
| 1 | D | 231[A] | LYS |
| 1 | D | 231[B] | LYS |
| 1 | D | 262 | THR |
| 1 | D | 263[A] | GLU |
| 1 | D | 263[B] | GLU |
| 1 | D | 265[A] | LYS |
| 1 | D | 265[B] | LYS |
| 1 | D | 269 | SER |
| 1 | D | 329 | GLU |
| 1 | D | 330 | VAL |
| 1 | D | 343[A] | ARG |
| 1 | D | 343[B] | ARG |
| 1 | D | 374 | VAL |
| 1 | E | 12 | ASP |
| 1 | E | 21 | LEU |
| 1 | E | 55 | VAL |

Continued on next page...

CONFIDENTIAL VALIDATION REPORT

Continued from previous page...

| Mol | Chain | Res | Type |
|---|---|---|---|
| 1 | E | 71 | SER |
| 1 | E | 80 | THR |
| 1 | E | 93[A] | VAL |
| 1 | E | 93[B] | VAL |
| 1 | E | 106 | ARG |
| 1 | E | 107 | LEU |
| 1 | E | 110 | LYS |
| 1 | E | 125 | ARG |
| 1 | E | 126 | SER |
| 1 | E | 130 | SER |
| 1 | E | 154 | VAL |
| 1 | E | 161 | LEU |
| 1 | E | 172 | ARG |
| 1 | E | 196 | PHE |
| 1 | E | 206 | GLN |
| 1 | E | 225 | ASN |
| 1 | E | 227[A] | ASP |
| 1 | E | 227[B] | ASP |
| 1 | E | 265[A] | LYS |
| 1 | E | 265[B] | LYS |
| 1 | E | 269 | SER |
| 1 | E | 278 | PRO |
| 1 | E | 309 | VAL |
| 1 | E | 312[A] | ARG |
| 1 | E | 312[B] | ARG |
| 1 | E | 329[A] | GLU |
| 1 | E | 329[B] | GLU |
| 1 | E | 343[A] | ARG |
| 1 | E | 343[B] | ARG |
| 1 | E | 355[A] | HIS |
| 1 | E | 355[B] | HIS |
| 1 | E | 393[A] | GLN |
| 1 | E | 393[B] | GLN |
| 1 | F | 21 | LEU |
| 1 | F | 27 | LEU |
| 1 | F | 44 | ARG |
| 1 | F | 115 | ARG |
| 1 | F | 116 | ARG |
| 1 | F | 120[A] | MET |
| 1 | F | 120[B] | MET |
| 1 | F | 125 | ARG |
| 1 | F | 127[A] | LEU |

Continued on next page...

CONFIDENTIAL VALIDATION REPORT

*Continued from previous page...*

| Mol | Chain | Res | Type |
|---|---|---|---|
| 1 | F | 127[B] | LEU |
| 1 | F | 131[A] | LEU |
| 1 | F | 131[B] | LEU |
| 1 | F | 135 | MET |
| 1 | F | 165 | LEU |
| 1 | F | 190[A] | GLN |
| 1 | F | 190[B] | GLN |
| 1 | F | 196 | PHE |
| 1 | F | 198 | VAL |
| 1 | F | 201 | ASP |
| 1 | F | 207 | ARG |
| 1 | F | 213 | GLU |
| 1 | F | 214 | ASP |
| 1 | F | 216 | LEU |
| 1 | F | 219 | LEU |
| 1 | F | 225 | ASN |
| 1 | F | 227 | ASP |
| 1 | F | 231 | LYS |
| 1 | F | 250 | VAL |
| 1 | F | 262 | THR |
| 1 | F | 264 | ARG |
| 1 | F | 302 | GLU |
| 1 | F | 309 | VAL |
| 1 | F | 312 | ARG |
| 1 | F | 330 | VAL |
| 1 | F | 374 | VAL |
| 1 | F | 375[A] | ARG |
| 1 | F | 375[B] | ARG |
| 1 | F | 376[A] | ARG |
| 1 | F | 376[B] | ARG |

Some sidechains can be flipped to improve hydrogen bonding and reduce clashes. All (19) such sidechains are listed below:

| Mol | Chain | Res | Type |
|---|---|---|---|
| 1 | A | 193 | GLN |
| 1 | A | 236 | ASN |
| 1 | A | 258 | HIS |
| 1 | A | 393 | GLN |
| 1 | B | 193 | GLN |
| 1 | B | 258 | HIS |
| 1 | C | 206 | GLN |
| 1 | C | 225 | ASN |

*Continued on next page...*

CONFIDENTIAL VALIDATION REPORT

*Continued from previous page...*

| Mol | Chain | Res | Type |
|---|---|---|---|
| 1 | C | 360 | GLN |
| 1 | D | 96 | GLN |
| 1 | D | 193 | GLN |
| 1 | D | 225 | ASN |
| 1 | D | 320 | HIS |
| 1 | E | 193 | GLN |
| 1 | E | 206 | GLN |
| 1 | E | 236 | ASN |
| 1 | F | 193 | GLN |
| 1 | F | 225 | ASN |
| 1 | F | 236 | ASN |

### 5.3.3 RNA (i)

There are no RNA molecules in this entry.

## 5.4 Non-standard residues in protein, DNA, RNA chains (i)

There are no non-standard protein/DNA/RNA residues in this entry.

## 5.5 Carbohydrates (i)

There are no monosaccharides in this entry.

## 5.6 Ligand geometry (i)

Of 236 ligands modelled in this entry, 6 are monoatomic - leaving 230 for Mogul analysis.

In the following table, the Counts columns list the number of bonds (or angles) for which Mogul statistics could be retrieved, the number of bonds (or angles) that are observed in the model and the number of bonds (or angles) that are defined in the Chemical Component Dictionary. The Link column lists molecule types, if any, to which the group is linked. The Z score for a bond length (or angle) is the number of standard deviations the observed value is removed from the expected value. A bond length (or angle) with $|Z| > 2$ is considered an outlier worth inspection. RMSZ is the root-mean-square of all Z scores of the bond lengths (or angles).

| Mol | Type | Chain | Res | Link | Bond lengths | | | Bond angles | | |
|---|---|---|---|---|---|---|---|---|---|---|
| | | | | | Counts | RMSZ | $\#\|Z\| > 2$ | Counts | RMSZ | $\#\|Z\| > 2$ |
| 5 | FMT | F | 501 | - | 0,2,2 | 0.00 | - | 0,1,1 | 0.00 | - |
| 5 | FMT | C | 525 | - | 0,2,2 | 0.00 | - | 0,1,1 | 0.00 | - |
| 5 | FMT | A | 511 | - | 0,2,2 | 0.00 | - | 0,1,1 | 0.00 | - |

CONFIDENTIAL VALIDATION REPORT

| Mol | Type | Chain | Res | Link | Bond lengths | | | Bond angles | | |
|---|---|---|---|---|---|---|---|---|---|---|
| | | | | | Counts | RMSZ | #\|Z\| > 2 | Counts | RMSZ | #\|Z\| > 2 |
| 5 | FMT | D | 536 | - | 0,2,2 | 0.00 | - | 0,1,1 | 0.00 | - |
| 5 | FMT | D | 522 | - | 0,2,2 | 0.00 | - | 0,1,1 | 0.00 | - |
| 5 | FMT | C | 526 | - | 0,2,2 | 0.00 | - | 0,1,1 | 0.00 | - |
| 5 | FMT | F | 505 | - | 0,2,2 | 0.00 | - | 0,1,1 | 0.00 | - |
| 5 | FMT | D | 511 | - | 0,2,2 | 0.00 | - | 0,1,1 | 0.00 | - |
| 5 | FMT | A | 515 | - | 0,2,2 | 0.00 | - | 0,1,1 | 0.00 | - |
| 5 | FMT | A | 512 | - | 0,2,2 | 0.00 | - | 0,1,1 | 0.00 | - |
| 5 | FMT | D | 512 | - | 0,2,2 | 0.00 | - | 0,1,1 | 0.00 | - |
| 5 | FMT | A | 550 | - | 0,2,2 | 0.00 | - | 0,1,1 | 0.00 | - |
| 5 | FMT | A | 562 | - | 0,2,2 | 0.00 | - | 0,1,1 | 0.00 | - |
| 5 | FMT | A | 559 | - | 0,2,2 | 0.00 | - | 0,1,1 | 0.00 | - |
| 5 | FMT | A | 547 | - | 0,2,2 | 0.00 | - | 0,1,1 | 0.00 | - |
| 5 | FMT | F | 509 | - | 0,2,2 | 0.00 | - | 0,1,1 | 0.00 | - |
| 5 | FMT | B | 540 | - | 0,2,2 | 0.00 | - | 0,1,1 | 0.00 | - |
| 3 | QR8 | F | 503 | - | 26,26,26 | 1.68 | 3 (11%) | 35,38,38 | 1.59 | 9 (25%) |
| 5 | FMT | C | 551 | - | 0,2,2 | 0.00 | - | 0,1,1 | 0.00 | - |
| 5 | FMT | B | 514 | - | 0,2,2 | 0.00 | - | 0,1,1 | 0.00 | - |
| 5 | FMT | C | 552 | - | 0,2,2 | 0.00 | - | 0,1,1 | 0.00 | - |
| 5 | FMT | A | 552 | - | 0,2,2 | 0.00 | - | 0,1,1 | 0.00 | - |
| 5 | FMT | A | 546 | - | 0,2,2 | 0.00 | - | 0,1,1 | 0.00 | - |
| 3 | QR8 | D | 502 | - | 26,26,26 | 1.64 | 4 (15%) | 35,38,38 | 1.90 | 9 (25%) |
| 5 | FMT | B | 521 | - | 0,2,2 | 0.00 | - | 0,1,1 | 0.00 | - |
| 3 | QR8 | B | 502 | - | 26,26,26 | 1.79 | 4 (15%) | 35,38,38 | 1.73 | 10 (28%) |
| 5 | FMT | A | 529 | - | 0,2,2 | 0.00 | - | 0,1,1 | 0.00 | - |
| 5 | FMT | B | 533 | - | 0,2,2 | 0.00 | - | 0,1,1 | 0.00 | - |
| 5 | FMT | A | 518 | - | 0,2,2 | 0.00 | - | 0,1,1 | 0.00 | - |
| 2 | HEM | D | 501 | 1 | 27,50,50 | 1.64 | 4 (14%) | 17,82,82 | 1.87 | 5 (29%) |
| 5 | FMT | A | 506 | - | 0,2,2 | 0.00 | - | 0,1,1 | 0.00 | - |
| 4 | RAM | D | 503 | - | 11,11,11 | 1.14 | 1 (9%) | 15,16,16 | 2.77 | 10 (66%) |
| 5 | FMT | E | 514 | - | 0,2,2 | 0.00 | - | 0,1,1 | 0.00 | - |
| 5 | FMT | C | 539 | - | 0,2,2 | 0.00 | - | 0,1,1 | 0.00 | - |
| 3 | QR8 | E | 503 | - | 26,26,26 | 1.55 | 4 (15%) | 35,38,38 | 1.77 | 10 (28%) |
| 5 | FMT | F | 507 | - | 0,2,2 | 0.00 | - | 0,1,1 | 0.00 | - |
| 5 | FMT | C | 535 | - | 0,2,2 | 0.00 | - | 0,1,1 | 0.00 | - |
| 5 | FMT | A | 558 | - | 0,2,2 | 0.00 | - | 0,1,1 | 0.00 | - |
| 5 | FMT | B | 526 | - | 0,2,2 | 0.00 | - | 0,1,1 | 0.00 | - |
| 5 | FMT | D | 533 | - | 0,2,2 | 0.00 | - | 0,1,1 | 0.00 | - |
| 5 | FMT | B | 523 | - | 0,2,2 | 0.00 | - | 0,1,1 | 0.00 | - |
| 5 | FMT | A | 549 | - | 0,2,2 | 0.00 | - | 0,1,1 | 0.00 | - |
| 5 | FMT | D | 505 | - | 0,2,2 | 0.00 | - | 0,1,1 | 0.00 | - |
| 5 | FMT | D | 537 | - | 0,2,2 | 0.00 | - | 0,1,1 | 0.00 | - |
| 5 | FMT | C | 511 | - | 0,2,2 | 0.00 | - | 0,1,1 | 0.00 | - |

CONFIDENTIAL VALIDATION REPORT

| Mol | Type | Chain | Res | Link | Bond lengths | | | Bond angles | | |
|---|---|---|---|---|---|---|---|---|---|---|
| | | | | | Counts | RMSZ | #\|Z\| > 2 | Counts | RMSZ | #\|Z\| > 2 |
| 5 | FMT | A | 557 | - | 0,2,2 | 0.00 | - | 0,1,1 | 0.00 | - |
| 5 | FMT | C | 528 | - | 0,2,2 | 0.00 | - | 0,1,1 | 0.00 | - |
| 5 | FMT | A | 532 | - | 0,2,2 | 0.00 | - | 0,1,1 | 0.00 | - |
| 5 | FMT | E | 515 | - | 0,2,2 | 0.00 | - | 0,1,1 | 0.00 | - |
| 5 | FMT | D | 509 | - | 0,2,2 | 0.00 | - | 0,1,1 | 0.00 | - |
| 5 | FMT | B | 535 | - | 0,2,2 | 0.00 | - | 0,1,1 | 0.00 | - |
| 5 | FMT | C | 522 | - | 0,2,2 | 0.00 | - | 0,1,1 | 0.00 | - |
| 5 | FMT | A | 522 | - | 0,2,2 | 0.00 | - | 0,1,1 | 0.00 | - |
| 5 | FMT | B | 508 | - | 0,2,2 | 0.00 | - | 0,1,1 | 0.00 | - |
| 5 | FMT | D | 535 | - | 0,2,2 | 0.00 | - | 0,1,1 | 0.00 | - |
| 5 | FMT | B | 536 | - | 0,2,2 | 0.00 | - | 0,1,1 | 0.00 | - |
| 5 | FMT | A | 533 | - | 0,2,2 | 0.00 | - | 0,1,1 | 0.00 | - |
| 5 | FMT | A | 539 | - | 0,2,2 | 0.00 | - | 0,1,1 | 0.00 | - |
| 2 | HEM | C | 501 | 1 | 27,50,50 | 1.73 | 3 (11%) | 17,82,82 | 2.36 | 7 (41%) |
| 5 | FMT | E | 511 | - | 0,2,2 | 0.00 | - | 0,1,1 | 0.00 | - |
| 5 | FMT | E | 516 | - | 0,2,2 | 0.00 | - | 0,1,1 | 0.00 | - |
| 5 | FMT | B | 520 | - | 0,2,2 | 0.00 | - | 0,1,1 | 0.00 | - |
| 5 | FMT | C | 543 | - | 0,2,2 | 0.00 | - | 0,1,1 | 0.00 | - |
| 5 | FMT | A | 535 | - | 0,2,2 | 0.00 | - | 0,1,1 | 0.00 | - |
| 5 | FMT | D | 518 | - | 0,2,2 | 0.00 | - | 0,1,1 | 0.00 | - |
| 5 | FMT | C | 510 | - | 0,2,2 | 0.00 | - | 0,1,1 | 0.00 | - |
| 5 | FMT | A | 568 | - | 0,2,2 | 0.00 | - | 0,1,1 | 0.00 | - |
| 5 | FMT | B | 506 | - | 0,2,2 | 0.00 | - | 0,1,1 | 0.00 | - |
| 5 | FMT | E | 504 | - | 0,2,2 | 0.00 | - | 0,1,1 | 0.00 | - |
| 5 | FMT | C | 523 | - | 0,2,2 | 0.00 | - | 0,1,1 | 0.00 | - |
| 5 | FMT | A | 509 | - | 0,2,2 | 0.00 | - | 0,1,1 | 0.00 | - |
| 5 | FMT | D | 520 | - | 0,2,2 | 0.00 | - | 0,1,1 | 0.00 | - |
| 5 | FMT | C | 520 | - | 0,2,2 | 0.00 | - | 0,1,1 | 0.00 | - |
| 5 | FMT | C | 509 | - | 0,2,2 | 0.00 | - | 0,1,1 | 0.00 | - |
| 5 | FMT | D | 530 | - | 0,2,2 | 0.00 | - | 0,1,1 | 0.00 | - |
| 5 | FMT | C | 531 | - | 0,2,2 | 0.00 | - | 0,1,1 | 0.00 | - |
| 5 | FMT | D | 527 | - | 0,2,2 | 0.00 | - | 0,1,1 | 0.00 | - |
| 5 | FMT | B | 503 | - | 0,2,2 | 0.00 | - | 0,1,1 | 0.00 | - |
| 5 | FMT | E | 508 | - | 0,2,2 | 0.00 | - | 0,1,1 | 0.00 | - |
| 5 | FMT | A | 507 | - | 0,2,2 | 0.00 | - | 0,1,1 | 0.00 | - |
| 5 | FMT | B | 531 | - | 0,2,2 | 0.00 | - | 0,1,1 | 0.00 | - |
| 5 | FMT | A | 574 | - | 0,2,2 | 0.00 | - | 0,1,1 | 0.00 | - |
| 5 | FMT | B | 525 | - | 0,2,2 | 0.00 | - | 0,1,1 | 0.00 | - |
| 5 | FMT | D | 528 | - | 0,2,2 | 0.00 | - | 0,1,1 | 0.00 | - |
| 5 | FMT | C | 512 | - | 0,2,2 | 0.00 | - | 0,1,1 | 0.00 | - |
| 5 | FMT | D | 521 | - | 0,2,2 | 0.00 | - | 0,1,1 | 0.00 | - |
| 5 | FMT | C | 529 | - | 0,2,2 | 0.00 | - | 0,1,1 | 0.00 | - |
| 5 | FMT | E | 506 | - | 0,2,2 | 0.00 | - | 0,1,1 | 0.00 | - |

CONFIDENTIAL VALIDATION REPORT

| Mol | Type | Chain | Res | Link | Bond lengths | | | Bond angles | | |
|---|---|---|---|---|---|---|---|---|---|---|
| | | | | | Counts | RMSZ | #\|Z\| > 2 | Counts | RMSZ | #\|Z\| > 2 |
| 5 | FMT | A | 536 | - | 0,2,2 | 0.00 | - | 0,1,1 | 0.00 | - |
| 5 | FMT | C | 518 | - | 0,2,2 | 0.00 | - | 0,1,1 | 0.00 | - |
| 5 | FMT | B | 507 | - | 0,2,2 | 0.00 | - | 0,1,1 | 0.00 | - |
| 5 | FMT | C | 516 | - | 0,2,2 | 0.00 | - | 0,1,1 | 0.00 | - |
| 5 | FMT | A | 534 | - | 0,2,2 | 0.00 | - | 0,1,1 | 0.00 | - |
| 5 | FMT | C | 530 | - | 0,2,2 | 0.00 | - | 0,1,1 | 0.00 | - |
| 4 | RAM | A | 503[A] | - | 11,11,11 | 1.32 | 3 (27%) | 15,16,16 | 3.59 | 9 (60%) |
| 5 | FMT | A | 528 | - | 0,2,2 | 0.00 | - | 0,1,1 | 0.00 | - |
| 5 | FMT | C | 517 | - | 0,2,2 | 0.00 | - | 0,1,1 | 0.00 | - |
| 5 | FMT | C | 533 | - | 0,2,2 | 0.00 | - | 0,1,1 | 0.00 | - |
| 5 | FMT | D | 519 | - | 0,2,2 | 0.00 | - | 0,1,1 | 0.00 | - |
| 5 | FMT | B | 512 | - | 0,2,2 | 0.00 | - | 0,1,1 | 0.00 | - |
| 5 | FMT | A | 571 | - | 0,2,2 | 0.00 | - | 0,1,1 | 0.00 | - |
| 5 | FMT | C | 508 | - | 0,2,2 | 0.00 | - | 0,1,1 | 0.00 | - |
| 5 | FMT | A | 566 | - | 0,2,2 | 0.00 | - | 0,1,1 | 0.00 | - |
| 5 | FMT | B | 539 | - | 0,2,2 | 0.00 | - | 0,1,1 | 0.00 | - |
| 5 | FMT | D | 507 | - | 0,2,2 | 0.00 | - | 0,1,1 | 0.00 | - |
| 5 | FMT | A | 544 | - | 0,2,2 | 0.00 | - | 0,1,1 | 0.00 | - |
| 2 | HEM | E | 502 | 1 | 27,50,50 | 1.12 | 4 (14%) | 17,82,82 | 2.15 | 5 (29%) |
| 5 | FMT | C | 503 | - | 0,2,2 | 0.00 | - | 0,1,1 | 0.00 | - |
| 5 | FMT | B | 518 | - | 0,2,2 | 0.00 | - | 0,1,1 | 0.00 | - |
| 5 | FMT | D | 515 | - | 0,2,2 | 0.00 | - | 0,1,1 | 0.00 | - |
| 5 | FMT | B | 538 | - | 0,2,2 | 0.00 | - | 0,1,1 | 0.00 | - |
| 5 | FMT | E | 509 | - | 0,2,2 | 0.00 | - | 0,1,1 | 0.00 | - |
| 5 | FMT | B | 532 | - | 0,2,2 | 0.00 | - | 0,1,1 | 0.00 | - |
| 5 | FMT | A | 560 | - | 0,2,2 | 0.00 | - | 0,1,1 | 0.00 | - |
| 5 | FMT | C | 515 | - | 0,2,2 | 0.00 | - | 0,1,1 | 0.00 | - |
| 5 | FMT | D | 516 | - | 0,2,2 | 0.00 | - | 0,1,1 | 0.00 | - |
| 5 | FMT | E | 517 | - | 0,2,2 | 0.00 | - | 0,1,1 | 0.00 | - |
| 5 | FMT | A | 525 | - | 0,2,2 | 0.00 | - | 0,1,1 | 0.00 | - |
| 5 | FMT | B | 534 | - | 0,2,2 | 0.00 | - | 0,1,1 | 0.00 | - |
| 5 | FMT | D | 504 | - | 0,2,2 | 0.00 | - | 0,1,1 | 0.00 | - |
| 5 | FMT | A | 570 | - | 0,2,2 | 0.00 | - | 0,1,1 | 0.00 | - |
| 5 | FMT | F | 510 | - | 0,2,2 | 0.00 | - | 0,1,1 | 0.00 | - |
| 5 | FMT | E | 510 | - | 0,2,2 | 0.00 | - | 0,1,1 | 0.00 | - |
| 5 | FMT | A | 527 | - | 0,2,2 | 0.00 | - | 0,1,1 | 0.00 | - |
| 5 | FMT | C | 545 | - | 0,2,2 | 0.00 | - | 0,1,1 | 0.00 | - |
| 5 | FMT | A | 523 | - | 0,2,2 | 0.00 | - | 0,1,1 | 0.00 | - |
| 5 | FMT | A | 554 | - | 0,2,2 | 0.00 | - | 0,1,1 | 0.00 | - |
| 5 | FMT | B | 530 | - | 0,2,2 | 0.00 | - | 0,1,1 | 0.00 | - |
| 5 | FMT | B | 537 | - | 0,2,2 | 0.00 | - | 0,1,1 | 0.00 | - |
| 5 | FMT | C | 546 | - | 0,2,2 | 0.00 | - | 0,1,1 | 0.00 | - |

CONFIDENTIAL VALIDATION REPORT

| Mol | Type | Chain | Res | Link | Bond lengths | | | Bond angles | | |
|---|---|---|---|---|---|---|---|---|---|---|
| | | | | | Counts | RMSZ | #\|Z\| > 2 | Counts | RMSZ | #\|Z\| > 2 |
| 5 | FMT | B | 505 | - | 0,2,2 | 0.00 | - | 0,1,1 | 0.00 | - |
| 5 | FMT | E | 512 | - | 0,2,2 | 0.00 | - | 0,1,1 | 0.00 | - |
| 5 | FMT | C | 506 | - | 0,2,2 | 0.00 | - | 0,1,1 | 0.00 | - |
| 5 | FMT | A | 553 | - | 0,2,2 | 0.00 | - | 0,1,1 | 0.00 | - |
| 5 | FMT | A | 531 | - | 0,2,2 | 0.00 | - | 0,1,1 | 0.00 | - |
| 5 | FMT | F | 515 | - | 0,2,2 | 0.00 | - | 0,1,1 | 0.00 | - |
| 5 | FMT | C | 505 | - | 0,2,2 | 0.00 | - | 0,1,1 | 0.00 | - |
| 5 | FMT | A | 555 | - | 0,2,2 | 0.00 | - | 0,1,1 | 0.00 | - |
| 5 | FMT | F | 511 | - | 0,2,2 | 0.00 | - | 0,1,1 | 0.00 | - |
| 3 | QR8 | A | 502 | - | 26,26,26 | 1.69 | 4 (15%) | 35,38,38 | 1.83 | 9 (25%) |
| 5 | FMT | C | 514 | - | 0,2,2 | 0.00 | - | 0,1,1 | 0.00 | - |
| 5 | FMT | B | 509 | - | 0,2,2 | 0.00 | - | 0,1,1 | 0.00 | - |
| 5 | FMT | A | 516 | - | 0,2,2 | 0.00 | - | 0,1,1 | 0.00 | - |
| 5 | FMT | C | 536 | - | 0,2,2 | 0.00 | - | 0,1,1 | 0.00 | - |
| 5 | FMT | F | 513 | - | 0,2,2 | 0.00 | - | 0,1,1 | 0.00 | - |
| 5 | FMT | C | 521 | - | 0,2,2 | 0.00 | - | 0,1,1 | 0.00 | - |
| 5 | FMT | A | 540 | - | 0,2,2 | 0.00 | - | 0,1,1 | 0.00 | - |
| 5 | FMT | D | 525 | - | 0,2,2 | 0.00 | - | 0,1,1 | 0.00 | - |
| 5 | FMT | A | 563 | - | 0,2,2 | 0.00 | - | 0,1,1 | 0.00 | - |
| 5 | FMT | D | 529 | - | 0,2,2 | 0.00 | - | 0,1,1 | 0.00 | - |
| 5 | FMT | B | 510 | - | 0,2,2 | 0.00 | - | 0,1,1 | 0.00 | - |
| 5 | FMT | C | 549 | - | 0,2,2 | 0.00 | - | 0,1,1 | 0.00 | - |
| 5 | FMT | E | 513 | - | 0,2,2 | 0.00 | - | 0,1,1 | 0.00 | - |
| 2 | HEM | B | 501 | 1 | 27,50,50 | 1.52 | 6 (22%) | 17,82,82 | 2.30 | 6 (35%) |
| 5 | FMT | B | 524 | - | 0,2,2 | 0.00 | - | 0,1,1 | 0.00 | - |
| 5 | FMT | A | 519 | - | 0,2,2 | 0.00 | - | 0,1,1 | 0.00 | - |
| 5 | FMT | E | 505 | - | 0,2,2 | 0.00 | - | 0,1,1 | 0.00 | - |
| 5 | FMT | A | 556 | - | 0,2,2 | 0.00 | - | 0,1,1 | 0.00 | - |
| 5 | FMT | D | 508 | - | 0,2,2 | 0.00 | - | 0,1,1 | 0.00 | - |
| 5 | FMT | C | 513 | - | 0,2,2 | 0.00 | - | 0,1,1 | 0.00 | - |
| 5 | FMT | D | 534 | - | 0,2,2 | 0.00 | - | 0,1,1 | 0.00 | - |
| 5 | FMT | B | 517 | - | 0,2,2 | 0.00 | - | 0,1,1 | 0.00 | - |
| 5 | FMT | A | 510 | - | 0,2,2 | 0.00 | - | 0,1,1 | 0.00 | - |
| 5 | FMT | D | 510 | - | 0,2,2 | 0.00 | - | 0,1,1 | 0.00 | - |
| 5 | FMT | C | 519 | - | 0,2,2 | 0.00 | - | 0,1,1 | 0.00 | - |
| 5 | FMT | A | 513 | - | 0,2,2 | 0.00 | - | 0,1,1 | 0.00 | - |
| 5 | FMT | B | 516 | - | 0,2,2 | 0.00 | - | 0,1,1 | 0.00 | - |
| 5 | FMT | C | 540 | - | 0,2,2 | 0.00 | - | 0,1,1 | 0.00 | - |
| 5 | FMT | D | 532 | - | 0,2,2 | 0.00 | - | 0,1,1 | 0.00 | - |
| 5 | FMT | F | 506 | - | 0,2,2 | 0.00 | - | 0,1,1 | 0.00 | - |
| 5 | FMT | C | 524 | - | 0,2,2 | 0.00 | - | 0,1,1 | 0.00 | - |
| 5 | FMT | A | 543 | - | 0,2,2 | 0.00 | - | 0,1,1 | 0.00 | - |

CONFIDENTIAL VALIDATION REPORT

| Mol | Type | Chain | Res | Link | Bond lengths | | | Bond angles | | |
|---|---|---|---|---|---|---|---|---|---|---|
| | | | | | Counts | RMSZ | #\|Z\| > 2 | Counts | RMSZ | #\|Z\| > 2 |
| 5 | FMT | D | 517 | - | 0,2,2 | 0.00 | - | 0,1,1 | 0.00 | - |
| 5 | FMT | B | 504 | - | 0,2,2 | 0.00 | - | 0,1,1 | 0.00 | - |
| 5 | FMT | F | 504 | - | 0,2,2 | 0.00 | - | 0,1,1 | 0.00 | - |
| 3 | QR8 | C | 502 | - | 26,26,26 | 1.53 | 2 (7%) | 35,38,38 | 1.70 | 7 (20%) |
| 5 | FMT | C | 532 | - | 0,2,2 | 0.00 | - | 0,1,1 | 0.00 | - |
| 2 | HEM | F | 502 | 1 | 27,50,50 | 0.85 | 1 (3%) | 17,82,82 | 1.59 | 6 (35%) |
| 5 | FMT | A | 564 | - | 0,2,2 | 0.00 | - | 0,1,1 | 0.00 | - |
| 5 | FMT | D | 514 | - | 0,2,2 | 0.00 | - | 0,1,1 | 0.00 | - |
| 5 | FMT | C | 537 | - | 0,2,2 | 0.00 | - | 0,1,1 | 0.00 | - |
| 5 | FMT | A | 541 | - | 0,2,2 | 0.00 | - | 0,1,1 | 0.00 | - |
| 2 | HEM | A | 501 | 1 | 27,50,50 | 1.68 | 6 (22%) | 17,82,82 | 2.06 | 6 (35%) |
| 5 | FMT | A | 572 | - | 0,2,2 | 0.00 | - | 0,1,1 | 0.00 | - |
| 5 | FMT | B | 519 | - | 0,2,2 | 0.00 | - | 0,1,1 | 0.00 | - |
| 5 | FMT | D | 524 | - | 0,2,2 | 0.00 | - | 0,1,1 | 0.00 | - |
| 5 | FMT | A | 542 | - | 0,2,2 | 0.00 | - | 0,1,1 | 0.00 | - |
| 5 | FMT | A | 521 | - | 0,2,2 | 0.00 | - | 0,1,1 | 0.00 | - |
| 5 | FMT | B | 529 | - | 0,2,2 | 0.00 | - | 0,1,1 | 0.00 | - |
| 5 | FMT | B | 528 | - | 0,2,2 | 0.00 | - | 0,1,1 | 0.00 | - |
| 5 | FMT | A | 514 | - | 0,2,2 | 0.00 | - | 0,1,1 | 0.00 | - |
| 5 | FMT | D | 526 | - | 0,2,2 | 0.00 | - | 0,1,1 | 0.00 | - |
| 5 | FMT | A | 548 | - | 0,2,2 | 0.00 | - | 0,1,1 | 0.00 | - |
| 5 | FMT | C | 534 | - | 0,2,2 | 0.00 | - | 0,1,1 | 0.00 | - |
| 5 | FMT | A | 520 | - | 0,2,2 | 0.00 | - | 0,1,1 | 0.00 | - |
| 5 | FMT | C | 550 | - | 0,2,2 | 0.00 | - | 0,1,1 | 0.00 | - |
| 5 | FMT | A | 573 | - | 0,2,2 | 0.00 | - | 0,1,1 | 0.00 | - |
| 4 | RAM | B | 513 | - | 11,11,11 | 1.77 | 4 (36%) | 15,16,16 | 3.53 | 8 (53%) |
| 5 | FMT | A | 567 | - | 0,2,2 | 0.00 | - | 0,1,1 | 0.00 | - |
| 5 | FMT | E | 501 | - | 0,2,2 | 0.00 | - | 0,1,1 | 0.00 | - |
| 5 | FMT | D | 506 | - | 0,2,2 | 0.00 | - | 0,1,1 | 0.00 | - |
| 5 | FMT | A | 538 | - | 0,2,2 | 0.00 | - | 0,1,1 | 0.00 | - |
| 5 | FMT | B | 527 | - | 0,2,2 | 0.00 | - | 0,1,1 | 0.00 | - |
| 5 | FMT | C | 541 | - | 0,2,2 | 0.00 | - | 0,1,1 | 0.00 | - |
| 5 | FMT | C | 547 | - | 0,2,2 | 0.00 | - | 0,1,1 | 0.00 | - |
| 5 | FMT | A | 551 | - | 0,2,2 | 0.00 | - | 0,1,1 | 0.00 | - |
| 5 | FMT | D | 531 | - | 0,2,2 | 0.00 | - | 0,1,1 | 0.00 | - |
| 5 | FMT | A | 505 | - | 0,2,2 | 0.00 | - | 0,1,1 | 0.00 | - |
| 5 | FMT | C | 538 | - | 0,2,2 | 0.00 | - | 0,1,1 | 0.00 | - |
| 4 | RAM | A | 503[B] | - | 11,11,11 | 1.37 | 2 (18%) | 15,16,16 | 2.60 | 9 (60%) |
| 5 | FMT | A | 524 | - | 0,2,2 | 0.00 | - | 0,1,1 | 0.00 | - |
| 5 | FMT | F | 512 | - | 0,2,2 | 0.00 | - | 0,1,1 | 0.00 | - |
| 5 | FMT | B | 511 | - | 0,2,2 | 0.00 | - | 0,1,1 | 0.00 | - |
| 5 | FMT | A | 530 | - | 0,2,2 | 0.00 | - | 0,1,1 | 0.00 | - |

CONFIDENTIAL VALIDATION REPORT

| Mol | Type | Chain | Res | Link | Bond lengths | | | Bond angles | | |
|---|---|---|---|---|---|---|---|---|---|---|
| | | | | | Counts | RMSZ | #\|Z\| > 2 | Counts | RMSZ | #\|Z\| > 2 |
| 5 | FMT | A | 561 | - | 0,2,2 | 0.00 | - | 0,1,1 | 0.00 | - |
| 5 | FMT | A | 565 | - | 0,2,2 | 0.00 | - | 0,1,1 | 0.00 | - |
| 5 | FMT | A | 537 | - | 0,2,2 | 0.00 | - | 0,1,1 | 0.00 | - |
| 5 | FMT | D | 523 | - | 0,2,2 | 0.00 | - | 0,1,1 | 0.00 | - |
| 5 | FMT | C | 507 | - | 0,2,2 | 0.00 | - | 0,1,1 | 0.00 | - |
| 5 | FMT | A | 545 | - | 0,2,2 | 0.00 | - | 0,1,1 | 0.00 | - |
| 5 | FMT | C | 548 | - | 0,2,2 | 0.00 | - | 0,1,1 | 0.00 | - |
| 5 | FMT | F | 514 | - | 0,2,2 | 0.00 | - | 0,1,1 | 0.00 | - |
| 5 | FMT | A | 508 | - | 0,2,2 | 0.00 | - | 0,1,1 | 0.00 | - |
| 5 | FMT | B | 522 | - | 0,2,2 | 0.00 | - | 0,1,1 | 0.00 | - |
| 5 | FMT | C | 544 | - | 0,2,2 | 0.00 | - | 0,1,1 | 0.00 | - |
| 5 | FMT | A | 504 | - | 0,2,2 | 0.00 | - | 0,1,1 | 0.00 | - |
| 5 | FMT | A | 569 | - | 0,2,2 | 0.00 | - | 0,1,1 | 0.00 | - |
| 5 | FMT | A | 517 | - | 0,2,2 | 0.00 | - | 0,1,1 | 0.00 | - |
| 5 | FMT | C | 504 | - | 0,2,2 | 0.00 | - | 0,1,1 | 0.00 | - |
| 5 | FMT | C | 542 | - | 0,2,2 | 0.00 | - | 0,1,1 | 0.00 | - |

In the following table, the Chirals column lists the number of chiral outliers, the number of chiral centers analysed, the number of these observed in the model and the number defined in the Chemical Component Dictionary. Similar counts are reported in the Torsion and Rings columns. '-' means no outliers of that kind were identified.

| Mol | Type | Chain | Res | Link | Chirals | Torsions | Rings |
|---|---|---|---|---|---|---|---|
| 4 | RAM | A | 503[A] | - | - | - | 0/1/1/1 |
| 2 | HEM | D | 501 | 1 | - | 0/6/54/54 | - |
| 3 | QR8 | C | 502 | - | - | 14/48/48/48 | 0/1/1/1 |
| 2 | HEM | F | 502 | 1 | - | 0/6/54/54 | - |
| 3 | QR8 | F | 503 | - | - | 13/48/48/48 | 0/1/1/1 |
| 3 | QR8 | E | 503 | - | - | 15/48/48/48 | 0/1/1/1 |
| 2 | HEM | E | 502 | 1 | - | 0/6/54/54 | - |
| 4 | RAM | B | 513 | - | - | - | 0/1/1/1 |
| 4 | RAM | A | 503[B] | - | - | - | 0/1/1/1 |
| 3 | QR8 | D | 502 | - | - | 13/48/48/48 | 0/1/1/1 |
| 2 | HEM | A | 501 | 1 | - | 0/6/54/54 | - |
| 3 | QR8 | B | 502 | - | - | 14/48/48/48 | 0/1/1/1 |
| 2 | HEM | C | 501 | 1 | - | 0/6/54/54 | - |
| 4 | RAM | D | 503 | - | - | - | 0/1/1/1 |
| 3 | QR8 | A | 502 | - | - | 13/48/48/48 | 0/1/1/1 |
| 2 | HEM | B | 501 | 1 | - | 0/6/54/54 | - |

All (55) bond length outliers are listed below:

CONFIDENTIAL VALIDATION REPORT

| Mol | Chain | Res | Type | Atoms | Z | Observed(Å) | Ideal(Å) |
|---|---|---|---|---|---|---|---|
| 3 | B | 502 | QR8 | O2-C13 | -5.69 | 1.37 | 1.46 |
| 2 | C | 501 | HEM | C3B-C2B | -5.33 | 1.33 | 1.40 |
| 3 | C | 502 | QR8 | O2-C13 | -5.12 | 1.38 | 1.46 |
| 2 | A | 501 | HEM | C3B-C2B | -4.86 | 1.33 | 1.40 |
| 3 | A | 502 | QR8 | O2-C13 | -4.73 | 1.39 | 1.46 |
| 3 | D | 502 | QR8 | O2-C13 | -4.63 | 1.39 | 1.46 |
| 3 | F | 503 | QR8 | O2-C13 | -4.34 | 1.39 | 1.46 |
| 2 | D | 501 | HEM | C1D-ND | -4.29 | 1.27 | 1.36 |
| 2 | D | 501 | HEM | C3B-C2B | -4.24 | 1.34 | 1.40 |
| 3 | E | 503 | QR8 | O2-C13 | -4.13 | 1.39 | 1.46 |
| 3 | F | 503 | QR8 | O2-C1 | 4.02 | 1.43 | 1.34 |
| 3 | D | 502 | QR8 | C10-C9 | -3.89 | 1.46 | 1.52 |
| 3 | E | 503 | QR8 | O2-C1 | 3.78 | 1.43 | 1.34 |
| 2 | C | 501 | HEM | C3B-CAB | -3.68 | 1.40 | 1.47 |
| 3 | F | 503 | QR8 | C10-C9 | -3.53 | 1.47 | 1.52 |
| 2 | B | 501 | HEM | C3C-C2C | -3.50 | 1.35 | 1.40 |
| 3 | A | 502 | QR8 | O2-C1 | 3.39 | 1.42 | 1.34 |
| 3 | D | 502 | QR8 | O2-C1 | 3.28 | 1.41 | 1.34 |
| 3 | B | 502 | QR8 | O2-C1 | 3.25 | 1.41 | 1.34 |
| 3 | B | 502 | QR8 | C10-C9 | -3.22 | 1.47 | 1.52 |
| 4 | B | 513 | RAM | O3-C3 | 3.10 | 1.50 | 1.43 |
| 2 | B | 501 | HEM | C3B-C2B | -3.07 | 1.36 | 1.40 |
| 4 | B | 513 | RAM | C4-C5 | 3.05 | 1.59 | 1.52 |
| 2 | A | 501 | HEM | C1D-CHD | -3.03 | 1.32 | 1.41 |
| 2 | A | 501 | HEM | C4D-C3D | 2.96 | 1.49 | 1.42 |
| 3 | C | 502 | QR8 | O2-C1 | 2.87 | 1.41 | 1.34 |
| 2 | D | 501 | HEM | C4D-C3D | 2.85 | 1.49 | 1.42 |
| 4 | A | 503[B] | RAM | C6-C5 | -2.85 | 1.44 | 1.51 |
| 4 | B | 513 | RAM | C3-C2 | 2.67 | 1.59 | 1.52 |
| 2 | E | 502 | HEM | C3B-C2B | -2.66 | 1.36 | 1.40 |
| 2 | D | 501 | HEM | C3C-C2C | -2.65 | 1.36 | 1.40 |
| 2 | B | 501 | HEM | CMD-C2D | -2.65 | 1.46 | 1.51 |
| 3 | A | 502 | QR8 | C10-C9 | -2.59 | 1.48 | 1.52 |
| 3 | E | 503 | QR8 | C10-C9 | -2.55 | 1.48 | 1.52 |
| 4 | A | 503[B] | RAM | O5-C1 | 2.47 | 1.49 | 1.42 |
| 4 | D | 503 | RAM | O3-C3 | 2.40 | 1.48 | 1.43 |
| 2 | C | 501 | HEM | C1D-CHD | -2.40 | 1.34 | 1.41 |
| 2 | E | 502 | HEM | C4D-C3D | 2.38 | 1.48 | 1.42 |
| 3 | A | 502 | QR8 | C7-C6 | -2.37 | 1.50 | 1.54 |
| 4 | A | 503[A] | RAM | C6-C5 | -2.36 | 1.45 | 1.51 |
| 4 | B | 513 | RAM | C6-C5 | 2.35 | 1.57 | 1.51 |
| 2 | A | 501 | HEM | C4A-NA | 2.33 | 1.41 | 1.36 |
| 2 | A | 501 | HEM | C1A-CHA | -2.32 | 1.34 | 1.41 |

*Continued on next page...*

CONFIDENTIAL VALIDATION REPORT

*Continued from previous page...*

| Mol | Chain | Res | Type | Atoms | Z | Observed(Å) | Ideal(Å) |
|---|---|---|---|---|---|---|---|
| 2 | E | 502 | HEM | C4A-NA | 2.29 | 1.40 | 1.36 |
| 3 | D | 502 | QR8 | C7-C6 | -2.28 | 1.50 | 1.54 |
| 2 | E | 502 | HEM | C1A-NA | 2.26 | 1.40 | 1.36 |
| 3 | B | 502 | QR8 | C7-C6 | -2.25 | 1.50 | 1.54 |
| 4 | A | 503[A] | RAM | O3-C3 | -2.24 | 1.37 | 1.43 |
| 2 | B | 501 | HEM | C3C-CAC | -2.16 | 1.43 | 1.47 |
| 2 | B | 501 | HEM | C1D-ND | -2.12 | 1.31 | 1.36 |
| 2 | F | 502 | HEM | C4D-C3D | 2.05 | 1.47 | 1.42 |
| 3 | E | 503 | QR8 | C10-C11 | -2.04 | 1.49 | 1.53 |
| 4 | A | 503[A] | RAM | C1-C2 | -2.04 | 1.47 | 1.52 |
| 2 | B | 501 | HEM | C1A-NA | 2.01 | 1.40 | 1.36 |
| 2 | A | 501 | HEM | CAA-C2A | 2.01 | 1.55 | 1.52 |

All (125) bond angle outliers are listed below:

| Mol | Chain | Res | Type | Atoms | Z | Observed(°) | Ideal(°) |
|---|---|---|---|---|---|---|---|
| 4 | A | 503[A] | RAM | O1-C1-C2 | -8.65 | 84.68 | 109.03 |
| 4 | A | 503[A] | RAM | O3-C3-C2 | -6.69 | 94.89 | 110.35 |
| 4 | B | 513 | RAM | C6-C5-C4 | 5.66 | 123.54 | 113.07 |
| 4 | B | 513 | RAM | O3-C3-C2 | 5.50 | 123.06 | 110.35 |
| 2 | C | 501 | HEM | CBA-CAA-C2A | 5.40 | 122.44 | 112.49 |
| 4 | B | 513 | RAM | C4-C3-C2 | -5.02 | 102.06 | 110.82 |
| 3 | A | 502 | QR8 | C8-C9-C10 | -4.94 | 110.53 | 119.10 |
| 4 | A | 503[A] | RAM | O4-C4-C3 | 4.78 | 121.40 | 110.35 |
| 4 | B | 513 | RAM | O2-C2-C3 | 4.72 | 121.27 | 110.35 |
| 2 | D | 501 | HEM | CBD-CAD-C3D | -4.72 | 103.78 | 112.48 |
| 2 | A | 501 | HEM | CAD-CBD-CGD | 4.70 | 120.56 | 112.67 |
| 4 | B | 513 | RAM | C3-C4-C5 | -4.66 | 102.51 | 109.77 |
| 4 | B | 513 | RAM | O3-C3-C4 | 4.57 | 120.92 | 110.35 |
| 3 | C | 502 | QR8 | C8-C9-C10 | -4.50 | 111.29 | 119.10 |
| 4 | A | 503[B] | RAM | C6-C5-C4 | -4.49 | 104.78 | 113.07 |
| 2 | B | 501 | HEM | CMA-C3A-C4A | -4.40 | 121.69 | 128.46 |
| 2 | B | 501 | HEM | C4A-C3A-C2A | 4.23 | 109.94 | 107.00 |
| 3 | B | 502 | QR8 | C36-C13-C12 | -4.23 | 108.26 | 114.39 |
| 4 | D | 503 | RAM | O1-C1-C2 | 4.19 | 120.82 | 109.03 |
| 4 | A | 503[B] | RAM | O3-C3-C4 | -4.11 | 100.84 | 110.35 |
| 3 | D | 502 | QR8 | C8-C9-C10 | -4.11 | 111.96 | 119.10 |
| 4 | B | 513 | RAM | O4-C4-C5 | 4.09 | 118.74 | 109.67 |
| 2 | A | 501 | HEM | C4A-C3A-C2A | 4.02 | 109.79 | 107.00 |
| 3 | D | 502 | QR8 | O11-C9-C8 | 3.96 | 128.61 | 121.26 |
| 2 | E | 502 | HEM | CBD-CAD-C3D | -3.95 | 105.20 | 112.48 |
| 3 | D | 502 | QR8 | C34-C10-C11 | 3.94 | 118.95 | 112.37 |

*Continued on next page...*

CONFIDENTIAL VALIDATION REPORT

*Continued from previous page...*

| Mol | Chain | Res | Type | Atoms | Z | Observed(°) | Ideal(°) |
|---|---|---|---|---|---|---|---|
| 2 | E | 502 | HEM | CBA-CAA-C2A | 3.93 | 119.73 | 112.49 |
| 2 | C | 501 | HEM | CMA-C3A-C4A | 3.92 | 134.49 | 128.46 |
| 4 | D | 503 | RAM | O2-C2-C1 | 3.83 | 118.04 | 109.16 |
| 4 | D | 503 | RAM | O5-C1-C2 | -3.83 | 103.46 | 110.28 |
| 4 | D | 503 | RAM | O3-C3-C2 | 3.82 | 119.19 | 110.35 |
| 3 | A | 502 | QR8 | O11-C9-C8 | 3.78 | 128.28 | 121.26 |
| 3 | D | 502 | QR8 | C36-C13-C12 | -3.76 | 108.94 | 114.39 |
| 2 | C | 501 | HEM | CMA-C3A-C2A | -3.70 | 117.97 | 124.94 |
| 4 | A | 503[B] | RAM | C1-C2-C3 | -3.63 | 102.79 | 110.31 |
| 2 | B | 501 | HEM | CMC-C2C-C3C | 3.59 | 131.40 | 124.68 |
| 3 | B | 502 | QR8 | C8-C9-C10 | -3.56 | 112.92 | 119.10 |
| 3 | E | 503 | QR8 | C8-C9-C10 | -3.55 | 112.93 | 119.10 |
| 2 | E | 502 | HEM | C1D-C2D-C3D | -3.55 | 104.53 | 107.00 |
| 4 | D | 503 | RAM | C4-C3-C2 | -3.50 | 104.71 | 110.82 |
| 3 | C | 502 | QR8 | C34-C10-C9 | 3.48 | 114.13 | 108.08 |
| 3 | E | 503 | QR8 | O2-C1-C2 | 3.42 | 119.07 | 111.56 |
| 2 | B | 501 | HEM | C4C-C3C-C2C | 3.41 | 109.28 | 106.90 |
| 3 | F | 503 | QR8 | C32-C6-C5 | -3.40 | 105.24 | 111.54 |
| 2 | E | 502 | HEM | CMC-C2C-C3C | 3.39 | 131.02 | 124.68 |
| 3 | E | 503 | QR8 | C36-C13-C12 | -3.36 | 109.53 | 114.39 |
| 4 | B | 513 | RAM | C1-C2-C3 | 3.32 | 117.21 | 110.31 |
| 3 | A | 502 | QR8 | C7-C8-C9 | 3.32 | 118.64 | 110.85 |
| 3 | F | 503 | QR8 | O2-C1-C2 | 3.31 | 118.83 | 111.56 |
| 4 | A | 503[A] | RAM | O2-C2-C1 | -3.30 | 101.51 | 109.16 |
| 3 | C | 502 | QR8 | C34-C10-C11 | 3.30 | 117.87 | 112.37 |
| 4 | D | 503 | RAM | C1-C2-C3 | -3.22 | 103.63 | 110.31 |
| 3 | E | 503 | QR8 | C32-C6-C7 | -3.20 | 105.89 | 110.69 |
| 2 | A | 501 | HEM | CBA-CAA-C2A | 3.18 | 118.35 | 112.49 |
| 4 | A | 503[B] | RAM | O5-C1-C2 | -3.16 | 104.65 | 110.28 |
| 3 | A | 502 | QR8 | C32-C6-C7 | -3.15 | 105.97 | 110.69 |
| 4 | A | 503[B] | RAM | O3-C3-C2 | 3.13 | 117.59 | 110.35 |
| 2 | C | 501 | HEM | CMC-C2C-C3C | 3.13 | 130.53 | 124.68 |
| 2 | F | 502 | HEM | CMC-C2C-C3C | 3.11 | 130.49 | 124.68 |
| 3 | C | 502 | QR8 | O11-C9-C10 | 3.10 | 125.01 | 120.60 |
| 3 | B | 502 | QR8 | O2-C1-C2 | 3.04 | 118.22 | 111.56 |
| 3 | A | 502 | QR8 | C34-C10-C11 | 3.03 | 117.43 | 112.37 |
| 3 | B | 502 | QR8 | C34-C10-C11 | 3.01 | 117.40 | 112.37 |
| 4 | A | 503[B] | RAM | O1-C1-O5 | 2.98 | 119.31 | 110.38 |
| 3 | B | 502 | QR8 | C32-C6-C7 | -2.95 | 106.28 | 110.69 |
| 3 | C | 502 | QR8 | O2-C1-O1 | -2.94 | 118.45 | 123.94 |
| 3 | F | 503 | QR8 | C36-C13-C12 | -2.90 | 110.19 | 114.39 |
| 3 | E | 503 | QR8 | C7-C8-C9 | 2.90 | 117.66 | 110.85 |

*Continued on next page...*

CONFIDENTIAL VALIDATION REPORT

*Continued from previous page...*

| Mol | Chain | Res | Type | Atoms | Z | Observed(°) | Ideal(°) |
|---|---|---|---|---|---|---|---|
| 3 | D | 502 | QR8 | O2-C1-C2 | 2.89 | 117.89 | 111.56 |
| 3 | A | 502 | QR8 | C36-C13-C12 | -2.88 | 110.22 | 114.39 |
| 4 | D | 503 | RAM | O5-C5-C4 | -2.85 | 104.40 | 109.52 |
| 3 | F | 503 | QR8 | C10-C11-C12 | -2.83 | 108.57 | 114.41 |
| 3 | A | 502 | QR8 | C32-C6-C5 | -2.80 | 106.34 | 111.54 |
| 3 | E | 503 | QR8 | C32-C6-C5 | -2.78 | 106.38 | 111.54 |
| 2 | A | 501 | HEM | CMA-C3A-C4A | -2.77 | 124.21 | 128.46 |
| 2 | D | 501 | HEM | CMA-C3A-C4A | -2.76 | 124.22 | 128.46 |
| 3 | A | 502 | QR8 | C2-C3-C4 | -2.76 | 108.72 | 114.41 |
| 2 | B | 501 | HEM | CAD-CBD-CGD | 2.74 | 117.27 | 112.67 |
| 3 | F | 503 | QR8 | O7-C5-C6 | -2.72 | 104.72 | 109.83 |
| 3 | C | 502 | QR8 | O2-C1-C2 | 2.72 | 117.52 | 111.56 |
| 3 | B | 502 | QR8 | C7-C8-C9 | 2.71 | 117.22 | 110.85 |
| 3 | D | 502 | QR8 | C7-C8-C9 | 2.67 | 117.13 | 110.85 |
| 3 | D | 502 | QR8 | C2-C3-C4 | -2.65 | 108.94 | 114.41 |
| 4 | D | 503 | RAM | O4-C4-C5 | 2.65 | 115.53 | 109.67 |
| 2 | D | 501 | HEM | C4A-C3A-C2A | 2.65 | 108.84 | 107.00 |
| 3 | C | 502 | QR8 | C7-C8-C9 | 2.65 | 117.06 | 110.85 |
| 2 | F | 502 | HEM | C4A-C3A-C2A | 2.64 | 108.83 | 107.00 |
| 2 | F | 502 | HEM | CBD-CAD-C3D | -2.62 | 107.65 | 112.48 |
| 3 | E | 503 | QR8 | O2-C1-O1 | -2.61 | 119.06 | 123.94 |
| 4 | A | 503[A] | RAM | O5-C1-C2 | 2.61 | 114.94 | 110.28 |
| 3 | A | 502 | QR8 | O2-C1-C2 | 2.58 | 117.23 | 111.56 |
| 2 | C | 501 | HEM | CAD-CBD-CGD | 2.57 | 116.98 | 112.67 |
| 3 | F | 503 | QR8 | C35-C12-C13 | -2.57 | 108.86 | 112.18 |
| 4 | A | 503[B] | RAM | O5-C5-C6 | 2.55 | 112.22 | 106.70 |
| 3 | D | 502 | QR8 | C11-C10-C9 | -2.54 | 105.76 | 110.36 |
| 3 | F | 503 | QR8 | C2-C3-C4 | -2.53 | 109.19 | 114.41 |
| 3 | B | 502 | QR8 | O11-C9-C8 | 2.44 | 125.80 | 121.26 |
| 3 | B | 502 | QR8 | C2-C3-C4 | -2.38 | 109.51 | 114.41 |
| 4 | A | 503[A] | RAM | C6-C5-C4 | -2.37 | 108.69 | 113.07 |
| 3 | E | 503 | QR8 | C6-C5-C4 | -2.33 | 112.66 | 116.27 |
| 3 | F | 503 | QR8 | O2-C1-O1 | -2.33 | 119.59 | 123.94 |
| 3 | B | 502 | QR8 | C32-C6-C5 | -2.31 | 107.25 | 111.54 |
| 2 | D | 501 | HEM | CBA-CAA-C2A | 2.29 | 116.70 | 112.49 |
| 3 | E | 503 | QR8 | C11-C10-C9 | -2.29 | 106.22 | 110.36 |
| 3 | E | 503 | QR8 | C34-C10-C9 | 2.27 | 112.03 | 108.08 |
| 3 | B | 502 | QR8 | C11-C10-C9 | -2.27 | 106.24 | 110.36 |
| 2 | A | 501 | HEM | CBD-CAD-C3D | -2.27 | 108.30 | 112.48 |
| 3 | F | 503 | QR8 | C13-O2-C1 | -2.25 | 114.25 | 117.51 |
| 4 | A | 503[B] | RAM | O2-C2-C3 | 2.24 | 115.52 | 110.35 |
| 4 | A | 503[A] | RAM | O2-C2-C3 | 2.24 | 115.52 | 110.35 |

*Continued on next page...*

CONFIDENTIAL VALIDATION REPORT

Continued from previous page...

| Mol | Chain | Res | Type | Atoms | Z | Observed(°) | Ideal(°) |
|---|---|---|---|---|---|---|---|
| 2 | F | 502 | HEM | C1D-C2D-C3D | -2.21 | 105.45 | 107.00 |
| 2 | A | 501 | HEM | CMC-C2C-C3C | 2.20 | 128.79 | 124.68 |
| 4 | A | 503[A] | RAM | O3-C3-C4 | 2.20 | 115.43 | 110.35 |
| 4 | A | 503[B] | RAM | O5-C5-C4 | -2.19 | 105.59 | 109.52 |
| 2 | C | 501 | HEM | CMD-C2D-C1D | -2.19 | 125.10 | 128.46 |
| 2 | F | 502 | HEM | CBA-CAA-C2A | 2.17 | 116.49 | 112.49 |
| 3 | D | 502 | QR8 | O2-C1-O1 | -2.16 | 119.91 | 123.94 |
| 4 | A | 503[A] | RAM | C3-C4-C5 | -2.14 | 106.43 | 109.77 |
| 2 | E | 502 | HEM | C3B-C4B-NB | -2.11 | 106.48 | 109.21 |
| 2 | B | 501 | HEM | CMB-C2B-C3B | 2.10 | 128.61 | 124.68 |
| 2 | D | 501 | HEM | CAD-CBD-CGD | 2.10 | 116.20 | 112.67 |
| 2 | C | 501 | HEM | C1D-C2D-C3D | 2.08 | 108.44 | 107.00 |
| 4 | D | 503 | RAM | O2-C2-C3 | 2.08 | 115.15 | 110.35 |
| 2 | F | 502 | HEM | CMA-C3A-C4A | -2.03 | 125.35 | 128.46 |
| 4 | D | 503 | RAM | O5-C5-C6 | 2.02 | 111.05 | 106.70 |

There are no chirality outliers.

All (82) torsion outliers are listed below:

| Mol | Chain | Res | Type | Atoms |
|---|---|---|---|---|
| 3 | F | 503 | QR8 | C30-C2-C3-C4 |
| 3 | D | 502 | QR8 | C6-C7-C8-C9 |
| 3 | D | 502 | QR8 | C6-C7-C8-C33 |
| 3 | D | 502 | QR8 | C31-C4-C5-O7 |
| 3 | D | 502 | QR8 | C3-C4-C5-O7 |
| 3 | D | 502 | QR8 | C30-C2-C3-C4 |
| 3 | D | 502 | QR8 | C1-C2-C3-O3 |
| 3 | B | 502 | QR8 | C6-C7-C8-C9 |
| 3 | B | 502 | QR8 | C1-C2-C3-O3 |
| 3 | E | 503 | QR8 | C6-C7-C8-C9 |
| 3 | A | 502 | QR8 | C6-C7-C8-C9 |
| 3 | A | 502 | QR8 | C1-C2-C3-O3 |
| 3 | C | 502 | QR8 | C6-C7-C8-C9 |
| 3 | C | 502 | QR8 | C6-C7-C8-C33 |
| 3 | C | 502 | QR8 | C1-C2-C3-O3 |
| 3 | F | 503 | QR8 | C3-C4-C5-O7 |
| 3 | B | 502 | QR8 | C3-C4-C5-O7 |
| 3 | E | 503 | QR8 | C3-C4-C5-O7 |
| 3 | A | 502 | QR8 | C3-C4-C5-O7 |
| 3 | C | 502 | QR8 | C3-C4-C5-O7 |
| 3 | F | 503 | QR8 | C30-C2-C3-O3 |
| 3 | D | 502 | QR8 | C30-C2-C3-O3 |

Continued on next page...

CONFIDENTIAL VALIDATION REPORT

Continued from previous page...

| Mol | Chain | Res | Type | Atoms |
|---|---|---|---|---|
| 3 | B | 502 | QR8 | C30-C2-C3-O3 |
| 3 | A | 502 | QR8 | C30-C2-C3-O3 |
| 3 | C | 502 | QR8 | C30-C2-C3-O3 |
| 3 | B | 502 | QR8 | C30-C2-C3-C4 |
| 3 | A | 502 | QR8 | C30-C2-C3-C4 |
| 3 | C | 502 | QR8 | C30-C2-C3-C4 |
| 3 | F | 503 | QR8 | C31-C4-C5-O7 |
| 3 | B | 502 | QR8 | C31-C4-C5-O7 |
| 3 | E | 503 | QR8 | C31-C4-C5-O7 |
| 3 | A | 502 | QR8 | C31-C4-C5-O7 |
| 3 | C | 502 | QR8 | C31-C4-C5-O7 |
| 3 | E | 503 | QR8 | C6-C7-C8-C33 |
| 3 | A | 502 | QR8 | C6-C7-C8-C33 |
| 3 | F | 503 | QR8 | C3-C4-C5-C6 |
| 3 | D | 502 | QR8 | C3-C4-C5-C6 |
| 3 | E | 503 | QR8 | C30-C2-C3-C4 |
| 3 | D | 502 | QR8 | C31-C4-C5-C6 |
| 3 | B | 502 | QR8 | C5-C6-C7-C8 |
| 3 | E | 503 | QR8 | C30-C2-C3-O3 |
| 3 | F | 503 | QR8 | C7-C8-C9-C10 |
| 3 | F | 503 | QR8 | C31-C4-C5-C6 |
| 3 | A | 502 | QR8 | C31-C4-C5-C6 |
| 3 | C | 502 | QR8 | C31-C4-C5-C6 |
| 3 | B | 502 | QR8 | C31-C4-C5-C6 |
| 3 | E | 503 | QR8 | C31-C4-C5-C6 |
| 3 | B | 502 | QR8 | C3-C4-C5-C6 |
| 3 | E | 503 | QR8 | C3-C4-C5-C6 |
| 3 | C | 502 | QR8 | C3-C4-C5-C6 |
| 3 | A | 502 | QR8 | C3-C4-C5-C6 |
| 3 | D | 502 | QR8 | C32-C6-C7-C8 |
| 3 | A | 502 | QR8 | C32-C6-C7-C8 |
| 3 | F | 503 | QR8 | C1-C2-C3-C4 |
| 3 | F | 503 | QR8 | C1-C2-C3-O3 |
| 3 | D | 502 | QR8 | C1-C2-C3-C4 |
| 3 | B | 502 | QR8 | C1-C2-C3-C4 |
| 3 | E | 503 | QR8 | C1-C2-C3-C4 |
| 3 | E | 503 | QR8 | C1-C2-C3-O3 |
| 3 | A | 502 | QR8 | C1-C2-C3-C4 |
| 3 | C | 502 | QR8 | C1-C2-C3-C4 |
| 3 | B | 502 | QR8 | O7-C5-C6-C7 |
| 3 | B | 502 | QR8 | C6-C7-C8-C33 |
| 3 | D | 502 | QR8 | C5-C6-C7-C8 |

Continued on next page...

CONFIDENTIAL VALIDATION REPORT

*Continued from previous page...*

| Mol | Chain | Res | Type | Atoms |
|---|---|---|---|---|
| 3 | E | 503 | QR8 | C5-C6-C7-C8 |
| 3 | A | 502 | QR8 | C5-C6-C7-C8 |
| 3 | F | 503 | QR8 | C33-C8-C9-C10 |
| 3 | A | 502 | QR8 | C33-C8-C9-C10 |
| 3 | B | 502 | QR8 | C32-C6-C7-C8 |
| 3 | E | 503 | QR8 | C32-C6-C7-C8 |
| 3 | F | 503 | QR8 | C33-C8-C9-O11 |
| 3 | B | 502 | QR8 | C4-C5-C6-C7 |
| 3 | C | 502 | QR8 | O1-C1-O2-C13 |
| 3 | E | 503 | QR8 | C2-C1-O2-C13 |
| 3 | C | 502 | QR8 | C32-C6-C7-C8 |
| 3 | C | 502 | QR8 | C12-C13-O2-C1 |
| 3 | F | 503 | QR8 | O12-C11-C12-C35 |
| 3 | C | 502 | QR8 | C2-C1-O2-C13 |
| 3 | D | 502 | QR8 | C33-C8-C9-C10 |
| 3 | E | 503 | QR8 | C33-C8-C9-C10 |
| 3 | F | 503 | QR8 | C7-C8-C9-O11 |
| 3 | E | 503 | QR8 | C4-C5-C6-C7 |

There are no ring outliers.

50 monomers are involved in 139 short contacts:

| Mol | Chain | Res | Type | Clashes | Symm-Clashes |
|---|---|---|---|---|---|
| 5 | A | 511 | FMT | 2 | 0 |
| 5 | F | 505 | FMT | 2 | 0 |
| 5 | D | 512 | FMT | 1 | 0 |
| 5 | A | 550 | FMT | 2 | 0 |
| 5 | F | 509 | FMT | 2 | 0 |
| 5 | C | 552 | FMT | 2 | 0 |
| 5 | A | 546 | FMT | 1 | 0 |
| 3 | D | 502 | QR8 | 2 | 0 |
| 3 | B | 502 | QR8 | 1 | 0 |
| 2 | D | 501 | HEM | 4 | 0 |
| 5 | A | 506 | FMT | 1 | 0 |
| 4 | D | 503 | RAM | 14 | 0 |
| 2 | C | 501 | HEM | 3 | 0 |
| 5 | D | 518 | FMT | 1 | 0 |
| 5 | C | 523 | FMT | 1 | 0 |
| 5 | B | 503 | FMT | 2 | 0 |
| 5 | D | 528 | FMT | 2 | 0 |
| 5 | C | 512 | FMT | 1 | 0 |
| 5 | B | 507 | FMT | 1 | 0 |

*Continued on next page...*

CONFIDENTIAL VALIDATION REPORT

*Continued from previous page...*

| Mol | Chain | Res | Type | Clashes | Symm-Clashes |
|---|---|---|---|---|---|
| 4 | A | 503[A] | RAM | 17 | 0 |
| 5 | D | 507 | FMT | 1 | 0 |
| 2 | E | 502 | HEM | 3 | 0 |
| 5 | B | 538 | FMT | 1 | 0 |
| 5 | B | 534 | FMT | 2 | 0 |
| 5 | D | 504 | FMT | 2 | 0 |
| 5 | A | 531 | FMT | 2 | 0 |
| 3 | A | 502 | QR8 | 3 | 0 |
| 5 | C | 514 | FMT | 1 | 0 |
| 5 | C | 521 | FMT | 1 | 0 |
| 5 | B | 510 | FMT | 1 | 0 |
| 2 | B | 501 | HEM | 7 | 0 |
| 5 | D | 508 | FMT | 1 | 0 |
| 5 | A | 510 | FMT | 2 | 0 |
| 5 | F | 504 | FMT | 1 | 0 |
| 2 | F | 502 | HEM | 4 | 0 |
| 2 | A | 501 | HEM | 4 | 0 |
| 5 | A | 514 | FMT | 1 | 0 |
| 4 | B | 513 | RAM | 11 | 0 |
| 5 | A | 567 | FMT | 0 | 1 |
| 5 | E | 501 | FMT | 2 | 0 |
| 5 | A | 505 | FMT | 1 | 0 |
| 4 | A | 503[B] | RAM | 17 | 0 |
| 5 | A | 524 | FMT | 1 | 0 |
| 5 | B | 511 | FMT | 2 | 0 |
| 5 | A | 530 | FMT | 1 | 0 |
| 5 | A | 565 | FMT | 6 | 0 |
| 5 | C | 507 | FMT | 2 | 0 |
| 5 | F | 514 | FMT | 5 | 0 |
| 5 | A | 504 | FMT | 1 | 0 |
| 5 | C | 504 | FMT | 1 | 0 |

The following is a two-dimensional graphical depiction of Mogul quality analysis of bond lengths, bond angles, torsion angles, and ring geometry for all instances of the Ligand of Interest. In addition, ligands with molecular weight > 250 and outliers as shown on the validation Tables will also be included. For torsion angles, if less then 5% of the Mogul distribution of torsion angles is within 10 degrees of the torsion angle in question, then that torsion angle is considered an outlier. Any bond that is central to one or more torsion angles identified as an outlier by Mogul will be highlighted in the graph. For rings, the root-mean-square deviation (RMSD) between the ring in question and similar rings identified by Mogul is calculated over all ring torsion angles. If the average RMSD is greater than 60 degrees and the minimal RMSD between the ring in question and any Mogul-identified rings is also greater than 60 degrees, then that ring is considered an outlier. The outliers are highlighted in purple. The color gray indicates Mogul did not find sufficient

CONFIDENTIAL VALIDATION REPORT

equivalents in the CSD to analyse the geometry.

**Ligand QR8 F 503**

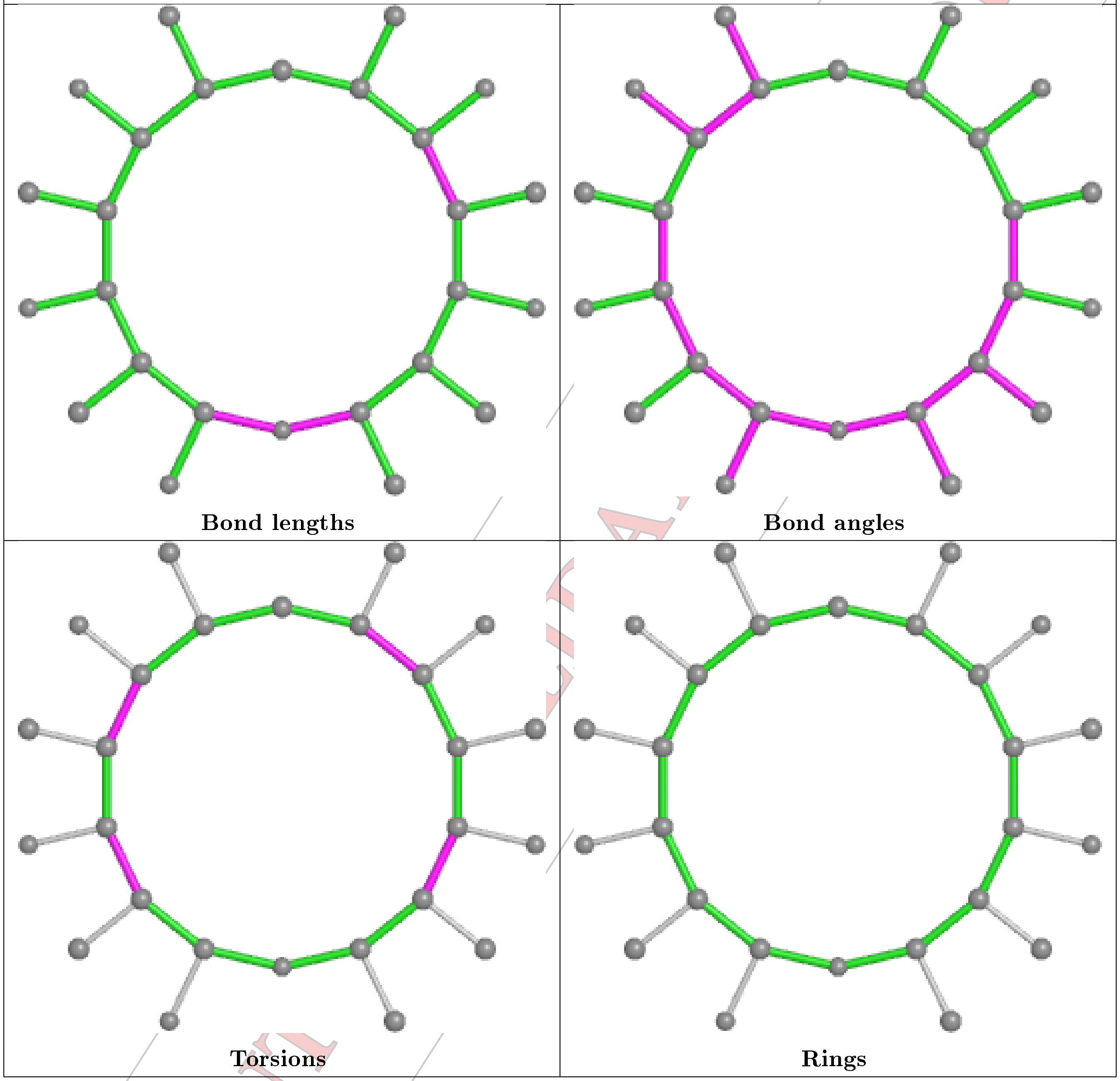

**Ligand QR8 D 502**

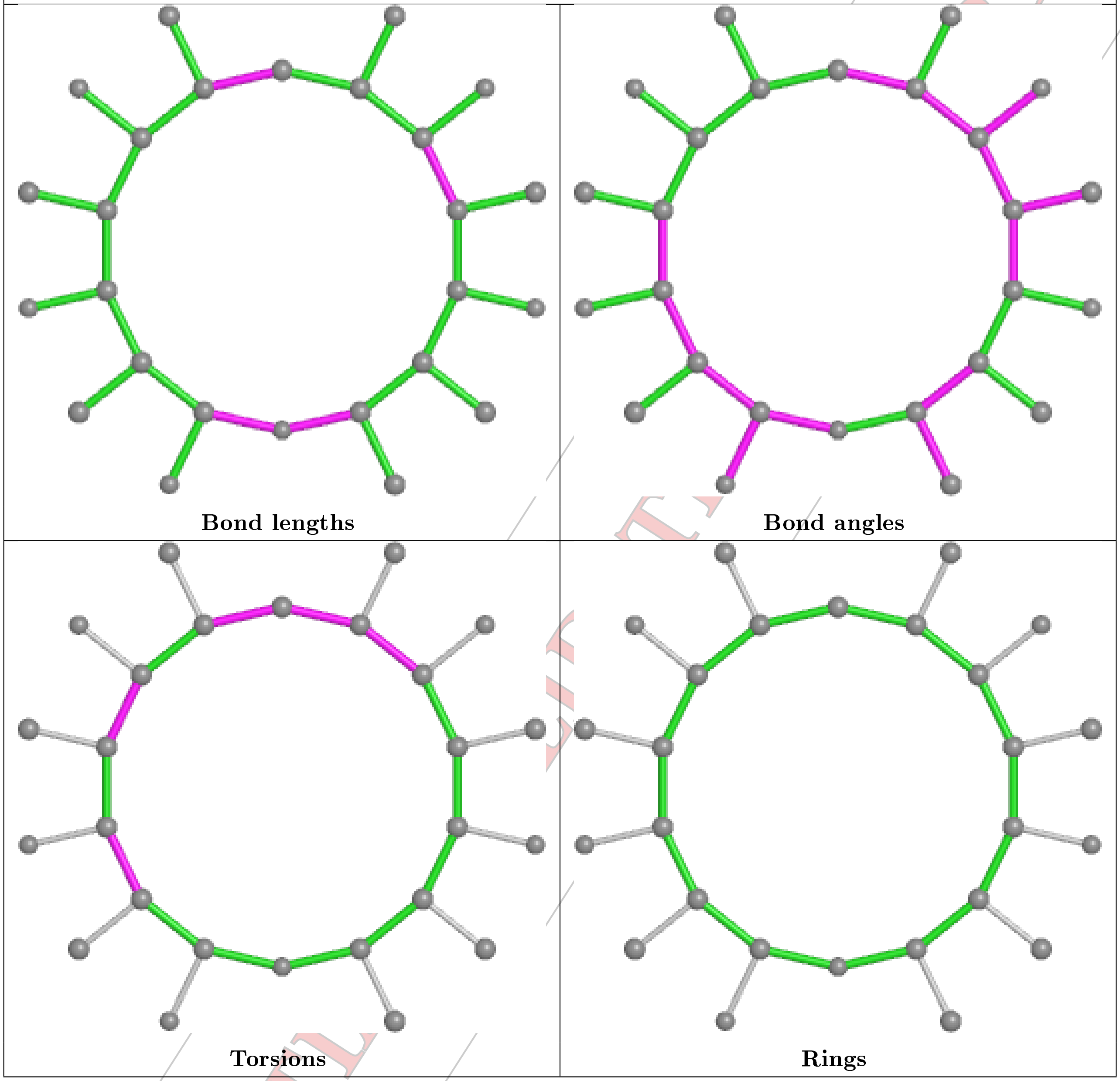

**Ligand QR8 B 502**

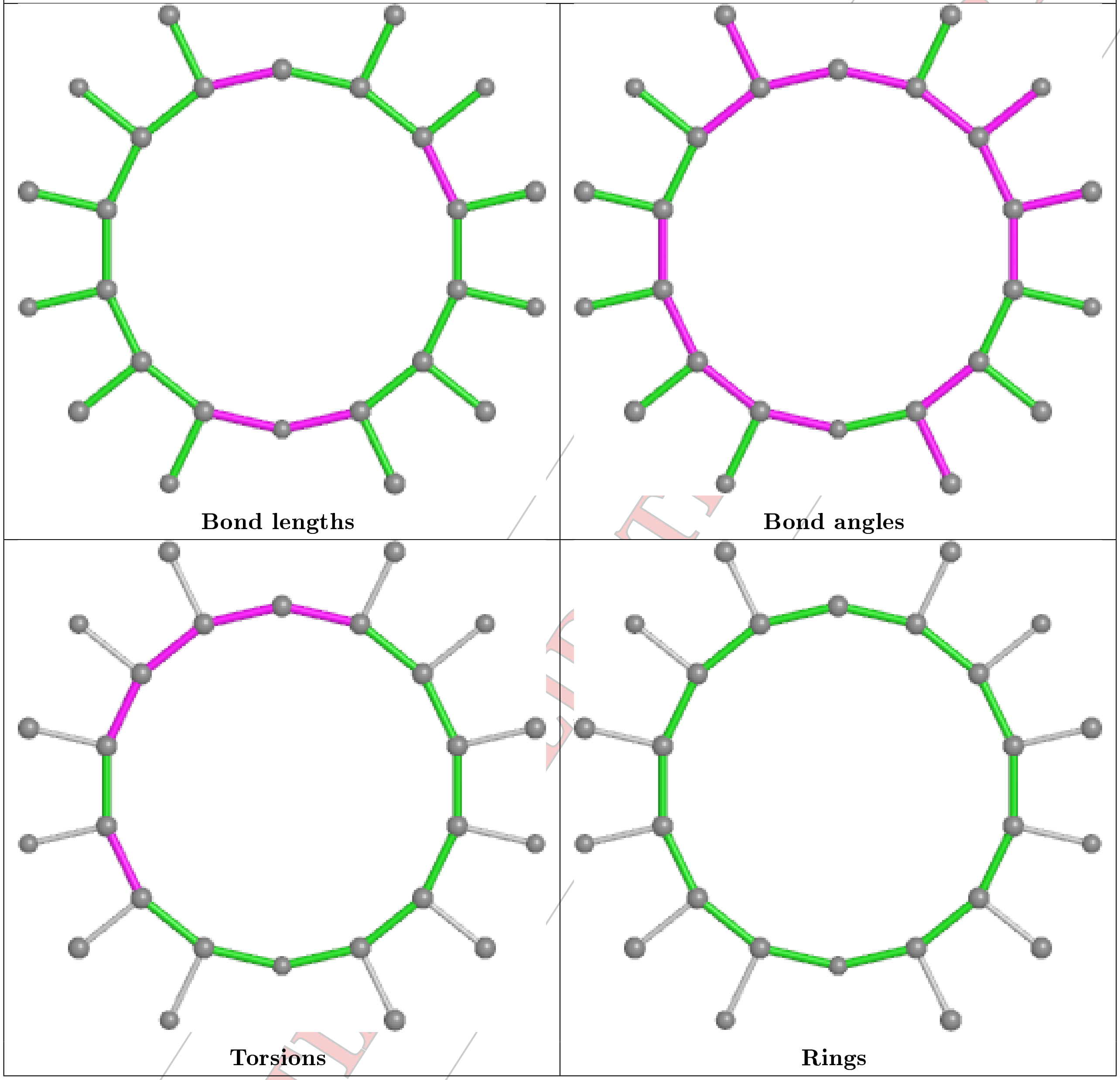


CONFIDENTIAL DRAFT

**Ligand HEM D 501**

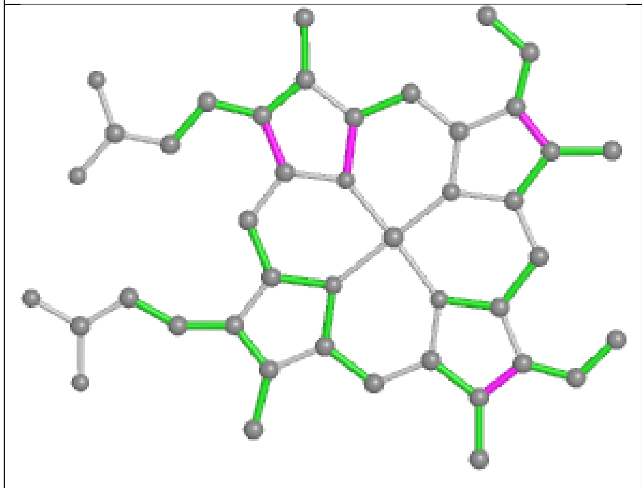
Bond lengths

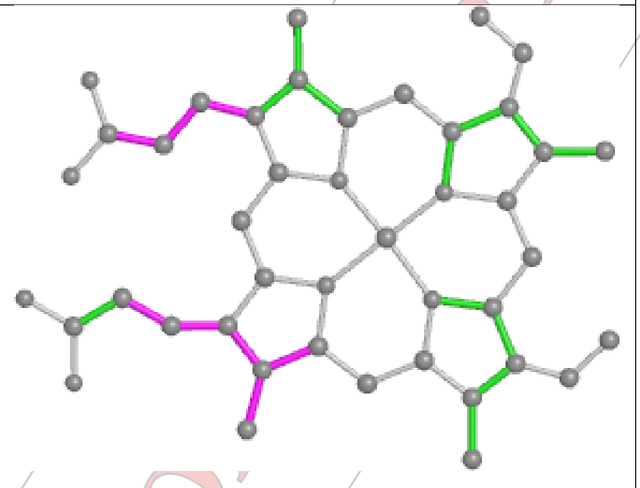
Bond angles

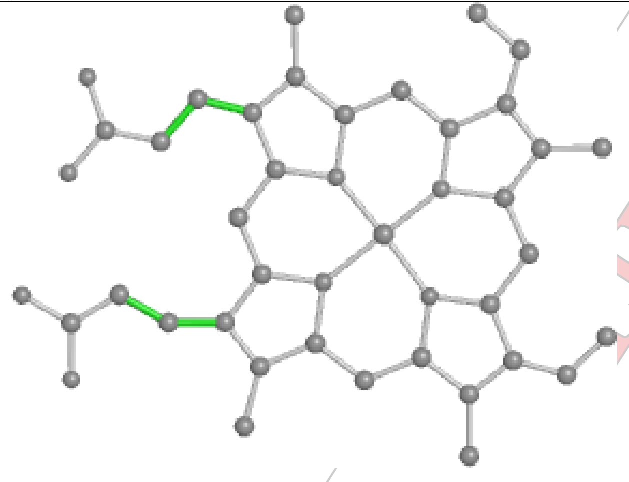
Torsions

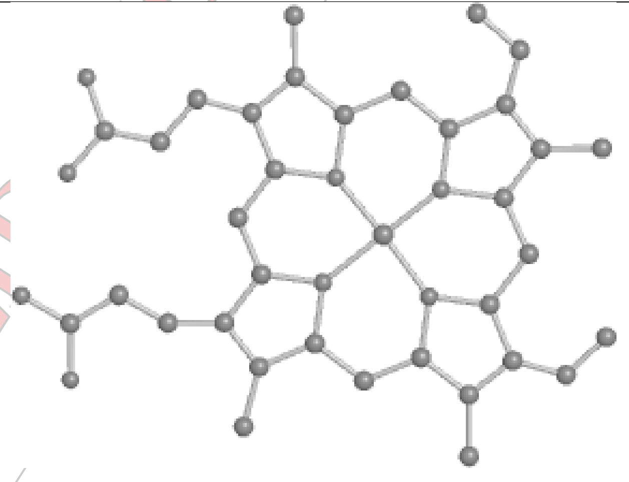
Rings

CONFIDENTIAL

**Ligand QR8 E 503**

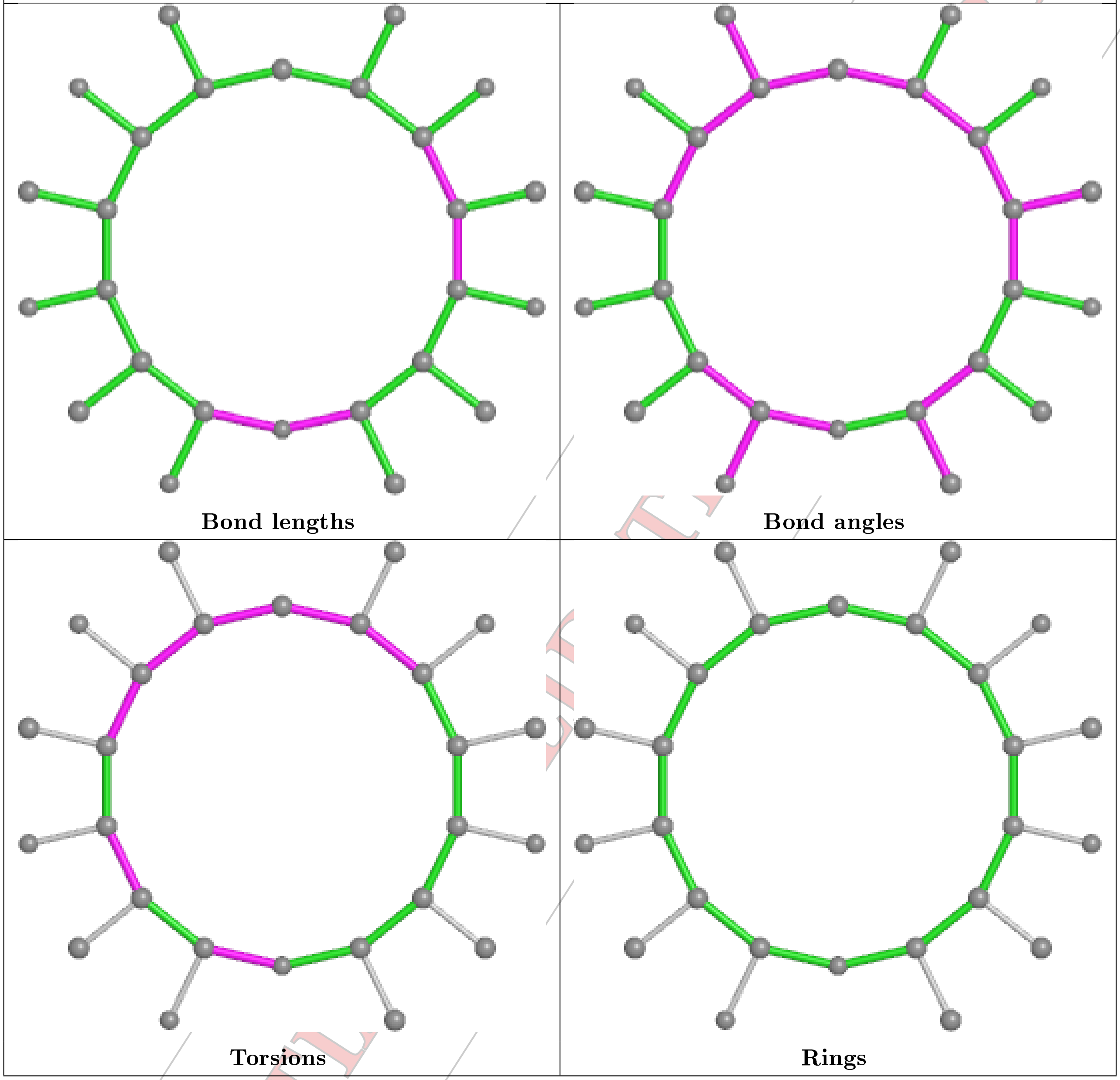

Bond lengths

Bond angles

Torsions

Rings

CONFIDENTIAL DRAFT

**Ligand HEM C 501**

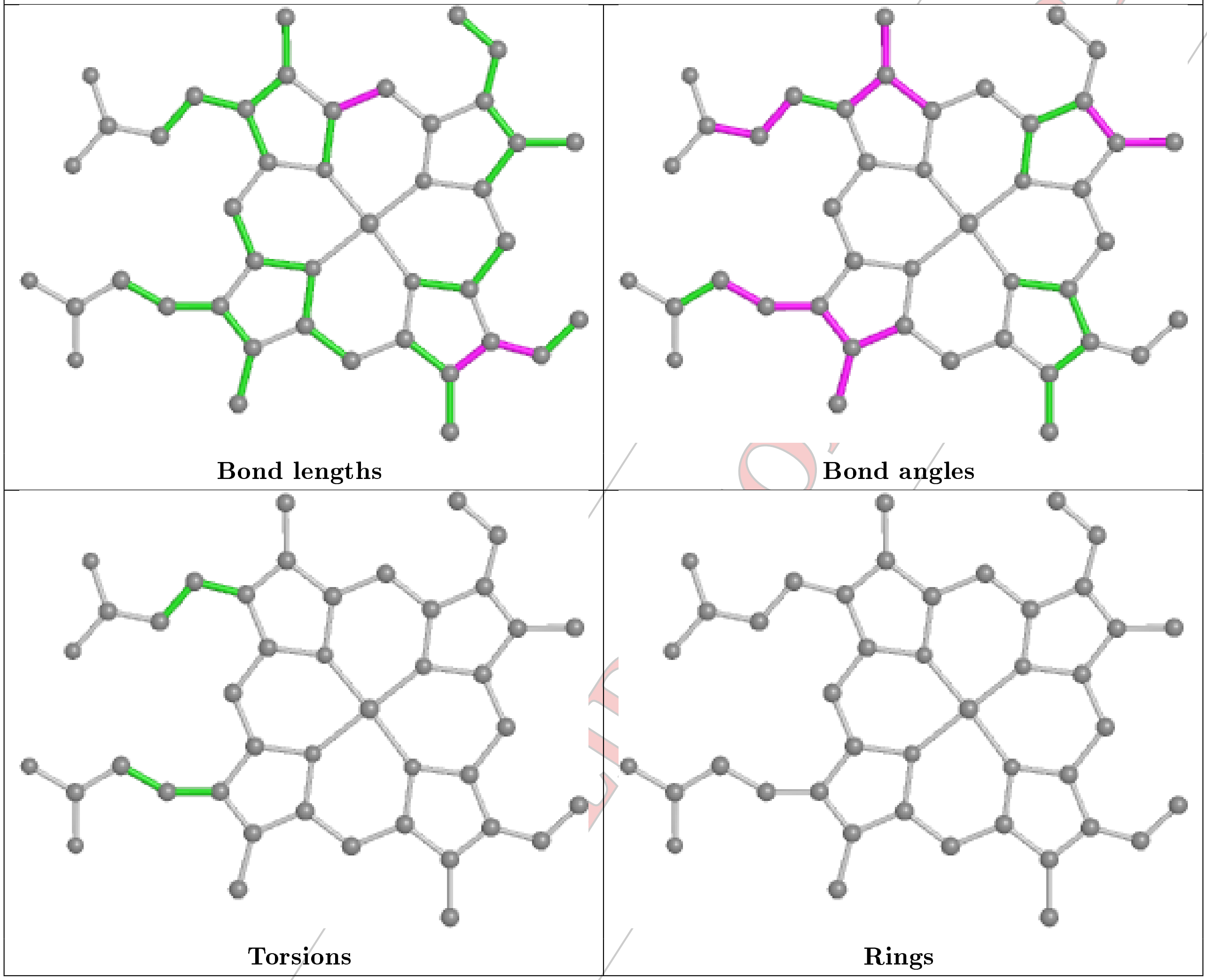

Bond lengths

Bond angles

Torsions

Rings

CONFIDENTIAL

**Ligand HEM E 502**

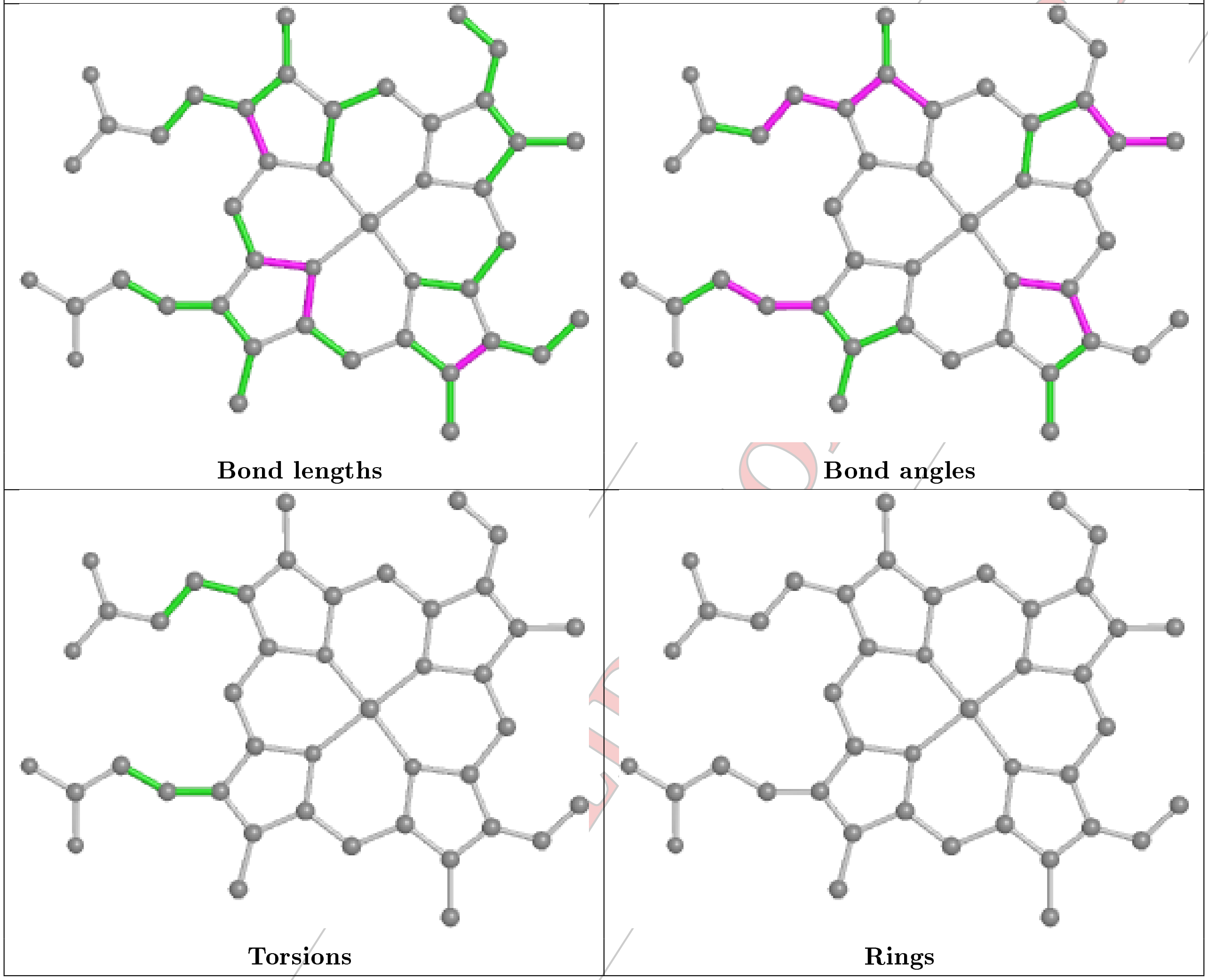


CONFIDENTIAL

**Ligand QR8 A 502**

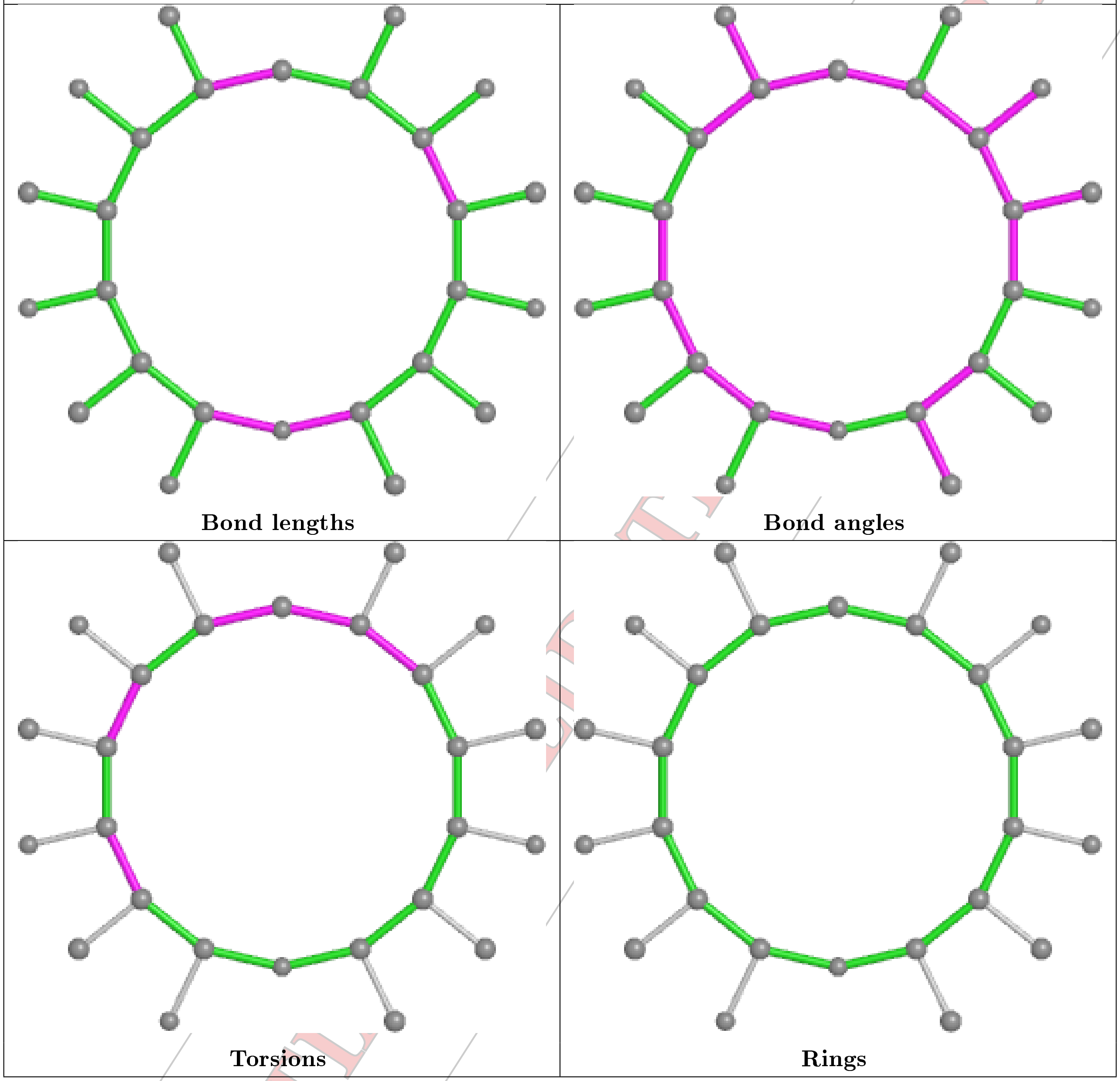


CONFIDENTIAL REPORT

**Ligand HEM B 501**

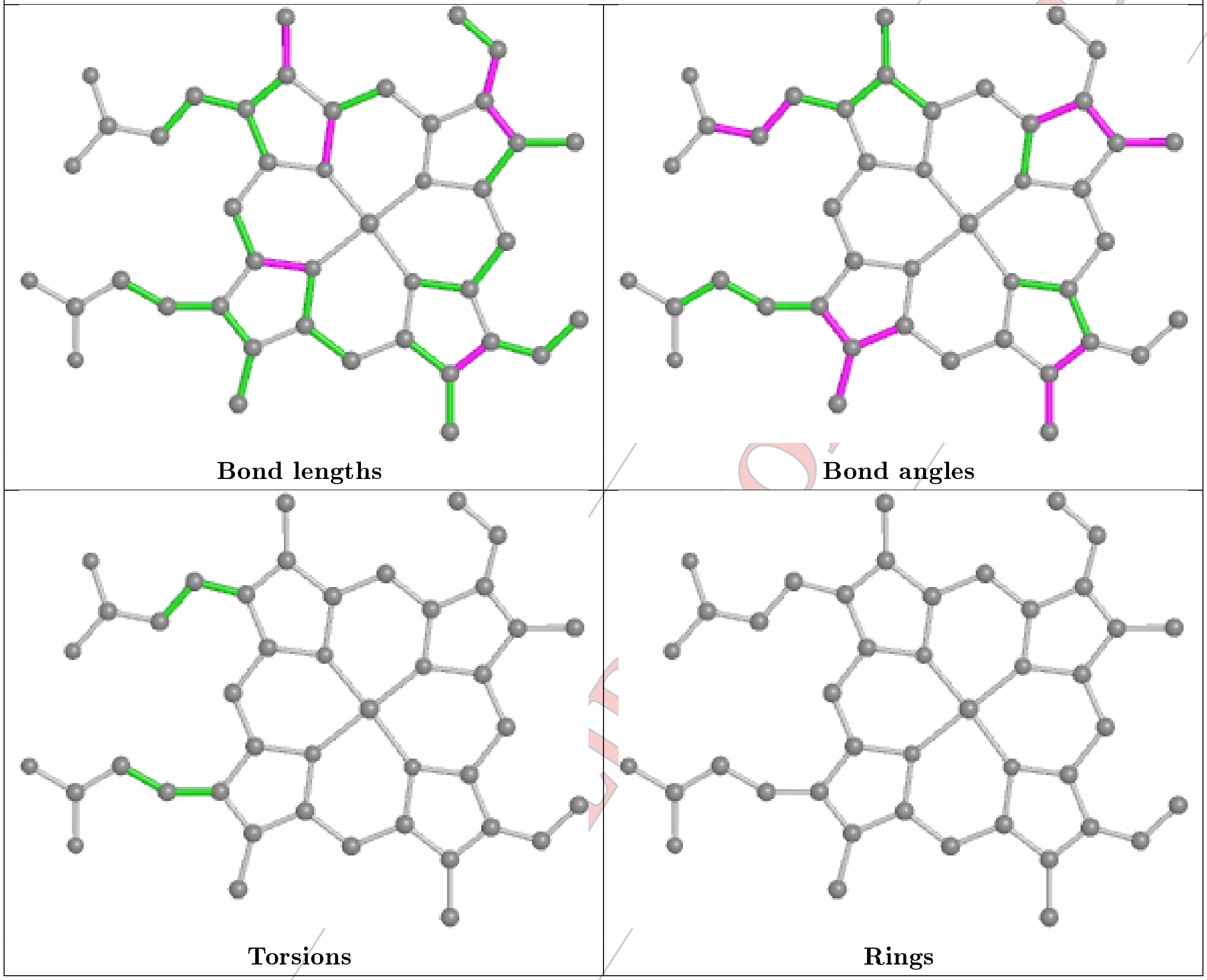


CONFIDENTIAL

**Ligand QR8 C 502**

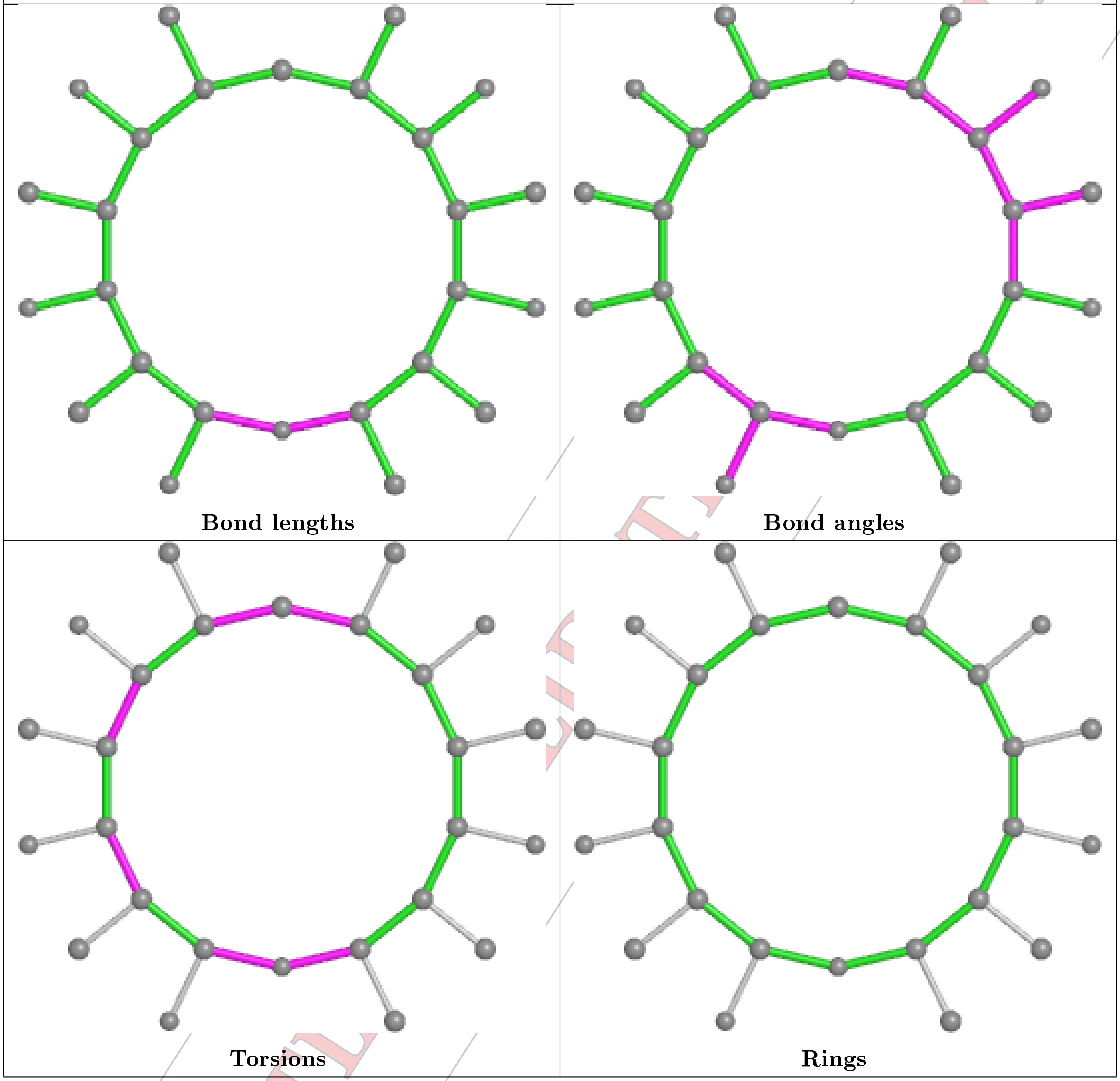


CONFIDENTIAL

Ligand HEM F 502

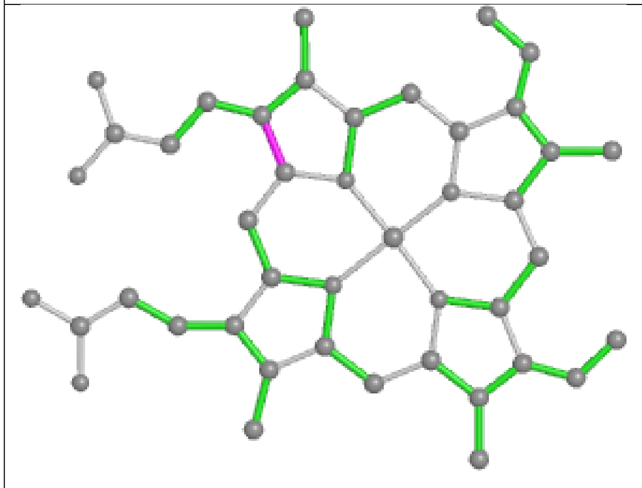
Bond lengths

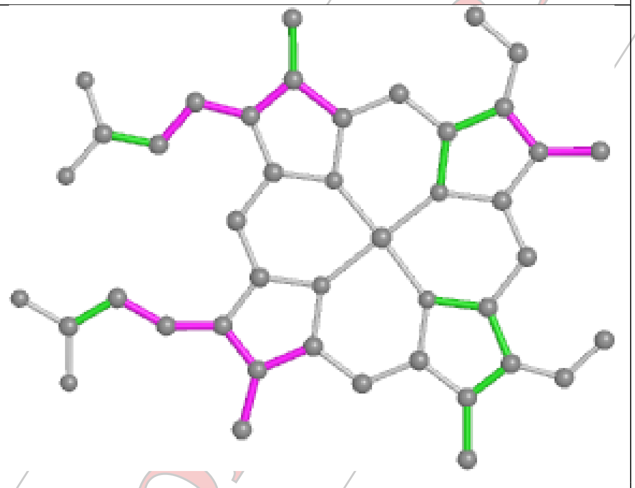
Bond angles

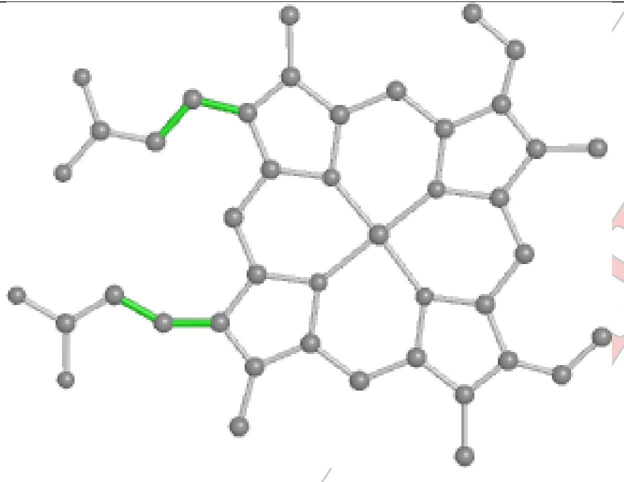
Torsions

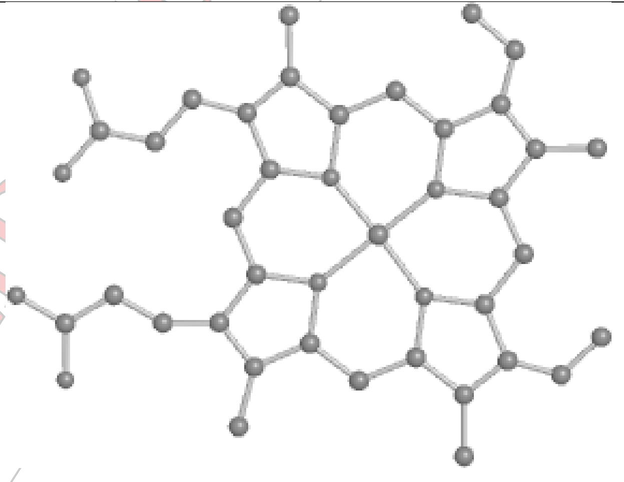
Rings

CONFIDENTIAL

**Ligand HEM A 501**

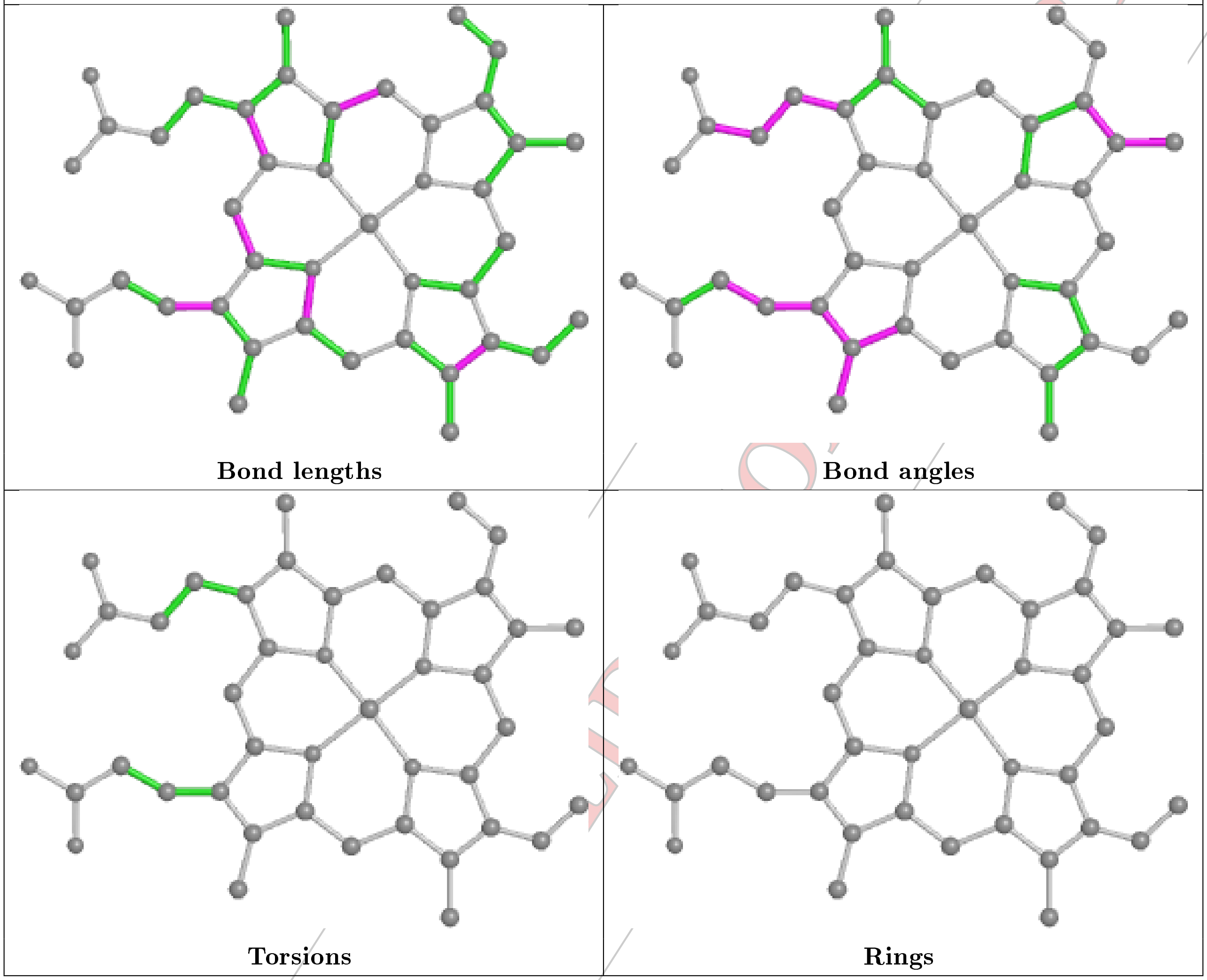


## 5.7 Other polymers (i)

There are no such residues in this entry.

## 5.8 Polymer linkage issues (i)

There are no chain breaks in this entry.

CONFIDENTIAL

# 6 Fit of model and data (i)

## 6.1 Protein, DNA and RNA chains (i)

In the following table, the column labelled '#RSRZ> 2' contains the number (and percentage) of RSRZ outliers, followed by percent RSRZ outliers for the chain as percentile scores relative to all X-ray entries and entries of similar resolution. The OWAB column contains the minimum, median, $95^{th}$ percentile and maximum values of the occupancy-weighted average B-factor per residue. The column labelled 'Q< 0.9' lists the number of (and percentage) of residues with an average occupancy less than 0.9.

| Mol | Chain | Analysed | <RSRZ> | #RSRZ>2 | | | OWAB(Å$^2$) | Q<0.9 |
|---|---|---|---|---|---|---|---|---|
| 1 | A | 396/407 (97%) | -0.26 | 5 (1%) | 77 | 81 | 31, 48, 71, 103 | 0 |
| 1 | B | 400/407 (98%) | -0.29 | 6 (1%) | 73 | 78 | 34, 49, 74, 154 | 0 |
| 1 | C | 400/407 (98%) | -0.27 | 4 (1%) | 82 | 86 | 29, 41, 58, 154 | 0 |
| 1 | D | 400/407 (98%) | 0.07 | 24 (6%) | 21 | 26 | 39, 60, 92, 170 | 0 |
| 1 | E | 397/407 (97%) | 0.35 | 47 (11%) | 4 | 6 | 43, 63, 94, 147 | 0 |
| 1 | F | 398/407 (97%) | 0.83 | 80 (20%) | 1 | 1 | 47, 72, 115, 146 | 0 |
| All | All | 2391/2442 (97%) | 0.07 | 166 (6%) | 16 | 21 | 29, 55, 97, 170 | 0 |

All (166) RSRZ outliers are listed below:

| Mol | Chain | Res | Type | RSRZ |
|---|---|---|---|---|
| 1 | D | 9 | THR | 10.0 |
| 1 | E | 226 | ASP | 8.8 |
| 1 | F | 405 | VAL | 8.7 |
| 1 | F | 127[A] | LEU | 8.7 |
| 1 | D | 11 | ALA | 8.6 |
| 1 | D | 10 | PRO | 7.6 |
| 1 | E | 313 | ALA | 7.6 |
| 1 | F | 377[A] | PHE | 7.5 |
| 1 | F | 380[A] | LEU | 7.4 |
| 1 | F | 129[A] | ASP | 7.4 |
| 1 | B | 9 | THR | 7.1 |
| 1 | F | 124 | VAL | 6.7 |
| 1 | B | 209[A] | ASP | 6.4 |
| 1 | F | 132[A] | LEU | 6.2 |
| 1 | F | 125 | ARG | 5.9 |
| 1 | F | 262 | THR | 5.9 |
| 1 | F | 141 | PRO | 5.8 |
| 1 | F | 123 | ARG | 5.7 |
| 1 | F | 23[A] | HIS | 5.4 |

*Continued on next page...*

CONFIDENTIAL VALIDATION REPORT

*Continued from previous page...*

| Mol | Chain | Res | Type | RSRZ |
|---|---|---|---|---|
| 1 | F | 379[A] | THR | 5.4 |
| 1 | F | 271 | VAL | 5.2 |
| 1 | E | 312[A] | ARG | 5.1 |
| 1 | D | 208[A] | ARG | 5.0 |
| 1 | F | 273 | ASP | 5.0 |
| 1 | D | 226 | ASP | 4.8 |
| 1 | F | 404 | ILE | 4.8 |
| 1 | F | 36 | ARG | 4.8 |
| 1 | F | 277 | VAL | 4.8 |
| 1 | D | 225 | ASN | 4.8 |
| 1 | F | 382[A] | LEU | 4.7 |
| 1 | C | 9 | THR | 4.7 |
| 1 | D | 224 | ASP | 4.7 |
| 1 | F | 270 | LEU | 4.7 |
| 1 | D | 210 | ALA | 4.6 |
| 1 | F | 265 | LYS | 4.6 |
| 1 | F | 385 | PRO | 4.6 |
| 1 | F | 142 | ALA | 4.5 |
| 1 | E | 304 | VAL | 4.5 |
| 1 | F | 332 | ASP | 4.5 |
| 1 | E | 209[A] | ASP | 4.4 |
| 1 | E | 311[A] | VAL | 4.4 |
| 1 | E | 11 | ALA | 4.4 |
| 1 | D | 209[A] | ASP | 4.3 |
| 1 | F | 126 | SER | 4.3 |
| 1 | D | 205 | ALA | 4.3 |
| 1 | E | 305[A] | GLU | 4.2 |
| 1 | F | 267 | TYR | 4.1 |
| 1 | F | 388 | GLY | 4.1 |
| 1 | D | 223 | THR | 4.1 |
| 1 | F | 407 | TRP | 4.0 |
| 1 | F | 406 | SER | 4.0 |
| 1 | F | 364 | LEU | 4.0 |
| 1 | E | 307 | SER | 4.0 |
| 1 | E | 212 | THR | 4.0 |
| 1 | E | 306 | LEU | 3.9 |
| 1 | E | 213 | GLU | 3.9 |
| 1 | E | 13 | ALA | 3.9 |
| 1 | D | 228[A] | HIS | 3.9 |
| 1 | F | 140 | SER | 3.8 |
| 1 | F | 264 | ARG | 3.8 |
| 1 | E | 265[A] | LYS | 3.8 |

*Continued on next page...*

CONFIDENTIAL VALIDATION REPORT

*Continued from previous page...*

| Mol | Chain | Res | Type | RSRZ |
|---|---|---|---|---|
| 1 | D | 13 | ALA | 3.8 |
| 1 | F | 24 | ALA | 3.7 |
| 1 | F | 131[A] | LEU | 3.7 |
| 1 | E | 12 | ASP | 3.7 |
| 1 | B | 210 | ALA | 3.7 |
| 1 | A | 209[A] | ASP | 3.7 |
| 1 | F | 272 | ALA | 3.6 |
| 1 | E | 333 | HIS | 3.6 |
| 1 | E | 310[A] | THR | 3.6 |
| 1 | E | 301 | THR | 3.5 |
| 1 | F | 376[A] | ARG | 3.4 |
| 1 | D | 191[A] | ARG | 3.4 |
| 1 | F | 386 | VAL | 3.4 |
| 1 | F | 375[A] | ARG | 3.3 |
| 1 | D | 222 | ALA | 3.3 |
| 1 | E | 14[A] | VAL | 3.3 |
| 1 | F | 328 | GLU | 3.3 |
| 1 | E | 332 | ASP | 3.3 |
| 1 | E | 36[A] | ARG | 3.2 |
| 1 | E | 39 | PRO | 3.2 |
| 1 | F | 215 | LEU | 3.2 |
| 1 | F | 225 | ASN | 3.2 |
| 1 | E | 37 | ASP | 3.2 |
| 1 | F | 120[A] | MET | 3.2 |
| 1 | F | 130[A] | SER | 3.1 |
| 1 | E | 309 | VAL | 3.1 |
| 1 | D | 263[A] | GLU | 3.1 |
| 1 | F | 261 | LEU | 3.1 |
| 1 | F | 139 | GLY | 3.1 |
| 1 | F | 343 | ARG | 3.1 |
| 1 | F | 334 | ALA | 3.0 |
| 1 | F | 371 | SER | 3.0 |
| 1 | F | 383 | ALA | 3.0 |
| 1 | E | 314 | GLY | 3.0 |
| 1 | F | 329 | GLU | 2.9 |
| 1 | E | 34[A] | LEU | 2.9 |
| 1 | E | 42 | ARG | 2.9 |
| 1 | F | 224[A] | ASP | 2.9 |
| 1 | F | 330 | VAL | 2.8 |
| 1 | D | 141 | PRO | 2.8 |
| 1 | F | 378 | PRO | 2.8 |
| 1 | A | 225[A] | ASN | 2.8 |

*Continued on next page...*

CONFIDENTIAL VALIDATION REPORT

*Continued from previous page...*

| Mol | Chain | Res | Type | RSRZ |
|---|---|---|---|---|
| 1 | E | 66 | GLY | 2.8 |
| 1 | E | 210 | ALA | 2.8 |
| 1 | E | 272 | ALA | 2.8 |
| 1 | E | 29 | PRO | 2.8 |
| 1 | F | 21 | LEU | 2.8 |
| 1 | F | 333 | HIS | 2.8 |
| 1 | F | 340 | HIS | 2.8 |
| 1 | B | 265[A] | LYS | 2.8 |
| 1 | E | 69 | ARG | 2.8 |
| 1 | F | 134 | ASP | 2.7 |
| 1 | F | 402[A] | ARG | 2.8 |
| 1 | A | 224[A] | ASP | 2.7 |
| 1 | F | 331 | PHE | 2.7 |
| 1 | E | 303 | ASP | 2.7 |
| 1 | F | 29 | PRO | 2.7 |
| 1 | D | 8 | PRO | 2.6 |
| 1 | E | 388 | GLY | 2.6 |
| 1 | F | 37 | ASP | 2.6 |
| 1 | E | 223[A] | THR | 2.6 |
| 1 | F | 269 | SER | 2.6 |
| 1 | E | 30 | HIS | 2.6 |
| 1 | A | 379 | THR | 2.6 |
| 1 | D | 227[A] | ASP | 2.5 |
| 1 | F | 57[A] | ARG | 2.5 |
| 1 | F | 274 | PRO | 2.5 |
| 1 | F | 372 | ALA | 2.5 |
| 1 | E | 60 | ASP | 2.5 |
| 1 | F | 138 | HIS | 2.4 |
| 1 | F | 32 | ALA | 2.4 |
| 1 | B | 333[A] | HIS | 2.4 |
| 1 | F | 268 | GLU | 2.4 |
| 1 | F | 326[A] | ARG | 2.4 |
| 1 | F | 136[A] | VAL | 2.4 |
| 1 | F | 263 | GLU | 2.3 |
| 1 | F | 137 | ALA | 2.3 |
| 1 | F | 128[A] | VAL | 2.3 |
| 1 | F | 122 | PRO | 2.3 |
| 1 | C | 10 | PRO | 2.3 |
| 1 | F | 327 | ASP | 2.3 |
| 1 | E | 329[A] | GLU | 2.2 |
| 1 | E | 336[A] | GLU | 2.2 |
| 1 | E | 51 | THR | 2.2 |

*Continued on next page...*

CONFIDENTIAL VALIDATION REPORT

*Continued from previous page...*

| Mol | Chain | Res | Type | RSRZ |
|---|---|---|---|---|
| 1 | E | 343[A] | ARG | 2.2 |
| 1 | E | 269 | SER | 2.2 |
| 1 | F | 10 | PRO | 2.2 |
| 1 | D | 335[A] | ASP | 2.2 |
| 1 | F | 35 | ARG | 2.1 |
| 1 | E | 211 | PRO | 2.1 |
| 1 | C | 11 | ALA | 2.1 |
| 1 | D | 385 | PRO | 2.1 |
| 1 | D | 406 | SER | 2.1 |
| 1 | E | 268 | GLU | 2.1 |
| 1 | F | 30 | HIS | 2.1 |
| 1 | E | 271 | VAL | 2.1 |
| 1 | C | 343[A] | ARG | 2.1 |
| 1 | B | 211 | PRO | 2.1 |
| 1 | F | 33[A] | GLU | 2.0 |
| 1 | E | 40 | VAL | 2.0 |
| 1 | E | 299 | VAL | 2.0 |
| 1 | D | 334 | ALA | 2.0 |
| 1 | D | 332[A] | ASP | 2.0 |
| 1 | F | 337[A] | LEU | 2.0 |
| 1 | A | 343 | ARG | 2.0 |

## 6.2 Non-standard residues in protein, DNA, RNA chains (i)

There are no non-standard protein/DNA/RNA residues in this entry.

## 6.3 Carbohydrates (i)

There are no monosaccharides in this entry.

## 6.4 Ligands (i)

In the following table, the Atoms column lists the number of modelled atoms in the group and the number defined in the chemical component dictionary. The B-factors column lists the minimum, median, $95^{th}$ percentile and maximum values of B factors of atoms in the group. The column labelled 'Q< 0.9' lists the number of atoms with occupancy less than 0.9.

| Mol | Type | Chain | Res | Atoms | RSCC | RSR | B-factors(Å$^2$) | Q<0.9 |
|---|---|---|---|---|---|---|---|---|
| 5 | FMT | D | 530 | 3/3 | -0.18 | 0.47 | 101,101,109,109 | 0 |
| 5 | FMT | E | 511 | 3/3 | 0.03 | 0.71 | 119,119,120,122 | 0 |

*Continued on next page...*

CONFIDENTIAL VALIDATION REPORT

*Continued from previous page...*

| Mol | Type | Chain | Res | Atoms | RSCC | RSR | B-factors(Å$^2$) | Q<0.9 |
|---|---|---|---|---|---|---|---|---|
| 5 | FMT | A | 529 | 3/3 | 0.09 | 0.43 | 100,100,107,109 | 0 |
| 5 | FMT | E | 515 | 3/3 | 0.11 | 0.39 | 103,103,114,115 | 0 |
| 5 | FMT | F | 512 | 3/3 | 0.16 | 0.30 | 106,106,112,113 | 0 |
| 5 | FMT | A | 551 | 3/3 | 0.17 | 0.38 | 104,104,113,115 | 0 |
| 5 | FMT | B | 540 | 3/3 | 0.18 | 0.32 | 95,95,99,100 | 0 |
| 5 | FMT | B | 517 | 3/3 | 0.23 | 0.35 | 86,86,94,100 | 0 |
| 5 | FMT | E | 509 | 3/3 | 0.25 | 0.45 | 86,86,101,104 | 0 |
| 5 | FMT | B | 533 | 3/3 | 0.25 | 0.35 | 78,78,79,89 | 0 |
| 5 | FMT | A | 554 | 3/3 | 0.27 | 0.32 | 100,100,107,111 | 0 |
| 5 | FMT | D | 526 | 3/3 | 0.28 | 0.23 | 104,104,106,112 | 0 |
| 5 | FMT | C | 530 | 3/3 | 0.30 | 0.31 | 105,105,110,113 | 0 |
| 5 | FMT | C | 539 | 3/3 | 0.31 | 0.45 | 87,87,90,98 | 0 |
| 5 | FMT | D | 517 | 3/3 | 0.35 | 0.56 | 114,114,121,129 | 0 |
| 5 | FMT | A | 533 | 3/3 | 0.35 | 0.21 | 87,87,91,92 | 0 |
| 5 | FMT | D | 524 | 3/3 | 0.36 | 0.25 | 111,111,112,116 | 0 |
| 5 | FMT | B | 527 | 3/3 | 0.37 | 0.16 | 96,96,99,103 | 0 |
| 5 | FMT | C | 549 | 3/3 | 0.38 | 0.20 | 100,100,100,101 | 0 |
| 5 | FMT | F | 511 | 3/3 | 0.39 | 0.39 | 101,101,109,109 | 0 |
| 5 | FMT | C | 531 | 3/3 | 0.39 | 0.37 | 102,102,102,106 | 0 |
| 5 | FMT | C | 551 | 3/3 | 0.42 | 0.44 | 90,90,94,103 | 0 |
| 5 | FMT | C | 541 | 3/3 | 0.45 | 0.33 | 83,83,85,91 | 0 |
| 5 | FMT | A | 563 | 3/3 | 0.50 | 0.44 | 97,97,102,107 | 0 |
| 5 | FMT | A | 564 | 3/3 | 0.50 | 0.28 | 90,90,102,105 | 0 |
| 5 | FMT | D | 521 | 3/3 | 0.51 | 0.21 | 102,102,102,104 | 0 |
| 5 | FMT | E | 510 | 3/3 | 0.51 | 0.26 | 95,95,107,108 | 0 |
| 5 | FMT | B | 520 | 3/3 | 0.51 | 0.21 | 95,95,102,102 | 0 |
| 5 | FMT | A | 545 | 3/3 | 0.52 | 0.32 | 83,83,87,101 | 0 |
| 5 | FMT | C | 528 | 3/3 | 0.53 | 0.44 | 95,95,95,101 | 0 |
| 5 | FMT | B | 526 | 3/3 | 0.54 | 0.20 | 96,96,96,99 | 0 |
| 5 | FMT | C | 515 | 3/3 | 0.55 | 0.19 | 63,63,76,76 | 0 |
| 5 | FMT | A | 560 | 3/3 | 0.55 | 0.23 | 74,74,81,93 | 0 |
| 5 | FMT | C | 511 | 3/3 | 0.56 | 0.22 | 68,68,76,80 | 0 |
| 5 | FMT | D | 527 | 3/3 | 0.56 | 0.43 | 91,91,97,97 | 0 |
| 5 | FMT | A | 517 | 3/3 | 0.56 | 0.49 | 98,98,106,108 | 0 |
| 5 | FMT | C | 526 | 3/3 | 0.57 | 0.20 | 75,75,80,83 | 0 |
| 5 | FMT | A | 574 | 3/3 | 0.58 | 0.43 | 102,102,108,111 | 0 |
| 5 | FMT | A | 537 | 3/3 | 0.59 | 0.35 | 100,100,109,109 | 0 |
| 5 | FMT | D | 519 | 3/3 | 0.59 | 0.38 | 90,90,91,96 | 0 |
| 5 | FMT | B | 511 | 3/3 | 0.60 | 0.20 | 73,73,77,78 | 0 |
| 5 | FMT | A | 552 | 3/3 | 0.60 | 0.25 | 83,83,89,92 | 0 |
| 5 | FMT | D | 525 | 3/3 | 0.60 | 0.32 | 87,87,91,92 | 0 |
| 5 | FMT | F | 510 | 3/3 | 0.61 | 0.31 | 88,88,98,100 | 0 |

*Continued on next page...*

CONFIDENTIAL VALIDATION REPORT

*Continued from previous page...*

| Mol | Type | Chain | Res | Atoms | RSCC | RSR | B-factors(Å$^2$) | Q<0.9 |
|---|---|---|---|---|---|---|---|---|
| 5 | FMT | C | 540 | 3/3 | 0.61 | 0.31 | 74,74,80,83 | 0 |
| 5 | FMT | A | 542 | 3/3 | 0.62 | 0.21 | 102,102,103,104 | 0 |
| 4 | RAM | A | 503[B] | 11/11 | 0.63 | 0.49 | 37,41,44,46 | 11 |
| 5 | FMT | A | 507 | 3/3 | 0.63 | 0.18 | 69,69,73,76 | 0 |
| 4 | RAM | A | 503[A] | 11/11 | 0.63 | 0.49 | 34,37,39,44 | 11 |
| 5 | FMT | A | 515 | 3/3 | 0.63 | 0.23 | 80,80,82,89 | 0 |
| 5 | FMT | E | 508 | 3/3 | 0.63 | 0.29 | 86,86,89,93 | 0 |
| 5 | FMT | C | 544 | 3/3 | 0.64 | 0.49 | 68,68,73,82 | 0 |
| 5 | FMT | E | 517 | 3/3 | 0.65 | 0.50 | 95,95,99,100 | 0 |
| 5 | FMT | B | 528 | 3/3 | 0.65 | 0.15 | 85,85,86,89 | 0 |
| 5 | FMT | C | 525 | 3/3 | 0.65 | 0.26 | 90,90,91,94 | 0 |
| 5 | FMT | C | 543 | 3/3 | 0.66 | 0.47 | 82,82,87,92 | 0 |
| 5 | FMT | A | 539 | 3/3 | 0.66 | 0.22 | 101,101,110,111 | 0 |
| 5 | FMT | B | 523 | 3/3 | 0.67 | 0.30 | 86,86,87,92 | 0 |
| 5 | FMT | B | 531 | 3/3 | 0.68 | 0.17 | 98,98,102,104 | 0 |
| 5 | FMT | A | 571 | 3/3 | 0.68 | 0.25 | 94,94,108,108 | 0 |
| 4 | RAM | B | 513 | 11/11 | 0.68 | 0.32 | 50,57,63,64 | 11 |
| 5 | FMT | A | 559 | 3/3 | 0.69 | 0.22 | 90,90,91,95 | 0 |
| 5 | FMT | B | 504 | 3/3 | 0.69 | 0.34 | 69,69,74,80 | 0 |
| 5 | FMT | A | 527 | 3/3 | 0.69 | 0.23 | 88,88,93,100 | 0 |
| 5 | FMT | C | 535 | 3/3 | 0.69 | 0.31 | 89,89,112,116 | 0 |
| 5 | FMT | B | 519 | 3/3 | 0.69 | 0.36 | 100,100,101,104 | 0 |
| 5 | FMT | C | 548 | 3/3 | 0.69 | 0.21 | 85,85,91,91 | 0 |
| 5 | FMT | A | 508 | 3/3 | 0.69 | 0.31 | 74,74,76,83 | 0 |
| 5 | FMT | C | 537 | 3/3 | 0.69 | 0.38 | 97,97,109,111 | 0 |
| 5 | FMT | C | 517 | 3/3 | 0.70 | 0.25 | 70,70,71,74 | 0 |
| 5 | FMT | B | 525 | 3/3 | 0.70 | 0.25 | 98,98,105,105 | 0 |
| 5 | FMT | B | 516 | 3/3 | 0.70 | 0.13 | 100,100,103,107 | 0 |
| 5 | FMT | C | 504 | 3/3 | 0.70 | 0.15 | 48,48,52,54 | 0 |
| 5 | FMT | D | 512 | 3/3 | 0.71 | 0.20 | 85,85,88,92 | 0 |
| 5 | FMT | A | 568 | 3/3 | 0.71 | 0.19 | 85,85,90,91 | 0 |
| 5 | FMT | A | 540 | 3/3 | 0.72 | 0.40 | 105,105,112,114 | 0 |
| 5 | FMT | B | 521 | 3/3 | 0.72 | 0.29 | 66,66,78,84 | 0 |
| 5 | FMT | C | 523 | 3/3 | 0.72 | 0.26 | 69,69,75,81 | 0 |
| 5 | FMT | C | 547 | 3/3 | 0.72 | 0.36 | 65,65,73,82 | 0 |
| 5 | FMT | D | 507 | 3/3 | 0.72 | 0.35 | 85,85,90,91 | 0 |
| 5 | FMT | B | 538 | 3/3 | 0.72 | 0.45 | 82,82,83,86 | 0 |
| 5 | FMT | A | 556 | 3/3 | 0.72 | 0.30 | 87,87,89,93 | 0 |
| 5 | FMT | F | 504 | 3/3 | 0.73 | 0.17 | 78,78,87,91 | 0 |
| 5 | FMT | B | 522 | 3/3 | 0.73 | 0.57 | 76,76,82,90 | 0 |
| 5 | FMT | C | 532 | 3/3 | 0.73 | 0.14 | 49,49,57,72 | 0 |
| 5 | FMT | A | 566 | 3/3 | 0.73 | 0.14 | 87,87,89,96 | 0 |

*Continued on next page...*

CONFIDENTIAL VALIDATION REPORT

*Continued from previous page...*

| Mol | Type | Chain | Res | Atoms | RSCC | RSR | B-factors(Å$^2$) | Q<0.9 |
|---|---|---|---|---|---|---|---|---|
| 5 | FMT | A | 531 | 3/3 | 0.74 | 0.21 | 63,63,65,75 | 0 |
| 5 | FMT | A | 550 | 3/3 | 0.74 | 0.31 | 64,64,78,80 | 0 |
| 5 | FMT | D | 514 | 3/3 | 0.74 | 0.24 | 60,60,60,71 | 0 |
| 5 | FMT | E | 516 | 3/3 | 0.74 | 0.14 | 90,90,90,96 | 0 |
| 5 | FMT | A | 561 | 3/3 | 0.74 | 0.16 | 77,77,83,87 | 0 |
| 5 | FMT | D | 515 | 3/3 | 0.74 | 0.29 | 79,79,80,88 | 0 |
| 5 | FMT | C | 508 | 3/3 | 0.75 | 0.23 | 81,81,87,90 | 0 |
| 5 | FMT | B | 518 | 3/3 | 0.75 | 0.19 | 89,89,104,105 | 0 |
| 5 | FMT | C | 545 | 3/3 | 0.75 | 0.50 | 78,78,80,86 | 0 |
| 5 | FMT | F | 509 | 3/3 | 0.75 | 0.17 | 66,66,67,74 | 0 |
| 5 | FMT | A | 565 | 3/3 | 0.75 | 0.19 | 66,66,82,85 | 0 |
| 5 | FMT | A | 547 | 3/3 | 0.75 | 0.19 | 70,70,73,77 | 0 |
| 5 | FMT | A | 546 | 3/3 | 0.76 | 0.43 | 64,64,73,83 | 0 |
| 5 | FMT | B | 514 | 3/3 | 0.76 | 0.22 | 75,75,80,87 | 0 |
| 5 | FMT | A | 558 | 3/3 | 0.76 | 0.22 | 83,83,84,92 | 0 |
| 5 | FMT | D | 535 | 3/3 | 0.76 | 0.33 | 96,96,104,104 | 0 |
| 5 | FMT | A | 544 | 3/3 | 0.76 | 0.23 | 67,67,77,81 | 0 |
| 5 | FMT | A | 528 | 3/3 | 0.77 | 0.21 | 84,84,84,93 | 0 |
| 5 | FMT | A | 509 | 3/3 | 0.77 | 0.15 | 61,61,64,71 | 0 |
| 5 | FMT | D | 536 | 3/3 | 0.78 | 0.51 | 111,111,113,115 | 0 |
| 5 | FMT | C | 536 | 3/3 | 0.78 | 0.31 | 98,98,99,104 | 0 |
| 5 | FMT | B | 524 | 3/3 | 0.78 | 0.32 | 68,68,74,78 | 0 |
| 5 | FMT | D | 531 | 3/3 | 0.78 | 0.39 | 59,59,65,71 | 0 |
| 5 | FMT | A | 523 | 3/3 | 0.78 | 0.19 | 67,67,70,77 | 0 |
| 5 | FMT | D | 518 | 3/3 | 0.78 | 0.15 | 84,84,96,100 | 0 |
| 5 | FMT | D | 506 | 3/3 | 0.78 | 0.26 | 68,68,81,84 | 0 |
| 5 | FMT | B | 539 | 3/3 | 0.78 | 0.42 | 85,85,89,91 | 0 |
| 5 | FMT | B | 532 | 3/3 | 0.79 | 0.18 | 96,96,99,102 | 0 |
| 5 | FMT | A | 520 | 3/3 | 0.79 | 0.21 | 84,84,87,90 | 0 |
| 4 | RAM | D | 503 | 11/11 | 0.80 | 0.38 | 58,66,68,68 | 11 |
| 5 | FMT | B | 537 | 3/3 | 0.80 | 0.12 | 82,82,94,96 | 0 |
| 5 | FMT | A | 549 | 3/3 | 0.80 | 0.49 | 71,71,87,90 | 0 |
| 5 | FMT | D | 522 | 3/3 | 0.80 | 0.14 | 97,97,106,113 | 0 |
| 5 | FMT | C | 509 | 3/3 | 0.80 | 0.18 | 60,60,67,68 | 0 |
| 5 | FMT | F | 515 | 3/3 | 0.81 | 0.13 | 101,101,103,108 | 0 |
| 5 | FMT | E | 514 | 3/3 | 0.81 | 0.38 | 86,86,94,97 | 0 |
| 5 | FMT | A | 548 | 3/3 | 0.81 | 0.18 | 64,64,65,76 | 0 |
| 5 | FMT | B | 535 | 3/3 | 0.81 | 0.25 | 88,88,89,94 | 0 |
| 5 | FMT | C | 510 | 3/3 | 0.81 | 0.15 | 83,83,84,91 | 0 |
| 5 | FMT | A | 553 | 3/3 | 0.81 | 0.22 | 86,86,94,95 | 0 |
| 5 | FMT | D | 523 | 3/3 | 0.81 | 0.26 | 85,85,94,95 | 0 |
| 5 | FMT | A | 510 | 3/3 | 0.81 | 0.15 | 75,75,84,85 | 0 |

*Continued on next page...*

CONFIDENTIAL VALIDATION REPORT

*Continued from previous page...*

| Mol | Type | Chain | Res | Atoms | RSCC | RSR | B-factors(Å$^2$) | Q<0.9 |
|---|---|---|---|---|---|---|---|---|
| 5 | FMT | A | 572 | 3/3 | 0.81 | 0.51 | 91,91,97,98 | 0 |
| 5 | FMT | A | 513 | 3/3 | 0.81 | 0.17 | 75,75,81,88 | 0 |
| 5 | FMT | F | 513 | 3/3 | 0.81 | 0.28 | 75,75,79,82 | 0 |
| 5 | FMT | D | 505 | 3/3 | 0.81 | 0.20 | 69,69,69,73 | 0 |
| 5 | FMT | B | 529 | 3/3 | 0.81 | 0.16 | 71,71,71,79 | 0 |
| 5 | FMT | A | 562 | 3/3 | 0.82 | 0.29 | 55,55,70,72 | 0 |
| 5 | FMT | D | 529 | 3/3 | 0.82 | 0.17 | 76,76,79,82 | 0 |
| 5 | FMT | C | 546 | 3/3 | 0.82 | 0.17 | 87,87,88,91 | 0 |
| 5 | FMT | E | 501 | 3/3 | 0.82 | 0.15 | 76,76,78,79 | 0 |
| 5 | FMT | A | 524 | 3/3 | 0.82 | 0.30 | 60,60,68,74 | 0 |
| 5 | FMT | A | 538 | 3/3 | 0.82 | 0.16 | 63,63,75,85 | 0 |
| 5 | FMT | A | 567 | 3/3 | 0.82 | 0.21 | 85,85,85,85 | 0 |
| 5 | FMT | D | 534 | 3/3 | 0.82 | 0.40 | 91,91,91,93 | 0 |
| 5 | FMT | D | 520 | 3/3 | 0.82 | 0.14 | 96,96,100,108 | 0 |
| 5 | FMT | C | 506 | 3/3 | 0.83 | 0.33 | 62,62,69,73 | 0 |
| 5 | FMT | C | 512 | 3/3 | 0.83 | 0.17 | 59,59,73,74 | 0 |
| 5 | FMT | A | 516 | 3/3 | 0.83 | 0.27 | 77,77,81,85 | 0 |
| 5 | FMT | E | 512 | 3/3 | 0.83 | 0.16 | 80,80,81,86 | 0 |
| 5 | FMT | C | 534 | 3/3 | 0.83 | 0.19 | 88,88,90,92 | 0 |
| 5 | FMT | D | 533 | 3/3 | 0.84 | 0.35 | 70,70,70,73 | 0 |
| 5 | FMT | A | 530 | 3/3 | 0.84 | 0.20 | 55,55,75,77 | 0 |
| 5 | FMT | A | 543 | 3/3 | 0.84 | 0.14 | 54,54,69,69 | 0 |
| 5 | FMT | C | 529 | 3/3 | 0.84 | 0.18 | 84,84,88,92 | 0 |
| 5 | FMT | F | 514 | 3/3 | 0.84 | 0.25 | 56,56,70,72 | 0 |
| 5 | FMT | C | 505 | 3/3 | 0.85 | 0.24 | 57,57,64,68 | 0 |
| 5 | FMT | C | 518 | 3/3 | 0.85 | 0.26 | 65,65,72,77 | 0 |
| 5 | FMT | C | 516 | 3/3 | 0.85 | 0.09 | 81,81,86,87 | 0 |
| 5 | FMT | C | 507 | 3/3 | 0.85 | 0.15 | 60,60,65,68 | 0 |
| 5 | FMT | A | 512 | 3/3 | 0.85 | 0.13 | 98,98,103,109 | 0 |
| 5 | FMT | A | 522 | 3/3 | 0.85 | 0.26 | 74,74,80,83 | 0 |
| 5 | FMT | C | 552 | 3/3 | 0.86 | 0.16 | 72,72,74,79 | 0 |
| 5 | FMT | F | 501 | 3/3 | 0.86 | 0.15 | 85,85,86,88 | 0 |
| 5 | FMT | E | 513 | 3/3 | 0.86 | 0.20 | 74,74,74,75 | 0 |
| 5 | FMT | B | 512 | 3/3 | 0.86 | 0.13 | 68,68,72,74 | 0 |
| 5 | FMT | A | 534 | 3/3 | 0.86 | 0.19 | 60,60,72,81 | 0 |
| 5 | FMT | D | 516 | 3/3 | 0.86 | 0.23 | 90,90,91,91 | 0 |
| 5 | FMT | A | 541 | 3/3 | 0.87 | 0.11 | 92,92,93,96 | 0 |
| 5 | FMT | B | 509 | 3/3 | 0.87 | 0.20 | 60,60,62,64 | 0 |
| 5 | FMT | C | 550 | 3/3 | 0.87 | 0.10 | 57,57,67,70 | 0 |
| 5 | FMT | A | 532 | 3/3 | 0.87 | 0.68 | 76,76,77,85 | 0 |
| 5 | FMT | D | 509 | 3/3 | 0.87 | 0.29 | 66,66,76,76 | 0 |
| 5 | FMT | E | 506 | 3/3 | 0.87 | 0.24 | 70,70,72,74 | 0 |

*Continued on next page...*

CONFIDENTIAL VALIDATION REPORT

*Continued from previous page...*

| Mol | Type | Chain | Res | Atoms | RSCC | RSR | B-factors(Å$^2$) | Q<0.9 |
|---|---|---|---|---|---|---|---|---|
| 5 | FMT | A | 518 | 3/3 | 0.88 | 0.22 | 58,58,66,71 | 0 |
| 5 | FMT | A | 521 | 3/3 | 0.88 | 0.20 | 49,49,63,67 | 0 |
| 5 | FMT | A | 557 | 3/3 | 0.88 | 0.19 | 87,87,90,91 | 0 |
| 5 | FMT | E | 504 | 3/3 | 0.88 | 0.14 | 63,63,63,74 | 0 |
| 5 | FMT | F | 505 | 3/3 | 0.88 | 0.15 | 79,79,84,85 | 0 |
| 5 | FMT | A | 555 | 3/3 | 0.88 | 0.24 | 61,61,65,71 | 0 |
| 5 | FMT | C | 521 | 3/3 | 0.88 | 0.18 | 59,59,70,87 | 0 |
| 5 | FMT | C | 522 | 3/3 | 0.88 | 0.22 | 54,54,62,62 | 0 |
| 3 | QR8 | E | 503 | 26/26 | 0.88 | 0.17 | 46,53,56,57 | 0 |
| 5 | FMT | C | 542 | 3/3 | 0.88 | 0.54 | 91,91,115,119 | 0 |
| 5 | FMT | A | 511 | 3/3 | 0.89 | 0.11 | 61,61,66,66 | 0 |
| 5 | FMT | D | 504 | 3/3 | 0.89 | 0.21 | 67,67,71,76 | 0 |
| 5 | FMT | A | 570 | 3/3 | 0.89 | 0.18 | 92,92,98,98 | 0 |
| 5 | FMT | A | 536 | 3/3 | 0.89 | 0.11 | 71,71,71,77 | 0 |
| 5 | FMT | C | 520 | 3/3 | 0.89 | 0.18 | 55,55,66,70 | 0 |
| 5 | FMT | A | 569 | 3/3 | 0.89 | 0.24 | 94,94,95,95 | 0 |
| 6 | NA | E | 507 | 1/1 | 0.89 | 0.34 | 81,81,81,81 | 0 |
| 5 | FMT | B | 506 | 3/3 | 0.89 | 0.14 | 63,63,69,71 | 0 |
| 5 | FMT | B | 507 | 3/3 | 0.89 | 0.12 | 75,75,84,84 | 0 |
| 5 | FMT | B | 508 | 3/3 | 0.90 | 0.09 | 73,73,79,83 | 0 |
| 5 | FMT | A | 506 | 3/3 | 0.90 | 0.31 | 66,66,73,77 | 0 |
| 5 | FMT | C | 519 | 3/3 | 0.90 | 0.10 | 69,69,76,84 | 0 |
| 5 | FMT | D | 511 | 3/3 | 0.90 | 0.25 | 68,68,73,74 | 0 |
| 5 | FMT | A | 535 | 3/3 | 0.90 | 0.19 | 89,89,103,109 | 0 |
| 5 | FMT | E | 505 | 3/3 | 0.91 | 0.12 | 72,72,79,81 | 0 |
| 5 | FMT | C | 533 | 3/3 | 0.91 | 0.13 | 68,68,69,76 | 0 |
| 2 | HEM | F | 502 | 43/43 | 0.91 | 0.15 | 53,64,75,93 | 0 |
| 5 | FMT | B | 510 | 3/3 | 0.91 | 0.16 | 86,86,91,92 | 0 |
| 5 | FMT | D | 528 | 3/3 | 0.91 | 0.59 | 79,79,80,81 | 0 |
| 5 | FMT | F | 506 | 3/3 | 0.91 | 0.10 | 74,74,75,76 | 0 |
| 3 | QR8 | F | 503 | 26/26 | 0.91 | 0.12 | 55,62,66,75 | 0 |
| 5 | FMT | A | 505 | 3/3 | 0.91 | 0.10 | 59,59,68,71 | 0 |
| 5 | FMT | D | 537 | 3/3 | 0.92 | 0.21 | 48,48,60,65 | 0 |
| 5 | FMT | A | 525 | 3/3 | 0.92 | 0.34 | 76,76,82,83 | 0 |
| 5 | FMT | A | 519 | 3/3 | 0.92 | 0.21 | 74,74,75,76 | 0 |
| 6 | NA | F | 508 | 1/1 | 0.92 | 0.16 | 59,59,59,59 | 0 |
| 5 | FMT | B | 536 | 3/3 | 0.92 | 0.09 | 69,69,76,81 | 0 |
| 5 | FMT | A | 573 | 3/3 | 0.92 | 0.13 | 67,67,85,97 | 0 |
| 5 | FMT | C | 503 | 3/3 | 0.92 | 0.11 | 48,48,55,60 | 0 |
| 3 | QR8 | A | 502 | 26/26 | 0.93 | 0.17 | 33,38,42,43 | 0 |
| 5 | FMT | A | 504 | 3/3 | 0.93 | 0.10 | 52,52,56,57 | 0 |
| 5 | FMT | D | 508 | 3/3 | 0.93 | 0.11 | 57,57,59,59 | 0 |

*Continued on next page...*

CONFIDENTIAL VALIDATION REPORT

*Continued from previous page...*

| Mol | Type | Chain | Res | Atoms | RSCC | RSR | B-factors(Å$^2$) | Q<0.9 |
|---|---|---|---|---|---|---|---|---|
| 5 | FMT | B | 505 | 3/3 | 0.93 | 0.08 | 62,62,65,69 | 0 |
| 5 | FMT | A | 514 | 3/3 | 0.93 | 0.17 | 46,46,48,51 | 0 |
| 3 | QR8 | C | 502 | 26/26 | 0.94 | 0.15 | 30,35,37,42 | 0 |
| 5 | FMT | B | 503 | 3/3 | 0.94 | 0.07 | 53,53,58,63 | 0 |
| 5 | FMT | B | 530 | 3/3 | 0.94 | 0.16 | 71,71,80,93 | 0 |
| 6 | NA | D | 513 | 1/1 | 0.94 | 0.14 | 54,54,54,54 | 0 |
| 3 | QR8 | D | 502 | 26/26 | 0.94 | 0.16 | 43,48,51,58 | 0 |
| 5 | FMT | B | 534 | 3/3 | 0.95 | 0.14 | 70,70,72,74 | 0 |
| 5 | FMT | D | 532 | 3/3 | 0.95 | 0.18 | 79,79,80,84 | 0 |
| 6 | NA | A | 526 | 1/1 | 0.95 | 0.15 | 41,41,41,41 | 0 |
| 2 | HEM | E | 502 | 43/43 | 0.95 | 0.12 | 42,48,54,58 | 0 |
| 6 | NA | B | 515 | 1/1 | 0.95 | 0.17 | 57,57,57,57 | 0 |
| 3 | QR8 | B | 502 | 26/26 | 0.95 | 0.17 | 35,41,45,48 | 0 |
| 5 | FMT | C | 513 | 3/3 | 0.95 | 0.19 | 54,54,62,63 | 0 |
| 5 | FMT | C | 514 | 3/3 | 0.96 | 0.12 | 53,53,56,64 | 0 |
| 5 | FMT | C | 538 | 3/3 | 0.96 | 0.25 | 58,58,61,64 | 0 |
| 5 | FMT | F | 507 | 3/3 | 0.96 | 0.10 | 66,66,69,72 | 0 |
| 5 | FMT | C | 524 | 3/3 | 0.97 | 0.12 | 48,48,49,52 | 0 |
| 2 | HEM | B | 501 | 43/43 | 0.97 | 0.14 | 32,36,39,47 | 0 |
| 5 | FMT | D | 510 | 3/3 | 0.97 | 0.14 | 70,70,70,72 | 0 |
| 2 | HEM | D | 501 | 43/43 | 0.97 | 0.13 | 36,40,46,49 | 0 |
| 2 | HEM | A | 501 | 43/43 | 0.97 | 0.14 | 29,32,39,41 | 0 |
| 6 | NA | C | 527 | 1/1 | 0.98 | 0.15 | 42,42,42,42 | 0 |
| 2 | HEM | C | 501 | 43/43 | 0.98 | 0.15 | 27,30,37,38 | 0 |

The following is a graphical depiction of the model fit to experimental electron density of all instances of the Ligand of Interest. In addition, ligands with molecular weight > 250 and outliers as shown on the geometry validation Tables will also be included. Each fit is shown from different orientation to approximate a three-dimensional view.

CONFIDENTIAL VALIDATION REPORT

**Electron density around QR8 E 503:**

2mF$_o$-DF$_c$ (at 0.7 rmsd) in gray
mF$_o$-DF$_c$ (at 3 rmsd) in purple (negative)
and green (positive)

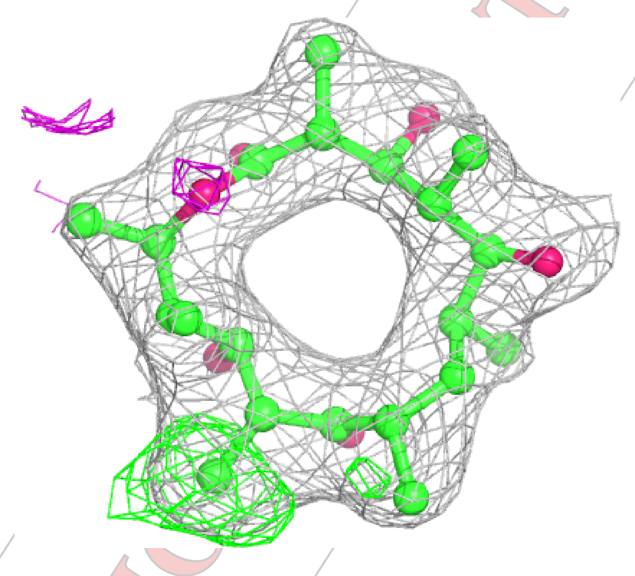

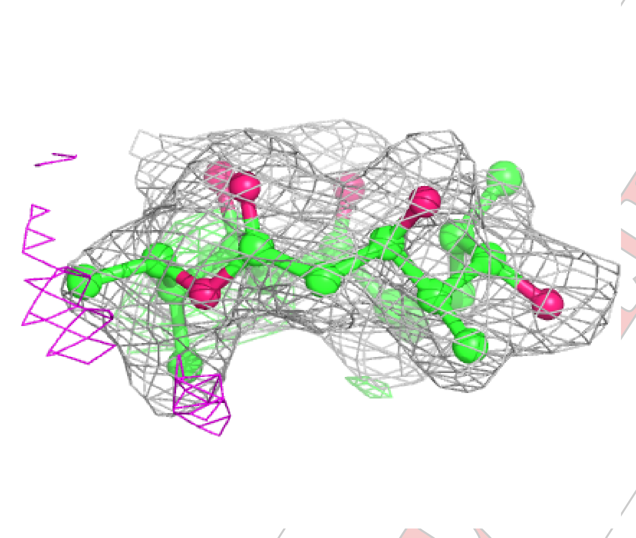

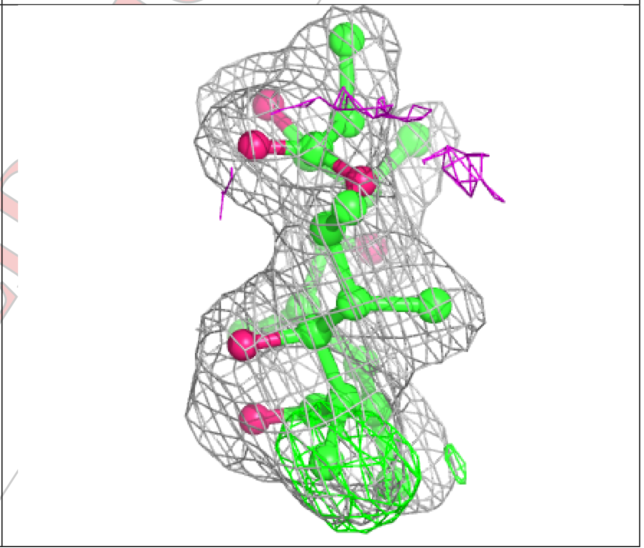

CONFIDENTIAL DRAFT

**Electron density around HEM F 502:**

$2mF_o$-$DF_c$ (at 0.7 rmsd) in gray
$mF_o$-$DF_c$ (at 3 rmsd) in purple (negative)
and green (positive)

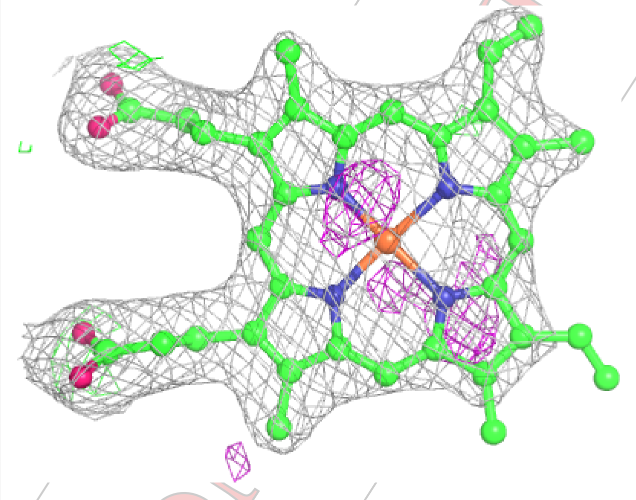

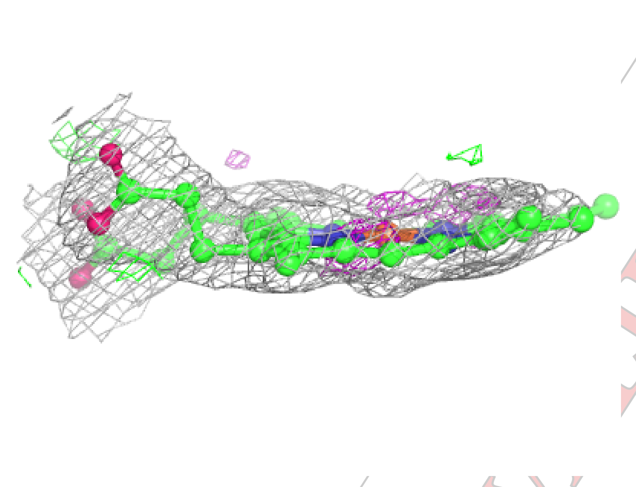

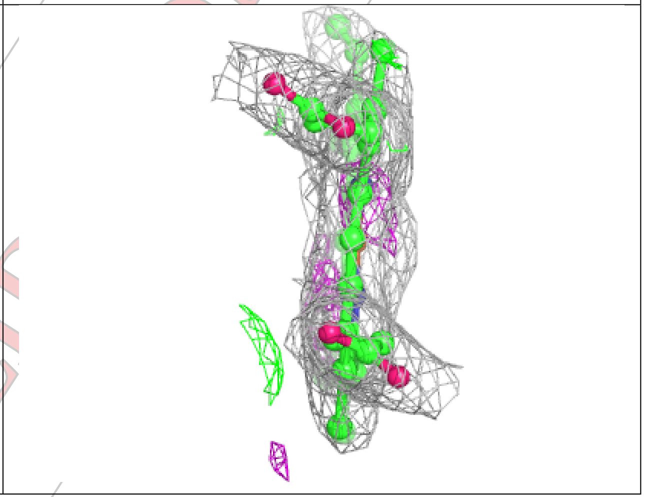

CONFIDENTIAL

**Electron density around QR8 F 503:**

$2mF_o$-$DF_c$ (at 0.7 rmsd) in gray
$mF_o$-$DF_c$ (at 3 rmsd) in purple (negative)
and green (positive)

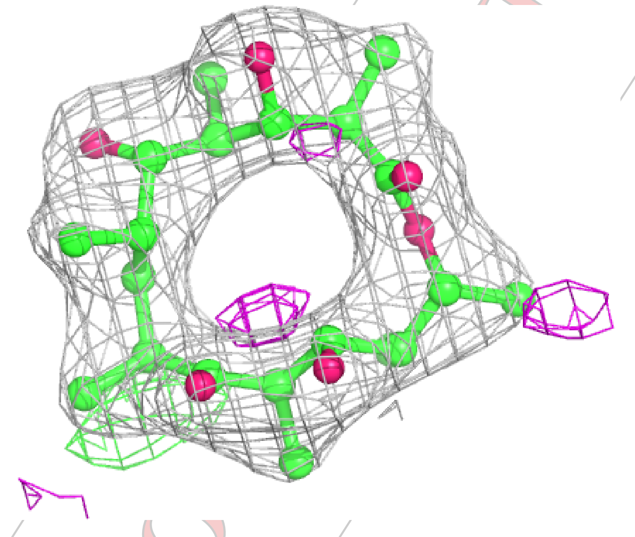

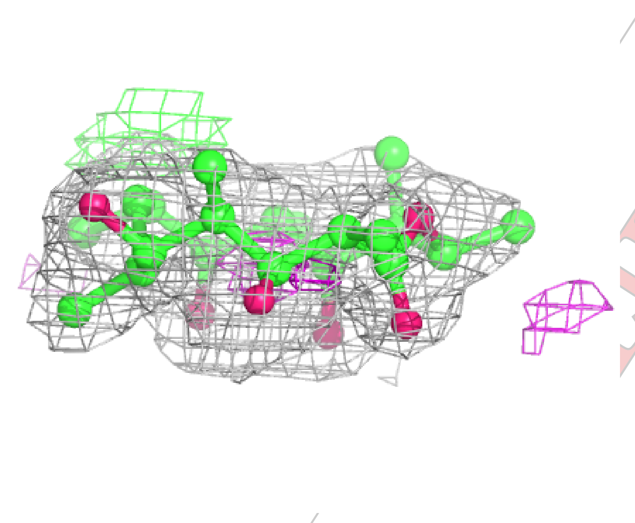

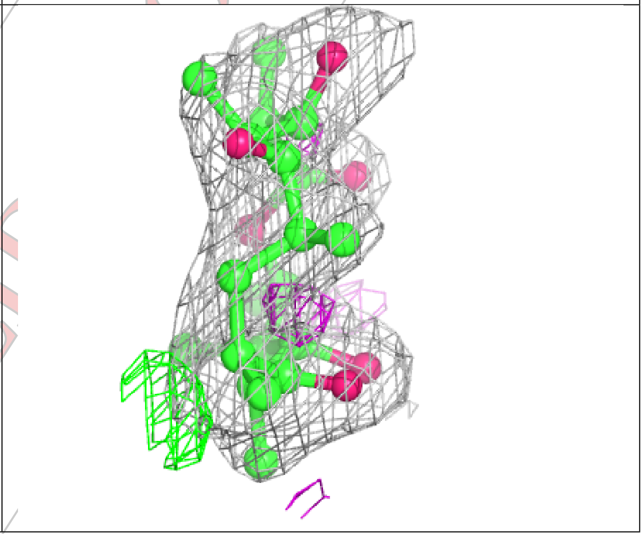

CONFIDENTIAL

**Electron density around QR8 A 502:**

$2mF_o$-$DF_c$ (at 0.7 rmsd) in gray
$mF_o$-$DF_c$ (at 3 rmsd) in purple (negative)
and green (positive)

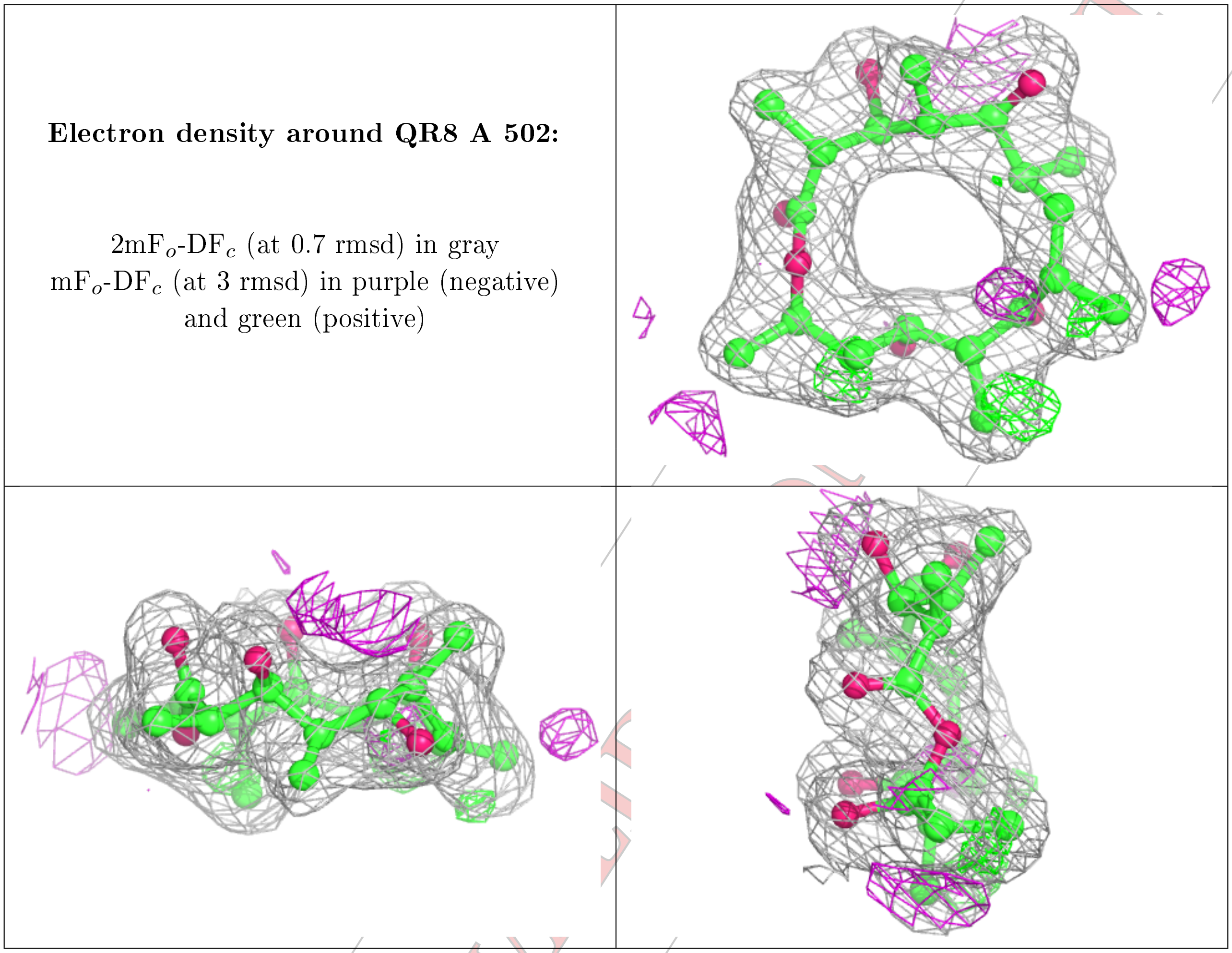

CONFIDENTIAL VALIDATION REPORT

**Electron density around QR8 C 502:**

$2mF_o$-$DF_c$ (at 0.7 rmsd) in gray
$mF_o$-$DF_c$ (at 3 rmsd) in purple (negative)
and green (positive)

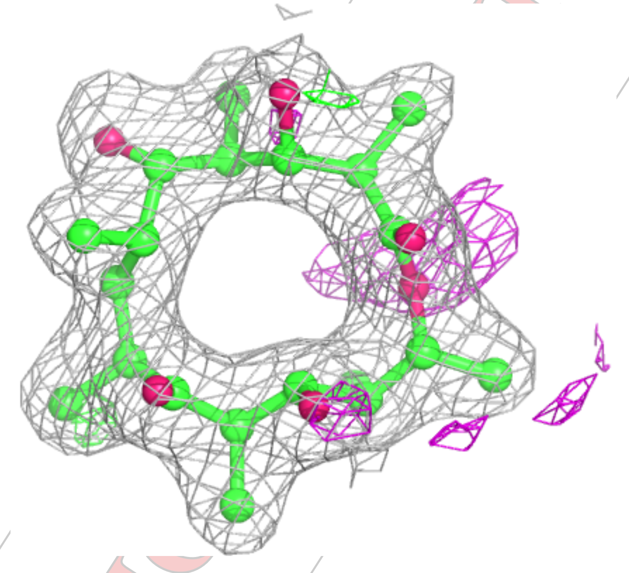

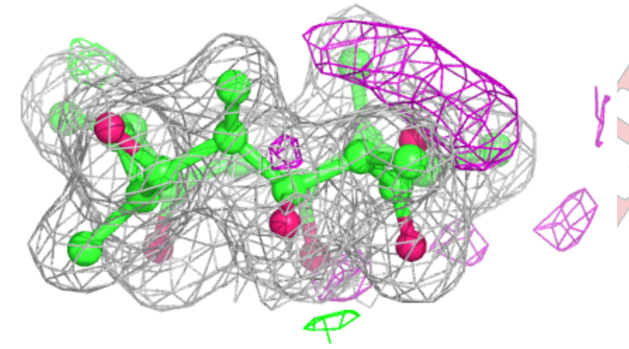

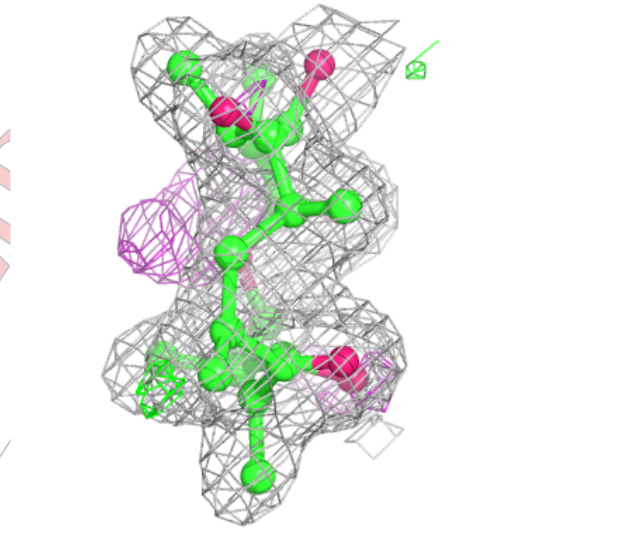

CONFIDENTIAL

**Electron density around QR8 D 502:**

$2mF_o$-$DF_c$ (at 0.7 rmsd) in gray
$mF_o$-$DF_c$ (at 3 rmsd) in purple (negative)
and green (positive)

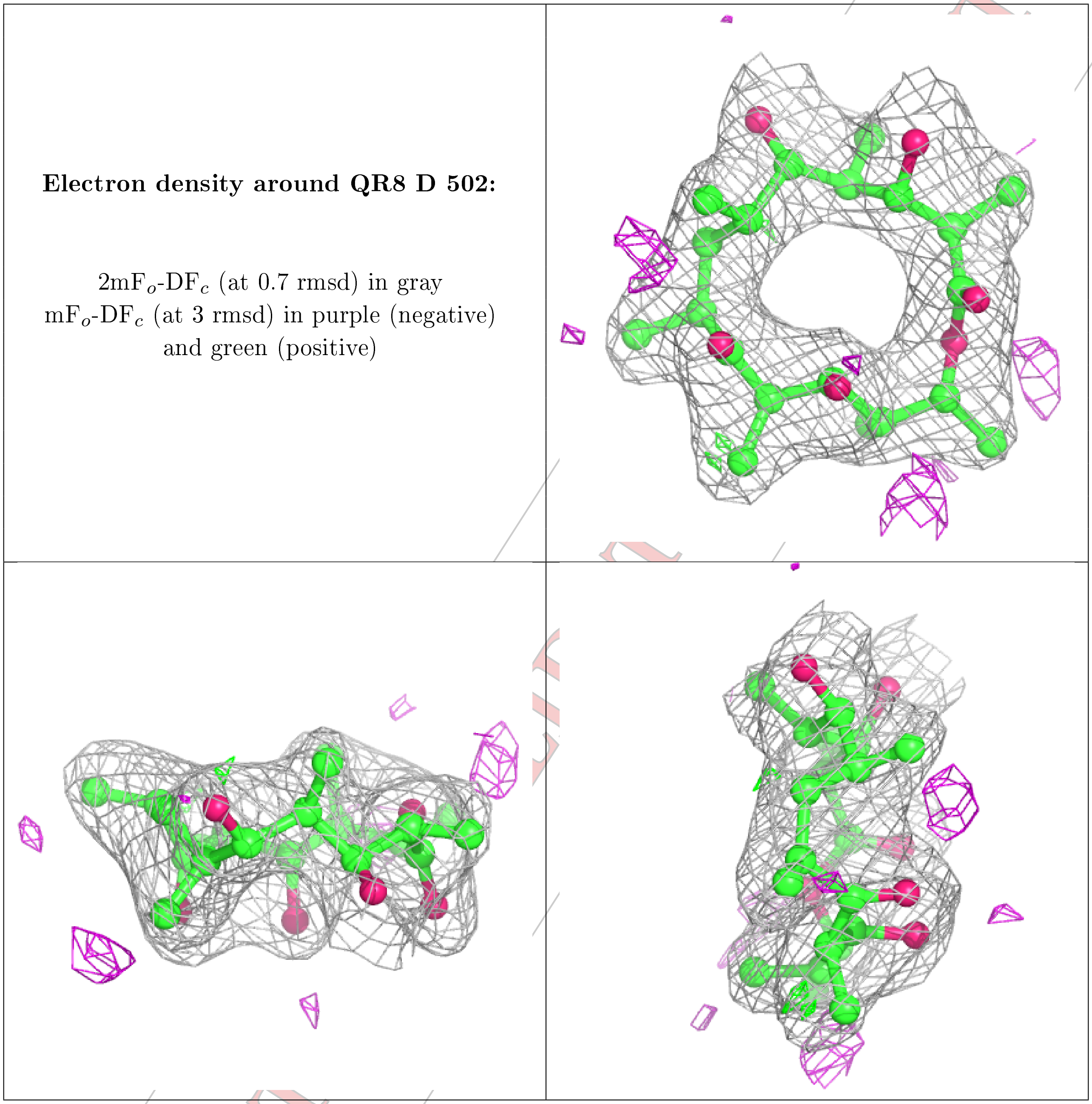

CONFIDENTIAL

**Electron density around HEM E 502:**

$2mF_o$-$DF_c$ (at 0.7 rmsd) in gray
$mF_o$-$DF_c$ (at 3 rmsd) in purple (negative)
and green (positive)

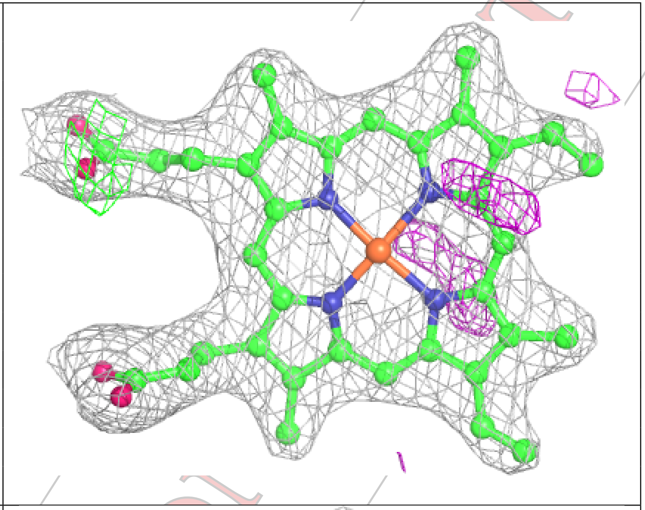

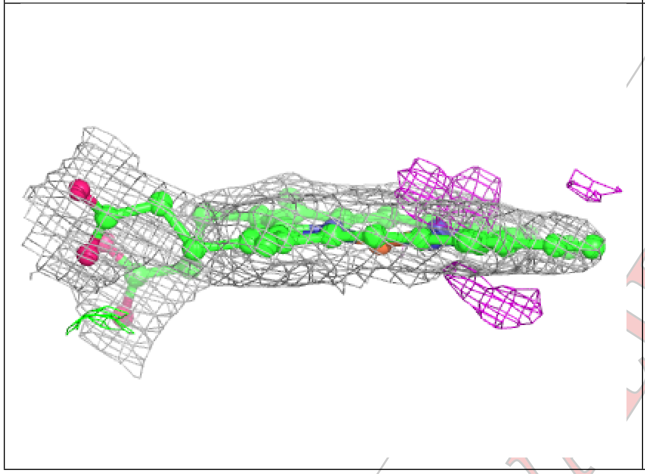

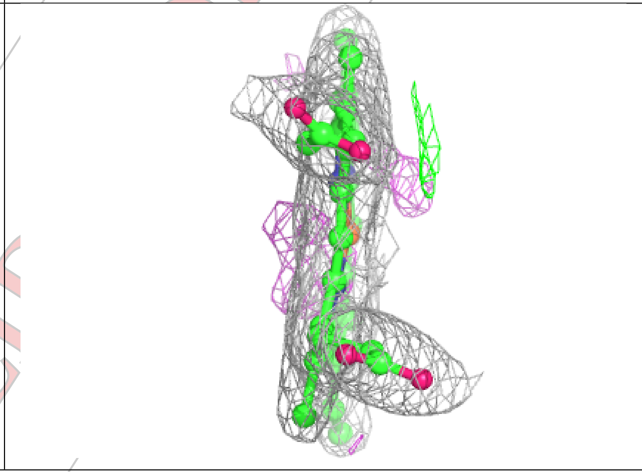

**Electron density around QR8 B 502:**

$2mF_o$-$DF_c$ (at 0.7 rmsd) in gray
$mF_o$-$DF_c$ (at 3 rmsd) in purple (negative)
and green (positive)

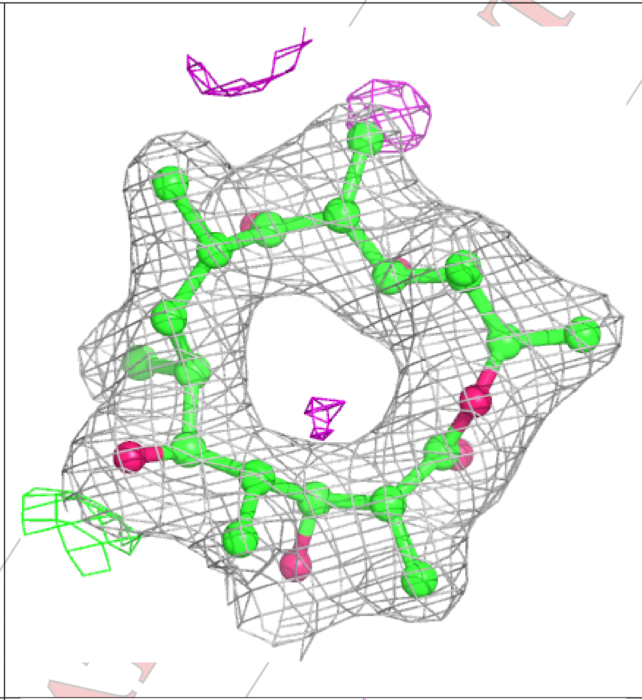

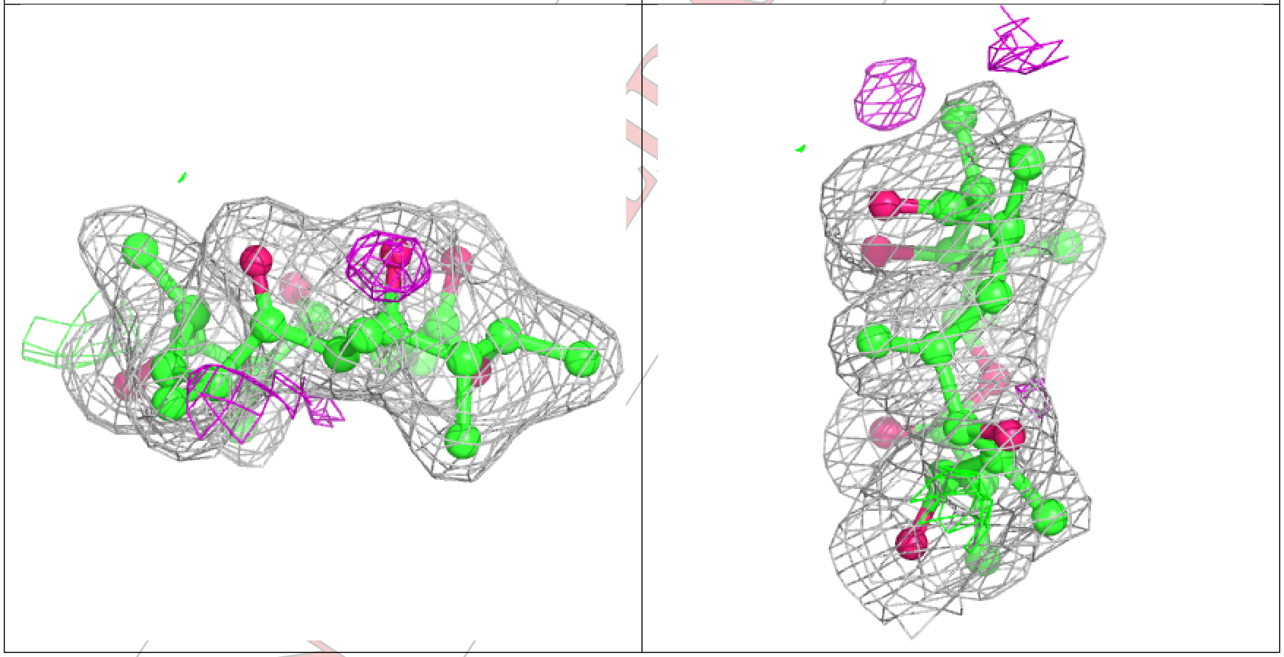

CONFIDEN

**Electron density around HEM B 501:**

2mF$_o$-DF$_c$ (at 0.7 rmsd) in gray
mF$_o$-DF$_c$ (at 3 rmsd) in purple (negative)
and green (positive)

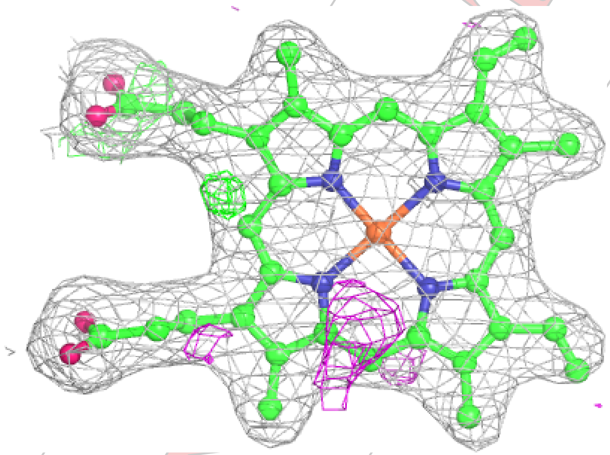

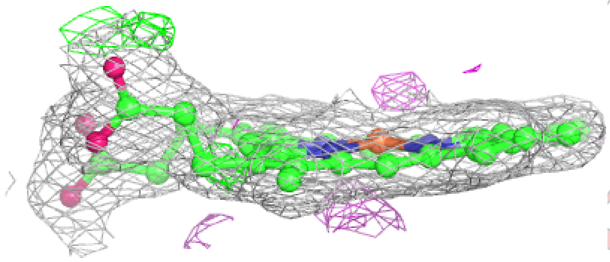

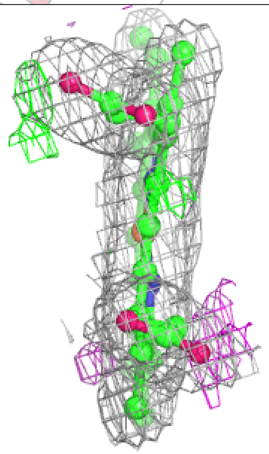

CONFIDENTIAL VALIDATION REPORT

**Electron density around HEM D 501:**

$2mF_o$-$DF_c$ (at 0.7 rmsd) in gray
$mF_o$-$DF_c$ (at 3 rmsd) in purple (negative)
and green (positive)

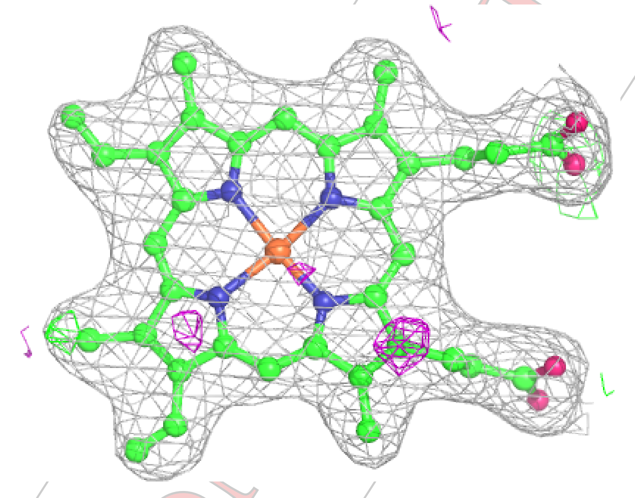

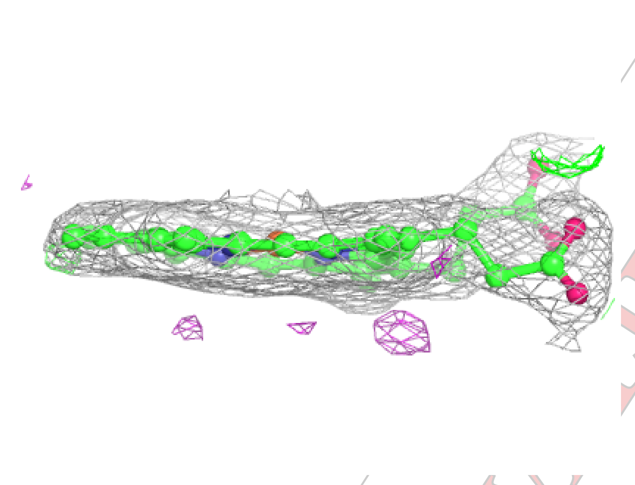

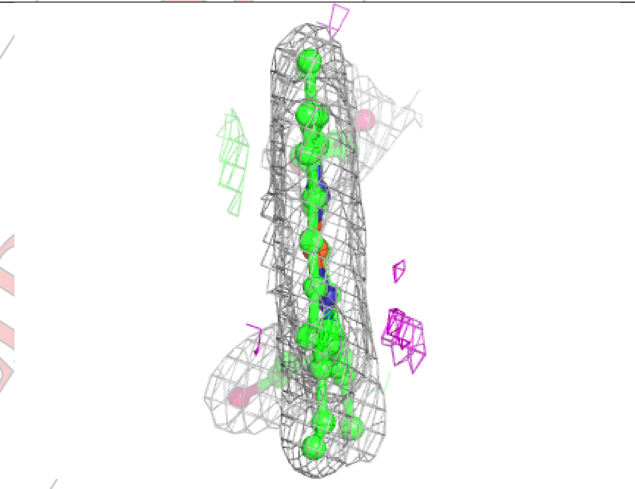

CONFIDENTIAL

**Electron density around HEM A 501:**

$2mF_o$-$DF_c$ (at 0.7 rmsd) in gray
$mF_o$-$DF_c$ (at 3 rmsd) in purple (negative)
and green (positive)

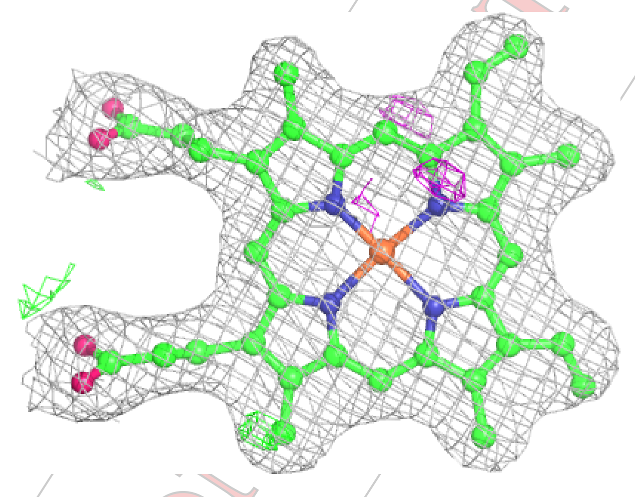

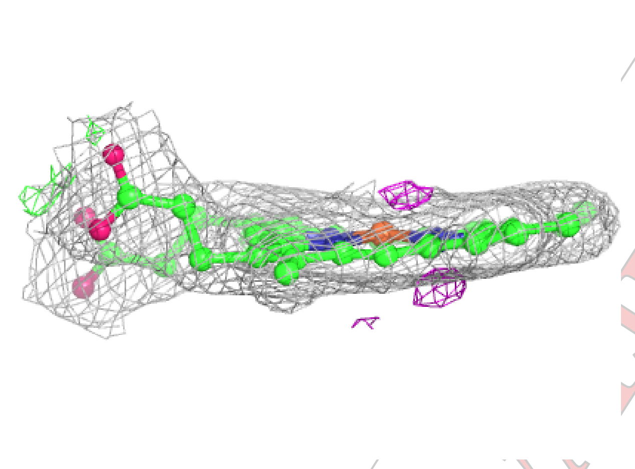

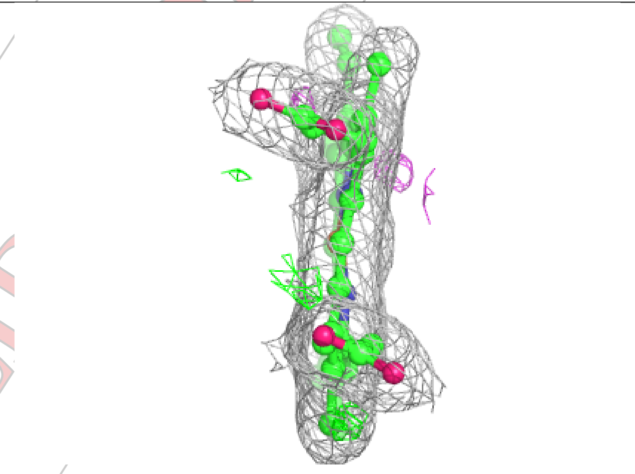

CONFIDENTIAL VALIDATION REPORT

**Electron density around HEM C 501:**

2mF$_o$-DF$_c$ (at 0.7 rmsd) in gray
mF$_o$-DF$_c$ (at 3 rmsd) in purple (negative)
and green (positive)

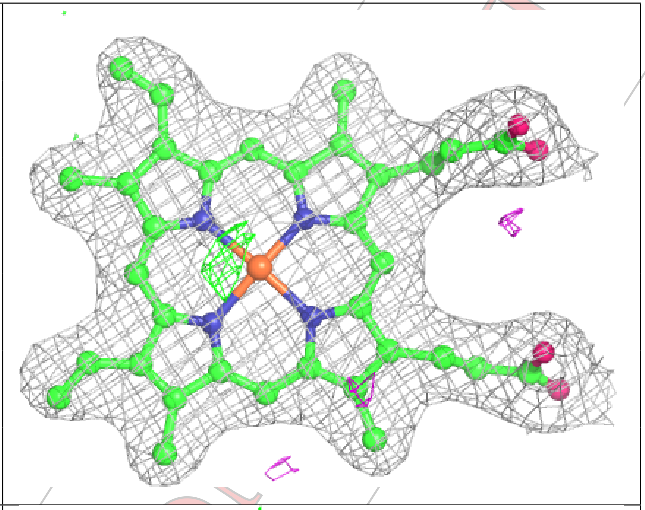

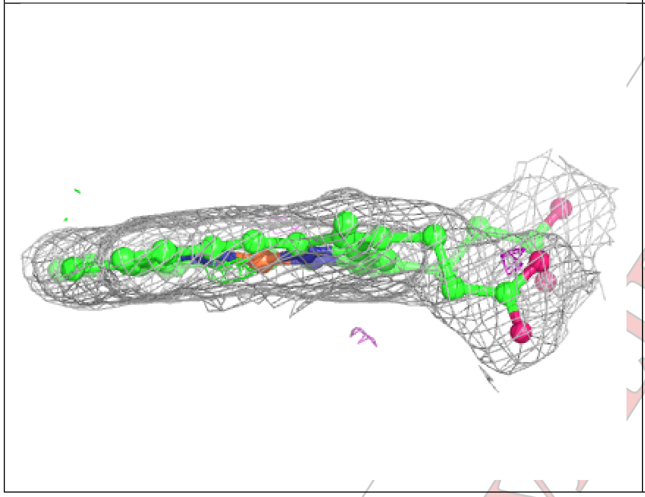

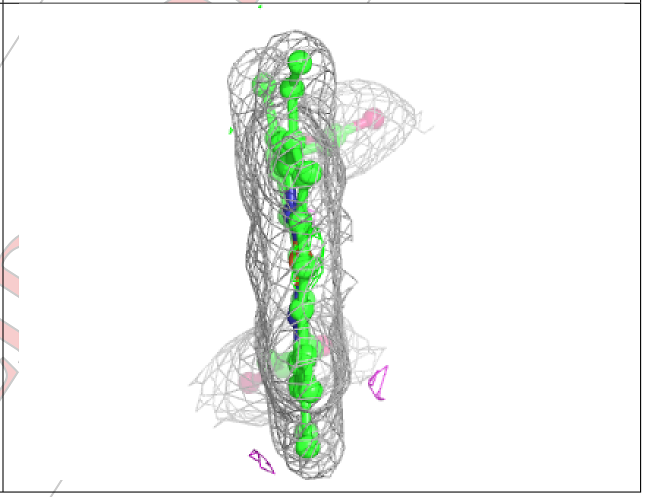

## 6.5 Other polymers (i)

There are no such residues in this entry.

CONFIDENTIAL
