# Supplementary material for: Dissecting the Cytochrome P450 OleP Substrate Specificity: Evidence for a Preferential Substrate
Source: Biomolecules. 2020 Oct 6;10(10):1411. doi: 10.3390/biom10101411 (PMC7600006; doi:10.3390/biom10101411)
Supplement: Supplementary file 1 [file biomolecules-10-01411-s001.zip › SupplMat_&_ValRep/6ZI3_D_1292109597_val-report-full_P1.pdf]

Full wwPDB X-ray Structure Validation Report (i)

Jul 13, 2020 – 10:30 PM BST

PDB ID	:	6ZI3
Title	:	Crystal structure of OleP-6DEB bound to L-rhamnose
Deposited on	:	2020-06-24
Resolution	:	2.08 Å(reported)

This is a Full wwPDB X-ray Structure Validation Report.

This report is produced by the wwPDB biocuration pipeline after annotation of the structure.

We welcome your comments at *validation@mail.wwpdb.org*
A user guide is available at
https://www.wwpdb.org/validation/2017/XrayValidationReportHelp
with specific help available everywhere you see the (i) symbol.

The following versions of software and data (see references (i)) were used in the production of this report:

MolProbity	:	4.02b-467
Mogul	:	1.8.5 (274361), CSD as541be (2020)
Xtriage (Phenix)	:	1.13
EDS	:	2.13.dev1
buster-report	:	1.1.7 (2018)
Percentile statistics	:	20191225.v01 (using entries in the PDB archive December 25th 2019)
Refmac	:	5.8.0158
CCP4	:	7.0.044 (Gargrove)
Ideal geometry (proteins)	:	Engh & Huber (2001)
Ideal geometry (DNA, RNA)	:	Parkinson et al. (1996)
Validation Pipeline (wwPDB-VP)	:	2.13.dev1

CONFIDENTIAL VALIDATION REPORT

1 Overall quality at a glance (i)

The following experimental techniques were used to determine the structure:
X-RAY DIFFRACTION

The reported resolution of this entry is 2.08 Å.

Percentile scores (ranging between 0-100) for global validation metrics of the entry are shown in the following graphic. The table shows the number of entries on which the scores are based.

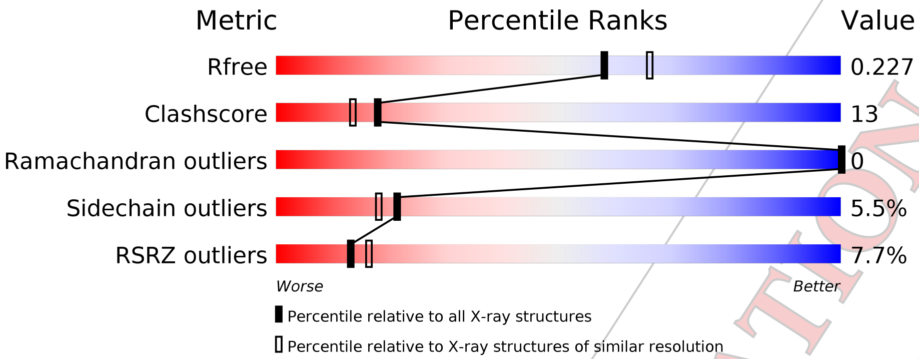

Metric	Whole archive (#Entries)	Similar resolution (#Entries, resolution range(Å))
R_{free}	130704	6189 (2.10-2.06)
Clashscore	141614	6738 (2.10-2.06)
Ramachandran outliers	138981	6663 (2.10-2.06)
Sidechain outliers	138945	6664 (2.10-2.06)
RSRZ outliers	127900	6057 (2.10-2.06)

The table below summarises the geometric issues observed across the polymeric chains and their fit to the electron density. The red, orange, yellow and green segments on the lower bar indicate the fraction of residues that contain outliers for >=3, 2, 1 and 0 types of geometric quality criteria respectively. A grey segment represents the fraction of residues that are not modelled. The numeric value for each fraction is indicated below the corresponding segment, with a dot representing fractions <=5% The upper red bar (where present) indicates the fraction of residues that have poor fit to the electron density. The numeric value is given above the bar.

Mol	Chain	Length	Quality of chain
1	A	403	4% / 79% 18% • •
1	B	403	3% / 79% 18% •
1	C	403	2% / 81% 15% • •
1	D	403	7% / 74% 21% • •
1	E	403	9% / 77% 18% • •
1	F	403	20% / 77% 19% • •

CONFIDENTIAL VALIDATION REPORT

The following table lists non-polymeric compounds, carbohydrate monomers and non-standard residues in protein, DNA, RNA chains that are outliers for geometric or electron-density-fit criteria:

Mol	Type	Chain	Res	Chirality	Geometry	Clashes	Electron density
4	RAM	A	503[A]	-	-	X	X
4	RAM	B	503[A]	-	-	X	-
4	RAM	C	503[A]	-	-	X	X
4	RAM	D	503	-	-	X	-
4	RAM	E	503[A]	-	-	X	-
5	TRS	A	504	-	-	-	X
6	FMT	A	508	-	-	X	-
6	FMT	A	514	-	-	X	-
6	FMT	A	521	-	-	-	X
6	FMT	A	523	-	-	-	X
6	FMT	A	524	-	-	-	X
6	FMT	A	525	-	-	-	X
6	FMT	A	526	-	-	-	X
6	FMT	A	537	-	-	-	X
6	FMT	A	542	-	-	X	-
6	FMT	B	513	-	-	X	-
6	FMT	B	519	-	-	X	-
6	FMT	B	530	-	-	-	X
6	FMT	B	532	-	-	-	X
6	FMT	B	538	-	-	X	-
6	FMT	B	540	-	-	X	X
6	FMT	B	542	-	-	-	X
6	FMT	B	554	-	-	-	X
6	FMT	B	556	-	-	X	-
6	FMT	B	558	-	-	-	X
6	FMT	B	563	-	-	-	X
6	FMT	B	565	-	-	X	-
6	FMT	C	513[B]	-	-	X	-
6	FMT	C	533	-	-	-	X
6	FMT	C	540	-	-	-	X
6	FMT	C	546	-	-	-	X
6	FMT	C	552	-	-	-	X
6	FMT	C	554	-	-	-	X
6	FMT	C	555[B]	-	-	X	-
6	FMT	C	560	-	-	-	X
6	FMT	C	562	-	-	-	X
6	FMT	D	504	-	-	-	X
6	FMT	D	506	-	-	-	X
6	FMT	E	504	-	-	X	-
6	FMT	E	514[B]	-	-	X	-

Continued on next page...

CONFIDENTIAL VALIDATION REPORT

Continued from previous page...

Mol	Type	Chain	Res	Chirality	Geometry	Clashes	Electron density
6	FMT	F	715	-	-	-	X
8	GOL	B	505	-	-	X	-
8	GOL	C	507	-	-	X	X

CONFIDENTIAL VALIDATION REPORT

2 Entry composition (i)

There are 9 unique types of molecules in this entry. The entry contains 22632 atoms, of which 0 are hydrogens and 0 are deuteriums.

In the tables below, the ZeroOcc column contains the number of atoms modelled with zero occupancy, the AltConf column contains the number of residues with at least one atom in alternate conformation and the Trace column contains the number of residues modelled with at most 2 atoms.

- Molecule 1 is a protein called Cytochrome P-450.

Mol	Chain	Residues	Atoms					ZeroOcc	AltConf	Trace
1	A	397	Total 3451	C 2210	N 601	O 622	S 18	0	60	0
1	B	403	Total 3347	C 2125	N 598	O 608	S 16	0	37	0
1	C	397	Total 3346	C 2120	N 601	O 609	S 16	0	39	0
1	D	396	Total 3425	C 2190	N 608	O 611	S 16	0	53	0
1	E	395	Total 3336	C 2127	N 596	O 595	S 18	0	42	0
1	F	397	Total 3395	C 2162	N 603	O 616	S 14	0	48	0

There are 36 discrepancies between the modelled and reference sequences:

Chain	Residue	Modelled	Actual	Comment	Reference
A	5	HIS	-	expression tag	UNP Q59819
A	6	THR	-	expression tag	UNP Q59819
A	7	GLY	-	expression tag	UNP Q59819
A	8	PRO	-	expression tag	UNP Q59819
A	9	THR	-	expression tag	UNP Q59819
A	10	PRO	-	expression tag	UNP Q59819
B	5	HIS	-	expression tag	UNP Q59819
B	6	THR	-	expression tag	UNP Q59819
B	7	GLY	-	expression tag	UNP Q59819
B	8	PRO	-	expression tag	UNP Q59819
B	9	THR	-	expression tag	UNP Q59819
B	10	PRO	-	expression tag	UNP Q59819
C	5	HIS	-	expression tag	UNP Q59819
C	6	THR	-	expression tag	UNP Q59819
C	7	GLY	-	expression tag	UNP Q59819
C	8	PRO	-	expression tag	UNP Q59819
C	9	THR	-	expression tag	UNP Q59819

Continued on next page...

CONFIDENTIAL VALIDATION REPORT

Continued from previous page...

Chain	Residue	Modelled	Actual	Comment	Reference
C	10	PRO	-	expression tag	UNP Q59819
D	5	HIS	-	expression tag	UNP Q59819
D	6	THR	-	expression tag	UNP Q59819
D	7	GLY	-	expression tag	UNP Q59819
D	8	PRO	-	expression tag	UNP Q59819
D	9	THR	-	expression tag	UNP Q59819
D	10	PRO	-	expression tag	UNP Q59819
E	5	HIS	-	expression tag	UNP Q59819
E	6	THR	-	expression tag	UNP Q59819
E	7	GLY	-	expression tag	UNP Q59819
E	8	PRO	-	expression tag	UNP Q59819
E	9	THR	-	expression tag	UNP Q59819
E	10	PRO	-	expression tag	UNP Q59819
F	5	HIS	-	expression tag	UNP Q59819
F	6	THR	-	expression tag	UNP Q59819
F	7	GLY	-	expression tag	UNP Q59819
F	8	PRO	-	expression tag	UNP Q59819
F	9	THR	-	expression tag	UNP Q59819
F	10	PRO	-	expression tag	UNP Q59819

- Molecule 2 is PROTOPORPHYRIN IX CONTAINING FE (three-letter code: HEM) (formula: $C_{34}H_{32}FeN_4O_4$).

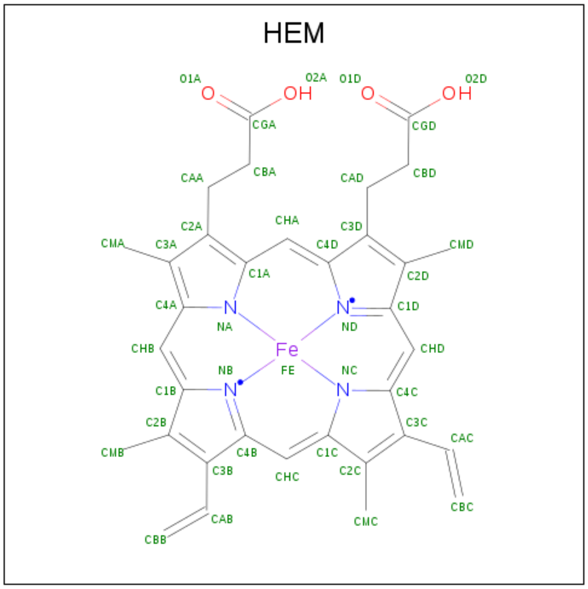

Mol	Chain	Residues	Atoms					ZeroOcc	AltConf
			Total	C	Fe	N	O		
2	A	1	43	34	1	4	4	0	0

Continued on next page...

CONFIDENTIAL VALIDATION REPORT

Continued from previous page...

Mol	Chain	Residues	Atoms					ZeroOcc	AltConf
2	B	1	Total 43	C 34	Fe 1	N 4	O 4	0	0
2	C	1	Total 43	C 34	Fe 1	N 4	O 4	0	0
2	D	1	Total 43	C 34	Fe 1	N 4	O 4	0	0
2	E	1	Total 43	C 34	Fe 1	N 4	O 4	0	0
2	F	1	Total 43	C 34	Fe 1	N 4	O 4	0	0

- Molecule 3 is 6-DEOXYERYTHRONOLIDE B (three-letter code: DEB) (formula: $C_{21}H_{38}O_6$).

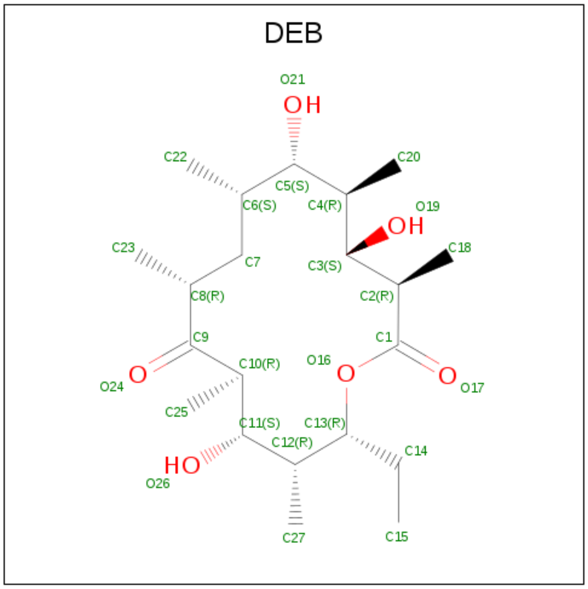

Mol	Chain	Residues	Atoms			ZeroOcc	AltConf
3	A	1	Total 27	C 21	O 6	0	0
3	B	1	Total 27	C 21	O 6	0	0
3	C	1	Total 27	C 21	O 6	0	0
3	D	1	Total 27	C 21	O 6	0	0
3	E	1	Total 27	C 21	O 6	0	0
3	F	1	Total 27	C 21	O 6	0	0

CONFIDENTIAL VALIDATION REPORT

- Molecule 4 is ALPHA-L-RHAMNOSE (three-letter code: RAM) (formula: $C_6H_{12}O_5$).

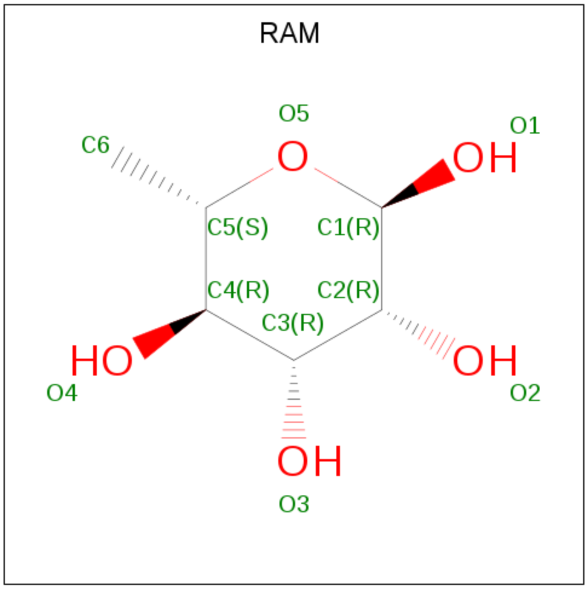

Mol	Chain	Residues	Atoms	ZeroOcc	AltConf
4	A	1	Total C O<br>11 6 5	0	1
4	B	1	Total C O<br>11 6 5	0	1
4	C	1	Total C O<br>11 6 5	0	1
4	C	1	Total C O<br>11 6 5	0	0
4	D	1	Total C O<br>11 6 5	0	0
4	E	1	Total C O<br>11 6 5	0	1
4	F	1	Total C O<br>11 6 5	0	0

- Molecule 5 is 2-AMINO-2-HYDROXYMETHYL-PROPANE-1,3-DIOL (three-letter code: TRS) (formula: $C_4H_{12}NO_3$).

CONFIDENTIAL VALIDATION REPORT

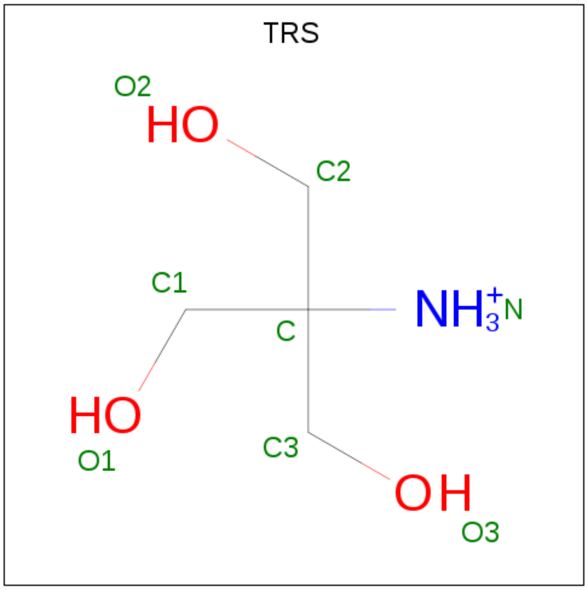

Mol	Chain	Residues	Atoms	ZeroOcc	AltConf
5	A	1	Total C N O<br>8 4 1 3	0	0
5	B	1	Total C N O<br>8 4 1 3	0	0
5	F	1	Total C N O<br>8 4 1 3	0	0

- Molecule 6 is FORMIC ACID (three-letter code: FMT) (formula: CH_2O_2).

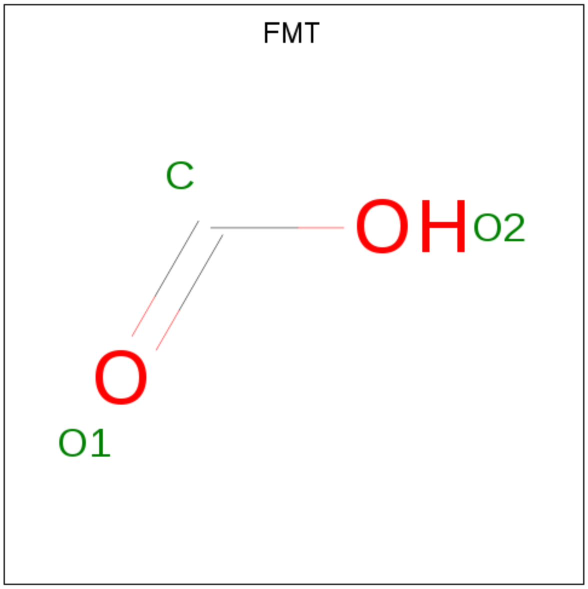

Mol	Chain	Residues	Atoms	ZeroOcc	AltConf
6	A	1	Total C O<br>3 1 2	0	0

Continued on next page...

CONFIDENTIAL VALIDATION REPORT

Continued from previous page...

Mol	Chain	Residues	Atoms	ZeroOcc	AltConf
6	A	1	Total C O<br>3 1 2	0	0
6	A	1	Total C O<br>3 1 2	0	0
6	A	1	Total C O<br>3 1 2	0	0
6	A	1	Total C O<br>3 1 2	0	0
6	A	1	Total C O<br>3 1 2	0	0
6	A	1	Total C O<br>3 1 2	0	0
6	A	1	Total C O<br>3 1 2	0	0
6	A	1	Total C O<br>3 1 2	0	0
6	A	1	Total C O<br>3 1 2	0	0
6	A	1	Total C O<br>3 1 2	0	0
6	A	1	Total C O<br>3 1 2	0	0
6	A	1	Total C O<br>3 1 2	0	0
6	A	1	Total C O<br>3 1 2	0	0
6	A	1	Total C O<br>3 1 2	0	0
6	A	1	Total C O<br>3 1 2	0	0
6	A	1	Total C O<br>3 1 2	0	0
6	A	1	Total C O<br>3 1 2	0	0
6	A	1	Total C O<br>3 1 2	0	0
6	A	1	Total C O<br>3 1 2	0	0
6	A	1	Total C O<br>3 1 2	0	0
6	A	1	Total C O<br>3 1 2	0	0

Continued on next page...

CONFIDENTIAL VALIDATION REPORT

Continued from previous page...

Mol	Chain	Residues	Atoms	ZeroOcc	AltConf
6	A	1	Total C O<br>3 1 2	0	0
6	A	1	Total C O<br>3 1 2	0	0
6	A	1	Total C O<br>3 1 2	0	0
6	A	1	Total C O<br>3 1 2	0	0
6	A	1	Total C O<br>3 1 2	0	0
6	A	1	Total C O<br>3 1 2	0	0
6	A	1	Total C O<br>3 1 2	0	0
6	A	1	Total C O<br>3 1 2	0	0
6	A	1	Total C O<br>3 1 2	0	0
6	A	1	Total C O<br>3 1 2	0	0
6	A	1	Total C O<br>3 1 2	0	0
6	A	1	Total C O<br>3 1 2	0	0
6	A	1	Total C O<br>3 1 2	0	0
6	A	1	Total C O<br>3 1 2	0	0
6	A	1	Total C O<br>3 1 2	0	0
6	A	1	Total C O<br>3 1 2	0	0
6	A	1	Total C O<br>3 1 2	0	0
6	A	1	Total C O<br>3 1 2	0	0
6	A	1	Total C O<br>3 1 2	0	1
6	B	1	Total C O<br>3 1 2	0	0
6	B	1	Total C O<br>3 1 2	0	0

Continued on next page...

CONFIDENTIAL VALIDATION REPORT

Continued from previous page...

Mol	Chain	Residues	Atoms	ZeroOcc	AltConf
6	B	1	Total C O<br>3 1 2	0	0
6	B	1	Total C O<br>3 1 2	0	0
6	B	1	Total C O<br>3 1 2	0	0
6	B	1	Total C O<br>3 1 2	0	0
6	B	1	Total C O<br>3 1 2	0	0
6	B	1	Total C O<br>3 1 2	0	0
6	B	1	Total C O<br>3 1 2	0	0
6	B	1	Total C O<br>3 1 2	0	0
6	B	1	Total C O<br>3 1 2	0	0
6	B	1	Total C O<br>3 1 2	0	0
6	B	1	Total C O<br>3 1 2	0	0
6	B	1	Total C O<br>3 1 2	0	0
6	B	1	Total C O<br>3 1 2	0	0
6	B	1	Total C O<br>3 1 2	0	0
6	B	1	Total C O<br>3 1 2	0	0
6	B	1	Total C O<br>3 1 2	0	0
6	B	1	Total C O<br>3 1 2	0	0
6	B	1	Total C O<br>3 1 2	0	0
6	B	1	Total C O<br>3 1 2	0	0
6	B	1	Total C O<br>3 1 2	0	0
6	B	1	Total C O<br>3 1 2	0	0

Continued on next page...

CONFIDENTIAL VALIDATION REPORT

Continued from previous page...

Mol	Chain	Residues	Atoms	ZeroOcc	AltConf
6	B	1	Total C O<br>3 1 2	0	0
6	B	1	Total C O<br>3 1 2	0	0
6	B	1	Total C O<br>3 1 2	0	0
6	B	1	Total C O<br>3 1 2	0	0
6	B	1	Total C O<br>3 1 2	0	0
6	B	1	Total C O<br>3 1 2	0	0
6	B	1	Total C O<br>3 1 2	0	0
6	B	1	Total C O<br>3 1 2	0	0
6	B	1	Total C O<br>3 1 2	0	0
6	B	1	Total C O<br>3 1 2	0	0
6	B	1	Total C O<br>3 1 2	0	0
6	B	1	Total C O<br>3 1 2	0	0
6	B	1	Total C O<br>3 1 2	0	0
6	B	1	Total C O<br>3 1 2	0	0
6	B	1	Total C O<br>3 1 2	0	0
6	B	1	Total C O<br>3 1 2	0	0
6	B	1	Total C O<br>3 1 2	0	0
6	B	1	Total C O<br>3 1 2	0	0
6	B	1	Total C O<br>3 1 2	0	0
6	B	1	Total C O<br>3 1 2	0	0
6	B	1	Total C O<br>3 1 2	0	0

Continued on next page...

CONFIDENTIAL VALIDATION REPORT

Continued from previous page...

Mol	Chain	Residues	Atoms	ZeroOcc	AltConf
6	B	1	Total C O<br>3 1 2	0	0
6	B	1	Total C O<br>3 1 2	0	0
6	B	1	Total C O<br>3 1 2	0	0
6	B	1	Total C O<br>3 1 2	0	0
6	B	1	Total C O<br>3 1 2	0	0
6	B	1	Total C O<br>3 1 2	0	0
6	B	1	Total C O<br>3 1 2	0	0
6	B	1	Total C O<br>3 1 2	0	0
6	B	1	Total C O<br>3 1 2	0	0
6	B	1	Total C O<br>3 1 2	0	0
6	B	1	Total C O<br>3 1 2	0	0
6	B	1	Total C O<br>3 1 2	0	0
6	B	1	Total C O<br>3 1 2	0	0
6	B	1	Total C O<br>3 1 2	0	0
6	B	1	Total C O<br>3 1 2	0	0
6	B	1	Total C O<br>3 1 2	0	0
6	B	1	Total C O<br>3 1 2	0	0
6	B	1	Total C O<br>3 1 2	0	0
6	B	1	Total C O<br>3 1 2	0	0
6	C	1	Total C O<br>3 1 2	0	0
6	C	1	Total C O<br>3 1 2	0	0

Continued on next page...

CONFIDENTIAL VALIDATION REPORT

Continued from previous page...

Mol	Chain	Residues	Atoms	ZeroOcc	AltConf
6	C	1	Total C O<br>3 1 2	0	0
6	C	1	Total C O<br>3 1 2	0	0
6	C	1	Total C O<br>3 1 2	0	0
6	C	1	Total C O<br>3 1 2	0	1
6	C	1	Total C O<br>3 1 2	0	0
6	C	1	Total C O<br>3 1 2	0	0
6	C	1	Total C O<br>3 1 2	0	0
6	C	1	Total C O<br>3 1 2	0	0
6	C	1	Total C O<br>3 1 2	0	0
6	C	1	Total C O<br>3 1 2	0	0
6	C	1	Total C O<br>3 1 2	0	0
6	C	1	Total C O<br>3 1 2	0	0
6	C	1	Total C O<br>3 1 2	0	0
6	C	1	Total C O<br>3 1 2	0	0
6	C	1	Total C O<br>3 1 2	0	0
6	C	1	Total C O<br>3 1 2	0	0
6	C	1	Total C O<br>3 1 2	0	0
6	C	1	Total C O<br>3 1 2	0	0
6	C	1	Total C O<br>3 1 2	0	0
6	C	1	Total C O<br>3 1 2	0	0
6	C	1	Total C O<br>3 1 2	0	0

Continued on next page...

CONFIDENTIAL VALIDATION REPORT

Continued from previous page...

Mol	Chain	Residues	Atoms	ZeroOcc	AltConf
6	C	1	Total C O<br>3 1 2	0	0
6	C	1	Total C O<br>3 1 2	0	0
6	C	1	Total C O<br>3 1 2	0	0
6	C	1	Total C O<br>3 1 2	0	0
6	C	1	Total C O<br>3 1 2	0	0
6	C	1	Total C O<br>3 1 2	0	0
6	C	1	Total C O<br>3 1 2	0	0
6	C	1	Total C O<br>3 1 2	0	0
6	C	1	Total C O<br>3 1 2	0	0
6	C	1	Total C O<br>3 1 2	0	0
6	C	1	Total C O<br>3 1 2	0	0
6	C	1	Total C O<br>3 1 2	0	0
6	C	1	Total C O<br>3 1 2	0	0
6	C	1	Total C O<br>3 1 2	0	0
6	C	1	Total C O<br>3 1 2	0	0
6	C	1	Total C O<br>3 1 2	0	0
6	C	1	Total C O<br>3 1 2	0	0
6	C	1	Total C O<br>3 1 2	0	0
6	C	1	Total C O<br>3 1 2	0	0
6	C	1	Total C O<br>3 1 2	0	0
6	C	1	Total C O<br>3 1 2	0	0

Continued on next page...

CONFIDENTIAL VALIDATION REPORT

Continued from previous page...

Mol	Chain	Residues	Atoms	ZeroOcc	AltConf
6	C	1	Total C O<br>3 1 2	0	0
6	C	1	Total C O<br>3 1 2	0	0
6	C	1	Total C O<br>3 1 2	0	0
6	C	1	Total C O<br>3 1 2	0	1
6	C	1	Total C O<br>3 1 2	0	0
6	C	1	Total C O<br>3 1 2	0	0
6	C	1	Total C O<br>3 1 2	0	0
6	C	1	Total C O<br>3 1 2	0	0
6	C	1	Total C O<br>3 1 2	0	0
6	C	1	Total C O<br>3 1 2	0	0
6	C	1	Total C O<br>3 1 2	0	0
6	C	1	Total C O<br>3 1 2	0	0
6	C	1	Total C O<br>3 1 2	0	0
6	C	1	Total C O<br>3 1 2	0	0
6	D	1	Total C O<br>3 1 2	0	0
6	D	1	Total C O<br>3 1 2	0	0
6	D	1	Total C O<br>3 1 2	0	0
6	D	1	Total C O<br>3 1 2	0	0
6	D	1	Total C O<br>3 1 2	0	0
6	D	1	Total C O<br>3 1 2	0	0
6	D	1	Total C O<br>3 1 2	0	0

Continued on next page...

CONFIDENTIAL VALIDATION REPORT

Continued from previous page...

Mol	Chain	Residues	Atoms	ZeroOcc	AltConf
6	D	1	Total C O<br>3 1 2	0	0
6	D	1	Total C O<br>3 1 2	0	0
6	D	1	Total C O<br>3 1 2	0	0
6	D	1	Total C O<br>3 1 2	0	0
6	D	1	Total C O<br>3 1 2	0	0
6	D	1	Total C O<br>3 1 2	0	0
6	D	1	Total C O<br>3 1 2	0	0
6	D	1	Total C O<br>3 1 2	0	0
6	D	1	Total C O<br>3 1 2	0	0
6	E	1	Total C O<br>3 1 2	0	0
6	E	1	Total C O<br>3 1 2	0	0
6	E	1	Total C O<br>3 1 2	0	0
6	E	1	Total C O<br>3 1 2	0	0
6	E	1	Total C O<br>3 1 2	0	0
6	E	1	Total C O<br>3 1 2	0	0
6	E	1	Total C O<br>3 1 2	0	0
6	E	1	Total C O<br>3 1 2	0	0
6	E	1	Total C O<br>3 1 2	0	0
6	E	1	Total C O<br>3 1 2	0	0
6	E	1	Total C O<br>3 1 2	0	1
6	F	1	Total C O<br>3 1 2	0	0

Continued on next page...

CONFIDENTIAL VALIDATION REPORT

Continued from previous page...

Mol	Chain	Residues	Atoms	ZeroOcc	AltConf
6	F	1	Total C O<br>3 1 2	0	0
6	F	1	Total C O<br>3 1 2	0	0
6	F	1	Total C O<br>3 1 2	0	0
6	F	1	Total C O<br>3 1 2	0	0
6	F	1	Total C O<br>3 1 2	0	0
6	F	1	Total C O<br>3 1 2	0	0
6	F	1	Total C O<br>3 1 2	0	0
6	F	1	Total C O<br>3 1 2	0	0
6	F	1	Total C O<br>3 1 2	0	0
6	F	1	Total C O<br>3 1 2	0	0
6	F	1	Total C O<br>3 1 2	0	0
6	F	1	Total C O<br>3 1 2	0	0
6	F	1	Total C O<br>3 1 2	0	0

- Molecule 7 is SODIUM ION (three-letter code: NA) (formula: Na).

Mol	Chain	Residues	Atoms	ZeroOcc	AltConf
7	D	1	Total Na<br>1 1	0	0
7	E	1	Total Na<br>1 1	0	0
7	B	1	Total Na<br>1 1	0	0
7	C	1	Total Na<br>1 1	0	0
7	A	2	Total Na<br>2 2	0	0
7	F	1	Total Na<br>1 1	0	0

CONFIDENTIAL VALIDATION REPORT

- Molecule 8 is GLYCEROL (three-letter code: GOL) (formula: $C_3H_8O_3$).

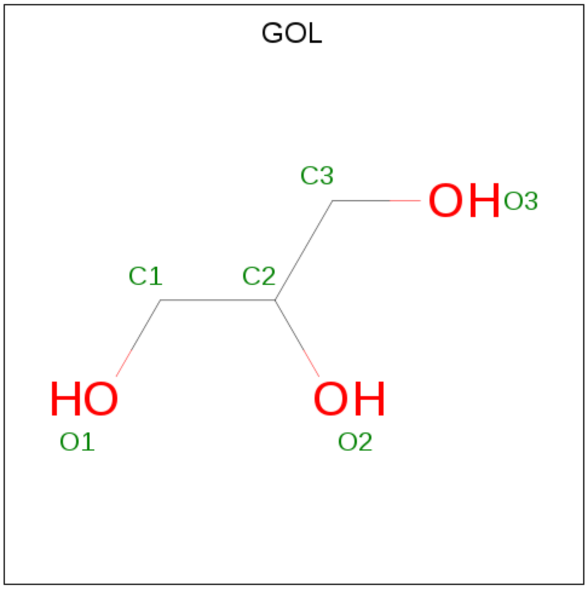

Mol	Chain	Residues	Atoms	ZeroOcc	AltConf
8	B	1	Total C O<br>6 3 3	0	0
8	B	1	Total C O<br>6 3 3	0	0
8	C	1	Total C O<br>6 3 3	0	0
8	C	1	Total C O<br>6 3 3	0	0
8	C	1	Total C O<br>6 3 3	0	0
8	F	1	Total C O<br>6 3 3	0	0

- Molecule 9 is water.

Mol	Chain	Residues	Atoms	ZeroOcc	AltConf
9	A	202	Total O<br>202 202	0	0
9	B	258	Total O<br>258 258	0	1
9	C	308	Total O<br>308 308	0	1
9	D	128	Total O<br>128 128	0	0
9	E	144	Total O<br>144 144	0	1

Continued on next page...

CONFIDENTIAL VALIDATION REPORT

Continued from previous page...

Mol	Chain	Residues	Atoms		ZeroOcc	AltConf
9	F	119	Total 119	O 119	0	0

CONFIDENTIAL VALIDATION REPORT

3 Residue-property plots (i)

These plots are drawn for all protein, RNA, DNA and oligosaccharide chains in the entry. The first graphic for a chain summarises the proportions of the various outlier classes displayed in the second graphic. The second graphic shows the sequence view annotated by issues in geometry and electron density. Residues are color-coded according to the number of geometric quality criteria for which they contain at least one outlier: green = 0, yellow = 1, orange = 2 and red = 3 or more. A red dot above a residue indicates a poor fit to the electron density (RSRZ > 2). Stretches of 2 or more consecutive residues without any outlier are shown as a green connector. Residues present in the sample, but not in the model, are shown in grey.

- Molecule 1: Cytochrome P-450

Chain A:

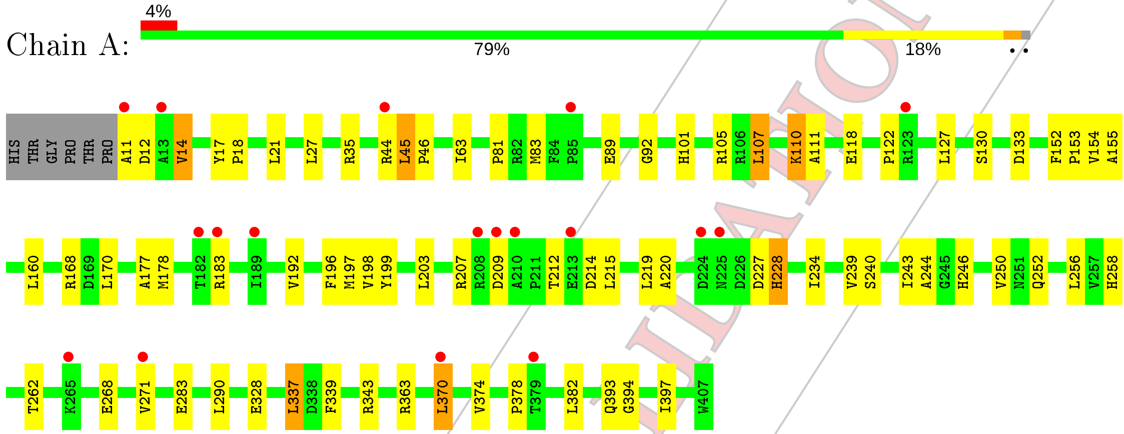

- Molecule 1: Cytochrome P-450

Chain B:

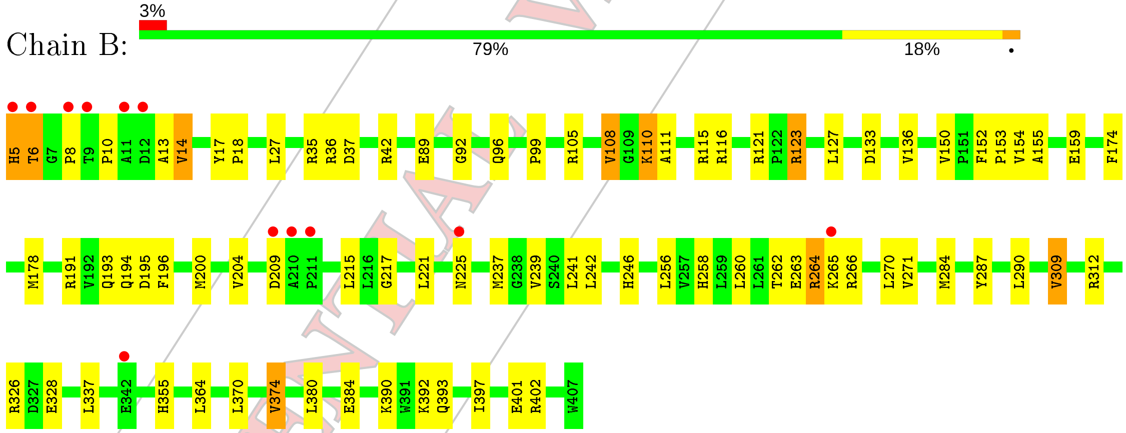

- Molecule 1: Cytochrome P-450

Chain C:

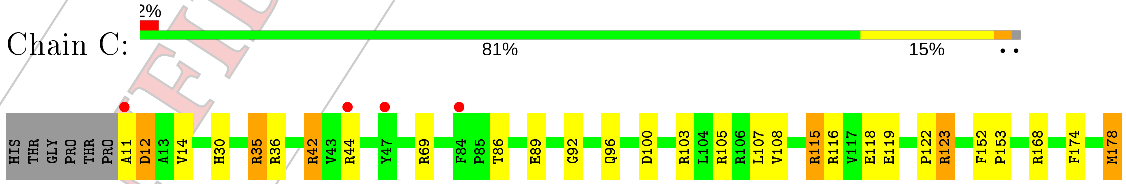

CONFIDENTIAL VALIDATION REPORT

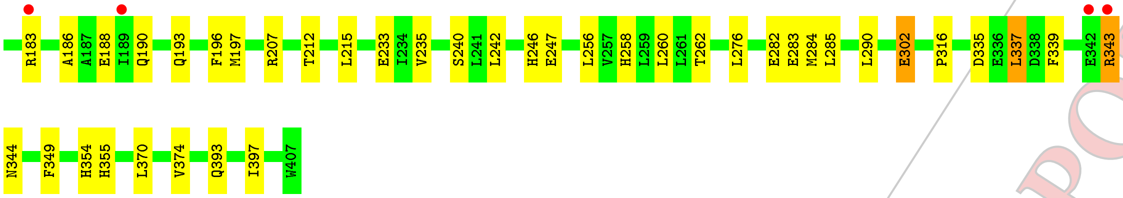

• Molecule 1: Cytochrome P-450

Chain D:

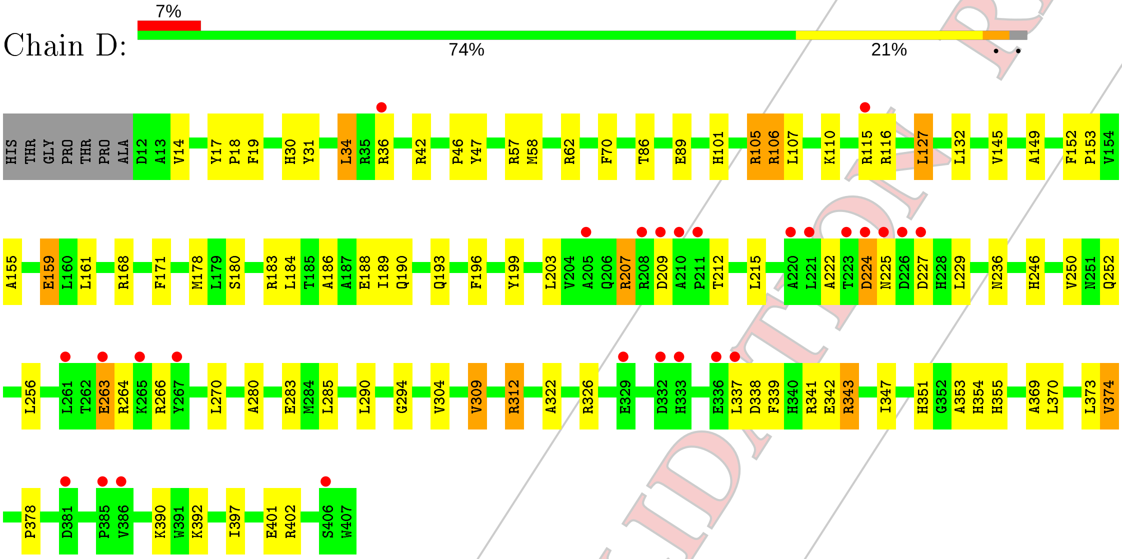

• Molecule 1: Cytochrome P-450

Chain E:

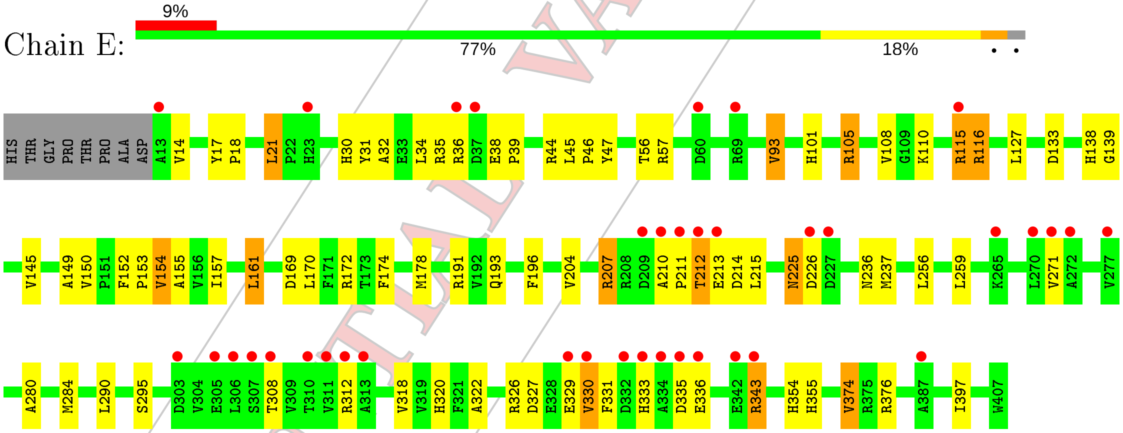

• Molecule 1: Cytochrome P-450

Chain F:

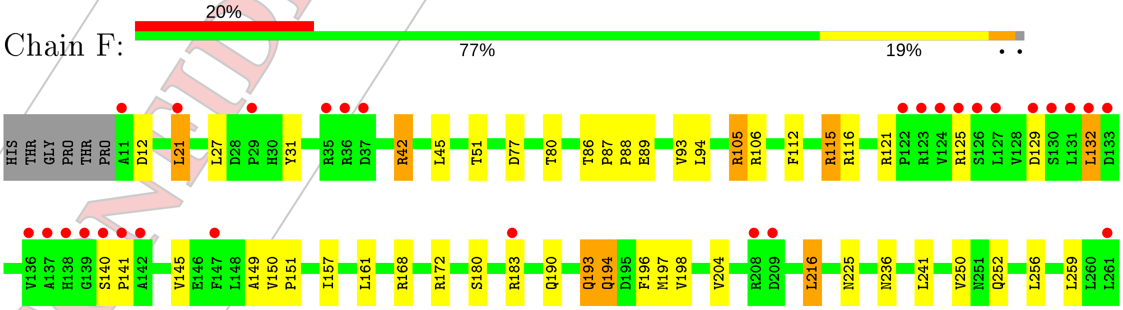

CONFIDENTIAL VALIDATION REPORT

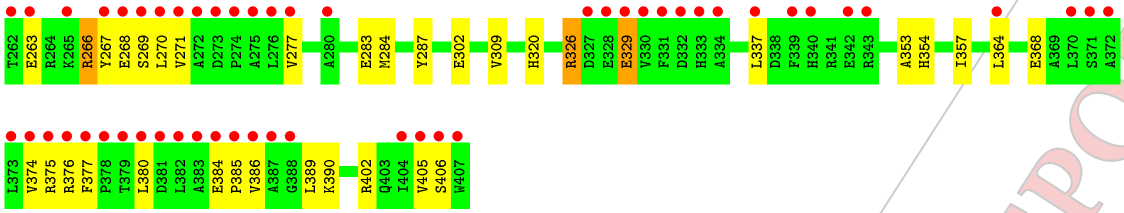

CONFIDENTIAL VALIDATION REPORT

4 Data and refinement statistics (i)

Property	Value	Source
Space group	C 1 2 1	Depositor
Cell constants<br>a, b, c, α, β, γ	247.38Å 111.15Å 159.14Å<br>90.00° 129.40° 90.00°	Depositor
Resolution (Å)	48.09 – 2.08<br>48.04 – 1.96	Depositor<br>EDS
% Data completeness<br>(in resolution range)	99.7 (48.09-2.08)<br>99.6 (48.04-1.96)	Depositor<br>EDS
R_{merge}	0.06	Depositor
R_{sym}	(Not available)	Depositor
$< I/\sigma(I) >$ [1]	1.15 (at 1.95Å)	Xtriage
Refinement program	REFMAC 5.8.0238	Depositor
R, R_{free}	0.172 , 0.225<br>0.179 , 0.227	Depositor<br>DCC
R_{free} test set	11822 reflections (4.97%)	wwPDB-VP
Wilson B-factor (Å^2)	53.5	Xtriage
Anisotropy	0.220	Xtriage
Bulk solvent k_{sol}(e/Å^3), B_{sol}(Å^2)	0.37 , 54.5	EDS
L-test for twinning[2]	$< \|L\| >$ = 0.49, $< L^2 >$ = 0.33	Xtriage
Estimated twinning fraction	0.012 for -h-2*l,-k,l	Xtriage
F_o,F_c correlation	0.98	EDS
Total number of atoms	22632	wwPDB-VP
Average B, all atoms (Å^2)	60.0	wwPDB-VP

Xtriage's analysis on translational NCS is as follows: *The analyses of the Patterson function reveals a significant off-origin peak that is 35.22 % of the origin peak, indicating pseudo-translational symmetry. The chance of finding a peak of this or larger height randomly in a structure without pseudo-translational symmetry is equal to 6.0389e-04. The detected translational NCS is most likely also responsible for the elevated intensity ratio.*

[1] Intensities estimated from amplitudes.
[2] Theoretical values of $< |L| >$, $< L^2 >$ for acentric reflections are 0.5, 0.333 respectively for untwinned datasets, and 0.375, 0.2 for perfectly twinned datasets.

CONFIDENTIAL VALIDATION REPORT

5 Model quality (i)

5.1 Standard geometry (i)

Bond lengths and bond angles in the following residue types are not validated in this section: GOL, NA, FMT, RAM, HEM, TRS, DEB

The Z score for a bond length (or angle) is the number of standard deviations the observed value is removed from the expected value. A bond length (or angle) with $|Z| > 5$ is considered an outlier worth inspection. RMSZ is the root-mean-square of all Z scores of the bond lengths (or angles).

Mol	Chain	Bond lengths		Bond angles	
		RMSZ	#$\|Z\|$ >5	RMSZ	#$\|Z\|$ >5
1	A	0.73	0/3685	0.91	3/5004 (0.1%)
1	B	0.75	0/3529	0.91	0/4798
1	C	0.79	1/3519 (0.0%)	0.97	4/4775 (0.1%)
1	D	0.78	0/3657	0.92	1/4965 (0.0%)
1	E	0.72	0/3534	0.90	3/4796 (0.1%)
1	F	0.82	2/3612 (0.1%)	0.89	1/4907 (0.0%)
All	All	0.77	3/21536 (0.0%)	0.92	12/29245 (0.0%)

All (3) bond length outliers are listed below:

Mol	Chain	Res	Type	Atoms	Z	Observed(Å)	Ideal(Å)
1	F	283	GLU	CD-OE2	21.23	1.49	1.25
1	F	283	GLU	CD-OE1	7.37	1.33	1.25
1	C	188	GLU	CD-OE1	5.19	1.31	1.25

All (12) bond angle outliers are listed below:

Mol	Chain	Res	Type	Atoms	Z	Observed(^{o})	Ideal(^{o})
1	C	35	ARG	NE-CZ-NH2	-12.01	114.29	120.30
1	A	105	ARG	NE-CZ-NH2	-9.44	115.58	120.30
1	C	35	ARG	CG-CD-NE	-7.38	96.31	111.80
1	F	105	ARG	NE-CZ-NH2	-6.88	116.86	120.30
1	C	105	ARG	NE-CZ-NH2	-6.43	117.08	120.30
1	E	105	ARG	NE-CZ-NH2	-6.19	117.21	120.30
1	C	35	ARG	NE-CZ-NH1	5.29	122.94	120.30
1	D	57	ARG	NE-CZ-NH1	5.15	122.88	120.30
1	A	105	ARG	CB-CG-CD	-5.10	98.34	111.60
1	E	335[A]	ASP	CB-CA-C	5.02	120.45	110.40
1	E	335[B]	ASP	CB-CA-C	5.02	120.45	110.40
1	A	363	ARG	NE-CZ-NH2	-5.02	117.79	120.30

CONFIDENTIAL VALIDATION REPORT

There are no chirality outliers.

There are no planarity outliers.

5.2 Too-close contacts (i)

In the following table, the Non-H and H(model) columns list the number of non-hydrogen atoms and hydrogen atoms in the chain respectively. The H(added) column lists the number of hydrogen atoms added and optimized by MolProbity. The Clashes column lists the number of clashes within the asymmetric unit, whereas Symm-Clashes lists symmetry related clashes.

Mol	Chain	Non-H	H(model)	H(added)	Clashes	Symm-Clashes
1	A	3451	0	3612	65	0
1	B	3347	0	3448	102	0
1	C	3346	0	3420	91	1
1	D	3425	0	3549	114	0
1	E	3336	0	3456	77	1
1	F	3395	0	3494	95	0
2	A	43	0	30	5	0
2	B	43	0	30	2	0
2	C	43	0	30	1	0
2	D	43	0	30	5	0
2	E	43	0	30	7	0
2	F	43	0	30	7	0
3	A	27	0	38	3	0
3	B	27	0	38	0	0
3	C	27	0	38	1	0
3	D	27	0	38	6	0
3	E	27	0	38	4	0
3	F	27	0	38	0	0
4	A	11	0	12	15	0
4	B	11	0	12	7	0
4	C	22	0	24	10	0
4	D	11	0	12	11	0
4	E	11	0	12	10	0
4	F	11	0	12	0	0
5	A	8	0	12	0	0
5	B	8	0	12	0	0
5	F	8	0	12	3	0
6	A	123	0	41	10	0
6	B	189	0	63	18	0
6	C	174	0	56	11	0
6	D	48	0	16	1	0
6	E	33	0	11	5	0
6	F	42	0	14	1	0

Continued on next page...

CONFIDENTIAL VALIDATION REPORT

Continued from previous page...

Mol	Chain	Non-H	H(model)	H(added)	Clashes	Symm-Clashes
7	A	2	0	0	0	0
7	B	1	0	0	0	0
7	C	1	0	0	0	0
7	D	1	0	0	0	0
7	E	1	0	0	0	0
7	F	1	0	0	0	0
8	B	12	0	16	4	0
8	C	18	0	24	14	0
8	F	6	0	8	1	0
9	A	202	0	0	10	0
9	B	258	0	0	4	0
9	C	308	0	0	13	0
9	D	128	0	0	8	0
9	E	144	0	0	6	0
9	F	119	0	0	3	0
All	All	22632	0	21756	582	1

The all-atom clashscore is defined as the number of clashes found per 1000 atoms (including hydrogen atoms). The all-atom clashscore for this structure is 13.

All (582) close contacts within the same asymmetric unit are listed below, sorted by their clash magnitude.

Atom-1	Atom-2	Interatomic distance (Å)	Clash overlap (Å)
1:B:10:PRO:HB2	1:B:13:ALA:CB	1.26	1.60
1:B:10:PRO:CB	1:B:13:ALA:HB3	1.25	1.58
1:C:178[B]:MET:CE	1:C:193[B]:GLN:HA	1.55	1.34
1:D:178[B]:MET:CE	1:D:193[B]:GLN:HG2	1.60	1.30
1:C:178[B]:MET:HE2	1:C:193[B]:GLN:CB	1.70	1.20
1:B:10:PRO:CB	1:B:13:ALA:CB	1.95	1.19
1:E:236:ASN:HD21	4:E:503[A]:RAM:C3	1.55	1.19
1:B:178[B]:MET:CE	1:B:193[B]:GLN:HA	1.73	1.18
1:D:107[B]:LEU:HD11	1:D:229[B]:LEU:HD23	1.17	1.13
1:C:178[B]:MET:HE3	1:C:193[B]:GLN:HA	1.14	1.12
1:F:377[B]:PHE:HB3	1:F:380[B]:LEU:CD1	1.79	1.11
1:C:178[B]:MET:CE	1:C:193[B]:GLN:CA	2.28	1.10
1:C:178[A]:MET:HE1	1:C:193[A]:GLN:HG3	1.10	1.10
1:C:178[B]:MET:HE2	1:C:193[B]:GLN:HB2	1.19	1.09
1:D:178[B]:MET:CE	1:D:193[B]:GLN:HA	1.82	1.09
1:D:178[B]:MET:HE1	1:D:193[B]:GLN:CG	1.81	1.08
1:E:236:ASN:HD21	4:E:503[A]:RAM:H3	1.13	1.08
1:F:270[B]:LEU:HD22	1:F:374[B]:VAL:HG11	1.15	1.08

Continued on next page...

CONFIDENTIAL VALIDATION REPORT

Continued from previous page...

Atom-1	Atom-2	Interatomic distance (Å)	Clash overlap (Å)
1:D:343[A]:ARG:HH11	1:D:343[A]:ARG:HB3	1.22	1.05
1:B:193[B]:GLN:HE22	8:B:505:GOL:H12	1.22	1.04
6:E:514[B]:FMT:H	9:E:663[B]:HOH:O	1.58	1.04
1:D:105[A]:ARG:NH1	1:D:355:HIS:O	1.91	1.03
1:F:129[B]:ASP:OD1	1:F:376[B]:ARG:NH2	1.92	1.02
1:A:240:SER:HA	4:A:503[A]:RAM:H61	1.45	0.99
1:C:178[B]:MET:CE	1:C:193[B]:GLN:CB	2.40	0.99
1:B:326[A]:ARG:NH1	9:B:602:HOH:O	1.95	0.98
1:D:178[B]:MET:CE	1:D:193[B]:GLN:CG	2.37	0.98
1:C:178[B]:MET:HE3	1:C:193[B]:GLN:CA	1.91	0.98
1:F:125[B]:ARG:NH1	1:F:375[B]:ARG:HH22	1.61	0.98
1:A:256[B]:LEU:HD23	1:A:370[B]:LEU:HD21	1.44	0.97
1:D:390[B]:LYS:CE	1:D:402[B]:ARG:NH2	2.28	0.96
1:C:115[A]:ARG:NH2	1:C:115[A]:ARG:HG3	1.78	0.96
1:E:57[A]:ARG:HH12	1:E:329[A]:GLU:HG3	1.29	0.94
1:E:172:ARG:NH1	9:E:601:HOH:O	2.01	0.93
1:B:10:PRO:HB3	1:B:13:ALA:CB	1.99	0.93
6:C:513[B]:FMT:O2	9:C:601[B]:HOH:O	1.87	0.93
1:F:377[B]:PHE:HB3	1:F:380[B]:LEU:HD13	1.48	0.93
1:D:178[B]:MET:HE1	1:D:193[B]:GLN:HG2	0.92	0.92
1:D:390[B]:LYS:HE2	1:D:402[B]:ARG:HH22	1.33	0.91
1:E:35[A]:ARG:HD2	1:E:57[A]:ARG:HG2	1.51	0.90
1:F:377[B]:PHE:CB	1:F:380[B]:LEU:HD13	2.02	0.90
1:C:108:VAL:HG12	1:C:215[B]:LEU:HD11	1.53	0.89
1:A:256[B]:LEU:HD23	1:A:370[B]:LEU:CD2	2.02	0.88
1:C:96:GLN:OE1	4:C:504:RAM:O1	1.92	0.88
1:B:105[A]:ARG:NH2	1:B:355:HIS:O	2.07	0.88
1:B:10:PRO:CB	1:B:13:ALA:HB2	2.03	0.87
1:E:236:ASN:ND2	4:E:503[A]:RAM:H3	1.87	0.87
1:B:178[A]:MET:SD	1:B:193[A]:GLN:HG2	2.14	0.87
1:B:193[B]:GLN:NE2	8:B:505:GOL:H12	1.90	0.87
1:C:119:GLU:OE1	9:C:602:HOH:O	1.93	0.87
2:F:702:HEM:HBB2	2:F:702:HEM:HMB2	1.54	0.87
1:F:354[B]:HIS:HE1	5:F:705:TRS:O3	1.56	0.86
1:D:107[B]:LEU:HD11	1:D:229[B]:LEU:CD2	2.04	0.86
1:C:178[A]:MET:HE1	1:C:193[A]:GLN:CG	2.03	0.86
1:C:115[A]:ARG:HH21	1:C:115[A]:ARG:HG3	1.39	0.86
1:F:270[B]:LEU:CD2	1:F:374[B]:VAL:HG21	2.07	0.85
1:F:377[B]:PHE:CB	1:F:380[B]:LEU:CD1	2.53	0.85
1:B:178[B]:MET:HE3	1:B:193[B]:GLN:HA	1.59	0.85
1:A:243:ILE:HD12	4:A:503[A]:RAM:H62	1.57	0.85

Continued on next page...

CONFIDENTIAL VALIDATION REPORT

Continued from previous page...

Atom-1	Atom-2	Interatomic distance (Å)	Clash overlap (Å)
1:B:150:VAL:O	1:B:154:VAL:HG13	1.77	0.84
3:E:502:DEB:H203	4:E:503[A]:RAM:C6	2.07	0.84
1:E:44[A]:ARG:HH11	1:E:44[A]:ARG:HG3	1.41	0.84
1:F:268[A]:GLU:HA	1:F:271[A]:VAL:HG12	1.60	0.84
1:E:57[A]:ARG:NH1	1:E:329[A]:GLU:HG3	1.93	0.83
1:D:30[A]:HIS:NE2	1:D:34[A]:LEU:HD21	1.94	0.82
1:E:236:ASN:ND2	4:E:503[A]:RAM:C3	2.39	0.82
1:C:86[B]:THR:HB	8:C:507:GOL:HO1	1.44	0.82
1:C:100:ASP:OD2	4:C:504:RAM:O5	1.98	0.81
1:B:178[B]:MET:HE2	1:B:193[B]:GLN:HA	1.59	0.81
1:A:89:GLU:HB3	4:A:503[A]:RAM:O2	1.80	0.81
1:B:178[B]:MET:CE	1:B:193[B]:GLN:CA	2.57	0.81
1:E:191:ARG:NH1	9:E:602:HOH:O	2.14	0.80
1:B:178[B]:MET:HE2	1:B:193[B]:GLN:CA	2.12	0.79
1:C:178[A]:MET:CE	1:C:193[A]:GLN:HG3	2.03	0.79
1:D:178[B]:MET:CE	1:D:193[B]:GLN:CA	2.60	0.79
1:F:377[B]:PHE:HB3	1:F:380[B]:LEU:HD12	1.66	0.78
3:D:502:DEB:O19	4:D:503:RAM:O5	2.01	0.78
1:C:178[B]:MET:HE1	1:C:193[B]:GLN:HA	1.61	0.77
1:D:390[B]:LYS:CE	1:D:402[B]:ARG:HH22	1.94	0.77
1:D:178[B]:MET:HE3	1:D:193[B]:GLN:HA	1.63	0.77
1:D:390[B]:LYS:HD2	1:D:402[B]:ARG:HH21	1.48	0.77
1:C:86[A]:THR:OG1	8:C:507:GOL:O1	2.02	0.77
1:C:343:ARG:NH1	6:C:538:FMT:O1	2.17	0.76
1:D:369:ALA:O	1:D:373[B]:LEU:HD23	1.85	0.76
1:D:236:ASN:OD1	4:D:503:RAM:O3	2.04	0.76
1:A:240:SER:CA	4:A:503[A]:RAM:H61	2.16	0.75
1:E:280:ALA:O	1:E:284[A]:MET:HG3	1.87	0.74
1:D:390[B]:LYS:CD	1:D:402[B]:ARG:NH2	2.51	0.74
1:B:10:PRO:HA	9:B:726:HOH:O	1.86	0.74
1:E:110[A]:LYS:HG2	1:E:116[A]:ARG:NH1	2.02	0.74
1:A:35[B]:ARG:HE	6:A:514:FMT:C	2.00	0.74
1:F:270[B]:LEU:HD22	1:F:374[B]:VAL:CG1	2.07	0.73
3:E:502:DEB:H203	4:E:503[A]:RAM:H63	1.71	0.73
1:F:384[A]:GLU:OE1	1:F:402:ARG:NH1	2.22	0.73
2:D:501:HEM:HBC2	2:D:501:HEM:HMC2	1.70	0.73
1:F:266:ARG:NH2	1:F:337[B]:LEU:HD12	2.04	0.73
1:F:145:VAL:HA	1:F:149:ALA:HB3	1.71	0.73
1:D:390[B]:LYS:HD2	1:D:402[B]:ARG:NH2	2.03	0.73
4:C:504:RAM:O2	9:C:604:HOH:O	2.06	0.72
1:B:8:PRO:HG3	1:C:282:GLU:HG3	1.70	0.72

Continued on next page...

CONFIDENTIAL VALIDATION REPORT

Continued from previous page...

Atom-1	Atom-2	Interatomic distance (Å)	Clash overlap (Å)
1:E:110[A]:LYS:HG2	1:E:116[A]:ARG:HH11	1.55	0.72
1:D:224:ASP:OD1	9:D:601:HOH:O	2.07	0.71
1:D:178[B]:MET:HE2	1:D:193[B]:GLN:CG	2.17	0.71
1:D:401[B]:GLU:OE2	9:D:602:HOH:O	2.08	0.71
1:F:354[B]:HIS:CE1	5:F:705:TRS:O3	2.42	0.71
1:D:343[B]:ARG:HB3	1:D:343[B]:ARG:HH21	1.55	0.71
2:E:501:HEM:HBB2	2:E:501:HEM:HHC	1.73	0.71
1:C:344:ASN:N	9:C:603:HOH:O	2.03	0.71
1:D:343[A]:ARG:CB	1:D:343[A]:ARG:HH11	2.01	0.71
1:D:322:ALA:O	1:D:326:ARG:HG2	1.91	0.70
2:F:702:HEM:CMB	2:F:702:HEM:HBB2	2.21	0.70
1:E:44[A]:ARG:NH1	1:E:44[A]:ARG:HG3	2.05	0.70
1:D:252[A]:GLN:HE21	1:D:285:LEU:HD23	1.57	0.70
3:D:502:DEB:O19	4:D:503:RAM:C5	2.39	0.69
2:D:501:HEM:HBB2	2:D:501:HEM:HMB2	1.74	0.69
1:F:190[A]:GLN:HE22	1:F:193:GLN:HE21	1.38	0.69
1:E:127[B]:LEU:HD21	1:E:155:ALA:HB3	1.74	0.69
1:D:252[B]:GLN:HA	1:D:252[B]:GLN:OE1	1.91	0.69
1:B:193[B]:GLN:HE22	8:B:505:GOL:C1	2.02	0.69
1:A:127[A]:LEU:HD21	1:A:155:ALA:HB3	1.74	0.69
1:C:260:LEU:CD1	1:C:370[B]:LEU:HD11	2.23	0.69
1:E:256:LEU:HD22	1:E:284[B]:MET:HB3	1.75	0.69
1:E:110[A]:LYS:CG	1:E:116[A]:ARG:NH1	2.56	0.69
1:C:178[A]:MET:HA	1:C:178[A]:MET:HE3	1.74	0.68
1:B:193[B]:GLN:NE2	8:B:505:GOL:C1	2.57	0.68
1:A:228:HIS:HB2	9:A:697:HOH:O	1.93	0.68
1:E:57[A]:ARG:NH1	1:E:329[A]:GLU:CG	2.56	0.68
8:C:507:GOL:H32	6:C:555[B]:FMT:O1	1.93	0.68
1:F:86:THR:HB	1:F:190[B]:GLN:HE22	1.59	0.68
1:C:174:PHE:O	1:C:178[A]:MET:HG2	1.94	0.68
1:C:256:LEU:HD22	1:C:284:MET:HB3	1.77	0.67
1:B:14:VAL:HG11	1:B:42[A]:ARG:HD3	1.75	0.67
1:E:150:VAL:O	1:E:154:VAL:HG13	1.95	0.67
1:C:115[A]:ARG:CG	1:C:115[A]:ARG:HH21	2.06	0.67
1:C:178[B]:MET:HE2	1:C:193[B]:GLN:CG	2.24	0.67
1:E:236:ASN:ND2	4:E:503[A]:RAM:O3	2.28	0.67
1:D:390[B]:LYS:CE	1:D:402[B]:ARG:HH21	2.06	0.66
1:B:35[A]:ARG:NH1	6:B:519:FMT:C	2.57	0.66
6:B:540:FMT:C	1:C:115[A]:ARG:NH1	2.58	0.66
1:F:105:ARG:CZ	1:F:357[B]:ILE:HG23	2.26	0.66
1:B:42[B]:ARG:NE	6:B:556:FMT:O1	2.29	0.66

Continued on next page...

CONFIDENTIAL VALIDATION REPORT

Continued from previous page...

Atom-1	Atom-2	Interatomic distance (Å)	Clash overlap (Å)
1:D:106[A]:ARG:NH2	1:D:110[A]:LYS:HZ1	1.93	0.66
1:E:178[B]:MET:SD	1:E:193:GLN:HG2	2.36	0.66
1:F:125[B]:ARG:NH1	1:F:375[B]:ARG:NH2	2.41	0.66
1:C:233:GLU:OE2	4:C:504:RAM:H5	1.94	0.66
1:C:123:ARG:NH1	6:C:532:FMT:O1	2.29	0.66
1:F:270[B]:LEU:CD2	1:F:374[B]:VAL:HG11	2.09	0.66
1:D:343[B]:ARG:CB	1:D:343[B]:ARG:HH21	2.09	0.65
3:D:502:DEB:O19	4:D:503:RAM:H61	1.96	0.65
1:D:184:LEU:HB2	1:D:189:ILE:HD11	1.77	0.65
1:C:108:VAL:CG1	1:C:215[B]:LEU:HD11	2.27	0.65
1:F:129[B]:ASP:OD1	1:F:376[B]:ARG:CZ	2.45	0.65
1:C:178[B]:MET:CE	1:C:193[B]:GLN:HB2	2.08	0.65
1:D:107[B]:LEU:CD1	1:D:229[B]:LEU:HD23	2.11	0.65
1:D:390[B]:LYS:CD	1:D:402[B]:ARG:HH21	2.07	0.65
1:E:333[A]:HIS:HD2	1:E:336[A]:GLU:HB2	1.62	0.65
1:C:283:GLU:HG3	1:C:337:LEU:HD22	1.79	0.64
1:B:264[A]:ARG:NH1	1:B:380:LEU:O	2.30	0.64
1:F:353:ALA:HB3	5:F:705:TRS:N	2.13	0.64
2:D:501:HEM:CMB	2:D:501:HEM:HBB2	2.28	0.64
8:C:507:GOL:HO3	6:C:555[B]:FMT:C	2.11	0.63
1:E:333[A]:HIS:CD2	1:E:336[A]:GLU:HB2	2.33	0.63
1:F:267[B]:TYR:CE1	1:F:374[B]:VAL:HG12	2.32	0.63
1:E:101[B]:HIS:CD2	1:E:354[B]:HIS:NE2	2.66	0.63
1:D:101[B]:HIS:HE1	1:D:105[B]:ARG:HE	1.46	0.63
1:D:116[B]:ARG:NH2	9:D:603:HOH:O	2.30	0.63
1:E:35[A]:ARG:CD	1:E:57[A]:ARG:HG2	2.27	0.63
1:D:390[B]:LYS:HE2	1:D:402[B]:ARG:NH2	1.99	0.62
1:B:191[B]:ARG:NH2	1:B:194[B]:GLN:OE1	2.32	0.62
6:E:514[B]:FMT:C	9:E:663[B]:HOH:O	2.30	0.62
6:A:515:FMT:H	9:A:678:HOH:O	1.99	0.62
1:B:328:GLU:HB2	6:B:519:FMT:C	2.29	0.62
1:A:183[B]:ARG:NH1	9:A:606:HOH:O	2.28	0.62
1:C:354[B]:HIS:HE1	8:C:505:GOL:O1	1.83	0.62
1:D:178[B]:MET:HE2	1:D:193[B]:GLN:CA	2.30	0.62
1:D:30[A]:HIS:CD2	1:D:34[A]:LEU:HD21	2.34	0.62
1:E:259:LEU:HB2	1:E:284[A]:MET:CE	2.30	0.62
1:F:357[B]:ILE:HD11	2:F:702:HEM:HMD3	1.81	0.61
1:F:125[B]:ARG:HD3	1:F:375[B]:ARG:HH12	1.63	0.61
1:D:390[A]:LYS:CE	1:D:401[A]:GLU:OE2	2.48	0.61
1:F:377[B]:PHE:HB2	1:F:380[B]:LEU:HD13	1.81	0.61
1:A:268:GLU:O	1:A:271[B]:VAL:HG12	2.01	0.61

Continued on next page...

CONFIDENTIAL VALIDATION REPORT

Continued from previous page...

Atom-1	Atom-2	Interatomic distance (Å)	Clash overlap (Å)
1:B:384:GLU:OE2	1:B:402[A]:ARG:HD2	2.00	0.61
1:E:105:ARG:NH2	1:E:355:HIS:O	2.23	0.61
1:A:14[A]:VAL:HG13	1:A:44[A]:ARG:NH2	2.16	0.61
1:D:390[B]:LYS:HE3	1:D:402[B]:ARG:NH2	2.15	0.61
1:B:178[A]:MET:CE	1:B:193[A]:GLN:HG2	2.31	0.61
2:D:501:HEM:HBC2	2:D:501:HEM:CMC	2.28	0.60
1:A:122:PRO:HB3	1:D:309:VAL:HG13	1.83	0.60
1:B:390[A]:LYS:NZ	1:B:402[A]:ARG:NH2	2.49	0.60
1:B:178[A]:MET:CE	4:B:503[A]:RAM:H2	2.32	0.60
6:C:513[B]:FMT:C	9:C:601[B]:HOH:O	2.47	0.60
1:A:252:GLN:O	1:A:256[A]:LEU:HG	2.01	0.60
2:A:501:HEM:HMB2	2:A:501:HEM:HBB2	1.84	0.59
6:B:507:FMT:O2	6:B:548:FMT:O2	2.20	0.59
1:D:178[B]:MET:HE1	1:D:193[B]:GLN:HA	1.81	0.59
1:A:246:HIS:O	1:A:250[B]:VAL:HG13	2.01	0.59
1:E:326:ARG:NH2	9:E:605:HOH:O	2.35	0.59
1:F:287:TYR:O	1:F:326:ARG:NH2	2.36	0.59
1:F:86:THR:HB	1:F:190[B]:GLN:NE2	2.16	0.59
1:B:326[A]:ARG:HH11	1:B:326[A]:ARG:HG2	1.67	0.59
1:C:42[B]:ARG:NE	9:C:611:HOH:O	2.36	0.59
1:C:36[A]:ARG:NH2	6:C:543:FMT:O1	2.35	0.59
1:D:17[B]:TYR:HE1	1:D:31[B]:TYR:HH	1.49	0.59
1:D:106[A]:ARG:NH2	1:D:110[A]:LYS:NZ	2.51	0.58
1:F:268[A]:GLU:HA	1:F:271[A]:VAL:CG1	2.33	0.58
1:B:312[A]:ARG:HD2	9:B:714:HOH:O	2.02	0.58
1:D:101[A]:HIS:HD2	9:D:612:HOH:O	1.86	0.58
1:C:108:VAL:HG12	1:C:215[B]:LEU:CD1	2.30	0.58
1:B:390[A]:LYS:HZ2	1:B:402[A]:ARG:NH2	2.02	0.58
1:B:159:GLU:HG2	6:B:565:FMT:H	1.85	0.58
1:D:207:ARG:HD2	1:D:212:THR:OG1	2.03	0.57
1:E:210:ALA:HB1	1:E:211:PRO:CD	2.34	0.57
1:D:264:ARG:NH2	1:D:378:PRO:O	2.37	0.57
1:B:127:LEU:HD21	1:B:155:ALA:HB3	1.85	0.57
1:C:14:VAL:HG23	1:C:44[B]:ARG:HG3	1.86	0.57
1:F:190[A]:GLN:NE2	1:F:193:GLN:HE21	2.00	0.57
1:C:193[A]:GLN:OE1	8:C:507:GOL:H12	2.04	0.57
1:C:335[A]:ASP:OD1	6:C:558:FMT:O2	2.22	0.57
1:B:178[A]:MET:HE3	4:B:503[A]:RAM:H2	1.86	0.57
1:C:193[A]:GLN:OE1	8:C:507:GOL:C1	2.53	0.57
1:E:207:ARG:NH2	1:E:214:ASP:OD2	2.38	0.57
1:B:5:HIS:CD2	1:B:5:HIS:N	2.73	0.56

Continued on next page...

CONFIDENTIAL VALIDATION REPORT

Continued from previous page...

Atom-1	Atom-2	Interatomic distance (Å)	Clash overlap (Å)
1:F:140:SER:OG	1:F:406:SER:HA	2.05	0.56
1:F:263[A]:GLU:OE2	1:F:263[A]:GLU:HA	2.04	0.56
3:E:502:DEB:O19	4:E:503[A]:RAM:H63	2.03	0.56
1:B:260:LEU:HD12	1:B:370[B]:LEU:HD11	1.87	0.56
1:C:89:GLU:HB2	1:C:193[B]:GLN:NE2	2.21	0.56
1:C:12:ASP:HB3	9:C:798:HOH:O	2.04	0.56
1:B:256:LEU:HD22	1:B:284:MET:HB3	1.86	0.56
1:B:239:VAL:HG11	4:B:503[A]:RAM:O3	2.06	0.56
1:C:260:LEU:HD11	1:C:370[B]:LEU:HD11	1.88	0.56
1:E:57[A]:ARG:HH12	1:E:329[A]:GLU:CG	2.09	0.56
1:C:11:ALA:N	9:C:614:HOH:O	2.39	0.55
1:B:108[A]:VAL:CG2	1:B:215:LEU:HD22	2.36	0.55
1:D:17[B]:TYR:OH	1:D:294:GLY:N	2.37	0.55
3:D:502:DEB:O19	4:D:503:RAM:C6	2.54	0.55
1:F:270[B]:LEU:HD23	1:F:374[B]:VAL:HG21	1.85	0.55
9:A:720:HOH:O	1:D:312:ARG:HD3	2.05	0.55
3:D:502:DEB:HO9	4:D:503:RAM:C5	2.17	0.55
1:C:240:SER:OG	4:C:503[A]:RAM:H1	2.07	0.55
1:C:30:HIS:HE1	9:C:851:HOH:O	1.88	0.55
1:D:152:PHE:HB3	1:D:153:PRO:HD3	1.87	0.55
1:B:35[A]:ARG:NH1	6:B:519:FMT:O1	2.40	0.55
1:C:42[B]:ARG:HD2	9:C:611:HOH:O	2.07	0.55
1:E:259:LEU:HB2	1:E:284[A]:MET:HE1	1.88	0.55
1:D:193[B]:GLN:NE2	4:D:503:RAM:O2	2.39	0.55
4:D:503:RAM:H63	9:D:615:HOH:O	2.06	0.54
1:A:243:ILE:HD12	4:A:503[A]:RAM:C6	2.32	0.54
1:C:183[B]:ARG:HD2	1:C:393:GLN:O	2.07	0.54
1:D:246:HIS:O	1:D:250:VAL:HG23	2.07	0.54
1:D:178[B]:MET:HE2	1:D:193[B]:GLN:HA	1.82	0.54
1:D:252[A]:GLN:HG3	1:D:256:LEU:HD13	1.89	0.54
1:A:240:SER:HA	4:A:503[A]:RAM:C6	2.28	0.54
1:D:17[A]:TYR:CD1	1:D:19[A]:PHE:CE2	2.95	0.54
1:D:390[A]:LYS:HE2	1:D:401[A]:GLU:OE2	2.07	0.54
2:A:501:HEM:CMB	2:A:501:HEM:HBB2	2.38	0.54
1:F:375[A]:ARG:HH11	1:F:376[A]:ARG:HG3	1.73	0.54
1:F:268[A]:GLU:CA	1:F:271[A]:VAL:HG12	2.35	0.54
1:E:210:ALA:HB1	1:E:211:PRO:HD2	1.89	0.53
1:A:170:LEU:C	1:A:170:LEU:HD23	2.29	0.53
1:B:309:VAL:HG13	1:C:122:PRO:HB3	1.91	0.53
1:A:118:GLU:HG2	1:D:14:VAL:HG12	1.89	0.53
1:B:174:PHE:O	1:B:178[A]:MET:HG2	2.08	0.53

Continued on next page...

CONFIDENTIAL VALIDATION REPORT

Continued from previous page...

Atom-1	Atom-2	Interatomic distance (Å)	Clash overlap (Å)
1:B:209:ASP:HB3	1:F:302:GLU:OE2	2.09	0.53
2:B:501:HEM:HBC2	2:B:501:HEM:CMC	2.38	0.53
1:F:190[A]:GLN:NE2	1:F:193:GLN:NE2	2.57	0.53
1:A:154:VAL:HG11	1:A:168[B]:ARG:HG3	1.91	0.53
1:C:197[B]:MET:SD	1:C:235:VAL:HG12	2.49	0.53
1:D:89:GLU:HB2	1:D:193[A]:GLN:HE22	1.72	0.53
1:F:241:LEU:HD21	1:F:357[B]:ILE:CD1	2.39	0.52
1:F:384[B]:GLU:HG2	1:F:385:PRO:HD2	1.91	0.52
1:A:101[B]:HIS:HE1	2:A:501:HEM:O2D	1.92	0.52
1:D:266[B]:ARG:CZ	1:D:337:LEU:HD12	2.40	0.52
3:D:502:DEB:H5	4:D:503:RAM:H5	1.91	0.52
1:E:46:PRO:HB2	1:E:47:TYR:CD2	2.45	0.52
1:F:256:LEU:HD22	1:F:284:MET:HB3	1.92	0.52
1:A:92:GLY:HA2	4:A:503[A]:RAM:H1	1.92	0.52
1:A:177:ALA:HB3	1:A:192:VAL:HG11	1.91	0.52
1:B:42[B]:ARG:CD	6:B:556:FMT:O1	2.57	0.52
1:C:178[B]:MET:CE	1:C:193[B]:GLN:CG	2.85	0.52
2:E:501:HEM:HBB2	2:E:501:HEM:CHC	2.37	0.52
1:F:266:ARG:HD2	1:F:337[A]:LEU:HD23	1.91	0.52
1:E:145:VAL:HA	1:E:149:ALA:HB3	1.92	0.52
1:D:101[B]:HIS:CE1	1:D:105[B]:ARG:HG3	2.44	0.52
1:B:108[A]:VAL:HG22	1:B:215:LEU:HD22	1.91	0.52
1:C:89:GLU:N	8:C:507:GOL:O3	2.39	0.52
1:A:393[A]:GLN:NE2	9:A:601:HOH:O	2.17	0.52
3:A:502:DEB:O19	4:A:503[A]:RAM:O4	2.25	0.52
1:B:242:LEU:O	1:B:246[A]:HIS:HD2	1.92	0.52
1:D:199:TYR:CZ	1:D:203:LEU:HD11	2.45	0.51
1:F:270[B]:LEU:HD21	1:F:374[B]:VAL:HG21	1.89	0.51
1:C:89:GLU:HB2	1:C:193[B]:GLN:HE22	1.75	0.51
1:C:178[B]:MET:CE	1:C:193[B]:GLN:HG3	2.40	0.51
1:A:12:ASP:HA	1:A:44[A]:ARG:NH2	2.25	0.51
1:A:92:GLY:CA	4:A:503[A]:RAM:H1	2.40	0.51
1:C:242:LEU:O	1:C:246[A]:HIS:HD2	1.93	0.51
1:F:180:SER:HB2	1:F:183[A]:ARG:HB2	1.91	0.51
6:B:540:FMT:O1	1:C:115[A]:ARG:NH1	2.43	0.51
1:E:354[B]:HIS:HD2	2:E:501:HEM:O1D	1.94	0.51
1:F:89:GLU:HB3	8:F:704:GOL:H12	1.93	0.51
1:A:178[A]:MET:CE	4:A:503[A]:RAM:H4	2.40	0.51
1:B:27[B]:LEU:CD2	1:B:326[B]:ARG:HG3	2.41	0.50
1:C:178[A]:MET:HA	1:C:178[A]:MET:CE	2.39	0.50
1:E:333[A]:HIS:CD2	1:E:336[A]:GLU:OE1	2.64	0.50

Continued on next page...

CONFIDENTIAL VALIDATION REPORT

Continued from previous page...

Atom-1	Atom-2	Interatomic distance (Å)	Clash overlap (Å)
1:A:246:HIS:O	1:A:250[A]:VAL:HG23	2.11	0.50
6:A:542:FMT:O2	9:A:603:HOH:O	2.18	0.50
1:F:256:LEU:O	1:F:284:MET:HE1	2.11	0.50
1:F:266:ARG:CZ	1:F:337[B]:LEU:HD12	2.40	0.50
1:E:225:ASN:ND2	1:E:225:ASN:N	2.59	0.50
1:B:178[B]:MET:HE2	1:B:193[B]:GLN:CB	2.40	0.50
1:E:17:TYR:HA	1:E:18:PRO:C	2.32	0.50
1:C:258:HIS:CE1	1:C:262:THR:HG21	2.47	0.50
6:A:512:FMT:C	6:A:542:FMT:O2	2.60	0.50
1:B:309:VAL:HG13	1:C:122:PRO:CB	2.42	0.50
1:E:31:TYR:CZ	1:E:320:HIS:CD2	3.00	0.50
1:F:241:LEU:CD2	1:F:357[B]:ILE:CD1	2.90	0.50
1:A:337[A]:LEU:HD13	1:A:339:PHE:CE1	2.47	0.50
1:A:17:TYR:O	1:A:46:PRO:HD3	2.12	0.49
1:A:63[B]:ILE:HD13	6:A:505:FMT:O1	2.12	0.49
1:B:209:ASP:CB	1:F:302:GLU:OE2	2.60	0.49
1:C:246[A]:HIS:CE1	1:C:247:GLU:HG2	2.47	0.49
1:B:271:VAL:HA	1:B:374[A]:VAL:HG13	1.94	0.49
1:C:183[B]:ARG:CD	1:C:393:GLN:O	2.61	0.49
1:D:145:VAL:HA	1:D:149:ALA:HB3	1.94	0.49
1:E:170:LEU:HD22	1:E:174:PHE:CZ	2.47	0.49
1:A:17:TYR:HA	1:A:18:PRO:C	2.32	0.49
1:B:239:VAL:CG1	4:B:503[A]:RAM:O3	2.60	0.49
1:E:333[A]:HIS:HD2	1:E:336[A]:GLU:OE1	1.95	0.49
1:D:127:LEU:HD13	1:D:152:PHE:HD1	1.76	0.49
1:D:58[A]:MET:HE1	1:D:347:ILE:CG2	2.43	0.49
1:E:212:THR:OG1	1:E:212:THR:O	2.18	0.49
2:E:501:HEM:HMC2	2:E:501:HEM:HBC2	1.95	0.49
4:E:503[A]:RAM:O2	6:E:506:FMT:C	2.60	0.49
1:F:125[B]:ARG:NH1	1:F:129[B]:ASP:OD2	2.45	0.49
1:A:258:HIS:CE1	1:A:262[A]:THR:HG21	2.48	0.49
1:C:42[B]:ARG:CD	9:C:611:HOH:O	2.60	0.49
1:F:380[A]:LEU:HD11	1:F:405:VAL:HG11	1.94	0.49
1:B:260:LEU:CD1	1:B:370[B]:LEU:HD11	2.43	0.49
1:D:58[A]:MET:HE1	1:D:347:ILE:HG21	1.94	0.49
1:E:322:ALA:O	1:E:326:ARG:HG2	2.12	0.49
1:A:268:GLU:HA	1:A:271[B]:VAL:HG12	1.95	0.48
1:D:161:LEU:HD23	1:D:215:LEU:HB2	1.94	0.48
1:D:17[B]:TYR:HE1	1:D:31[B]:TYR:OH	1.96	0.48
1:A:107[B]:LEU:HD21	1:A:219:LEU:HD22	1.95	0.48
1:F:77:ASP:HB3	1:F:80:THR:OG1	2.14	0.48

Continued on next page...

CONFIDENTIAL VALIDATION REPORT

Continued from previous page...

Atom-1	Atom-2	Interatomic distance (Å)	Clash overlap (Å)
1:B:123:ARG:HE	6:B:565:FMT:C	2.26	0.48
1:D:127:LEU:HD11	1:D:155:ALA:HB3	1.96	0.48
1:E:354[B]:HIS:HE1	9:E:644:HOH:O	1.96	0.48
1:F:375[A]:ARG:HH11	1:F:376[A]:ARG:CG	2.26	0.48
1:A:101[B]:HIS:HD2	9:A:635:HOH:O	1.96	0.48
1:A:11:ALA:O	1:A:14[A]:VAL:HG12	2.14	0.48
2:A:501:HEM:HBC2	2:A:501:HEM:CMC	2.44	0.48
1:D:343[B]:ARG:NH2	1:D:343[B]:ARG:CB	2.76	0.47
1:E:110[A]:LYS:CG	1:E:116[A]:ARG:HH11	2.21	0.47
1:E:330:VAL:HG22	1:E:331:PHE:CD2	2.49	0.47
1:E:101[A]:HIS:HE1	2:E:501:HEM:O2D	1.97	0.47
1:A:110[A]:LYS:HA	1:A:110[A]:LYS:HD3	1.53	0.47
1:A:283:GLU:HG3	1:A:337[A]:LEU:HD22	1.97	0.47
1:A:45[A]:LEU:HG	1:A:83:MET:SD	2.54	0.47
1:B:10:PRO:CA	1:B:13:ALA:CB	2.85	0.47
1:B:123:ARG:HD2	1:B:123:ARG:O	2.14	0.47
1:F:21[B]:LEU:HD23	1:F:21[B]:LEU:HA	1.78	0.47
1:F:31:TYR:CZ	1:F:320[B]:HIS:CD2	3.03	0.47
1:B:270:LEU:HB2	1:B:374[A]:VAL:HG21	1.97	0.47
1:B:99:PRO:HD2	6:B:508:FMT:O2	2.15	0.47
1:B:17:TYR:HA	1:B:18:PRO:C	2.35	0.47
1:C:339:PHE:N	8:C:506:GOL:O1	2.39	0.47
1:E:343[B]:ARG:HB2	1:E:343[B]:ARG:CZ	2.43	0.47
1:E:174:PHE:O	1:E:178[B]:MET:HG2	2.15	0.47
1:E:30:HIS:CD2	1:E:34[A]:LEU:HD11	2.50	0.47
1:B:287:TYR:CG	1:B:337:LEU:HD13	2.49	0.47
1:F:197:MET:CE	1:F:236[B]:ASN:ND2	2.78	0.47
1:A:122:PRO:CB	1:D:309:VAL:HG13	2.45	0.47
1:C:152:PHE:HB3	1:C:153:PRO:HD3	1.96	0.47
1:D:263[A]:GLU:HA	1:D:263[A]:GLU:OE1	2.15	0.47
1:B:111:ALA:HA	1:B:116[A]:ARG:HG2	1.96	0.46
1:A:127[B]:LEU:HD11	6:A:543:FMT:C	2.46	0.46
1:D:17[A]:TYR:HA	1:D:18:PRO:C	2.34	0.46
1:F:197:MET:CE	1:F:236[B]:ASN:HD22	2.28	0.46
1:F:386:VAL:O	1:F:389:LEU:HB2	2.16	0.46
1:D:101[B]:HIS:CE1	1:D:105[B]:ARG:HE	2.31	0.46
1:E:152:PHE:HB3	1:E:153:PRO:HD3	1.97	0.46
1:A:239:VAL:HG12	4:A:503[A]:RAM:H63	1.98	0.46
1:F:129[B]:ASP:CG	1:F:376[B]:ARG:HH21	2.19	0.46
1:D:392:LYS:HG2	1:D:401[B]:GLU:HG3	1.97	0.46
1:D:390[B]:LYS:HE3	1:D:402[B]:ARG:HH21	1.77	0.46

Continued on next page...

CONFIDENTIAL VALIDATION REPORT

Continued from previous page...

Atom-1	Atom-2	Interatomic distance (Å)	Clash overlap (Å)
1:D:252[B]:GLN:HG3	2:D:501:HEM:CBB	2.45	0.46
1:E:295:SER:H	1:E:318[B]:VAL:CG2	2.29	0.46
1:B:178[B]:MET:CE	1:B:193[B]:GLN:CB	2.94	0.46
1:C:290:LEU:O	1:C:397:ILE:HA	2.16	0.46
2:E:501:HEM:CBB	2:E:501:HEM:HHC	2.44	0.46
1:E:355:HIS:NE2	6:E:504:FMT:O2	2.41	0.46
1:A:382[B]:LEU:HA	1:A:382[B]:LEU:HD23	1.72	0.46
1:B:264[A]:ARG:NH2	9:B:622:HOH:O	2.48	0.46
1:C:354[B]:HIS:CE1	8:C:505:GOL:O1	2.66	0.46
1:D:30[A]:HIS:CD2	1:D:34[A]:LEU:CD2	2.98	0.46
1:D:116[B]:ARG:NE	9:D:605:HOH:O	2.38	0.45
1:C:285:LEU:HD13	1:C:349[B]:PHE:CE2	2.51	0.45
1:A:337[A]:LEU:CD1	1:A:339:PHE:CE1	2.99	0.45
1:A:212:THR:OG1	1:A:214:ASP:OD1	2.26	0.45
2:F:702:HEM:HBC2	2:F:702:HEM:CMC	2.47	0.45
1:D:188:GLU:OE2	1:E:115[B]:ARG:HB2	2.16	0.45
1:D:58[A]:MET:HE3	1:D:347:ILE:HD13	1.99	0.45
1:F:357[B]:ILE:CD1	2:F:702:HEM:HMD3	2.45	0.45
1:A:35[A]:ARG:NH2	9:A:602:HOH:O	2.17	0.45
1:B:215:LEU:HD23	1:B:215:LEU:HA	1.69	0.45
1:B:217:GLY:O	1:B:221[B]:LEU:HG	2.16	0.45
1:B:42[A]:ARG:HD2	1:C:118:GLU:OE2	2.17	0.45
1:C:14:VAL:HG23	1:C:44[B]:ARG:CG	2.46	0.45
2:C:501:HEM:HMB2	2:C:501:HEM:HBB2	1.99	0.45
9:A:720:HOH:O	1:D:312:ARG:CD	2.63	0.45
1:D:186:ALA:O	1:D:190[A]:GLN:HG2	2.16	0.45
1:C:86[B]:THR:HB	8:C:507:GOL:O1	2.14	0.45
1:D:115[A]:ARG:HA	1:D:115[A]:ARG:HD3	1.65	0.45
1:D:270:LEU:HB3	1:D:374:VAL:HG21	1.99	0.45
1:E:138[B]:HIS:HD2	1:E:139:GLY:O	1.99	0.45
1:F:252:GLN:O	1:F:256:LEU:HG	2.16	0.45
1:E:32:ALA:O	1:E:36[A]:ARG:HG2	2.16	0.45
1:A:111:ALA:HB2	1:A:215[B]:LEU:HD21	1.99	0.45
1:C:246[A]:HIS:HE1	9:C:698:HOH:O	2.00	0.45
1:F:357[B]:ILE:HD11	2:F:702:HEM:CMD	2.45	0.45
1:F:375[A]:ARG:HE	1:F:376[A]:ARG:HG3	1.81	0.45
1:C:35:ARG:HH11	6:C:525:FMT:C	2.30	0.44
4:A:503[A]:RAM:H2	6:A:508:FMT:C	2.46	0.44
1:B:89:GLU:HB3	4:B:503[A]:RAM:H1	1.98	0.44
1:B:42[B]:ARG:NH2	1:C:118:GLU:OE2	2.45	0.44
1:C:92:GLY:HA3	4:C:503[A]:RAM:O3	2.18	0.44

Continued on next page...

CONFIDENTIAL VALIDATION REPORT

Continued from previous page...

Atom-1	Atom-2	Interatomic distance (Å)	Clash overlap (Å)
1:D:283:GLU:HG3	1:D:337:LEU:CD2	2.47	0.44
1:F:125[B]:ARG:HG2	1:F:368:GLU:OE1	2.16	0.44
1:A:107[A]:LEU:CD1	1:A:234:ILE:HG12	2.47	0.44
1:B:121:ARG:HG3	1:B:364:LEU:HD11	2.00	0.44
3:C:502:DEB:O21	4:C:503[A]:RAM:O1	2.25	0.44
1:E:333[A]:HIS:HD2	1:E:336[A]:GLU:CB	2.28	0.44
1:B:258:HIS:CE1	1:B:262:THR:HG21	2.53	0.44
1:C:178[B]:MET:HE1	1:C:193[B]:GLN:HG3	1.99	0.44
1:F:375[A]:ARG:HH11	1:F:376[A]:ARG:CD	2.30	0.44
1:D:178[B]:MET:HE1	1:D:193[B]:GLN:CB	2.43	0.44
1:D:62:ARG:HH11	1:D:351[B]:HIS:CE1	2.34	0.44
1:A:152:PHE:HB3	1:A:153:PRO:HD3	2.00	0.44
1:A:244:ALA:HB1	2:A:501:HEM:C4C	2.53	0.44
1:D:159:GLU:HG3	6:D:514:FMT:C	2.47	0.44
1:E:215:LEU:HD23	1:E:215:LEU:HA	1.69	0.44
1:D:89:GLU:HB2	1:D:193[A]:GLN:NE2	2.32	0.44
1:A:207:ARG:HB2	1:A:220:ALA:CB	2.48	0.44
3:A:502:DEB:H203	4:A:503[A]:RAM:O4	2.18	0.44
1:F:121:ARG:HG3	1:F:364:LEU:HD11	1.99	0.44
1:C:69:ARG:HG2	1:C:302[A]:GLU:HG3	2.00	0.43
1:E:57[A]:ARG:NH1	1:E:329[A]:GLU:HB2	2.33	0.43
1:E:271:VAL:HA	1:E:374:VAL:HG13	2.00	0.43
1:E:101[B]:HIS:NE2	1:E:354[B]:HIS:CD2	2.86	0.43
1:F:42:ARG:NH2	9:F:814:HOH:O	2.50	0.43
1:B:200:MET:O	1:B:204[A]:VAL:HG13	2.18	0.43
1:E:35[A]:ARG:HG2	1:E:57[A]:ARG:HG3	1.99	0.43
1:F:168[B]:ARG:HG2	1:F:172:ARG:HG3	2.00	0.43
1:F:375[A]:ARG:NH1	1:F:376[A]:ARG:HG3	2.31	0.43
1:B:392:LYS:HG2	1:B:401:GLU:HG3	2.01	0.43
1:D:101[A]:HIS:CD2	9:D:612:HOH:O	2.67	0.43
1:D:178[B]:MET:CE	1:D:193[B]:GLN:CB	2.95	0.43
1:B:13:ALA:O	1:C:115[A]:ARG:NE	2.49	0.43
1:D:236:ASN:OD1	4:D:503:RAM:C3	2.67	0.43
1:A:183[B]:ARG:HB2	1:A:394:GLY:O	2.19	0.43
1:A:45[A]:LEU:HD12	1:A:45[A]:LEU:HA	1.87	0.43
1:B:309:VAL:HG13	1:C:122:PRO:HA	1.99	0.43
2:E:501:HEM:CMC	2:E:501:HEM:HBC2	2.48	0.43
1:F:197:MET:SD	1:F:236[B]:ASN:ND2	2.91	0.43
1:F:216:LEU:HA	1:F:216:LEU:HD12	1.90	0.43
1:B:270:LEU:CB	1:B:374[A]:VAL:HG21	2.49	0.43
1:D:280:ALA:HA	1:D:339:PHE:CD1	2.54	0.43

Continued on next page...

CONFIDENTIAL VALIDATION REPORT

Continued from previous page...

Atom-1	Atom-2	Interatomic distance (Å)	Clash overlap (Å)
1:E:115[A]:ARG:HB2	1:E:115[A]:ARG:HE	1.56	0.43
1:F:115:ARG:HB2	6:F:706:FMT:C	2.49	0.43
1:B:178[A]:MET:HE1	1:B:193[A]:GLN:HG2	2.01	0.42
1:C:107:LEU:HD12	1:C:107:LEU:HA	1.87	0.42
1:C:86[A]:THR:HG22	1:C:186:ALA:O	2.19	0.42
1:D:290:LEU:O	1:D:397:ILE:HA	2.18	0.42
1:D:46:PRO:HB2	1:D:47:TYR:CD2	2.54	0.42
1:E:93:VAL:HG13	1:E:237[A]:MET:SD	2.59	0.42
1:F:161:LEU:O	1:F:216:LEU:HB2	2.19	0.42
4:B:503[A]:RAM:O1	6:B:513:FMT:C	2.66	0.42
1:D:70:PHE:CE2	1:D:304:VAL:HG11	2.54	0.42
1:B:260:LEU:HD11	1:B:370[B]:LEU:HD21	2.02	0.42
1:E:39:PRO:HG2	1:E:308:THR:OG1	2.18	0.42
1:E:327:ASP:HB3	1:E:330:VAL:HG13	2.01	0.42
1:A:271[B]:VAL:HG21	1:A:378:PRO:HB3	2.02	0.42
1:B:110[A]:LYS:H	1:B:110[A]:LYS:HD3	1.85	0.42
1:D:107[A]:LEU:HD21	1:D:222:ALA:HB1	2.01	0.42
1:A:63[B]:ILE:HA	1:A:63[B]:ILE:HD13	1.85	0.42
1:B:328:GLU:HG2	6:B:519:FMT:H	2.00	0.42
1:B:92:GLY:O	1:B:96:GLN:HG2	2.20	0.42
1:D:178[B]:MET:HE2	1:D:193[B]:GLN:HG3	2.00	0.42
1:E:290:LEU:O	1:E:397:ILE:HA	2.19	0.42
1:B:10:PRO:O	1:B:10:PRO:HG2	2.20	0.42
1:D:101[B]:HIS:CD2	1:D:354:HIS:CE1	3.08	0.42
1:D:283:GLU:OE2	1:D:338:ASP:N	2.38	0.42
1:D:236:ASN:CG	4:D:503:RAM:HO3	2.14	0.42
1:E:207:ARG:HA	1:E:210:ALA:HB3	2.00	0.42
1:A:107[A]:LEU:HD11	1:A:234:ILE:HG12	2.01	0.42
1:B:108[A]:VAL:HG22	1:B:215:LEU:CD2	2.50	0.42
1:B:263:GLU:HB2	1:B:266:ARG:HD2	2.01	0.42
1:B:326[A]:ARG:HH11	1:B:326[A]:ARG:CG	2.28	0.42
1:D:19[B]:PHE:CE1	1:D:30[B]:HIS:HD2	2.37	0.42
1:B:209:ASP:HB2	1:F:302:GLU:HG3	2.01	0.42
1:B:6[A]:THR:OG1	6:B:526:FMT:O2	2.30	0.42
1:E:31:TYR:CE1	1:E:320:HIS:CD2	3.08	0.42
1:B:42[B]:ARG:HH21	1:C:118:GLU:CD	2.22	0.42
1:E:355:HIS:HE2	6:E:504:FMT:C	2.33	0.42
1:A:130:SER:O	1:A:133:ASP:HB2	2.20	0.41
1:A:328[A]:GLU:HG2	6:A:514:FMT:H	2.02	0.41
6:B:538:FMT:O1	1:F:106:ARG:NH1	2.47	0.41
1:D:107[A]:LEU:HA	1:D:107[A]:LEU:HD12	1.83	0.41

Continued on next page...

CONFIDENTIAL VALIDATION REPORT

Continued from previous page...

Atom-1	Atom-2	Interatomic distance (Å)	Clash overlap (Å)
1:A:240:SER:OG	4:A:503[A]:RAM:H61	2.20	0.41
1:A:45[B]:LEU:HD12	1:A:81:PRO:CB	2.50	0.41
1:C:207[B]:ARG:HD3	1:C:212:THR:OG1	2.21	0.41
4:C:503[A]:RAM:H5	8:C:507:GOL:O2	2.20	0.41
1:F:270[B]:LEU:HD21	1:F:277:VAL:CG2	2.50	0.41
1:B:152:PHE:HB3	1:B:153:PRO:HD3	2.01	0.41
6:B:538:FMT:C	1:F:106:ARG:NH1	2.83	0.41
8:C:507:GOL:O3	6:C:555[B]:FMT:O2	2.37	0.41
1:D:270:LEU:CB	1:D:374:VAL:HG21	2.50	0.41
1:E:157:ILE:HG13	1:E:161:LEU:HD22	2.02	0.41
1:A:337[A]:LEU:HA	1:A:337[A]:LEU:HD22	1.88	0.41
3:A:502:DEB:H5	4:A:503[A]:RAM:H5	2.02	0.41
1:B:133:ASP:O	1:B:136:VAL:CG2	2.68	0.41
1:D:115[B]:ARG:HD2	9:D:614:HOH:O	2.20	0.41
1:D:58[A]:MET:HE2	1:D:62:ARG:HG3	2.01	0.41
1:F:259:LEU:HB2	1:F:284:MET:HE2	2.01	0.41
1:B:191[A]:ARG:NH1	1:B:195:ASP:OD1	2.53	0.41
4:B:503[A]:RAM:O1	6:B:513:FMT:O1	2.38	0.41
1:D:168[A]:ARG:HA	1:D:171:PHE:CZ	2.56	0.41
1:F:150:VAL:HB	1:F:151:PRO:HD3	2.02	0.41
1:A:290:LEU:O	1:A:397:ILE:HA	2.21	0.41
1:A:271[B]:VAL:HA	1:A:374:VAL:HG13	2.03	0.41
1:F:241:LEU:HD21	1:F:357[B]:ILE:HD13	2.03	0.41
1:F:267[A]:TYR:OH	1:F:374[A]:VAL:HA	2.21	0.41
1:A:199:TYR:CZ	1:A:203:LEU:HD11	2.56	0.41
1:C:14:VAL:O	1:C:44[B]:ARG:NE	2.53	0.41
1:C:168:ARG:NH1	9:C:610:HOH:O	2.34	0.41
1:D:180:SER:HB2	1:D:183:ARG:HB2	2.03	0.41
1:B:10:PRO:HB3	1:B:13:ALA:HB2	1.86	0.41
1:B:237:MET:HE2	1:B:241:LEU:HD11	2.02	0.41
1:C:276:LEU:HD12	1:C:276:LEU:HA	1.95	0.41
1:C:42[A]:ARG:HH22	1:C:316:PRO:HD2	1.84	0.41
1:D:101[A]:HIS:NE2	1:D:353:ALA:O	2.50	0.41
1:F:157:ILE:HA	1:F:157:ILE:HD12	1.91	0.41
1:F:183[A]:ARG:NH2	9:F:805:HOH:O	2.51	0.41
1:E:21:LEU:HA	1:E:21:LEU:HD12	1.94	0.41
1:F:132:LEU:HD12	1:F:132:LEU:HA	1.90	0.41
1:F:87:PRO:HA	1:F:88:PRO:HD3	1.93	0.41
6:A:511:FMT:O1	9:A:604:HOH:O	2.22	0.41
1:D:70:PHE:HE2	1:D:304:VAL:HG11	1.86	0.41
1:E:35[A]:ARG:HD3	1:E:56:THR:O	2.20	0.41

Continued on next page...

CONFIDENTIAL VALIDATION REPORT

Continued from previous page...

Atom-1	Atom-2	Interatomic distance (Å)	Clash overlap (Å)
6:A:508:FMT:O1	6:A:509:FMT:O2	2.39	0.41
1:B:178[B]:MET:CE	1:B:193[B]:GLN:HB2	2.51	0.41
1:D:106[A]:ARG:CG	1:D:106[A]:ARG:HH11	2.34	0.41
2:F:702:HEM:CBB	2:F:702:HEM:HMB2	2.36	0.41
1:C:178[A]:MET:SD	4:C:503[A]:RAM:C6	3.09	0.40
1:B:290:LEU:O	1:B:397:ILE:HA	2.21	0.40
1:E:133:ASP:OD1	1:E:376:ARG:NH2	2.43	0.40
1:F:259:LEU:HB2	1:F:284:MET:CE	2.51	0.40
1:B:36:ARG:NH1	1:B:37:ASP:OD2	2.54	0.40
1:B:6[A]:THR:HG21	1:C:355:HIS:CE1	2.56	0.40
1:C:240:SER:OG	4:C:503[A]:RAM:O2	2.39	0.40
1:E:101[B]:HIS:CD2	1:E:354[B]:HIS:CD2	3.09	0.40
2:B:501:HEM:HMC2	2:B:501:HEM:HBC2	2.03	0.40
1:B:328:GLU:CB	6:B:519:FMT:C	2.98	0.40
3:E:502:DEB:C20	4:E:503[A]:RAM:H63	2.48	0.40
1:F:194[A]:GLN:NE2	9:F:816:HOH:O	2.54	0.40
1:B:178[B]:MET:HE1	1:B:193[B]:GLN:HB2	2.02	0.40
8:C:507:GOL:C3	6:C:555[B]:FMT:O1	2.66	0.40
1:F:270[B]:LEU:HD11	1:F:277:VAL:HG22	2.03	0.40

All (1) symmetry-related close contacts are listed below. The label for Atom-2 includes the symmetry operator and encoded unit-cell translations to be applied.

Atom-1	Atom-2	Interatomic distance (Å)	Clash overlap (Å)
1:C:103[A]:ARG:NH1	1:E:169:ASP:OD2[4_546]	2.14	0.06

5.3 Torsion angles (i)

5.3.1 Protein backbone (i)

In the following table, the Percentiles column shows the percent Ramachandran outliers of the chain as a percentile score with respect to all X-ray entries followed by that with respect to entries of similar resolution.

The Analysed column shows the number of residues for which the backbone conformation was analysed, and the total number of residues.

Mol	Chain	Analysed	Favoured	Allowed	Outliers	Percentiles	
1	A	455/403 (113%)	440 (97%)	15 (3%)	0	100	100

Continued on next page...

CONFIDENTIAL VALIDATION REPORT

Continued from previous page...

Mol	Chain	Analysed	Favoured	Allowed	Outliers	Percentiles	
1	B	438/403 (109%)	425 (97%)	13 (3%)	0	100	100
1	C	434/403 (108%)	428 (99%)	6 (1%)	0	100	100
1	D	447/403 (111%)	428 (96%)	19 (4%)	0	100	100
1	E	435/403 (108%)	413 (95%)	22 (5%)	0	100	100
1	F	443/403 (110%)	424 (96%)	19 (4%)	0	100	100
All	All	2652/2418 (110%)	2558 (96%)	94 (4%)	0	100	100

There are no Ramachandran outliers to report.

5.3.2 Protein sidechains ⓘ

In the following table, the Percentiles column shows the percent sidechain outliers of the chain as a percentile score with respect to all X-ray entries followed by that with respect to entries of similar resolution.

The Analysed column shows the number of residues for which the sidechain conformation was analysed, and the total number of residues.

Mol	Chain	Analysed	Rotameric	Outliers	Percentiles	
1	A	392/337 (116%)	367 (94%)	25 (6%)	17	14
1	B	374/337 (111%)	353 (94%)	21 (6%)	21	18
1	C	371/337 (110%)	354 (95%)	17 (5%)	27	25
1	D	385/337 (114%)	354 (92%)	31 (8%)	11	8
1	E	373/337 (111%)	349 (94%)	24 (6%)	17	14
1	F	380/337 (113%)	351 (92%)	29 (8%)	13	9
All	All	2275/2022 (112%)	2128 (94%)	147 (6%)	21	13

All (147) residues with a non-rotameric sidechain are listed below:

Mol	Chain	Res	Type
1	A	14[A]	VAL
1	A	14[B]	VAL
1	A	21	LEU
1	A	27	LEU
1	A	45[A]	LEU
1	A	45[B]	LEU
1	A	107[A]	LEU
1	A	107[B]	LEU

Continued on next page...

CONFIDENTIAL VALIDATION REPORT

Continued from previous page...

Mol	Chain	Res	Type
1	A	110[A]	LYS
1	A	110[B]	LYS
1	A	160[A]	LEU
1	A	160[B]	LEU
1	A	196	PHE
1	A	197[A]	MET
1	A	197[B]	MET
1	A	198	VAL
1	A	209	ASP
1	A	227[A]	ASP
1	A	227[B]	ASP
1	A	228	HIS
1	A	337[A]	LEU
1	A	337[B]	LEU
1	A	343	ARG
1	A	370[A]	LEU
1	A	370[B]	LEU
1	B	5	HIS
1	B	6[A]	THR
1	B	6[B]	THR
1	B	14	VAL
1	B	108[A]	VAL
1	B	108[B]	VAL
1	B	110[A]	LYS
1	B	110[B]	LYS
1	B	115	ARG
1	B	123	ARG
1	B	196	PHE
1	B	225[A]	ASN
1	B	225[B]	ASN
1	B	264[A]	ARG
1	B	264[B]	ARG
1	B	265[A]	LYS
1	B	265[B]	LYS
1	B	309	VAL
1	B	374[A]	VAL
1	B	374[B]	VAL
1	B	393	GLN
1	C	12	ASP
1	C	42[A]	ARG
1	C	42[B]	ARG
1	C	115[A]	ARG

Continued on next page...

CONFIDENTIAL VALIDATION REPORT

Continued from previous page...

Mol	Chain	Res	Type
1	C	115[B]	ARG
1	C	116	ARG
1	C	123	ARG
1	C	178[A]	MET
1	C	178[B]	MET
1	C	190[A]	GLN
1	C	190[B]	GLN
1	C	196	PHE
1	C	302[A]	GLU
1	C	302[B]	GLU
1	C	337	LEU
1	C	343	ARG
1	C	374	VAL
1	D	34[A]	LEU
1	D	34[B]	LEU
1	D	36[A]	ARG
1	D	36[B]	ARG
1	D	42	ARG
1	D	86	THR
1	D	105[A]	ARG
1	D	105[B]	ARG
1	D	106[A]	ARG
1	D	106[B]	ARG
1	D	127	LEU
1	D	132	LEU
1	D	159	GLU
1	D	196	PHE
1	D	207	ARG
1	D	209	ASP
1	D	224	ASP
1	D	225	ASN
1	D	227[A]	ASP
1	D	227[B]	ASP
1	D	263[A]	GLU
1	D	263[B]	GLU
1	D	309	VAL
1	D	312	ARG
1	D	341	ARG
1	D	342[A]	GLU
1	D	342[B]	GLU
1	D	343[A]	ARG
1	D	343[B]	ARG

Continued on next page...

CONFIDENTIAL VALIDATION REPORT

Continued from previous page...

Mol	Chain	Res	Type
1	D	370	LEU
1	D	374	VAL
1	E	14	VAL
1	E	21	LEU
1	E	45	LEU
1	E	93	VAL
1	E	108	VAL
1	E	115[A]	ARG
1	E	115[B]	ARG
1	E	116[A]	ARG
1	E	116[B]	ARG
1	E	154	VAL
1	E	161	LEU
1	E	196	PHE
1	E	204	VAL
1	E	207	ARG
1	E	212	THR
1	E	213	GLU
1	E	225	ASN
1	E	226	ASP
1	E	312[A]	ARG
1	E	312[B]	ARG
1	E	330	VAL
1	E	343[A]	ARG
1	E	343[B]	ARG
1	E	374	VAL
1	F	12	ASP
1	F	21[A]	LEU
1	F	21[B]	LEU
1	F	27	LEU
1	F	42	ARG
1	F	45	LEU
1	F	51	THR
1	F	93	VAL
1	F	94	LEU
1	F	115	ARG
1	F	116	ARG
1	F	132	LEU
1	F	141	PRO
1	F	193	GLN
1	F	194[A]	GLN
1	F	194[B]	GLN

Continued on next page...

CONFIDENTIAL VALIDATION REPORT

Continued from previous page...

Mol	Chain	Res	Type
1	F	196	PHE
1	F	198	VAL
1	F	204	VAL
1	F	216	LEU
1	F	225	ASN
1	F	266	ARG
1	F	269	SER
1	F	309	VAL
1	F	326	ARG
1	F	329[A]	GLU
1	F	329[B]	GLU
1	F	390[A]	LYS
1	F	390[B]	LYS

Some sidechains can be flipped to improve hydrogen bonding and reduce clashes. All (17) such sidechains are listed below:

Mol	Chain	Res	Type
1	A	258	HIS
1	A	340	HIS
1	B	5	HIS
1	B	96	GLN
1	B	258	HIS
1	B	393	GLN
1	C	30	HIS
1	C	206	GLN
1	C	228	HIS
1	C	258	HIS
1	C	393	GLN
1	D	320	HIS
1	E	193	GLN
1	E	206	GLN
1	E	225	ASN
1	E	236	ASN
1	E	320	HIS

5.3.3 RNA (i)

There are no RNA molecules in this entry.

CONFIDENTIAL VALIDATION REPORT

5.4 Non-standard residues in protein, DNA, RNA chains (i)

There are no non-standard protein/DNA/RNA residues in this entry.

5.5 Carbohydrates (i)

There are no carbohydrates in this entry.

5.6 Ligand geometry (i)

Of 238 ligands modelled in this entry, 7 are monoatomic - leaving 231 for Mogul analysis.

In the following table, the Counts columns list the number of bonds (or angles) for which Mogul statistics could be retrieved, the number of bonds (or angles) that are observed in the model and the number of bonds (or angles) that are defined in the Chemical Component Dictionary. The Link column lists molecule types, if any, to which the group is linked. The Z score for a bond length (or angle) is the number of standard deviations the observed value is removed from the expected value. A bond length (or angle) with $|Z| > 2$ is considered an outlier worth inspection. RMSZ is the root-mean-square of all Z scores of the bond lengths (or angles).

Mol	Type	Chain	Res	Link	Bond lengths			Bond angles		
					Counts	RMSZ	$\#\|Z\| > 2$	Counts	RMSZ	$\#\|Z\| > 2$
6	FMT	F	719	-	0,2,2	0.00	-	0,1,1	0.00	-
6	FMT	A	533	-	0,2,2	0.00	-	0,1,1	0.00	-
6	FMT	B	519	-	0,2,2	0.00	-	0,1,1	0.00	-
6	FMT	A	539	-	0,2,2	0.00	-	0,1,1	0.00	-
6	FMT	D	511	-	0,2,2	0.00	-	0,1,1	0.00	-
2	HEM	C	501	1	27,50,50	1.54	5 (18%)	17,82,82	1.73	4 (23%)
6	FMT	D	509	-	0,2,2	0.00	-	0,1,1	0.00	-
6	FMT	F	716	-	0,2,2	0.00	-	0,1,1	0.00	-
3	DEB	D	502	-	27,27,27	0.74	1 (3%)	35,39,39	0.74	1 (2%)
6	FMT	D	516	-	0,2,2	0.00	-	0,1,1	0.00	-
6	FMT	B	540	-	0,2,2	0.00	-	0,1,1	0.00	-
6	FMT	A	505	-	0,2,2	0.00	-	0,1,1	0.00	-
6	FMT	B	517	-	0,2,2	0.00	-	0,1,1	0.00	-
6	FMT	A	537	-	0,2,2	0.00	-	0,1,1	0.00	-
6	FMT	A	530	-	0,2,2	0.00	-	0,1,1	0.00	-
6	FMT	B	543	-	0,2,2	0.00	-	0,1,1	0.00	-
6	FMT	C	534	-	0,2,2	0.00	-	0,1,1	0.00	-
6	FMT	E	509	-	0,2,2	0.00	-	0,1,1	0.00	-
6	FMT	C	520	-	0,2,2	0.00	-	0,1,1	0.00	-
6	FMT	B	510	-	0,2,2	0.00	-	0,1,1	0.00	-
2	HEM	A	501	1	27,50,50	1.57	6 (22%)	17,82,82	1.48	3 (17%)
6	FMT	A	518	-	0,2,2	0.00	-	0,1,1	0.00	-

CONFIDENTIAL VALIDATION REPORT

Mol	Type	Chain	Res	Link	Bond lengths			Bond angles		
					Counts	RMSZ	#|Z| > 2	Counts	RMSZ	#|Z| > 2
6	FMT	C	536	-	0,2,2	0.00	-	0,1,1	0.00	-
6	FMT	A	508	-	0,2,2	0.00	-	0,1,1	0.00	-
6	FMT	B	561	-	0,2,2	0.00	-	0,1,1	0.00	-
6	FMT	B	516	-	0,2,2	0.00	-	0,1,1	0.00	-
6	FMT	D	508	-	0,2,2	0.00	-	0,1,1	0.00	-
6	FMT	D	514	-	0,2,2	0.00	-	0,1,1	0.00	-
6	FMT	E	506	-	0,2,2	0.00	-	0,1,1	0.00	-
6	FMT	B	555	-	0,2,2	0.00	-	0,1,1	0.00	-
6	FMT	E	514[B]	-	0,2,2	0.00	-	0,1,1	0.00	-
6	FMT	B	533	-	0,2,2	0.00	-	0,1,1	0.00	-
6	FMT	C	516	-	0,2,2	0.00	-	0,1,1	0.00	-
6	FMT	B	567	-	0,2,2	0.00	-	0,1,1	0.00	-
6	FMT	B	524	-	0,2,2	0.00	-	0,1,1	0.00	-
6	FMT	F	706	-	0,2,2	0.00	-	0,1,1	0.00	-
6	FMT	C	541	-	0,2,2	0.00	-	0,1,1	0.00	-
6	FMT	C	517	-	0,2,2	0.00	-	0,1,1	0.00	-
6	FMT	D	505	-	0,2,2	0.00	-	0,1,1	0.00	-
6	FMT	A	538	-	0,2,2	0.00	-	0,1,1	0.00	-
6	FMT	E	508	-	0,2,2	0.00	-	0,1,1	0.00	-
6	FMT	C	509	-	0,2,2	0.00	-	0,1,1	0.00	-
6	FMT	B	563	-	0,2,2	0.00	-	0,1,1	0.00	-
6	FMT	A	523	-	0,2,2	0.00	-	0,1,1	0.00	-
6	FMT	A	545[B]	-	0,2,2	0.00	-	0,1,1	0.00	-
6	FMT	C	533	-	0,2,2	0.00	-	0,1,1	0.00	-
6	FMT	B	527	-	0,2,2	0.00	-	0,1,1	0.00	-
6	FMT	C	542	-	0,2,2	0.00	-	0,1,1	0.00	-
6	FMT	A	510	-	0,2,2	0.00	-	0,1,1	0.00	-
6	FMT	F	707	-	0,2,2	0.00	-	0,1,1	0.00	-
6	FMT	B	553	-	0,2,2	0.00	-	0,1,1	0.00	-
3	DEB	F	703	-	27,27,27	0.67	1 (3%)	35,39,39	0.57	0
6	FMT	C	560	-	0,2,2	0.00	-	0,1,1	0.00	-
6	FMT	A	543	-	0,2,2	0.00	-	0,1,1	0.00	-
6	FMT	B	550	-	0,2,2	0.00	-	0,1,1	0.00	-
6	FMT	B	522	-	0,2,2	0.00	-	0,1,1	0.00	-
6	FMT	C	557	-	0,2,2	0.00	-	0,1,1	0.00	-
6	FMT	C	544	-	0,2,2	0.00	-	0,1,1	0.00	-
6	FMT	B	521	-	0,2,2	0.00	-	0,1,1	0.00	-
6	FMT	C	556	-	0,2,2	0.00	-	0,1,1	0.00	-
6	FMT	E	511	-	0,2,2	0.00	-	0,1,1	0.00	-
6	FMT	B	560	-	0,2,2	0.00	-	0,1,1	0.00	-
6	FMT	F	717	-	0,2,2	0.00	-	0,1,1	0.00	-
6	FMT	C	529	-	0,2,2	0.00	-	0,1,1	0.00	-
6	FMT	B	537	-	0,2,2	0.00	-	0,1,1	0.00	-

CONFIDENTIAL VALIDATION REPORT

Mol	Type	Chain	Res	Link	Bond lengths			Bond angles		
					Counts	RMSZ	#|Z| > 2	Counts	RMSZ	#|Z| > 2
6	FMT	A	517	-	0,2,2	0.00	-	0,1,1	0.00	-
6	FMT	B	549	-	0,2,2	0.00	-	0,1,1	0.00	-
6	FMT	C	532	-	0,2,2	0.00	-	0,1,1	0.00	-
6	FMT	B	509	-	0,2,2	0.00	-	0,1,1	0.00	-
6	FMT	E	504	-	0,2,2	0.00	-	0,1,1	0.00	-
6	FMT	D	512	-	0,2,2	0.00	-	0,1,1	0.00	-
6	FMT	F	718	-	0,2,2	0.00	-	0,1,1	0.00	-
8	GOL	F	704	-	5,5,5	0.11	0	5,5,5	0.29	0
6	FMT	C	549	-	0,2,2	0.00	-	0,1,1	0.00	-
6	FMT	B	568	-	0,2,2	0.00	-	0,1,1	0.00	-
6	FMT	B	539	-	0,2,2	0.00	-	0,1,1	0.00	-
6	FMT	A	519	-	0,2,2	0.00	-	0,1,1	0.00	-
6	FMT	C	558	-	0,2,2	0.00	-	0,1,1	0.00	-
6	FMT	B	529	-	0,2,2	0.00	-	0,1,1	0.00	-
3	DEB	E	502	-	27,27,27	0.52	0	35,39,39	0.84	0
6	FMT	A	507	-	0,2,2	0.00	-	0,1,1	0.00	-
6	FMT	F	714	-	0,2,2	0.00	-	0,1,1	0.00	-
8	GOL	B	506	-	5,5,5	0.10	0	5,5,5	0.27	0
6	FMT	B	566	-	0,2,2	0.00	-	0,1,1	0.00	-
6	FMT	B	557	-	0,2,2	0.00	-	0,1,1	0.00	-
6	FMT	C	518	-	0,2,2	0.00	-	0,1,1	0.00	-
6	FMT	B	565	-	0,2,2	0.00	-	0,1,1	0.00	-
6	FMT	B	531	-	0,2,2	0.00	-	0,1,1	0.00	-
6	FMT	A	506	-	0,2,2	0.00	-	0,1,1	0.00	-
6	FMT	E	505	-	0,2,2	0.00	-	0,1,1	0.00	-
6	FMT	A	540	-	0,2,2	0.00	-	0,1,1	0.00	-
6	FMT	B	538	-	0,2,2	0.00	-	0,1,1	0.00	-
6	FMT	B	532	-	0,2,2	0.00	-	0,1,1	0.00	-
3	DEB	B	502	-	27,27,27	0.78	1 (3%)	35,39,39	0.57	0
6	FMT	C	552	-	0,2,2	0.00	-	0,1,1	0.00	-
8	GOL	C	505	-	5,5,5	0.13	0	5,5,5	0.25	0
4	RAM	A	503[A]	-	11,11,11	0.72	0	15,16,16	1.32	1 (6%)
6	FMT	B	542	-	0,2,2	0.00	-	0,1,1	0.00	-
6	FMT	C	564	-	0,2,2	0.00	-	0,1,1	0.00	-
6	FMT	D	506	-	0,2,2	0.00	-	0,1,1	0.00	-
6	FMT	B	512	-	0,2,2	0.00	-	0,1,1	0.00	-
6	FMT	A	511	-	0,2,2	0.00	-	0,1,1	0.00	-
4	RAM	F	701	-	11,11,11	1.08	0	15,16,16	1.47	2 (13%)
6	FMT	A	516	-	0,2,2	0.00	-	0,1,1	0.00	-
6	FMT	C	512	-	0,2,2	0.00	-	0,1,1	0.00	-
8	GOL	C	506	-	5,5,5	0.10	0	5,5,5	0.20	0
6	FMT	A	542	-	0,2,2	0.00	-	0,1,1	0.00	-

CONFIDENTIAL VALIDATION REPORT

Mol	Type	Chain	Res	Link	Bond lengths			Bond angles		
					Counts	RMSZ	#|Z| > 2	Counts	RMSZ	#|Z| > 2
6	FMT	A	509	-	0,2,2	0.00	-	0,1,1	0.00	-
6	FMT	C	511	-	0,2,2	0.00	-	0,1,1	0.00	-
6	FMT	B	548	-	0,2,2	0.00	-	0,1,1	0.00	-
6	FMT	D	517	-	0,2,2	0.00	-	0,1,1	0.00	-
6	FMT	C	547	-	0,2,2	0.00	-	0,1,1	0.00	-
6	FMT	B	541	-	0,2,2	0.00	-	0,1,1	0.00	-
6	FMT	B	507	-	0,2,2	0.00	-	0,1,1	0.00	-
6	FMT	D	507	-	0,2,2	0.00	-	0,1,1	0.00	-
6	FMT	A	525	-	0,2,2	0.00	-	0,1,1	0.00	-
6	FMT	B	544	-	0,2,2	0.00	-	0,1,1	0.00	-
6	FMT	C	539	-	0,2,2	0.00	-	0,1,1	0.00	-
6	FMT	B	554	-	0,2,2	0.00	-	0,1,1	0.00	-
6	FMT	A	514	-	0,2,2	0.00	-	0,1,1	0.00	-
6	FMT	C	522	-	0,2,2	0.00	-	0,1,1	0.00	-
6	FMT	B	547	-	0,2,2	0.00	-	0,1,1	0.00	-
6	FMT	D	504	-	0,2,2	0.00	-	0,1,1	0.00	-
6	FMT	D	518	-	0,2,2	0.00	-	0,1,1	0.00	-
6	FMT	A	541	-	0,2,2	0.00	-	0,1,1	0.00	-
6	FMT	B	520	-	0,2,2	0.00	-	0,1,1	0.00	-
6	FMT	B	552	-	0,2,2	0.00	-	0,1,1	0.00	-
6	FMT	B	558	-	0,2,2	0.00	-	0,1,1	0.00	-
6	FMT	C	540	-	0,2,2	0.00	-	0,1,1	0.00	-
4	RAM	C	504	-	11,11,11	0.41	0	15,16,16	1.03	2 (13%)
5	TRS	B	504	-	7,7,7	0.27	0	9,9,9	0.52	0
6	FMT	B	523	-	0,2,2	0.00	-	0,1,1	0.00	-
6	FMT	C	550	-	0,2,2	0.00	-	0,1,1	0.00	-
6	FMT	C	565	-	0,2,2	0.00	-	0,1,1	0.00	-
6	FMT	C	561	-	0,2,2	0.00	-	0,1,1	0.00	-
6	FMT	C	530	-	0,2,2	0.00	-	0,1,1	0.00	-
6	FMT	B	556	-	0,2,2	0.00	-	0,1,1	0.00	-
6	FMT	B	551	-	0,2,2	0.00	-	0,1,1	0.00	-
6	FMT	B	513	-	0,2,2	0.00	-	0,1,1	0.00	-
6	FMT	C	537	-	0,2,2	0.00	-	0,1,1	0.00	-
6	FMT	D	510	-	0,2,2	0.00	-	0,1,1	0.00	-
6	FMT	C	554	-	0,2,2	0.00	-	0,1,1	0.00	-
4	RAM	B	503[A]	-	11,11,11	0.72	0	15,16,16	1.61	2 (13%)
6	FMT	A	513	-	0,2,2	0.00	-	0,1,1	0.00	-
6	FMT	B	511	-	0,2,2	0.00	-	0,1,1	0.00	-
6	FMT	A	544	-	0,2,2	0.00	-	0,1,1	0.00	-
6	FMT	C	538	-	0,2,2	0.00	-	0,1,1	0.00	-
6	FMT	A	526	-	0,2,2	0.00	-	0,1,1	0.00	-
6	FMT	C	531	-	0,2,2	0.00	-	0,1,1	0.00	-

CONFIDENTIAL VALIDATION REPORT

CONFIDENTIAL VALIDATION REPORT

Mol	Type	Chain	Res	Link	Bond lengths			Bond angles		
					Counts	RMSZ	#|Z| > 2	Counts	RMSZ	#|Z| > 2
6	FMT	D	513	-	0,2,2	0.00	-	0,1,1	0.00	-
6	FMT	A	534	-	0,2,2	0.00	-	0,1,1	0.00	-
6	FMT	C	514	-	0,2,2	0.00	-	0,1,1	0.00	-
6	FMT	A	520	-	0,2,2	0.00	-	0,1,1	0.00	-
6	FMT	C	521	-	0,2,2	0.00	-	0,1,1	0.00	-
6	FMT	B	530	-	0,2,2	0.00	-	0,1,1	0.00	-
6	FMT	C	551	-	0,2,2	0.00	-	0,1,1	0.00	-
6	FMT	B	559	-	0,2,2	0.00	-	0,1,1	0.00	-
8	GOL	C	507	-	5,5,5	0.07	0	5,5,5	0.28	0
6	FMT	C	545	-	0,2,2	0.00	-	0,1,1	0.00	-
6	FMT	A	524	-	0,2,2	0.00	-	0,1,1	0.00	-
6	FMT	B	564	-	0,2,2	0.00	-	0,1,1	0.00	-
6	FMT	B	518	-	0,2,2	0.00	-	0,1,1	0.00	-
2	HEM	B	501	1	27,50,50	1.58	5 (18%)	17,82,82	1.61	4 (23%)
6	FMT	B	514	-	0,2,2	0.00	-	0,1,1	0.00	-
6	FMT	D	519	-	0,2,2	0.00	-	0,1,1	0.00	-
6	FMT	C	548	-	0,2,2	0.00	-	0,1,1	0.00	-
4	RAM	C	503[A]	-	11,11,11	1.03	1 (9%)	15,16,16	3.49	11 (73%)
6	FMT	E	510	-	0,2,2	0.00	-	0,1,1	0.00	-
6	FMT	C	555[B]	6	0,2,2	0.00	-	0,1,1	0.00	-
6	FMT	A	532	-	0,2,2	0.00	-	0,1,1	0.00	-
6	FMT	C	543	-	0,2,2	0.00	-	0,1,1	0.00	-
6	FMT	A	531	-	0,2,2	0.00	-	0,1,1	0.00	-
6	FMT	C	508	-	0,2,2	0.00	-	0,1,1	0.00	-
6	FMT	C	524	-	0,2,2	0.00	-	0,1,1	0.00	-
6	FMT	F	711	-	0,2,2	0.00	-	0,1,1	0.00	-
6	FMT	C	553	-	0,2,2	0.00	-	0,1,1	0.00	-
6	FMT	A	527	-	0,2,2	0.00	-	0,1,1	0.00	-
6	FMT	A	529	-	0,2,2	0.00	-	0,1,1	0.00	-
6	FMT	B	546	-	0,2,2	0.00	-	0,1,1	0.00	-
6	FMT	C	510	-	0,2,2	0.00	-	0,1,1	0.00	-
3	DEB	C	502	-	27,27,27	0.82	1 (3%)	35,39,39	0.61	1 (2%)
6	FMT	F	715	-	0,2,2	0.00	-	0,1,1	0.00	-
6	FMT	C	526	-	0,2,2	0.00	-	0,1,1	0.00	-
6	FMT	B	515	-	0,2,2	0.00	-	0,1,1	0.00	-
6	FMT	F	709	-	0,2,2	0.00	-	0,1,1	0.00	-
6	FMT	B	545	-	0,2,2	0.00	-	0,1,1	0.00	-
6	FMT	F	712	-	0,2,2	0.00	-	0,1,1	0.00	-
6	FMT	B	526	-	0,2,2	0.00	-	0,1,1	0.00	-
5	TRS	A	504	-	7,7,7	0.20	0	9,9,9	0.30	0
6	FMT	A	528	-	0,2,2	0.00	-	0,1,1	0.00	-
6	FMT	C	546	-	0,2,2	0.00	-	0,1,1	0.00	-

Mol	Type	Chain	Res	Link	Bond lengths			Bond angles		
					Counts	RMSZ	#|Z| > 2	Counts	RMSZ	#|Z| > 2
6	FMT	E	507	-	0,2,2	0.00	-	0,1,1	0.00	-
6	FMT	B	534	-	0,2,2	0.00	-	0,1,1	0.00	-
6	FMT	C	519	-	0,2,2	0.00	-	0,1,1	0.00	-
6	FMT	C	563	-	0,2,2	0.00	-	0,1,1	0.00	-
6	FMT	B	569	-	0,2,2	0.00	-	0,1,1	0.00	-
6	FMT	E	513	-	0,2,2	0.00	-	0,1,1	0.00	-
6	FMT	A	521	-	0,2,2	0.00	-	0,1,1	0.00	-
6	FMT	A	536	-	0,2,2	0.00	-	0,1,1	0.00	-
6	FMT	A	522	-	0,2,2	0.00	-	0,1,1	0.00	-
4	RAM	D	503	-	11,11,11	1.17	0	15,16,16	2.82	12 (80%)
6	FMT	C	562	-	0,2,2	0.00	-	0,1,1	0.00	-
6	FMT	F	708	-	0,2,2	0.00	-	0,1,1	0.00	-
6	FMT	C	515	-	0,2,2	0.00	-	0,1,1	0.00	-
6	FMT	F	710	-	0,2,2	0.00	-	0,1,1	0.00	-
2	HEM	D	501	1	27,50,50	1.74	7 (25%)	17,82,82	1.39	3 (17%)
5	TRS	F	705	-	7,7,7	0.08	0	9,9,9	0.49	0
4	RAM	E	503[A]	-	11,11,11	0.55	0	15,16,16	1.66	4 (26%)
2	HEM	F	702	1	27,50,50	1.32	2 (7%)	17,82,82	1.26	2 (11%)
6	FMT	B	525	-	0,2,2	0.00	-	0,1,1	0.00	-
8	GOL	B	505	-	5,5,5	0.10	0	5,5,5	0.29	0
6	FMT	C	535	-	0,2,2	0.00	-	0,1,1	0.00	-
6	FMT	B	528	-	0,2,2	0.00	-	0,1,1	0.00	-
3	DEB	A	502	-	27,27,27	0.55	0	35,39,39	0.88	0
6	FMT	C	513[B]	6	0,2,2	0.00	-	0,1,1	0.00	-
6	FMT	B	535	-	0,2,2	0.00	-	0,1,1	0.00	-
6	FMT	A	512	-	0,2,2	0.00	-	0,1,1	0.00	-
6	FMT	C	523	-	0,2,2	0.00	-	0,1,1	0.00	-
6	FMT	B	536	-	0,2,2	0.00	-	0,1,1	0.00	-
6	FMT	C	527	-	0,2,2	0.00	-	0,1,1	0.00	-
6	FMT	A	515	-	0,2,2	0.00	-	0,1,1	0.00	-
6	FMT	D	515	-	0,2,2	0.00	-	0,1,1	0.00	-
2	HEM	E	501	1	27,50,50	1.45	2 (7%)	17,82,82	1.47	3 (17%)
6	FMT	B	562	-	0,2,2	0.00	-	0,1,1	0.00	-
6	FMT	C	525	-	0,2,2	0.00	-	0,1,1	0.00	-
6	FMT	C	559	-	0,2,2	0.00	-	0,1,1	0.00	-
6	FMT	B	508	-	0,2,2	0.00	-	0,1,1	0.00	-
6	FMT	E	512	-	0,2,2	0.00	-	0,1,1	0.00	-
6	FMT	A	535	-	0,2,2	0.00	-	0,1,1	0.00	-
6	FMT	C	528	-	0,2,2	0.00	-	0,1,1	0.00	-
6	FMT	F	713	-	0,2,2	0.00	-	0,1,1	0.00	-

In the following table, the Chirals column lists the number of chiral outliers, the number of chiral

CONFIDENTIAL VALIDATION REPORT

centers analysed, the number of these observed in the model and the number defined in the Chemical Component Dictionary. Similar counts are reported in the Torsion and Rings columns. '-' means no outliers of that kind were identified.

Mol	Type	Chain	Res	Link	Chirals	Torsions	Rings
3	DEB	C	502	-	-	7/50/50/50	0/1/1/1
2	HEM	C	501	1	-	0/6/54/54	-
4	RAM	C	504	-	-	-	0/1/1/1
5	TRS	B	504	-	-	2/9/9/9	-
5	TRS	A	504	-	-	3/9/9/9	-
3	DEB	D	502	-	-	6/50/50/50	0/1/1/1
8	GOL	C	506	-	-	2/4/4/4	-
3	DEB	E	502	-	-	9/50/50/50	0/1/1/1
2	HEM	E	501	1	-	0/6/54/54	-
2	HEM	A	501	1	-	0/6/54/54	-
4	RAM	D	503	-	-	-	0/1/1/1
8	GOL	C	507	-	-	2/4/4/4	-
3	DEB	B	502	-	-	8/50/50/50	0/1/1/1
2	HEM	D	501	1	-	0/6/54/54	-
5	TRS	F	705	-	-	6/9/9/9	-
4	RAM	E	503[A]	-	-	-	0/1/1/1
2	HEM	F	702	1	-	0/6/54/54	-
4	RAM	A	503[A]	-	-	-	0/1/1/1
8	GOL	C	505	-	-	0/4/4/4	-
8	GOL	B	505	-	-	2/4/4/4	-
4	RAM	F	701	-	-	-	0/1/1/1
3	DEB	A	502	-	-	10/50/50/50	0/1/1/1
2	HEM	B	501	1	-	0/6/54/54	-
4	RAM	C	503[A]	-	-	-	0/1/1/1
3	DEB	F	703	-	-	10/50/50/50	0/1/1/1
8	GOL	B	506	-	-	0/4/4/4	-
4	RAM	B	503[A]	-	-	-	0/1/1/1
8	GOL	F	704	-	-	2/4/4/4	-

All (32) bond length outliers are listed below:

Mol	Chain	Res	Type	Atoms	Z	Observed(Å)	Ideal(Å)
2	E	501	HEM	C3B-C2B	-4.83	1.33	1.40
2	D	501	HEM	C3B-C2B	-4.32	1.34	1.40
2	C	501	HEM	C3B-C2B	-4.06	1.34	1.40
2	F	702	HEM	C3B-C2B	-3.80	1.35	1.40
2	B	501	HEM	C3B-C2B	-3.70	1.35	1.40

Continued on next page...

CONFIDENTIAL VALIDATION REPORT

Continued from previous page...

Mol	Chain	Res	Type	Atoms	Z	Observed(Å)	Ideal(Å)
2	A	501	HEM	C3B-C2B	-3.55	1.35	1.40
2	B	501	HEM	C3C-C2C	-3.55	1.35	1.40
2	A	501	HEM	C3C-C2C	-3.10	1.36	1.40
2	D	501	HEM	C3C-C2C	-3.02	1.36	1.40
2	A	501	HEM	C4B-NB	-2.79	1.30	1.36
2	C	501	HEM	C3C-C2C	-2.74	1.36	1.40
2	C	501	HEM	C4B-NB	-2.63	1.30	1.36
2	B	501	HEM	C4B-NB	-2.49	1.31	1.36
4	C	503[A]	RAM	O5-C1	-2.48	1.36	1.42
2	D	501	HEM	C1C-C2C	-2.41	1.37	1.42
2	D	501	HEM	C4B-NB	-2.40	1.31	1.36
2	D	501	HEM	C1D-ND	-2.37	1.31	1.36
3	B	502	DEB	O24-C9	-2.31	1.17	1.21
2	D	501	HEM	C1A-CHA	-2.29	1.34	1.41
2	C	501	HEM	C3D-C2D	-2.27	1.30	1.37
2	D	501	HEM	C3D-C2D	-2.27	1.30	1.37
2	B	501	HEM	C3D-C2D	-2.20	1.31	1.37
2	A	501	HEM	C1C-C2C	-2.20	1.37	1.42
3	D	502	DEB	O24-C9	-2.19	1.18	1.21
2	F	702	HEM	C3C-C2C	-2.19	1.37	1.40
2	A	501	HEM	C3D-C2D	-2.19	1.31	1.37
2	B	501	HEM	C1A-CHA	-2.17	1.35	1.41
2	E	501	HEM	C3C-C2C	-2.17	1.37	1.40
3	F	703	DEB	O24-C9	-2.16	1.18	1.21
2	A	501	HEM	C1D-ND	-2.13	1.31	1.36
2	C	501	HEM	C1C-C2C	-2.12	1.37	1.42
3	C	502	DEB	O24-C9	-2.11	1.18	1.21

All (55) bond angle outliers are listed below:

Mol	Chain	Res	Type	Atoms	Z	Observed(°)	Ideal(°)
4	C	503[A]	RAM	C3-C4-C5	-8.48	96.57	109.77
4	D	503	RAM	C4-C3-C2	4.80	119.21	110.82
4	C	503[A]	RAM	O5-C1-C2	4.56	118.42	110.28
4	C	503[A]	RAM	O1-C1-O5	-4.46	96.98	110.38
4	F	701	RAM	O5-C1-C2	4.40	118.14	110.28
4	B	503[A]	RAM	C4-C3-C2	4.34	118.39	110.82
2	E	501	HEM	CBA-CAA-C2A	3.95	119.78	112.49
4	D	503	RAM	C6-C5-C4	-3.75	106.14	113.07
4	C	503[A]	RAM	O5-C5-C4	-3.71	102.86	109.52
4	D	503	RAM	O5-C1-C2	-3.60	103.85	110.28
2	C	501	HEM	CBA-CAA-C2A	3.60	119.12	112.49

Continued on next page...

CONFIDENTIAL VALIDATION REPORT

Continued from previous page...

Mol	Chain	Res	Type	Atoms	Z	Observed(°)	Ideal(°)
2	C	501	HEM	CAD-CBD-CGD	3.52	118.58	112.67
4	E	503[A]	RAM	C6-C5-C4	-3.41	106.77	113.07
4	C	503[A]	RAM	C1-C2-C3	3.36	117.28	110.31
4	C	503[A]	RAM	O4-C4-C3	3.28	117.93	110.35
2	B	501	HEM	CAD-CBD-CGD	3.24	118.10	112.67
4	D	503	RAM	C3-C4-C5	3.18	114.73	109.77
4	D	503	RAM	O4-C4-C3	3.17	117.68	110.35
4	D	503	RAM	O5-C5-C6	3.12	113.44	106.70
2	D	501	HEM	CBA-CAA-C2A	3.11	118.22	112.49
4	E	503[A]	RAM	C3-C4-C5	-3.10	104.94	109.77
3	D	502	DEB	C10-C11-C12	3.03	120.65	114.41
4	D	503	RAM	O3-C3-C2	-2.99	103.42	110.35
4	C	503[A]	RAM	C6-C5-C4	2.88	118.39	113.07
2	B	501	HEM	CBA-CAA-C2A	2.85	117.75	112.49
4	A	503[A]	RAM	C1-C2-C3	2.72	115.96	110.31
4	B	503[A]	RAM	C3-C4-C5	2.71	113.99	109.77
2	C	501	HEM	C4A-C3A-C2A	2.70	108.87	107.00
4	C	503[A]	RAM	O3-C3-C4	2.67	116.53	110.35
2	B	501	HEM	C4A-C3A-C2A	2.61	108.81	107.00
4	C	503[A]	RAM	O2-C2-C1	-2.61	103.10	109.16
3	C	502	DEB	C10-C11-C12	2.45	119.47	114.41
2	F	702	HEM	C4A-C3A-C2A	2.44	108.70	107.00
2	A	501	HEM	CBD-CAD-C3D	-2.44	107.99	112.48
2	D	501	HEM	CMC-C2C-C3C	2.42	129.21	124.68
4	D	503	RAM	O5-C5-C4	2.42	113.87	109.52
4	D	503	RAM	O3-C3-C4	-2.42	104.76	110.35
4	D	503	RAM	O1-C1-O5	2.41	117.60	110.38
4	C	503[A]	RAM	C4-C3-C2	-2.40	106.63	110.82
2	F	702	HEM	CBD-CAD-C3D	-2.40	108.06	112.48
4	E	503[A]	RAM	O5-C1-C2	-2.39	106.02	110.28
4	E	503[A]	RAM	O5-C5-C6	2.29	111.65	106.70
2	C	501	HEM	CMC-C2C-C3C	2.28	128.95	124.68
2	A	501	HEM	CBA-CAA-C2A	2.28	116.69	112.49
2	E	501	HEM	C4A-C3A-C2A	2.28	108.58	107.00
2	A	501	HEM	CAD-CBD-CGD	2.26	116.47	112.67
2	B	501	HEM	CBD-CAD-C3D	-2.23	108.37	112.48
4	C	503[A]	RAM	O4-C4-C5	2.20	114.55	109.67
4	D	503	RAM	C1-C2-C3	-2.20	105.75	110.31
2	D	501	HEM	C4A-C3A-C2A	2.11	108.46	107.00
2	E	501	HEM	C3B-C4B-NB	-2.10	106.50	109.21
4	C	504	RAM	C4-C3-C2	-2.06	107.22	110.82
4	D	503	RAM	O2-C2-C3	2.05	115.09	110.35

Continued on next page...

CONFIDENTIAL VALIDATION REPORT

Continued from previous page...

Mol	Chain	Res	Type	Atoms	Z	Observed(°)	Ideal(°)
4	C	504	RAM	C1-C2-C3	-2.03	106.11	110.31
4	F	701	RAM	O5-C5-C6	2.02	111.07	106.70

There are no chirality outliers.

All (69) torsion outliers are listed below:

Mol	Chain	Res	Type	Atoms
3	D	502	DEB	C3-C4-C5-O21
3	D	502	DEB	C20-C4-C5-O21
3	F	703	DEB	C1-C2-C3-O19
3	F	703	DEB	C18-C2-C3-C4
8	F	704	GOL	O1-C1-C2-C3
3	B	502	DEB	C3-C4-C5-O21
3	B	502	DEB	O16-C13-C14-C15
8	C	506	GOL	O1-C1-C2-C3
8	C	507	GOL	C1-C2-C3-O3
5	A	504	TRS	C2-C-C1-O1
5	A	504	TRS	N-C-C1-O1
5	F	705	TRS	C3-C-C1-O1
5	F	705	TRS	C1-C-C2-O2
5	F	705	TRS	C3-C-C2-O2
5	F	705	TRS	N-C-C2-O2
8	B	505	GOL	C1-C2-C3-O3
3	A	502	DEB	C3-C4-C5-O21
3	F	703	DEB	C3-C4-C5-O21
3	E	502	DEB	C3-C4-C5-O21
3	C	502	DEB	C3-C4-C5-O21
3	E	502	DEB	C20-C4-C5-O21
3	B	502	DEB	C20-C4-C5-O21
3	C	502	DEB	C20-C4-C5-O21
3	A	502	DEB	C20-C4-C5-O21
8	C	506	GOL	O1-C1-C2-O2
3	F	703	DEB	C18-C2-C3-O19
3	E	502	DEB	C18-C2-C3-O19
3	B	502	DEB	C18-C2-C3-O19
3	C	502	DEB	C18-C2-C3-O19
3	A	502	DEB	C18-C2-C3-O19
3	E	502	DEB	C18-C2-C3-C4
3	A	502	DEB	C18-C2-C3-C4
3	F	703	DEB	C20-C4-C5-O21
3	D	502	DEB	C3-C4-C5-C6
8	F	704	GOL	O1-C1-C2-O2

Continued on next page...

CONFIDENTIAL VALIDATION REPORT

Continued from previous page...

Mol	Chain	Res	Type	Atoms
8	C	507	GOL	O2-C2-C3-O3
8	B	505	GOL	O2-C2-C3-O3
3	D	502	DEB	C20-C4-C5-C6
3	E	502	DEB	C20-C4-C5-C6
3	C	502	DEB	C3-C4-C5-C6
5	A	504	TRS	C3-C-C1-O1
5	F	705	TRS	C2-C-C1-O1
3	D	502	DEB	C18-C2-C3-C4
3	B	502	DEB	C18-C2-C3-C4
3	C	502	DEB	C18-C2-C3-C4
3	B	502	DEB	C3-C4-C5-C6
3	A	502	DEB	C3-C4-C5-C6
3	B	502	DEB	C20-C4-C5-C6
3	C	502	DEB	C20-C4-C5-C6
3	A	502	DEB	C20-C4-C5-C6
3	D	502	DEB	C18-C2-C3-O19
3	F	703	DEB	C20-C4-C5-C6
3	E	502	DEB	C3-C4-C5-C6
3	F	703	DEB	C3-C4-C5-C6
3	B	502	DEB	C12-C13-C14-C15
3	F	703	DEB	C1-C2-C3-C4
3	E	502	DEB	C1-C2-C3-O19
3	A	502	DEB	C1-C2-C3-C4
3	A	502	DEB	C1-C2-C3-O19
3	F	703	DEB	C23-C8-C9-O24
3	E	502	DEB	C23-C8-C9-O24
3	A	502	DEB	C23-C8-C9-O24
5	B	504	TRS	C1-C-C2-O2
5	B	504	TRS	C2-C-C3-O3
5	F	705	TRS	N-C-C1-O1
3	C	502	DEB	C23-C8-C9-O24
3	F	703	DEB	C7-C8-C9-O24
3	E	502	DEB	C7-C8-C9-O24
3	A	502	DEB	C7-C8-C9-O24

There are no ring outliers.

53 monomers are involved in 139 short contacts:

Mol	Chain	Res	Type	Clashes	Symm-Clashes
6	B	519	FMT	5	0
2	C	501	HEM	1	0
3	D	502	DEB	6	0

Continued on next page...

CONFIDENTIAL VALIDATION REPORT

Continued from previous page...

Mol	Chain	Res	Type	Clashes	Symm-Clashes
6	B	540	FMT	2	0
6	A	505	FMT	1	0
2	A	501	HEM	5	0
6	A	508	FMT	2	0
6	D	514	FMT	1	0
6	E	506	FMT	1	0
6	E	514[B]	FMT	2	0
6	F	706	FMT	1	0
6	A	543	FMT	1	0
6	C	532	FMT	1	0
6	E	504	FMT	2	0
8	F	704	GOL	1	0
6	C	558	FMT	1	0
3	E	502	DEB	4	0
6	B	565	FMT	2	0
6	B	538	FMT	2	0
8	C	505	GOL	2	0
4	A	503[A]	RAM	15	0
6	A	511	FMT	1	0
8	C	506	GOL	1	0
6	A	542	FMT	2	0
6	A	509	FMT	1	0
6	B	548	FMT	1	0
6	B	507	FMT	1	0
6	A	514	FMT	2	0
4	C	504	RAM	4	0
6	B	556	FMT	2	0
6	B	513	FMT	2	0
4	B	503[A]	RAM	7	0
6	C	538	FMT	1	0
8	C	507	GOL	11	0
2	B	501	HEM	2	0
4	C	503[A]	RAM	6	0
6	C	555[B]	FMT	4	0
6	C	543	FMT	1	0
3	C	502	DEB	1	0
6	B	526	FMT	1	0
4	D	503	RAM	11	0
2	D	501	HEM	5	0
5	F	705	TRS	3	0
4	E	503[A]	RAM	10	0
2	F	702	HEM	7	0

Continued on next page...

CONFIDENTIAL VALIDATION REPORT

Continued from previous page...

Mol	Chain	Res	Type	Clashes	Symm-Clashes
8	B	505	GOL	4	0
3	A	502	DEB	3	0
6	C	513[B]	FMT	2	0
6	A	512	FMT	1	0
6	A	515	FMT	1	0
2	E	501	HEM	7	0
6	C	525	FMT	1	0
6	B	508	FMT	1	0

The following is a two-dimensional graphical depiction of Mogul quality analysis of bond lengths, bond angles, torsion angles, and ring geometry for all instances of the Ligand of Interest. In addition, ligands with molecular weight > 250 and outliers as shown on the validation Tables will also be included. For torsion angles, if less then 5% of the Mogul distribution of torsion angles is within 10 degrees of the torsion angle in question, then that torsion angle is considered an outlier. Any bond that is central to one or more torsion angles identified as an outlier by Mogul will be highlighted in the graph. For rings, the root-mean-square deviation (RMSD) between the ring in question and similar rings identified by Mogul is calculated over all ring torsion angles. If the average RMSD is greater than 60 degrees and the minimal RMSD between the ring in question and any Mogul-identified rings is also greater than 60 degrees, then that ring is considered an outlier. The outliers are highlighted in purple. The color gray indicates Mogul did not find sufficient equivalents in the CSD to analyse the geometry.

CONFIDENTIAL VALIDATION REPORT

Ligand HEM C 501

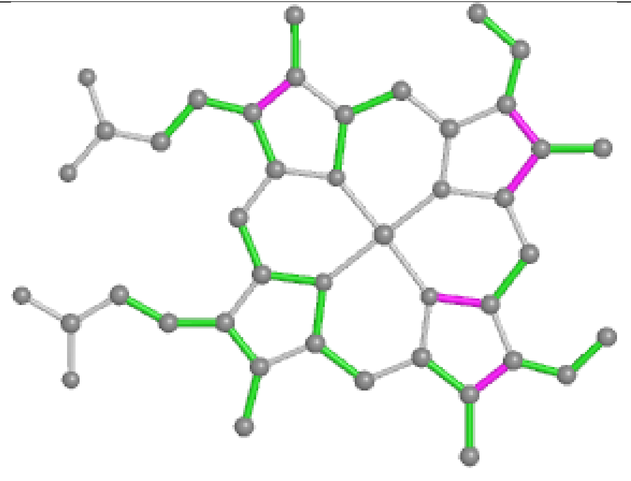

Bond lengths

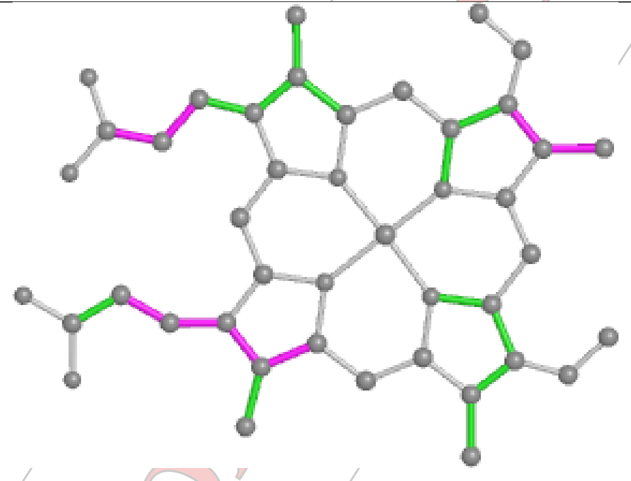

Bond angles

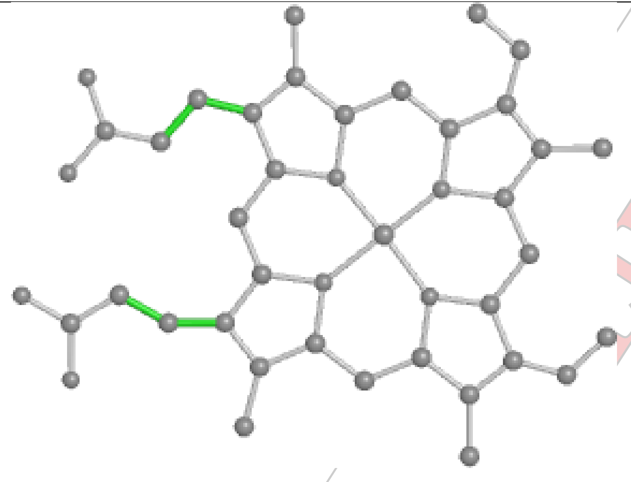

Torsions

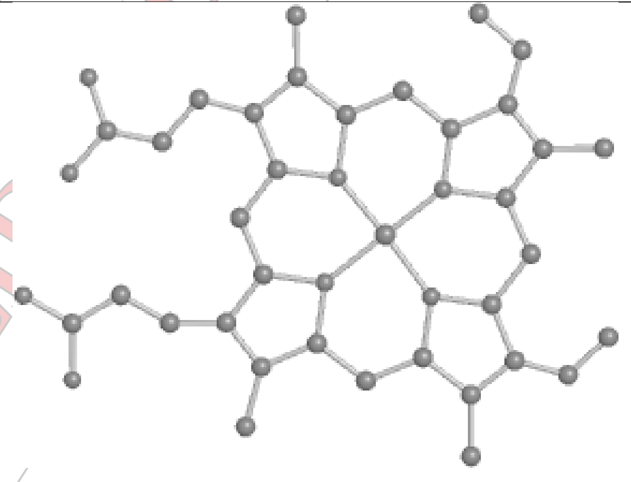

Rings

CONFIDENTIAL

Ligand DEB D 502

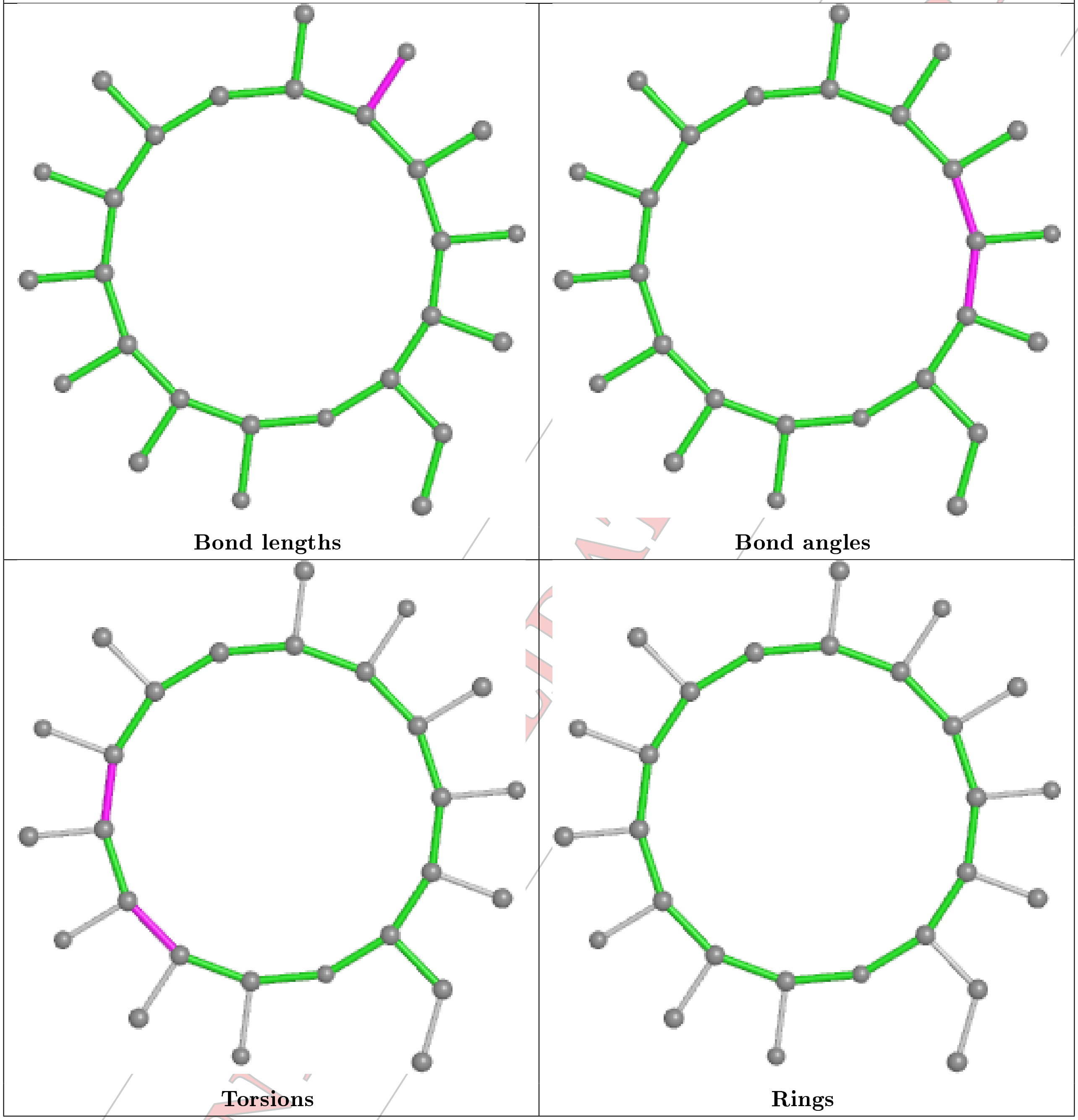

Ligand HEM A 501

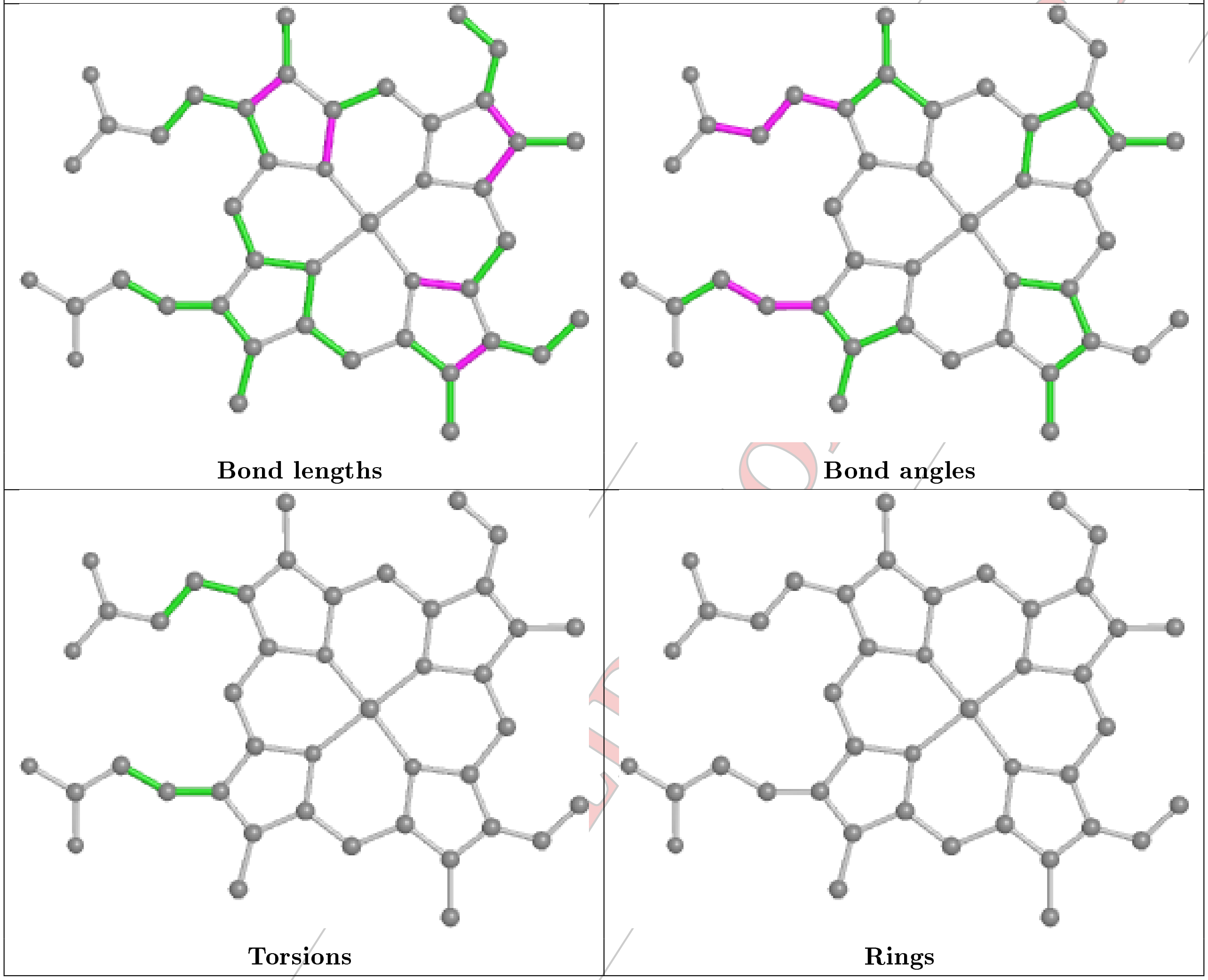

CONFIDENTIAL

Ligand DEB F 703

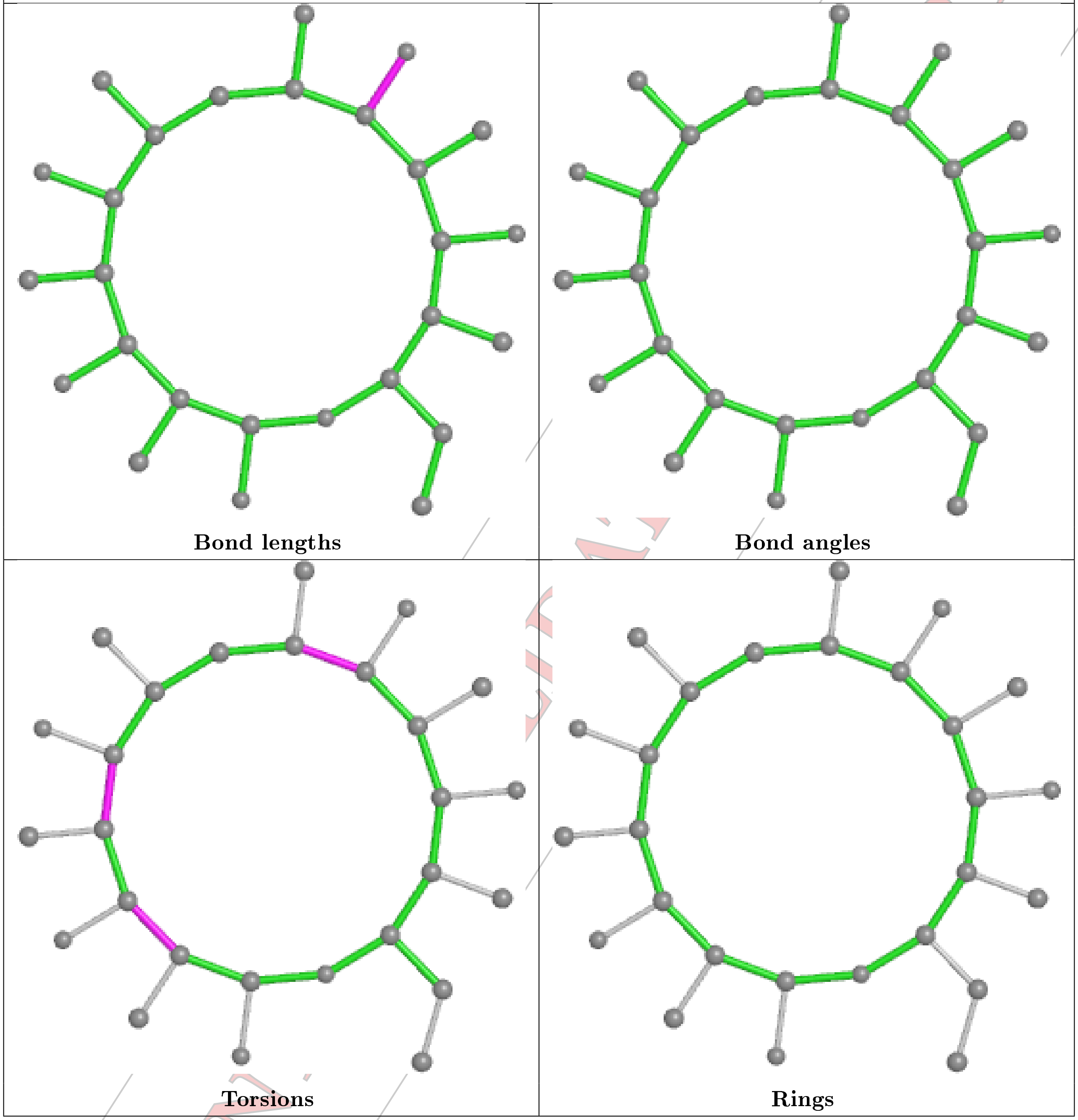

Bond lengths | Bond angles | Torsions | Rings

Ligand DEB E 502

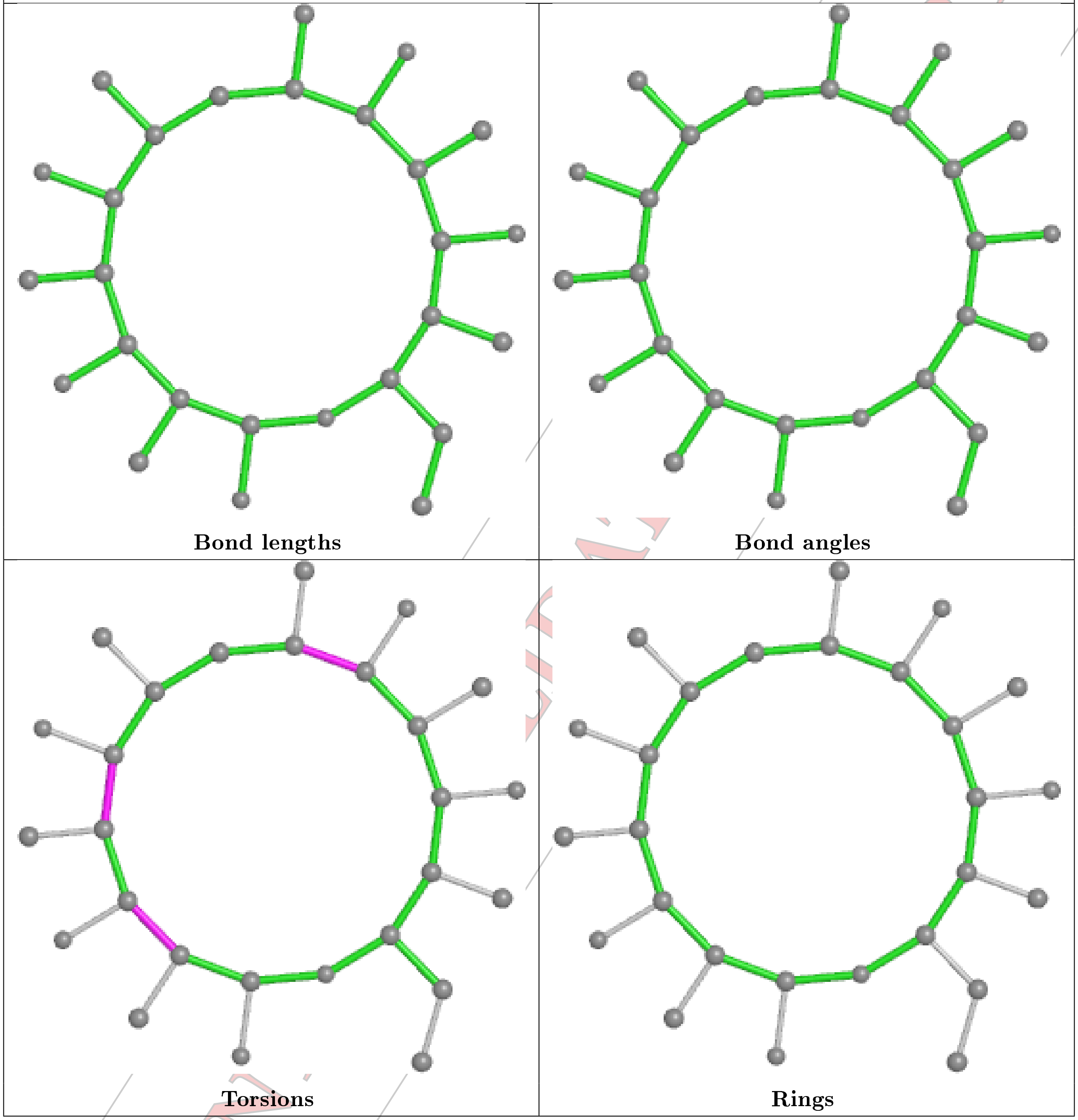

Ligand DEB B 502

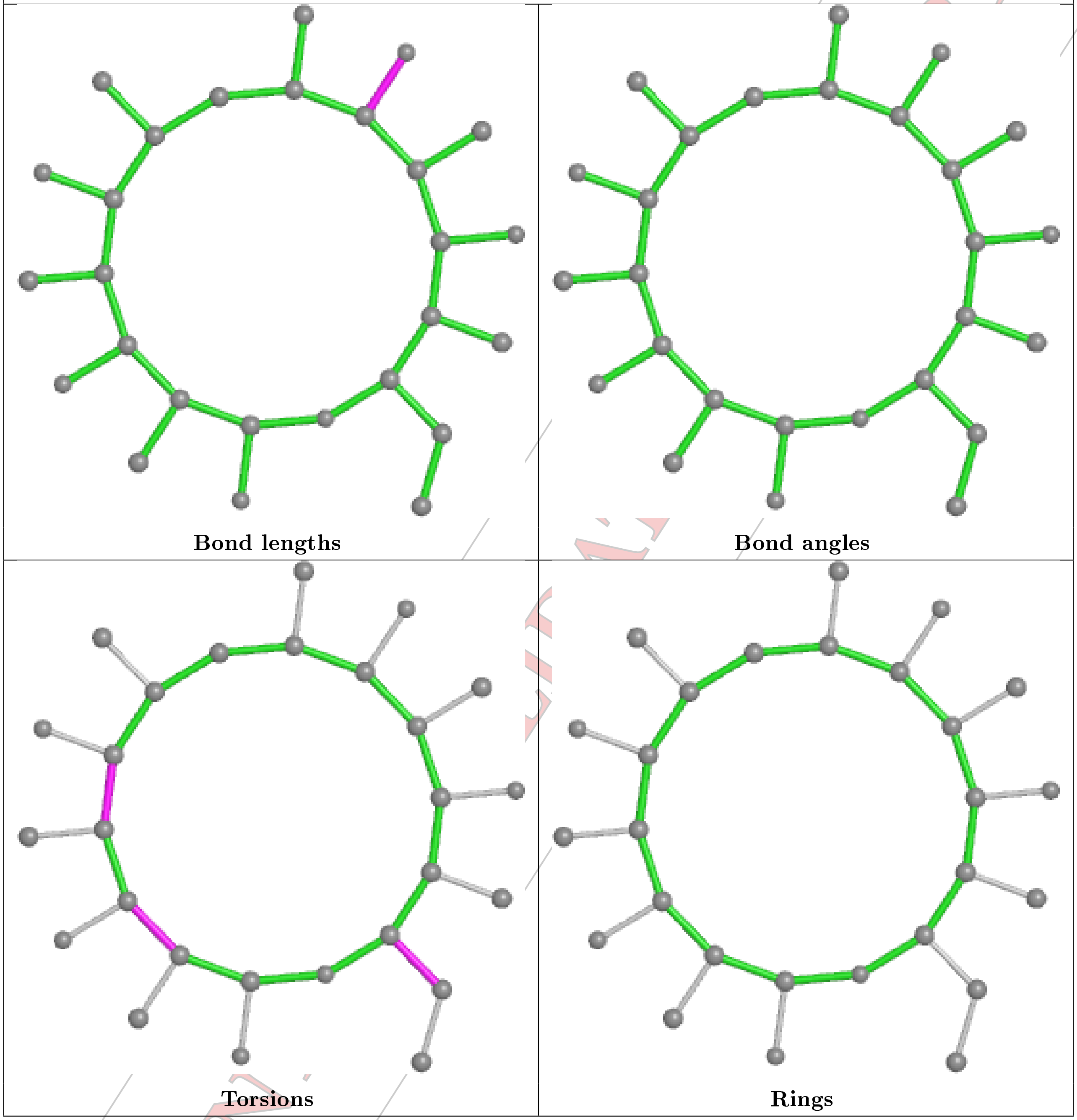

CONFIDENTIAL - DO NOT DISTRIBUTE

Ligand HEM B 501

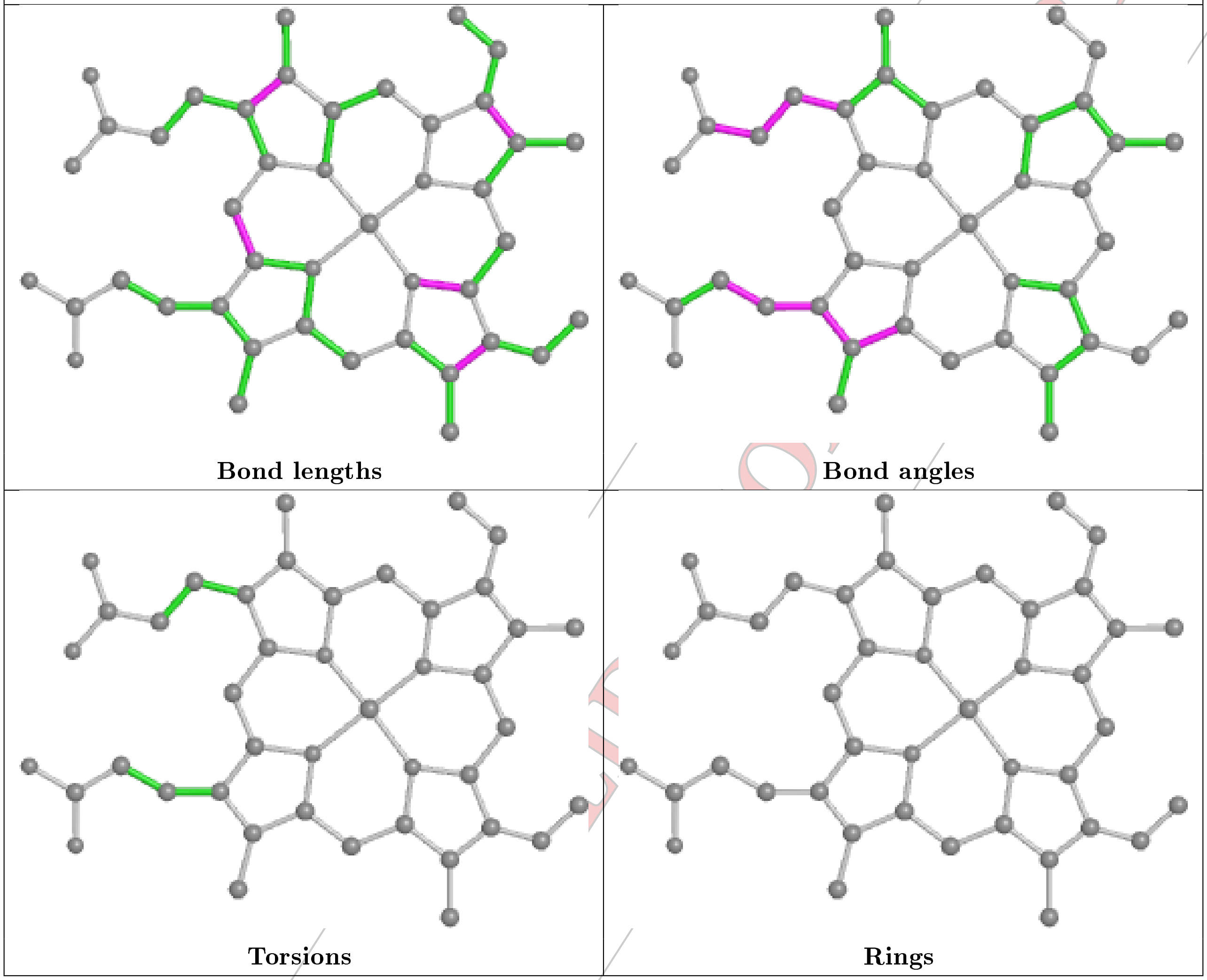

CONFIDENTIAL

Ligand DEB C 502

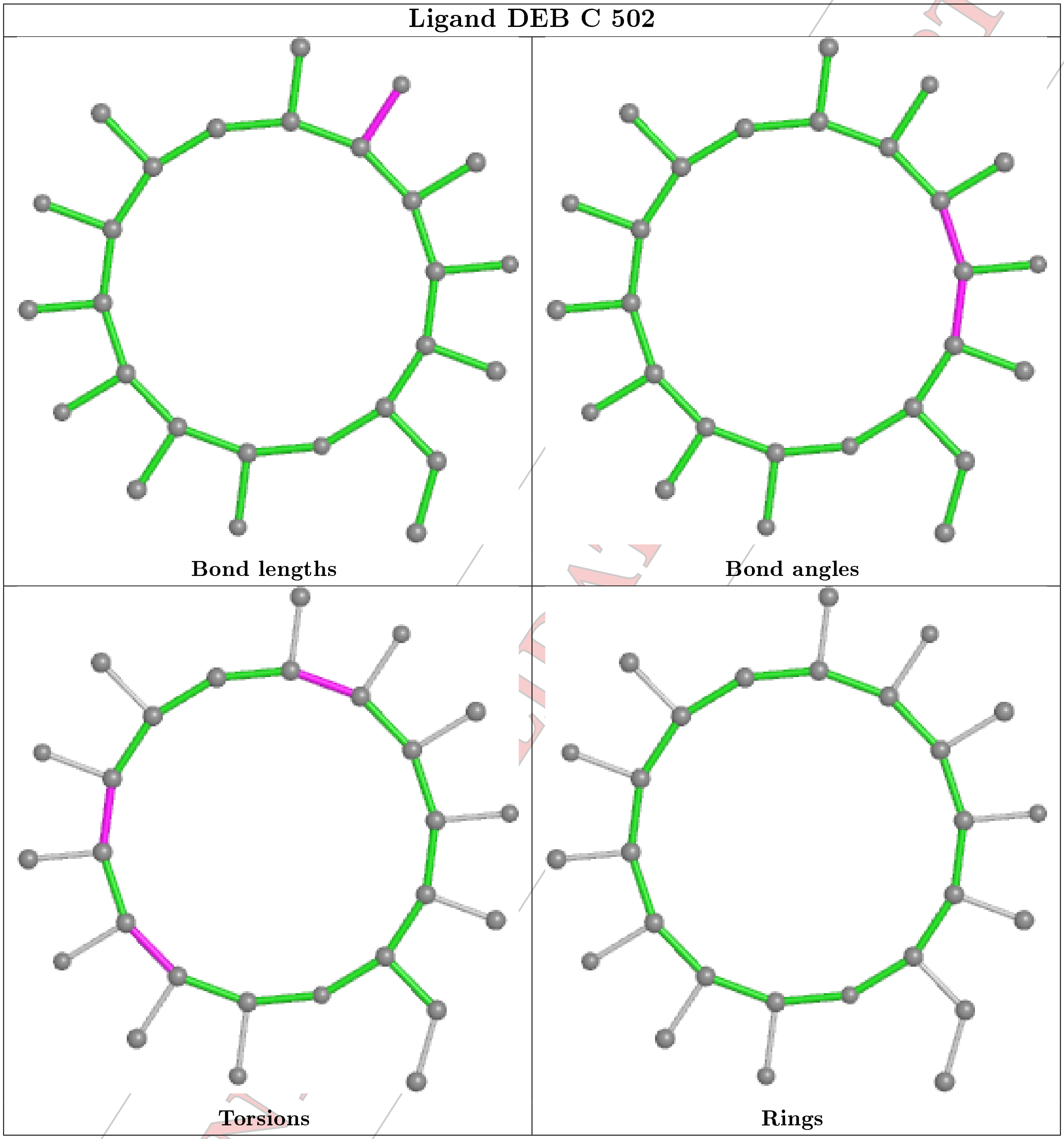

CONFIDENTIAL MANUSCRIPT

Ligand HEM D 501

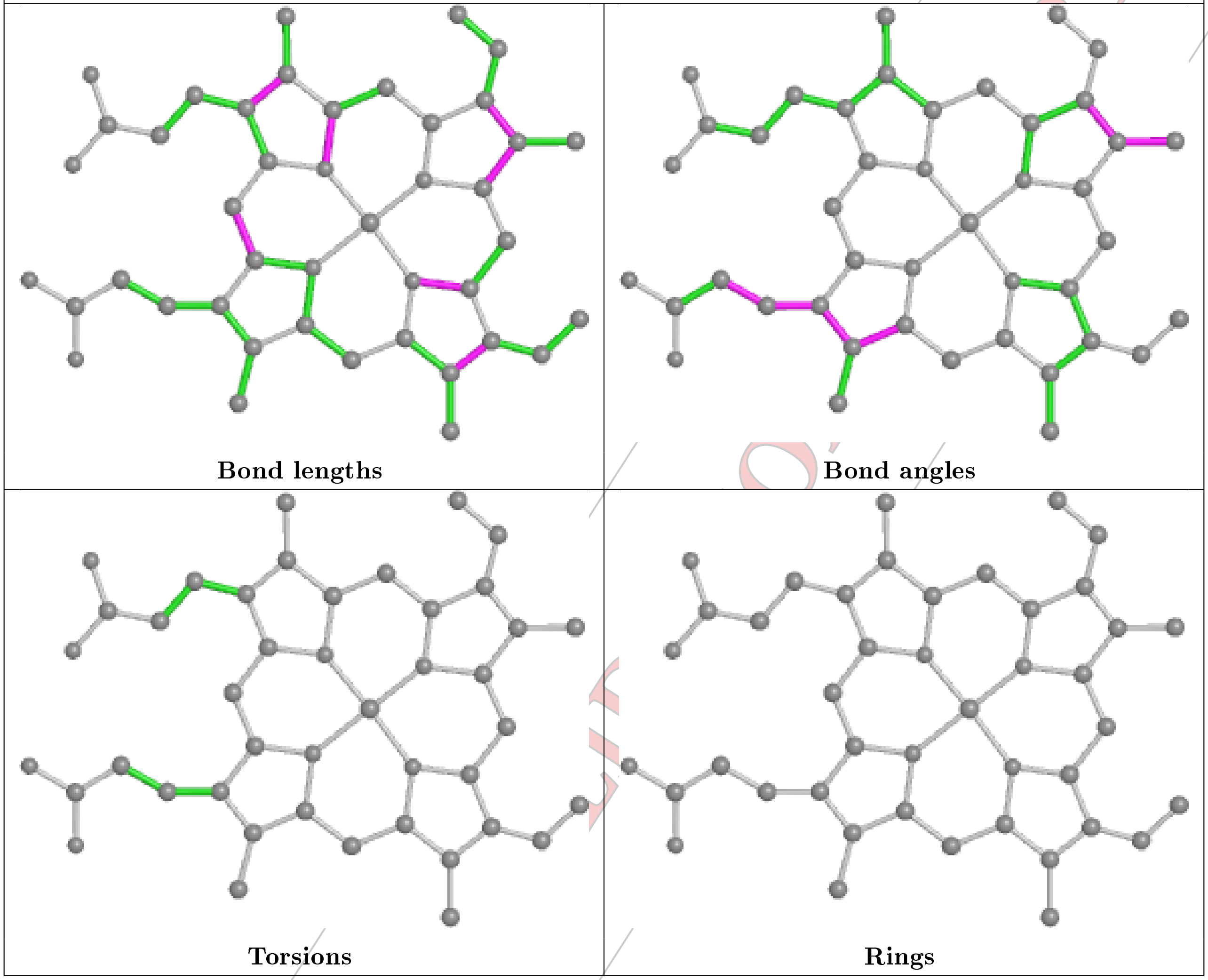

CONFIDENTIAL

Ligand HEM F 702	
Bond lengths	Bond angles
Torsions	Rings

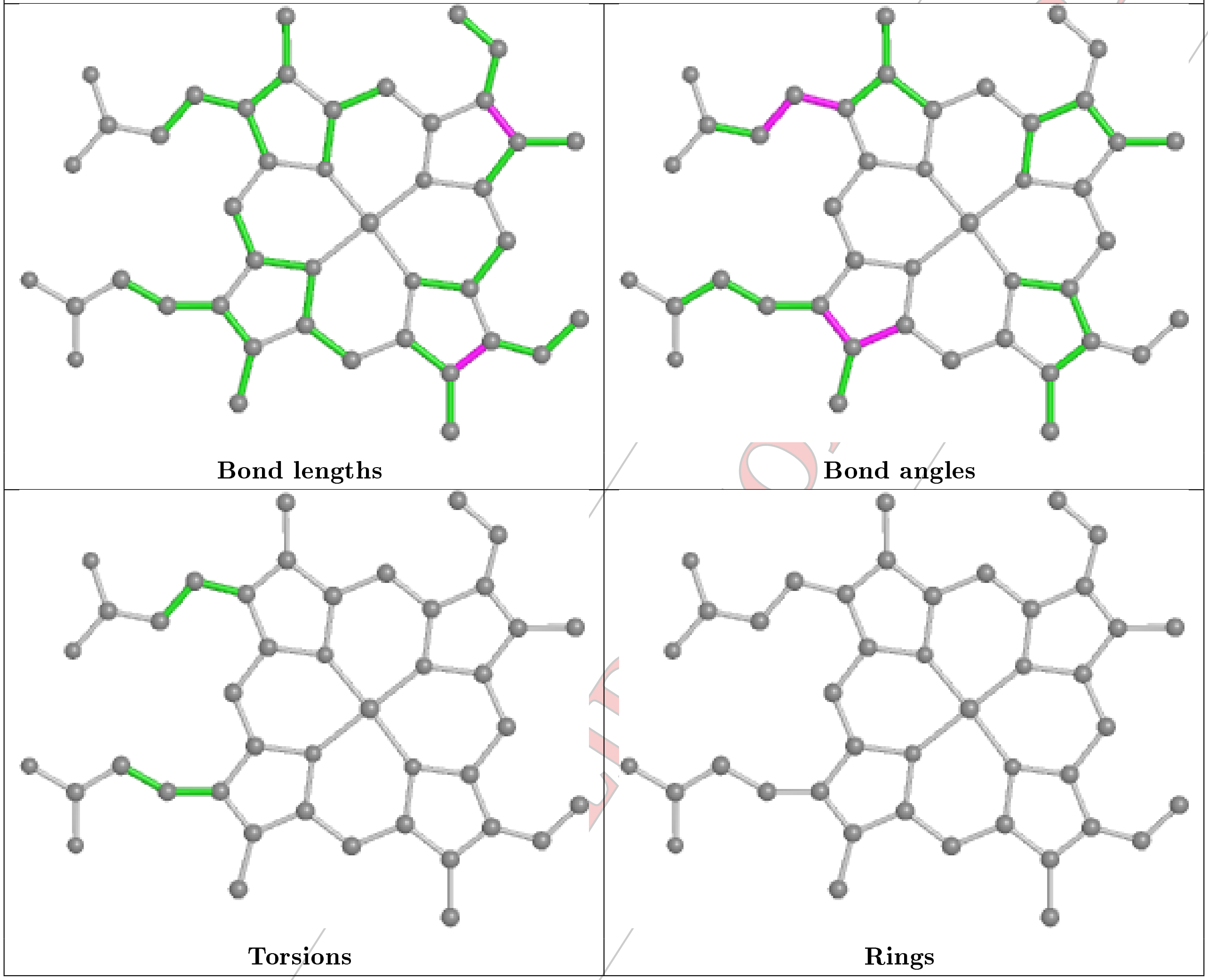

CONFIDENTIAL

Ligand DEB A 502

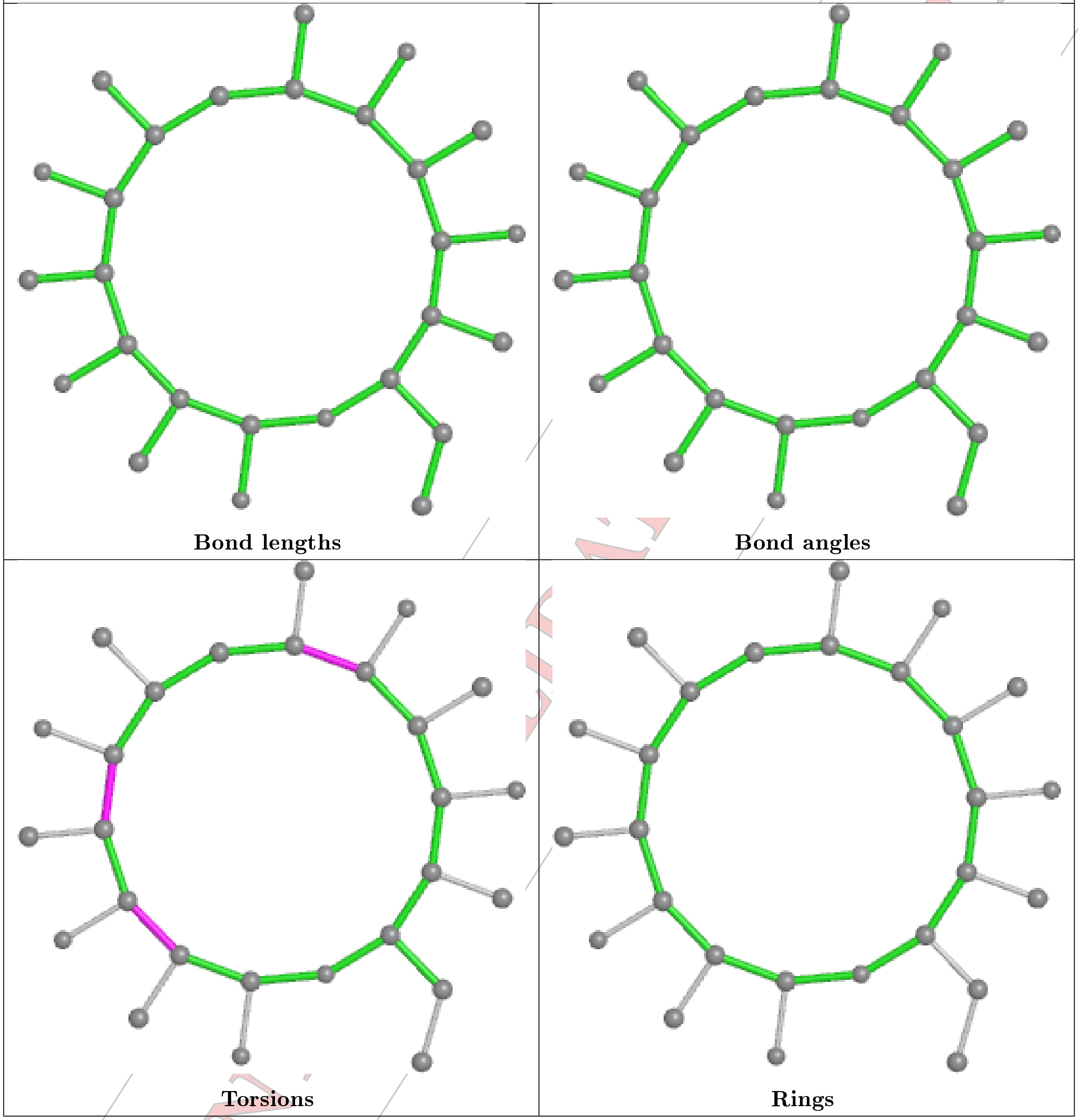

Ligand HEM E 501

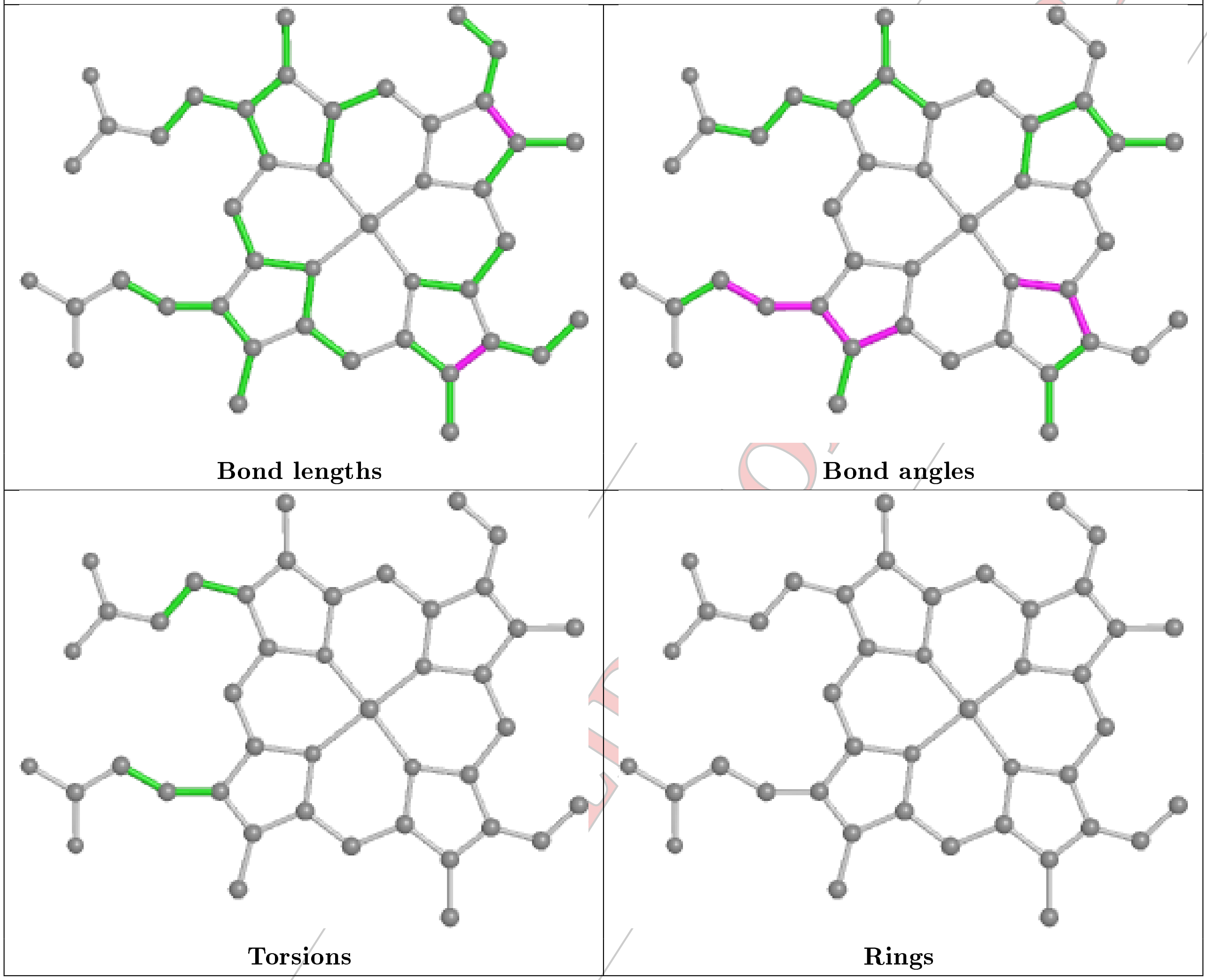

5.7 Other polymers (i)

There are no such residues in this entry.

5.8 Polymer linkage issues (i)

There are no chain breaks in this entry.

CONFIDENTIAL REPORT

6 Fit of model and data (i)

6.1 Protein, DNA and RNA chains (i)

In the following table, the column labelled '#RSRZ> 2' contains the number (and percentage) of RSRZ outliers, followed by percent RSRZ outliers for the chain as percentile scores relative to all X-ray entries and entries of similar resolution. The OWAB column contains the minimum, median, 95th percentile and maximum values of the occupancy-weighted average B-factor per residue. The column labelled 'Q< 0.9' lists the number of (and percentage) of residues with an average occupancy less than 0.9.

Mol	Chain	Analysed	<RSRZ>	#RSRZ>2			OWAB(Å^2)	Q<0.9
1	A	397/403 (98%)	0.20	18 (4%)	33	37	36, 52, 75, 134	0
1	B	403/403 (100%)	0.09	12 (2%)	50	55	34, 49, 76, 134	1 (0%)
1	C	397/403 (98%)	0.01	8 (2%)	65	68	33, 43, 59, 90	1 (0%)
1	D	396/403 (98%)	0.29	27 (6%)	17	21	43, 65, 87, 119	0
1	E	395/403 (98%)	0.31	38 (9%)	8	10	41, 61, 86, 145	0
1	F	397/403 (98%)	0.90	81 (20%)	1	1	45, 68, 105, 115	0
All	All	2385/2418 (98%)	0.30	184 (7%)	13	16	33, 56, 91, 145	2 (0%)

All (184) RSRZ outliers are listed below:

Mol	Chain	Res	Type	RSRZ
1	F	405	VAL	10.5
1	F	271[A]	VAL	9.7
1	F	270[A]	LEU	9.7
1	F	127[A]	LEU	8.8
1	F	407	TRP	8.0
1	B	209	ASP	7.6
1	F	377[A]	PHE	6.7
1	F	126	SER	6.4
1	F	123[A]	ARG	6.3
1	F	129[A]	ASP	6.1
1	A	209	ASP	5.9
1	F	132	LEU	5.9
1	F	380[A]	LEU	5.8
1	F	125[A]	ARG	5.5
1	F	142	ALA	5.5
1	E	305[A]	GLU	5.4
1	F	343[A]	ARG	5.4
1	B	265[A]	LYS	5.4
1	D	210	ALA	5.4

Continued on next page...

CONFIDENTIAL VALIDATION REPORT

Continued from previous page...

Mol	Chain	Res	Type	RSRZ
1	F	383	ALA	5.4
1	B	210	ALA	5.4
1	E	313	ALA	5.3
1	E	212	THR	5.2
1	F	139	GLY	5.2
1	F	141	PRO	5.2
1	F	130	SER	5.2
1	F	375[A]	ARG	5.1
1	E	211	PRO	5.1
1	F	267[A]	TYR	5.0
1	F	376[A]	ARG	5.0
1	D	226	ASP	5.0
1	B	9	THR	5.0
1	F	140	SER	4.9
1	F	332[A]	ASP	4.9
1	F	272	ALA	4.9
1	F	262	THR	4.9
1	F	261	LEU	4.9
1	F	385	PRO	4.9
1	F	328[A]	GLU	4.9
1	F	406	SER	4.9
1	F	404	ILE	4.8
1	F	274	PRO	4.8
1	F	379	THR	4.8
1	F	131	LEU	4.7
1	E	209	ASP	4.6
1	E	312[A]	ARG	4.6
1	F	273	ASP	4.5
1	F	382	LEU	4.5
1	D	209	ASP	4.5
1	D	224	ASP	4.5
1	B	211	PRO	4.4
1	F	364	LEU	4.4
1	D	221[A]	LEU	4.3
1	E	13	ALA	4.3
1	A	210	ALA	4.2
1	F	265[A]	LYS	4.1
1	F	337[A]	LEU	4.1
1	F	124	VAL	4.1
1	E	307	SER	4.1
1	F	37	ASP	4.1
1	F	209[A]	ASP	4.0

Continued on next page...

CONFIDENTIAL VALIDATION REPORT

Continued from previous page...

Mol	Chain	Res	Type	RSRZ
1	F	374[A]	VAL	4.0
1	D	333[A]	HIS	3.9
1	B	8	PRO	3.9
1	E	265[A]	LYS	3.8
1	D	36[A]	ARG	3.8
1	E	333[A]	HIS	3.7
1	E	306	LEU	3.7
1	F	277	VAL	3.6
1	B	6[A]	THR	3.6
1	F	339	PHE	3.6
1	A	370[A]	LEU	3.5
1	A	11	ALA	3.5
1	F	331	PHE	3.5
1	C	343	ARG	3.5
1	A	271[A]	VAL	3.5
1	E	271	VAL	3.4
1	D	208[A]	ARG	3.4
1	A	379	THR	3.4
1	D	220	ALA	3.4
1	F	342[A]	GLU	3.4
1	C	11	ALA	3.4
1	F	268[A]	GLU	3.4
1	E	226	ASP	3.4
1	D	406	SER	3.3
1	E	227	ASP	3.3
1	F	36[A]	ARG	3.3
1	F	280	ALA	3.2
1	F	138	HIS	3.2
1	D	225	ASN	3.2
1	E	303	ASP	3.2
1	D	263[A]	GLU	3.2
1	A	44[A]	ARG	3.2
1	F	384[A]	GLU	3.2
1	E	36[A]	ARG	3.2
1	E	310	THR	3.2
1	F	136	VAL	3.2
1	D	223	THR	3.1
1	E	308	THR	3.1
1	A	208	ARG	3.1
1	F	275	ALA	3.1
1	F	183[A]	ARG	3.1
1	F	208[A]	ARG	3.1

Continued on next page...

CONFIDENTIAL VALIDATION REPORT

Continued from previous page...

Mol	Chain	Res	Type	RSRZ
1	D	336[A]	GLU	3.1
1	F	122	PRO	3.0
1	D	227[A]	ASP	3.0
1	F	372	ALA	3.0
1	E	332[A]	ASP	3.0
1	D	265[A]	LYS	3.0
1	E	334	ALA	3.0
1	B	225[A]	ASN	3.0
1	F	388	GLY	2.9
1	E	270[A]	LEU	2.9
1	E	210	ALA	2.8
1	F	387	ALA	2.8
1	E	329[A]	GLU	2.8
1	E	387	ALA	2.8
1	D	386	VAL	2.8
1	F	330	VAL	2.8
1	F	378	PRO	2.8
1	A	189	ILE	2.8
1	A	224[A]	ASP	2.8
1	D	205	ALA	2.7
1	E	213	GLU	2.7
1	A	13	ALA	2.7
1	F	11	ALA	2.7
1	E	330	VAL	2.7
1	E	336[A]	GLU	2.7
1	F	263[A]	GLU	2.7
1	D	337	LEU	2.7
1	C	342[A]	GLU	2.6
1	F	386	VAL	2.6
1	F	371	SER	2.6
1	F	276	LEU	2.6
1	F	370	LEU	2.6
1	D	381	ASP	2.6
1	F	147	PHE	2.6
1	E	311[A]	VAL	2.6
1	F	327	ASP	2.6
1	D	332	ASP	2.5
1	A	85	PRO	2.5
1	D	267	TYR	2.5
1	F	340	HIS	2.5
1	E	272	ALA	2.5
1	B	5	HIS	2.5

Continued on next page...

CONFIDENTIAL VALIDATION REPORT

Continued from previous page...

Mol	Chain	Res	Type	RSRZ
1	F	35[A]	ARG	2.5
1	A	183[A]	ARG	2.4
1	F	373	LEU	2.4
1	E	335[A]	ASP	2.4
1	F	333	HIS	2.4
1	E	69	ARG	2.4
1	E	37	ASP	2.4
1	D	211	PRO	2.4
1	F	137	ALA	2.4
1	A	225[A]	ASN	2.4
1	F	269	SER	2.3
1	F	21[A]	LEU	2.3
1	F	334	ALA	2.3
1	F	133	ASP	2.3
1	B	11	ALA	2.2
1	C	47	TYR	2.2
1	B	12[A]	ASP	2.2
1	E	60	ASP	2.2
1	C	189	ILE	2.2
1	E	342[A]	GLU	2.2
1	E	343[A]	ARG	2.2
1	A	265[A]	LYS	2.2
1	E	115[A]	ARG	2.2
1	A	123	ARG	2.2
1	F	329[A]	GLU	2.1
1	E	23	HIS	2.1
1	C	44[A]	ARG	2.1
1	D	115[A]	ARG	2.1
1	D	385	PRO	2.1
1	B	342[A]	GLU	2.1
1	F	29	PRO	2.1
1	C	183[A]	ARG	2.1
1	D	329[A]	GLU	2.1
1	E	277	VAL	2.1
1	D	261	LEU	2.1
1	A	213[A]	GLU	2.1
1	A	182	THR	2.1
1	C	84	PHE	2.1
1	F	381[A]	ASP	2.0

CONFIDENTIAL VALIDATION REPORT

6.2 Non-standard residues in protein, DNA, RNA chains (i)

There are no non-standard protein/DNA/RNA residues in this entry.

6.3 Carbohydrates (i)

There are no carbohydrates in this entry.

6.4 Ligands (i)

In the following table, the Atoms column lists the number of modelled atoms in the group and the number defined in the chemical component dictionary. The B-factors column lists the minimum, median, 95th percentile and maximum values of B factors of atoms in the group. The column labelled 'Q< 0.9' lists the number of atoms with occupancy less than 0.9.

Mol	Type	Chain	Res	Atoms	RSCC	RSR	B-factors(Å^2)	Q<0.9
6	FMT	D	506	3/3	-0.17	0.49	104,104,107,110	0
6	FMT	C	554	3/3	-0.08	1.06	110,110,114,116	0
6	FMT	A	526	3/3	0.06	0.47	100,100,103,106	0
6	FMT	C	549	3/3	0.10	0.36	99,99,106,106	0
6	FMT	A	539	3/3	0.19	0.26	106,106,107,109	0
6	FMT	A	525	3/3	0.21	0.58	103,103,110,111	0
6	FMT	B	559	3/3	0.24	0.21	111,111,121,121	0
6	FMT	C	551	3/3	0.25	0.28	101,101,103,103	0
6	FMT	B	530	3/3	0.27	0.43	92,92,97,99	0
6	FMT	C	540	3/3	0.28	0.46	112,112,117,119	0
6	FMT	E	511	3/3	0.30	0.35	108,108,112,113	0
6	FMT	F	716	3/3	0.30	0.35	98,98,104,107	0
6	FMT	F	711	3/3	0.32	0.21	110,110,112,116	0
6	FMT	A	517	3/3	0.33	0.29	105,105,106,107	0
6	FMT	B	566	3/3	0.38	0.26	97,97,104,104	0
6	FMT	B	537	3/3	0.38	0.28	90,90,93,96	0
6	FMT	B	524	3/3	0.41	0.30	84,84,86,91	0
6	FMT	A	541	3/3	0.42	0.29	83,83,85,92	0
6	FMT	D	513	3/3	0.42	0.23	103,103,106,106	0
6	FMT	F	712	3/3	0.42	0.31	86,86,90,97	0
6	FMT	A	524	3/3	0.44	0.45	95,95,101,101	0
6	FMT	C	552	3/3	0.47	0.65	107,107,108,110	0
6	FMT	B	532	3/3	0.48	0.54	87,87,95,97	0
6	FMT	D	512	3/3	0.49	0.26	95,95,103,105	0
6	FMT	C	553	3/3	0.50	0.35	89,89,90,95	0
6	FMT	E	509	3/3	0.51	0.25	88,88,94,95	0
6	FMT	A	527	3/3	0.51	0.25	97,97,99,101	0

Continued on next page...

CONFIDENTIAL VALIDATION REPORT

Continued from previous page...

Mol	Type	Chain	Res	Atoms	RSCC	RSR	B-factors(Å^2)	Q<0.9
6	FMT	B	528	3/3	0.51	0.33	84,84,86,93	0
6	FMT	E	513	3/3	0.52	0.23	86,86,93,95	0
6	FMT	F	717	3/3	0.52	0.21	91,91,100,100	0
6	FMT	B	555	3/3	0.52	0.37	103,103,104,109	0
6	FMT	E	508	3/3	0.54	0.29	89,89,94,97	0
6	FMT	C	560	3/3	0.54	0.53	88,88,89,95	0
5	TRS	B	504	8/8	0.55	0.24	77,85,93,93	0
6	FMT	B	545	3/3	0.55	0.20	101,101,102,102	0
6	FMT	C	545	3/3	0.55	0.33	99,99,103,104	0
6	FMT	A	536	3/3	0.56	0.31	76,76,81,88	0
6	FMT	E	505	3/3	0.56	0.20	84,84,88,92	0
6	FMT	A	530	3/3	0.56	0.34	103,103,105,108	0
6	FMT	B	549	3/3	0.57	0.21	103,103,104,106	0
6	FMT	B	540	3/3	0.57	0.46	76,76,84,87	0
6	FMT	C	562	3/3	0.58	0.47	102,102,102,103	0
6	FMT	C	533	3/3	0.58	0.58	85,85,90,91	0
6	FMT	C	547	3/3	0.58	0.15	77,77,95,96	0
6	FMT	D	507	3/3	0.59	0.33	88,88,89,93	0
4	RAM	F	701	11/11	0.59	0.33	103,111,117,120	0
6	FMT	F	713	3/3	0.59	0.18	91,91,94,100	0
6	FMT	D	519	3/3	0.60	0.26	89,89,95,98	0
6	FMT	D	514	3/3	0.61	0.17	83,83,91,92	0
6	FMT	C	537	3/3	0.61	0.20	76,76,85,88	0
6	FMT	C	522	3/3	0.61	0.31	82,82,83,83	0
6	FMT	C	508	3/3	0.62	0.36	79,79,83,88	0
4	RAM	D	503	11/11	0.63	0.39	66,71,75,75	11
6	FMT	F	709	3/3	0.63	0.35	84,84,90,90	0
6	FMT	C	550	3/3	0.64	0.28	82,82,90,90	0
6	FMT	A	544	3/3	0.64	0.22	88,88,93,93	0
6	FMT	B	546	3/3	0.64	0.24	86,86,89,94	0
6	FMT	C	564	3/3	0.64	0.16	92,92,92,97	0
4	RAM	C	504	11/11	0.65	0.26	71,78,83,84	11
6	FMT	B	568	3/3	0.65	0.27	80,80,85,100	0
6	FMT	B	544	3/3	0.66	0.36	95,95,99,101	0
8	GOL	C	507	6/6	0.66	0.41	78,82,88,89	0
6	FMT	A	537	3/3	0.67	0.81	87,87,89,96	0
6	FMT	D	515	3/3	0.67	0.21	98,98,101,102	0
6	FMT	E	512	3/3	0.67	0.14	78,78,80,92	0
6	FMT	C	546	3/3	0.67	0.55	103,103,106,111	0
6	FMT	F	718	3/3	0.69	0.32	75,75,80,85	0
6	FMT	D	510	3/3	0.69	0.30	85,85,91,92	0
6	FMT	A	540	3/3	0.69	0.15	86,86,86,94	0

Continued on next page...

CONFIDENTIAL VALIDATION REPORT

Continued from previous page...

Mol	Type	Chain	Res	Atoms	RSCC	RSR	B-factors(Å^2)	Q<0.9
6	FMT	F	714	3/3	0.69	0.24	92,92,94,97	0
6	FMT	B	557	3/3	0.69	0.19	102,102,102,108	0
6	FMT	B	531	3/3	0.70	0.21	92,92,97,103	0
6	FMT	C	561	3/3	0.70	0.15	80,80,101,102	0
6	FMT	C	538	3/3	0.70	0.39	85,85,85,95	0
6	FMT	E	507	3/3	0.70	0.20	83,83,86,89	0
6	FMT	B	529	3/3	0.71	0.29	106,106,107,108	0
6	FMT	B	536	3/3	0.71	0.35	97,97,107,107	0
6	FMT	F	719	3/3	0.71	0.20	95,95,101,102	0
6	FMT	C	532	3/3	0.71	0.28	89,89,100,100	0
6	FMT	C	515	3/3	0.71	0.20	90,90,92,94	0
6	FMT	A	531	3/3	0.72	0.38	79,79,80,81	0
6	FMT	A	506	3/3	0.72	0.30	76,76,86,88	0
6	FMT	A	538	3/3	0.72	0.28	68,68,73,82	0
6	FMT	B	522	3/3	0.72	0.17	74,74,83,84	0
6	FMT	B	550	3/3	0.73	0.21	82,82,86,92	0
6	FMT	C	517	3/3	0.73	0.31	75,75,77,84	0
6	FMT	F	715	3/3	0.73	0.50	74,74,78,90	0
6	FMT	B	542	3/3	0.74	0.41	86,86,88,92	0
6	FMT	B	560	3/3	0.74	0.32	91,91,91,95	0
6	FMT	C	511	3/3	0.74	0.17	82,82,87,88	0
6	FMT	B	548	3/3	0.74	0.27	65,65,85,87	0
6	FMT	B	527	3/3	0.74	0.26	88,88,92,98	0
6	FMT	C	531	3/3	0.74	0.34	76,76,88,91	0
6	FMT	B	563	3/3	0.75	0.47	102,102,103,103	0
6	FMT	B	517	3/3	0.75	0.12	74,74,80,80	0
6	FMT	B	561	3/3	0.75	0.18	109,109,110,113	0
6	FMT	D	517	3/3	0.75	0.20	94,94,96,104	0
6	FMT	B	558	3/3	0.75	0.45	100,100,105,105	0
6	FMT	C	563	3/3	0.75	0.17	75,75,78,86	0
6	FMT	B	562	3/3	0.75	0.23	74,74,87,90	0
4	RAM	A	503[A]	11/11	0.75	0.41	64,76,87,88	11
6	FMT	A	534	3/3	0.75	0.19	86,86,90,93	0
8	GOL	B	505	6/6	0.76	0.36	100,102,106,107	0
6	FMT	D	511	3/3	0.76	0.26	90,90,96,99	0
6	FMT	C	523	3/3	0.76	0.18	84,84,87,95	0
6	FMT	A	521	3/3	0.76	0.58	102,102,102,107	0
5	TRS	A	504	8/8	0.76	0.44	111,118,119,121	0
6	FMT	B	520	3/3	0.76	0.18	82,82,86,86	0
6	FMT	B	556	3/3	0.76	0.33	88,88,94,98	0
6	FMT	D	504	3/3	0.76	0.41	90,90,94,95	0
6	FMT	C	539	3/3	0.77	0.17	81,81,82,89	0

Continued on next page...

CONFIDENTIAL VALIDATION REPORT

Continued from previous page...

Mol	Type	Chain	Res	Atoms	RSCC	RSR	B-factors(Å^2)	Q<0.9
4	RAM	C	503[A]	11/11	0.78	0.51	46,56,62,66	11
6	FMT	B	541	3/3	0.78	0.17	62,62,76,80	0
6	FMT	A	523	3/3	0.78	0.44	75,75,81,86	0
6	FMT	C	544	3/3	0.78	0.21	72,72,78,81	0
6	FMT	B	509	3/3	0.79	0.18	69,69,74,80	0
6	FMT	B	554	3/3	0.79	0.41	96,96,101,103	0
6	FMT	C	520	3/3	0.79	0.12	82,82,84,88	0
6	FMT	A	528	3/3	0.79	0.18	81,81,99,99	0
6	FMT	C	535	3/3	0.79	0.23	64,64,77,77	0
6	FMT	B	552	3/3	0.79	0.19	89,89,95,98	0
6	FMT	C	534	3/3	0.80	0.16	69,69,85,86	0
6	FMT	A	515	3/3	0.80	0.25	59,59,74,83	0
6	FMT	C	558	3/3	0.80	0.32	57,57,66,79	0
6	FMT	C	514	3/3	0.80	0.28	81,81,86,87	0
6	FMT	C	541	3/3	0.80	0.35	69,69,73,77	0
6	FMT	B	519	3/3	0.80	0.26	62,62,72,73	0
6	FMT	A	516	3/3	0.81	0.16	88,88,90,91	0
4	RAM	B	503[A]	11/11	0.81	0.41	43,50,55,58	11
6	FMT	C	526	3/3	0.81	0.18	56,56,59,62	0
6	FMT	B	533	3/3	0.81	0.12	93,93,94,104	0
6	FMT	C	556	3/3	0.81	0.22	66,66,79,82	0
6	FMT	B	539	3/3	0.81	0.27	83,83,89,91	0
6	FMT	D	516	3/3	0.81	0.18	92,92,99,100	0
6	FMT	F	707	3/3	0.81	0.16	79,79,89,90	0
6	FMT	A	529	3/3	0.81	0.19	75,75,77,84	0
6	FMT	B	525	3/3	0.81	0.21	77,77,82,83	0
6	FMT	A	514	3/3	0.82	0.16	60,60,60,65	0
6	FMT	A	507	3/3	0.82	0.26	71,71,72,73	0
6	FMT	B	526	3/3	0.82	0.15	90,90,94,95	0
4	RAM	E	503[A]	11/11	0.82	0.41	71,82,87,87	11
6	FMT	A	505	3/3	0.82	0.11	77,77,79,82	0
6	FMT	B	535	3/3	0.83	0.34	75,75,86,89	0
6	FMT	B	538	3/3	0.83	0.27	74,74,77,82	0
6	FMT	A	510	3/3	0.83	0.24	84,84,86,88	0
6	FMT	C	536	3/3	0.83	0.16	77,77,83,86	0
6	FMT	E	510	3/3	0.83	0.20	76,76,84,87	0
6	FMT	F	706	3/3	0.83	0.29	65,65,77,78	0
6	FMT	A	543	3/3	0.83	0.24	96,96,96,96	0
6	FMT	A	509	3/3	0.83	0.40	74,74,74,85	0
6	FMT	C	512	3/3	0.84	0.22	88,88,93,99	0
6	FMT	B	564	3/3	0.84	0.27	113,113,115,115	0
6	FMT	B	515	3/3	0.84	0.14	73,73,74,82	0

Continued on next page...

CONFIDENTIAL VALIDATION REPORT

Continued from previous page...

Mol	Type	Chain	Res	Atoms	RSCC	RSR	B-factors(Å^2)	Q<0.9
6	FMT	B	534	3/3	0.84	0.12	75,75,83,87	0
6	FMT	A	532	3/3	0.84	0.17	100,100,110,116	0
6	FMT	B	521	3/3	0.85	0.15	79,79,80,84	0
6	FMT	A	533	3/3	0.85	0.16	95,95,96,98	0
6	FMT	A	545[B]	3/3	0.86	0.36	30,30,37,43	3
6	FMT	C	530	3/3	0.86	0.38	81,81,82,88	0
6	FMT	C	510	3/3	0.86	0.28	61,61,82,86	0
8	GOL	C	506	6/6	0.86	0.19	86,90,99,102	0
6	FMT	C	543	3/3	0.86	0.28	83,83,86,92	0
6	FMT	C	529	3/3	0.86	0.20	78,78,88,89	0
6	FMT	A	508	3/3	0.86	0.23	59,59,74,79	0
6	FMT	B	508	3/3	0.86	0.09	49,49,65,73	0
6	FMT	B	507	3/3	0.86	0.17	87,87,88,88	0
6	FMT	C	565	3/3	0.86	0.26	68,68,74,77	0
6	FMT	A	519	3/3	0.87	0.24	87,87,89,94	0
6	FMT	C	548	3/3	0.87	0.17	63,63,76,78	0
6	FMT	B	547	3/3	0.87	0.30	72,72,84,91	0
5	TRS	F	705	8/8	0.87	0.17	50,57,61,62	0
6	FMT	B	516	3/3	0.87	0.13	87,87,89,90	0
6	FMT	A	535	3/3	0.87	0.10	90,90,92,98	0
6	FMT	C	519	3/3	0.87	0.17	71,71,77,78	0
6	FMT	E	506	3/3	0.88	0.18	58,58,62,70	3
6	FMT	D	509	3/3	0.88	0.23	68,68,71,76	0
6	FMT	A	512	3/3	0.88	0.21	60,60,69,75	0
6	FMT	A	518	3/3	0.89	0.23	88,88,94,96	0
6	FMT	B	512	3/3	0.89	0.17	58,58,72,79	0
6	FMT	D	505	3/3	0.89	0.21	84,84,84,86	0
6	FMT	C	527	3/3	0.89	0.26	78,78,81,89	0
6	FMT	C	521	3/3	0.89	0.17	50,50,67,73	0
6	FMT	B	518	3/3	0.89	0.12	81,81,81,88	0
6	FMT	B	569	3/3	0.89	0.13	81,81,85,86	0
6	FMT	B	514	3/3	0.89	0.10	81,81,89,89	0
6	FMT	D	508	3/3	0.89	0.10	58,58,59,63	0
6	FMT	B	510	3/3	0.89	0.14	85,85,89,92	0
6	FMT	B	565	3/3	0.89	0.11	85,85,88,92	0
6	FMT	C	559	3/3	0.90	0.18	80,80,81,88	0
8	GOL	F	704	6/6	0.90	0.24	74,84,85,94	0
6	FMT	A	522	3/3	0.90	0.46	69,69,71,80	0
6	FMT	B	511	3/3	0.90	0.11	74,74,79,87	0
6	FMT	C	513[B]	3/3	0.90	0.35	45,45,45,53	3
6	FMT	A	542	3/3	0.91	0.33	49,49,60,77	0
6	FMT	B	567	3/3	0.91	0.34	64,64,73,74	0

Continued on next page...

CONFIDENTIAL VALIDATION REPORT

Continued from previous page...

Mol	Type	Chain	Res	Atoms	RSCC	RSR	B-factors(Å^2)	Q<0.9
6	FMT	C	542	3/3	0.91	0.33	94,94,100,102	0
6	FMT	B	551	3/3	0.92	0.20	73,73,80,82	0
6	FMT	B	513	3/3	0.92	0.21	78,78,89,96	0
6	FMT	F	708	3/3	0.92	0.24	74,74,77,78	0
7	NA	E	515	1/1	0.92	0.23	63,63,63,63	0
6	FMT	F	710	3/3	0.92	0.14	77,77,80,86	0
6	FMT	C	525	3/3	0.93	0.19	52,52,62,66	0
8	GOL	B	506	6/6	0.93	0.21	76,81,89,96	0
6	FMT	A	511	3/3	0.93	0.16	68,68,80,87	0
6	FMT	D	518	3/3	0.93	0.27	77,77,80,81	0
6	FMT	A	520	3/3	0.93	0.11	78,78,79,83	0
6	FMT	C	509	3/3	0.93	0.17	76,76,79,83	0
6	FMT	C	555[B]	3/3	0.94	0.48	26,26,30,33	3
7	NA	D	520	1/1	0.94	0.23	62,62,62,62	0
6	FMT	C	518	3/3	0.94	0.12	53,53,68,76	0
6	FMT	B	543	3/3	0.94	0.29	81,81,86,87	0
3	DEB	C	502	27/27	0.94	0.15	34,37,41,45	0
3	DEB	E	502	27/27	0.94	0.13	44,49,54,59	0
6	FMT	C	524	3/3	0.94	0.09	75,75,77,82	0
2	HEM	F	702	43/43	0.94	0.12	48,56,71,80	0
6	FMT	C	528	3/3	0.94	0.10	60,60,65,84	0
7	NA	F	720	1/1	0.94	0.18	56,56,56,56	0
6	FMT	C	516	3/3	0.95	0.10	68,68,74,75	0
3	DEB	D	502	27/27	0.95	0.14	46,55,61,65	0
6	FMT	B	553	3/3	0.95	0.09	72,72,84,88	0
3	DEB	F	703	27/27	0.95	0.11	50,55,59,60	0
7	NA	A	547	1/1	0.96	0.41	66,66,66,66	0
3	DEB	B	502	27/27	0.96	0.20	36,39,45,46	0
6	FMT	E	514[B]	3/3	0.96	0.21	47,47,47,56	3
7	NA	C	566	1/1	0.96	0.13	43,43,43,43	0
2	HEM	E	501	43/43	0.96	0.10	39,44,50,55	0
2	HEM	D	501	43/43	0.97	0.14	38,43,53,58	0
6	FMT	B	523	3/3	0.97	0.13	51,51,52,59	0
2	HEM	B	501	43/43	0.97	0.16	32,35,38,49	0
7	NA	B	570	1/1	0.97	0.16	53,53,53,53	0
8	GOL	C	505	6/6	0.97	0.11	44,48,50,59	0
2	HEM	A	501	43/43	0.97	0.14	32,36,42,51	0
2	HEM	C	501	43/43	0.97	0.14	30,33,37,43	0
6	FMT	E	504	3/3	0.97	0.12	66,66,70,74	0
3	DEB	A	502	27/27	0.97	0.17	36,42,48,49	0
6	FMT	A	513	3/3	0.98	0.12	59,59,62,63	0
7	NA	A	546	1/1	0.98	0.19	45,45,45,45	0

Continued on next page...

CONFIDENTIAL VALIDATION REPORT

Continued from previous page...

Mol	Type	Chain	Res	Atoms	RSCC	RSR	B-factors($Å^2$)	Q<0.9
6	FMT	C	557	3/3	0.99	0.12	48,48,53,53	0

The following is a graphical depiction of the model fit to experimental electron density of all instances of the Ligand of Interest. In addition, ligands with molecular weight > 250 and outliers as shown on the geometry validation Tables will also be included. Each fit is shown from different orientation to approximate a three-dimensional view.

Electron density around DEB C 502:

$2mF_o$-DF_c (at 0.7 rmsd) in gray
mF_o-DF_c (at 3 rmsd) in purple (negative)
and green (positive)

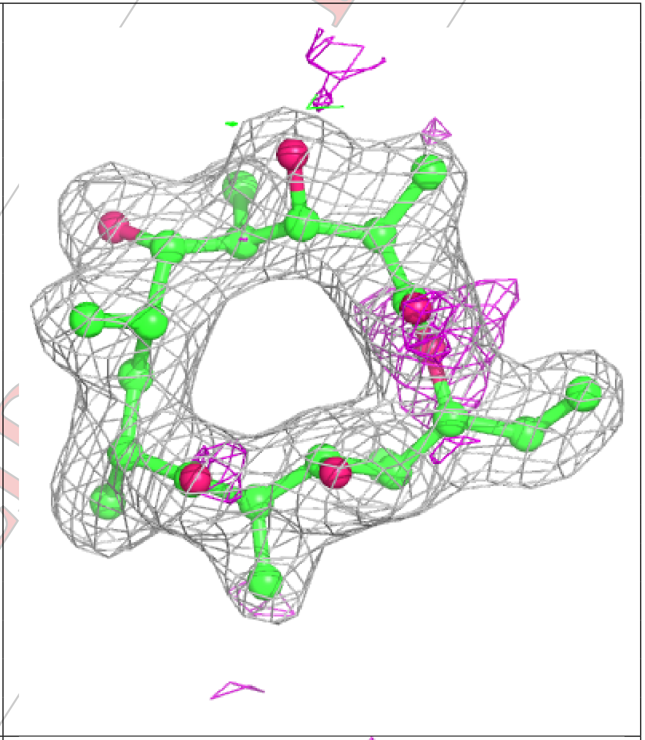

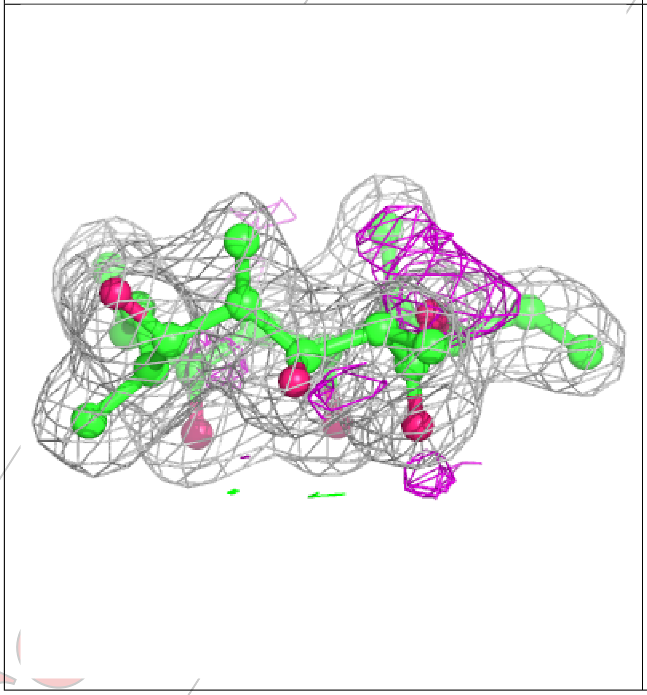

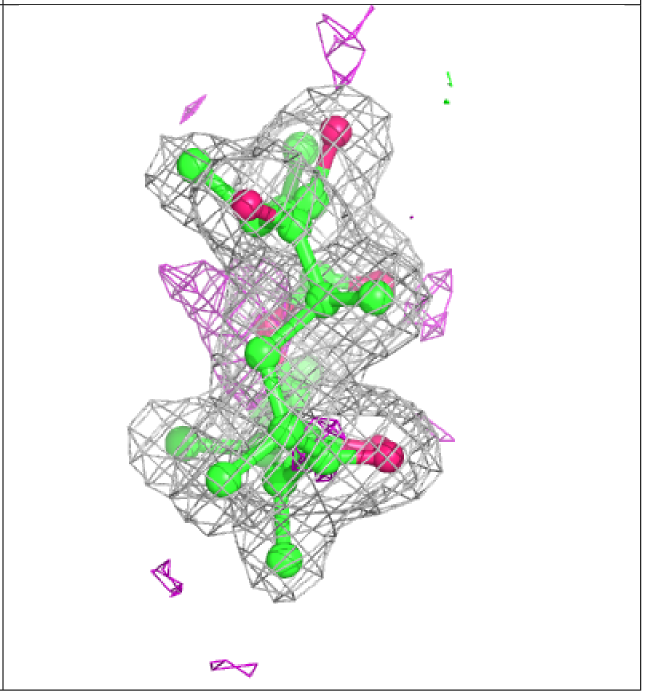

Electron density around DEB E 502:

$2mF_o$-DF_c (at 0.7 rmsd) in gray
mF_o-DF_c (at 3 rmsd) in purple (negative)
and green (positive)

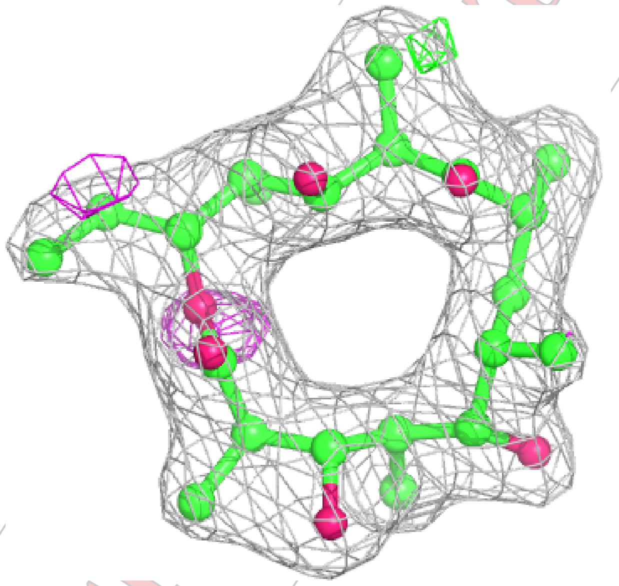

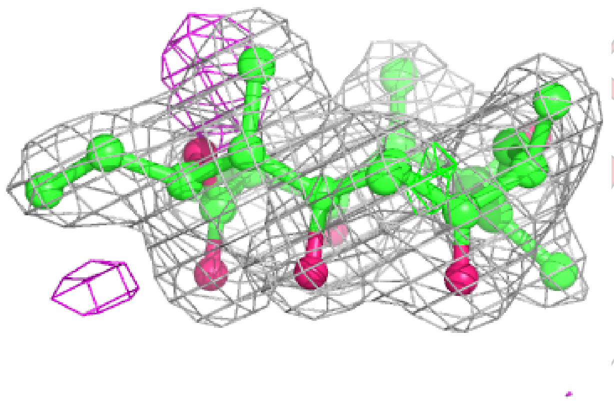

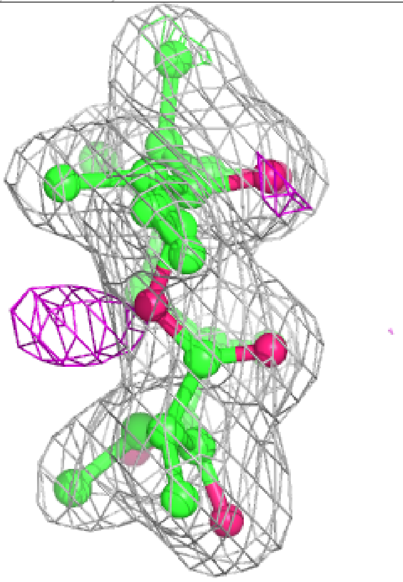

Electron density around HEM F 702:

$2mF_o$-DF_c (at 0.7 rmsd) in gray
mF_o-DF_c (at 3 rmsd) in purple (negative)
and green (positive)

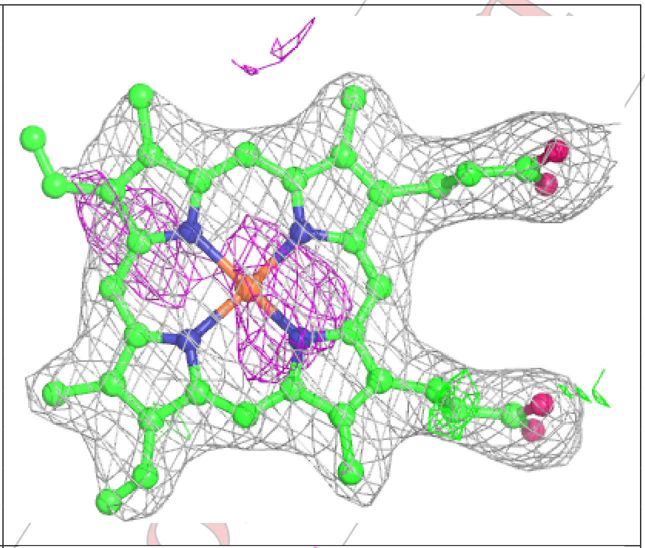

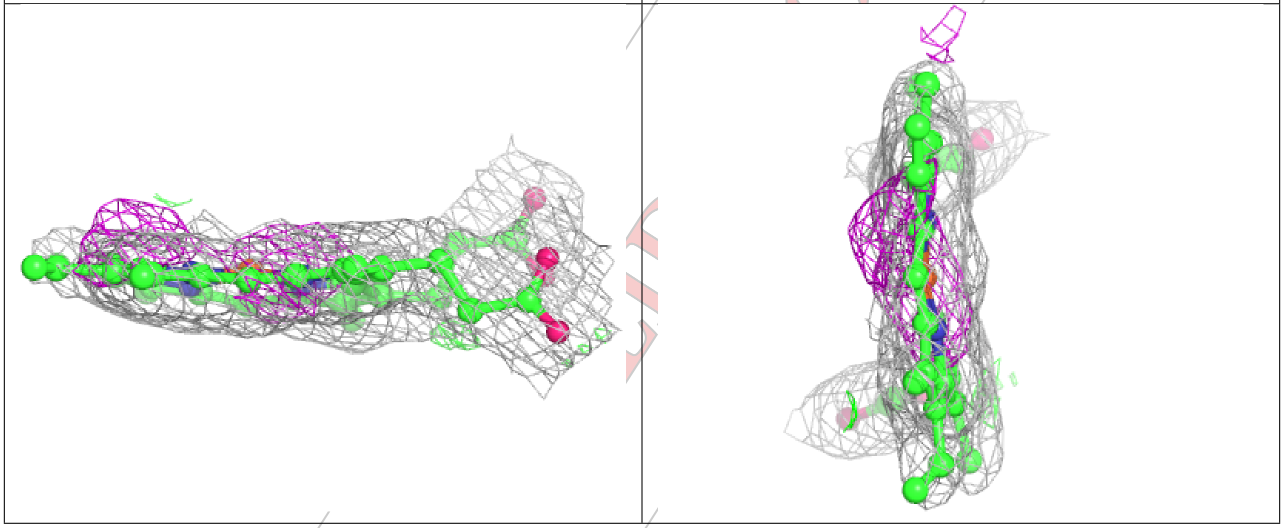

CONFIDENTIAL

Electron density around DEB D 502:

$2mF_o$-DF_c (at 0.7 rmsd) in gray
mF_o-DF_c (at 3 rmsd) in purple (negative)
and green (positive)

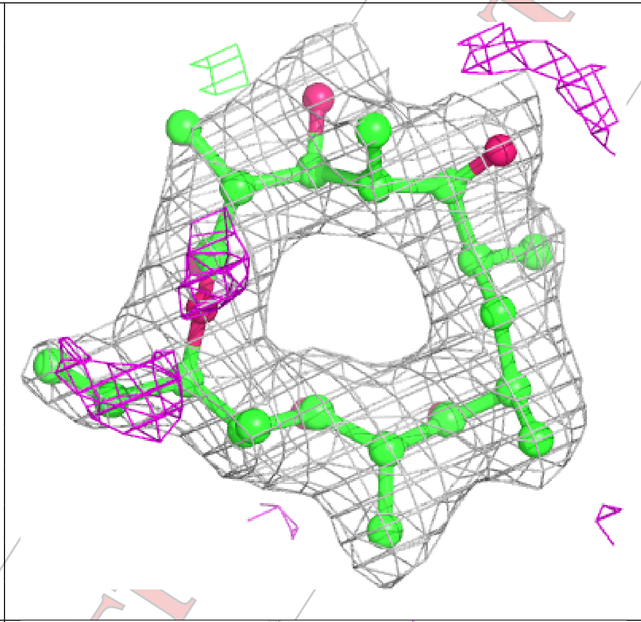

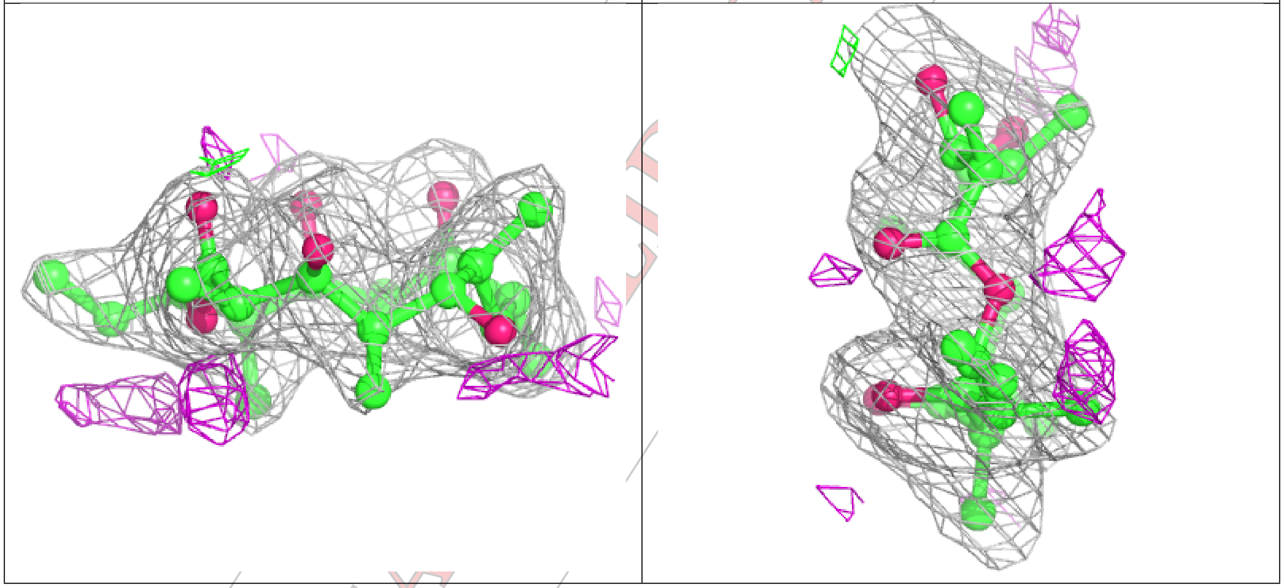

CONFIDENTIAL DRAFT

Electron density around DEB F 703:

$2mF_o$-DF_c (at 0.7 rmsd) in gray
mF_o-DF_c (at 3 rmsd) in purple (negative)
and green (positive)

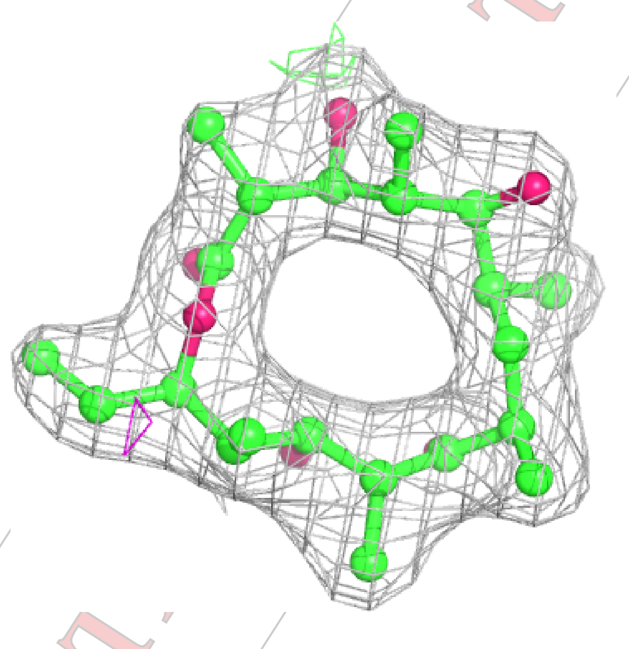

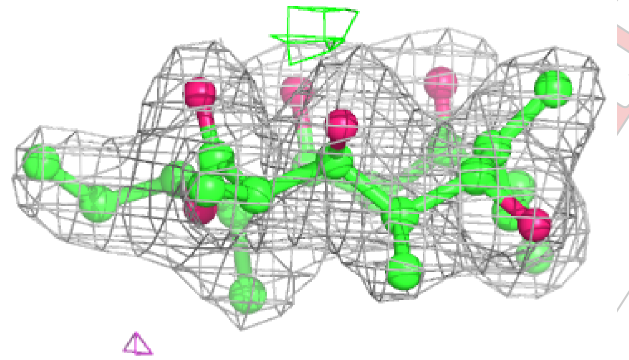

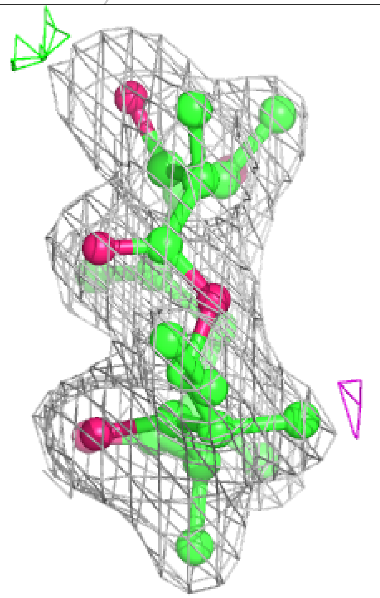

Electron density around DEB B 502:

2mF$_o$-DF$_c$ (at 0.7 rmsd) in gray
mF$_o$-DF$_c$ (at 3 rmsd) in purple (negative)
and green (positive)

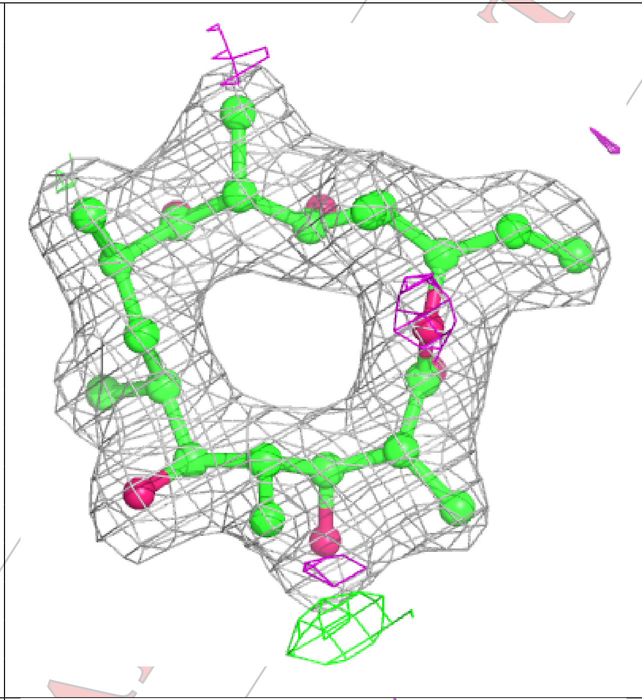

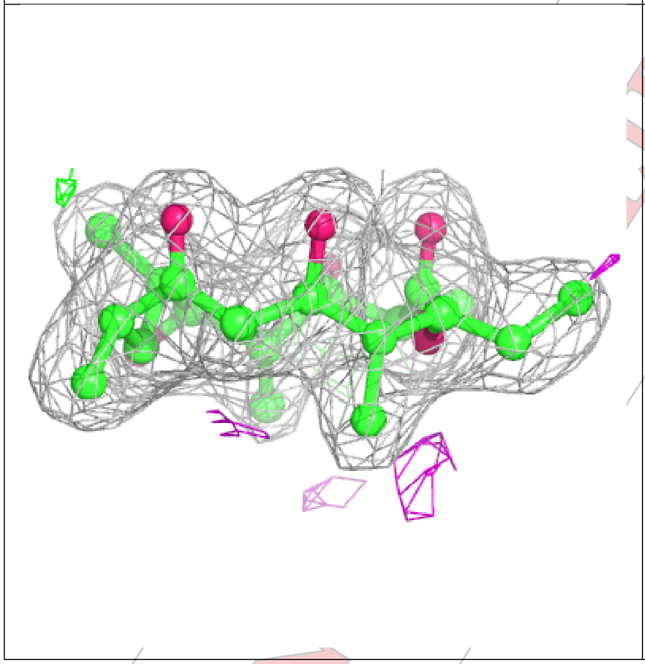

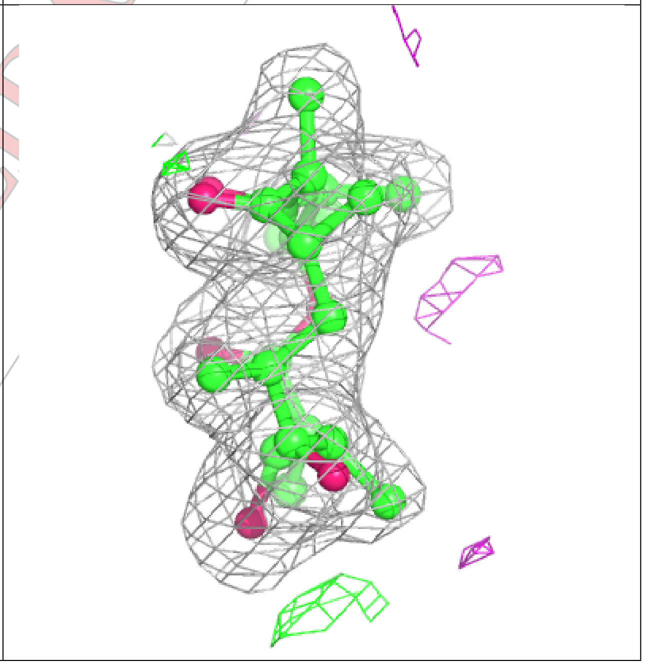

Electron density around HEM E 501:

$2mF_o-DF_c$ (at 0.7 rmsd) in gray
mF_o-DF_c (at 3 rmsd) in purple (negative)
and green (positive)

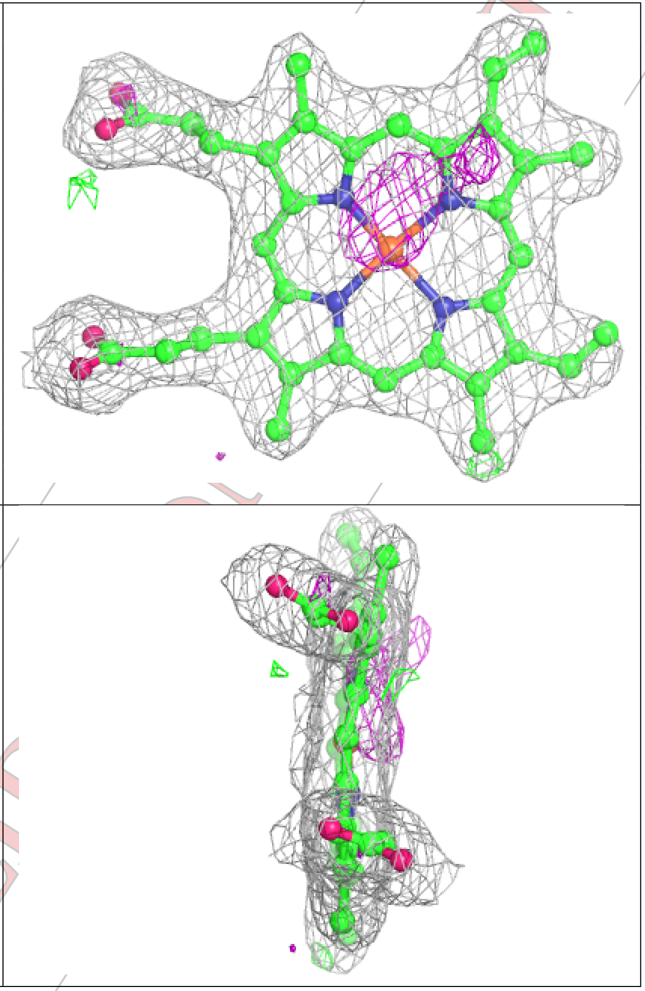

CONFIDENTIAL VALIDATION REPORT

Electron density around HEM D 501:

$2mF_o$-DF_c (at 0.7 rmsd) in gray
mF_o-DF_c (at 3 rmsd) in purple (negative)
and green (positive)

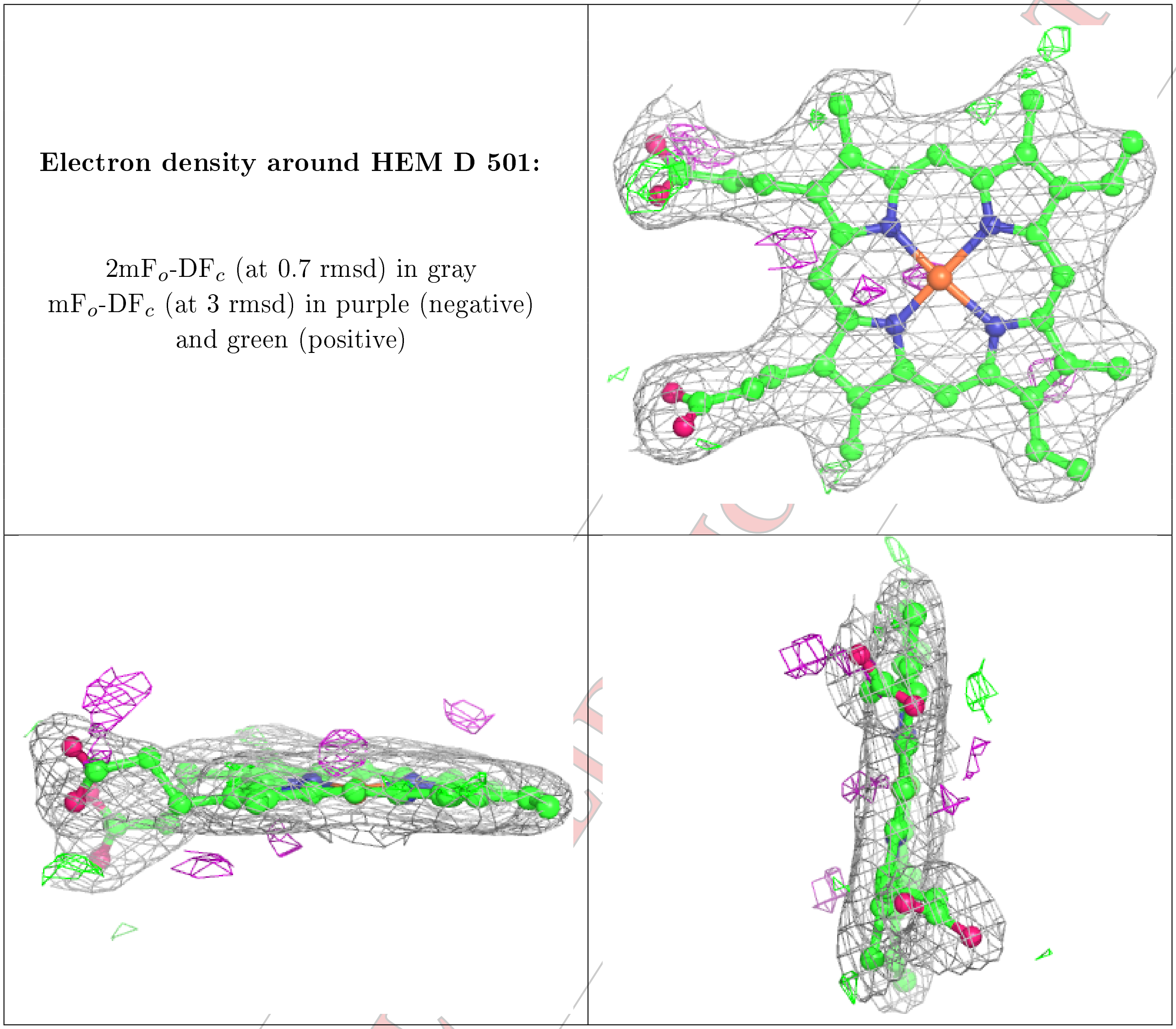

CONFIDENTIAL DRAFT

Electron density around HEM B 501:

2mF$_o$-DF$_c$ (at 0.7 rmsd) in gray
mF$_o$-DF$_c$ (at 3 rmsd) in purple (negative)
and green (positive)

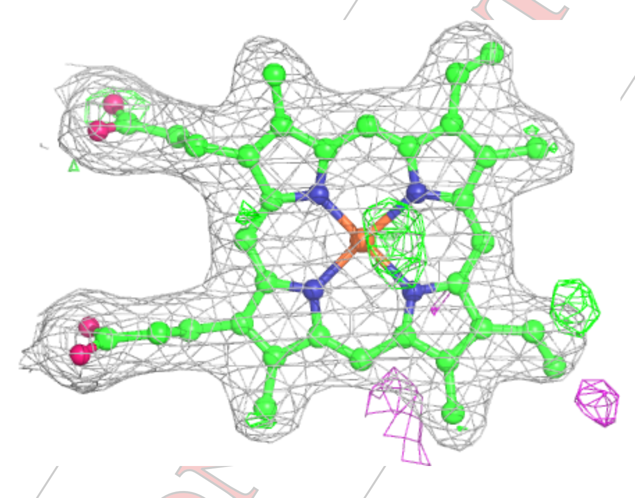

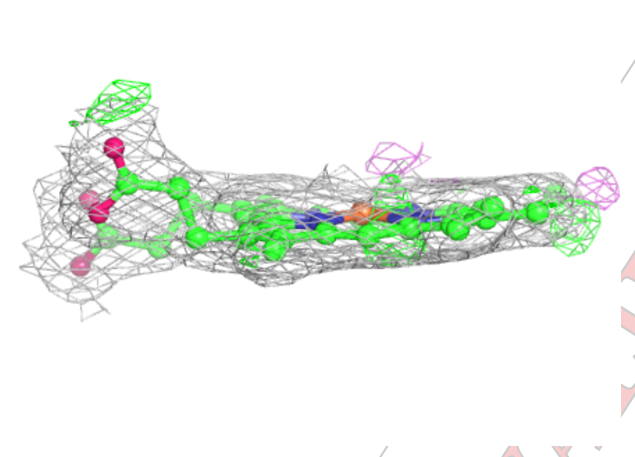

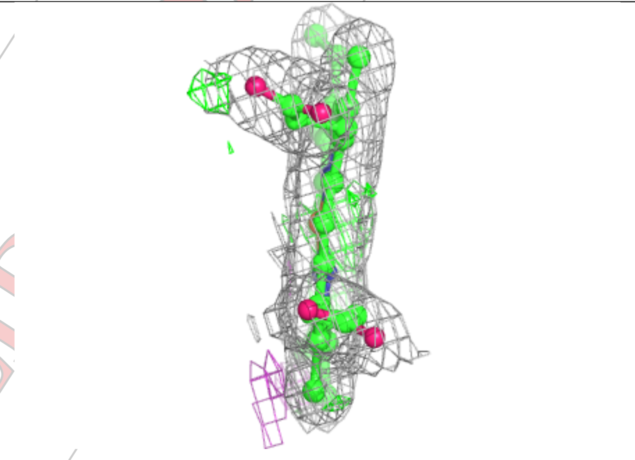

CONFIDENTIAL VALIDATION REPORT

Electron density around HEM A 501:

2mF$_o$-DF$_c$ (at 0.7 rmsd) in gray
mF$_o$-DF$_c$ (at 3 rmsd) in purple (negative)
and green (positive)

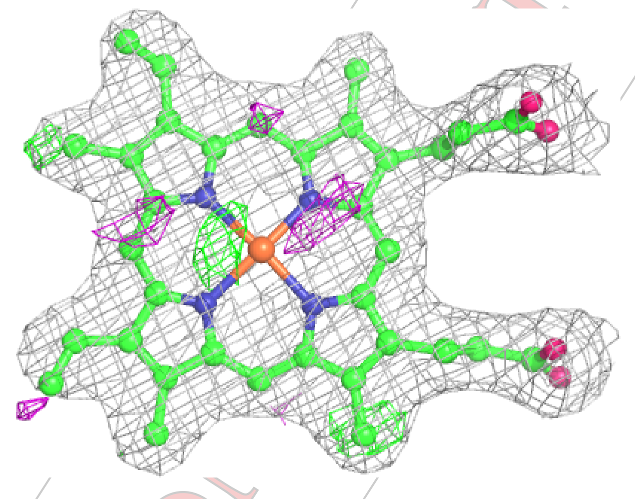

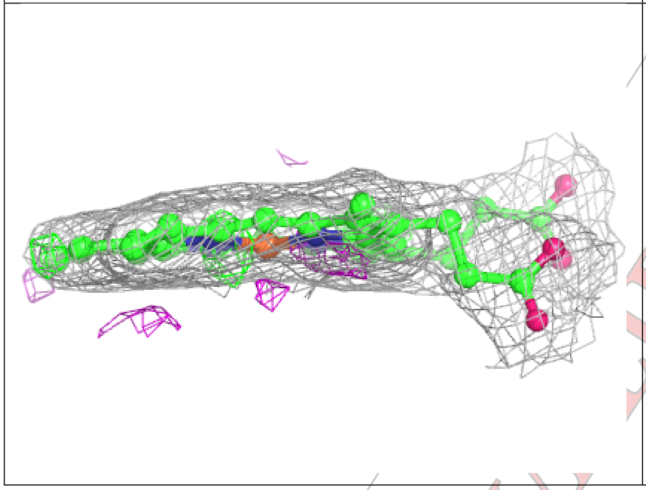

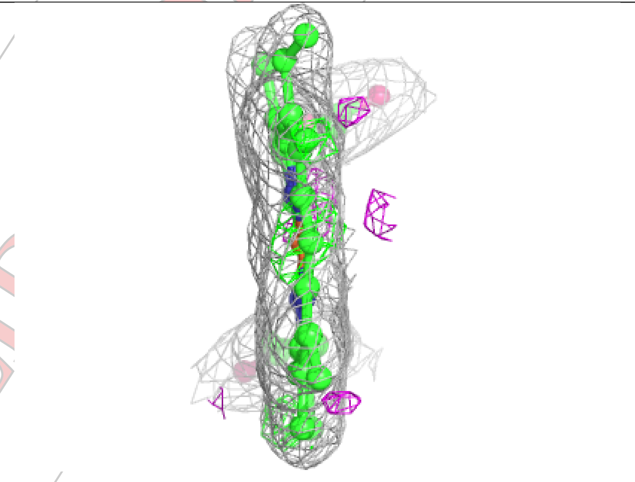

CONFIDENTIAL VALIDATION REPORT

Electron density around HEM C 501:

$2mF_o$-DF_c (at 0.7 rmsd) in gray
mF_o-DF_c (at 3 rmsd) in purple (negative)
and green (positive)

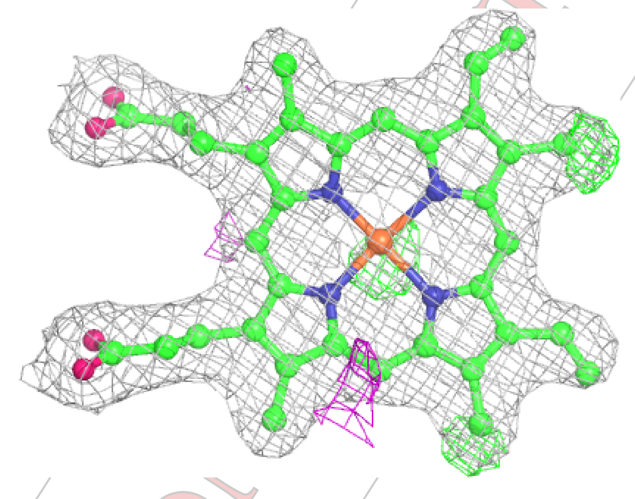

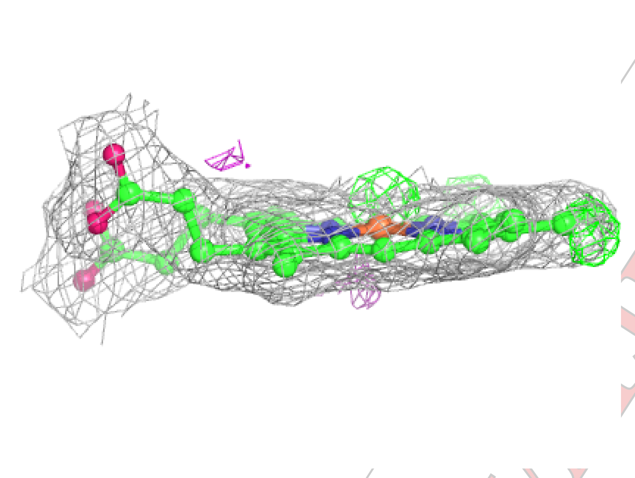

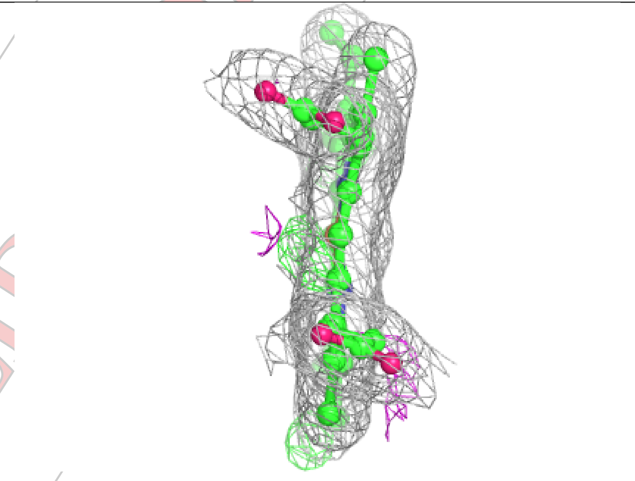

CONFIDENTIAL

Electron density around DEB A 502:

2mF$_o$-DF$_c$ (at 0.7 rmsd) in gray
mF$_o$-DF$_c$ (at 3 rmsd) in purple (negative)
and green (positive)

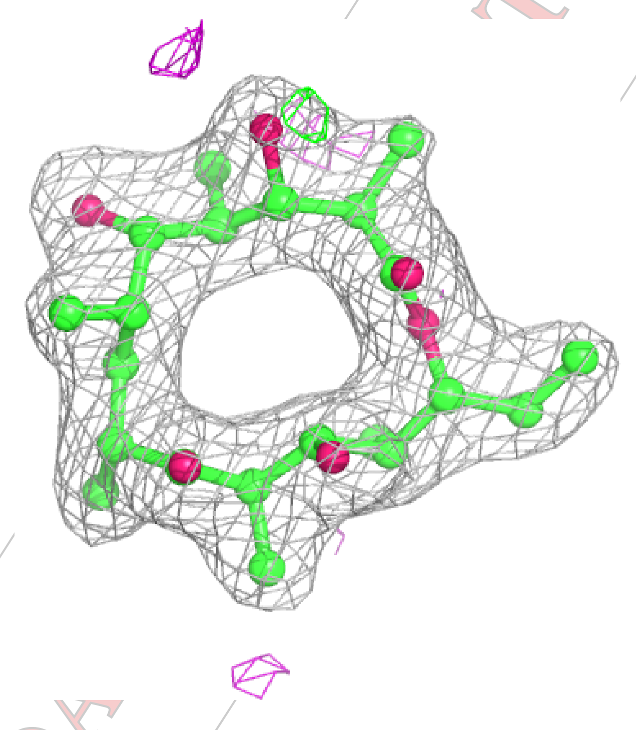

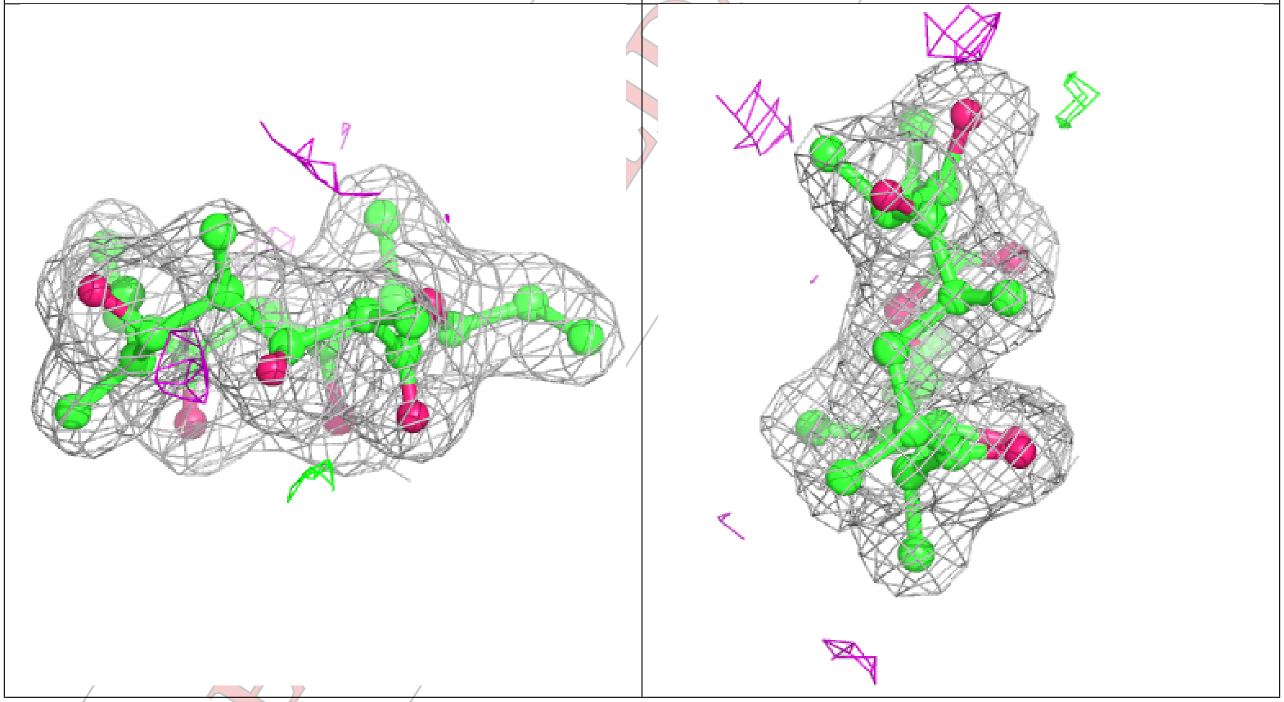

6.5 Other polymers

There are no such residues in this entry.
