# Supplementary material for: Dissecting the Cytochrome P450 OleP Substrate Specificity: Evidence for a Preferential Substrate
Source: Biomolecules. 2020 Oct 6;10(10):1411. doi: 10.3390/biom10101411 (PMC7600006; doi:10.3390/biom10101411)
Supplement: Supplementary file 1 [file biomolecules-10-01411-s001.zip › SupplMat_&_ValRep/6ZI2_D_1292109595_val-report-full_P1.pdf]

Full wwPDB X-ray Structure Validation Report (i)

Jul 28, 2020 – 04:15 PM BST

PDB ID	:	6ZI2
Title	:	OleP-oleandolide(DEO) in low salt crystallization conditions
Deposited on	:	2020-06-24
Resolution	:	2.93 Å(reported)

This is a Full wwPDB X-ray Structure Validation Report.

This report is produced by the wwPDB biocuration pipeline after annotation of the structure.

We welcome your comments at *validation@mail.wwpdb.org*
A user guide is available at
https://www.wwpdb.org/validation/2017/XrayValidationReportHelp
with specific help available everywhere you see the (i) symbol.

The following versions of software and data (see references (i)) were used in the production of this report:

MolProbity	:	4.02b-467
Mogul	:	1.8.5 (274361), CSD as541be (2020)
Xtriage (Phenix)	:	1.13
EDS	:	2.13
buster-report	:	1.1.7 (2018)
Percentile statistics	:	20191225.v01 (using entries in the PDB archive December 25th 2019)
Refmac	:	5.8.0158
CCP4	:	7.0.044 (Gargrove)
Ideal geometry (proteins)	:	Engh & Huber (2001)
Ideal geometry (DNA, RNA)	:	Parkinson et al. (1996)
Validation Pipeline (wwPDB-VP)	:	2.13

CONFIDENTIAL VALIDATION REPORT

1 Overall quality at a glance (i)

The following experimental techniques were used to determine the structure:
X-RAY DIFFRACTION

The reported resolution of this entry is 2.93 Å.

Percentile scores (ranging between 0-100) for global validation metrics of the entry are shown in the following graphic. The table shows the number of entries on which the scores are based.

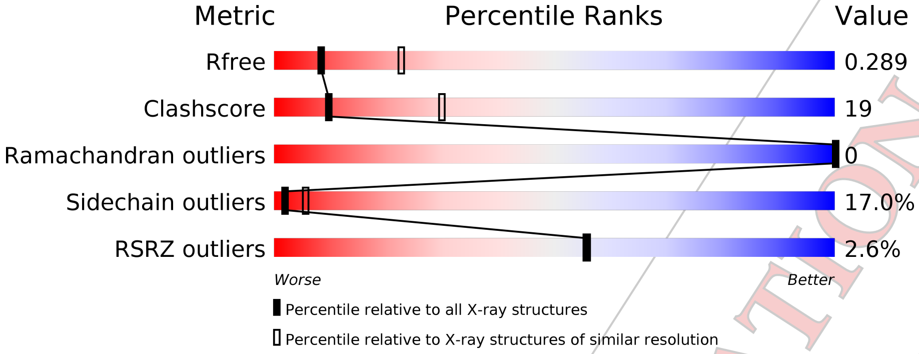

Metric	Whole archive (#Entries)	Similar resolution (#Entries, resolution range(Å))
R_{free}	130704	2969 (2.98-2.90)
Clashscore	141614	3218 (2.98-2.90)
Ramachandran outliers	138981	3122 (2.98-2.90)
Sidechain outliers	138945	3124 (2.98-2.90)
RSRZ outliers	127900	2902 (2.98-2.90)

The table below summarises the geometric issues observed across the polymeric chains and their fit to the electron density. The red, orange, yellow and green segments on the lower bar indicate the fraction of residues that contain outliers for >=3, 2, 1 and 0 types of geometric quality criteria respectively. A grey segment represents the fraction of residues that are not modelled. The numeric value for each fraction is indicated below the corresponding segment, with a dot representing fractions <=5% The upper red bar (where present) indicates the fraction of residues that have poor fit to the electron density. The numeric value is given above the bar.

Mol	Chain	Length	Quality of chain
1	A	407	61% 32% • •
1	B	407	55% 32% 11% •
1	C	407	57% 33% 7% •
1	D	407	63% 30% • •
1	E	407	% 60% 29% 6% 5%
1	F	407	% 64% 27% 6% •

Continued on next page...

CONFIDENTIAL VALIDATION REPORT

Continued from previous page...

Mol	Chain	Length	Quality of chain
1	G	407	5% / 60% 28% 6% 6%
1	H	407	63% 28% 6% •
1	I	407	14% / 31% 23% 7% 39%

The following table lists non-polymeric compounds, carbohydrate monomers and non-standard residues in protein, DNA, RNA chains that are outliers for geometric or electron-density-fit criteria:

Mol	Type	Chain	Res	Chirality	Geometry	Clashes	Electron density
3	QR8	E	502	X	X	-	-

CONFIDENTIAL VALIDATION REPORT

2 Entry composition ⓘ

There are 4 unique types of molecules in this entry. The entry contains 27067 atoms, of which 0 are hydrogens and 0 are deuteriums.

In the tables below, the ZeroOcc column contains the number of atoms modelled with zero occupancy, the AltConf column contains the number of residues with at least one atom in alternate conformation and the Trace column contains the number of residues modelled with at most 2 atoms.

- Molecule 1 is a protein called Cytochrome P-450.

Mol	Chain	Residues	Atoms					ZeroOcc	AltConf	Trace
1	A	394	Total 3073	C 1934	N 551	O 575	S 13	0	0	0
1	B	395	Total 3078	C 1937	N 552	O 576	S 13	0	0	0
1	C	395	Total 3078	C 1937	N 552	O 576	S 13	0	0	0
1	D	394	Total 3067	C 1931	N 548	O 575	S 13	0	0	0
1	E	386	Total 3012	C 1897	N 540	O 563	S 12	0	0	0
1	F	394	Total 3073	C 1934	N 551	O 575	S 13	0	0	0
1	G	381	Total 2976	C 1879	N 534	O 550	S 13	0	0	0
1	H	395	Total 3078	C 1937	N 552	O 576	S 13	0	0	0
1	I	248	Total 1915	C 1214	N 344	O 346	S 11	0	0	0

- Molecule 2 is PROTOPORPHYRIN IX CONTAINING FE (three-letter code: HEM) (formula: $C_{34}H_{32}FeN_4O_4$).

CONFIDENTIAL VALIDATION REPORT

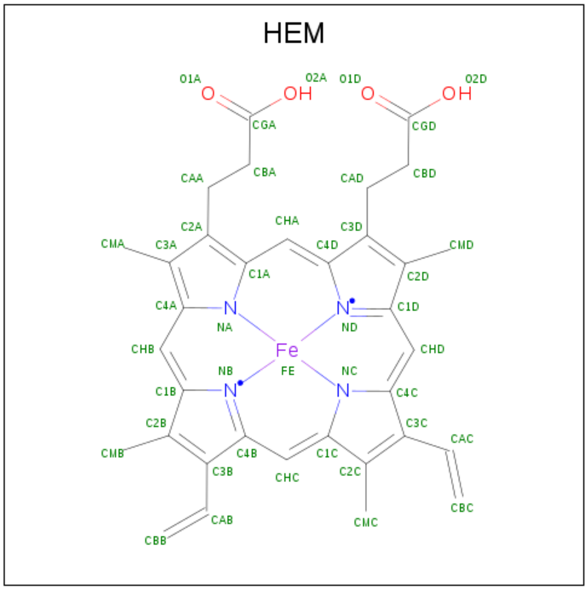

Mol	Chain	Residues	Atoms	ZeroOcc	AltConf
2	A	1	Total C Fe N O<br>43 34 1 4 4	0	0
2	B	1	Total C Fe N O<br>43 34 1 4 4	0	0
2	C	1	Total C Fe N O<br>43 34 1 4 4	0	0
2	D	1	Total C Fe N O<br>43 34 1 4 4	0	0
2	E	1	Total C Fe N O<br>43 34 1 4 4	0	0
2	F	1	Total C Fe N O<br>43 34 1 4 4	0	0
2	G	1	Total C Fe N O<br>43 34 1 4 4	0	0
2	H	1	Total C Fe N O<br>43 34 1 4 4	0	0
2	I	1	Total C Fe N O<br>43 34 1 4 4	0	0

- Molecule 3 is (3 {R},4 {S},5 {R},6 {S},7 {S},9 {S},11 {R},12 {S},13 {R},14 {R})-3,5,7,9 ,11,13,14-heptamethyl-4,6,12-tris(oxidanyl)-1-oxacyclotetradecane-2,10-dione (three-letter code: QR8) (formula: $C_{20}H_{36}O_6$) (labeled as "Ligand of Interest" by author).

CONFIDENTIAL VALIDATION REPORT

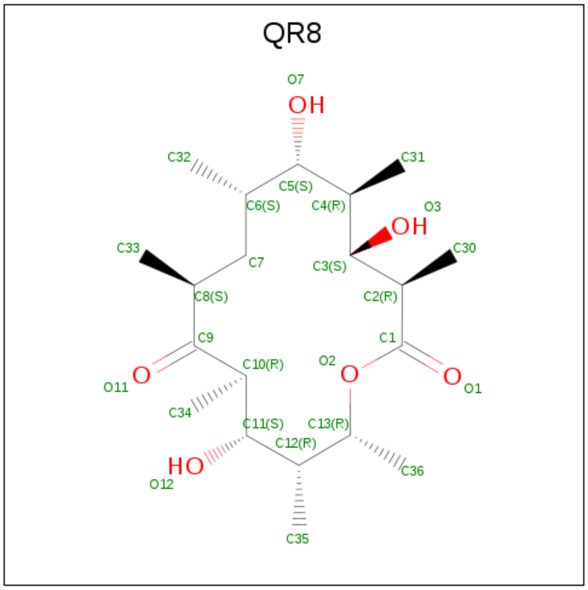

Mol	Chain	Residues	Atoms			ZeroOcc	AltConf
3	A	1	Total 26	C 20	O 6	0	0
3	B	1	Total 26	C 20	O 6	0	0
3	C	1	Total 26	C 20	O 6	0	0
3	D	1	Total 26	C 20	O 6	0	0
3	E	1	Total 26	C 20	O 6	0	0
3	F	1	Total 26	C 20	O 6	0	0
3	G	1	Total 26	C 20	O 6	0	0
3	H	1	Total 26	C 20	O 6	0	0
3	I	1	Total 26	C 20	O 6	0	0

- Molecule 4 is water.

Mol	Chain	Residues	Atoms		ZeroOcc	AltConf
4	A	19	Total 19	O 19	0	0
4	B	7	Total 7	O 7	0	0
4	C	14	Total 14	O 14	0	0

Continued on next page...

CONFIDENTIAL VALIDATION REPORT

Continued from previous page...

Mol	Chain	Residues	Atoms	ZeroOcc	AltConf
4	D	25	Total O<br>25 25	0	0
4	E	6	Total O<br>6 6	0	0
4	F	7	Total O<br>7 7	0	0
4	G	9	Total O<br>9 9	0	0
4	H	4	Total O<br>4 4	0	0
4	I	5	Total O<br>5 5	0	0

CONFIDENTIAL VALIDATION REPORT

3 Residue-property plots ⓘ

These plots are drawn for all protein, RNA, DNA and oligosaccharide chains in the entry. The first graphic for a chain summarises the proportions of the various outlier classes displayed in the second graphic. The second graphic shows the sequence view annotated by issues in geometry and electron density. Residues are color-coded according to the number of geometric quality criteria for which they contain at least one outlier: green = 0, yellow = 1, orange = 2 and red = 3 or more. A red dot above a residue indicates a poor fit to the electron density (RSRZ > 2). Stretches of 2 or more consecutive residues without any outlier are shown as a green connector. Residues present in the sample, but not in the model, are shown in grey.

• Molecule 1: Cytochrome P-450

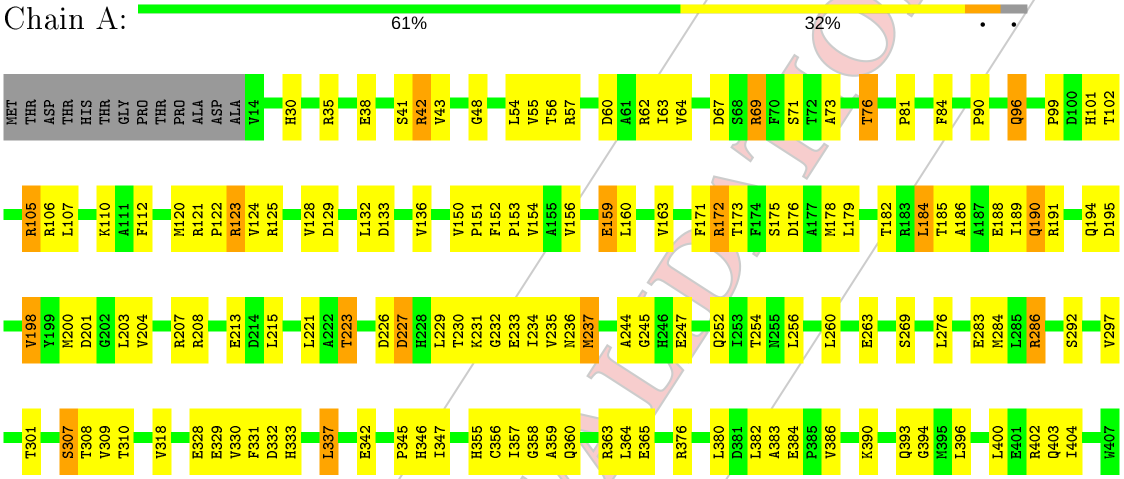

• Molecule 1: Cytochrome P-450

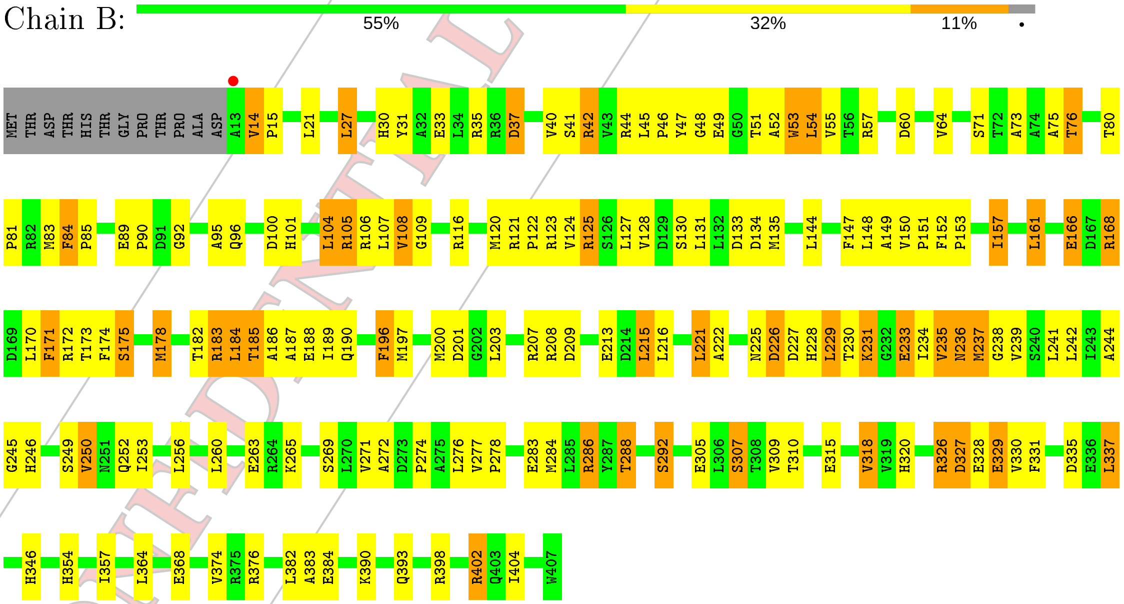

• Molecule 1: Cytochrome P-450

CONFIDENTIAL VALIDATION REPORT

Chain C: 57% 33% 7% •

MET THR ASP THR HIS THR GLY PRO THR PRO ALA ASP A13 V14 H23 D26 L27 D28 P29 H30 Y31 V40 S41 R42 P46 Y47 G48 T51 A52 W53 L54 V55 T56 R57 V64 L65 G66 D67 S68 A73 T76 P81 P85 E89 P90 D91 G92 V93 L94 A95 H101 T102 R103 L104 R105 R106 L107 V108 T113 R116 R121 P122 R123 V124 R125 S126 L127 V128 D129 S130 L131 L132 M135 L144 L148 A149 V150 P151 F152 P153 V154 I157 L160 L161 L165 E166 D167 R168 F171 R172 T173 F174 S175 D176 A177 M178 L179 T185 I189 Q190 R191 V192 Q193 Q194 D195 F196 M197 V198 Y199 M200 D201 G202 L203 V204 A205 Q206 R207 R208 D209 L215 L216 G217 A220 L221 A222 T223 D224 D227 H228 L229 T230 E233 I234 V235 N236 M237 G238 V239 S240 L241 L242 E247 T248 S249 V250 N251 Q252 I253 L260 E263 E268 S269 L270 V271 A272 D273 P274 A275 L276 E283 M284 Y287 T288 P289 L290 V291 S292 V297 E302 D303 V304 E305 T308 V309 T310 V311 R312 E315 V318 V319 H320 R326 E329 V330 F331 D332 H333 L337 R343 F349 C356 I357 G358 A359 R363 L364 V374 P378 L382 A383 E384 L389 K390 M395 L396 I397 R402 Q403 I404 W407

• Molecule 1: Cytochrome P-450

Chain D: 63% 30% • •

MET THR ASP THR HIS THR GLY PRO THR PRO ALA ASP ALA V14 H23 D26 L27 R35 S41 R42 V43 L54 V55 T56 R57 V64 S71 A75 T76 T80 P81 F84 P90 D91 L94 A95 Q96 D100 H101 R105 R106 L107 K110 A111 F112 R115 M120 R121 P122 R123 V124 R125 V128 D129 S130 L131 L132 V136 L148 A149 V150 P151 F152 P153 V154 A155 V156 E159 V163 L170 F171 R172 T173 F174 S175 M178 L179 S180 S181 T182 R183 L184 T185 E188 I189 Q190 D195 V198 Y199 M200 L203 V204 R208 D209 T212 E213 D214 L215 L216 G217 A218 L221 A222 T223 D224 N225 D226 D227 H228 L229 T230 K231 G232 E233 I234 V235 N236 A244 E247 Q252 I253 T254 N255 L256 R264 K265 S269 L276 V277 P278 E283 M284 L285 R286 V297 S307 T308 V309 T310 V311 R312 V318 E328 E329 V330 F331 L337 P345 H346 F349 H354 H355 C356 I357 G358 A359 R363 L364 E365 R375 R376 F377 L380 D381 L382 A383 E384 P385 V386 K390 G394 M395 L396 I397 L400 E401 R402 Q403 I404 W407

• Molecule 1: Cytochrome P-450

Chain E: % 60% 29% 6% 5%

MET THR ASP THR HIS THR GLY PRO THR PRO ALA ASP ALA V14 S20 L27 D28 P29 H30 R35 R36 D37 E38 P39 V40 S41 R42 V43 R44 L45 P46 Y47 G48 E49 L54 V55 T56 R57 D60 V64 D67 S68 S71 T72 A73 A74 A75 T76 D77 T80 P81 R82 M83 F84 P87 P90 L94 A95 Q96 L104 R105 R106 L107 V108 F112 R115 E118 E119 M120 R121 P122 R123 V124 R125 S126 L127 V128 D129 L132 V136 L148 A149 V150 P151 F152 P153 V154 A155 V156 E159 L170 F171 R172 T173 F174 S175 D176 A177 MET LEU SER SER THR ARG LEU THR A186 A187 E188 I189 Q190 R191 V192 Q193 Q194 D195 V198 Y199 M200 V204 R207 R208 D209 T212 E213 D214 L219 A220 L221 A222 T223 D224 N225 D226 D227 H228 L229 T230 K231 G232 E233 I234 V235 I243 A244 G245 S249 I253 T254 N255 L256

CONFIDENTIAL VALIDATION REPORT

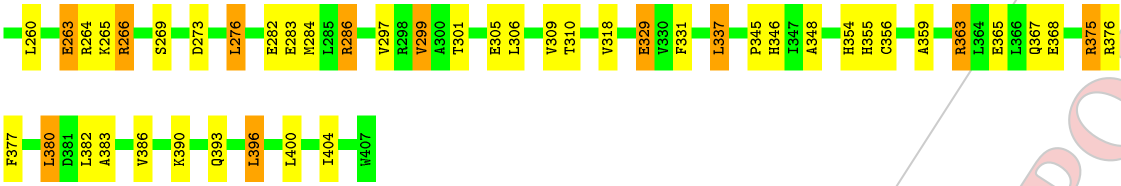

• Molecule 1: Cytochrome P-450

Chain F:

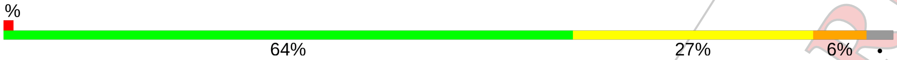

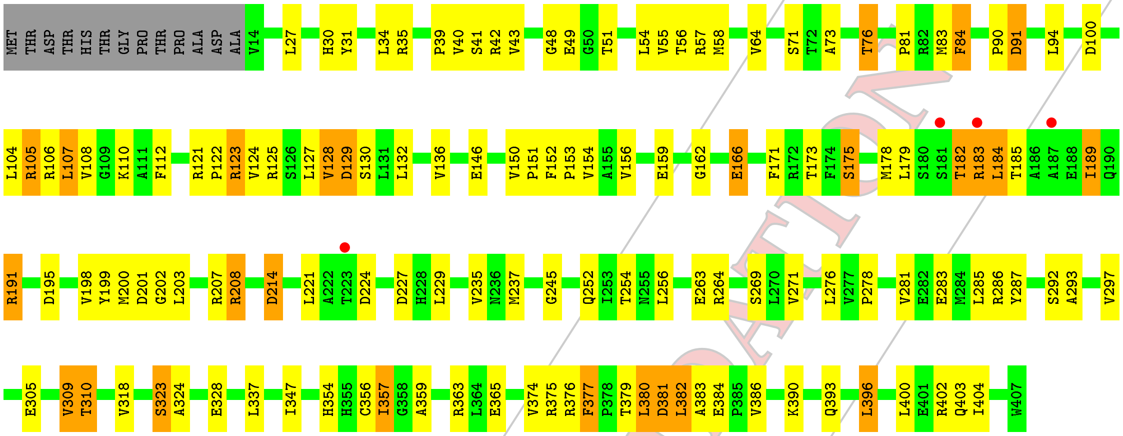

• Molecule 1: Cytochrome P-450

Chain G:

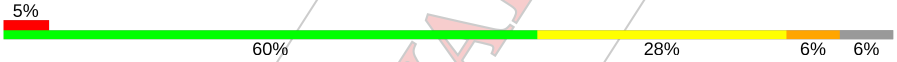

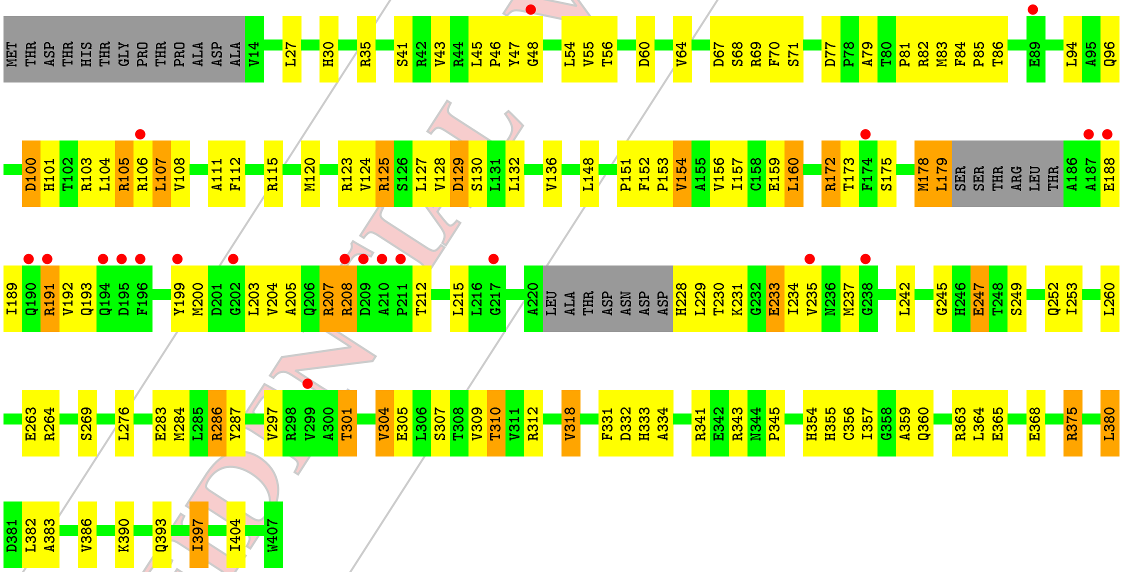

• Molecule 1: Cytochrome P-450

Chain H:

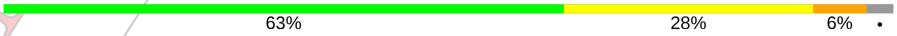

CONFIDENTIAL VALIDATION REPORT

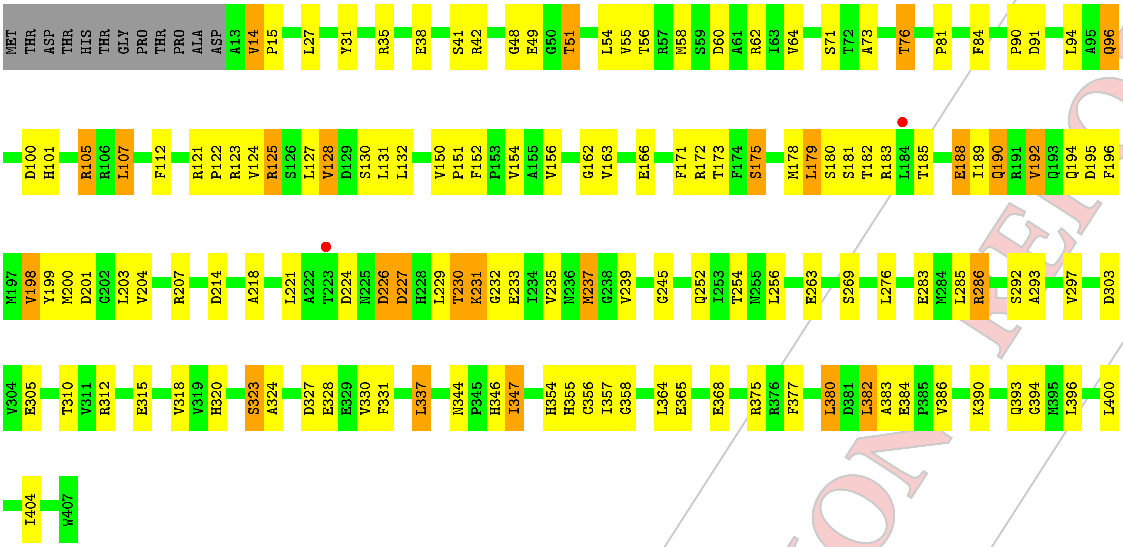

- Molecule 1: Cytochrome P-450

Chain I:
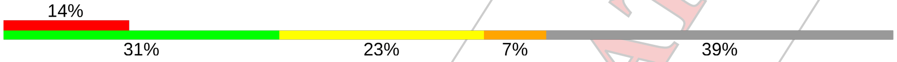

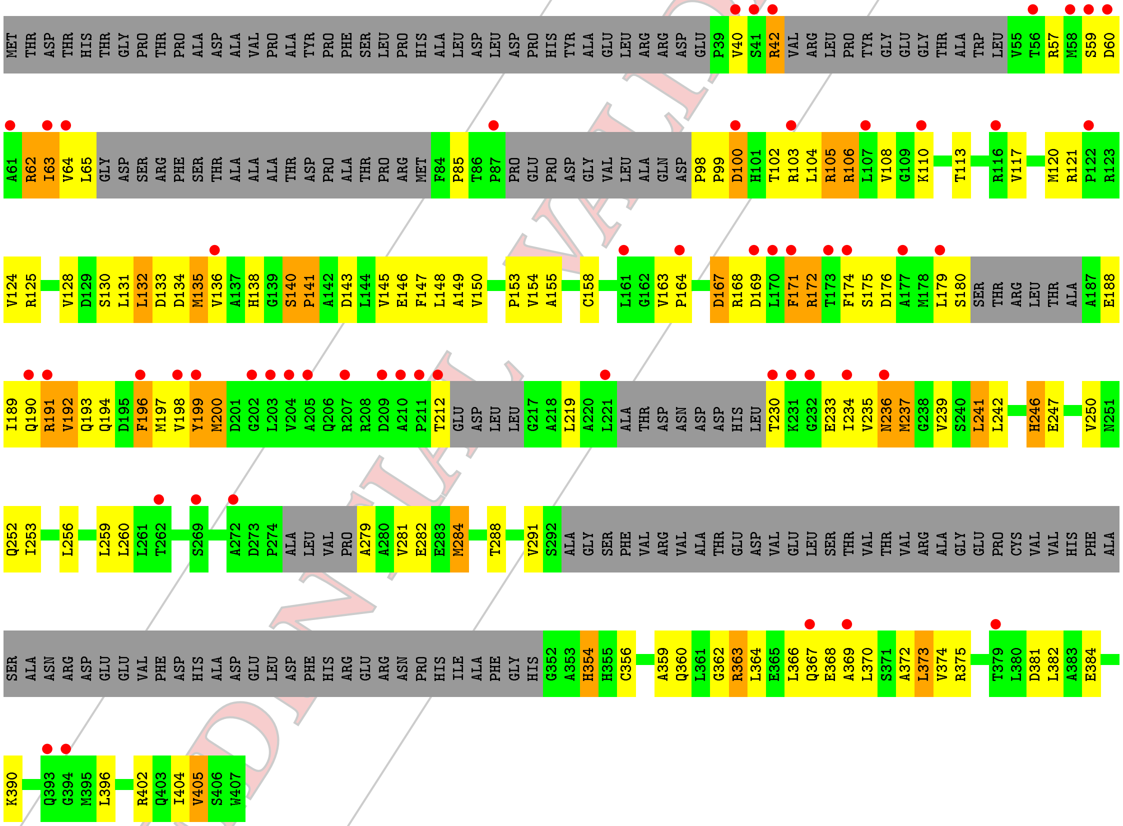

CONFIDENTIAL VALIDATION REPORT

4 Data and refinement statistics (i)

Property	Value	Source
Space group	P 1	Depositor
Cell constants<br>a, b, c, α, β, γ	112.07Å 116.64Å 125.17Å<br>104.43° 104.25° 113.91°	Depositor
Resolution (Å)	39.89 – 2.93<br>39.89 – 2.93	Depositor<br>EDS
% Data completeness<br>(in resolution range)	84.2 (39.89-2.93)<br>84.2 (39.89-2.93)	Depositor<br>EDS
R_{merge}	0.17	Depositor
R_{sym}	(Not available)	Depositor
$< I/\sigma(I) >$ [1]	1.57 (at 2.95Å)	Xtriage
Refinement program	REFMAC 5.8.0238	Depositor
R, R_{free}	0.239 , 0.291<br>0.240 , 0.289	Depositor<br>DCC
R_{free} test set	4684 reflections (5.02%)	wwPDB-VP
Wilson B-factor (Å^2)	44.1	Xtriage
Anisotropy	0.036	Xtriage
Bulk solvent k_{sol}(e/Å^3), B_{sol}(Å^2)	0.28 , 40.0	EDS
L-test for twinning[2]	$< \|L\| > = 0.46$, $< L^2 > = 0.28$	Xtriage
Estimated twinning fraction	0.015 for -k,-h,-l	Xtriage
F_o,F_c correlation	0.88	EDS
Total number of atoms	27067	wwPDB-VP
Average B, all atoms (Å^2)	54.0	wwPDB-VP

Xtriage's analysis on translational NCS is as follows: *The largest off-origin peak in the Patterson function is 3.43% of the height of the origin peak. No significant pseudotranslation is detected.*

[1]Intensities estimated from amplitudes.

[2]Theoretical values of $< |L| >$, $< L^2 >$ for acentric reflections are 0.5, 0.333 respectively for untwinned datasets, and 0.375, 0.2 for perfectly twinned datasets.

CONFIDENTIAL VALIDATION REPORT

5 Model quality (i)

5.1 Standard geometry (i)

Bond lengths and bond angles in the following residue types are not validated in this section: QR8, HEM

The Z score for a bond length (or angle) is the number of standard deviations the observed value is removed from the expected value. A bond length (or angle) with $|Z| > 5$ is considered an outlier worth inspection. RMSZ is the root-mean-square of all Z scores of the bond lengths (or angles).

Mol	Chain	Bond lengths		Bond angles	
		RMSZ	#|Z| >5	RMSZ	#|Z| >5
1	A	0.74	0/3140	0.93	1/4277 (0.0%)
1	B	0.70	0/3145	0.93	1/4284 (0.0%)
1	C	0.72	1/3145 (0.0%)	0.91	1/4284 (0.0%)
1	D	0.73	0/3134	0.90	2/4270 (0.0%)
1	E	0.68	0/3078	0.87	2/4192 (0.0%)
1	F	0.67	0/3140	0.87	0/4277
1	G	0.67	0/3041	0.85	1/4138 (0.0%)
1	H	0.67	0/3145	0.87	1/4284 (0.0%)
1	I	0.84	2/1942 (0.1%)	0.92	2/2622 (0.1%)
All	All	0.71	3/26910 (0.0%)	0.89	11/36628 (0.0%)

Chiral center outliers are detected by calculating the chiral volume of a chiral center and verifying if the center is modelled as a planar moiety or with the opposite hand.A planarity outlier is detected by checking planarity of atoms in a peptide group, atoms in a mainchain group or atoms of a sidechain that are expected to be planar.

Mol	Chain	#Chirality outliers	#Planarity outliers
1	A	0	1
1	B	0	1
1	D	0	1
1	H	0	1
1	I	0	1
All	All	0	5

All (3) bond length outliers are listed below:

Mol	Chain	Res	Type	Atoms	Z	Observed(Å)	Ideal(Å)
1	I	141	PRO	C-O	-17.84	0.87	1.23
1	I	212	THR	C-O	5.61	1.34	1.23
1	C	268	GLU	CD-OE2	5.09	1.31	1.25

CONFIDENTIAL VALIDATION REPORT

All (11) bond angle outliers are listed below:

Mol	Chain	Res	Type	Atoms	Z	Observed(°)	Ideal(°)
1	I	141	PRO	O-C-N	15.29	147.17	122.70
1	I	141	PRO	CA-C-O	-9.18	98.17	120.20
1	E	363	ARG	NE-CZ-NH2	8.08	124.34	120.30
1	E	363	ARG	NE-CZ-NH1	-6.90	116.85	120.30
1	A	57	ARG	NE-CZ-NH1	6.90	123.75	120.30
1	B	326	ARG	NE-CZ-NH1	6.53	123.57	120.30
1	D	375	ARG	NE-CZ-NH2	-6.39	117.10	120.30
1	G	375	ARG	NE-CZ-NH2	-6.25	117.18	120.30
1	C	103	ARG	NE-CZ-NH1	5.49	123.05	120.30
1	H	105	ARG	NE-CZ-NH2	-5.24	117.68	120.30
1	D	375	ARG	NE-CZ-NH1	5.05	122.83	120.30

There are no chirality outliers.

All (5) planarity outliers are listed below:

Mol	Chain	Res	Type	Group
1	A	394	GLY	Peptide
1	B	402	ARG	Sidechain
1	D	394	GLY	Peptide
1	H	394	GLY	Peptide
1	I	140	SER	Peptide

5.2 Too-close contacts ⓘ

In the following table, the Non-H and H(model) columns list the number of non-hydrogen atoms and hydrogen atoms in the chain respectively. The H(added) column lists the number of hydrogen atoms added and optimized by MolProbity. The Clashes column lists the number of clashes within the asymmetric unit, whereas Symm-Clashes lists symmetry related clashes.

Mol	Chain	Non-H	H(model)	H(added)	Clashes	Symm-Clashes
1	A	3073	0	3051	103	0
1	B	3078	0	3056	159	0
1	C	3078	0	3057	115	0
1	D	3067	0	3041	79	0
1	E	3012	0	2983	114	0
1	F	3073	0	3052	95	0
1	G	2976	0	2961	94	0
1	H	3078	0	3057	120	0
1	I	1915	0	1945	123	0
2	A	43	0	30	12	0

Continued on next page...

CONFIDENTIAL VALIDATION REPORT

Continued from previous page...

Mol	Chain	Non-H	H(model)	H(added)	Clashes	Symm-Clashes
2	B	43	0	30	5	0
2	C	43	0	30	3	0
2	D	43	0	30	9	0
2	E	43	0	30	15	0
2	F	43	0	30	11	0
2	G	43	0	30	9	0
2	H	43	0	30	13	0
2	I	43	0	30	6	0
3	A	26	0	0	10	0
3	B	26	0	0	2	0
3	C	26	0	0	1	0
3	D	26	0	0	4	0
3	E	26	0	0	5	0
3	F	26	0	0	4	0
3	G	26	0	0	4	0
3	H	26	0	0	4	0
3	I	26	0	0	5	0
4	A	19	0	0	2	0
4	B	7	0	0	1	0
4	C	14	0	0	1	0
4	D	25	0	0	1	0
4	E	6	0	0	1	0
4	F	7	0	0	2	0
4	G	9	0	0	0	0
4	H	4	0	0	0	0
4	I	5	0	0	1	0
All	All	27067	0	26473	1030	0

The all-atom clashscore is defined as the number of clashes found per 1000 atoms (including hydrogen atoms). The all-atom clashscore for this structure is 19.

All (1030) close contacts within the same asymmetric unit are listed below, sorted by their clash magnitude.

Atom-1	Atom-2	Interatomic distance (Å)	Clash overlap (Å)
1:I:135:MET:CE	1:I:147:PHE:HB2	1.71	1.20
1:B:185:THR:HG21	1:H:166:GLU:OE1	0.99	1.16
1:F:185:THR:O	1:F:189:ILE:HG22	1.45	1.16
1:D:185:THR:HB	1:D:188:GLU:HB2	1.18	1.14
1:B:185:THR:CG2	1:H:166:GLU:OE1	1.94	1.14
1:H:218:ALA:O	1:H:221:LEU:CD2	1.96	1.13
1:E:74:ALA:HB3	1:E:299:VAL:CG2	1.87	1.05

Continued on next page...

CONFIDENTIAL VALIDATION REPORT

Continued from previous page...

Atom-1	Atom-2	Interatomic distance (Å)	Clash overlap (Å)
1:B:104:LEU:O	1:B:107:LEU:HB3	1.58	1.04
1:F:198:VAL:O	1:F:201:ASP:OD1	1.76	1.02
1:D:185:THR:CB	1:D:188:GLU:HB2	1.89	1.02
1:C:40:VAL:HG21	1:C:309:VAL:HG12	1.40	1.01
1:A:42:ARG:HG2	1:A:42:ARG:HH11	1.22	1.00
1:H:185:THR:HG21	1:H:188:GLU:HB2	1.42	0.99
1:I:135:MET:HE3	1:I:147:PHE:HB2	1.44	0.98
1:H:218:ALA:O	1:H:221:LEU:HD23	1.63	0.96
1:B:41:SER:HB3	1:B:54:LEU:CD2	1.96	0.95
1:E:356:CYS:SG	2:E:501:HEM:NC	2.40	0.95
1:B:108:VAL:CG1	1:B:215:LEU:HD23	1.96	0.95
1:D:384:GLU:CD	1:D:402:ARG:HH22	1.71	0.94
1:B:40:VAL:HG21	1:B:309:VAL:HG12	1.46	0.94
1:I:196:PHE:CE1	1:I:239:VAL:HG13	2.02	0.94
1:A:182:THR:O	1:A:185:THR:HG23	1.68	0.93
1:A:223:THR:HG23	1:A:226:ASP:HB2	1.50	0.92
1:B:197:MET:HE1	1:B:235:VAL:HG12	1.53	0.91
1:B:221:LEU:O	1:B:225:ASN:HB3	1.71	0.90
1:I:197:MET:HE1	1:I:236:ASN:HA	1.53	0.90
1:E:74:ALA:HB3	1:E:299:VAL:HG21	1.51	0.90
1:H:224:ASP:O	1:H:226:ASP:OD1	1.89	0.90
1:B:172:ARG:HG2	1:B:172:ARG:HH11	1.37	0.88
1:B:108:VAL:HG11	1:B:215:LEU:HD23	1.55	0.88
1:E:263:GLU:HB3	1:E:266:ARG:HD2	1.54	0.88
1:H:185:THR:CG2	1:H:188:GLU:HB2	2.06	0.86
1:B:237:MET:O	1:B:237:MET:HE3	1.76	0.86
1:H:218:ALA:O	1:H:221:LEU:HD21	1.74	0.86
1:A:105:ARG:NH2	1:A:355:HIS:O	2.09	0.86
1:B:197:MET:HE1	1:B:235:VAL:CG1	2.06	0.86
1:B:40:VAL:HG21	1:B:309:VAL:CG1	2.05	0.85
1:G:157:ILE:HD12	1:G:242:LEU:CD1	2.07	0.84
1:E:356:CYS:SG	2:E:501:HEM:ND	2.49	0.84
1:H:356:CYS:SG	2:H:501:HEM:NC	2.51	0.84
1:E:74:ALA:CB	1:E:299:VAL:HG21	2.09	0.83
1:B:330:VAL:HG13	1:B:331:PHE:CD2	2.13	0.83
1:B:107:LEU:HD12	1:B:107:LEU:O	1.79	0.82
1:B:54:LEU:O	1:B:54:LEU:HD23	1.78	0.82
1:E:231:LYS:O	1:E:234:ILE:HG22	1.80	0.82
1:E:356:CYS:SG	2:E:501:HEM:FE	1.69	0.82
1:B:197:MET:CE	1:B:235:VAL:CG1	2.57	0.82
1:I:369:ALA:O	1:I:373:LEU:HB2	1.79	0.81

Continued on next page...

CONFIDENTIAL VALIDATION REPORT

Continued from previous page...

Atom-1	Atom-2	Interatomic distance (Å)	Clash overlap (Å)
1:B:197:MET:HE3	1:B:235:VAL:HG11	1.60	0.81
1:H:185:THR:HG21	1:H:188:GLU:CB	2.10	0.81
2:E:501:HEM:HBA2	3:E:502:QR8:C32	2.10	0.81
1:G:231:LYS:O	1:G:234:ILE:HG22	1.81	0.81
1:F:356:CYS:SG	2:F:501:HEM:NC	2.53	0.81
1:D:179:LEU:HD11	1:D:247:GLU:HG3	1.62	0.81
1:B:354:HIS:HA	2:B:501:HEM:O2D	1.81	0.80
1:I:190:GLN:O	1:I:194:GLN:HG3	1.81	0.80
1:I:59:SER:O	1:I:63:ILE:HD13	1.81	0.80
1:B:48:GLY:HA3	1:B:81:PRO:HA	1.61	0.80
1:C:42:ARG:NH1	1:C:53:TRP:CZ2	2.50	0.80
2:H:501:HEM:HMB2	2:H:501:HEM:HBB2	1.63	0.79
2:E:501:HEM:HBB2	2:E:501:HEM:HMB2	1.65	0.79
1:B:197:MET:CE	1:B:235:VAL:HG11	2.11	0.79
1:C:92:GLY:HA2	1:C:236:ASN:ND2	1.98	0.79
1:G:356:CYS:SG	2:G:501:HEM:NB	2.54	0.79
1:A:48:GLY:HA3	1:A:81:PRO:HA	1.64	0.78
1:I:155:ALA:HA	1:I:158:CYS:SG	2.23	0.78
1:I:171:PHE:HZ	1:I:242:LEU:HD13	1.47	0.78
1:G:105:ARG:NH1	1:G:355:HIS:O	2.16	0.78
1:I:135:MET:CE	1:I:147:PHE:CB	2.59	0.78
1:F:396:LEU:HD23	3:F:502:QR8:C34	2.14	0.77
1:B:41:SER:HB3	1:B:54:LEU:HD21	1.66	0.77
1:C:284:MET:O	1:C:288:THR:CG2	2.32	0.77
1:B:85:PRO:HG2	1:B:189:ILE:HD12	1.65	0.77
1:A:184:LEU:HD12	1:A:186:ALA:H	1.49	0.77
1:B:183:ARG:C	1:B:184:LEU:HD23	2.05	0.76
2:F:501:HEM:HBB2	2:F:501:HEM:HMB2	1.68	0.76
1:B:221:LEU:O	1:B:225:ASN:CB	2.33	0.76
1:F:396:LEU:CD2	3:F:502:QR8:C34	2.64	0.76
1:A:99:PRO:O	1:A:102:THR:HG22	1.86	0.75
1:B:166:GLU:OE2	1:B:166:GLU:N	2.17	0.75
1:B:104:LEU:O	1:B:107:LEU:CB	2.34	0.75
1:E:82:ARG:HG3	1:E:82:ARG:HH11	1.52	0.75
1:F:40:VAL:HG21	1:F:309:VAL:HB	1.68	0.74
1:A:356:CYS:HB2	2:A:501:HEM:NA	2.02	0.74
1:C:40:VAL:HG21	1:C:309:VAL:CG1	2.18	0.74
1:A:244:ALA:CB	3:A:502:QR8:C31	2.66	0.74
1:F:185:THR:O	1:F:189:ILE:CG2	2.32	0.74
1:E:74:ALA:HB3	1:E:299:VAL:HG23	1.70	0.74
1:A:42:ARG:HH11	1:A:42:ARG:CG	2.01	0.74

Continued on next page...

CONFIDENTIAL VALIDATION REPORT

Continued from previous page...

Atom-1	Atom-2	Interatomic distance (Å)	Clash overlap (Å)
1:E:119:GLU:HB2	4:E:602:HOH:O	1.87	0.74
1:B:96:GLN:NE2	1:B:233:GLU:OE1	2.21	0.74
1:H:194:GLN:O	1:H:198:VAL:HG13	1.88	0.74
1:A:62:ARG:HH11	1:A:62:ARG:HG3	1.53	0.73
3:G:502:QR8:O12	3:G:502:QR8:O1	2.05	0.73
1:E:204:VAL:HG11	1:E:234:ILE:HG23	1.69	0.73
2:A:501:HEM:HBC2	2:A:501:HEM:HMC1	1.71	0.73
1:C:203:LEU:O	1:C:207:ARG:HD2	1.89	0.73
1:E:107:LEU:HD23	1:E:229:LEU:HD23	1.69	0.73
1:H:328:GLU:OE2	1:H:328:GLU:N	2.22	0.73
1:H:185:THR:CG2	1:H:188:GLU:CB	2.66	0.72
1:I:85:PRO:HA	4:I:603:HOH:O	1.87	0.72
1:B:149:ALA:O	1:B:250:VAL:HG23	1.89	0.72
1:D:100:ASP:OD1	1:F:208:ARG:NH2	2.21	0.72
1:G:305:GLU:HG2	1:G:310:THR:HB	1.70	0.72
1:I:197:MET:HE2	1:I:239:VAL:HG21	1.72	0.72
1:E:47:TYR:HB3	1:E:82:ARG:HH12	1.54	0.72
1:G:157:ILE:HD12	1:G:242:LEU:HD13	1.70	0.72
1:C:384:GLU:OE2	1:C:402:ARG:NH1	2.22	0.71
1:I:135:MET:HE2	1:I:147:PHE:HB2	1.69	0.71
1:A:182:THR:O	1:A:185:THR:CG2	2.38	0.71
1:A:191:ARG:O	1:A:195:ASP:OD1	2.06	0.71
1:C:85:PRO:HG2	1:C:189:ILE:HD12	1.72	0.71
1:D:356:CYS:SG	2:D:501:HEM:NC	2.64	0.71
1:A:244:ALA:HB1	3:A:502:QR8:C31	2.21	0.71
1:C:302:GLU:OE2	1:I:402:ARG:NH2	2.24	0.70
1:B:108:VAL:HG12	1:B:215:LEU:HD23	1.71	0.70
1:C:42:ARG:NH1	1:C:53:TRP:CE2	2.58	0.70
1:G:82:ARG:NH1	1:G:85:PRO:O	2.25	0.70
2:A:501:HEM:HBA1	3:A:502:QR8:C32	2.22	0.70
1:F:105:ARG:NH2	1:F:357:ILE:HG12	2.06	0.70
1:G:157:ILE:CD1	1:G:242:LEU:CD1	2.69	0.70
1:E:174:PHE:HZ	1:E:243:ILE:HD11	1.56	0.70
1:C:284:MET:O	1:C:288:THR:HG22	1.91	0.69
1:D:183:ARG:O	1:D:189:ILE:HD11	1.91	0.69
1:I:135:MET:HE3	1:I:147:PHE:CB	2.21	0.69
1:E:263:GLU:O	1:E:266:ARG:HD3	1.92	0.69
1:G:356:CYS:SG	2:G:501:HEM:NC	2.65	0.69
1:B:14:VAL:HG23	1:B:15:PRO:HD2	1.74	0.69
1:B:108:VAL:CG1	1:B:215:LEU:CD2	2.70	0.69
1:E:207:ARG:NH2	1:E:214:ASP:OD2	2.19	0.69

Continued on next page...

CONFIDENTIAL VALIDATION REPORT

Continued from previous page...

Atom-1	Atom-2	Interatomic distance (Å)	Clash overlap (Å)
1:E:282:GLU:OE2	1:E:363:ARG:NH2	2.25	0.69
2:B:501:HEM:HMC2	2:B:501:HEM:HBC2	1.75	0.69
1:G:204:VAL:HG11	1:G:234:ILE:CG2	2.23	0.69
1:D:384:GLU:CD	1:D:402:ARG:NH2	2.46	0.69
1:I:246:HIS:O	1:I:250:VAL:HG23	1.91	0.69
1:G:157:ILE:HD12	1:G:242:LEU:HD12	1.74	0.69
1:F:105:ARG:HH22	1:F:357:ILE:HG12	1.57	0.68
1:G:286:ARG:NH2	1:G:334:ALA:O	2.27	0.68
1:H:312:ARG:N	1:H:315:GLU:OE1	2.25	0.68
1:H:347:ILE:HG22	1:H:347:ILE:O	1.93	0.68
1:B:33:GLU:O	1:B:37:ASP:OD1	2.11	0.68
1:F:182:THR:O	1:F:184:LEU:HD23	1.93	0.68
1:A:96:GLN:HB3	1:A:101:HIS:HB2	1.76	0.68
1:I:200:MET:HA	1:I:200:MET:CE	2.24	0.68
1:I:359:ALA:O	1:I:363:ARG:HD2	1.94	0.68
1:G:100:ASP:N	1:G:100:ASP:OD1	2.19	0.67
1:B:41:SER:HB3	1:B:54:LEU:HD22	1.75	0.67
1:G:230:THR:HB	1:G:233:GLU:HB2	1.76	0.67
1:B:213:GLU:HG3	1:B:213:GLU:O	1.95	0.67
1:C:343:ARG:CZ	1:C:343:ARG:HB3	2.24	0.67
1:F:195:ASP:O	1:F:198:VAL:HG12	1.95	0.67
1:A:230:THR:HG22	1:A:232:GLY:H	1.59	0.67
1:G:204:VAL:HG11	1:G:234:ILE:HG21	1.77	0.67
1:I:219:LEU:HD11	1:I:234:ILE:O	1.94	0.67
1:B:209:ASP:OD1	1:G:355:HIS:HB3	1.95	0.67
1:A:356:CYS:HB2	2:A:501:HEM:C1A	2.31	0.66
1:B:284:MET:O	1:B:288:THR:CG2	2.43	0.66
1:E:47:TYR:HB3	1:E:82:ARG:NH1	2.09	0.66
1:I:250:VAL:O	1:I:253:ILE:HG22	1.95	0.66
1:D:356:CYS:SG	2:D:501:HEM:FE	1.87	0.66
1:D:356:CYS:SG	2:D:501:HEM:ND	2.68	0.66
1:B:237:MET:HE1	1:B:241:LEU:HG	1.77	0.66
1:A:186:ALA:HB3	1:A:188:GLU:HG2	1.78	0.66
1:D:283:GLU:HG3	1:D:337:LEU:CD2	2.26	0.66
1:B:168:ARG:HA	1:B:171:PHE:CE2	2.31	0.66
1:F:356:CYS:SG	2:F:501:HEM:ND	2.69	0.66
1:H:94:LEU:HD12	1:H:354:HIS:CD2	2.31	0.66
1:I:171:PHE:CZ	1:I:242:LEU:HD13	2.31	0.66
2:I:501:HEM:HMB1	2:I:501:HEM:HBB2	1.77	0.65
1:E:396:LEU:H	1:E:396:LEU:HD13	1.61	0.65
1:F:376:ARG:HB2	1:F:377:PHE:CD1	2.31	0.65

Continued on next page...

CONFIDENTIAL VALIDATION REPORT

Continued from previous page...

Atom-1	Atom-2	Interatomic distance (Å)	Clash overlap (Å)
1:H:105:ARG:NH2	1:H:355:HIS:O	2.29	0.65
1:H:231:LYS:HG3	1:H:232:GLY:N	2.10	0.65
1:H:73:ALA:O	1:H:76:THR:OG1	2.14	0.65
1:B:108:VAL:HG11	1:B:215:LEU:CD2	2.26	0.65
1:B:150:VAL:HA	1:B:250:VAL:CG2	2.27	0.65
1:D:185:THR:HB	1:D:188:GLU:CB	2.11	0.65
1:E:148:LEU:O	1:E:151:PRO:HD2	1.97	0.65
1:F:356:CYS:SG	2:F:501:HEM:FE	1.87	0.65
1:I:163:VAL:HG13	1:I:199:TYR:OH	1.97	0.65
1:A:62:ARG:HH11	1:A:62:ARG:CG	2.10	0.65
1:A:62:ARG:HH12	1:A:347:ILE:CG2	2.10	0.65
1:E:223:THR:HB	1:E:226:ASP:HA	1.78	0.65
1:G:203:LEU:O	1:G:207:ARG:HG2	1.97	0.65
2:I:501:HEM:HMC2	2:I:501:HEM:HBC2	1.79	0.65
1:C:252:GLN:HA	1:C:252:GLN:OE1	1.97	0.65
1:H:183:ARG:HB3	1:H:183:ARG:CZ	2.27	0.65
2:D:501:HEM:HBC2	2:D:501:HEM:HMC2	1.78	0.64
1:H:221:LEU:HD23	1:H:221:LEU:H	1.61	0.64
1:I:153:PRO:HG2	1:I:250:VAL:HG22	1.78	0.64
1:I:219:LEU:C	1:I:219:LEU:HD23	2.17	0.64
1:H:356:CYS:SG	2:H:501:HEM:FE	1.88	0.64
1:A:232:GLY:O	1:A:236:ASN:ND2	2.30	0.64
1:B:178:MET:CE	1:B:196:PHE:HD2	2.10	0.64
1:B:222:ALA:O	1:B:226:ASP:HB3	1.97	0.64
1:D:212:THR:HB	1:D:214:ASP:OD1	1.97	0.64
1:A:184:LEU:C	1:A:184:LEU:HD12	2.17	0.64
1:I:189:ILE:HG22	1:I:193:GLN:OE1	1.98	0.64
1:H:356:CYS:SG	2:H:501:HEM:NB	2.71	0.64
1:I:104:LEU:HD12	1:I:105:ARG:N	2.13	0.64
1:D:356:CYS:SG	2:D:501:HEM:NB	2.70	0.64
1:F:73:ALA:O	1:F:76:THR:OG1	2.15	0.64
2:H:501:HEM:HMC1	2:H:501:HEM:HBC2	1.80	0.64
1:D:94:LEU:CD2	3:D:502:QR8:C35	2.76	0.64
1:E:60:ASP:OD2	1:E:306:LEU:HB3	1.98	0.64
1:B:252:GLN:HA	1:B:252:GLN:OE1	1.98	0.64
1:C:200:MET:HG3	1:C:239:VAL:CG1	2.27	0.64
1:D:383:ALA:HB3	1:D:404:ILE:HG22	1.80	0.64
1:G:356:CYS:SG	2:G:501:HEM:FE	1.89	0.63
1:H:183:ARG:HB3	1:H:183:ARG:NH1	2.12	0.63
1:C:149:ALA:O	1:C:250:VAL:HG23	1.98	0.63
1:B:31:TYR:CZ	1:B:320:HIS:ND1	2.67	0.63

Continued on next page...

CONFIDENTIAL VALIDATION REPORT

Continued from previous page...

Atom-1	Atom-2	Interatomic distance (Å)	Clash overlap (Å)
1:C:23:HIS:CE1	1:D:312:ARG:HD2	2.33	0.63
1:E:200:MET:HB3	1:E:235:VAL:HG22	1.80	0.63
1:G:96:GLN:HB3	1:G:101:HIS:HB2	1.80	0.63
1:E:260:LEU:HG	1:E:284:MET:HE1	1.81	0.63
1:F:91:ASP:N	1:F:91:ASP:OD1	2.32	0.63
1:G:157:ILE:CD1	1:G:242:LEU:HD13	2.28	0.63
1:H:356:CYS:SG	2:H:501:HEM:ND	2.71	0.63
1:C:200:MET:HA	1:C:200:MET:CE	2.28	0.63
1:E:213:GLU:OE1	1:E:213:GLU:HA	1.99	0.63
1:B:42:ARG:H	1:B:53:TRP:HB3	1.64	0.62
1:E:329:GLU:OE2	1:E:329:GLU:HA	1.98	0.62
1:C:165:LEU:O	1:C:168:ARG:HG2	1.99	0.62
3:E:502:QR8:O12	3:E:502:QR8:O11	2.15	0.62
1:H:62:ARG:NH1	1:H:347:ILE:HD11	2.15	0.62
1:B:170:LEU:HD22	1:G:115:ARG:HE	1.62	0.62
1:B:172:ARG:HG2	1:B:172:ARG:NH1	2.11	0.62
1:C:55:VAL:HG21	1:C:64:VAL:HG21	1.81	0.62
1:F:328:GLU:N	1:F:328:GLU:OE2	2.33	0.62
1:D:203:LEU:CD1	1:D:216:LEU:HD12	2.30	0.62
3:F:502:QR8:O12	3:F:502:QR8:O1	2.17	0.62
1:H:347:ILE:CG2	1:H:347:ILE:O	2.47	0.62
1:C:236:ASN:O	1:C:239:VAL:HG22	2.00	0.62
1:F:123:ARG:O	1:F:127:LEU:HD23	2.00	0.62
1:C:283:GLU:HG3	1:C:337:LEU:CD2	2.30	0.62
1:I:104:LEU:O	1:I:108:VAL:HG12	1.99	0.62
1:B:152:PHE:HB3	1:B:153:PRO:HD3	1.81	0.62
1:B:178:MET:HE1	1:B:196:PHE:HD2	1.63	0.62
1:G:383:ALA:HB3	1:G:404:ILE:HG22	1.81	0.62
1:H:254:THR:HG21	1:H:400:LEU:HB2	1.81	0.62
1:A:189:ILE:HD12	1:A:190:GLN:N	2.13	0.62
1:I:133:ASP:O	1:I:136:VAL:CG2	2.48	0.62
1:E:230:THR:HB	1:E:233:GLU:H	1.63	0.61
1:C:284:MET:O	1:C:288:THR:HG23	1.98	0.61
1:I:175:SER:HB3	1:I:247:GLU:CD	2.20	0.61
1:I:171:PHE:CE2	1:I:242:LEU:HB3	2.35	0.61
1:H:14:VAL:HG12	1:H:15:PRO:HD2	1.80	0.61
1:H:252:GLN:OE1	1:H:252:GLN:HA	2.00	0.61
1:I:133:ASP:HA	1:I:136:VAL:HG22	1.80	0.61
1:E:107:LEU:HD23	1:E:229:LEU:CD2	2.31	0.61
1:E:82:ARG:HE	1:E:87:PRO:HA	1.65	0.61
1:B:213:GLU:CG	1:B:213:GLU:O	2.48	0.61

Continued on next page...

CONFIDENTIAL VALIDATION REPORT

Continued from previous page...

Atom-1	Atom-2	Interatomic distance (Å)	Clash overlap (Å)
1:F:94:LEU:HD12	1:F:354:HIS:CD2	2.35	0.61
1:C:200:MET:HG3	1:C:239:VAL:HG13	1.82	0.61
1:D:254:THR:HG21	1:D:400:LEU:HB2	1.83	0.61
1:E:254:THR:HG21	1:E:400:LEU:HB2	1.82	0.61
1:B:284:MET:O	1:B:288:THR:HG22	2.01	0.61
1:C:150:VAL:HA	1:C:250:VAL:CG2	2.31	0.61
1:I:402:ARG:HD2	1:I:404:ILE:HD11	1.82	0.61
1:A:160:LEU:HD11	1:A:215:LEU:HD22	1.83	0.61
1:A:254:THR:HG21	1:A:400:LEU:HB2	1.82	0.60
1:B:326:ARG:HH21	1:B:335:ASP:HA	1.66	0.60
1:C:160:LEU:HG	1:C:215:LEU:HD12	1.83	0.60
1:F:83:MET:HE2	1:F:293:ALA:HB1	1.82	0.60
2:A:501:HEM:HBC2	2:A:501:HEM:CMC	2.31	0.60
1:I:150:VAL:O	1:I:154:VAL:HG23	2.02	0.60
2:A:501:HEM:HBB2	2:A:501:HEM:HMB2	1.83	0.60
1:H:327:ASP:O	1:H:330:VAL:HG23	2.02	0.60
1:F:283:GLU:OE2	1:F:286:ARG:NE	2.30	0.60
1:H:221:LEU:H	1:H:221:LEU:CD2	2.15	0.60
1:G:283:GLU:O	1:G:286:ARG:HG2	2.02	0.60
1:E:212:THR:HB	1:E:214:ASP:OD1	2.02	0.59
1:F:183:ARG:HD3	1:F:184:LEU:H	1.66	0.59
1:H:151:PRO:HA	1:H:172:ARG:HH12	1.68	0.59
1:F:354:HIS:CD2	2:F:501:HEM:O1D	2.56	0.59
1:B:237:MET:CE	1:B:241:LEU:HG	2.31	0.59
1:F:30:HIS:CE1	4:F:601:HOH:O	2.56	0.59
1:H:195:ASP:O	1:H:198:VAL:HG22	2.02	0.59
1:H:327:ASP:O	1:H:330:VAL:CG2	2.50	0.59
3:H:502:QR8:O11	3:H:502:QR8:O12	2.20	0.59
1:G:77:ASP:OD1	1:G:79:ALA:HB3	2.03	0.59
1:F:252:GLN:HA	1:F:252:GLN:OE1	2.03	0.59
1:H:125:ARG:HG3	1:H:368:GLU:OE1	2.03	0.59
1:I:62:ARG:CZ	1:I:62:ARG:HB2	2.32	0.59
1:G:360:GLN:OE1	1:G:360:GLN:HA	2.02	0.59
1:B:157:ILE:HG21	1:B:245:GLY:HA3	1.84	0.59
1:F:254:THR:HG21	1:F:400:LEU:HB2	1.85	0.59
2:G:501:HEM:HBB2	2:G:501:HEM:HMB1	1.84	0.59
1:H:131:LEU:HD21	1:H:151:PRO:HB2	1.84	0.59
1:A:123:ARG:NE	1:A:159:GLU:OE1	2.31	0.58
1:C:152:PHE:HB3	1:C:153:PRO:HD3	1.84	0.58
1:B:171:PHE:O	1:B:175:SER:OG	2.20	0.58
1:G:48:GLY:HA3	1:G:81:PRO:HA	1.86	0.58

Continued on next page...

CONFIDENTIAL VALIDATION REPORT

Continued from previous page...

Atom-1	Atom-2	Interatomic distance (Å)	Clash overlap (Å)
1:E:125:ARG:HG3	1:E:368:GLU:OE2	2.04	0.58
1:A:200:MET:HB3	1:A:235:VAL:HG13	1.85	0.58
1:C:48:GLY:HA3	1:C:81:PRO:HA	1.84	0.58
2:G:501:HEM:HMC2	2:G:501:HEM:HBC2	1.86	0.58
1:B:283:GLU:HG3	1:B:337:LEU:CD2	2.34	0.58
1:F:354:HIS:HD2	2:F:501:HEM:O1D	1.85	0.58
1:G:125:ARG:HG3	1:G:368:GLU:OE2	2.03	0.58
1:H:127:LEU:O	1:H:131:LEU:HD13	2.03	0.58
1:A:223:THR:HG23	1:A:226:ASP:CB	2.28	0.58
1:E:396:LEU:N	1:E:396:LEU:HD13	2.19	0.58
1:F:376:ARG:HB2	1:F:377:PHE:HD1	1.68	0.58
1:G:152:PHE:HB3	1:G:153:PRO:HD3	1.85	0.58
1:C:108:VAL:HG11	1:C:241:LEU:HD11	1.85	0.58
1:C:196:PHE:CZ	1:C:242:LEU:HD23	2.39	0.57
1:D:223:THR:HB	1:D:226:ASP:HB2	1.86	0.57
1:F:48:GLY:HA3	1:F:81:PRO:HA	1.86	0.57
1:A:356:CYS:SG	2:A:501:HEM:C4C	2.96	0.57
1:E:36:ARG:HD3	1:E:37:ASP:OD1	2.04	0.57
1:I:106:ARG:HH21	1:I:110:LYS:HG2	1.68	0.57
1:B:237:MET:HE3	1:B:237:MET:C	2.24	0.57
1:I:196:PHE:CD1	1:I:239:VAL:HG13	2.39	0.57
1:D:94:LEU:HD12	1:D:354:HIS:CD2	2.39	0.57
1:E:191:ARG:HD3	1:E:191:ARG:C	2.25	0.57
1:A:133:ASP:OD1	1:A:376:ARG:NH2	2.36	0.57
1:B:383:ALA:HB3	1:B:404:ILE:HG22	1.86	0.57
1:D:230:THR:HB	1:D:233:GLU:H	1.68	0.57
1:E:356:CYS:SG	2:E:501:HEM:NB	2.77	0.57
1:H:221:LEU:HD23	1:H:221:LEU:N	2.18	0.57
1:A:330:VAL:HG22	1:C:272:ALA:HB1	1.86	0.57
1:B:166:GLU:H	1:B:166:GLU:CD	2.08	0.57
1:C:103:ARG:O	1:C:107:LEU:HG	2.04	0.57
1:I:133:ASP:O	1:I:136:VAL:HG22	2.05	0.57
2:B:501:HEM:CMC	2:B:501:HEM:HBC2	2.34	0.57
1:C:66:GLY:O	1:I:138:HIS:CE1	2.57	0.56
1:F:124:VAL:O	1:F:128:VAL:HG23	2.05	0.56
1:G:245:GLY:HA2	2:G:501:HEM:C2C	2.40	0.56
1:A:203:LEU:O	1:A:207:ARG:HG2	2.06	0.56
1:B:231:LYS:C	1:B:231:LYS:HD2	2.25	0.56
1:C:93:VAL:HG11	1:C:237:MET:CE	2.35	0.56
1:D:252:GLN:OE1	1:D:252:GLN:HA	2.05	0.56
1:E:47:TYR:CB	1:E:82:ARG:NH1	2.68	0.56

Continued on next page...

CONFIDENTIAL VALIDATION REPORT

Continued from previous page...

Atom-1	Atom-2	Interatomic distance (Å)	Clash overlap (Å)
1:G:104:LEU:O	1:G:108:VAL:HG13	2.04	0.56
1:G:200:MET:HB3	1:G:235:VAL:HG13	1.86	0.56
1:E:82:ARG:CG	1:E:82:ARG:HH11	2.18	0.56
2:E:501:HEM:CMB	2:E:501:HEM:HBB2	2.34	0.56
1:G:172:ARG:HH21	1:G:172:ARG:CG	2.19	0.56
1:B:184:LEU:HD23	1:B:184:LEU:N	2.17	0.56
1:B:53:TRP:N	1:B:53:TRP:CD1	2.71	0.56
1:F:40:VAL:CG2	1:F:309:VAL:HB	2.35	0.56
1:A:81:PRO:O	1:A:297:VAL:HG21	2.06	0.56
1:D:208:ARG:NH2	1:F:100:ASP:OD1	2.39	0.56
1:C:168:ARG:HA	1:C:171:PHE:CE2	2.39	0.56
1:D:35:ARG:HG3	1:D:56:THR:HG22	1.88	0.56
1:B:326:ARG:HH21	1:B:335:ASP:CB	2.19	0.56
1:E:55:VAL:HG21	1:E:64:VAL:HG21	1.87	0.56
1:I:281:VAL:O	1:I:281:VAL:HG12	2.06	0.55
1:B:284:MET:O	1:B:288:THR:HG23	2.07	0.55
1:I:359:ALA:O	1:I:363:ARG:CD	2.54	0.55
3:B:502:QR8:C33	3:B:502:QR8:C32	2.85	0.55
1:E:75:ALA:HB1	1:E:297:VAL:HG13	1.88	0.55
1:G:204:VAL:CG1	1:G:234:ILE:HG21	2.36	0.55
1:A:184:LEU:CD1	1:A:186:ALA:H	2.17	0.55
1:A:42:ARG:HG2	1:A:42:ARG:NH1	2.02	0.55
3:D:502:QR8:C31	3:D:502:QR8:C30	2.85	0.55
1:F:356:CYS:SG	2:F:501:HEM:NB	2.79	0.55
1:H:293:ALA:O	1:H:320:HIS:ND1	2.40	0.55
1:I:360:GLN:OE1	1:I:360:GLN:HA	2.07	0.55
3:I:502:QR8:C33	3:I:502:QR8:C32	2.85	0.55
2:E:501:HEM:CBA	3:E:502:QR8:C32	2.83	0.55
1:G:286:ARG:HG3	1:G:287:TYR:N	2.21	0.55
2:I:501:HEM:HBA2	3:I:502:QR8:O12	2.07	0.55
3:A:502:QR8:C34	3:A:502:QR8:C35	2.85	0.55
1:B:120:MET:C	1:B:122:PRO:HD2	2.27	0.55
1:D:84:PHE:CD1	1:D:84:PHE:N	2.75	0.55
1:E:375:ARG:HH11	1:E:375:ARG:HG3	1.72	0.55
1:H:48:GLY:HA3	1:H:81:PRO:HA	1.89	0.55
1:B:327:ASP:OD1	1:B:329:GLU:HB2	2.07	0.55
1:B:272:ALA:HB1	1:D:330:VAL:HG22	1.87	0.55
1:A:73:ALA:O	1:A:76:THR:OG1	2.19	0.55
1:B:42:ARG:HA	1:B:52:ALA:O	2.07	0.55
1:C:73:ALA:O	1:C:76:THR:OG1	2.24	0.55
1:G:84:PHE:CE1	3:G:502:QR8:C34	2.90	0.55

Continued on next page...

CONFIDENTIAL VALIDATION REPORT

Continued from previous page...

Atom-1	Atom-2	Interatomic distance (Å)	Clash overlap (Å)
1:C:154:VAL:O	1:C:157:ILE:HG22	2.08	0.54
2:F:501:HEM:HMC2	2:F:501:HEM:HBC2	1.89	0.54
1:G:252:GLN:HA	1:G:252:GLN:OE1	2.06	0.54
3:H:502:QR8:C1	3:H:502:QR8:C11	2.85	0.54
1:A:203:LEU:HD11	1:A:207:ARG:NH2	2.23	0.54
1:B:235:VAL:O	1:B:238:GLY:N	2.40	0.54
1:F:191:ARG:HH11	1:F:191:ARG:CG	2.19	0.54
1:G:179:LEU:HB2	1:G:247:GLU:OE1	2.07	0.54
1:G:286:ARG:HH12	1:G:341:ARG:NH2	2.04	0.54
1:I:98:PRO:HA	1:I:99:PRO:C	2.27	0.54
1:A:283:GLU:HG3	1:A:337:LEU:CD2	2.38	0.54
1:C:207:ARG:HE	1:C:216:LEU:HB3	1.73	0.54
3:G:502:QR8:C1	3:G:502:QR8:C11	2.85	0.54
1:H:124:VAL:O	1:H:128:VAL:HG23	2.07	0.54
1:H:81:PRO:O	1:H:297:VAL:HG21	2.07	0.54
1:B:271:VAL:HA	1:B:374:VAL:HG13	1.90	0.54
1:G:69:ARG:CZ	1:G:304:VAL:HG12	2.38	0.54
1:A:252:GLN:HA	1:A:252:GLN:OE1	2.07	0.54
1:E:174:PHE:CZ	1:E:243:ILE:HD11	2.40	0.54
1:D:107:LEU:HD12	1:D:229:LEU:CD2	2.38	0.54
1:E:75:ALA:CB	1:E:297:VAL:HG13	2.38	0.54
1:G:67:ASP:OD1	1:G:68:SER:N	2.41	0.54
1:H:218:ALA:C	1:H:221:LEU:CD2	2.74	0.54
1:G:124:VAL:O	1:G:128:VAL:HG23	2.08	0.54
1:H:107:LEU:HD23	1:H:229:LEU:HD23	1.89	0.54
3:I:502:QR8:C30	3:I:502:QR8:C31	2.86	0.54
3:E:502:QR8:C33	3:E:502:QR8:C34	2.85	0.54
2:A:501:HEM:CBA	3:A:502:QR8:C32	2.86	0.54
1:F:200:MET:HB3	1:F:235:VAL:HG13	1.90	0.54
1:I:135:MET:HE2	1:I:147:PHE:CG	2.43	0.54
1:I:135:MET:HE2	1:I:147:PHE:CB	2.33	0.53
1:I:175:SER:O	1:I:179:LEU:CD2	2.55	0.53
1:I:189:ILE:HA	1:I:192:VAL:CG2	2.39	0.53
3:A:502:QR8:C32	3:A:502:QR8:C33	2.85	0.53
1:A:99:PRO:HA	1:A:102:THR:HG22	1.89	0.53
1:D:152:PHE:HB3	1:D:153:PRO:HD3	1.90	0.53
1:I:256:LEU:HD11	1:I:366:LEU:HD13	1.89	0.53
1:A:328:GLU:N	1:A:328:GLU:OE2	2.40	0.53
1:C:194:GLN:O	1:C:198:VAL:HG23	2.09	0.53
1:C:200:MET:CE	1:C:203:LEU:HD12	2.38	0.53
3:H:502:QR8:C35	3:H:502:QR8:C34	2.85	0.53

Continued on next page...

CONFIDENTIAL VALIDATION REPORT

Continued from previous page...

Atom-1	Atom-2	Interatomic distance (Å)	Clash overlap (Å)
1:I:164:PRO:HG2	1:I:167:ASP:CG	2.29	0.53
1:I:179:LEU:CD1	1:I:396:LEU:HD12	2.39	0.53
3:I:502:QR8:C35	3:I:502:QR8:C34	2.85	0.53
1:A:194:GLN:O	1:A:198:VAL:HG22	2.07	0.53
1:B:237:MET:HE1	1:B:241:LEU:CG	2.39	0.53
1:C:178:MET:HE1	1:C:193:GLN:HG2	1.90	0.53
1:I:384:GLU:OE1	1:I:402:ARG:NH1	2.40	0.53
1:A:184:LEU:HD12	1:A:186:ALA:N	2.19	0.53
1:B:124:VAL:O	1:B:128:VAL:HG23	2.08	0.53
1:C:124:VAL:O	1:C:128:VAL:HG23	2.08	0.53
1:C:26:ASP:OD2	1:D:312:ARG:NH1	2.41	0.53
1:G:103:ARG:O	1:G:107:LEU:HG	2.07	0.53
1:G:191:ARG:C	1:G:191:ARG:HD2	2.29	0.53
1:G:70:PHE:CZ	1:G:304:VAL:HG21	2.44	0.53
1:E:245:GLY:HA2	2:E:501:HEM:C2C	2.44	0.53
1:F:292:SER:O	1:F:293:ALA:HB3	2.09	0.53
1:I:197:MET:HE1	1:I:236:ASN:CA	2.34	0.53
1:I:260:LEU:HD13	1:I:284:MET:CE	2.39	0.53
1:A:245:GLY:HA2	2:A:501:HEM:C2C	2.43	0.53
1:E:283:GLU:HG3	1:E:337:LEU:CD2	2.39	0.53
1:F:104:LEU:O	1:F:108:VAL:HG13	2.09	0.53
1:A:152:PHE:HB3	1:A:153:PRO:HD3	1.90	0.53
1:F:383:ALA:HB3	1:F:404:ILE:HG22	1.90	0.53
1:H:151:PRO:HB3	1:H:172:ARG:HH12	1.74	0.53
1:H:42:ARG:NH1	1:H:51:THR:HG23	2.24	0.52
1:F:376:ARG:CB	1:F:377:PHE:CD1	2.92	0.52
1:F:183:ARG:HD3	1:F:184:LEU:N	2.24	0.52
2:F:501:HEM:HBB2	2:F:501:HEM:CMB	2.38	0.52
1:G:94:LEU:HD12	1:G:354:HIS:CD2	2.44	0.52
1:H:200:MET:HB3	1:H:235:VAL:HG13	1.92	0.52
1:I:131:LEU:HD13	1:I:134:ASP:OD2	2.09	0.52
1:I:370:LEU:O	1:I:374:VAL:HG23	2.10	0.52
1:I:59:SER:O	1:I:62:ARG:HG3	2.09	0.52
1:B:183:ARG:NH1	1:B:393:GLN:O	2.42	0.52
1:E:124:VAL:O	1:E:128:VAL:HG23	2.10	0.52
1:B:105:ARG:HD3	1:B:105:ARG:C	2.30	0.52
1:B:170:LEU:HD11	1:B:174:PHE:CZ	2.44	0.52
1:B:172:ARG:HH11	1:B:172:ARG:CG	2.18	0.52
1:B:230:THR:O	1:B:233:GLU:HB2	2.07	0.52
1:C:76:THR:HG21	1:C:90:PRO:HA	1.92	0.52
1:D:55:VAL:HG21	1:D:64:VAL:HG21	1.92	0.52

Continued on next page...

CONFIDENTIAL VALIDATION REPORT

Continued from previous page...

Atom-1	Atom-2	Interatomic distance (Å)	Clash overlap (Å)
1:E:105:ARG:NH2	1:E:355:HIS:O	2.42	0.52
1:F:55:VAL:HG21	1:F:64:VAL:HG21	1.91	0.52
1:H:179:LEU:O	1:H:182:THR:HB	2.09	0.52
1:B:125:ARG:HG2	1:B:368:GLU:OE2	2.10	0.52
1:C:208:ARG:HG2	1:C:209:ASP:N	2.24	0.52
1:D:247:GLU:OE2	1:D:397:ILE:HD12	2.09	0.52
1:G:123:ARG:O	1:G:127:LEU:HD13	2.08	0.52
1:H:286:ARG:HD3	1:H:344:ASN:HD22	1.74	0.52
1:H:245:GLY:HA2	2:H:501:HEM:C2C	2.45	0.52
1:I:65:LEU:CD2	1:I:354:HIS:H	2.23	0.52
1:C:196:PHE:HZ	1:C:242:LEU:HD23	1.75	0.52
1:D:107:LEU:HD12	1:D:229:LEU:HD23	1.91	0.52
1:I:121:ARG:NH1	1:I:368:GLU:OE1	2.43	0.52
1:A:213:GLU:OE1	1:A:213:GLU:HA	2.10	0.52
1:E:74:ALA:CB	1:E:299:VAL:CG2	2.70	0.52
1:I:356:CYS:SG	2:I:501:HEM:C4B	3.02	0.52
1:B:121:ARG:N	1:B:122:PRO:HD2	2.24	0.52
1:B:135:MET:CE	1:B:144:LEU:HA	2.41	0.52
1:C:349:PHE:CE1	1:C:359:ALA:HA	2.44	0.52
1:A:184:LEU:HD12	1:A:185:THR:N	2.26	0.51
1:C:148:LEU:O	1:C:151:PRO:HD2	2.10	0.51
1:G:207:ARG:HA	1:G:207:ARG:HH11	1.75	0.51
1:A:186:ALA:HB3	1:A:188:GLU:CG	2.40	0.51
1:B:326:ARG:NH2	1:B:335:ASP:HB3	2.26	0.51
1:C:40:VAL:HG22	1:C:308:THR:HG23	1.92	0.51
1:E:263:GLU:CB	1:E:266:ARG:HD2	2.33	0.51
1:I:120:MET:O	1:I:124:VAL:HG23	2.09	0.51
1:E:132:LEU:O	1:E:136:VAL:HG23	2.10	0.51
1:G:260:LEU:HG	1:G:284:MET:HE1	1.92	0.51
1:H:35:ARG:HG3	1:H:56:THR:HG22	1.93	0.51
1:B:42:ARG:CG	1:B:53:TRP:CE3	2.93	0.51
1:E:282:GLU:CD	1:E:363:ARG:HH21	2.12	0.51
1:H:151:PRO:HA	1:H:172:ARG:NH1	2.25	0.51
1:H:55:VAL:HG21	1:H:64:VAL:HG21	1.92	0.51
1:B:35:ARG:HD3	1:B:327:ASP:HB2	1.93	0.51
1:C:135:MET:CE	1:C:144:LEU:HA	2.41	0.51
1:D:355:HIS:O	1:D:356:CYS:C	2.49	0.51
1:D:132:LEU:HD22	1:D:377:PHE:HE2	1.75	0.51
1:H:31:TYR:HB3	1:H:323:SER:OG	2.10	0.51
1:G:172:ARG:HG2	1:G:172:ARG:HH21	1.75	0.51
1:G:55:VAL:HG21	1:G:64:VAL:HG21	1.93	0.51

Continued on next page...

CONFIDENTIAL VALIDATION REPORT

Continued from previous page...

Atom-1	Atom-2	Interatomic distance (Å)	Clash overlap (Å)
1:H:185:THR:HG23	1:H:188:GLU:CB	2.41	0.51
1:I:132:LEU:HD11	1:I:148:LEU:HD22	1.93	0.51
1:C:104:LEU:HD13	1:C:229:LEU:CD2	2.40	0.51
1:C:396:LEU:HD13	3:C:502:QR8:C36	2.40	0.51
2:H:501:HEM:HBB2	2:H:501:HEM:CMB	2.36	0.51
1:I:132:LEU:CD1	1:I:148:LEU:HD22	2.40	0.51
1:E:121:ARG:NH2	1:E:367:GLN:OE1	2.44	0.51
1:H:150:VAL:HB	1:H:151:PRO:HD3	1.93	0.51
1:B:235:VAL:HG12	1:B:236:ASN:N	2.26	0.50
1:B:42:ARG:N	1:B:53:TRP:HB3	2.27	0.50
1:C:46:PRO:HB2	1:C:47:TYR:CD1	2.47	0.50
1:D:331:PHE:CE1	1:D:345:PRO:HD2	2.47	0.50
1:E:132:LEU:HG	1:E:377:PHE:HE2	1.76	0.50
1:B:260:LEU:HG	1:B:284:MET:HE1	1.93	0.50
1:G:83:MET:CE	1:G:318:VAL:HG21	2.41	0.50
1:B:326:ARG:NH2	1:B:335:ASP:CB	2.74	0.50
1:E:77:ASP:HB3	1:E:80:THR:HG1	1.76	0.50
1:B:161:LEU:HD12	1:B:215:LEU:HB2	1.93	0.50
1:D:195:ASP:O	1:D:198:VAL:HG12	2.11	0.50
1:F:83:MET:CE	1:F:293:ALA:HB1	2.42	0.50
1:G:69:ARG:O	1:G:301:THR:OG1	2.20	0.50
1:H:382:LEU:HD22	1:H:384:GLU:O	2.11	0.50
1:I:132:LEU:O	1:I:135:MET:HB3	2.10	0.50
1:I:252:GLN:HG2	1:I:366:LEU:HD11	1.94	0.50
1:A:172:ARG:CG	1:A:172:ARG:HH21	2.23	0.50
1:A:99:PRO:CA	1:A:102:THR:HG22	2.41	0.50
1:B:203:LEU:O	1:B:207:ARG:HG2	2.12	0.50
1:C:271:VAL:HA	1:C:374:VAL:HG13	1.94	0.50
1:F:245:GLY:HA2	2:F:501:HEM:C2C	2.47	0.50
1:G:179:LEU:HD12	1:G:179:LEU:C	2.32	0.50
1:G:189:ILE:O	1:G:193:GLN:HG3	2.11	0.50
1:A:359:ALA:O	1:A:363:ARG:HG3	2.12	0.50
1:B:200:MET:HG3	1:B:239:VAL:HG22	1.94	0.50
1:E:383:ALA:HB3	1:E:404:ILE:HG22	1.94	0.50
1:H:84:PHE:CE1	3:H:502:QR8:C34	2.95	0.50
1:B:54:LEU:HA	1:B:318:VAL:O	2.12	0.49
1:C:179:LEU:CD1	1:C:247:GLU:HG3	2.42	0.49
1:D:402:ARG:HH21	1:D:402:ARG:HG2	1.77	0.49
1:G:35:ARG:HG3	1:G:56:THR:HG22	1.94	0.49
1:E:48:GLY:HA3	1:E:81:PRO:HA	1.94	0.49
1:I:174:PHE:HB3	1:I:196:PHE:HD2	1.77	0.49

Continued on next page...

CONFIDENTIAL VALIDATION REPORT

Continued from previous page...

Atom-1	Atom-2	Interatomic distance (Å)	Clash overlap (Å)
3:I:502:QR8:C9	3:I:502:QR8:C32	2.90	0.49
1:B:277:VAL:HB	1:B:278:PRO:HD3	1.94	0.49
1:B:55:VAL:HG21	1:B:64:VAL:HG21	1.93	0.49
1:E:375:ARG:HH11	1:E:375:ARG:CG	2.26	0.49
1:G:154:VAL:O	1:G:157:ILE:HG12	2.12	0.49
1:H:327:ASP:HB3	1:H:330:VAL:HG21	1.92	0.49
1:B:73:ALA:HA	1:B:95:ALA:O	2.12	0.49
1:C:200:MET:HB3	1:C:235:VAL:HG13	1.94	0.49
1:D:185:THR:O	1:D:189:ILE:HG13	2.12	0.49
1:D:200:MET:HB3	1:D:235:VAL:HG13	1.94	0.49
1:H:58:MET:HE2	1:H:324:ALA:HB1	1.95	0.49
1:I:197:MET:CE	1:I:239:VAL:HG21	2.41	0.49
1:A:106:ARG:HH12	1:A:110:LYS:HE3	1.76	0.49
1:A:230:THR:HB	1:A:233:GLU:H	1.77	0.49
1:B:326:ARG:HH21	1:B:335:ASP:CA	2.24	0.49
1:I:106:ARG:NH2	1:I:110:LYS:HA	2.28	0.49
1:I:98:PRO:HB2	1:I:99:PRO:HA	1.95	0.49
1:F:245:GLY:HA2	2:F:501:HEM:C1C	2.48	0.49
1:G:356:CYS:SG	2:G:501:HEM:ND	2.86	0.49
1:I:133:ASP:O	1:I:136:VAL:HG23	2.12	0.49
1:A:55:VAL:HG21	1:A:64:VAL:HG21	1.95	0.49
1:B:121:ARG:N	1:B:122:PRO:CD	2.76	0.49
1:B:200:MET:HG3	1:B:239:VAL:CG2	2.42	0.49
1:B:330:VAL:CG1	1:B:331:PHE:CD2	2.91	0.49
1:C:108:VAL:HG13	1:C:215:LEU:CD2	2.43	0.49
1:H:327:ASP:HB3	1:H:330:VAL:CG2	2.43	0.49
1:A:245:GLY:HA2	2:A:501:HEM:C1C	2.47	0.49
1:C:402:ARG:HG2	1:C:403:GLN:N	2.27	0.49
1:D:96:GLN:HB3	1:D:101:HIS:HB2	1.95	0.49
1:I:99:PRO:O	1:I:102:THR:N	2.44	0.49
2:H:501:HEM:CMC	2:H:501:HEM:HBC2	2.43	0.49
1:B:60:ASP:OD1	1:B:307:SER:HB3	2.13	0.48
1:E:229:LEU:CD1	1:E:233:GLU:HG3	2.43	0.48
1:G:172:ARG:NH2	1:G:172:ARG:HG2	2.27	0.48
1:H:283:GLU:HG3	1:H:337:LEU:CD2	2.43	0.48
1:I:189:ILE:HD12	1:I:189:ILE:N	2.27	0.48
1:B:200:MET:CG	1:B:239:VAL:HG22	2.43	0.48
1:D:23:HIS:CD2	1:D:26:ASP:OD1	2.66	0.48
1:E:359:ALA:O	1:E:363:ARG:HG3	2.13	0.48
1:C:185:THR:HG22	1:F:166:GLU:OE1	2.13	0.48
1:H:151:PRO:CA	1:H:172:ARG:HH12	2.25	0.48

Continued on next page...

CONFIDENTIAL VALIDATION REPORT

Continued from previous page...

Atom-1	Atom-2	Interatomic distance (Å)	Clash overlap (Å)
1:I:190:GLN:O	1:I:194:GLN:CG	2.56	0.48
1:I:219:LEU:HD21	1:I:234:ILE:O	2.13	0.48
1:A:198:VAL:O	1:A:201:ASP:HB2	2.12	0.48
1:C:260:LEU:HG	1:C:284:MET:HE1	1.95	0.48
1:E:207:ARG:HH21	1:E:214:ASP:CG	2.10	0.48
1:I:172:ARG:O	1:I:176:ASP:CG	2.52	0.48
1:A:195:ASP:O	1:A:198:VAL:HG23	2.14	0.48
1:C:176:ASP:OD1	1:C:247:GLU:OE2	2.32	0.48
1:C:260:LEU:HG	1:C:284:MET:CE	2.43	0.48
1:D:375:ARG:NH1	4:D:601:HOH:O	2.38	0.48
1:H:162:GLY:HA3	1:H:214:ASP:OD2	2.13	0.48
1:A:124:VAL:O	1:A:128:VAL:HG23	2.14	0.48
1:C:101:HIS:CE1	1:C:105:ARG:HG3	2.49	0.48
1:C:93:VAL:HG11	1:C:237:MET:SD	2.53	0.48
1:D:150:VAL:HB	1:D:151:PRO:HD3	1.95	0.48
1:C:209:ASP:OD1	1:E:105:ARG:NH2	2.46	0.48
1:E:94:LEU:HD12	1:E:354:HIS:CD2	2.48	0.48
1:H:283:GLU:OE2	1:H:286:ARG:NH2	2.45	0.48
1:I:100:ASP:HA	1:I:103:ARG:CD	2.43	0.48
1:I:141:PRO:HB3	1:I:405:VAL:O	2.14	0.48
1:C:200:MET:HE2	1:C:200:MET:HA	1.95	0.48
1:C:66:GLY:O	1:I:138:HIS:HE1	1.96	0.48
1:E:396:LEU:HG	3:E:502:QR8:C36	2.43	0.48
1:I:146:GLU:HG2	1:I:147:PHE:CD1	2.47	0.48
1:I:65:LEU:HD23	1:I:354:HIS:H	1.78	0.48
1:B:104:LEU:HG	1:B:237:MET:HG3	1.94	0.48
1:C:287:TYR:O	1:C:326:ARG:NH1	2.46	0.48
1:E:264:ARG:NH2	1:E:380:LEU:O	2.40	0.48
1:I:279:ALA:HA	1:I:282:GLU:HG2	1.95	0.48
1:A:200:MET:O	1:A:204:VAL:HG23	2.13	0.48
1:C:230:THR:HB	1:C:233:GLU:H	1.78	0.48
1:D:384:GLU:OE1	1:D:402:ARG:NH2	2.47	0.48
1:F:377:PHE:O	1:F:380:LEU:HB2	2.13	0.48
1:B:208:ARG:NH2	1:G:355:HIS:NE2	2.62	0.48
1:H:330:VAL:HG23	1:H:331:PHE:CD2	2.49	0.48
1:H:254:THR:CG2	1:H:400:LEU:HB2	2.44	0.48
1:A:62:ARG:NH1	1:A:62:ARG:CG	2.72	0.47
1:C:108:VAL:HG13	1:C:215:LEU:HD22	1.94	0.47
1:F:201:ASP:OD1	1:F:202:GLY:N	2.47	0.47
1:H:185:THR:CG2	1:H:188:GLU:HB3	2.41	0.47
1:H:196:PHE:CE1	1:H:200:MET:HG2	2.49	0.47

Continued on next page...

CONFIDENTIAL VALIDATION REPORT

Continued from previous page...

Atom-1	Atom-2	Interatomic distance (Å)	Clash overlap (Å)
1:I:121:ARG:NH2	1:I:367:GLN:OE1	2.46	0.47
1:I:149:ALA:HA	1:I:253:ILE:HG21	1.95	0.47
1:A:150:VAL:HB	1:A:151:PRO:HD3	1.96	0.47
1:A:230:THR:O	1:A:234:ILE:HG13	2.14	0.47
1:D:232:GLY:O	1:D:236:ASN:ND2	2.47	0.47
1:F:162:GLY:HA3	1:F:214:ASP:CG	2.34	0.47
1:G:45:LEU:HD22	1:G:81:PRO:HB2	1.96	0.47
1:I:143:ASP:OD1	1:I:402:ARG:HB2	2.13	0.47
1:I:188:GLU:HA	1:I:191:ARG:HD2	1.96	0.47
1:B:57:ARG:NH2	1:B:329:GLU:HG2	2.29	0.47
1:E:200:MET:HB3	1:E:235:VAL:CG2	2.44	0.47
1:F:30:HIS:HD2	1:F:34:LEU:HG	1.79	0.47
1:G:56:THR:HG22	1:G:56:THR:O	2.14	0.47
3:A:502:QR8:C31	4:A:604:HOH:O	2.61	0.47
1:B:105:ARG:NH1	1:B:109:GLY:HA3	2.30	0.47
1:C:31:TYR:CZ	1:C:320:HIS:ND1	2.82	0.47
1:D:254:THR:CG2	1:D:400:LEU:HB2	2.45	0.47
1:G:356:CYS:SG	2:G:501:HEM:NA	2.87	0.47
1:I:106:ARG:NH2	1:I:110:LYS:HG2	2.28	0.47
1:D:106:ARG:NH1	1:D:110:LYS:HE3	2.30	0.47
1:F:152:PHE:HB3	1:F:153:PRO:HD3	1.97	0.47
1:I:149:ALA:CB	1:I:253:ILE:CG2	2.93	0.47
1:A:358:GLY:HA3	2:A:501:HEM:C3C	2.49	0.47
1:A:383:ALA:HB3	1:A:404:ILE:HG22	1.95	0.47
1:C:383:ALA:HB3	1:C:404:ILE:HG22	1.96	0.47
1:C:179:LEU:HD13	1:C:397:ILE:HD11	1.96	0.47
1:H:224:ASP:C	1:H:226:ASP:OD1	2.52	0.47
1:H:58:MET:CE	1:H:324:ALA:HB1	2.44	0.47
1:C:150:VAL:HB	1:C:151:PRO:HD3	1.96	0.47
1:A:230:THR:HG22	1:A:231:LYS:N	2.29	0.47
1:B:135:MET:HA	1:B:147:PHE:CD2	2.50	0.47
1:B:168:ARG:HD3	1:B:172:ARG:HD3	1.95	0.47
1:B:14:VAL:HG13	1:B:44:ARG:NH2	2.30	0.47
1:B:244:ALA:HB1	2:B:501:HEM:C4C	2.50	0.47
1:B:84:PHE:CD1	1:B:84:PHE:N	2.82	0.47
1:D:328:GLU:N	1:D:328:GLU:OE2	2.42	0.47
1:E:73:ALA:O	1:E:76:THR:OG1	2.28	0.47
1:F:123:ARG:HD3	1:F:159:GLU:OE1	2.15	0.47
1:F:39:PRO:HB3	1:F:57:ARG:HD2	1.96	0.47
1:I:194:GLN:O	1:I:198:VAL:HG23	2.15	0.47
1:B:75:ALA:HA	1:B:80:THR:HG21	1.96	0.47

Continued on next page...

CONFIDENTIAL VALIDATION REPORT

Continued from previous page...

Atom-1	Atom-2	Interatomic distance (Å)	Clash overlap (Å)
1:E:45:LEU:HD22	1:E:81:PRO:HB2	1.97	0.47
1:E:356:CYS:HA	2:E:501:HEM:C4D	2.49	0.47
1:A:99:PRO:C	1:A:102:THR:HG22	2.35	0.47
1:B:85:PRO:HB2	1:B:186:ALA:HB2	1.97	0.47
1:B:249:SER:O	1:B:253:ILE:HG13	2.15	0.47
1:C:167:ASP:HB3	1:C:199:TYR:OH	2.15	0.47
1:C:274:PRO:HD2	4:C:613:HOH:O	2.15	0.47
1:C:303:ASP:OD1	1:C:312:ARG:HA	2.15	0.47
1:C:81:PRO:O	1:C:297:VAL:HG21	2.15	0.47
1:E:123:ARG:O	1:E:127:LEU:HD13	2.15	0.47
1:F:107:LEU:O	1:F:110:LYS:HG2	2.15	0.47
1:F:252:GLN:HE21	1:F:285:LEU:HD23	1.79	0.47
1:I:133:ASP:CA	1:I:136:VAL:HG22	2.45	0.47
1:C:249:SER:O	1:C:253:ILE:HG13	2.16	0.46
1:F:305:GLU:HG2	1:F:310:THR:HB	1.97	0.46
1:H:203:LEU:HD21	1:H:207:ARG:NH2	2.30	0.46
1:I:219:LEU:CD1	1:I:234:ILE:O	2.63	0.46
1:B:149:ALA:O	1:B:250:VAL:CG2	2.60	0.46
1:B:274:PRO:O	1:B:277:VAL:HG23	2.15	0.46
1:D:329:GLU:OE2	1:D:329:GLU:HA	2.14	0.46
2:D:501:HEM:HBC2	2:D:501:HEM:CMC	2.44	0.46
1:E:152:PHE:HB3	1:E:153:PRO:HD3	1.97	0.46
1:E:120:MET:CE	1:E:159:GLU:HB3	2.44	0.46
1:E:212:THR:HG22	1:E:213:GLU:N	2.30	0.46
1:B:187:ALA:HB3	1:H:166:GLU:CG	2.45	0.46
1:A:286:ARG:HB2	1:A:346:HIS:HB3	1.97	0.46
1:A:382:LEU:HD22	1:A:384:GLU:O	2.15	0.46
1:A:35:ARG:HG3	1:A:56:THR:HG22	1.97	0.46
1:B:42:ARG:HG3	1:B:53:TRP:CE3	2.50	0.46
1:F:150:VAL:HB	1:F:151:PRO:HD3	1.98	0.46
1:F:30:HIS:ND1	4:F:601:HOH:O	2.36	0.46
1:A:355:HIS:O	1:A:356:CYS:C	2.52	0.46
1:A:67:ASP:OD2	1:A:69:ARG:NE	2.44	0.46
1:B:200:MET:HA	1:B:203:LEU:HD12	1.97	0.46
1:C:356:CYS:SG	2:C:501:HEM:C1C	3.00	0.46
1:H:107:LEU:HD23	1:H:229:LEU:CD2	2.45	0.46
1:B:42:ARG:HG2	1:B:53:TRP:CE3	2.50	0.46
1:D:163:VAL:HG21	1:D:171:PHE:CZ	2.50	0.46
1:G:156:VAL:HG21	1:G:365:GLU:HG2	1.98	0.46
1:H:200:MET:O	1:H:204:VAL:HG23	2.15	0.46
1:I:143:ASP:OD2	1:I:145:VAL:N	2.48	0.46

Continued on next page...

CONFIDENTIAL VALIDATION REPORT

Continued from previous page...

Atom-1	Atom-2	Interatomic distance (Å)	Clash overlap (Å)
1:E:74:ALA:HB1	1:E:299:VAL:HG21	1.95	0.46
1:H:127:LEU:O	1:H:131:LEU:CD1	2.63	0.46
1:H:38:GLU:HG3	1:H:38:GLU:O	2.15	0.46
1:C:220:ALA:O	1:C:223:THR:HG22	2.14	0.46
1:E:105:ARG:HA	1:E:105:ARG:HD2	1.58	0.46
1:E:286:ARG:HB2	1:E:346:HIS:HB3	1.98	0.46
1:E:55:VAL:HG13	1:E:60:ASP:HB3	1.97	0.46
1:F:377:PHE:N	1:F:377:PHE:CD1	2.84	0.46
1:F:376:ARG:CB	1:F:377:PHE:CE1	2.99	0.46
1:I:143:ASP:HB3	1:I:146:GLU:HB3	1.98	0.46
1:C:200:MET:HG3	1:C:239:VAL:HG12	1.96	0.46
1:C:67:ASP:OD1	1:C:68:SER:N	2.48	0.46
1:D:121:ARG:HB3	1:D:122:PRO:HD3	1.98	0.46
1:I:364:LEU:HD23	1:I:364:LEU:C	2.37	0.46
1:B:172:ARG:NH1	1:B:172:ARG:CG	2.78	0.46
1:B:46:PRO:HB2	1:B:47:TYR:CD1	2.51	0.46
2:B:501:HEM:HBA2	3:B:502:QR8:O12	2.16	0.46
1:C:207:ARG:HE	1:C:216:LEU:CB	2.28	0.46
1:C:57:ARG:NH1	1:C:329:GLU:OE2	2.49	0.46
1:C:359:ALA:O	1:C:363:ARG:HG3	2.16	0.46
2:C:501:HEM:HBB2	2:C:501:HEM:HMB2	1.98	0.46
1:F:254:THR:CG2	1:F:400:LEU:HB2	2.46	0.46
1:G:132:LEU:O	1:G:136:VAL:HG23	2.15	0.46
1:C:200:MET:CG	1:C:239:VAL:HG12	2.45	0.46
1:D:218:ALA:HA	1:D:221:LEU:HD23	1.98	0.46
1:B:272:ALA:HB1	1:D:330:VAL:CG2	2.46	0.46
1:E:356:CYS:SG	2:E:501:HEM:NA	2.89	0.46
1:I:104:LEU:HD12	1:I:105:ARG:HG2	1.98	0.46
1:F:376:ARG:C	1:F:377:PHE:CD1	2.90	0.45
1:H:96:GLN:HB3	1:H:101:HIS:HB2	1.98	0.45
1:H:156:VAL:HG21	1:H:365:GLU:HG2	1.97	0.45
1:I:40:VAL:HB	1:I:42:ARG:HH12	1.81	0.45
1:B:85:PRO:HG2	1:B:189:ILE:CD1	2.40	0.45
1:F:305:GLU:OE1	1:F:310:THR:HG21	2.16	0.45
1:G:208:ARG:HG3	1:G:231:LYS:HD3	1.99	0.45
1:E:156:VAL:HG21	1:E:365:GLU:HG2	1.98	0.45
1:H:221:LEU:N	1:H:221:LEU:CD2	2.79	0.45
1:B:168:ARG:HA	1:B:171:PHE:CD2	2.50	0.45
1:C:402:ARG:CD	1:C:404:ILE:HG13	2.46	0.45
2:E:501:HEM:HMC2	2:E:501:HEM:HBC2	1.97	0.45
1:I:189:ILE:O	1:I:192:VAL:HG23	2.15	0.45

Continued on next page...

CONFIDENTIAL VALIDATION REPORT

Continued from previous page...

Atom-1	Atom-2	Interatomic distance (Å)	Clash overlap (Å)
1:A:120:MET:CE	1:A:159:GLU:HB3	2.47	0.45
1:B:229:LEU:HD13	1:B:229:LEU:HA	1.76	0.45
1:E:212:THR:HG22	1:E:213:GLU:H	1.81	0.45
1:E:27:LEU:HA	1:E:27:LEU:HD23	1.81	0.45
1:E:75:ALA:HA	1:E:80:THR:HG21	1.98	0.45
1:F:107:LEU:HD23	1:F:229:LEU:HD23	1.98	0.45
1:F:31:TYR:HB3	1:F:323:SER:OG	2.16	0.45
1:F:81:PRO:O	1:F:297:VAL:HG21	2.17	0.45
1:H:286:ARG:HB2	1:H:346:HIS:HB3	1.98	0.45
1:E:35:ARG:HG3	1:E:56:THR:HG22	1.97	0.45
1:E:56:THR:HG22	1:E:56:THR:O	2.17	0.45
1:G:129:ASP:OD1	1:G:375:ARG:NH2	2.46	0.45
1:B:73:ALA:O	1:B:76:THR:OG1	2.27	0.45
1:A:307:SER:OG	1:A:308:THR:HG23	2.17	0.45
1:B:236:ASN:O	1:B:236:ASN:ND2	2.50	0.45
1:I:381:ASP:OD1	1:I:382:LEU:O	2.34	0.45
1:A:84:PHE:N	1:A:84:PHE:CD1	2.84	0.45
1:B:57:ARG:HH22	1:B:329:GLU:HG2	1.82	0.45
1:B:92:GLY:HA2	1:B:236:ASN:ND2	2.32	0.45
2:C:501:HEM:HBC2	2:C:501:HEM:HMC2	1.97	0.45
1:D:204:VAL:HG21	1:D:235:VAL:HG22	1.99	0.45
1:E:150:VAL:HB	1:E:151:PRO:HD3	1.99	0.45
1:E:260:LEU:HG	1:E:284:MET:CE	2.46	0.45
1:F:121:ARG:HB3	1:F:122:PRO:HD3	1.99	0.45
1:G:286:ARG:NH2	1:G:334:ALA:HA	2.32	0.45
1:B:150:VAL:HB	1:B:151:PRO:HD3	1.99	0.45
1:C:191:ARG:NH1	1:C:195:ASP:OD1	2.50	0.45
1:A:63:ILE:HG12	1:C:378:PRO:HB2	1.99	0.45
1:F:376:ARG:HB2	1:F:377:PHE:CE1	2.52	0.45
1:H:189:ILE:O	1:H:192:VAL:HG13	2.16	0.45
1:E:229:LEU:HD13	1:E:233:GLU:HB3	1.99	0.44
1:F:264:ARG:NE	1:F:381:ASP:OD1	2.48	0.44
1:H:27:LEU:HA	1:H:27:LEU:HD23	1.90	0.44
1:I:219:LEU:HD11	1:I:235:VAL:HA	1.98	0.44
1:E:377:PHE:O	1:E:380:LEU:HB2	2.18	0.44
2:E:501:HEM:HMB2	2:E:501:HEM:CBB	2.40	0.44
1:G:120:MET:CE	1:G:160:LEU:HD23	2.47	0.44
1:G:46:PRO:HB2	1:G:47:TYR:CD1	2.51	0.44
1:A:172:ARG:HG2	1:A:172:ARG:HH21	1.82	0.44
1:D:244:ALA:HA	3:D:502:QR8:C30	2.47	0.44
1:B:148:LEU:O	1:B:151:PRO:HD2	2.18	0.44

Continued on next page...

CONFIDENTIAL VALIDATION REPORT

Continued from previous page...

Atom-1	Atom-2	Interatomic distance (Å)	Clash overlap (Å)
1:B:157:ILE:HD11	1:B:246:HIS:HB3	2.00	0.44
1:B:101:HIS:CD2	1:B:354:HIS:CE1	3.05	0.44
1:D:286:ARG:HB2	1:D:346:HIS:HB3	2.00	0.44
1:E:346:HIS:HD2	1:E:348:ALA:H	1.65	0.44
1:F:191:ARG:HH11	1:F:191:ARG:HG3	1.81	0.44
3:G:502:QR8:C9	3:G:502:QR8:C32	2.95	0.44
1:A:208:ARG:NH2	1:H:100:ASP:OD1	2.50	0.44
1:H:183:ARG:CB	1:H:183:ARG:NH1	2.78	0.44
1:I:188:GLU:HA	1:I:191:ARG:CD	2.47	0.44
1:A:156:VAL:HG21	1:A:365:GLU:HG2	1.98	0.44
1:E:82:ARG:NH1	1:E:82:ARG:CG	2.81	0.44
1:F:191:ARG:NH1	1:F:191:ARG:CG	2.80	0.44
1:G:332:ASP:O	1:G:333:HIS:C	2.55	0.44
1:H:252:GLN:HE21	1:H:285:LEU:HD23	1.82	0.44
1:I:188:GLU:HA	1:I:191:ARG:CG	2.47	0.44
1:A:254:THR:CG2	1:A:400:LEU:HB2	2.46	0.44
1:B:231:LYS:C	1:B:231:LYS:CD	2.86	0.44
1:D:124:VAL:O	1:D:128:VAL:HG23	2.18	0.44
1:D:120:MET:CE	1:D:159:GLU:HB3	2.48	0.44
1:F:382:LEU:HD22	1:F:384:GLU:O	2.17	0.44
1:G:245:GLY:HA2	2:G:501:HEM:C1C	2.52	0.44
1:G:60:ASP:OD1	1:G:307:SER:HB3	2.17	0.44
1:I:153:PRO:CG	1:I:250:VAL:HG22	2.47	0.44
1:I:362:GLY:O	1:I:366:LEU:HG	2.18	0.44
1:B:83:MET:HB3	1:B:83:MET:HE2	1.62	0.44
1:D:184:LEU:HA	1:D:189:ILE:HD11	1.99	0.44
1:I:128:VAL:HG23	1:I:148:LEU:HD11	2.00	0.44
1:I:154:VAL:O	1:I:158:CYS:SG	2.76	0.44
1:A:332:ASP:O	1:A:333:HIS:C	2.55	0.44
1:C:332:ASP:O	1:C:333:HIS:C	2.56	0.44
1:D:384:GLU:OE2	1:D:402:ARG:NH2	2.51	0.44
1:D:81:PRO:O	1:D:297:VAL:HG21	2.18	0.44
1:E:363:ARG:O	1:E:367:GLN:HG3	2.17	0.44
1:F:30:HIS:CD2	1:F:34:LEU:HG	2.53	0.44
3:A:502:QR8:O12	3:A:502:QR8:O11	2.31	0.43
1:B:286:ARG:HB2	1:B:346:HIS:HB3	1.99	0.43
1:E:67:ASP:OD1	1:E:68:SER:N	2.51	0.43
1:G:157:ILE:CD1	1:G:242:LEU:HD12	2.43	0.43
1:I:364:LEU:O	1:I:368:GLU:HG2	2.18	0.43
1:A:38:GLU:OE1	4:A:601:HOH:O	2.21	0.43
1:A:76:THR:HG21	1:A:90:PRO:HA	2.00	0.43

Continued on next page...

CONFIDENTIAL VALIDATION REPORT

Continued from previous page...

Atom-1	Atom-2	Interatomic distance (Å)	Clash overlap (Å)
1:B:197:MET:CE	1:B:235:VAL:HG12	2.29	0.43
1:D:277:VAL:HB	1:D:278:PRO:HD3	2.00	0.43
1:H:163:VAL:HG21	1:H:171:PHE:CZ	2.53	0.43
1:C:402:ARG:HD3	1:C:404:ILE:CG1	2.48	0.43
1:E:195:ASP:O	1:E:198:VAL:HG12	2.18	0.43
1:H:151:PRO:CB	1:H:172:ARG:HH12	2.31	0.43
1:A:223:THR:CG2	1:A:226:ASP:HB2	2.36	0.43
1:C:171:PHE:O	1:C:175:SER:OG	2.36	0.43
1:G:178:MET:CE	1:G:193:GLN:HE21	2.31	0.43
1:I:260:LEU:HD13	1:I:284:MET:HE1	2.00	0.43
1:E:273:ASP:O	1:E:276:LEU:HB2	2.18	0.43
1:E:329:GLU:OE2	1:E:329:GLU:CA	2.65	0.43
1:F:359:ALA:O	1:F:363:ARG:HG3	2.18	0.43
1:G:154:VAL:HA	1:G:157:ILE:CD1	2.49	0.43
1:B:76:THR:HG21	1:B:90:PRO:HA	2.00	0.43
1:D:106:ARG:HH12	1:D:110:LYS:HE3	1.82	0.43
1:D:148:LEU:O	1:D:151:PRO:HD2	2.18	0.43
1:A:402:ARG:HG2	1:A:403:GLN:N	2.34	0.43
1:F:256:LEU:HD23	1:F:256:LEU:N	2.34	0.43
1:G:260:LEU:HG	1:G:284:MET:CE	2.49	0.43
1:H:190:GLN:HB3	1:H:190:GLN:HE21	1.56	0.43
1:H:76:THR:HG21	1:H:90:PRO:HA	2.01	0.43
1:E:118:GLU:O	1:E:118:GLU:HG3	2.19	0.43
1:E:204:VAL:HG11	1:E:234:ILE:CG2	2.45	0.43
1:E:331:PHE:CE2	1:E:345:PRO:HD2	2.54	0.43
1:F:129:ASP:OD1	1:F:375:ARG:NH2	2.46	0.43
1:F:171:PHE:O	1:F:175:SER:OG	2.35	0.43
1:F:58:MET:HE1	1:F:347:ILE:HG12	2.00	0.43
1:H:237:MET:HE3	1:H:237:MET:HA	2.01	0.43
1:I:149:ALA:CB	1:I:253:ILE:HG21	2.49	0.43
1:B:236:ASN:C	1:B:236:ASN:ND2	2.73	0.43
1:C:402:ARG:HD3	1:C:404:ILE:HG13	2.00	0.43
1:E:77:ASP:HB3	1:E:80:THR:OG1	2.18	0.43
1:A:163:VAL:HG21	1:A:171:PHE:CZ	2.54	0.43
1:A:237:MET:HA	1:A:237:MET:CE	2.48	0.43
1:C:113:THR:OG1	1:C:116:ARG:HG3	2.19	0.43
1:F:132:LEU:O	1:F:136:VAL:HG23	2.19	0.43
1:G:264:ARG:NH2	1:G:380:LEU:O	2.42	0.43
1:A:230:THR:HG22	1:A:232:GLY:N	2.29	0.42
1:B:45:LEU:HD22	1:B:81:PRO:HB2	2.01	0.42
1:C:27:LEU:HA	1:C:27:LEU:HD23	1.78	0.42

Continued on next page...

CONFIDENTIAL VALIDATION REPORT

Continued from previous page...

Atom-1	Atom-2	Interatomic distance (Å)	Clash overlap (Å)
1:D:57:ARG:HH22	1:D:329:GLU:HB2	1.84	0.42
1:H:383:ALA:HB3	1:H:404:ILE:HG22	2.01	0.42
1:B:14:VAL:CG1	1:B:44:ARG:NH2	2.82	0.42
1:B:170:LEU:CD1	1:B:174:PHE:CZ	3.03	0.42
1:C:161:LEU:HD12	1:C:215:LEU:HB2	2.01	0.42
1:D:27:LEU:HA	1:D:27:LEU:HD23	1.86	0.42
1:D:75:ALA:HA	1:D:80:THR:HG21	2.01	0.42
1:G:205:ALA:HA	1:G:231:LYS:NZ	2.34	0.42
1:H:303:ASP:OD1	1:H:312:ARG:HA	2.19	0.42
1:B:135:MET:HE3	1:B:144:LEU:HA	2.01	0.42
1:B:384:GLU:OE1	1:B:402:ARG:NH2	2.52	0.42
1:E:170:LEU:O	1:E:170:LEU:HD12	2.19	0.42
1:E:20:SER:OG	1:E:28:ASP:OD2	2.36	0.42
1:G:111:ALA:HB1	1:G:215:LEU:HD22	2.00	0.42
1:E:286:ARG:HB2	1:E:346:HIS:CB	2.49	0.42
1:F:287:TYR:CG	1:F:337:LEU:HD13	2.54	0.42
1:G:148:LEU:O	1:G:151:PRO:HD2	2.19	0.42
1:H:180:SER:O	1:H:181:SER:CB	2.66	0.42
1:H:94:LEU:HA	1:H:94:LEU:HD12	1.87	0.42
1:F:76:THR:HG21	1:F:90:PRO:HA	2.01	0.42
1:H:152:PHE:O	1:H:156:VAL:HG23	2.19	0.42
1:H:358:GLY:HA3	2:H:501:HEM:C3C	2.54	0.42
1:I:179:LEU:HD13	1:I:396:LEU:HD12	2.02	0.42
1:B:107:LEU:HD12	1:B:107:LEU:C	2.30	0.42
1:C:343:ARG:CZ	1:C:343:ARG:CB	2.96	0.42
1:F:58:MET:HE2	1:F:324:ALA:HB1	2.02	0.42
1:G:286:ARG:HH12	1:G:341:ARG:CZ	2.32	0.42
1:H:84:PHE:CD1	1:H:84:PHE:N	2.87	0.42
1:A:60:ASP:OD2	1:A:307:SER:HB3	2.20	0.42
1:C:252:GLN:OE1	1:C:290:LEU:HD13	2.20	0.42
1:D:359:ALA:O	1:D:363:ARG:HG3	2.19	0.42
1:D:356:CYS:SG	2:D:501:HEM:NA	2.92	0.42
1:H:171:PHE:O	1:H:175:SER:OG	2.38	0.42
1:H:55:VAL:HG13	1:H:60:ASP:HB3	2.01	0.42
1:A:356:CYS:HA	2:A:501:HEM:C4D	2.55	0.42
1:E:254:THR:CG2	1:E:400:LEU:HB2	2.49	0.42
1:E:73:ALA:HA	1:E:95:ALA:O	2.19	0.42
1:G:108:VAL:HG11	1:G:237:MET:CE	2.49	0.42
1:H:132:LEU:HG	1:H:377:PHE:HE2	1.85	0.42
1:I:384:GLU:OE2	1:I:404:ILE:HG12	2.20	0.42
2:I:501:HEM:HBB2	2:I:501:HEM:CMB	2.47	0.42

Continued on next page...

CONFIDENTIAL VALIDATION REPORT

Continued from previous page...

Atom-1	Atom-2	Interatomic distance (Å)	Clash overlap (Å)
1:A:227:ASP:N	1:A:227:ASP:OD1	2.42	0.42
1:A:360:GLN:OE1	1:A:363:ARG:NH1	2.53	0.42
1:B:208:ARG:NH2	1:G:355:HIS:CE1	2.88	0.42
1:H:121:ARG:HB3	1:H:122:PRO:HD3	2.00	0.42
1:H:226:ASP:O	1:H:227:ASP:C	2.57	0.42
1:I:384:GLU:CD	1:I:402:ARG:NH1	2.74	0.42
1:B:235:VAL:O	1:B:236:ASN:C	2.56	0.42
1:E:46:PRO:HB2	1:E:47:TYR:CD1	2.54	0.42
1:F:156:VAL:HG21	1:F:365:GLU:HG2	2.01	0.42
1:F:203:LEU:HD11	1:F:207:ARG:NH2	2.35	0.42
1:G:247:GLU:HG2	1:G:397:ILE:CD1	2.50	0.42
1:H:245:GLY:HA2	2:H:501:HEM:C1C	2.55	0.42
1:I:155:ALA:CA	1:I:158:CYS:SG	3.03	0.42
1:B:131:LEU:O	1:B:134:ASP:HB2	2.20	0.41
1:B:230:THR:HB	1:B:233:GLU:OE2	2.20	0.41
1:C:207:ARG:NE	1:C:216:LEU:HB3	2.34	0.41
1:C:273:ASP:O	1:C:276:LEU:HB2	2.19	0.41
1:D:349:PHE:C	2:D:501:HEM:HMA3	2.40	0.41
1:G:199:TYR:CD1	1:G:199:TYR:O	2.73	0.41
1:H:354:HIS:CD2	2:H:501:HEM:O1D	2.72	0.41
1:D:56:THR:HG22	1:D:56:THR:O	2.19	0.41
1:F:271:VAL:HA	1:F:374:VAL:HG13	2.00	0.41
1:F:376:ARG:HB3	1:F:377:PHE:CE1	2.55	0.41
1:G:81:PRO:O	1:G:297:VAL:HG21	2.21	0.41
1:B:150:VAL:HA	1:B:250:VAL:HG21	2.02	0.41
1:D:252:GLN:HE21	1:D:285:LEU:HD23	1.86	0.41
1:E:94:LEU:HA	1:E:94:LEU:HD12	1.92	0.41
1:F:84:PHE:CD1	1:F:84:PHE:N	2.89	0.41
1:I:113:THR:O	1:I:117:VAL:HG23	2.20	0.41
1:I:133:ASP:HA	1:I:136:VAL:CG2	2.48	0.41
1:I:219:LEU:CG	1:I:234:ILE:O	2.68	0.41
1:I:153:PRO:HB2	1:I:246:HIS:HA	2.02	0.41
1:A:107:LEU:HD12	1:A:229:LEU:HD23	2.01	0.41
1:A:42:ARG:NH1	1:A:42:ARG:CG	2.69	0.41
1:C:260:LEU:CD2	1:C:284:MET:HE3	2.51	0.41
1:C:290:LEU:O	1:C:397:ILE:HG22	2.20	0.41
1:E:39:PRO:HB3	1:E:57:ARG:HD2	2.02	0.41
1:G:27:LEU:HD23	1:G:27:LEU:HA	1.83	0.41
1:H:230:THR:HB	1:H:233:GLU:H	1.86	0.41
1:I:176:ASP:OD1	1:I:247:GLU:OE2	2.38	0.41
1:C:205:ALA:HA	1:C:208:ARG:HD2	2.02	0.41

Continued on next page...

CONFIDENTIAL VALIDATION REPORT

Continued from previous page...

Atom-1	Atom-2	Interatomic distance (Å)	Clash overlap (Å)
1:E:219:LEU:HB2	1:E:234:ILE:HD11	2.02	0.41
1:F:396:LEU:HG	3:F:502:QR8:C34	2.50	0.41
1:H:199:TYR:CE2	1:H:203:LEU:HD12	2.54	0.41
1:C:223:THR:HG22	1:C:224:ASP:OD1	2.21	0.41
1:D:227:ASP:HB3	1:F:227:ASP:HB2	2.03	0.41
1:D:94:LEU:HD21	3:D:502:QR8:C35	2.50	0.41
1:F:402:ARG:HG2	1:F:403:GLN:N	2.34	0.41
1:I:108:VAL:HG11	1:I:237:MET:SD	2.61	0.41
1:C:217:GLY:O	1:C:221:LEU:HD12	2.21	0.41
1:E:356:CYS:SG	2:E:501:HEM:C4C	3.13	0.41
1:H:356:CYS:HA	2:H:501:HEM:C4D	2.56	0.41
1:I:175:SER:O	1:I:179:LEU:HD23	2.20	0.41
1:A:227:ASP:HB3	1:H:227:ASP:HB2	2.02	0.41
1:B:31:TYR:CZ	1:B:320:HIS:CE1	3.09	0.41
1:B:54:LEU:C	1:B:54:LEU:HD23	2.36	0.41
1:C:150:VAL:HA	1:C:250:VAL:HG21	2.02	0.41
1:C:28:ASP:OD1	1:C:30:HIS:HB2	2.20	0.41
1:C:290:LEU:HG	1:C:397:ILE:HG22	2.03	0.41
1:D:156:VAL:HG21	1:D:365:GLU:HG2	2.02	0.41
1:G:247:GLU:HG2	1:G:397:ILE:HD11	2.02	0.41
1:H:94:LEU:HD12	1:H:354:HIS:HD2	1.79	0.41
1:A:176:ASP:O	1:A:182:THR:HB	2.21	0.41
1:A:204:VAL:HG21	1:A:235:VAL:HG22	2.03	0.41
1:A:260:LEU:HG	1:A:284:MET:HE1	2.02	0.41
1:B:133:ASP:OD1	1:B:376:ARG:NH2	2.50	0.41
1:F:278:PRO:O	1:F:281:VAL:HG22	2.20	0.41
1:G:249:SER:O	1:G:253:ILE:HG13	2.20	0.41
1:H:125:ARG:HG2	1:H:375:ARG:HH22	1.85	0.41
1:I:199:TYR:C	1:I:199:TYR:CD1	2.94	0.41
1:A:244:ALA:HB2	3:A:502:QR8:C31	2.51	0.41
3:A:502:QR8:C32	3:A:502:QR8:O11	2.69	0.41
1:B:292:SER:HB2	1:B:398:ARG:HG2	2.03	0.41
1:C:121:ARG:HB3	1:C:122:PRO:HD3	2.02	0.41
1:E:354:HIS:HA	2:E:501:HEM:O2D	2.21	0.41
1:G:331:PHE:CE2	1:G:345:PRO:HD2	2.55	0.41
1:H:377:PHE:O	1:H:380:LEU:HB2	2.21	0.41
1:H:55:VAL:HG13	1:H:60:ASP:CB	2.51	0.41
1:I:117:VAL:HG21	1:I:360:GLN:HB3	2.01	0.41
1:B:237:MET:HB3	1:B:237:MET:HE2	1.58	0.41
1:B:27:LEU:HD13	4:B:604:HOH:O	2.21	0.41
1:C:42:ARG:NH2	1:C:315:GLU:OE1	2.54	0.41

Continued on next page...

CONFIDENTIAL VALIDATION REPORT

Continued from previous page...

Atom-1	Atom-2	Interatomic distance (Å)	Clash overlap (Å)
1:D:94:LEU:HD12	1:D:94:LEU:HA	1.93	0.41
1:E:104:LEU:O	1:E:108:VAL:HG13	2.20	0.41
1:E:76:THR:HG21	1:E:90:PRO:HA	2.02	0.41
1:F:27:LEU:HA	1:F:27:LEU:HD23	1.93	0.41
1:G:215:LEU:HD13	1:G:215:LEU:C	2.41	0.41
1:H:189:ILE:HG23	1:H:190:GLN:N	2.36	0.41
1:H:218:ALA:HA	1:H:221:LEU:HD22	2.03	0.41
1:H:31:TYR:CE2	1:H:320:HIS:CD2	3.09	0.41
1:I:179:LEU:HD12	1:I:396:LEU:HD12	2.01	0.41
1:A:331:PHE:CE1	1:A:345:PRO:HD2	2.56	0.40
1:B:105:ARG:O	1:B:105:ARG:HD3	2.21	0.40
1:C:135:MET:HE3	1:C:144:LEU:HA	2.03	0.40
1:G:154:VAL:HA	1:G:157:ILE:HD11	2.03	0.40
1:B:327:ASP:O	1:B:330:VAL:HG12	2.21	0.40
1:C:73:ALA:HA	1:C:95:ALA:O	2.21	0.40
1:F:35:ARG:HG3	1:F:56:THR:HG22	2.03	0.40
1:F:58:MET:CE	1:F:324:ALA:HB1	2.51	0.40
1:A:121:ARG:HB3	1:A:122:PRO:HD3	2.02	0.40
1:A:132:LEU:O	1:A:136:VAL:HG23	2.22	0.40
1:A:107:LEU:HD12	1:A:229:LEU:CD2	2.51	0.40
1:C:250:VAL:CG1	1:C:251:ASN:N	2.83	0.40
1:D:105:ARG:NH1	2:D:501:HEM:O2D	2.54	0.40
1:D:76:THR:HG21	1:D:90:PRO:HA	2.02	0.40
1:F:199:TYR:CE1	1:F:203:LEU:HD22	2.56	0.40
1:G:359:ALA:O	1:G:363:ARG:HG3	2.22	0.40
1:H:127:LEU:HA	1:H:127:LEU:HD23	1.85	0.40
1:H:180:SER:O	1:H:181:SER:OG	2.24	0.40
1:H:235:VAL:O	1:H:239:VAL:HG23	2.21	0.40
1:I:372:ALA:HA	1:I:375:ARG:NH2	2.35	0.40
1:I:384:GLU:CD	1:I:402:ARG:HH12	2.22	0.40
1:I:241:LEU:CD2	2:I:501:HEM:HMD2	2.52	0.40
1:B:178:MET:CE	1:B:196:PHE:CD2	2.97	0.40
1:C:384:GLU:OE1	1:C:389:LEU:HD23	2.21	0.40
1:E:249:SER:O	1:E:253:ILE:HG13	2.21	0.40
1:E:263:GLU:O	1:E:266:ARG:CD	2.65	0.40
1:I:148:LEU:HA	1:I:148:LEU:HD12	1.78	0.40
1:A:172:ARG:HG2	1:A:172:ARG:NH2	2.37	0.40
1:C:174:PHE:HA	1:C:192:VAL:CG1	2.51	0.40
1:G:69:ARG:HD2	1:G:304:VAL:HG13	2.04	0.40

There are no symmetry-related clashes.

CONFIDENTIAL VALIDATION REPORT

5.3 Torsion angles ⓘ

5.3.1 Protein backbone ⓘ

In the following table, the Percentiles column shows the percent Ramachandran outliers of the chain as a percentile score with respect to all X-ray entries followed by that with respect to entries of similar resolution.

The Analysed column shows the number of residues for which the backbone conformation was analysed, and the total number of residues.

Mol	Chain	Analysed	Favoured	Allowed	Outliers	Percentiles	
1	A	392/407 (96%)	380 (97%)	12 (3%)	0	100	100
1	B	393/407 (97%)	374 (95%)	19 (5%)	0	100	100
1	C	393/407 (97%)	382 (97%)	11 (3%)	0	100	100
1	D	392/407 (96%)	381 (97%)	11 (3%)	0	100	100
1	E	382/407 (94%)	370 (97%)	12 (3%)	0	100	100
1	F	392/407 (96%)	374 (95%)	18 (5%)	0	100	100
1	G	375/407 (92%)	365 (97%)	10 (3%)	0	100	100
1	H	393/407 (97%)	379 (96%)	14 (4%)	0	100	100
1	I	230/407 (56%)	219 (95%)	11 (5%)	0	100	100
All	All	3342/3663 (91%)	3224 (96%)	118 (4%)	0	100	100

There are no Ramachandran outliers to report.

5.3.2 Protein sidechains ⓘ

In the following table, the Percentiles column shows the percent sidechain outliers of the chain as a percentile score with respect to all X-ray entries followed by that with respect to entries of similar resolution.

The Analysed column shows the number of residues for which the sidechain conformation was analysed, and the total number of residues.

Mol	Chain	Analysed	Rotameric	Outliers	Percentiles	
1	A	331/341 (97%)	281 (85%)	50 (15%)	3	8
1	B	331/341 (97%)	255 (77%)	76 (23%)	1	2
1	C	331/341 (97%)	279 (84%)	52 (16%)	2	7
1	D	330/341 (97%)	276 (84%)	54 (16%)	2	6
1	E	323/341 (95%)	268 (83%)	55 (17%)	2	5

Continued on next page...

CONFIDENTIAL VALIDATION REPORT

Continued from previous page...

Mol	Chain	Analysed	Rotameric	Outliers	Percentiles	
1	F	331/341 (97%)	278 (84%)	53 (16%)	2	7
1	G	319/341 (94%)	268 (84%)	51 (16%)	2	7
1	H	331/341 (97%)	281 (85%)	50 (15%)	3	8
1	I	205/341 (60%)	165 (80%)	40 (20%)	1	3
All	All	2832/3069 (92%)	2351 (83%)	481 (17%)	2	5

All (481) residues with a non-rotameric sidechain are listed below:

Mol	Chain	Res	Type
1	A	30	HIS
1	A	41	SER
1	A	42	ARG
1	A	43	VAL
1	A	54	LEU
1	A	69	ARG
1	A	71	SER
1	A	76	THR
1	A	96	GLN
1	A	105	ARG
1	A	112	PHE
1	A	123	ARG
1	A	125	ARG
1	A	129	ASP
1	A	154	VAL
1	A	159	GLU
1	A	172	ARG
1	A	173	THR
1	A	175	SER
1	A	178	MET
1	A	179	LEU
1	A	184	LEU
1	A	190	GLN
1	A	198	VAL
1	A	221	LEU
1	A	223	THR
1	A	227	ASP
1	A	237	MET
1	A	247	GLU
1	A	256	LEU
1	A	263	GLU
1	A	269	SER

Continued on next page...

CONFIDENTIAL VALIDATION REPORT

Continued from previous page...

Mol	Chain	Res	Type
1	A	276	LEU
1	A	286	ARG
1	A	292	SER
1	A	301	THR
1	A	307	SER
1	A	309	VAL
1	A	310	THR
1	A	318	VAL
1	A	329	GLU
1	A	337	LEU
1	A	342	GLU
1	A	357	ILE
1	A	364	LEU
1	A	380	LEU
1	A	386	VAL
1	A	390	LYS
1	A	393	GLN
1	A	396	LEU
1	B	14	VAL
1	B	21	LEU
1	B	27	LEU
1	B	30	HIS
1	B	37	ASP
1	B	42	ARG
1	B	49	GLU
1	B	51	THR
1	B	53	TRP
1	B	54	LEU
1	B	71	SER
1	B	76	THR
1	B	84	PHE
1	B	89	GLU
1	B	100	ASP
1	B	104	LEU
1	B	105	ARG
1	B	106	ARG
1	B	108	VAL
1	B	116	ARG
1	B	123	ARG
1	B	125	ARG
1	B	127	LEU
1	B	130	SER

Continued on next page...

CONFIDENTIAL VALIDATION REPORT

Continued from previous page...

Mol	Chain	Res	Type
1	B	157	ILE
1	B	161	LEU
1	B	166	GLU
1	B	168	ARG
1	B	171	PHE
1	B	173	THR
1	B	175	SER
1	B	178	MET
1	B	182	THR
1	B	183	ARG
1	B	184	LEU
1	B	185	THR
1	B	188	GLU
1	B	190	GLN
1	B	196	PHE
1	B	201	ASP
1	B	215	LEU
1	B	216	LEU
1	B	221	LEU
1	B	226	ASP
1	B	227	ASP
1	B	228	HIS
1	B	229	LEU
1	B	231	LYS
1	B	233	GLU
1	B	234	ILE
1	B	235	VAL
1	B	236	ASN
1	B	237	MET
1	B	242	LEU
1	B	250	VAL
1	B	256	LEU
1	B	263	GLU
1	B	265	LYS
1	B	269	SER
1	B	276	LEU
1	B	286	ARG
1	B	288	THR
1	B	292	SER
1	B	305	GLU
1	B	307	SER
1	B	310	THR

Continued on next page...

CONFIDENTIAL VALIDATION REPORT

Continued from previous page...

Mol	Chain	Res	Type
1	B	315	GLU
1	B	318	VAL
1	B	327	ASP
1	B	328	GLU
1	B	329	GLU
1	B	337	LEU
1	B	357	ILE
1	B	364	LEU
1	B	382	LEU
1	B	390	LYS
1	C	14	VAL
1	C	41	SER
1	C	42	ARG
1	C	51	THR
1	C	54	LEU
1	C	76	THR
1	C	89	GLU
1	C	104	LEU
1	C	125	ARG
1	C	127	LEU
1	C	130	SER
1	C	132	LEU
1	C	161	LEU
1	C	168	ARG
1	C	171	PHE
1	C	173	THR
1	C	175	SER
1	C	178	MET
1	C	185	THR
1	C	191	ARG
1	C	196	PHE
1	C	200	MET
1	C	201	ASP
1	C	207	ARG
1	C	209	ASP
1	C	216	LEU
1	C	221	LEU
1	C	223	THR
1	C	224	ASP
1	C	227	ASP
1	C	228	HIS
1	C	229	LEU

Continued on next page...

CONFIDENTIAL VALIDATION REPORT

Continued from previous page...

Mol	Chain	Res	Type
1	C	230	THR
1	C	250	VAL
1	C	263	GLU
1	C	269	SER
1	C	276	LEU
1	C	288	THR
1	C	292	SER
1	C	305	GLU
1	C	310	THR
1	C	315	GLU
1	C	318	VAL
1	C	330	VAL
1	C	337	LEU
1	C	343	ARG
1	C	357	ILE
1	C	364	LEU
1	C	382	LEU
1	C	390	LYS
1	C	395	MET
1	C	397	ILE
1	D	41	SER
1	D	42	ARG
1	D	43	VAL
1	D	54	LEU
1	D	71	SER
1	D	76	THR
1	D	84	PHE
1	D	91	ASP
1	D	96	GLN
1	D	105	ARG
1	D	106	ARG
1	D	112	PHE
1	D	115	ARG
1	D	123	ARG
1	D	125	ARG
1	D	129	ASP
1	D	130	SER
1	D	136	VAL
1	D	154	VAL
1	D	159	GLU
1	D	170	LEU
1	D	172	ARG

Continued on next page...

CONFIDENTIAL VALIDATION REPORT

Continued from previous page...

Mol	Chain	Res	Type
1	D	173	THR
1	D	175	SER
1	D	178	MET
1	D	179	LEU
1	D	181	SER
1	D	184	LEU
1	D	190	GLN
1	D	203	LEU
1	D	208	ARG
1	D	209	ASP
1	D	213	GLU
1	D	223	THR
1	D	224	ASP
1	D	230	THR
1	D	256	LEU
1	D	264	ARG
1	D	265	LYS
1	D	269	SER
1	D	276	LEU
1	D	286	ARG
1	D	307	SER
1	D	309	VAL
1	D	310	THR
1	D	318	VAL
1	D	337	LEU
1	D	357	ILE
1	D	364	LEU
1	D	380	LEU
1	D	382	LEU
1	D	386	VAL
1	D	390	LYS
1	D	396	LEU
1	E	30	HIS
1	E	36	ARG
1	E	41	SER
1	E	43	VAL
1	E	49	GLU
1	E	54	LEU
1	E	71	SER
1	E	76	THR
1	E	82	ARG
1	E	84	PHE

Continued on next page...

CONFIDENTIAL VALIDATION REPORT

Continued from previous page...

Mol	Chain	Res	Type
1	E	96	GLN
1	E	107	LEU
1	E	112	PHE
1	E	115	ARG
1	E	123	ARG
1	E	125	ARG
1	E	129	ASP
1	E	154	VAL
1	E	159	GLU
1	E	172	ARG
1	E	175	SER
1	E	191	ARG
1	E	193	GLN
1	E	208	ARG
1	E	209	ASP
1	E	213	GLU
1	E	221	LEU
1	E	226	ASP
1	E	228	HIS
1	E	229	LEU
1	E	230	THR
1	E	235	VAL
1	E	256	LEU
1	E	263	GLU
1	E	265	LYS
1	E	266	ARG
1	E	269	SER
1	E	276	LEU
1	E	286	ARG
1	E	299	VAL
1	E	301	THR
1	E	305	GLU
1	E	309	VAL
1	E	310	THR
1	E	318	VAL
1	E	329	GLU
1	E	337	LEU
1	E	375	ARG
1	E	376	ARG
1	E	380	LEU
1	E	382	LEU
1	E	386	VAL

Continued on next page...

CONFIDENTIAL VALIDATION REPORT

Continued from previous page...

Mol	Chain	Res	Type
1	E	390	LYS
1	E	393	GLN
1	E	396	LEU
1	F	41	SER
1	F	42	ARG
1	F	43	VAL
1	F	49	GLU
1	F	51	THR
1	F	54	LEU
1	F	71	SER
1	F	76	THR
1	F	84	PHE
1	F	91	ASP
1	F	105	ARG
1	F	106	ARG
1	F	107	LEU
1	F	112	PHE
1	F	123	ARG
1	F	125	ARG
1	F	128	VAL
1	F	129	ASP
1	F	130	SER
1	F	146	GLU
1	F	154	VAL
1	F	166	GLU
1	F	173	THR
1	F	175	SER
1	F	178	MET
1	F	179	LEU
1	F	182	THR
1	F	183	ARG
1	F	184	LEU
1	F	189	ILE
1	F	191	ARG
1	F	208	ARG
1	F	214	ASP
1	F	221	LEU
1	F	224	ASP
1	F	237	MET
1	F	263	GLU
1	F	269	SER
1	F	276	LEU

Continued on next page...

CONFIDENTIAL VALIDATION REPORT

Continued from previous page...

Mol	Chain	Res	Type
1	F	309	VAL
1	F	310	THR
1	F	318	VAL
1	F	323	SER
1	F	357	ILE
1	F	377	PHE
1	F	379	THR
1	F	380	LEU
1	F	381	ASP
1	F	382	LEU
1	F	386	VAL
1	F	390	LYS
1	F	393	GLN
1	F	396	LEU
1	G	30	HIS
1	G	41	SER
1	G	43	VAL
1	G	54	LEU
1	G	71	SER
1	G	86	THR
1	G	100	ASP
1	G	105	ARG
1	G	106	ARG
1	G	107	LEU
1	G	112	PHE
1	G	125	ARG
1	G	129	ASP
1	G	130	SER
1	G	154	VAL
1	G	159	GLU
1	G	160	LEU
1	G	172	ARG
1	G	173	THR
1	G	175	SER
1	G	178	MET
1	G	179	LEU
1	G	188	GLU
1	G	191	ARG
1	G	192	VAL
1	G	207	ARG
1	G	208	ARG
1	G	212	THR

Continued on next page...

CONFIDENTIAL VALIDATION REPORT

Continued from previous page...

Mol	Chain	Res	Type
1	G	228	HIS
1	G	229	LEU
1	G	233	GLU
1	G	247	GLU
1	G	263	GLU
1	G	269	SER
1	G	276	LEU
1	G	286	ARG
1	G	301	THR
1	G	304	VAL
1	G	309	VAL
1	G	310	THR
1	G	312	ARG
1	G	318	VAL
1	G	343	ARG
1	G	357	ILE
1	G	364	LEU
1	G	380	LEU
1	G	382	LEU
1	G	386	VAL
1	G	390	LYS
1	G	393	GLN
1	G	397	ILE
1	H	14	VAL
1	H	41	SER
1	H	49	GLU
1	H	51	THR
1	H	54	LEU
1	H	71	SER
1	H	76	THR
1	H	91	ASP
1	H	96	GLN
1	H	107	LEU
1	H	112	PHE
1	H	123	ARG
1	H	125	ARG
1	H	128	VAL
1	H	130	SER
1	H	154	VAL
1	H	173	THR
1	H	175	SER
1	H	178	MET

Continued on next page...

CONFIDENTIAL VALIDATION REPORT

Continued from previous page...

Mol	Chain	Res	Type
1	H	179	LEU
1	H	188	GLU
1	H	190	GLN
1	H	192	VAL
1	H	198	VAL
1	H	201	ASP
1	H	226	ASP
1	H	227	ASP
1	H	230	THR
1	H	231	LYS
1	H	237	MET
1	H	256	LEU
1	H	263	GLU
1	H	269	SER
1	H	276	LEU
1	H	286	ARG
1	H	292	SER
1	H	305	GLU
1	H	310	THR
1	H	318	VAL
1	H	323	SER
1	H	337	LEU
1	H	347	ILE
1	H	357	ILE
1	H	364	LEU
1	H	380	LEU
1	H	382	LEU
1	H	386	VAL
1	H	390	LYS
1	H	393	GLN
1	H	396	LEU
1	I	42	ARG
1	I	57	ARG
1	I	60	ASP
1	I	62	ARG
1	I	63	ILE
1	I	64	VAL
1	I	100	ASP
1	I	105	ARG
1	I	106	ARG
1	I	125	ARG
1	I	130	SER

Continued on next page...

CONFIDENTIAL VALIDATION REPORT

Continued from previous page...

Mol	Chain	Res	Type
1	I	132	LEU
1	I	135	MET
1	I	140	SER
1	I	167	ASP
1	I	168	ARG
1	I	169	ASP
1	I	171	PHE
1	I	172	ARG
1	I	180	SER
1	I	191	ARG
1	I	192	VAL
1	I	196	PHE
1	I	199	TYR
1	I	200	MET
1	I	230	THR
1	I	233	GLU
1	I	236	ASN
1	I	237	MET
1	I	241	LEU
1	I	246	HIS
1	I	259	LEU
1	I	284	MET
1	I	288	THR
1	I	291	VAL
1	I	354	HIS
1	I	363	ARG
1	I	373	LEU
1	I	390	LYS
1	I	405	VAL

Some sidechains can be flipped to improve hydrogen bonding and reduce clashes. All (30) such sidechains are listed below:

Mol	Chain	Res	Type
1	A	194	GLN
1	A	228	HIS
1	B	193	GLN
1	B	236	ASN
1	C	194	GLN
1	C	236	ASN
1	D	228	HIS
1	D	236	ASN
1	D	354	HIS

Continued on next page...

CONFIDENTIAL VALIDATION REPORT

Continued from previous page...

Mol	Chain	Res	Type
1	E	194	GLN
1	E	346	HIS
1	E	354	HIS
1	F	96	GLN
1	F	194	GLN
1	F	225	ASN
1	F	228	HIS
1	F	236	ASN
1	F	354	HIS
1	F	355	HIS
1	G	190	GLN
1	G	193	GLN
1	G	194	GLN
1	G	354	HIS
1	H	96	GLN
1	H	190	GLN
1	H	194	GLN
1	H	236	ASN
1	H	344	ASN
1	H	354	HIS
1	I	138	HIS

5.3.3 RNA (i)

There are no RNA molecules in this entry.

5.4 Non-standard residues in protein, DNA, RNA chains (i)

There are no non-standard protein/DNA/RNA residues in this entry.

5.5 Carbohydrates (i)

There are no monosaccharides in this entry.

5.6 Ligand geometry (i)

18 ligands are modelled in this entry.

In the following table, the Counts columns list the number of bonds (or angles) for which Mogul statistics could be retrieved, the number of bonds (or angles) that are observed in the model and the number of bonds (or angles) that are defined in the Chemical Component Dictionary. The

CONFIDENTIAL VALIDATION REPORT

Link column lists molecule types, if any, to which the group is linked. The Z score for a bond length (or angle) is the number of standard deviations the observed value is removed from the expected value. A bond length (or angle) with $|Z| > 2$ is considered an outlier worth inspection. RMSZ is the root-mean-square of all Z scores of the bond lengths (or angles).

Mol	Type	Chain	Res	Link	Bond lengths			Bond angles		
					Counts	RMSZ	$\#\|Z\| > 2$	Counts	RMSZ	$\#\|Z\| > 2$
2	HEM	I	501	1	27,50,50	0.98	2 (7%)	17,82,82	1.58	2 (11%)
2	HEM	C	501	1	27,50,50	1.05	2 (7%)	17,82,82	2.39	5 (29%)
2	HEM	A	501	1,4	27,50,50	1.36	4 (14%)	17,82,82	1.20	1 (5%)
3	QR8	B	502	-	26,26,26	2.01	7 (26%)	35,38,38	1.82	6 (17%)
2	HEM	G	501	-	27,50,50	0.98	1 (3%)	17,82,82	1.31	3 (17%)
3	QR8	D	502	-	26,26,26	1.70	5 (19%)	35,38,38	2.45	11 (31%)
2	HEM	E	501	-	27,50,50	1.47	3 (11%)	17,82,82	1.19	1 (5%)
3	QR8	F	502	-	26,26,26	1.59	5 (19%)	35,38,38	1.94	12 (34%)
3	QR8	G	502	-	26,26,26	1.59	5 (19%)	35,38,38	1.77	9 (25%)
2	HEM	D	501	4	27,50,50	1.11	1 (3%)	17,82,82	1.36	3 (17%)
3	QR8	I	502	1	26,26,26	1.32	3 (11%)	35,38,38	2.12	9 (25%)
2	HEM	H	501	4	27,50,50	1.11	2 (7%)	17,82,82	1.22	1 (5%)
3	QR8	A	502	-	26,26,26	1.79	7 (26%)	35,38,38	2.22	12 (34%)
2	HEM	B	501	1	27,50,50	1.26	4 (14%)	17,82,82	1.72	5 (29%)
3	QR8	C	502	-	26,26,26	1.71	6 (23%)	35,38,38	2.23	9 (25%)
3	QR8	E	502	-	26,26,26	1.51	2 (7%)	35,38,38	2.52	15 (42%)
2	HEM	F	501	4	27,50,50	1.03	2 (7%)	17,82,82	1.38	2 (11%)
3	QR8	H	502	-	26,26,26	1.60	4 (15%)	35,38,38	2.40	12 (34%)

In the following table, the Chirals column lists the number of chiral outliers, the number of chiral centers analysed, the number of these observed in the model and the number defined in the Chemical Component Dictionary. Similar counts are reported in the Torsion and Rings columns. '-' means no outliers of that kind were identified.

Mol	Type	Chain	Res	Link	Chirals	Torsions	Rings
2	HEM	I	501	1	-	2/6/54/54	-
2	HEM	C	501	1	-	2/6/54/54	-
2	HEM	A	501	1,4	-	0/6/54/54	-
3	QR8	B	502	-	-	24/48/48/48	0/1/1/1
2	HEM	G	501	-	-	0/6/54/54	-
3	QR8	D	502	-	-	25/48/48/48	0/1/1/1
2	HEM	E	501	-	-	0/6/54/54	-
3	QR8	F	502	-	-	27/48/48/48	0/1/1/1

Continued on next page...

CONFIDENTIAL VALIDATION REPORT

Continued from previous page...

Mol	Type	Chain	Res	Link	Chirals	Torsions	Rings
3	QR8	G	502	-	-	19/48/48/48	0/1/1/1
2	HEM	D	501	4	-	0/6/54/54	-
3	QR8	I	502	1	-	16/48/48/48	0/1/1/1
2	HEM	H	501	4	-	0/6/54/54	-
3	QR8	A	502	-	-	18/48/48/48	0/1/1/1
2	HEM	B	501	1	-	0/6/54/54	-
3	QR8	C	502	-	-	23/48/48/48	0/1/1/1
3	QR8	E	502	-	2/2/12/12	29/48/48/48	1/1/1/1
2	HEM	F	501	4	-	0/6/54/54	-
3	QR8	H	502	-	-	20/48/48/48	0/1/1/1

All (65) bond length outliers are listed below:

Mol	Chain	Res	Type	Atoms	Z	Observed(Å)	Ideal(Å)
2	E	501	HEM	C3B-C2B	-5.36	1.32	1.40
3	E	502	QR8	O2-C1	-5.26	1.22	1.34
3	B	502	QR8	O2-C13	-4.84	1.38	1.46
3	G	502	QR8	O2-C1	4.68	1.45	1.34
3	A	502	QR8	C10-C9	-4.57	1.45	1.52
3	D	502	QR8	O2-C13	-4.52	1.39	1.46
3	I	502	QR8	O2-C1	4.45	1.44	1.34
3	B	502	QR8	C10-C9	-4.33	1.46	1.52
3	A	502	QR8	O2-C1	4.16	1.44	1.34
3	H	502	QR8	O2-C1	4.14	1.44	1.34
3	C	502	QR8	O2-C13	-3.91	1.40	1.46
3	F	502	QR8	C10-C9	-3.86	1.46	1.52
2	H	501	HEM	C3B-C2B	-3.82	1.35	1.40
3	F	502	QR8	O2-C1	3.81	1.43	1.34
2	D	501	HEM	C4D-C3D	3.74	1.51	1.42
2	A	501	HEM	C4D-C3D	3.59	1.50	1.42
3	E	502	QR8	C10-C9	-3.55	1.47	1.52
3	D	502	QR8	C10-C9	-3.50	1.47	1.52
3	B	502	QR8	O2-C1	3.42	1.42	1.34
3	C	502	QR8	O2-C1	3.35	1.42	1.34
2	B	501	HEM	C4D-C3D	3.31	1.50	1.42
3	H	502	QR8	O2-C13	-3.20	1.41	1.46
3	F	502	QR8	O2-C13	-3.02	1.41	1.46
2	F	501	HEM	C3B-C2B	-2.92	1.36	1.40
2	C	501	HEM	C4D-C3D	2.92	1.49	1.42
2	I	501	HEM	CAA-C2A	2.81	1.56	1.52
3	G	502	QR8	O2-C13	-2.81	1.42	1.46

Continued on next page...

CONFIDENTIAL VALIDATION REPORT

Continued from previous page...

Mol	Chain	Res	Type	Atoms	Z	Observed(Å)	Ideal(Å)
2	F	501	HEM	C4D-C3D	2.80	1.48	1.42
3	C	502	QR8	C7-C6	-2.79	1.49	1.54
3	H	502	QR8	C2-C1	-2.73	1.45	1.51
3	B	502	QR8	O11-C9	-2.66	1.17	1.21
3	G	502	QR8	C7-C6	-2.66	1.49	1.54
3	A	502	QR8	O2-C13	-2.66	1.42	1.46
3	D	502	QR8	O2-C1	2.65	1.40	1.34
3	H	502	QR8	C10-C9	-2.64	1.48	1.52
2	H	501	HEM	C4D-C3D	2.58	1.48	1.42
3	A	502	QR8	O11-C9	-2.57	1.17	1.21
2	G	501	HEM	C1D-ND	-2.57	1.30	1.36
2	B	501	HEM	C1C-C2C	-2.56	1.36	1.42
3	I	502	QR8	O2-C13	-2.54	1.42	1.46
2	A	501	HEM	C3C-CAC	-2.53	1.42	1.47
3	A	502	QR8	C10-C11	-2.42	1.49	1.53
3	D	502	QR8	C7-C6	-2.42	1.50	1.54
2	E	501	HEM	C3D-C2D	-2.39	1.30	1.37
3	C	502	QR8	O11-C9	-2.30	1.17	1.21
3	G	502	QR8	C10-C9	-2.29	1.49	1.52
3	C	502	QR8	C10-C9	-2.29	1.49	1.52
3	F	502	QR8	C2-C1	-2.26	1.46	1.51
2	I	501	HEM	C4D-C3D	2.23	1.47	1.42
3	B	502	QR8	C2-C1	-2.23	1.46	1.51
3	F	502	QR8	C10-C11	-2.21	1.49	1.53
2	C	501	HEM	C4B-NB	-2.19	1.31	1.36
2	A	501	HEM	CMD-C2D	-2.14	1.47	1.51
2	A	501	HEM	C3C-C2C	-2.10	1.37	1.40
3	D	502	QR8	O11-C9	-2.10	1.18	1.21
3	I	502	QR8	C2-C1	-2.08	1.47	1.51
3	B	502	QR8	C2-C3	-2.07	1.49	1.53
3	A	502	QR8	C2-C1	-2.06	1.47	1.51
3	A	502	QR8	C7-C6	-2.06	1.50	1.54
3	B	502	QR8	C7-C6	-2.06	1.50	1.54
2	E	501	HEM	C4D-C3D	2.03	1.47	1.42
3	G	502	QR8	C10-C11	-2.03	1.49	1.53
3	C	502	QR8	C12-C13	-2.02	1.48	1.53
2	B	501	HEM	C4A-NA	-2.02	1.32	1.36
2	B	501	HEM	CAA-C2A	2.01	1.55	1.52

All (118) bond angle outliers are listed below:

Continued on next page...

CONFIDENTIAL VALIDATION REPORT

Continued from previous page...

Mol	Chain	Res	Type	Atoms	Z	Observed(^{o})	Ideal(^{o})
3	D	502	QR8	C13-O2-C1	-8.04	105.86	117.51
3	H	502	QR8	C35-C12-C13	-6.25	104.10	112.18
2	C	501	HEM	CAA-CBA-CGA	-6.16	102.33	112.67
3	H	502	QR8	C6-C5-C4	-5.97	107.02	116.27
3	E	502	QR8	O2-C1-C2	5.70	124.08	111.56
3	I	502	QR8	C13-O2-C1	-5.37	109.73	117.51
3	C	502	QR8	C36-C13-C12	-5.37	106.62	114.39
3	E	502	QR8	C34-C10-C9	-5.32	98.82	108.08
3	A	502	QR8	C13-O2-C1	-5.20	109.98	117.51
3	A	502	QR8	O2-C1-C2	5.16	122.89	111.56
3	C	502	QR8	O2-C1-C2	5.11	122.77	111.56
3	D	502	QR8	C6-C5-C4	-5.01	108.51	116.27
3	B	502	QR8	C36-C13-C12	-4.87	107.34	114.39
3	G	502	QR8	C6-C5-C4	-4.69	109.00	116.27
3	C	502	QR8	O2-C1-O1	-4.64	115.28	123.94
3	I	502	QR8	O2-C1-C2	4.60	121.67	111.56
3	C	502	QR8	C6-C5-C4	-4.56	109.21	116.27
3	H	502	QR8	C30-C2-C3	4.53	119.92	112.37
3	I	502	QR8	C3-C2-C1	-4.52	100.33	110.02
3	I	502	QR8	C36-C13-C12	-4.42	107.98	114.39
3	B	502	QR8	C3-C2-C1	-4.42	100.55	110.02
3	F	502	QR8	O2-C1-C2	4.31	121.02	111.56
3	D	502	QR8	O2-C1-O1	-4.28	115.95	123.94
3	D	502	QR8	O2-C1-C2	4.26	120.92	111.56
3	H	502	QR8	C3-C4-C5	-4.20	104.10	112.54
3	C	502	QR8	O12-C11-C12	-4.09	100.38	109.49
3	E	502	QR8	C6-C5-C4	-4.06	109.98	116.27
3	B	502	QR8	C6-C5-C4	-3.95	110.15	116.27
2	C	501	HEM	CBA-CAA-C2A	3.95	119.76	112.49
3	D	502	QR8	C11-C10-C9	-3.88	103.33	110.36
3	A	502	QR8	C11-C10-C9	-3.87	103.36	110.36
3	E	502	QR8	C36-C13-C12	-3.82	108.85	114.39
2	I	501	HEM	CBA-CAA-C2A	3.75	119.40	112.49
3	G	502	QR8	C32-C6-C7	-3.74	105.09	110.69
3	G	502	QR8	O2-C1-C2	3.61	119.47	111.56
3	F	502	QR8	C11-C10-C9	-3.56	103.90	110.36
3	I	502	QR8	C6-C5-C4	-3.56	110.76	116.27
2	C	501	HEM	CAD-CBD-CGD	3.54	118.61	112.67
3	E	502	QR8	O2-C13-C12	3.52	115.40	106.30
3	C	502	QR8	C35-C12-C13	-3.50	107.65	112.18
3	G	502	QR8	C13-O2-C1	-3.50	112.44	117.51

Continued on next page...

CONFIDENTIAL VALIDATION REPORT

Continued from previous page...

Mol	Chain	Res	Type	Atoms	Z	Observed(°)	Ideal(°)
3	A	502	QR8	C33-C8-C9	-3.45	101.15	109.44
3	F	502	QR8	C30-C2-C1	-3.43	101.26	109.02
3	D	502	QR8	C36-C13-C12	-3.43	109.42	114.39
2	C	501	HEM	CMC-C2C-C3C	3.42	131.07	124.68
2	F	501	HEM	C4A-C3A-C2A	3.36	109.34	107.00
2	I	501	HEM	C4A-C3A-C2A	3.34	109.32	107.00
3	E	502	QR8	C35-C12-C13	-3.31	107.89	112.18
3	E	502	QR8	C30-C2-C3	-3.31	106.85	112.37
3	E	502	QR8	C31-C4-C5	-3.30	104.71	111.39
3	H	502	QR8	O2-C1-C2	3.26	118.72	111.56
3	C	502	QR8	C32-C6-C7	-3.24	105.83	110.69
3	A	502	QR8	C35-C12-C13	-3.24	107.99	112.18
3	F	502	QR8	C36-C13-C12	-3.22	109.73	114.39
3	E	502	QR8	C35-C12-C11	-3.22	104.89	111.39
3	E	502	QR8	O11-C9-C10	-3.21	116.04	120.60
2	B	501	HEM	C1D-C2D-C3D	3.20	109.22	107.00
2	B	501	HEM	CBA-CAA-C2A	3.17	118.33	112.49
3	E	502	QR8	O3-C3-C2	-3.15	101.48	108.82
3	F	502	QR8	C34-C10-C11	-3.14	107.13	112.37
3	F	502	QR8	C35-C12-C11	-3.12	105.09	111.39
3	B	502	QR8	O2-C1-C2	3.11	118.39	111.56
3	F	502	QR8	C10-C11-C12	-3.05	108.13	114.41
3	A	502	QR8	O11-C9-C10	-3.01	116.33	120.60
3	B	502	QR8	C34-C10-C9	-3.00	102.87	108.08
3	E	502	QR8	O1-C1-C2	-2.99	116.09	124.08
3	H	502	QR8	C3-C2-C1	-2.96	103.66	110.02
3	A	502	QR8	O1-C1-C2	-2.96	116.16	124.08
3	H	502	QR8	O11-C9-C10	-2.96	116.39	120.60
3	D	502	QR8	C32-C6-C7	-2.94	106.29	110.69
3	A	502	QR8	C34-C10-C9	-2.90	103.03	108.08
3	H	502	QR8	C8-C9-C10	2.88	124.11	119.10
3	A	502	QR8	C36-C13-C12	-2.82	110.31	114.39
2	H	501	HEM	CMC-C2C-C3C	2.81	129.93	124.68
3	I	502	QR8	O2-C13-C12	2.79	113.51	106.30
3	H	502	QR8	C13-O2-C1	-2.76	113.52	117.51
3	A	502	QR8	C32-C6-C7	-2.71	106.62	110.69
2	B	501	HEM	CMC-C2C-C3C	2.68	129.70	124.68
3	C	502	QR8	C3-C2-C1	2.64	115.69	110.02
3	H	502	QR8	C30-C2-C1	-2.64	103.06	109.02
3	I	502	QR8	C35-C12-C13	-2.61	108.80	112.18
3	D	502	QR8	C2-C3-C4	-2.61	109.03	114.41
2	F	501	HEM	CBD-CAD-C3D	-2.61	107.67	112.48

Continued on next page...

CONFIDENTIAL VALIDATION REPORT

Continued from previous page...

Mol	Chain	Res	Type	Atoms	Z	Observed(°)	Ideal(°)
2	D	501	HEM	CMB-C2B-C3B	2.60	129.54	124.68
3	G	502	QR8	C33-C8-C7	-2.54	106.21	111.55
3	E	502	QR8	C3-C4-C5	2.45	117.46	112.54
2	G	501	HEM	CMC-C2C-C3C	2.42	129.21	124.68
3	I	502	QR8	O1-C1-C2	-2.42	117.62	124.08
3	B	502	QR8	O2-C1-O1	-2.40	119.46	123.94
3	D	502	QR8	C33-C8-C7	-2.37	106.58	111.55
2	G	501	HEM	C4A-C3A-C2A	2.36	108.64	107.00
2	D	501	HEM	CMC-C2C-C3C	2.33	129.04	124.68
2	E	501	HEM	C4A-C3A-C2A	2.32	108.61	107.00
3	G	502	QR8	C30-C2-C1	-2.28	103.87	109.02
3	H	502	QR8	C34-C10-C9	-2.26	104.14	108.08
3	C	502	QR8	C2-C3-C4	-2.26	109.75	114.41
3	F	502	QR8	C13-O2-C1	-2.25	114.24	117.51
2	G	501	HEM	CBA-CAA-C2A	2.21	116.56	112.49
3	A	502	QR8	C8-C9-C10	2.20	122.93	119.10
3	E	502	QR8	O2-C1-O1	-2.20	119.83	123.94
3	F	502	QR8	O2-C13-C36	2.18	112.55	108.21
2	C	501	HEM	CMD-C2D-C1D	-2.18	125.12	128.46
3	F	502	QR8	C32-C6-C7	-2.17	107.44	110.69
3	I	502	QR8	C30-C2-C3	-2.15	108.78	112.37
3	E	502	QR8	C32-C6-C5	-2.15	107.56	111.54
3	F	502	QR8	O2-C1-O1	-2.14	119.94	123.94
2	B	501	HEM	C3C-C4C-NC	-2.14	106.91	110.94
3	G	502	QR8	C32-C6-C5	2.13	115.48	111.54
3	F	502	QR8	C8-C9-C10	-2.10	115.45	119.10
3	G	502	QR8	O2-C1-O1	-2.08	120.05	123.94
2	B	501	HEM	C4C-C3C-C2C	2.07	108.35	106.90
2	D	501	HEM	CBD-CAD-C3D	-2.07	108.66	112.48
3	D	502	QR8	O11-C9-C8	-2.06	117.44	121.26
3	G	502	QR8	C35-C12-C11	-2.04	107.26	111.39
3	D	502	QR8	O3-C3-C2	2.04	113.55	108.82
3	H	502	QR8	C36-C13-C12	-2.03	111.45	114.39
3	A	502	QR8	C30-C2-C1	-2.02	104.45	109.02
2	A	501	HEM	CMC-C2C-C3C	2.00	128.43	124.68

All (2) chirality outliers are listed below:

Mol	Chain	Res	Type	Atom
3	E	502	QR8	C8
3	E	502	QR8	C11

CONFIDENTIAL VALIDATION REPORT

All (205) torsion outliers are listed below:

Mol	Chain	Res	Type	Atoms
2	I	501	HEM	C1A-C2A-CAA-CBA
2	I	501	HEM	C3A-C2A-CAA-CBA
2	C	501	HEM	C1A-C2A-CAA-CBA
2	C	501	HEM	C3A-C2A-CAA-CBA
3	B	502	QR8	C9-C10-C11-O12
3	B	502	QR8	C10-C11-C12-C35
3	B	502	QR8	C10-C11-C12-C13
3	B	502	QR8	O12-C11-C12-C35
3	B	502	QR8	O12-C11-C12-C13
3	B	502	QR8	C12-C13-O2-C1
3	B	502	QR8	C1-C2-C3-C4
3	D	502	QR8	C9-C10-C11-O12
3	D	502	QR8	C9-C10-C11-C12
3	D	502	QR8	C34-C10-C11-C12
3	D	502	QR8	C11-C12-C13-C36
3	D	502	QR8	C11-C12-C13-O2
3	D	502	QR8	C35-C12-C13-C36
3	D	502	QR8	C35-C12-C13-O2
3	D	502	QR8	C2-C1-O2-C13
3	D	502	QR8	O1-C1-O2-C13
3	D	502	QR8	C4-C5-C6-C7
3	D	502	QR8	O7-C5-C6-C32
3	D	502	QR8	C31-C4-C5-C6
3	D	502	QR8	C3-C4-C5-C6
3	D	502	QR8	C31-C4-C5-O7
3	D	502	QR8	C3-C4-C5-O7
3	F	502	QR8	C4-C5-C6-C7
3	F	502	QR8	C4-C5-C6-C32
3	F	502	QR8	C3-C4-C5-O7
3	F	502	QR8	O3-C3-C4-C5
3	F	502	QR8	C1-C2-C3-C4
3	I	502	QR8	O12-C11-C12-C35
3	I	502	QR8	C32-C6-C7-C8
3	I	502	QR8	C4-C5-C6-C7
3	I	502	QR8	O7-C5-C6-C32
3	I	502	QR8	C4-C5-C6-C32
3	I	502	QR8	C3-C4-C5-C6
3	A	502	QR8	C10-C11-C12-C35
3	A	502	QR8	C10-C11-C12-C13
3	A	502	QR8	O12-C11-C12-C13
3	A	502	QR8	C11-C12-C13-O2
3	A	502	QR8	C35-C12-C13-O2

Continued on next page...

CONFIDENTIAL VALIDATION REPORT

Continued from previous page...

Mol	Chain	Res	Type	Atoms
3	A	502	QR8	C3-C4-C5-C6
3	A	502	QR8	C3-C4-C5-O7
3	A	502	QR8	O3-C3-C4-C5
3	A	502	QR8	C2-C3-C4-C5
3	A	502	QR8	O3-C3-C4-C31
3	A	502	QR8	C2-C3-C4-C31
3	C	502	QR8	C9-C10-C11-O12
3	C	502	QR8	O12-C11-C12-C13
3	C	502	QR8	C5-C6-C7-C8
3	C	502	QR8	O7-C5-C6-C7
3	C	502	QR8	O7-C5-C6-C32
3	C	502	QR8	C4-C5-C6-C32
3	E	502	QR8	C33-C8-C9-O11
3	E	502	QR8	C33-C8-C9-C10
3	E	502	QR8	C9-C10-C11-O12
3	E	502	QR8	C9-C10-C11-C12
3	E	502	QR8	C34-C10-C11-O12
3	E	502	QR8	C10-C11-C12-C13
3	E	502	QR8	C12-C13-O2-C1
3	E	502	QR8	C36-C13-O2-C1
3	E	502	QR8	C6-C7-C8-C9
3	E	502	QR8	C6-C7-C8-C33
3	E	502	QR8	O7-C5-C6-C7
3	E	502	QR8	O7-C5-C6-C32
3	E	502	QR8	C3-C4-C5-C6
3	H	502	QR8	C10-C11-C12-C35
3	H	502	QR8	C10-C11-C12-C13
3	H	502	QR8	O12-C11-C12-C35
3	H	502	QR8	O12-C11-C12-C13
3	H	502	QR8	C6-C7-C8-C33
3	H	502	QR8	C5-C6-C7-C8
3	H	502	QR8	C31-C4-C5-C6
3	H	502	QR8	C3-C4-C5-C6
3	H	502	QR8	C31-C4-C5-O7
3	H	502	QR8	C3-C4-C5-O7
3	I	502	QR8	C2-C1-O2-C13
3	E	502	QR8	C2-C1-O2-C13
3	E	502	QR8	O1-C1-O2-C13
3	F	502	QR8	C31-C4-C5-O7
3	A	502	QR8	O12-C11-C12-C35
3	C	502	QR8	O12-C11-C12-C35
3	I	502	QR8	O12-C11-C12-C13

Continued on next page...

CONFIDENTIAL VALIDATION REPORT

Continued from previous page...

Mol	Chain	Res	Type	Atoms
3	I	502	QR8	C3-C4-C5-O7
3	E	502	QR8	O12-C11-C12-C13
3	E	502	QR8	O3-C3-C4-C5
3	I	502	QR8	C10-C11-C12-C35
3	C	502	QR8	C10-C11-C12-C35
3	C	502	QR8	C10-C11-C12-C13
3	A	502	QR8	C2-C1-O2-C13
3	I	502	QR8	O1-C1-O2-C13
3	C	502	QR8	C36-C13-O2-C1
3	F	502	QR8	O3-C3-C4-C31
3	G	502	QR8	O3-C3-C4-C31
3	G	502	QR8	O3-C3-C4-C5
3	C	502	QR8	C3-C4-C5-O7
3	F	502	QR8	C10-C11-C12-C35
3	B	502	QR8	C30-C2-C3-O3
3	D	502	QR8	C34-C10-C11-O12
3	C	502	QR8	C30-C2-C3-O3
3	B	502	QR8	C34-C10-C11-C12
3	D	502	QR8	C30-C2-C3-C4
3	C	502	QR8	C34-C10-C11-C12
3	C	502	QR8	C30-C2-C3-C4
3	E	502	QR8	C34-C10-C11-C12
3	E	502	QR8	C3-C4-C5-O7
3	F	502	QR8	C31-C4-C5-C6
3	F	502	QR8	C10-C11-C12-C13
3	F	502	QR8	C2-C3-C4-C5
3	G	502	QR8	C2-C3-C4-C5
3	F	502	QR8	O7-C5-C6-C32
3	F	502	QR8	O12-C11-C12-C35
3	C	502	QR8	C31-C4-C5-O7
3	E	502	QR8	C31-C4-C5-O7
3	F	502	QR8	C2-C3-C4-C31
3	G	502	QR8	C2-C3-C4-C31
3	F	502	QR8	C3-C4-C5-C6
3	I	502	QR8	C10-C11-C12-C13
3	A	502	QR8	O1-C1-O2-C13
3	D	502	QR8	O7-C5-C6-C7
3	F	502	QR8	O7-C5-C6-C7
3	B	502	QR8	C9-C10-C11-C12
3	C	502	QR8	C9-C10-C11-C12
3	E	502	QR8	C4-C5-C6-C32
3	F	502	QR8	O12-C11-C12-C13

Continued on next page...

CONFIDENTIAL VALIDATION REPORT

Continued from previous page...

Mol	Chain	Res	Type	Atoms
3	A	502	QR8	C31-C4-C5-C6
3	C	502	QR8	C34-C10-C11-O12
3	E	502	QR8	C2-C3-C4-C5
3	C	502	QR8	C32-C6-C7-C8
3	B	502	QR8	O3-C3-C4-C31
3	B	502	QR8	C30-C2-C3-C4
3	G	502	QR8	C30-C2-C3-C4
3	B	502	QR8	O3-C3-C4-C5
3	G	502	QR8	C3-C4-C5-O7
3	B	502	QR8	C4-C5-C6-C7
3	C	502	QR8	C4-C5-C6-C7
3	E	502	QR8	C4-C5-C6-C7
3	H	502	QR8	C4-C5-C6-C7
3	H	502	QR8	C6-C7-C8-C9
3	F	502	QR8	C11-C10-C9-O11
3	E	502	QR8	C31-C4-C5-C6
3	A	502	QR8	C31-C4-C5-O7
3	B	502	QR8	C34-C10-C11-O12
3	G	502	QR8	C30-C2-C3-O3
3	F	502	QR8	C11-C10-C9-C8
3	E	502	QR8	O3-C3-C4-C31
3	I	502	QR8	O7-C5-C6-C7
3	I	502	QR8	C31-C4-C5-C6
3	C	502	QR8	C31-C4-C5-C6
3	E	502	QR8	C10-C11-C12-C35
3	E	502	QR8	C2-C3-C4-C31
3	B	502	QR8	C2-C3-C4-C5
3	I	502	QR8	C31-C4-C5-O7
3	F	502	QR8	C34-C10-C9-C8
3	C	502	QR8	C3-C4-C5-C6
3	D	502	QR8	C32-C6-C7-C8
3	F	502	QR8	C32-C6-C7-C8
3	G	502	QR8	C32-C6-C7-C8
3	H	502	QR8	C32-C6-C7-C8
3	G	502	QR8	C31-C4-C5-O7
3	B	502	QR8	C2-C3-C4-C31
3	F	502	QR8	O2-C1-C2-C3
3	G	502	QR8	O2-C1-C2-C3
3	F	502	QR8	O1-C1-C2-C3
3	H	502	QR8	C2-C3-C4-C31
3	B	502	QR8	C1-C2-C3-O3
3	G	502	QR8	C1-C2-C3-O3

Continued on next page...

CONFIDENTIAL VALIDATION REPORT

Continued from previous page...

Mol	Chain	Res	Type	Atoms
3	A	502	QR8	C6-C7-C8-C9
3	C	502	QR8	C1-C2-C3-C4
3	C	502	QR8	C1-C2-C3-O3
3	G	502	QR8	O7-C5-C6-C7
3	H	502	QR8	O7-C5-C6-C7
3	E	502	QR8	O12-C11-C12-C35
3	G	502	QR8	C11-C10-C9-O11
3	G	502	QR8	C9-C10-C11-O12
3	G	502	QR8	O1-C1-C2-C3
3	B	502	QR8	C3-C4-C5-O7
3	H	502	QR8	C30-C2-C3-C4
3	D	502	QR8	C5-C6-C7-C8
3	F	502	QR8	C5-C6-C7-C8
3	G	502	QR8	C3-C4-C5-C6
3	F	502	QR8	C34-C10-C9-O11
3	G	502	QR8	C31-C4-C5-C6
3	D	502	QR8	C7-C8-C9-C10
3	F	502	QR8	C7-C8-C9-C10
3	G	502	QR8	C7-C8-C9-C10
3	H	502	QR8	C7-C8-C9-C10
3	E	502	QR8	C11-C12-C13-C36
3	B	502	QR8	O7-C5-C6-C7
3	B	502	QR8	C33-C8-C9-O11
3	A	502	QR8	C35-C12-C13-C36
3	H	502	QR8	O7-C5-C6-C32
3	I	502	QR8	O3-C3-C4-C31
3	H	502	QR8	C4-C5-C6-C32
3	B	502	QR8	O7-C5-C6-C32
3	B	502	QR8	O1-C1-C2-C3
3	B	502	QR8	C33-C8-C9-C10
3	D	502	QR8	C33-C8-C9-C10
3	D	502	QR8	C30-C2-C3-O3
3	F	502	QR8	C33-C8-C9-C10
3	D	502	QR8	C11-C10-C9-C8
3	G	502	QR8	C11-C10-C9-C8
3	D	502	QR8	C34-C10-C9-C8
3	H	502	QR8	C7-C8-C9-O11

All (1) ring outliers are listed below:

Mol	Chain	Res	Type	Atoms
3	E	502	QR8	C1-C10-C11-C12-C13-C2-C3-C4-C5-C6-C7-C8-C9-O2

CONFIDENTIAL VALIDATION REPORT

18 monomers are involved in 116 short contacts:

Mol	Chain	Res	Type	Clashes	Symm-Clashes
2	I	501	HEM	6	0
2	C	501	HEM	3	0
2	A	501	HEM	12	0
3	B	502	QR8	2	0
2	G	501	HEM	9	0
3	D	502	QR8	4	0
2	E	501	HEM	15	0
3	F	502	QR8	4	0
3	G	502	QR8	4	0
2	D	501	HEM	9	0
3	I	502	QR8	5	0
2	H	501	HEM	13	0
3	A	502	QR8	10	0
2	B	501	HEM	5	0
3	C	502	QR8	1	0
3	E	502	QR8	5	0
2	F	501	HEM	11	0
3	H	502	QR8	4	0

The following is a two-dimensional graphical depiction of Mogul quality analysis of bond lengths, bond angles, torsion angles, and ring geometry for all instances of the Ligand of Interest. In addition, ligands with molecular weight > 250 and outliers as shown on the validation Tables will also be included. For torsion angles, if less then 5% of the Mogul distribution of torsion angles is within 10 degrees of the torsion angle in question, then that torsion angle is considered an outlier. Any bond that is central to one or more torsion angles identified as an outlier by Mogul will be highlighted in the graph. For rings, the root-mean-square deviation (RMSD) between the ring in question and similar rings identified by Mogul is calculated over all ring torsion angles. If the average RMSD is greater than 60 degrees and the minimal RMSD between the ring in question and any Mogul-identified rings is also greater than 60 degrees, then that ring is considered an outlier. The outliers are highlighted in purple. The color gray indicates Mogul did not find sufficient equivalents in the CSD to analyse the geometry.

CONFIDENTIAL VALIDATION REPORT

Ligand HEM I 501

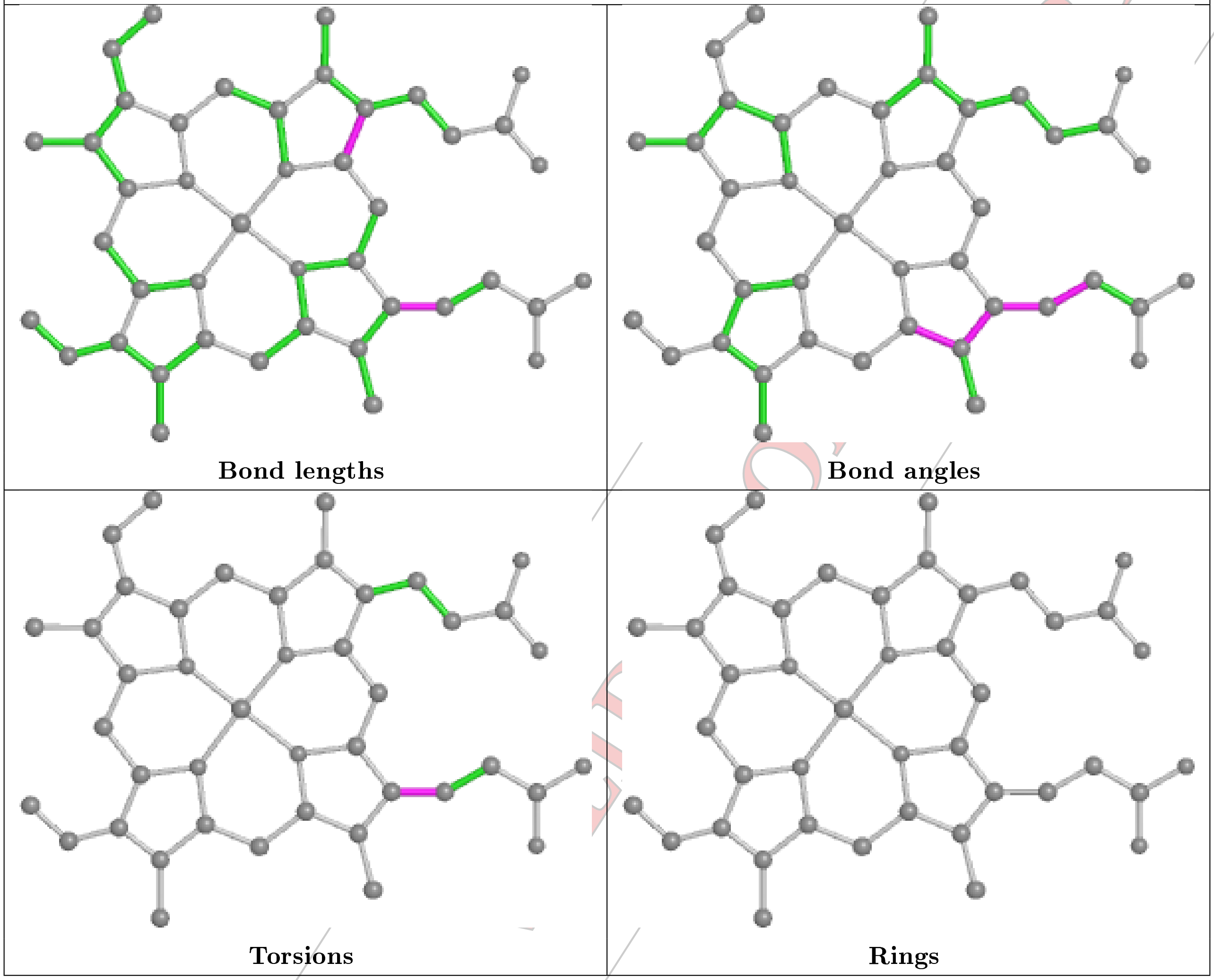

CONFIDENTIAL

Ligand HEM C 501

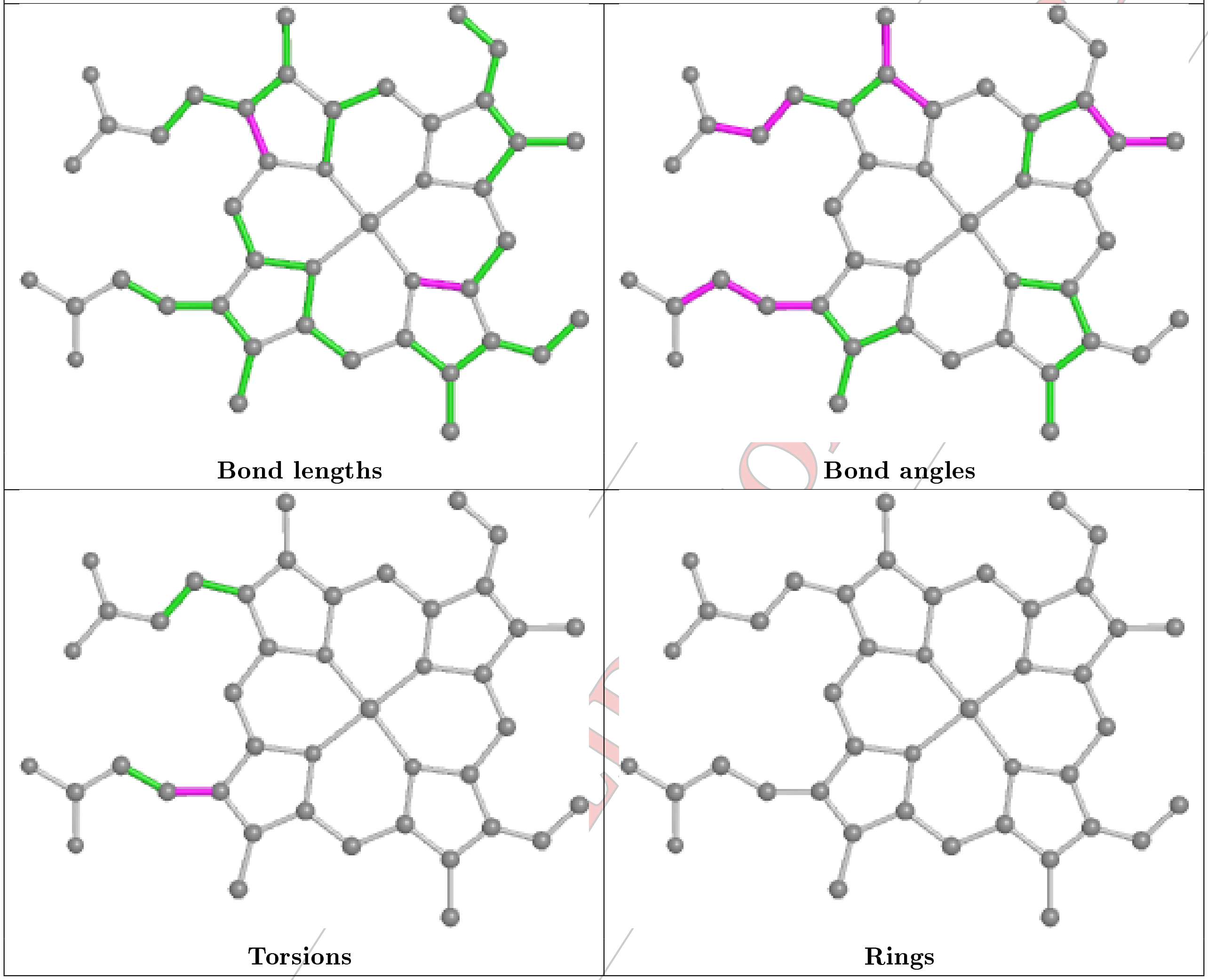

CONFIDENTIAL

Ligand HEM A 501

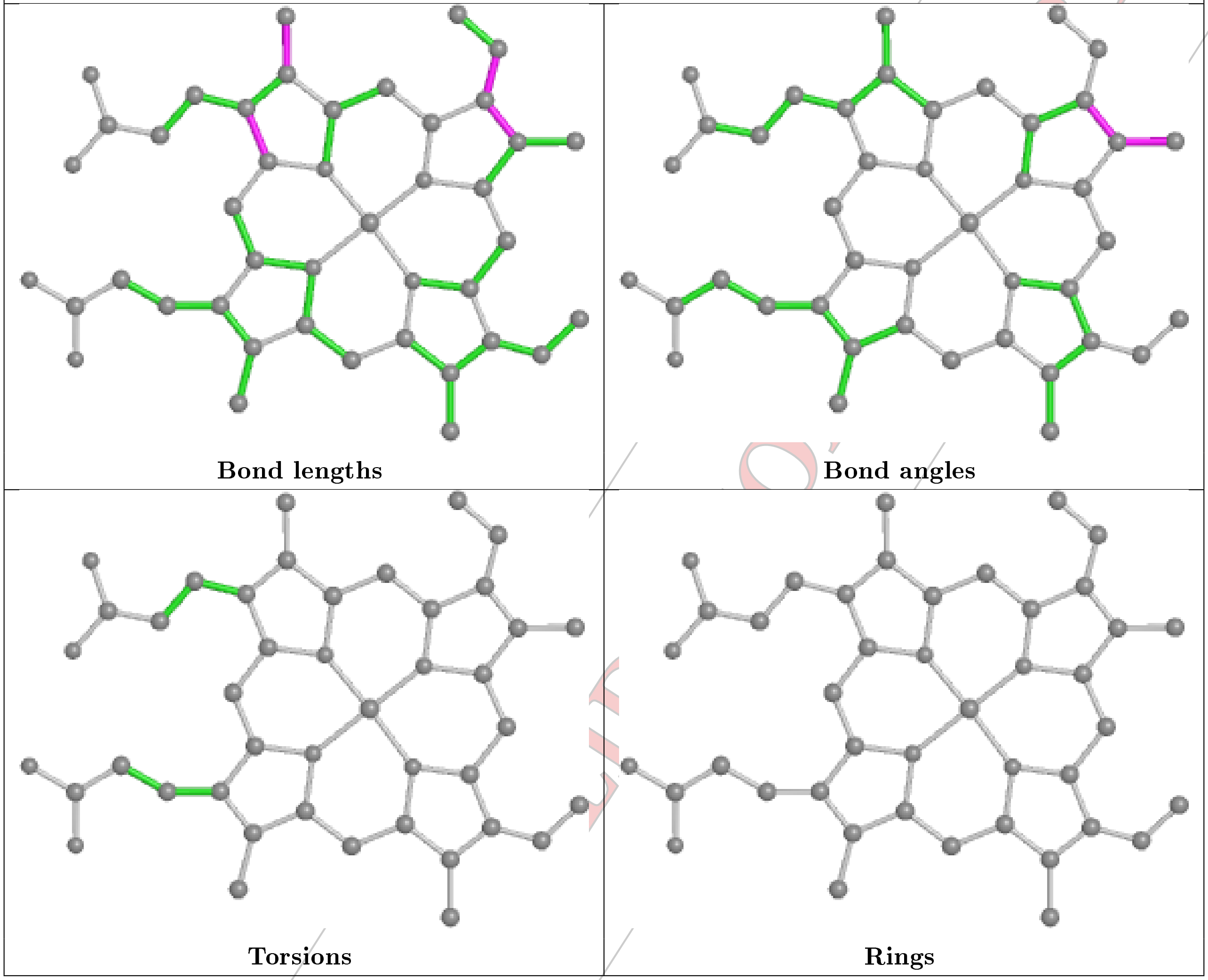

CONFIDENTIAL

Ligand QR8 B 502

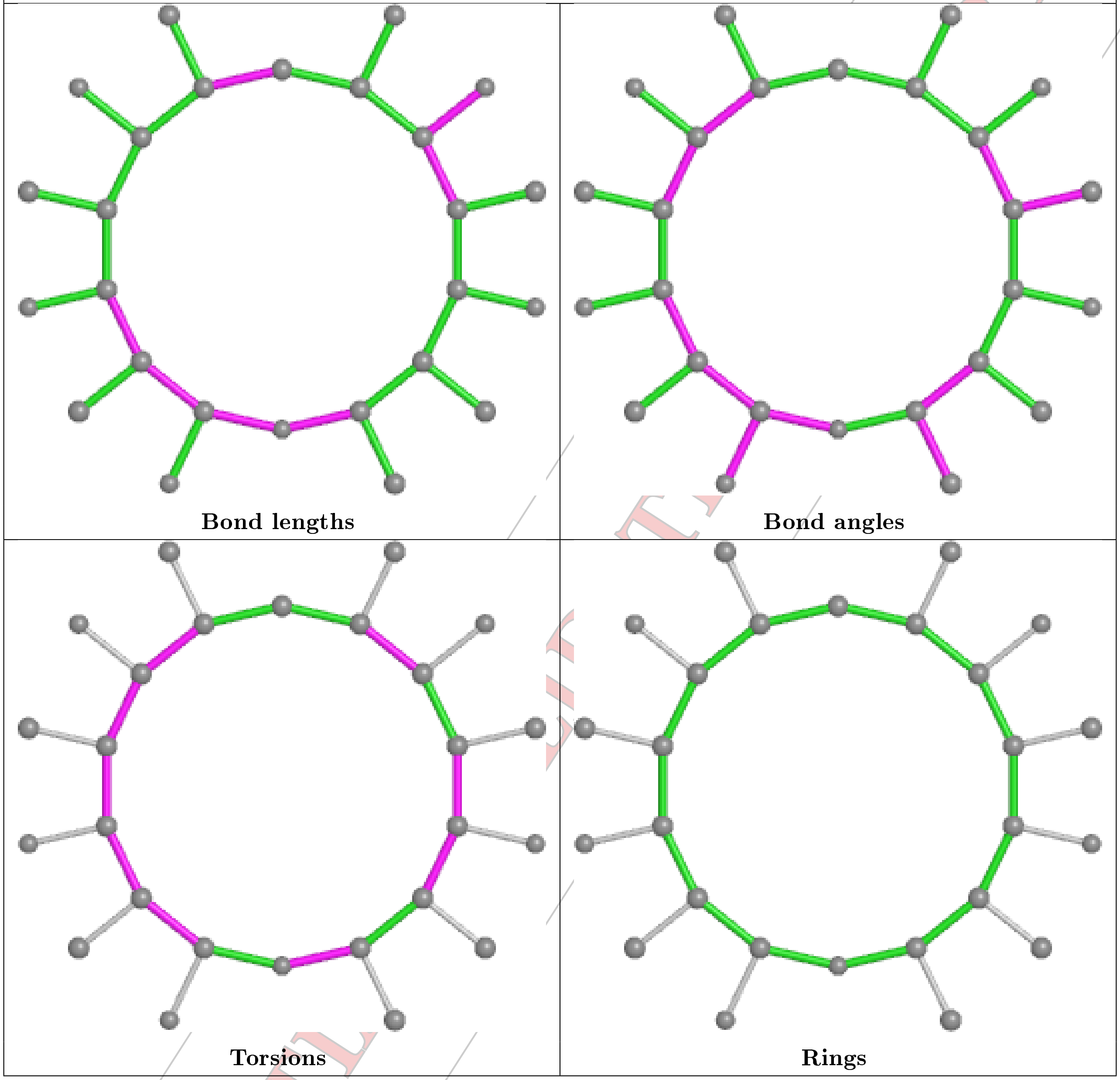

CONFIDENTIAL

Ligand HEM G 501

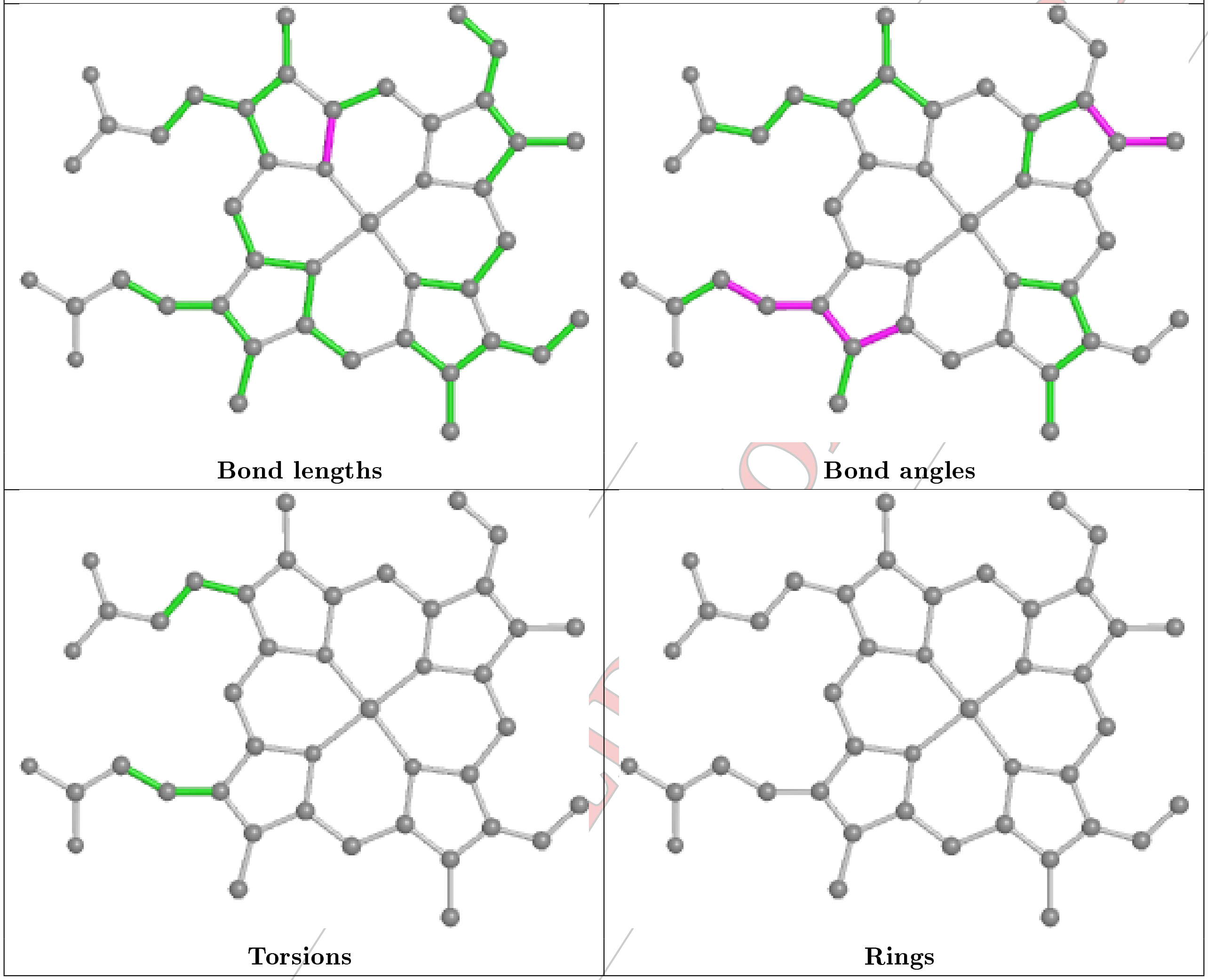

CONFIDENTIAL

Ligand QR8 D 502

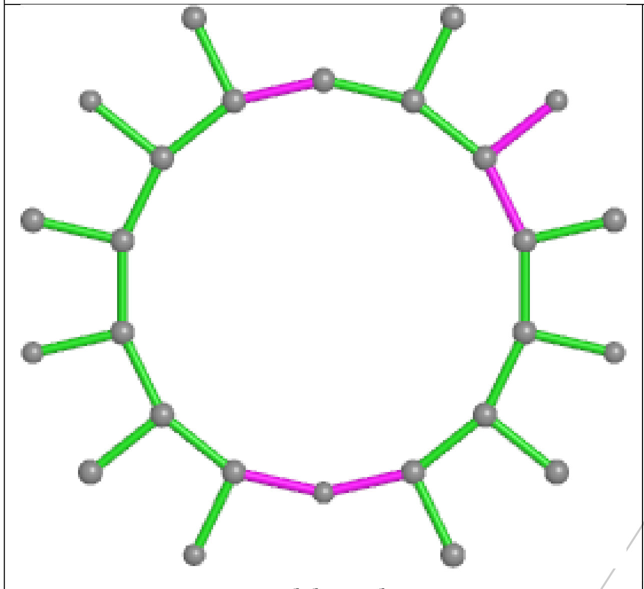

Bond lengths

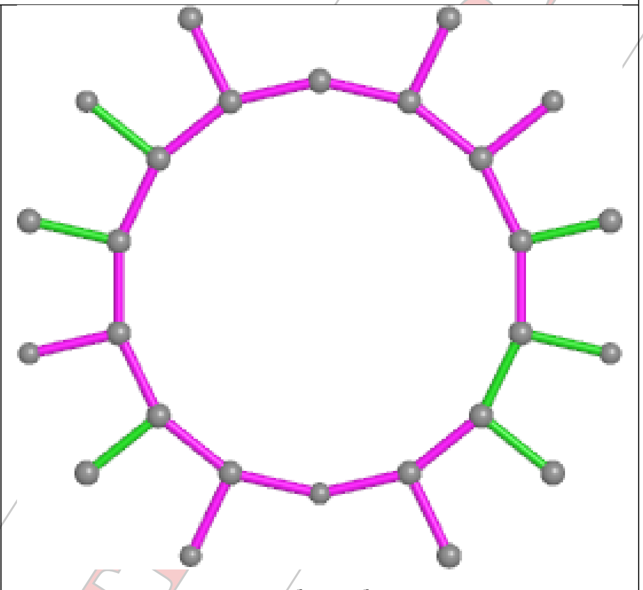

Bond angles

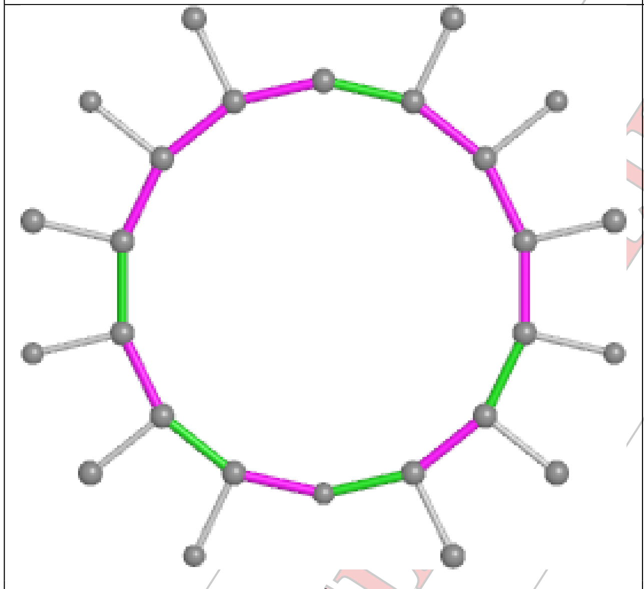

Torsions

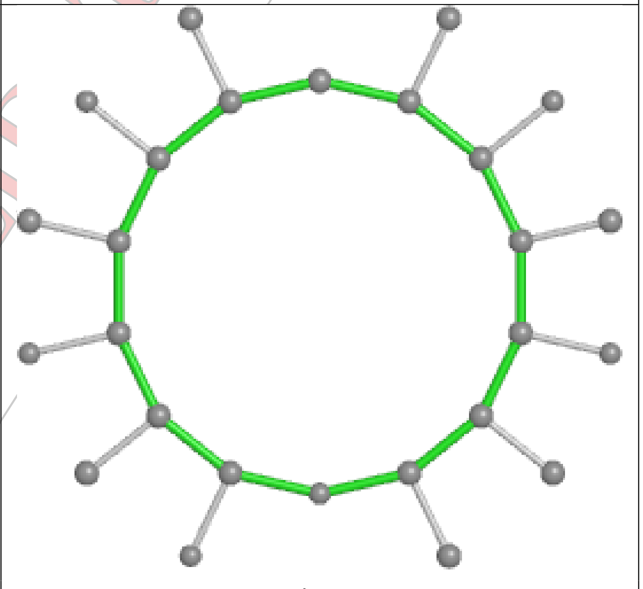

Rings

CONFIDENTIAL DRAFT

Ligand HEM E 501

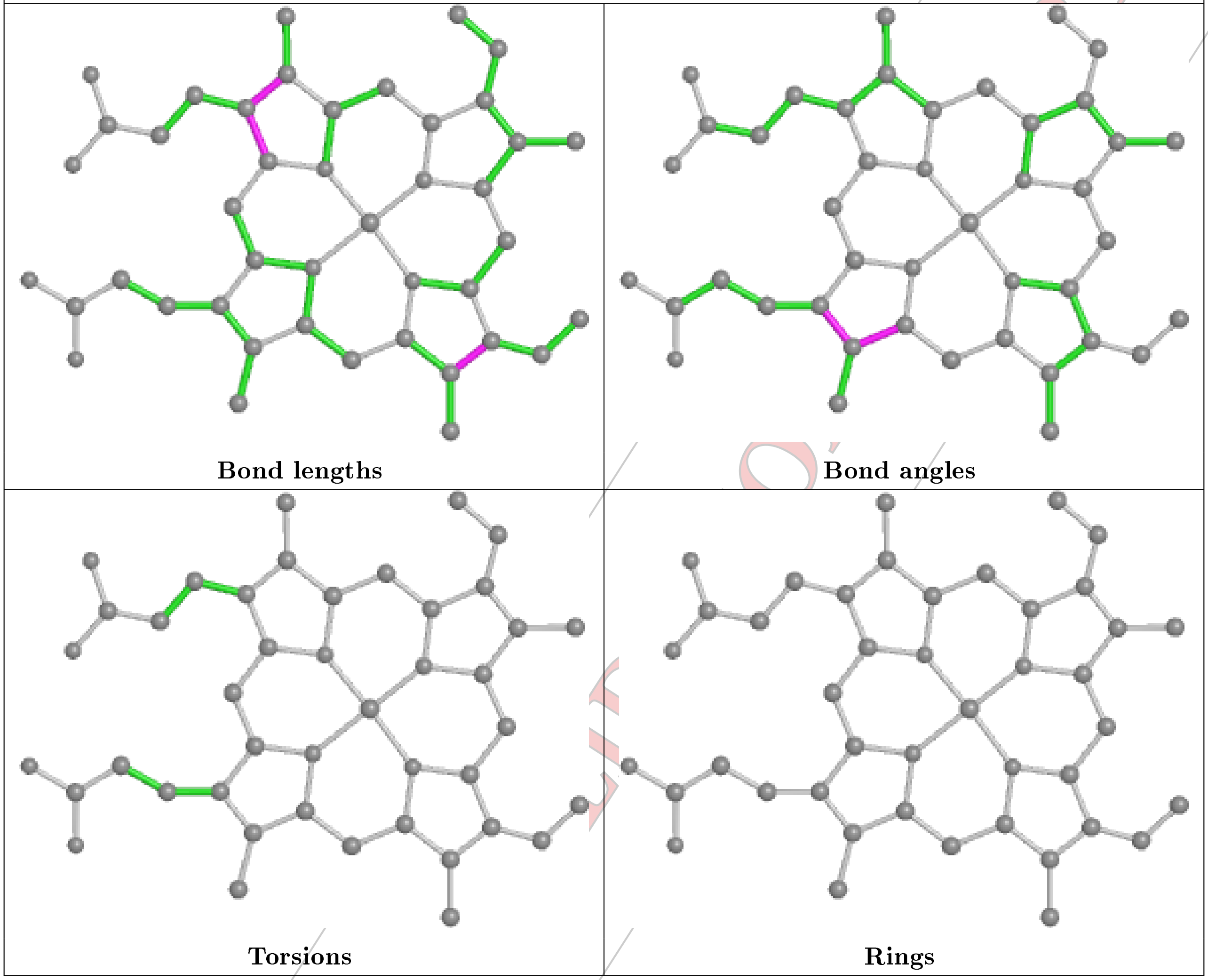

CONFIDENTIAL

Ligand QR8 F 502

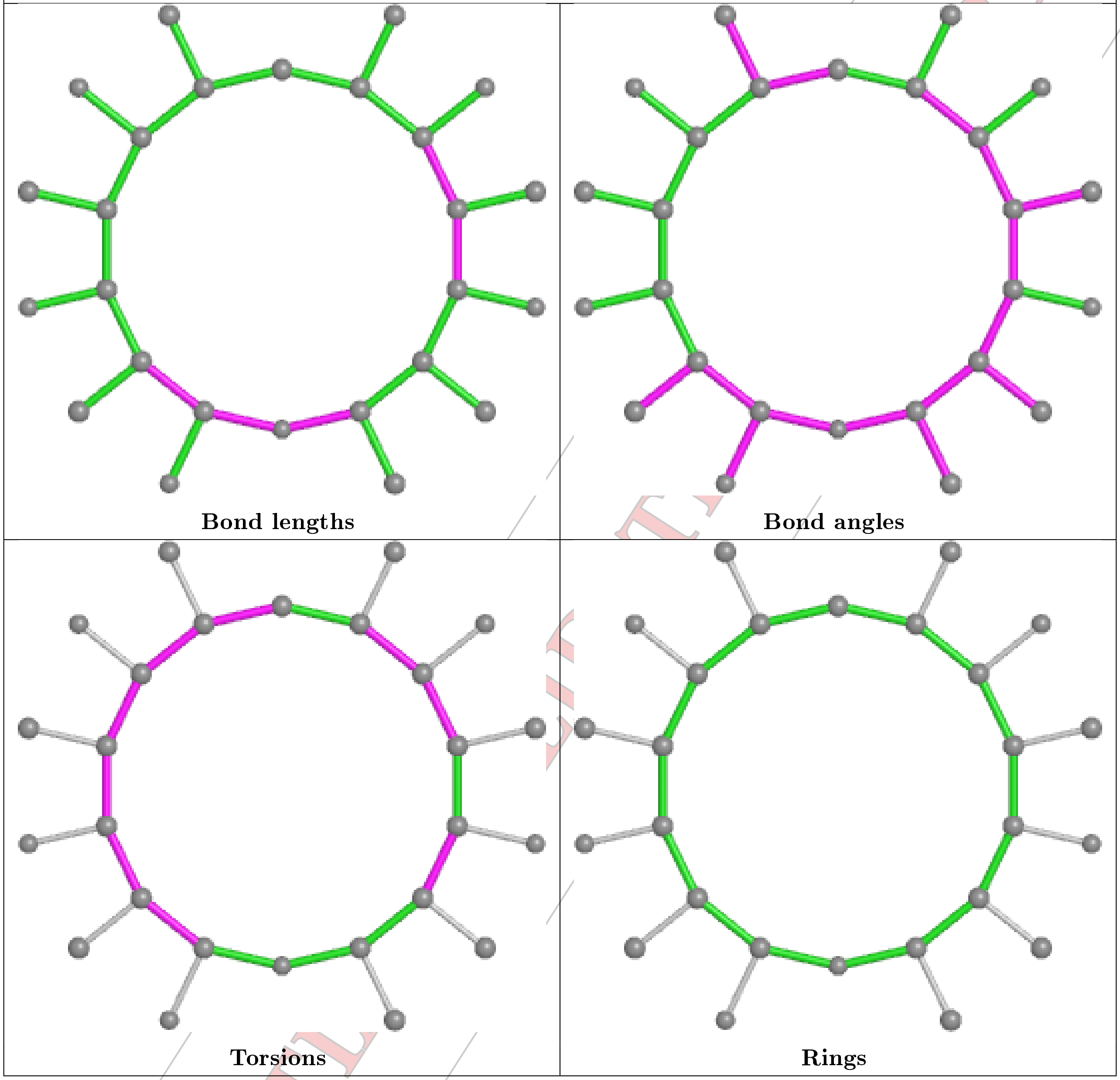

CONFIDENTIAL

Ligand QR8 G 502

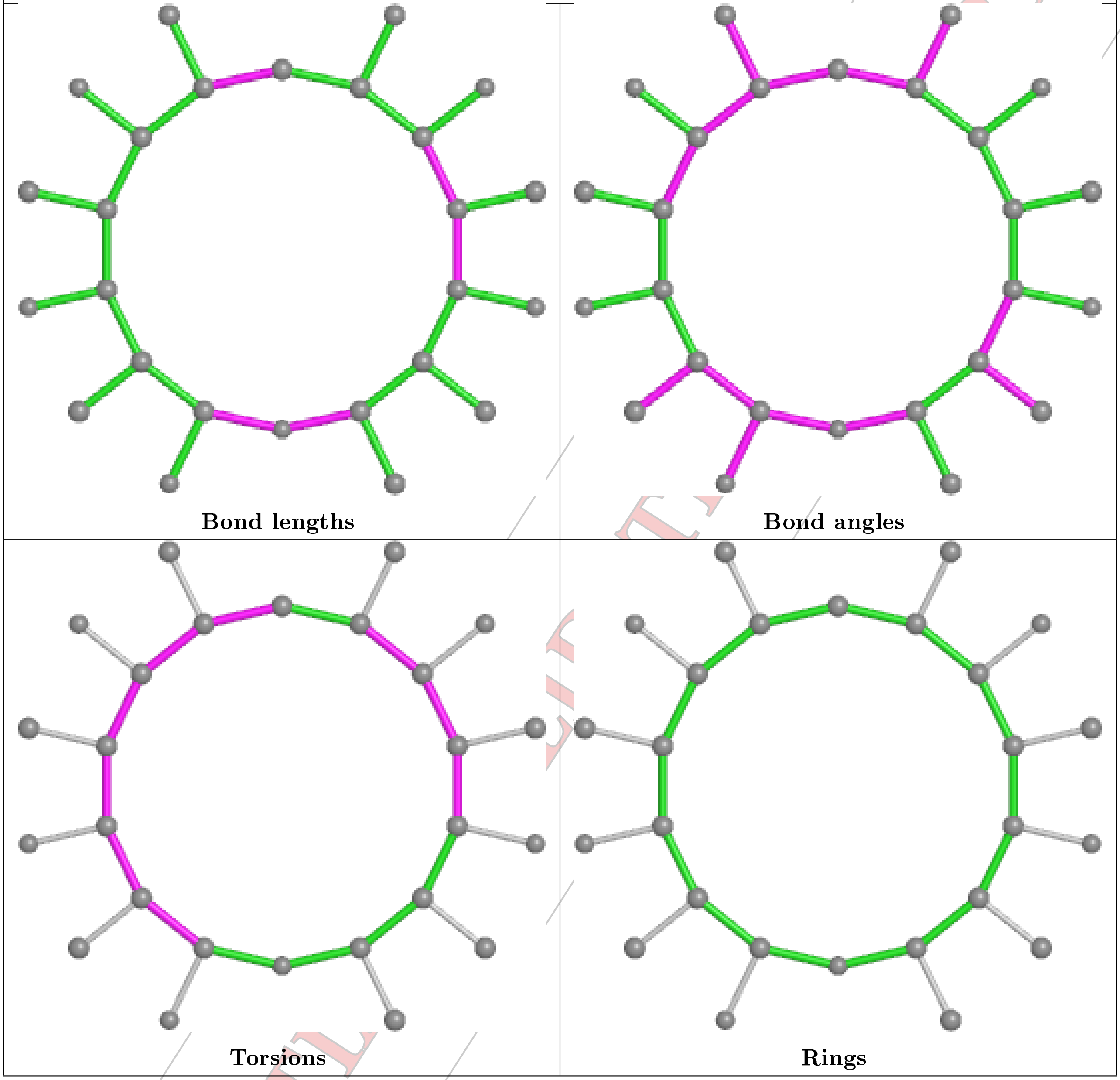

CONFIDENTIAL

Ligand HEM D 501

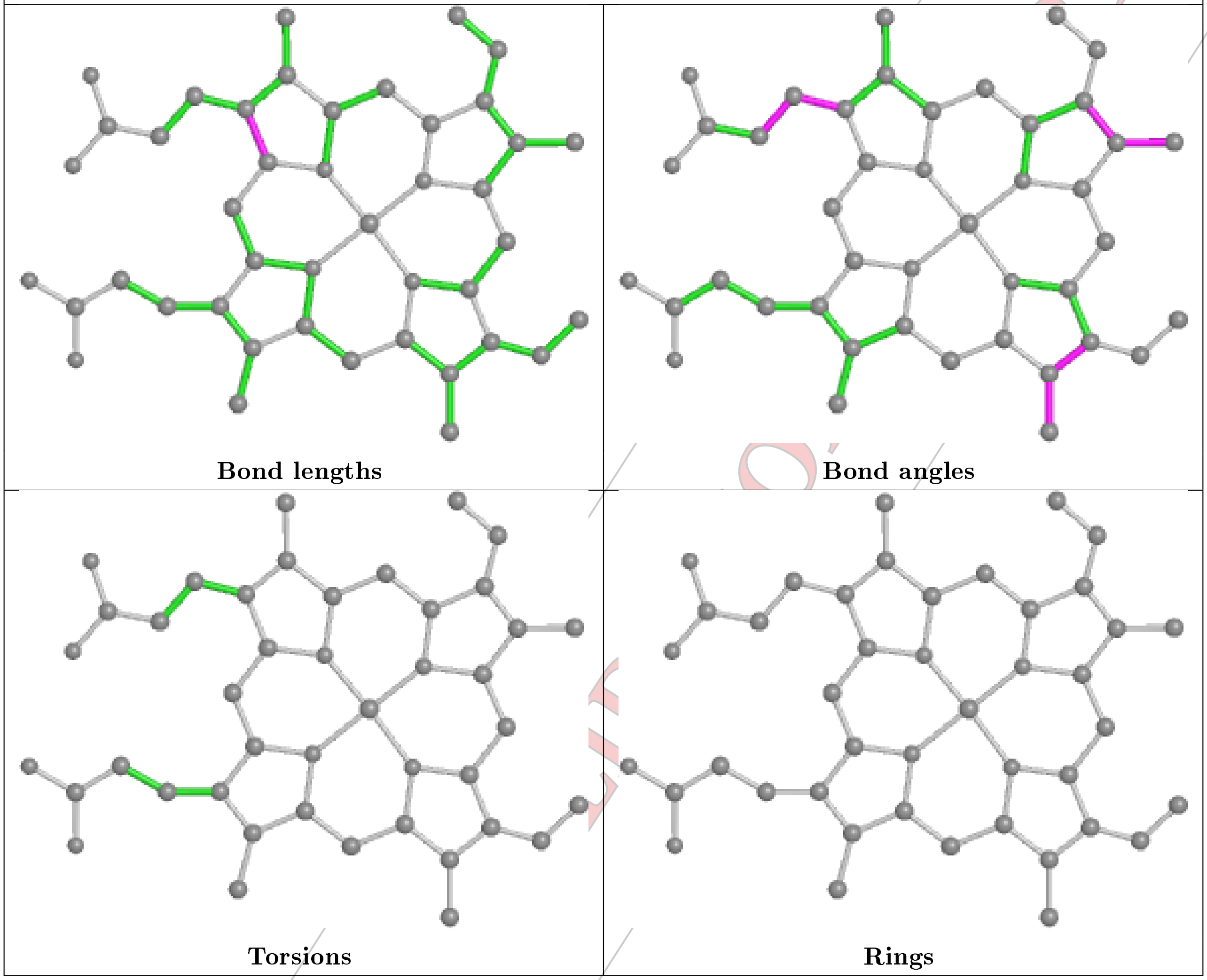

CONFIDENTIAL

Ligand QR8 I 502

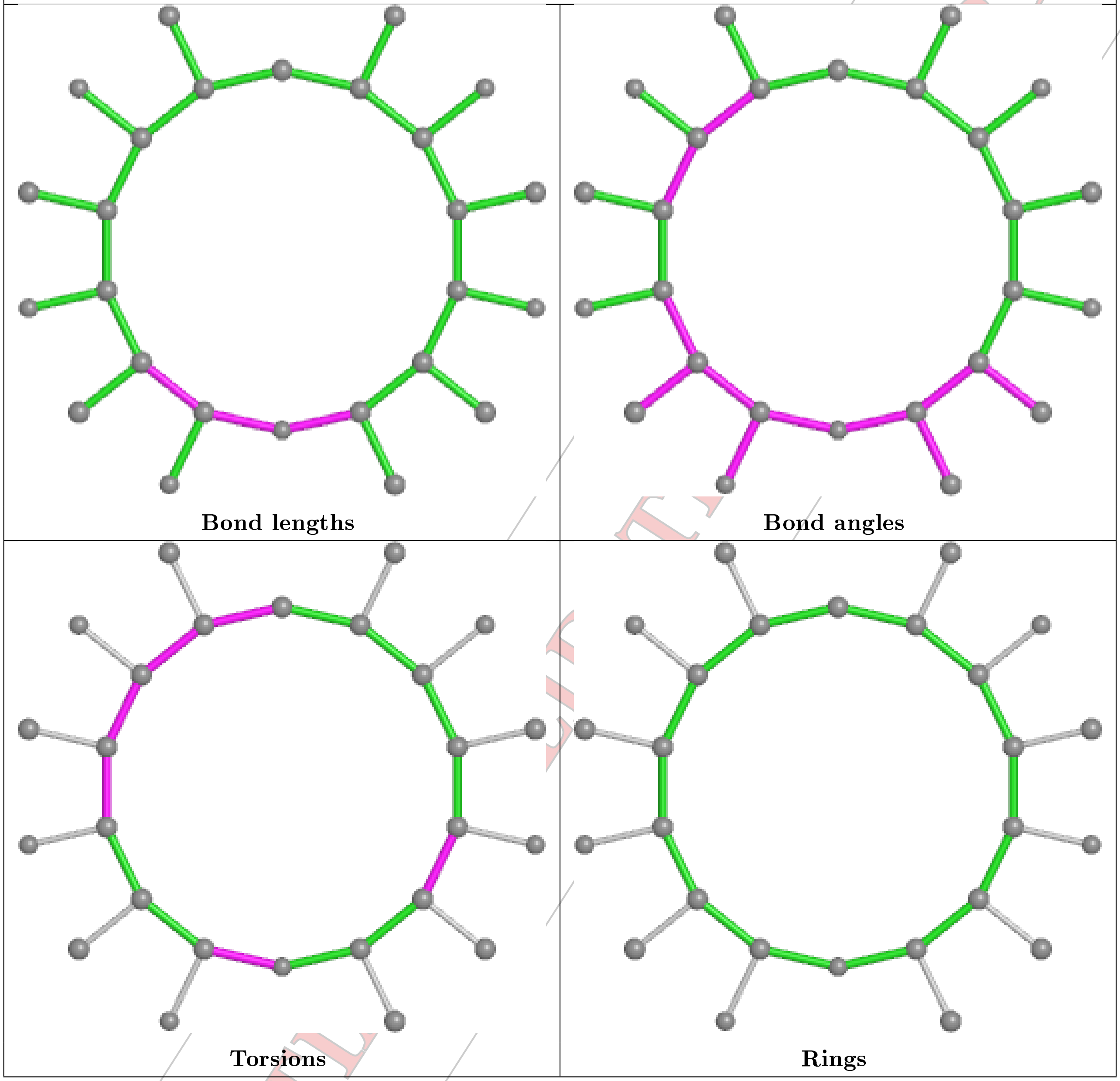

CONFIDENTIAL REPORT

Ligand HEM H 501

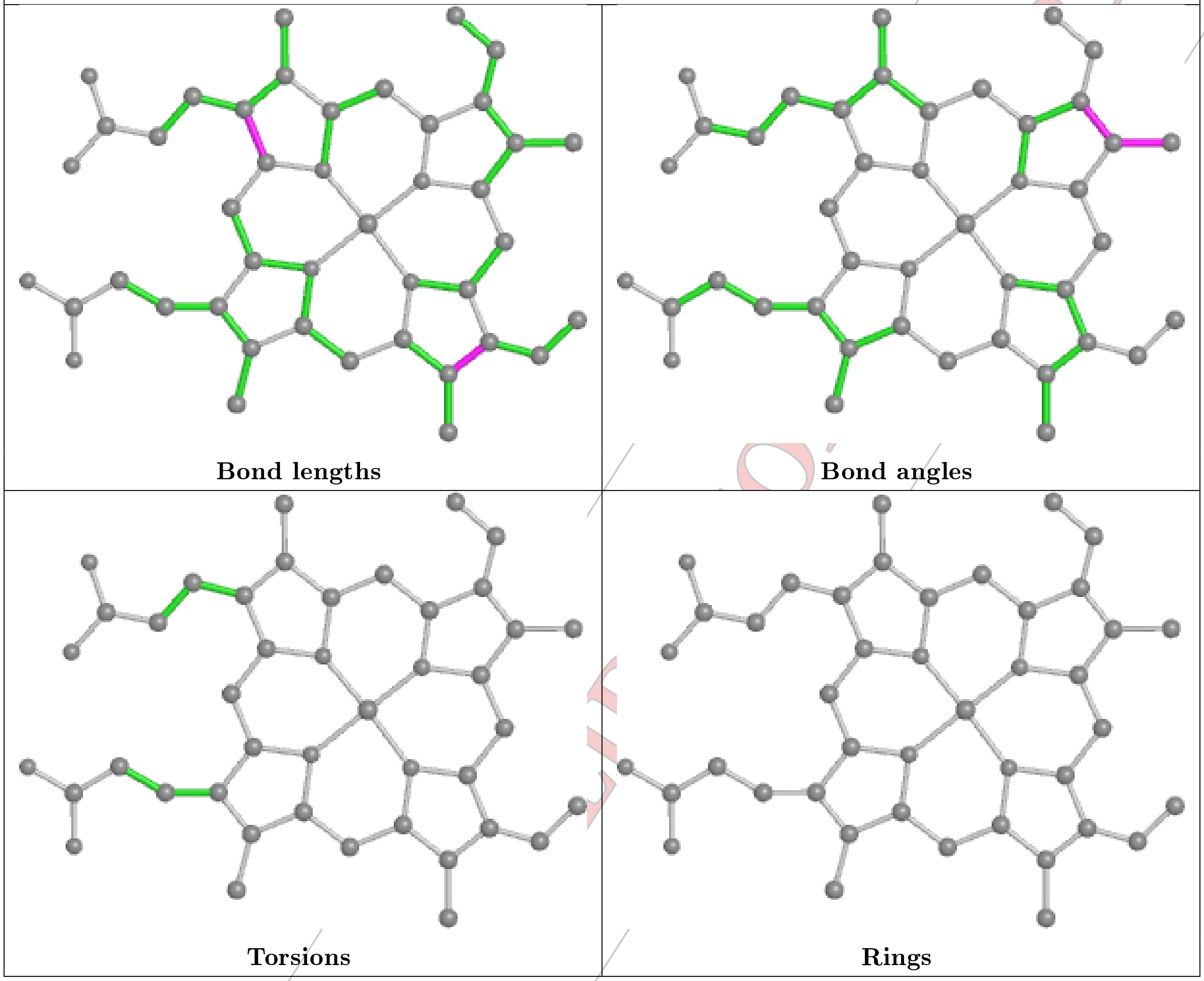

CONFIDENTIAL

Ligand QR8 A 502

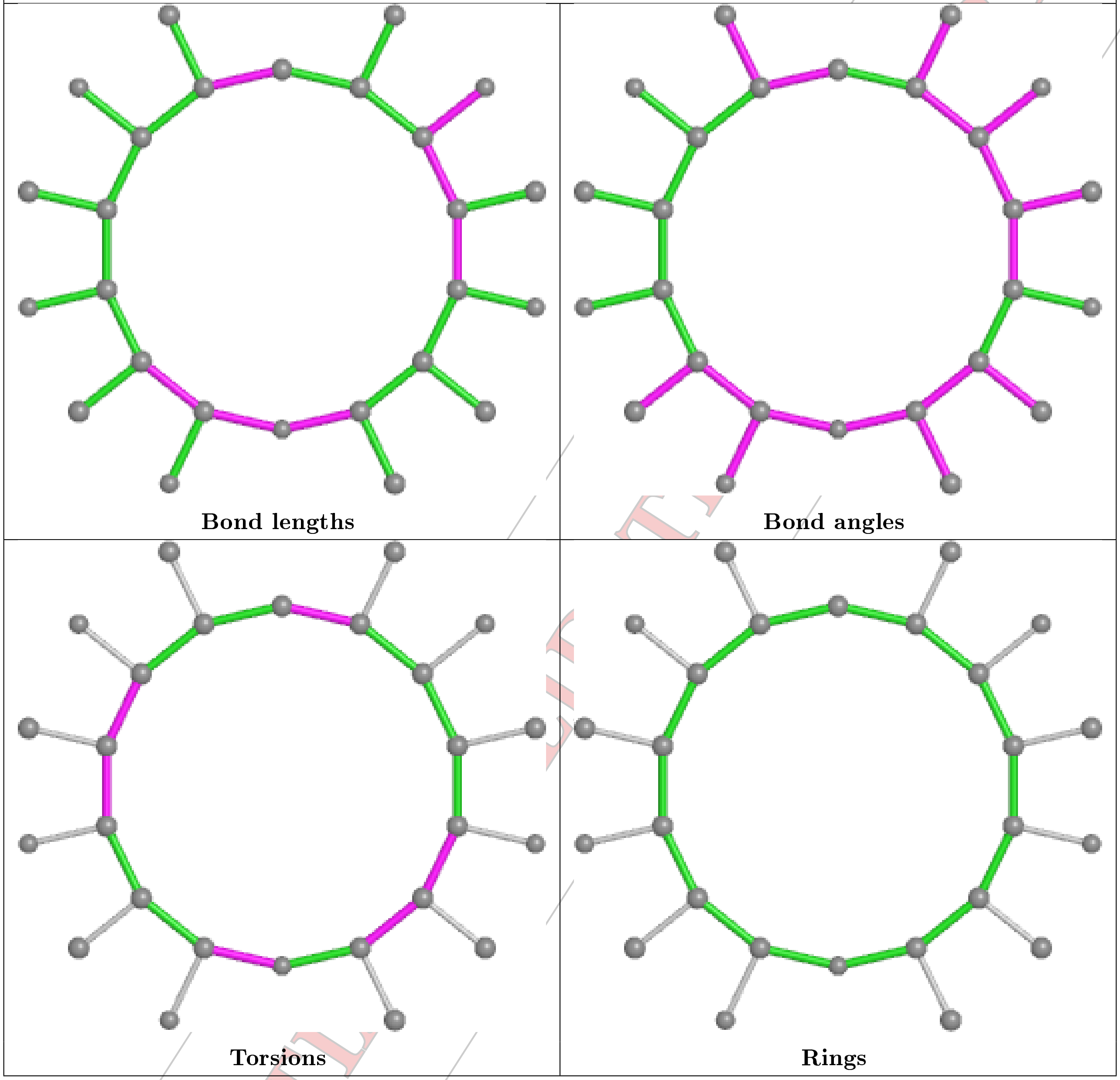

CONFIDENTIAL

Ligand HEM B 501

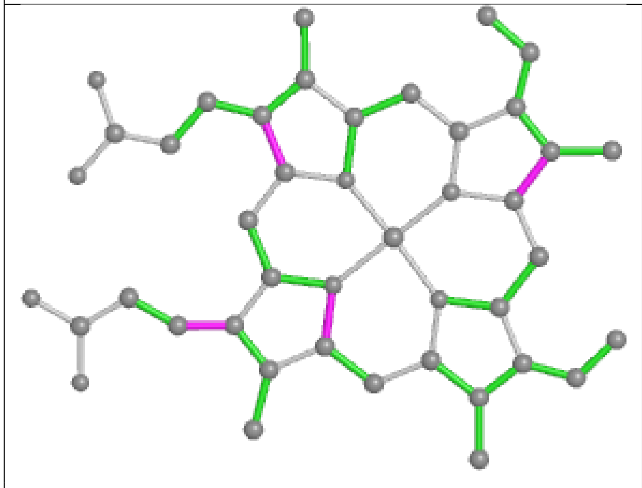

Bond lengths

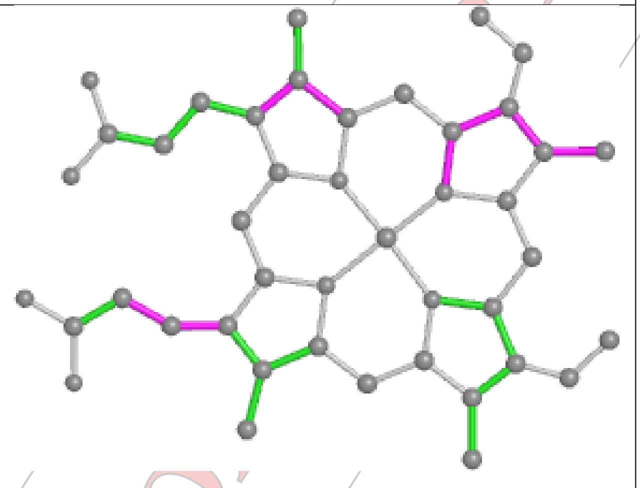

Bond angles

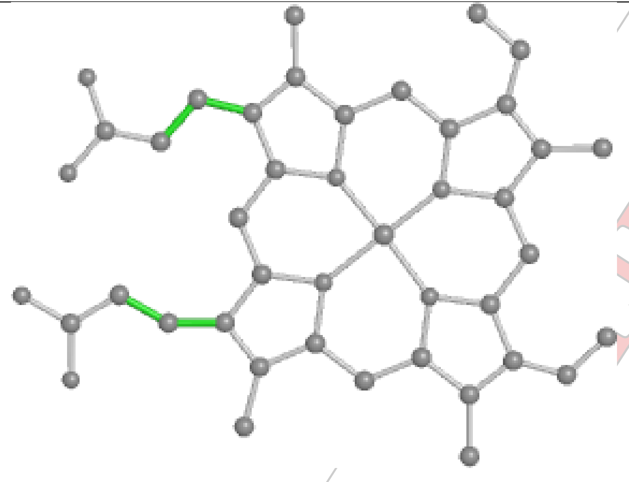

Torsions

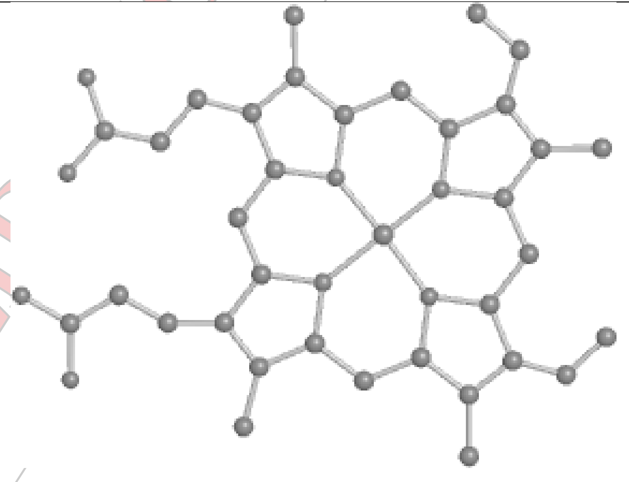

Rings

CONFIDENTIAL

Ligand QR8 C 502

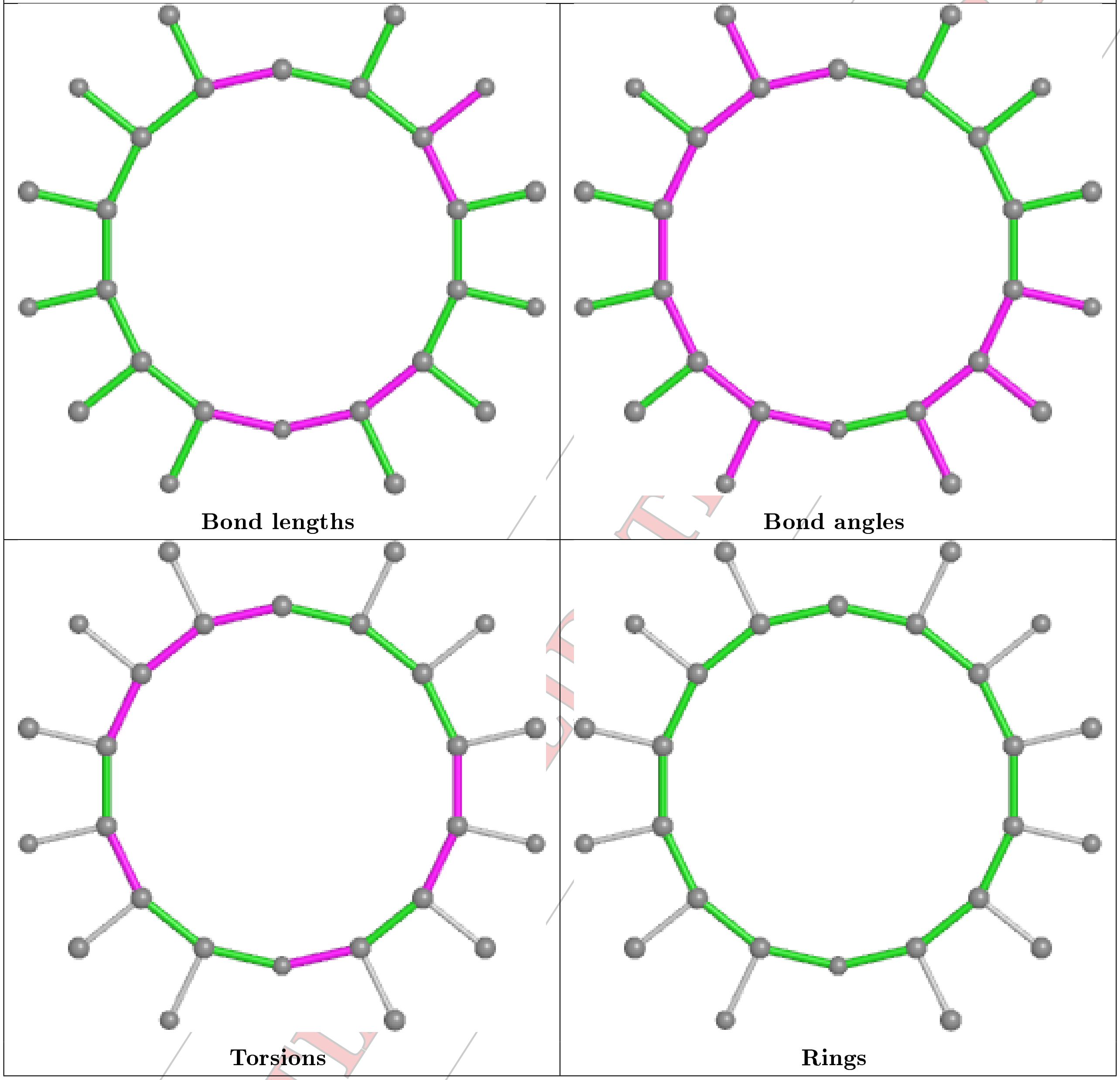

CONFIDENTIAL DRAFT

Ligand QR8 E 502

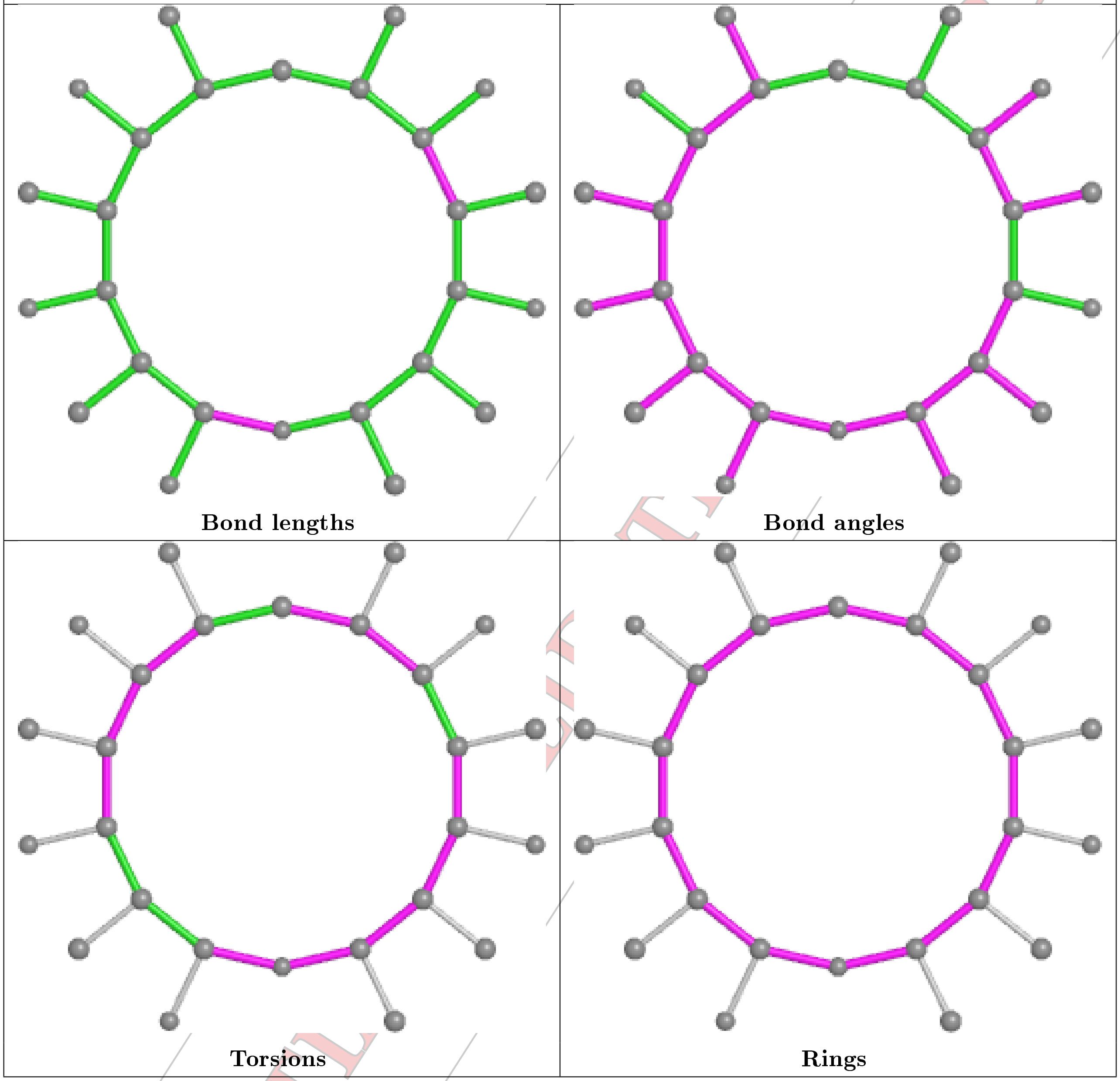

CONFIDENTIAL - MANUSCRIPT

Ligand HEM F 501

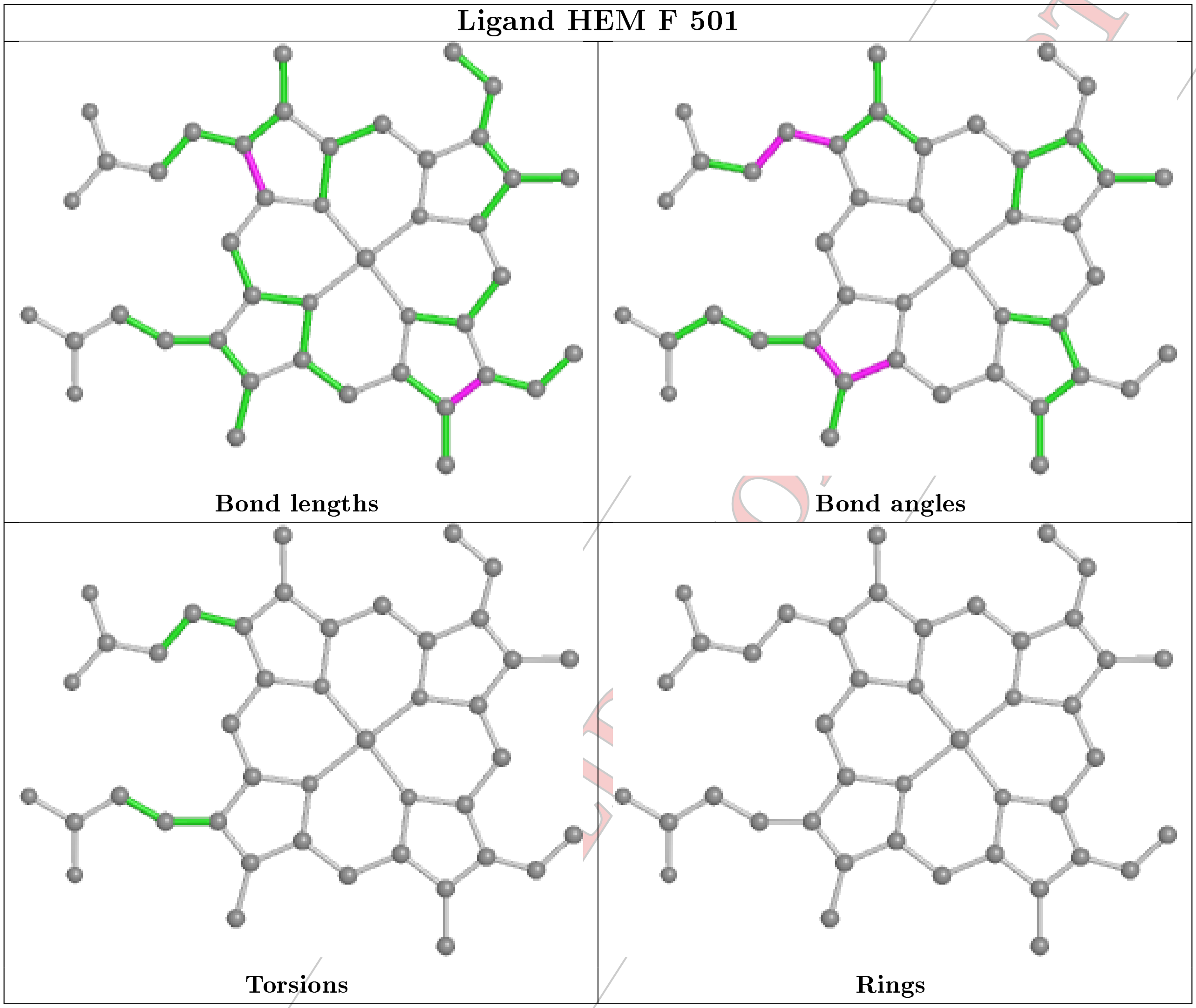

CONFIDENTIAL

Ligand QR8 H 502

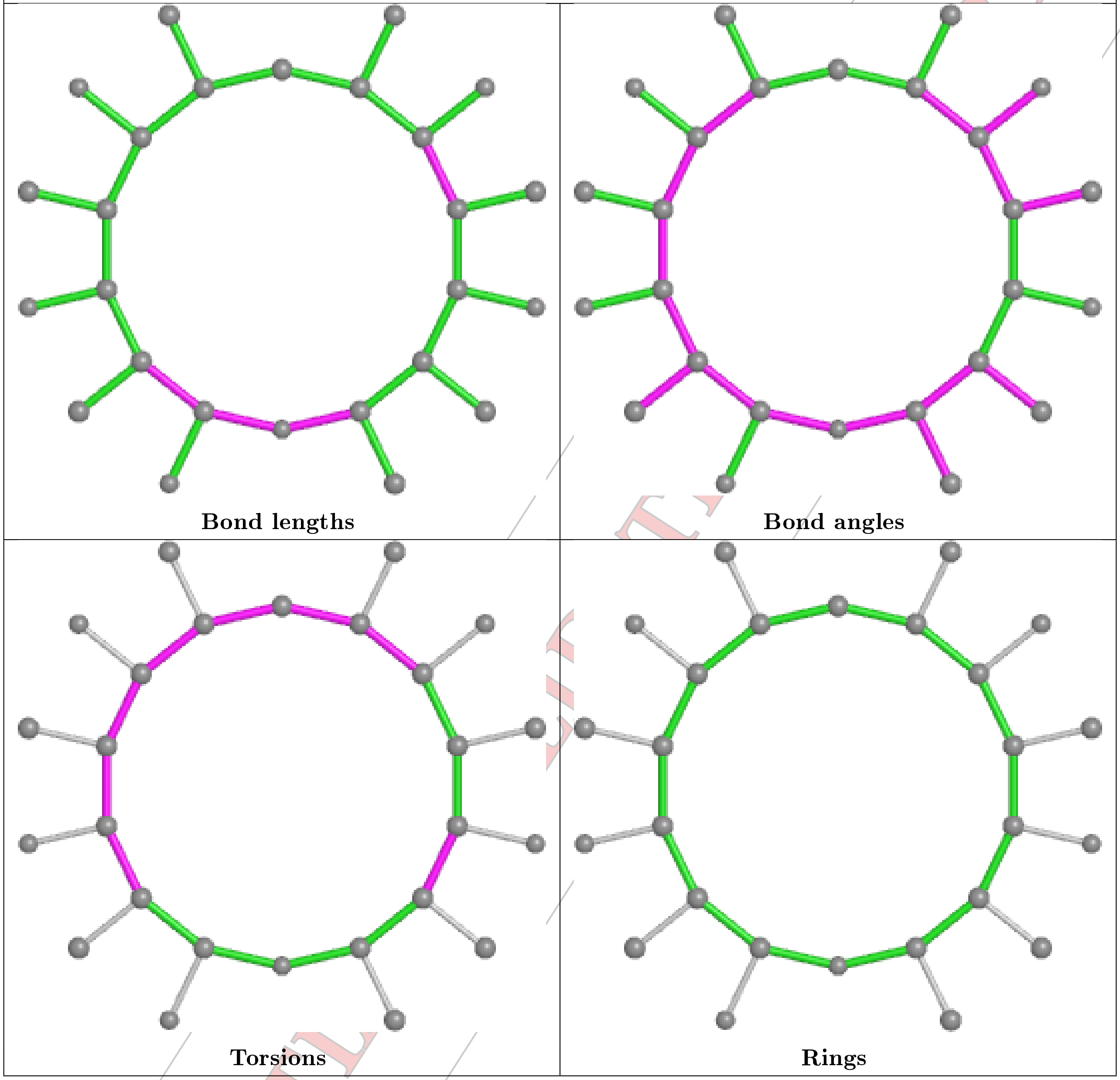

Bond lengths | Bond angles | Torsions | Rings

5.7 Other polymers (i)

There are no such residues in this entry.

5.8 Polymer linkage issues (i)

There are no chain breaks in this entry.

CONFIDENTIAL - DO NOT DISTRIBUTE

6 Fit of model and data (i)

6.1 Protein, DNA and RNA chains (i)

In the following table, the column labelled '#RSRZ> 2' contains the number (and percentage) of RSRZ outliers, followed by percent RSRZ outliers for the chain as percentile scores relative to all X-ray entries and entries of similar resolution. The OWAB column contains the minimum, median, 95th percentile and maximum values of the occupancy-weighted average B-factor per residue. The column labelled 'Q< 0.9' lists the number of (and percentage) of residues with an average occupancy less than 0.9.

Mol	Chain	Analysed	<RSRZ>	#RSRZ>2			OWAB(Å^2)	Q<0.9
1	A	394/407 (96%)	-0.69	0	100	100	9, 28, 77, 129	0
1	B	395/407 (97%)	-0.50	1 (0%)	94	94	16, 46, 84, 116	0
1	C	395/407 (97%)	-0.64	0	100	100	13, 40, 69, 107	0
1	D	394/407 (96%)	-0.61	1 (0%)	94	94	15, 33, 80, 128	0
1	E	386/407 (94%)	-0.24	5 (1%)	77	78	18, 58, 101, 167	0
1	F	394/407 (96%)	-0.47	4 (1%)	82	83	16, 49, 85, 147	0
1	G	381/407 (93%)	-0.01	21 (5%)	25	23	22, 65, 129, 172	0
1	H	395/407 (97%)	-0.39	2 (0%)	91	91	21, 50, 86, 126	0
1	I	248/407 (60%)	1.17	55 (22%)	0	0	69, 109, 148, 182	0
All	All	3382/3663 (92%)	-0.33	89 (2%)	56	56	9, 48, 112, 182	0

All (89) RSRZ outliers are listed below:

Mol	Chain	Res	Type	RSRZ
1	I	394	GLY	7.0
1	G	211	PRO	6.2
1	G	238	GLY	6.1
1	I	209	ASP	6.1
1	I	177	ALA	5.6
1	F	223	THR	5.3
1	G	190	GLN	4.9
1	F	181	SER	4.9
1	I	60	ASP	4.9
1	G	209	ASP	4.7
1	I	171	PHE	4.7
1	I	198	VAL	4.6
1	H	184	LEU	4.6
1	I	107	LEU	4.6
1	G	210	ALA	4.5

Continued on next page...

CONFIDENTIAL VALIDATION REPORT

Continued from previous page...

Mol	Chain	Res	Type	RSRZ
1	I	41	SER	4.5
1	I	202	GLY	4.4
1	I	196	PHE	4.4
1	E	225	ASN	4.3
1	I	59	SER	3.9
1	I	61	ALA	3.9
1	I	211	PRO	3.9
1	I	103	ARG	3.9
1	I	100	ASP	3.8
1	I	174	PHE	3.8
1	I	191	ARG	3.7
1	I	210	ALA	3.7
1	I	116	ARG	3.6
1	G	208	ARG	3.6
1	D	225	ASN	3.5
1	I	56	THR	3.4
1	G	191	ARG	3.3
1	I	179	LEU	3.2
1	I	63	ILE	3.2
1	I	234	ILE	3.2
1	G	202	GLY	3.1
1	I	262	THR	3.1
1	I	221	LEU	3.1
1	I	40	VAL	3.1
1	I	205	ALA	3.1
1	G	174	PHE	3.1
1	I	204	VAL	3.0
1	I	231	LYS	3.0
1	I	207	ARG	3.0
1	I	170	LEU	3.0
1	I	212	THR	3.0
1	E	187	ALA	2.9
1	I	232	GLY	2.9
1	G	217	GLY	2.9
1	I	236	ASN	2.9
1	I	203	LEU	2.9
1	I	87	PRO	2.8
1	I	173	THR	2.8
1	G	235	VAL	2.7
1	I	199	TYR	2.7
1	E	189	ILE	2.7
1	I	369	ALA	2.7

Continued on next page...

CONFIDENTIAL VALIDATION REPORT

Continued from previous page...

Mol	Chain	Res	Type	RSRZ
1	G	199	TYR	2.6
1	I	269	SER	2.6
1	I	367	GLN	2.6
1	E	36	ARG	2.6
1	G	89	GLU	2.6
1	I	161	LEU	2.6
1	I	122	PRO	2.6
1	G	187	ALA	2.6
1	I	164	PRO	2.6
1	G	106	ARG	2.6
1	I	64	VAL	2.5
1	I	230	THR	2.5
1	G	48	GLY	2.5
1	F	183	ARG	2.4
1	I	393	GLN	2.4
1	G	299	VAL	2.4
1	G	194	GLN	2.3
1	I	190	GLN	2.3
1	I	136	VAL	2.3
1	I	169	ASP	2.3
1	I	58	MET	2.2
1	G	188	GLU	2.2
1	B	13	ALA	2.2
1	F	187	ALA	2.2
1	I	42	ARG	2.2
1	G	195	ASP	2.2
1	I	272	ALA	2.1
1	I	110	LYS	2.1
1	H	223	THR	2.1
1	E	209	ASP	2.1
1	I	379	THR	2.0
1	G	196	PHE	2.0

6.2 Non-standard residues in protein, DNA, RNA chains (i)

There are no non-standard protein/DNA/RNA residues in this entry.

6.3 Carbohydrates (i)

There are no monosaccharides in this entry.

CONFIDENTIAL VALIDATION REPORT

6.4 Ligands ⓘ

In the following table, the Atoms column lists the number of modelled atoms in the group and the number defined in the chemical component dictionary. The B-factors column lists the minimum, median, 95th percentile and maximum values of B factors of atoms in the group. The column labelled 'Q< 0.9' lists the number of atoms with occupancy less than 0.9.

Mol	Type	Chain	Res	Atoms	RSCC	RSR	B-factors(Å^2)	Q<0.9
2	HEM	I	501	43/43	0.86	0.24	88,152,169,172	0
3	QR8	I	502	26/26	0.86	0.26	82,110,131,138	0
3	QR8	G	502	26/26	0.87	0.25	46,57,78,85	0
3	QR8	H	502	26/26	0.87	0.20	38,45,52,56	0
3	QR8	F	502	26/26	0.88	0.23	49,53,61,67	0
3	QR8	E	502	26/26	0.89	0.21	44,74,99,102	0
3	QR8	A	502	26/26	0.90	0.20	27,33,39,44	0
3	QR8	D	502	26/26	0.93	0.16	36,46,52,52	0
2	HEM	F	501	43/43	0.95	0.20	35,68,79,81	0
3	QR8	B	502	26/26	0.95	0.16	28,31,36,42	0
2	HEM	H	501	43/43	0.96	0.19	26,57,69,75	0
2	HEM	B	501	43/43	0.96	0.19	28,33,42,46	0
3	QR8	C	502	26/26	0.96	0.14	16,18,19,21	0
2	HEM	G	501	43/43	0.97	0.20	47,53,67,70	0
2	HEM	E	501	43/43	0.97	0.22	34,39,48,54	0
2	HEM	D	501	43/43	0.98	0.13	19,24,40,48	0
2	HEM	C	501	43/43	0.98	0.17	21,23,28,33	0
2	HEM	A	501	43/43	0.98	0.11	7,8,9,10	0

The following is a graphical depiction of the model fit to experimental electron density of all instances of the Ligand of Interest. In addition, ligands with molecular weight > 250 and outliers as shown on the geometry validation Tables will also be included. Each fit is shown from different orientation to approximate a three-dimensional view.

CONFIDENTIAL VALIDATION REPORT

Electron density around HEM I 501:

2mF$_o$-DF$_c$ (at 0.7 rmsd) in gray
mF$_o$-DF$_c$ (at 3 rmsd) in purple (negative)
and green (positive)

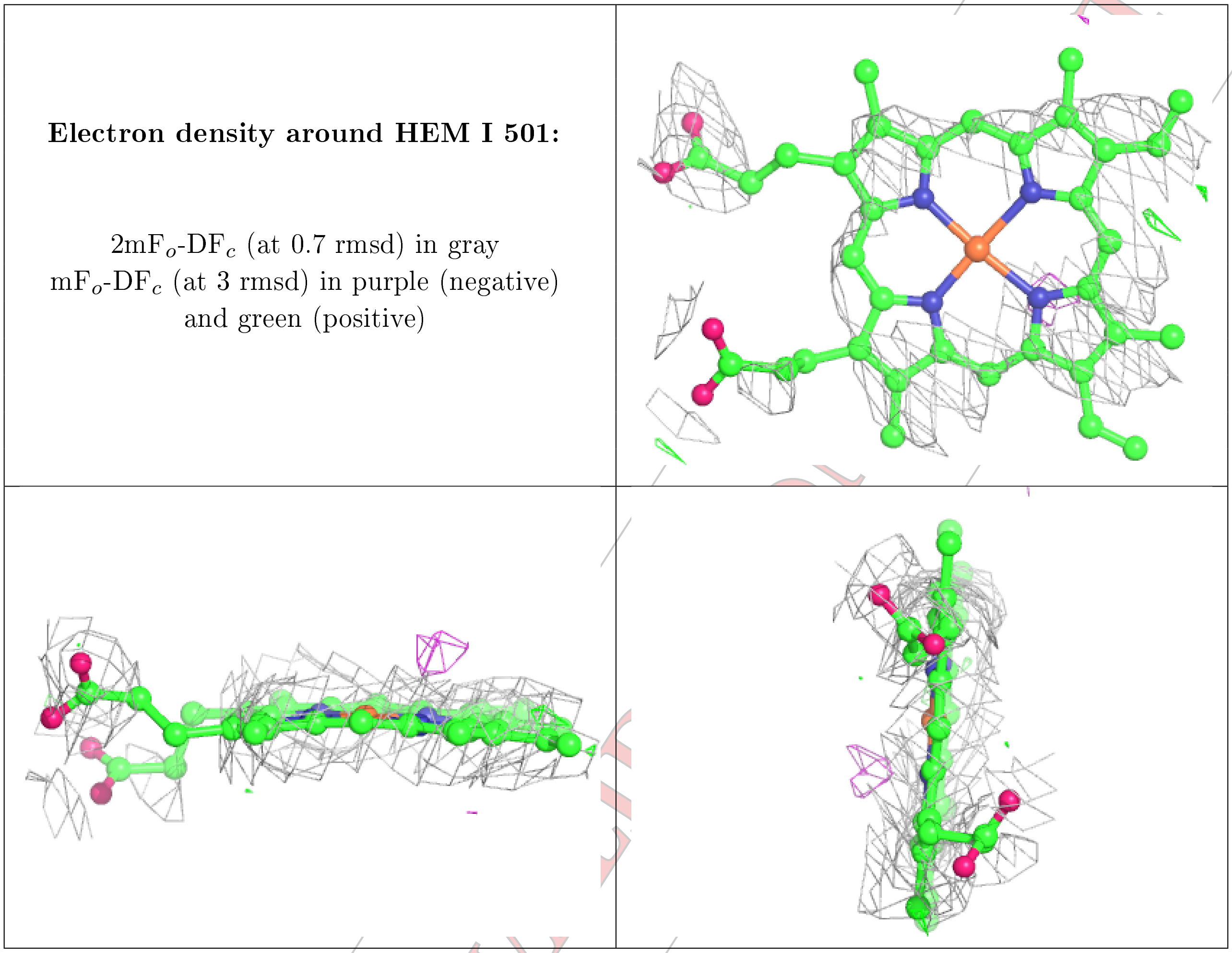

CONFIDENTIAL VALIDATION REPORT

Electron density around QR8 I 502:

$2mF_o$-DF_c (at 0.7 rmsd) in gray
mF_o-DF_c (at 3 rmsd) in purple (negative)
and green (positive)

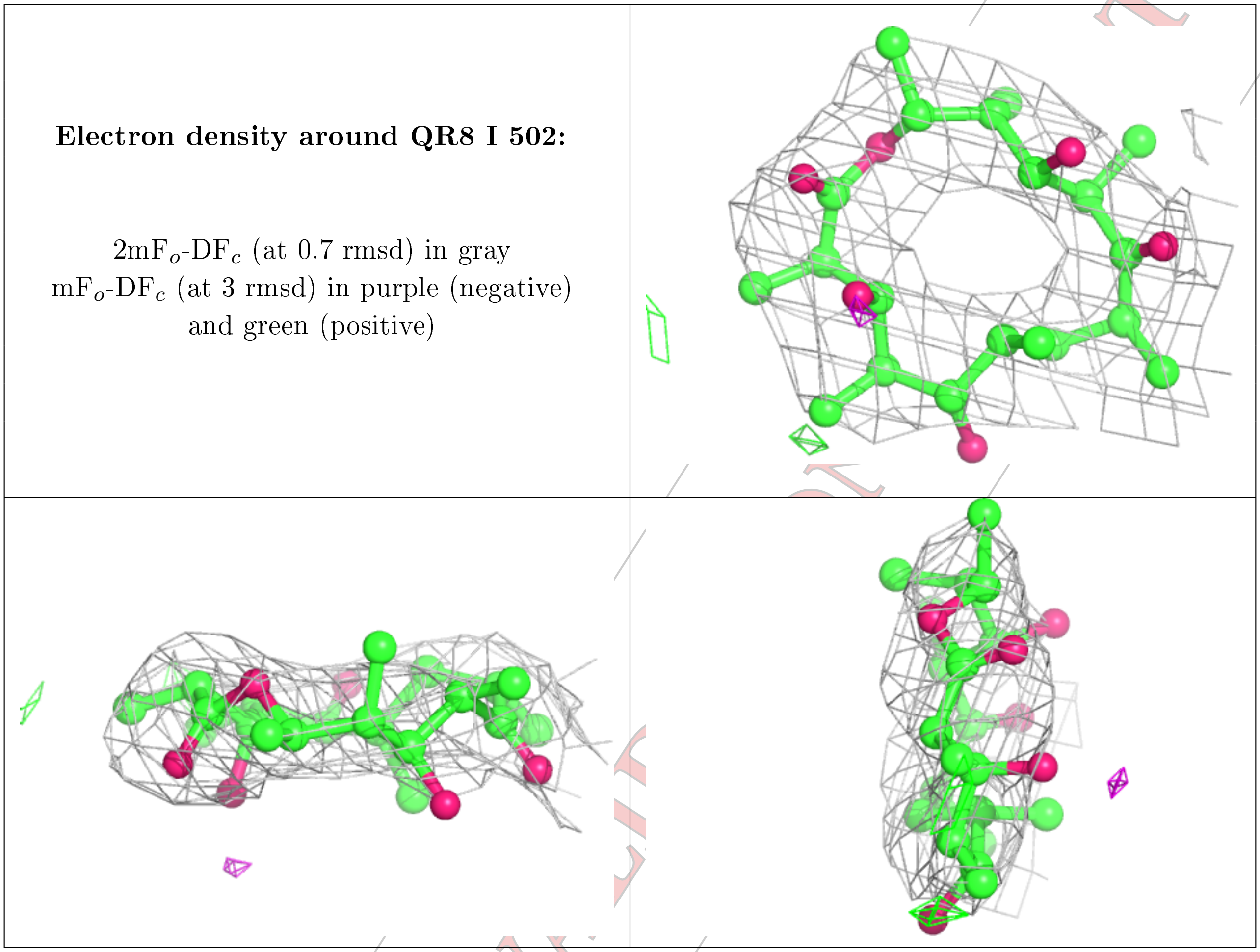

CONFIDENTIAL VALIDATION REPORT

Electron density around QR8 G 502:

2mF$_o$-DF$_c$ (at 0.7 rmsd) in gray
mF$_o$-DF$_c$ (at 3 rmsd) in purple (negative)
and green (positive)

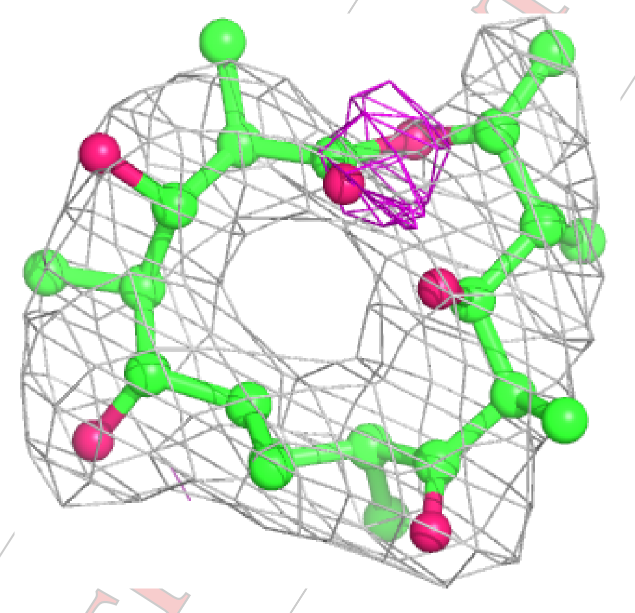

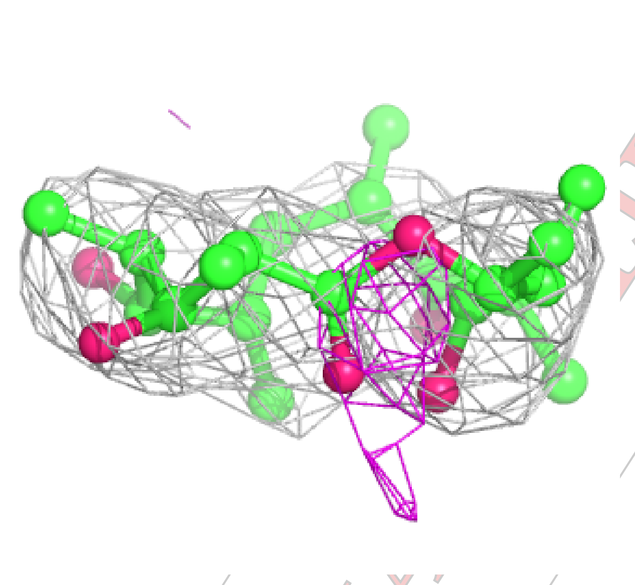

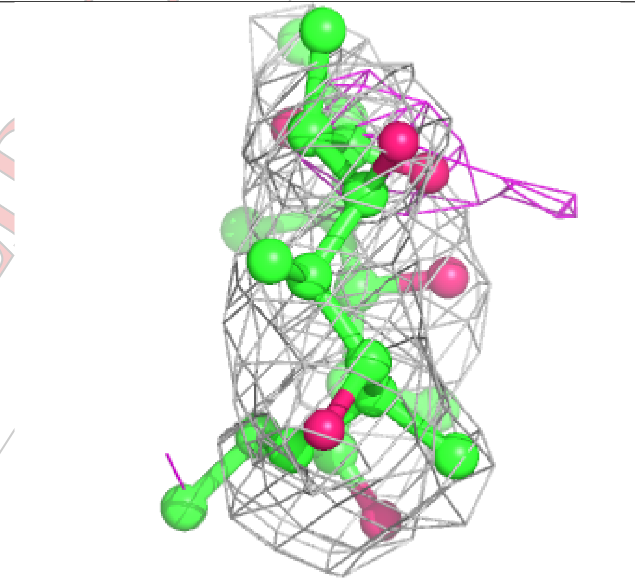

CONFIDENTIAL

Electron density around QR8 H 502:

$2mF_o$-DF_c (at 0.7 rmsd) in gray
mF_o-DF_c (at 3 rmsd) in purple (negative)
and green (positive)

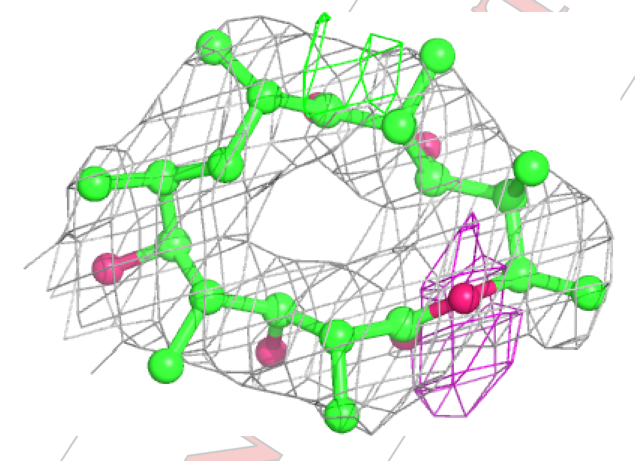

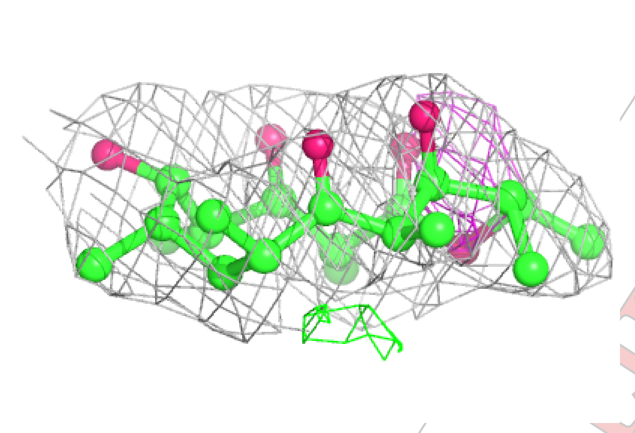

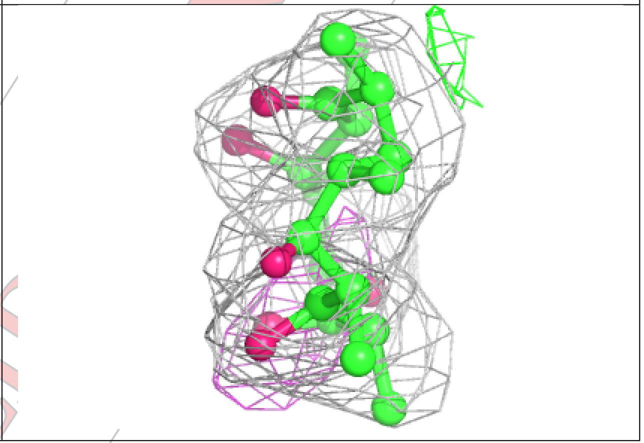

CONFIDENTIAL

Electron density around QR8 F 502:

$2mF_o$-DF_c (at 0.7 rmsd) in gray
mF_o-DF_c (at 3 rmsd) in purple (negative)
and green (positive)

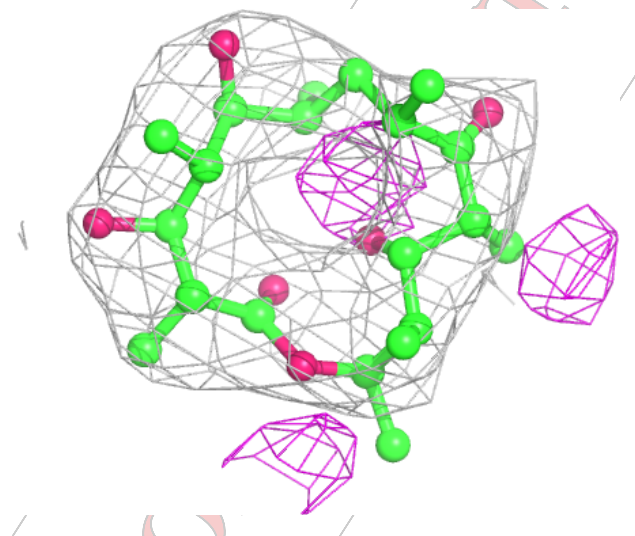

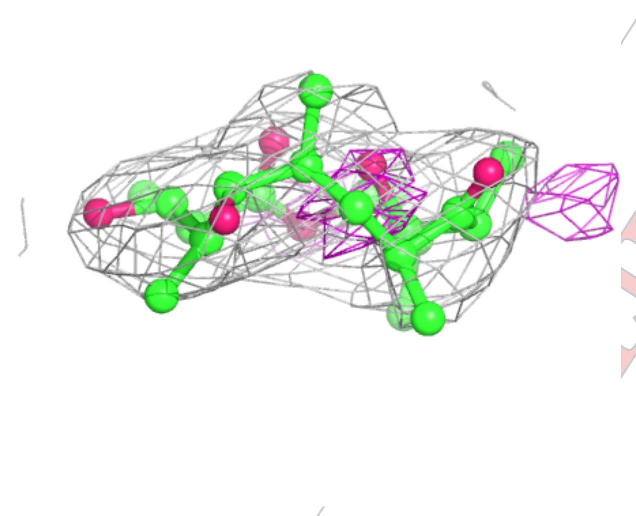

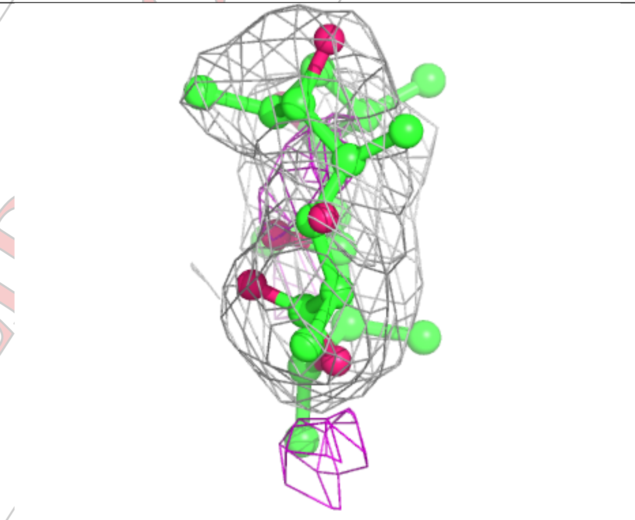

CONFIDENTIAL

Electron density around QR8 E 502:

$2mF_o$-DF_c (at 0.7 rmsd) in gray
mF_o-DF_c (at 3 rmsd) in purple (negative)
and green (positive)

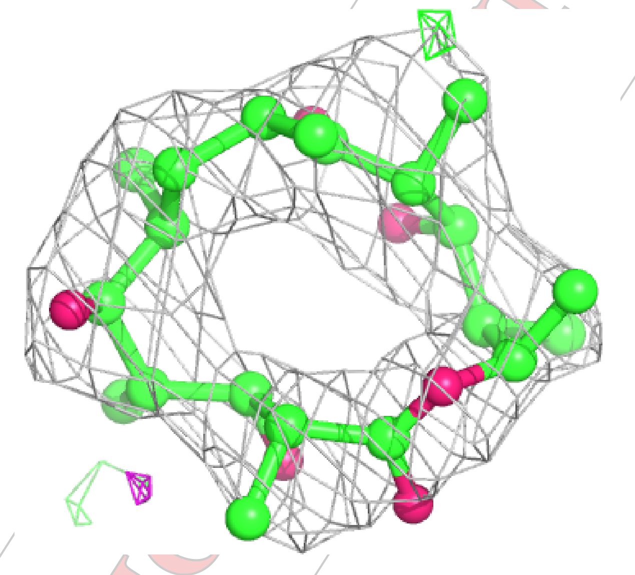

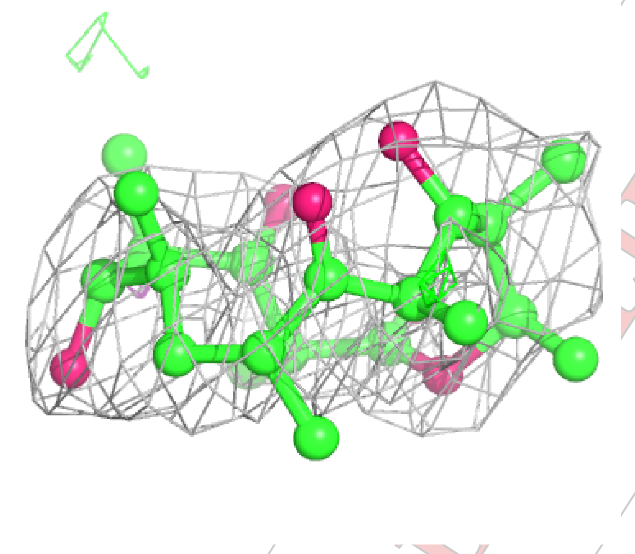

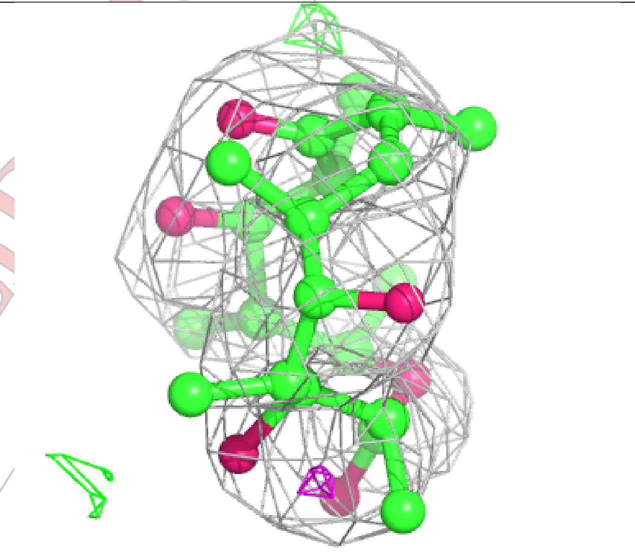

CONFIDENTIAL

Electron density around QR8 A 502:

$2mF_o$-DF_c (at 0.7 rmsd) in gray
mF_o-DF_c (at 3 rmsd) in purple (negative)
and green (positive)

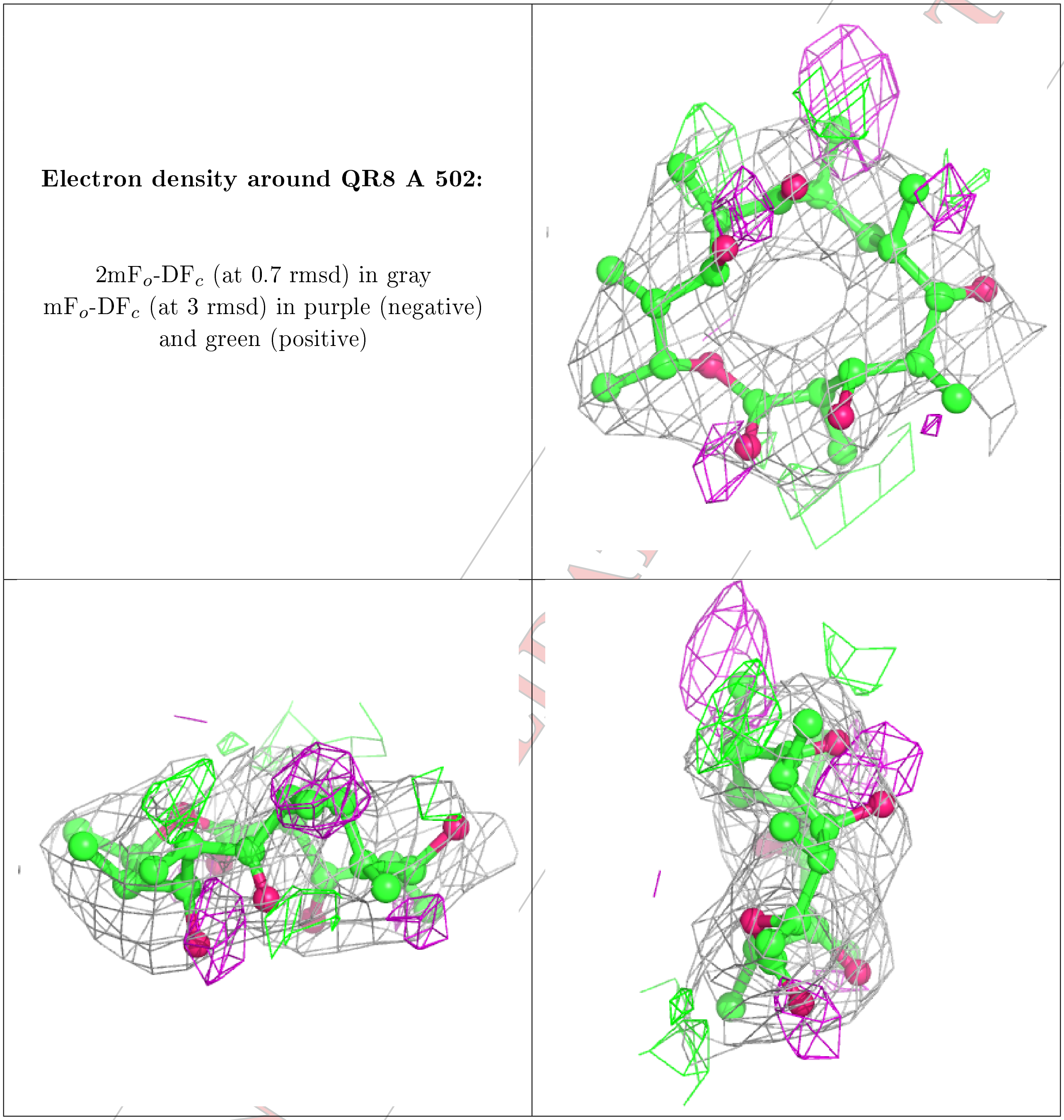

CONFIDENTIAL

Electron density around QR8 D 502:

2mF$_o$-DF$_c$ (at 0.7 rmsd) in gray
mF$_o$-DF$_c$ (at 3 rmsd) in purple (negative)
and green (positive)

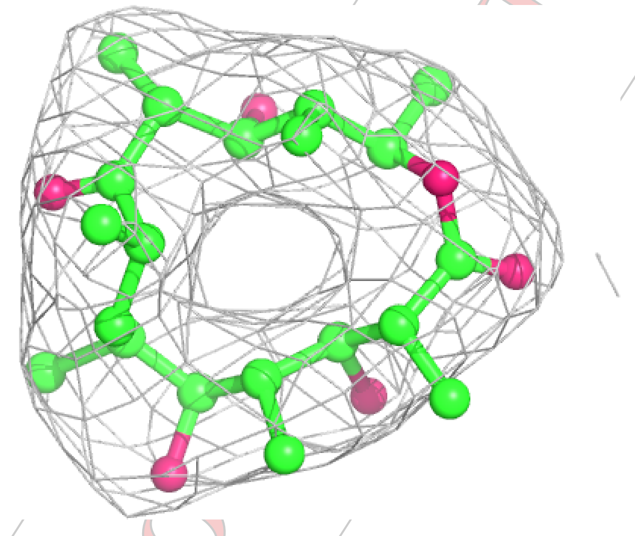

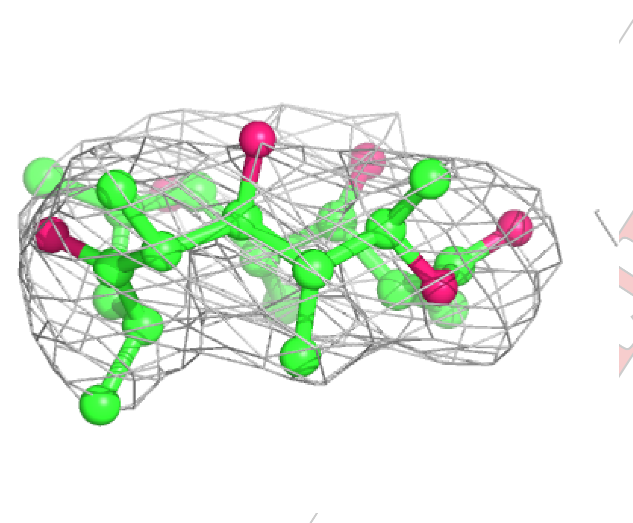

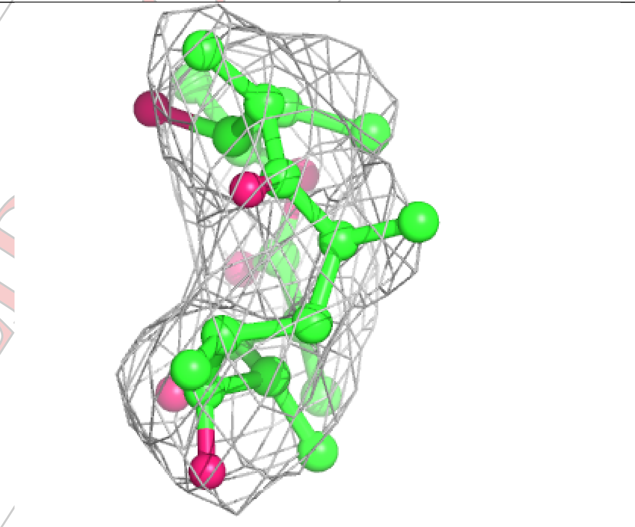

CONFIDENTIAL

Electron density around HEM F 501:

$2mF_o$-DF_c (at 0.7 rmsd) in gray
mF_o-DF_c (at 3 rmsd) in purple (negative)
and green (positive)

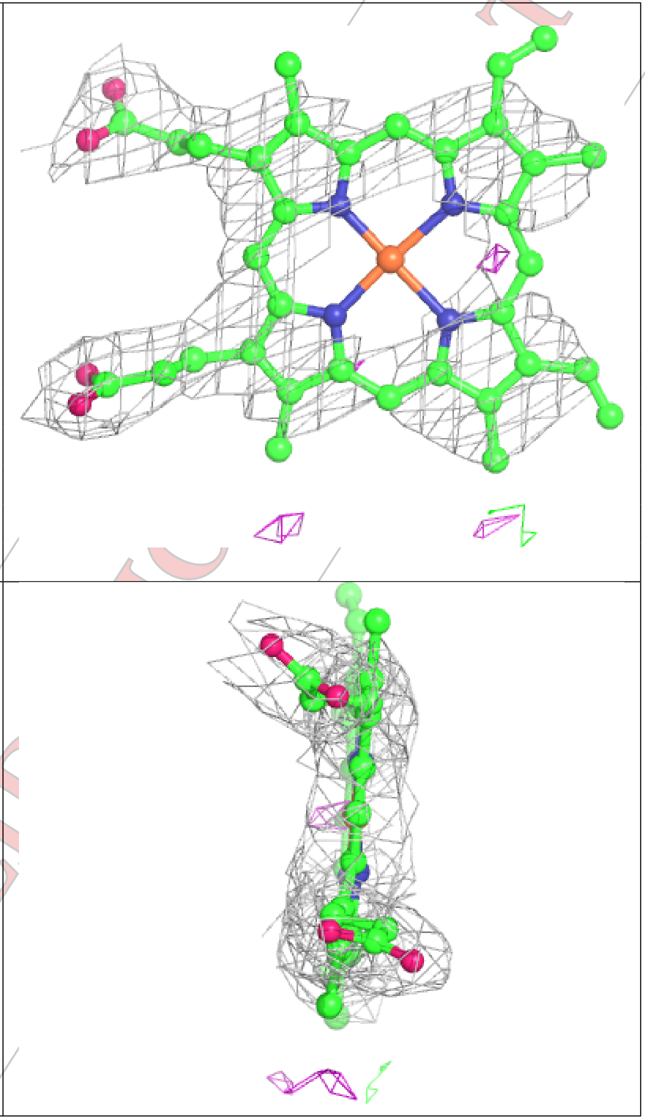

CONFIDENTIAL

Electron density around QR8 B 502:

$2mF_o$-DF_c (at 0.7 rmsd) in gray
mF_o-DF_c (at 3 rmsd) in purple (negative) and green (positive)

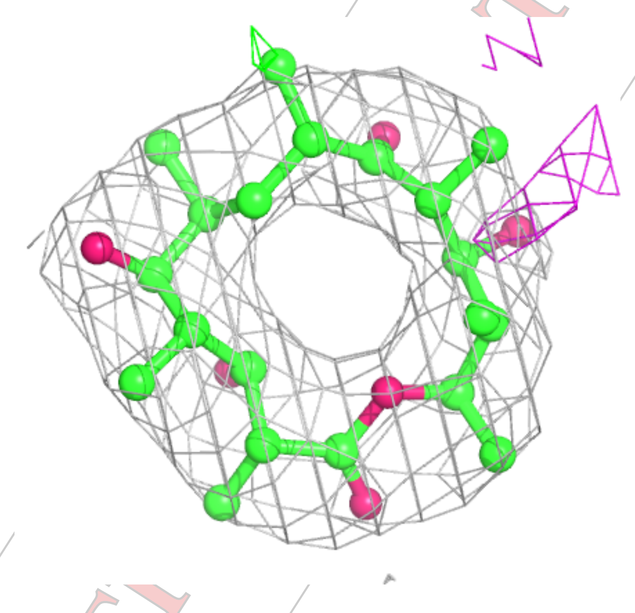

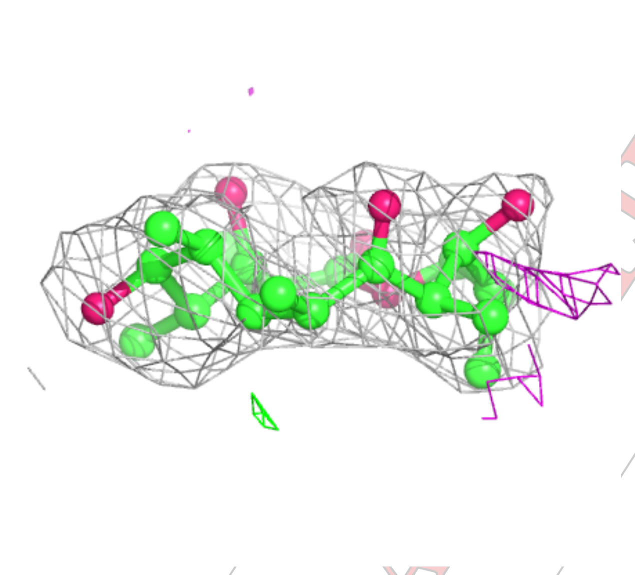

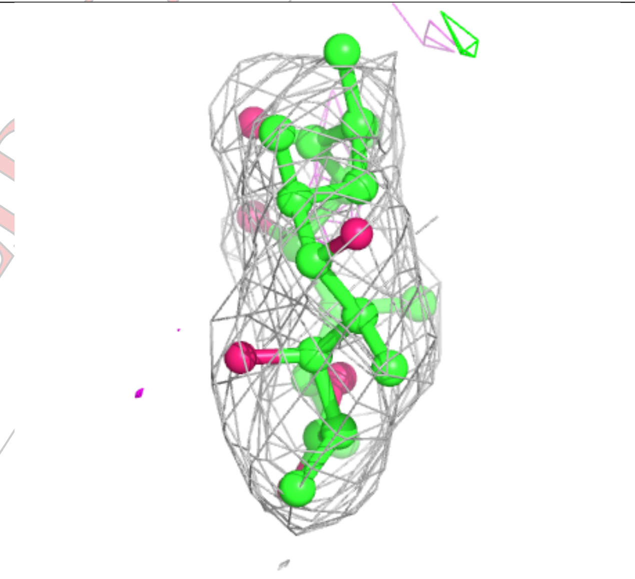

CONFIDENTIAL

Electron density around HEM H 501:

$2mF_o$-DF_c (at 0.7 rmsd) in gray
mF_o-DF_c (at 3 rmsd) in purple (negative)
and green (positive)

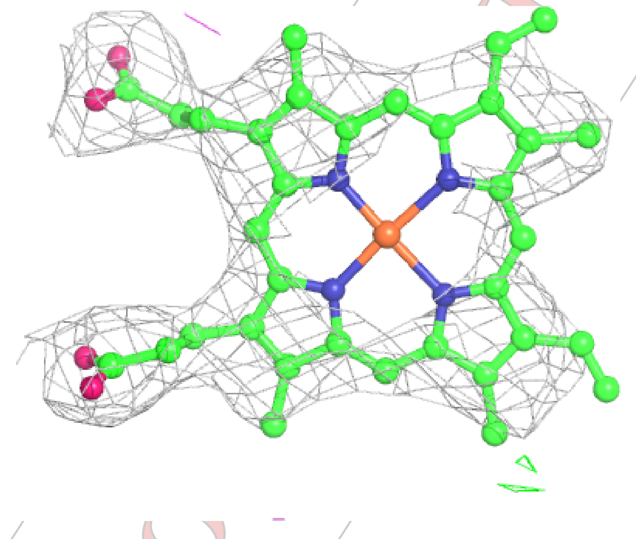

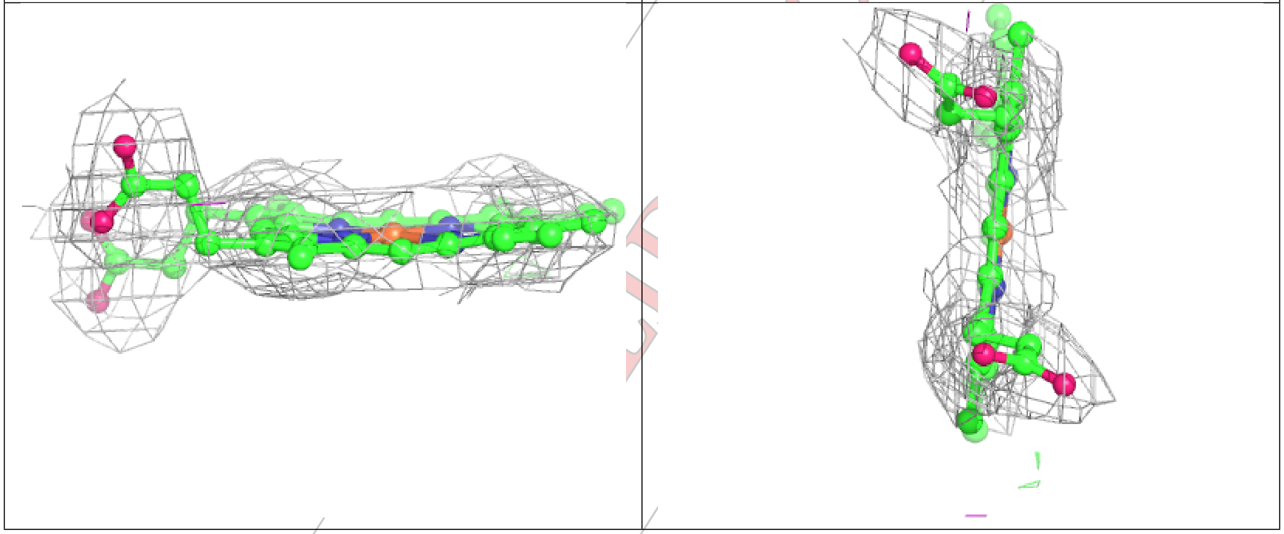

CONFIDENTIAL

Electron density around HEM B 501:

$2mF_o$-DF_c (at 0.7 rmsd) in gray
mF_o-DF_c (at 3 rmsd) in purple (negative)
and green (positive)

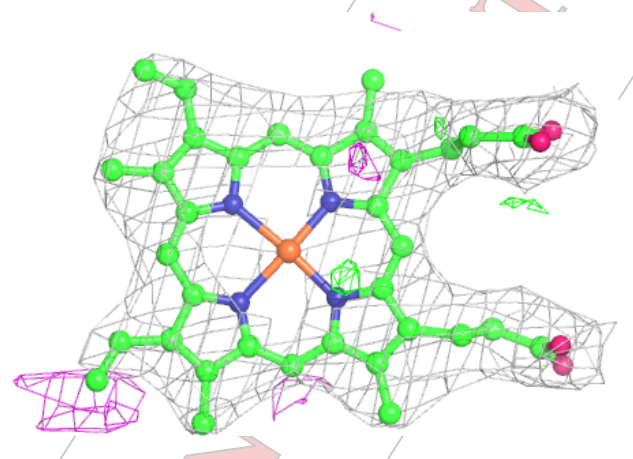

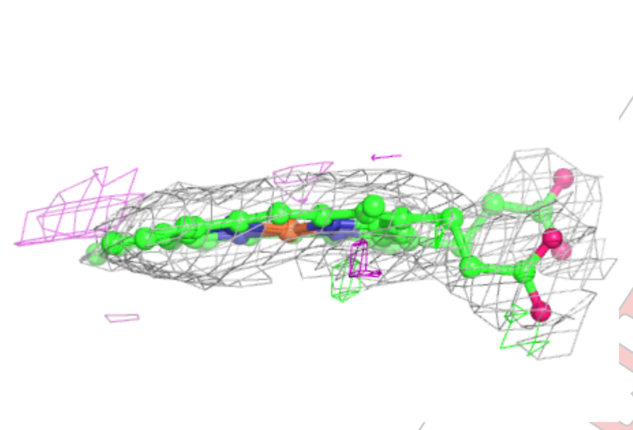

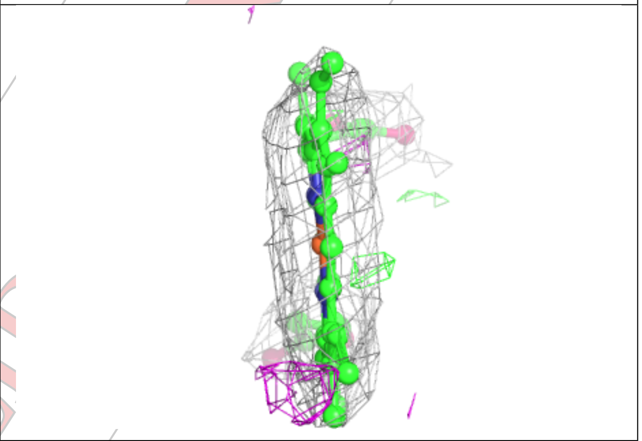

CONFIDENTIAL VAL

Electron density around QR8 C 502:

$2mF_o$-DF_c (at 0.7 rmsd) in gray
mF_o-DF_c (at 3 rmsd) in purple (negative)
and green (positive)

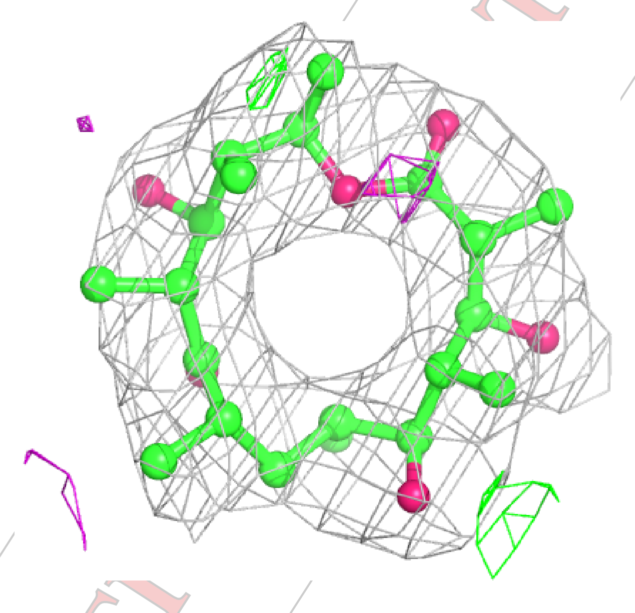

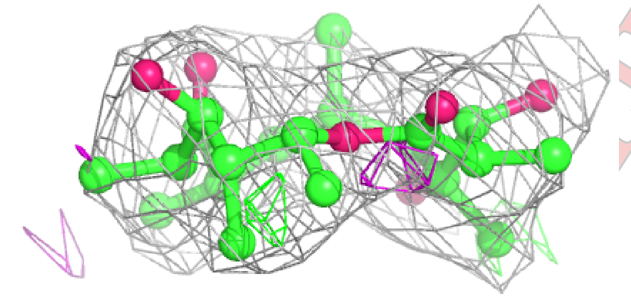

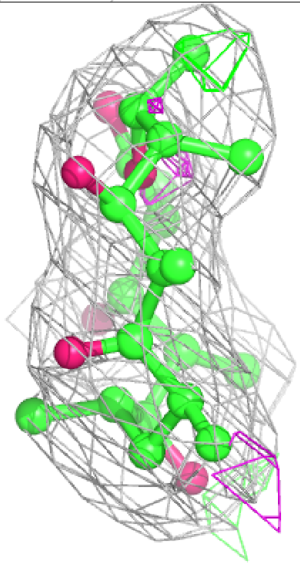

CONFIDENTIAL DRAFT

Electron density around HEM G 501:

2mF$_o$-DF$_c$ (at 0.7 rmsd) in gray
mF$_o$-DF$_c$ (at 3 rmsd) in purple (negative)
and green (positive)

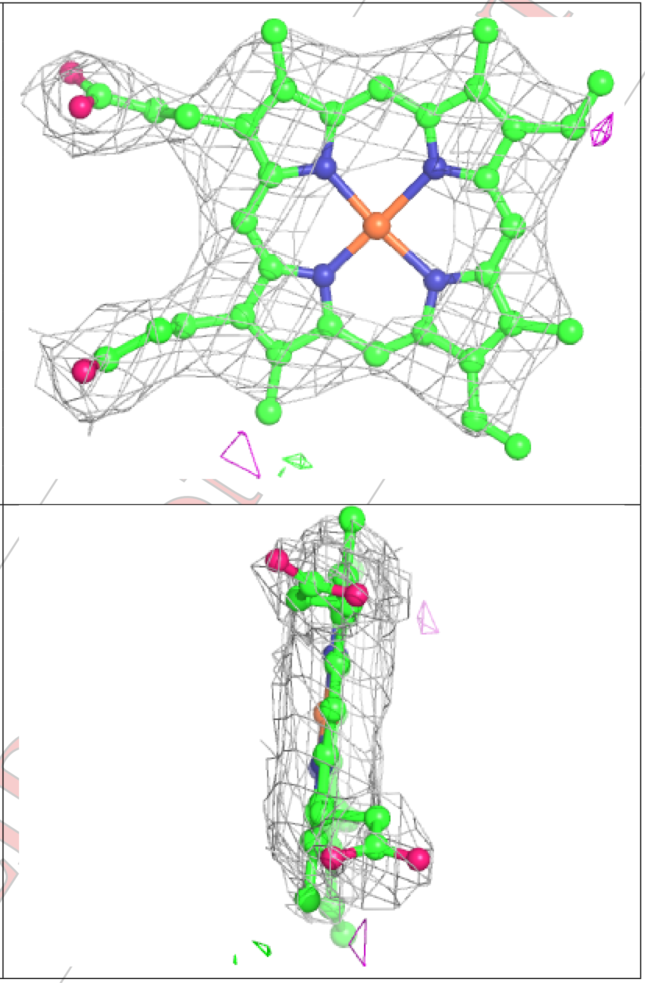

CONFIDENTIAL VALIDATION REPORT

Electron density around HEM E 501:

2mF$_o$-DF$_c$ (at 0.7 rmsd) in gray
mF$_o$-DF$_c$ (at 3 rmsd) in purple (negative)
and green (positive)

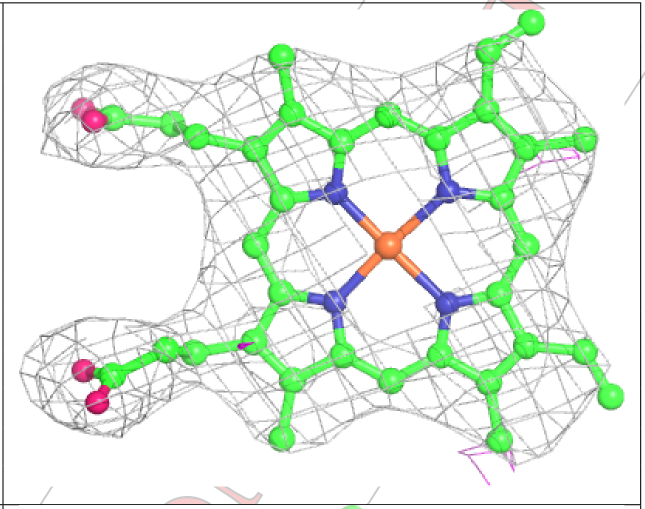

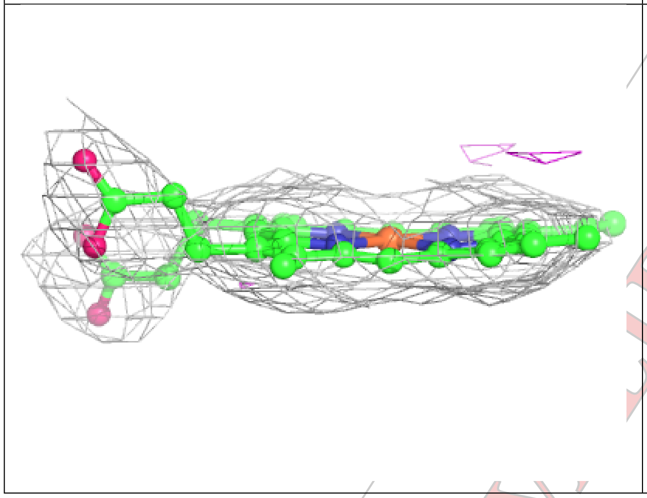

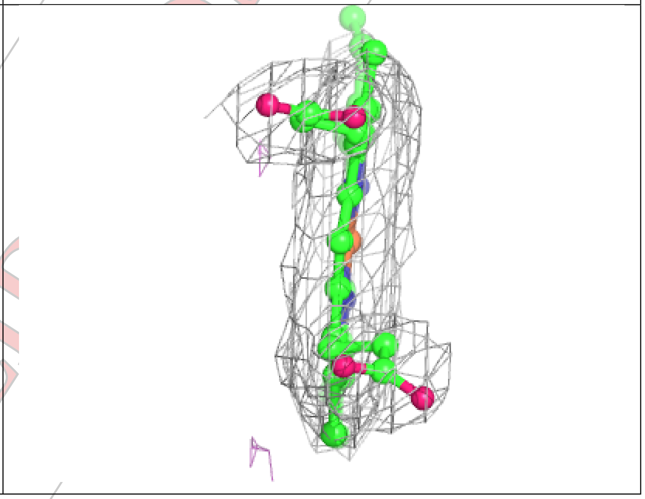

CONFIDENTIAL VALIDATION REPORT

Electron density around HEM D 501:

$2mF_o$-DF_c (at 0.7 rmsd) in gray
mF_o-DF_c (at 3 rmsd) in purple (negative)
and green (positive)

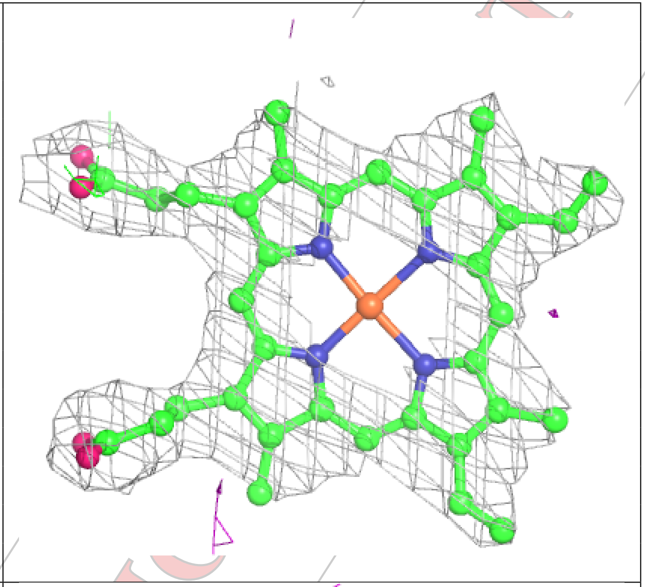

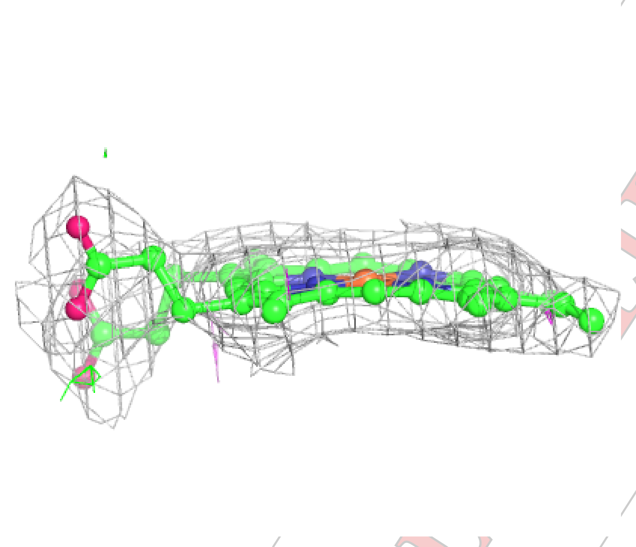

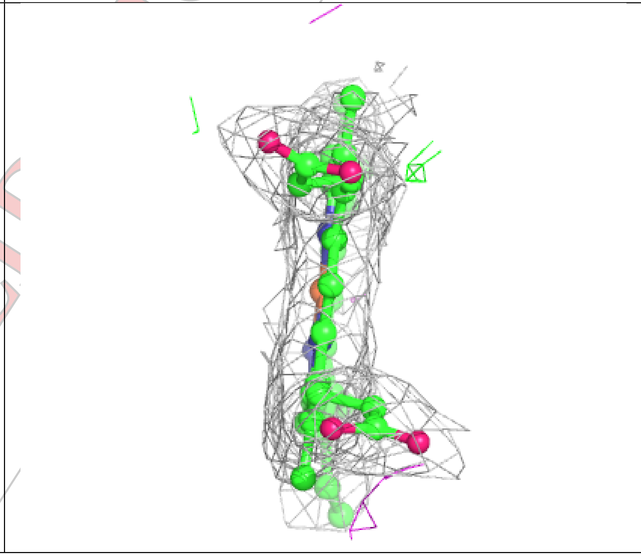

CONFIDENTIAL

Electron density around HEM C 501:

2mF$_o$-DF$_c$ (at 0.7 rmsd) in gray
mF$_o$-DF$_c$ (at 3 rmsd) in purple (negative)
and green (positive)

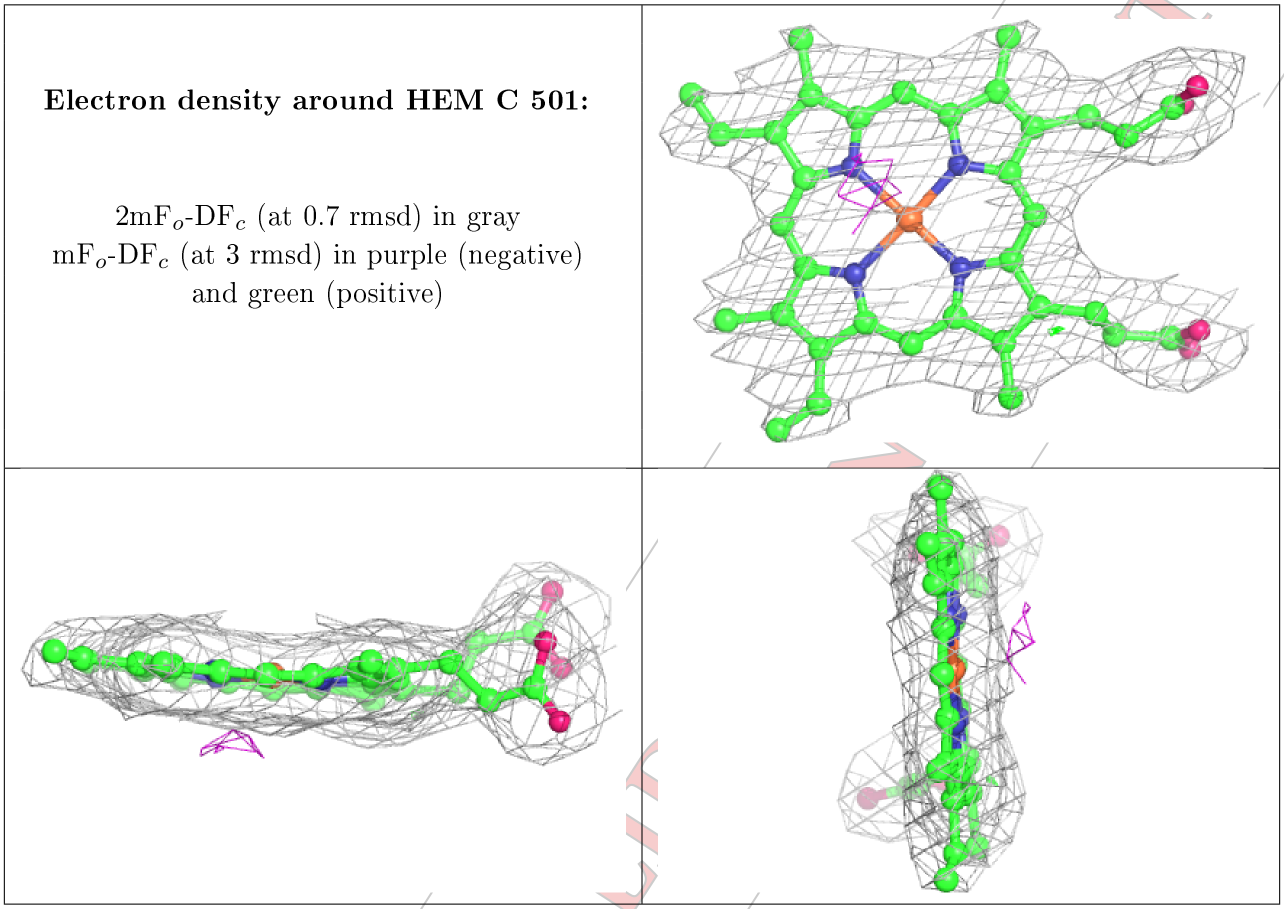

CONFIDENTIAL VALIDATION REPORT

Electron density around HEM A 501:

$2mF_o$-DF_c (at 0.7 rmsd) in gray
mF_o-DF_c (at 3 rmsd) in purple (negative)
and green (positive)

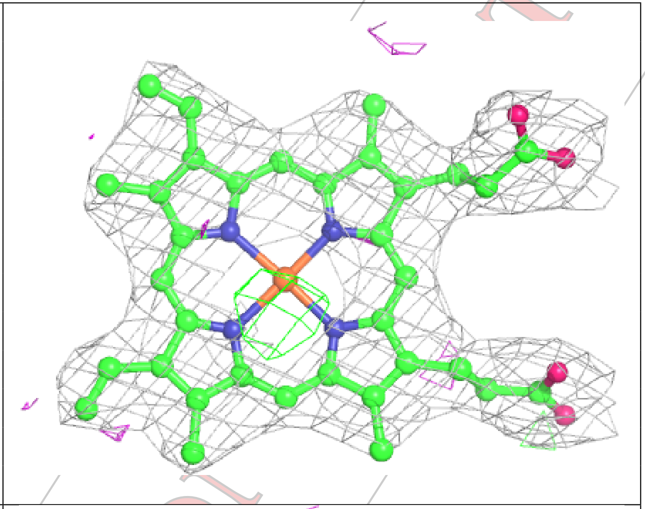

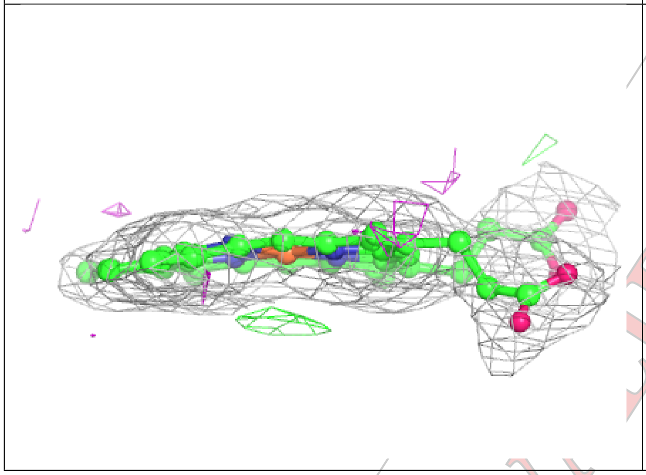

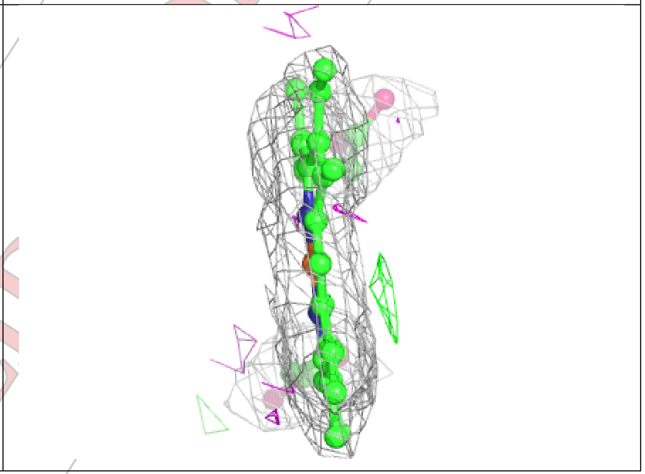

6.5 Other polymers ⓘ

There are no such residues in this entry.

CONFIDENTIAL VALIDATION REPORT
