# Supplementary material for: Dissecting the Cytochrome P450 OleP Substrate Specificity: Evidence for a Preferential Substrate
Source: Biomolecules. 2020 Oct 6;10(10):1411. doi: 10.3390/biom10101411 (PMC7600006; doi:10.3390/biom10101411)
Supplement: Supplementary file 1 [file biomolecules-10-01411-s001.zip › SupplMat_&_ValRep/6ZHZ_D_1292109583_val-report-full_P1.pdf]

Full wwPDB X-ray Structure Validation Report (i)

Jul 28, 2020 – 04:46 PM BST

PDB ID	:	6ZHZ
Title	:	OleP-oleandolide(DEO) in high salt crystallization conditions
Deposited on	:	2020-06-24
Resolution	:	2.20 Å(reported)

This is a Full wwPDB X-ray Structure Validation Report.

This report is produced by the wwPDB biocuration pipeline after annotation of the structure.

We welcome your comments at *validation@mail.wwpdb.org*
A user guide is available at
https://www.wwpdb.org/validation/2017/XrayValidationReportHelp
with specific help available everywhere you see the (i) symbol.

The following versions of software and data (see references (i)) were used in the production of this report:

MolProbity	:	4.02b-467
Mogul	:	1.8.5 (274361), CSD as541be (2020)
Xtriage (Phenix)	:	1.13
EDS	:	2.13
buster-report	:	1.1.7 (2018)
Percentile statistics	:	20191225.v01 (using entries in the PDB archive December 25th 2019)
Refmac	:	5.8.0158
CCP4	:	7.0.044 (Gargrove)
Ideal geometry (proteins)	:	Engh & Huber (2001)
Ideal geometry (DNA, RNA)	:	Parkinson et al. (1996)
Validation Pipeline (wwPDB-VP)	:	2.13

CONFIDENTIAL VALIDATION REPORT

1 Overall quality at a glance (i)

The following experimental techniques were used to determine the structure:
X-RAY DIFFRACTION

The reported resolution of this entry is 2.20 Å.

Percentile scores (ranging between 0-100) for global validation metrics of the entry are shown in the following graphic. The table shows the number of entries on which the scores are based.

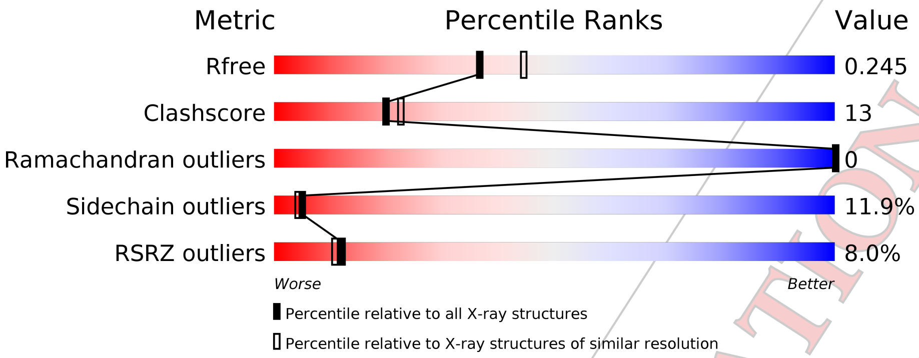

Metric	Whole archive (#Entries)	Similar resolution (#Entries, resolution range(Å))
R_{free}	130704	4898 (2.20-2.20)
Clashscore	141614	5594 (2.20-2.20)
Ramachandran outliers	138981	5503 (2.20-2.20)
Sidechain outliers	138945	5504 (2.20-2.20)
RSRZ outliers	127900	4800 (2.20-2.20)

The table below summarises the geometric issues observed across the polymeric chains and their fit to the electron density. The red, orange, yellow and green segments on the lower bar indicate the fraction of residues that contain outliers for >=3, 2, 1 and 0 types of geometric quality criteria respectively. A grey segment represents the fraction of residues that are not modelled. The numeric value for each fraction is indicated below the corresponding segment, with a dot representing fractions <=5% The upper red bar (where present) indicates the fraction of residues that have poor fit to the electron density. The numeric value is given above the bar.

Mol	Chain	Length	Quality of chain
1	A	407	5% / 70% 22% 6% •
1	B	407	4% / 72% 21% • •
1	C	407	3% / 75% 18% • •
1	D	407	7% / 63% 28% 6% •
1	E	407	10% / 70% 21% 6% •
1	F	407	18% / 67% 25% 5% •

CONFIDENTIAL VALIDATION REPORT

The following table lists non-polymeric compounds, carbohydrate monomers and non-standard residues in protein, DNA, RNA chains that are outliers for geometric or electron-density-fit criteria:

Mol	Type	Chain	Res	Chirality	Geometry	Clashes	Electron density
4	TRS	D	503	-	-	X	-
5	FMT	A	505	-	-	-	X
5	FMT	A	511	-	-	-	X
5	FMT	A	515	-	-	-	X
5	FMT	B	508	-	-	X	-
5	FMT	B	522	-	-	-	X
5	FMT	B	525	-	-	-	X
5	FMT	C	505	-	-	-	X
5	FMT	C	507	-	-	X	-
5	FMT	C	516	-	-	X	-
5	FMT	D	507	-	-	-	X
5	FMT	D	508	-	-	-	X
5	FMT	E	504	-	-	-	X
5	FMT	E	510	-	-	-	X
5	FMT	F	507	-	-	-	X

CONFIDENTIAL VALIDATION REPORT

2 Entry composition (i)

There are 8 unique types of molecules in this entry. The entry contains 20348 atoms, of which 0 are hydrogens and 0 are deuteriums.

In the tables below, the ZeroOcc column contains the number of atoms modelled with zero occupancy, the AltConf column contains the number of residues with at least one atom in alternate conformation and the Trace column contains the number of residues modelled with at most 2 atoms.

- Molecule 1 is a protein called Cytochrome P-450.

Mol	Chain	Residues	Atoms	ZeroOcc	AltConf	Trace
1	A	396	Total 3135, C 1978, N 561, O 583, S 13	0	7	0
1	B	397	Total 3148, C 1980, N 563, O 591, S 14	0	8	0
1	C	396	Total 3136, C 1976, N 563, O 584, S 13	0	7	0
1	D	395	Total 3082, C 1942, N 552, O 575, S 13	0	1	0
1	E	395	Total 3200, C 2006, N 580, O 601, S 13	0	13	0
1	F	395	Total 3102, C 1952, N 555, O 582, S 13	0	3	0

- Molecule 2 is PROTOPORPHYRIN IX CONTAINING FE (three-letter code: HEM) (formula: $C_{34}H_{32}FeN_4O_4$).

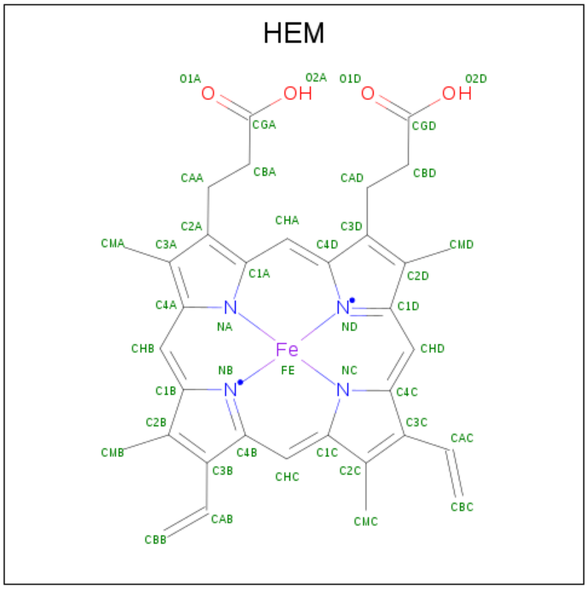

Mol	Chain	Residues	Atoms					ZeroOcc	AltConf
2	A	1	Total 43	C 34	Fe 1	N 4	O 4	0	0
2	B	1	Total 43	C 34	Fe 1	N 4	O 4	0	0
2	C	1	Total 43	C 34	Fe 1	N 4	O 4	0	0
2	D	1	Total 43	C 34	Fe 1	N 4	O 4	0	0
2	E	1	Total 43	C 34	Fe 1	N 4	O 4	0	0
2	F	1	Total 43	C 34	Fe 1	N 4	O 4	0	0

- Molecule 3 is (3 {R},4 {S},5 {R},6 {S},7 {S},9 {S},11 {R},12 {S},13 {R},14 {R})-3,5,7,9 ,11,13,14-heptamethyl-4,6,12-tris(oxidanyl)-1-oxacyclotetradecane-2,10-dione (three-letter code: QR8) (formula: $C_{20}H_{36}O_6$) (labeled as "Ligand of Interest" by author).

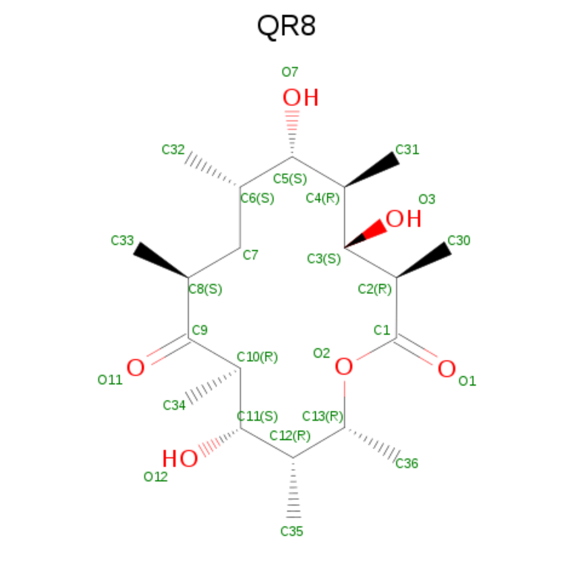

Mol	Chain	Residues	Atoms			ZeroOcc	AltConf
3	A	1	Total 26	C 20	O 6	0	0
3	B	1	Total 26	C 20	O 6	0	0
3	C	1	Total 26	C 20	O 6	0	0
3	D	1	Total 26	C 20	O 6	0	0
3	E	1	Total 26	C 20	O 6	0	0

Continued on next page...

CONFIDENTIAL - VALIDATION REPORT

Continued from previous page...

Mol	Chain	Residues	Atoms			ZeroOcc	AltConf
			Total	C	O		
3	F	1	26	20	6	0	0

- Molecule 4 is 2-AMINO-2-HYDROXYMETHYL-PROPANE-1,3-DIOL (three-letter code: TRS) (formula: $C_4H_{12}NO_3$).

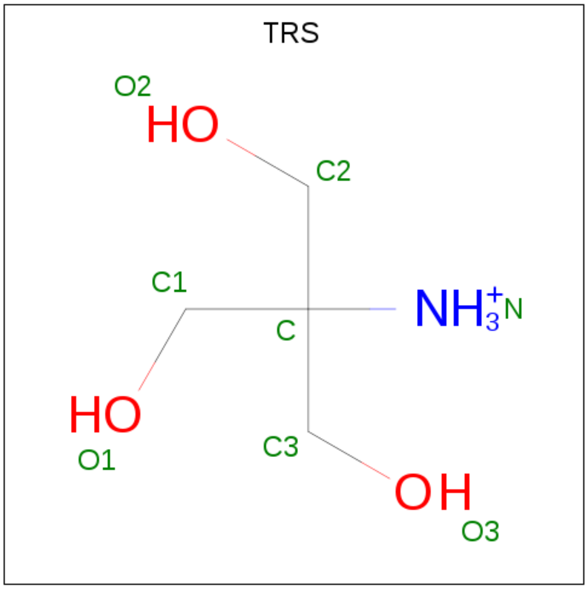

Mol	Chain	Residues	Atoms				ZeroOcc	AltConf
			Total	C	N	O		
4	A	1	8	4	1	3	0	0
4	D	1	8	4	1	3	0	0
4	F	1	8	4	1	3	0	0

- Molecule 5 is FORMIC ACID (three-letter code: FMT) (formula: CH_2O_2).

CONFIDENTIAL VALIDATION REPORT

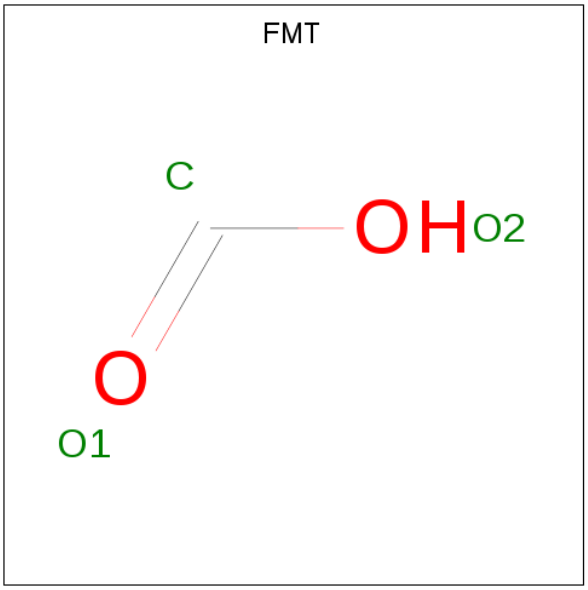

Mol	Chain	Residues	Atoms	ZeroOcc	AltConf
5	A	1	Total C O<br>3 1 2	0	0
5	A	1	Total C O<br>3 1 2	0	0
5	A	1	Total C O<br>3 1 2	0	0
5	A	1	Total C O<br>3 1 2	0	0
5	A	1	Total C O<br>3 1 2	0	0
5	A	1	Total C O<br>3 1 2	0	0
5	A	1	Total C O<br>3 1 2	0	0
5	A	1	Total C O<br>3 1 2	0	0
5	A	1	Total C O<br>3 1 2	0	0
5	A	1	Total C O<br>3 1 2	0	0
5	A	1	Total C O<br>3 1 2	0	0
5	A	1	Total C O<br>3 1 2	0	0
5	A	1	Total C O<br>3 1 2	0	0
5	A	1	Total C O<br>3 1 2	0	0

Continued on next page...

CONFIDENTIAL VALIDATION REPORT

Continued from previous page...

Mol	Chain	Residues	Atoms	ZeroOcc	AltConf
5	B	1	Total C O<br>3 1 2	0	0
5	B	1	Total C O<br>3 1 2	0	0
5	B	1	Total C O<br>3 1 2	0	0
5	B	1	Total C O<br>3 1 2	0	0
5	B	1	Total C O<br>3 1 2	0	0
5	B	1	Total C O<br>3 1 2	0	0
5	B	1	Total C O<br>3 1 2	0	0
5	B	1	Total C O<br>3 1 2	0	0
5	B	1	Total C O<br>3 1 2	0	0
5	B	1	Total C O<br>3 1 2	0	0
5	B	1	Total C O<br>3 1 2	0	0
5	B	1	Total C O<br>3 1 2	0	0
5	B	1	Total C O<br>3 1 2	0	0
5	B	1	Total C O<br>3 1 2	0	0
5	B	1	Total C O<br>3 1 2	0	0
5	B	1	Total C O<br>3 1 2	0	0
5	B	1	Total C O<br>3 1 2	0	0
5	B	1	Total C O<br>3 1 2	0	0
5	B	1	Total C O<br>3 1 2	0	0
5	B	1	Total C O<br>3 1 2	0	0
5	B	1	Total C O<br>3 1 2	0	0

Continued on next page...

CONFIDENTIAL VALIDATION REPORT

Continued from previous page...

Mol	Chain	Residues	Atoms	ZeroOcc	AltConf
5	B	1	Total C O<br>3 1 2	0	0
5	B	1	Total C O<br>3 1 2	0	0
5	B	1	Total C O<br>3 1 2	0	0
5	C	1	Total C O<br>3 1 2	0	0
5	C	1	Total C O<br>3 1 2	0	0
5	C	1	Total C O<br>3 1 2	0	0
5	C	1	Total C O<br>3 1 2	0	0
5	C	1	Total C O<br>3 1 2	0	0
5	C	1	Total C O<br>3 1 2	0	0
5	C	1	Total C O<br>3 1 2	0	0
5	C	1	Total C O<br>3 1 2	0	0
5	C	1	Total C O<br>3 1 2	0	0
5	C	1	Total C O<br>3 1 2	0	0
5	C	1	Total C O<br>3 1 2	0	0
5	C	1	Total C O<br>3 1 2	0	0
5	C	1	Total C O<br>3 1 2	0	0
5	C	1	Total C O<br>3 1 2	0	0
5	C	1	Total C O<br>3 1 2	0	0
5	C	1	Total C O<br>3 1 2	0	0
5	C	1	Total C O<br>3 1 2	0	0
5	C	1	Total C O<br>3 1 2	0	0

Continued on next page...

CONFIDENTIAL VALIDATION REPORT

Continued from previous page...

Mol	Chain	Residues	Atoms	ZeroOcc	AltConf
5	C	1	Total C O<br>3 1 2	0	0
5	C	1	Total C O<br>3 1 2	0	0
5	C	1	Total C O<br>3 1 2	0	0
5	D	1	Total C O<br>3 1 2	0	0
5	D	1	Total C O<br>3 1 2	0	0
5	D	1	Total C O<br>3 1 2	0	0
5	D	1	Total C O<br>3 1 2	0	0
5	D	1	Total C O<br>3 1 2	0	0
5	D	1	Total C O<br>3 1 2	0	0
5	D	1	Total C O<br>3 1 2	0	0
5	D	1	Total C O<br>3 1 2	0	0
5	E	1	Total C O<br>3 1 2	0	0
5	E	1	Total C O<br>3 1 2	0	0
5	E	1	Total C O<br>3 1 2	0	0
5	E	1	Total C O<br>3 1 2	0	0
5	E	1	Total C O<br>3 1 2	0	0
5	E	1	Total C O<br>3 1 2	0	0
5	E	1	Total C O<br>3 1 2	0	0
5	E	1	Total C O<br>3 1 2	0	0
5	F	1	Total C O<br>3 1 2	0	0
5	F	1	Total C O<br>3 1 2	0	0

Continued on next page...

CONFIDENTIAL VALIDATION REPORT

Continued from previous page...

Mol	Chain	Residues	Atoms	ZeroOcc	AltConf
5	F	1	Total C O<br>3 1 2	0	0
5	F	1	Total C O<br>3 1 2	0	0
5	F	1	Total C O<br>3 1 2	0	0
5	F	1	Total C O<br>3 1 2	0	0
5	F	1	Total C O<br>3 1 2	0	0
5	F	1	Total C O<br>3 1 2	0	0

- Molecule 6 is GLYCEROL (three-letter code: GOL) (formula: $C_3H_8O_3$).

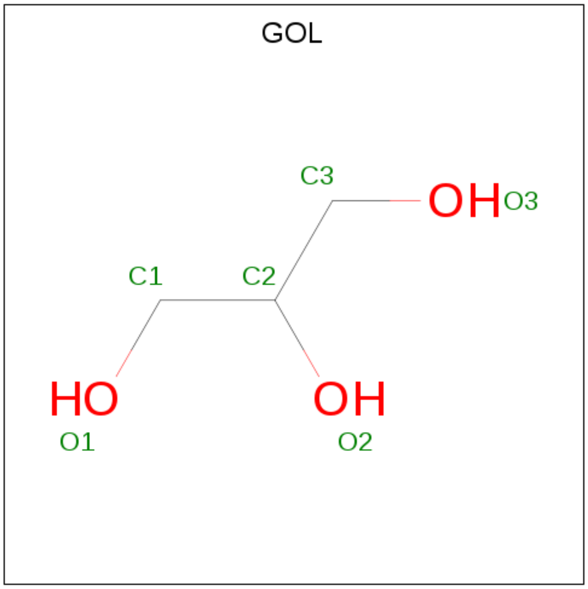

Mol	Chain	Residues	Atoms	ZeroOcc	AltConf
6	A	1	Total C O<br>6 3 3	0	0
6	B	1	Total C O<br>6 3 3	0	0
6	B	1	Total C O<br>6 3 3	0	0
6	B	1	Total C O<br>6 3 3	0	0
6	B	1	Total C O<br>6 3 3	0	0

Continued on next page...

CONFIDENTIAL VALIDATION REPORT

Continued from previous page...

Mol	Chain	Residues	Atoms	ZeroOcc	AltConf
6	B	1	Total C O<br>6 3 3	0	0
6	B	1	Total C O<br>6 3 3	0	0
6	C	1	Total C O<br>6 3 3	0	0
6	C	1	Total C O<br>6 3 3	0	0
6	C	1	Total C O<br>6 3 3	0	0
6	C	1	Total C O<br>6 3 3	0	0
6	C	1	Total C O<br>6 3 3	0	0
6	C	1	Total C O<br>6 3 3	0	0
6	D	1	Total C O<br>6 3 3	0	0
6	F	1	Total C O<br>6 3 3	0	0

- Molecule 7 is SODIUM ION (three-letter code: NA) (formula: Na).

Mol	Chain	Residues	Atoms	ZeroOcc	AltConf
7	B	1	Total Na<br>1 1	0	0
7	A	1	Total Na<br>1 1	0	0
7	D	1	Total Na<br>1 1	0	0
7	C	1	Total Na<br>1 1	0	0
7	F	1	Total Na<br>1 1	0	0

- Molecule 8 is water.

Mol	Chain	Residues	Atoms	ZeroOcc	AltConf
8	A	138	Total O<br>138 138	0	0
8	B	157	Total O<br>157 157	0	0

Continued on next page...

CONFIDENTIAL VALIDATION REPORT

Continued from previous page...

Mol	Chain	Residues	Atoms		ZeroOcc	AltConf
8	C	229	Total 229	O 229	0	0
8	D	84	Total 84	O 84	0	0
8	E	76	Total 76	O 76	0	0
8	F	79	Total 79	O 79	0	0

CONFIDENTIAL VALIDATION REPORT

3 Residue-property plots

These plots are drawn for all protein, RNA, DNA and oligosaccharide chains in the entry. The first graphic for a chain summarises the proportions of the various outlier classes displayed in the second graphic. The second graphic shows the sequence view annotated by issues in geometry and electron density. Residues are color-coded according to the number of geometric quality criteria for which they contain at least one outlier: green = 0, yellow = 1, orange = 2 and red = 3 or more. A red dot above a residue indicates a poor fit to the electron density (RSRZ > 2). Stretches of 2 or more consecutive residues without any outlier are shown as a green connector. Residues present in the sample, but not in the model, are shown in grey.

- Molecule 1: Cytochrome P-450

Chain A:

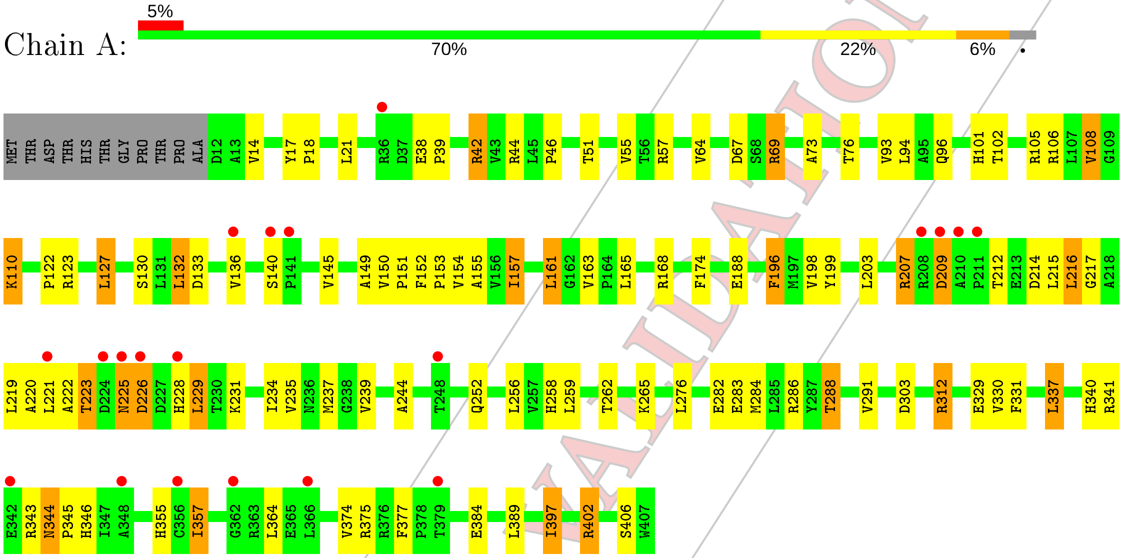

- Molecule 1: Cytochrome P-450

Chain B:

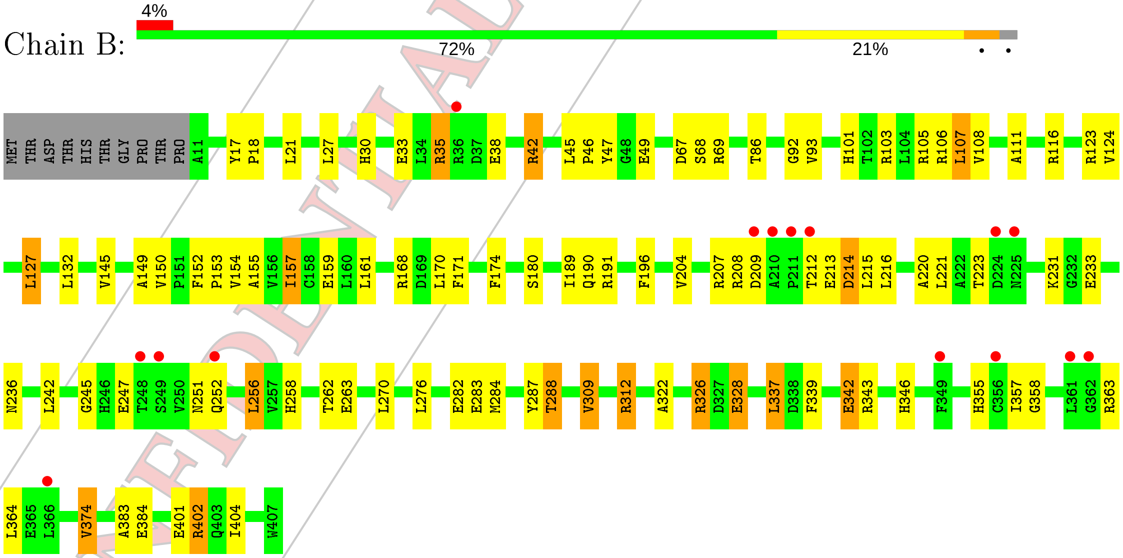

- Molecule 1: Cytochrome P-450

CONFIDENTIAL VALIDATION REPORT

Chain C:

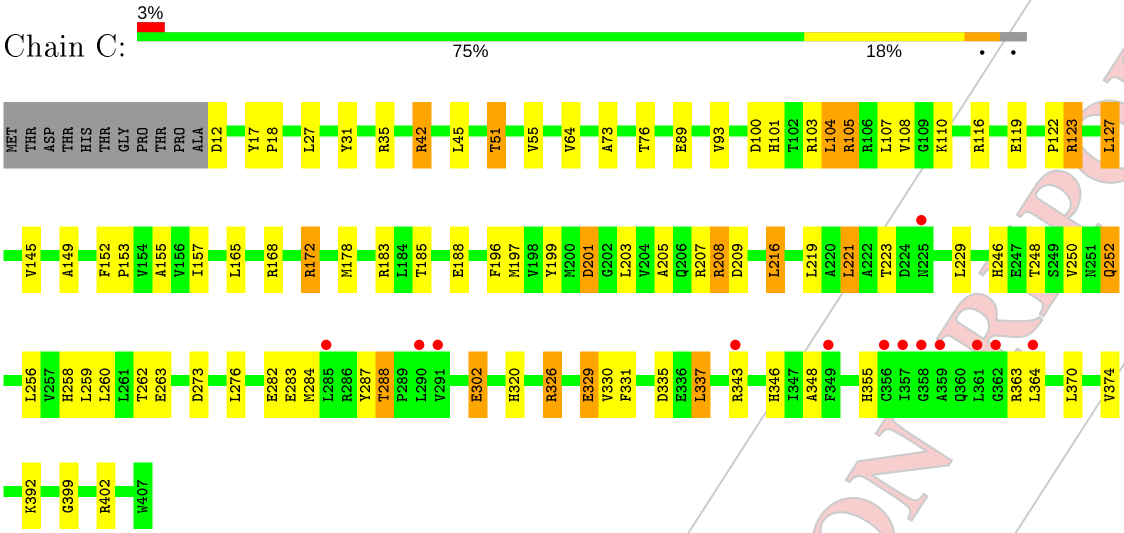

• Molecule 1: Cytochrome P-450

Chain D:

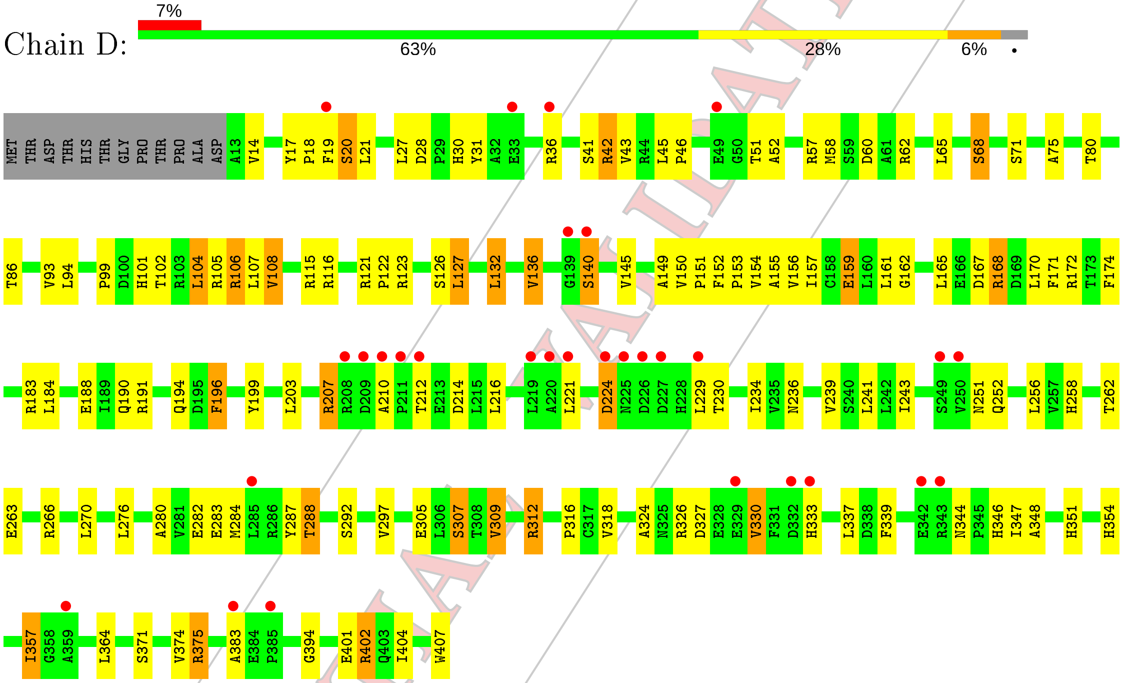

• Molecule 1: Cytochrome P-450

Chain E:

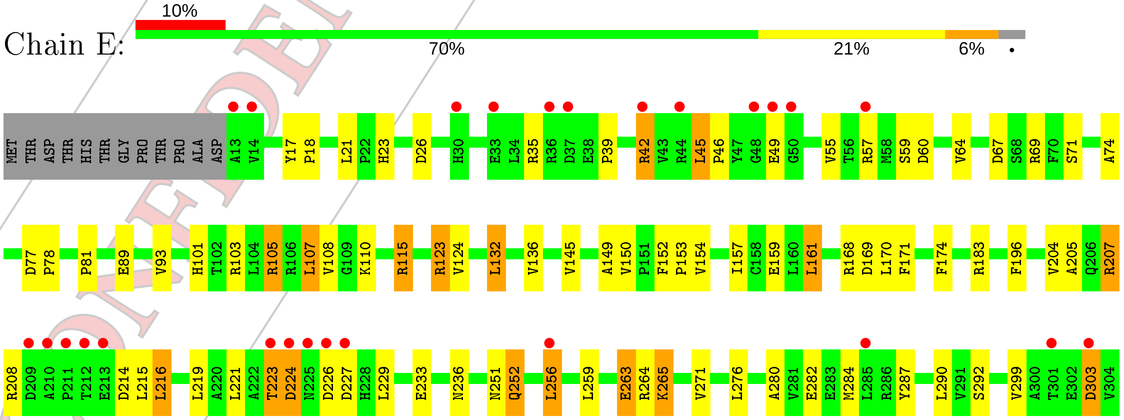

CONFIDENTIAL VALIDATION REPORT

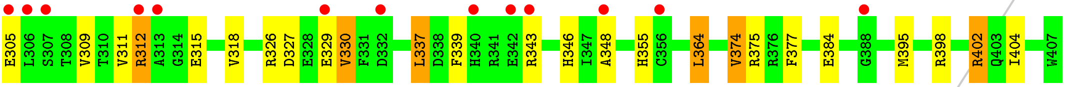

• Molecule 1: Cytochrome P-450

Chain F:

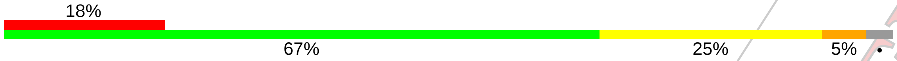

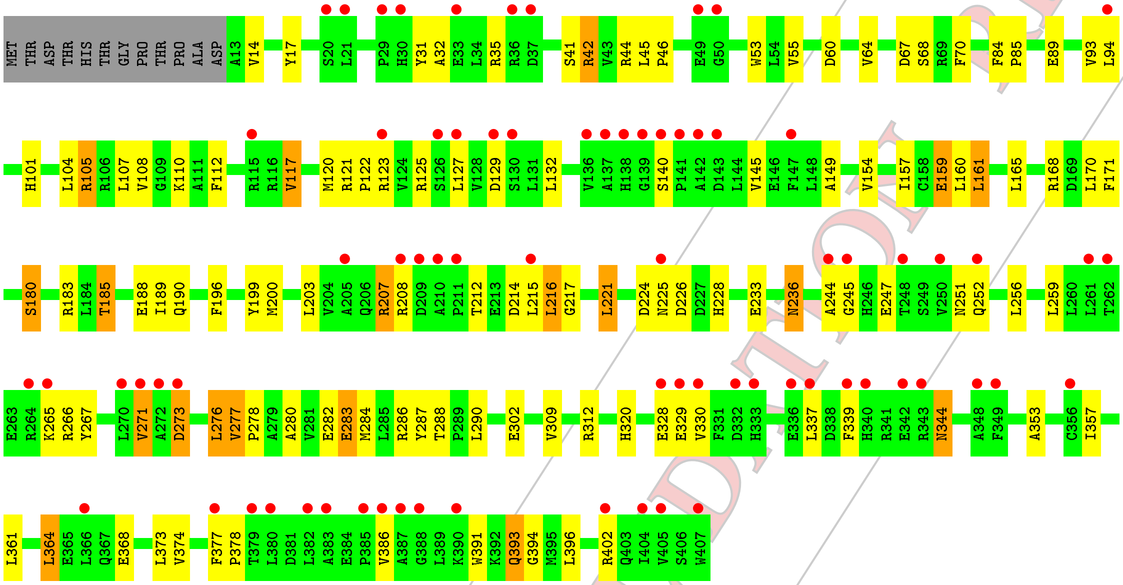

CONFIDENTIAL VALIDATION REPORT

4 Data and refinement statistics (i)

Property	Value	Source
Space group	C 1 2 1	Depositor
Cell constants<br>a, b, c, α, β, γ	247.47Å 111.22Å 159.20Å<br>90.00° 129.39° 90.00°	Depositor
Resolution (Å)	37.73 – 2.20<br>37.70 – 2.20	Depositor<br>EDS
% Data completeness<br>(in resolution range)	99.5 (37.73-2.20)<br>99.5 (37.70-2.20)	Depositor<br>EDS
R_{merge}	0.06	Depositor
R_{sym}	(Not available)	Depositor
$< I/\sigma(I) >$ [1]	1.39 (at 2.20Å)	Xtriage
Refinement program	REFMAC 5.8.0238	Depositor
R, R_{free}	0.187 , 0.243<br>0.192 , 0.245	Depositor<br>DCC
R_{free} test set	8476 reflections (5.04%)	wwPDB-VP
Wilson B-factor (Å^2)	44.9	Xtriage
Anisotropy	0.258	Xtriage
Bulk solvent k_{sol}(e/Å^3), B_{sol}(Å^2)	0.36 , 48.5	EDS
L-test for twinning[2]	$< \|L\| >$ = 0.48, $< L^2 >$ = 0.31	Xtriage
Estimated twinning fraction	0.022 for -h-2*l,-k,l	Xtriage
F_o,F_c correlation	0.97	EDS
Total number of atoms	20348	wwPDB-VP
Average B, all atoms (Å^2)	65.0	wwPDB-VP

Xtriage's analysis on translational NCS is as follows: *The analyses of the Patterson function reveals a significant off-origin peak that is 42.51 % of the origin peak, indicating pseudo-translational symmetry. The chance of finding a peak of this or larger height randomly in a structure without pseudo-translational symmetry is equal to 2.0173e-04. The detected translational NCS is most likely also responsible for the elevated intensity ratio.*

[1]Intensities estimated from amplitudes.

[2]Theoretical values of $< |L| >$, $< L^2 >$ for acentric reflections are 0.5, 0.333 respectively for untwinned datasets, and 0.375, 0.2 for perfectly twinned datasets.

CONFIDENTIAL VALIDATION REPORT

5 Model quality (i)

5.1 Standard geometry (i)

Bond lengths and bond angles in the following residue types are not validated in this section: GOL, NA, FMT, QR8, HEM, TRS

The Z score for a bond length (or angle) is the number of standard deviations the observed value is removed from the expected value. A bond length (or angle) with $|Z| > 5$ is considered an outlier worth inspection. RMSZ is the root-mean-square of all Z scores of the bond lengths (or angles).

Mol	Chain	Bond lengths		Bond angles	
		RMSZ	#|Z| >5	RMSZ	#|Z| >5
1	A	0.82	1/3214 (0.0%)	1.01	3/4376 (0.1%)
1	B	0.86	2/3227 (0.1%)	1.09	11/4395 (0.3%)
1	C	0.86	1/3218 (0.0%)	1.09	12/4380 (0.3%)
1	D	0.80	1/3152 (0.0%)	1.01	3/4295 (0.1%)
1	E	0.77	0/3270	0.95	1/4448 (0.0%)
1	F	0.84	2/3169 (0.1%)	0.97	1/4318 (0.0%)
All	All	0.83	7/19250 (0.0%)	1.02	31/26212 (0.1%)

All (7) bond length outliers are listed below:

Mol	Chain	Res	Type	Atoms	Z	Observed(Å)	Ideal(Å)
1	F	283	GLU	CD-OE2	15.47	1.42	1.25
1	B	49	GLU	CD-OE2	6.67	1.32	1.25
1	F	283	GLU	CD-OE1	6.48	1.32	1.25
1	A	188	GLU	CD-OE2	6.06	1.32	1.25
1	B	38	GLU	CD-OE1	5.62	1.31	1.25
1	C	178	MET	CG-SD	-5.47	1.67	1.81
1	D	188	GLU	CD-OE1	5.40	1.31	1.25

All (31) bond angle outliers are listed below:

Mol	Chain	Res	Type	Atoms	Z	Observed(^{o})	Ideal(^{o})
1	B	103	ARG	NE-CZ-NH1	-13.20	113.70	120.30
1	B	103	ARG	NE-CZ-NH2	12.35	126.48	120.30
1	C	105	ARG	NE-CZ-NH2	-8.11	116.24	120.30
1	C	35	ARG	NE-CZ-NH2	-7.32	116.64	120.30
1	B	363	ARG	NE-CZ-NH2	-7.13	116.74	120.30
1	B	35	ARG	NE-CZ-NH2	-6.67	116.97	120.30
1	C	326	ARG	NE-CZ-NH1	6.53	123.57	120.30
1	F	105	ARG	NE-CZ-NH2	-6.52	117.04	120.30

Continued on next page...

CONFIDENTIAL VALIDATION REPORT

Continued from previous page...

Mol	Chain	Res	Type	Atoms	Z	Observed(°)	Ideal(°)
1	C	326	ARG	CG-CD-NE	-6.52	98.12	111.80
1	B	103	ARG	CB-CG-CD	6.05	127.32	111.60
1	B	35	ARG	NE-CZ-NH1	6.01	123.30	120.30
1	C	326	ARG	NE-CZ-NH2	-6.01	117.30	120.30
1	B	363	ARG	NE-CZ-NH1	5.89	123.25	120.30
1	A	312	ARG	NE-CZ-NH1	5.83	123.21	120.30
1	E	105	ARG	CB-CG-CD	-5.82	96.46	111.60
1	D	375	ARG	NE-CZ-NH2	5.76	123.18	120.30
1	C	329	GLU	CB-CA-C	-5.68	99.04	110.40
1	C	35	ARG	NE-CZ-NH1	5.61	123.10	120.30
1	C	363	ARG	NE-CZ-NH1	5.59	123.09	120.30
1	B	326	ARG	NE-CZ-NH2	-5.57	117.52	120.30
1	C	105	ARG	NE-CZ-NH1	5.52	123.06	120.30
1	B	103	ARG	CD-NE-CZ	5.49	131.29	123.60
1	B	103	ARG	CG-CD-NE	-5.48	100.29	111.80
1	D	251	ASN	CB-CA-C	5.42	121.24	110.40
1	A	214	ASP	CB-CG-OD1	-5.31	113.52	118.30
1	C	35	ARG	CG-CD-NE	-5.30	100.67	111.80
1	C	178	MET	CA-CB-CG	-5.24	104.40	113.30
1	A	44	ARG	NE-CZ-NH2	5.23	122.91	120.30
1	D	168	ARG	NE-CZ-NH1	-5.10	117.75	120.30
1	B	326	ARG	NE-CZ-NH1	5.04	122.82	120.30
1	C	172	ARG	NE-CZ-NH1	5.02	122.81	120.30

There are no chirality outliers.

There are no planarity outliers.

5.2 Too-close contacts (i)

In the following table, the Non-H and H(model) columns list the number of non-hydrogen atoms and hydrogen atoms in the chain respectively. The H(added) column lists the number of hydrogen atoms added and optimized by MolProbity. The Clashes column lists the number of clashes within the asymmetric unit, whereas Symm-Clashes lists symmetry related clashes.

Mol	Chain	Non-H	H(model)	H(added)	Clashes	Symm-Clashes
1	A	3135	0	3135	75	0
1	B	3148	0	3127	77	0
1	C	3136	0	3133	65	2
1	D	3082	0	3066	100	0
1	E	3200	0	3162	79	2
1	F	3102	0	3074	83	0

Continued on next page...

CONFIDENTIAL VALIDATION REPORT

Continued from previous page...

Mol	Chain	Non-H	H(model)	H(added)	Clashes	Symm-Clashes
2	A	43	0	30	5	0
2	B	43	0	30	4	0
2	C	43	0	30	1	0
2	D	43	0	30	10	0
2	E	43	0	30	4	0
2	F	43	0	30	7	0
3	A	26	0	0	0	0
3	B	26	0	0	0	0
3	C	26	0	0	0	0
3	D	26	0	0	1	0
3	E	26	0	0	0	0
3	F	26	0	0	1	0
4	A	8	0	12	0	0
4	D	8	0	12	8	0
4	F	8	0	12	1	0
5	A	42	0	14	1	0
5	B	72	0	24	3	0
5	C	63	0	21	6	0
5	D	24	0	8	0	0
5	E	24	0	8	0	0
5	F	24	0	8	2	0
6	A	6	0	8	0	0
6	B	36	0	48	6	0
6	C	36	0	48	2	0
6	D	6	0	8	0	0
6	F	6	0	8	3	0
7	A	1	0	0	0	0
7	B	1	0	0	0	0
7	C	1	0	0	0	0
7	D	1	0	0	0	0
7	F	1	0	0	0	0
8	A	138	0	0	4	0
8	B	157	0	0	4	0
8	C	229	0	0	11	0
8	D	84	0	0	5	0
8	E	76	0	0	4	0
8	F	79	0	0	2	0
All	All	20348	0	19116	487	2

The all-atom clashscore is defined as the number of clashes found per 1000 atoms (including hydrogen atoms). The all-atom clashscore for this structure is 13.

All (487) close contacts within the same asymmetric unit are listed below, sorted by their clash magnitude.

CONFIDENTIAL VALIDATION REPORT

Atom-1	Atom-2	Interatomic distance (Å)	Clash overlap (Å)
1:C:207[A]:ARG:NH2	5:C:516:FMT:O2	1.84	1.10
1:E:375[B]:ARG:HG2	1:E:375[B]:ARG:HH21	0.86	1.02
1:E:226:ASP:OD1	1:E:229:LEU:N	1.93	1.01
1:E:375[B]:ARG:HH21	1:E:375[B]:ARG:CG	1.74	1.00
1:D:140:SER:O	1:D:407:TRP:CZ3	2.16	0.99
1:D:150:VAL:O	1:D:154:VAL:HG12	1.63	0.97
1:A:226:ASP:OD2	1:A:229:LEU:HD12	1.67	0.94
1:E:375[B]:ARG:HG2	1:E:375[B]:ARG:NH2	1.68	0.94
1:A:69:ARG:HG3	1:A:69:ARG:HH11	1.33	0.93
1:B:252[A]:GLN:HA	1:B:252[A]:GLN:OE1	1.67	0.91
1:C:284:MET:O	1:C:288:THR:HG23	1.70	0.91
1:D:284:MET:O	1:D:288:THR:HG23	1.74	0.88
1:B:209:ASP:HB2	1:F:302:GLU:OE2	1.73	0.88
1:A:226:ASP:OD2	1:A:229:LEU:CD1	2.23	0.87
1:E:105:ARG:NH2	1:E:355:HIS:O	2.08	0.86
1:F:267:TYR:OH	1:F:373:LEU:O	1.93	0.85
1:B:42:ARG:HD2	6:B:528:GOL:H11	1.58	0.85
1:A:357[A]:ILE:HD11	2:A:501:HEM:HMD2	1.58	0.83
1:A:284:MET:O	1:A:288:THR:HG23	1.78	0.82
1:A:105:ARG:NH2	1:A:355:HIS:O	2.11	0.81
1:B:342[A]:GLU:H	1:B:342[A]:GLU:CD	1.83	0.81
1:F:17:TYR:O	1:F:46:PRO:HD3	1.81	0.81
1:D:212:THR:OG1	8:D:601:HOH:O	1.99	0.81
1:B:326:ARG:HD3	8:B:658:HOH:O	1.82	0.80
1:A:259:LEU:HD11	1:A:288:THR:HG22	1.63	0.80
1:A:256:LEU:HD22	1:A:284:MET:HB3	1.65	0.79
1:D:60:ASP:OD2	1:D:307:SER:HB2	1.82	0.79
1:B:220:ALA:O	1:B:223:THR:OG1	2.00	0.79
1:F:89:GLU:OE1	5:F:511:FMT:O2	2.02	0.78
1:E:280:ALA:O	1:E:284:MET:HG3	1.84	0.78
1:D:101:HIS:HE1	2:D:501:HEM:O2D	1.67	0.77
1:B:150:VAL:O	1:B:154:VAL:HG13	1.84	0.77
1:B:207:ARG:NH2	1:B:214:ASP:OD2	2.18	0.76
1:F:159:GLU:OE1	1:F:159:GLU:HA	1.86	0.75
1:A:69:ARG:HG3	1:A:69:ARG:NH1	1.96	0.74
1:C:207[A]:ARG:HH22	5:C:516:FMT:C	2.01	0.74
1:D:159:GLU:HG3	4:D:503:TRS:H31	1.68	0.74
1:A:207:ARG:HD2	1:A:212:THR:OG1	1.86	0.74
1:C:100:ASP:OD2	6:C:527:GOL:H31	1.88	0.74
1:D:17:TYR:HH	1:D:31:TYR:HH	0.76	0.74
1:D:105:ARG:NE	1:D:357[B]:ILE:HD11	2.02	0.74
1:B:42:ARG:CD	6:B:528:GOL:H11	2.17	0.73

Continued on next page...

CONFIDENTIAL VALIDATION REPORT

Continued from previous page...

Atom-1	Atom-2	Interatomic distance (Å)	Clash overlap (Å)
1:D:383:ALA:HB3	1:D:404:ILE:HG22	1.72	0.72
1:F:101:HIS:HE1	2:F:501:HEM:O2D	1.72	0.72
1:D:256:LEU:HD22	1:D:284:MET:HB3	1.72	0.72
1:F:337[A]:LEU:HD21	1:F:339:PHE:CE1	2.25	0.72
1:F:233:GLU:OE2	6:F:512:GOL:H2	1.90	0.71
1:B:101:HIS:HD2	8:B:623:HOH:O	1.73	0.71
1:B:355:HIS:HE2	5:B:508:FMT:C	2.03	0.71
8:A:674:HOH:O	1:D:42:ARG:HG2	1.92	0.70
1:C:116[A]:ARG:HG3	1:C:116[A]:ARG:HH11	1.57	0.70
1:E:23:HIS:ND1	1:E:26:ASP:OD2	2.26	0.69
1:A:67:ASP:OD1	1:A:69:ARG:HG3	1.92	0.69
1:C:335:ASP:HB2	8:C:724:HOH:O	1.93	0.69
1:A:152:PHE:HB3	1:A:153:PRO:HD3	1.75	0.69
1:F:112:PHE:HB3	1:F:357:ILE:O	1.92	0.69
1:F:233:GLU:OE2	6:F:512:GOL:C2	2.41	0.68
1:D:140:SER:HA	1:D:407:TRP:CZ2	2.28	0.68
1:B:312:ARG:HD2	8:C:697:HOH:O	1.92	0.68
1:B:283:GLU:HG3	1:B:337:LEU:HD22	1.76	0.68
1:F:252:GLN:HE22	1:F:290:LEU:HB2	1.58	0.68
1:D:152:PHE:HB3	1:D:153:PRO:HD3	1.77	0.67
1:E:101:HIS:HE1	2:E:501:HEM:O2D	1.78	0.67
1:B:207:ARG:HG2	1:B:212:THR:HG21	1.77	0.67
1:F:276:LEU:HD11	1:F:339:PHE:HB3	1.77	0.67
1:E:67:ASP:OD1	1:E:69:ARG:HG3	1.96	0.66
1:E:42[A]:ARG:HH11	1:E:42[A]:ARG:HG2	1.60	0.66
1:F:145:VAL:HA	1:F:149:ALA:HB3	1.78	0.65
1:A:357[A]:ILE:HD11	2:A:501:HEM:CMD	2.27	0.65
1:E:233:GLU:OE2	8:E:602:HOH:O	2.14	0.65
1:E:216:LEU:HD12	1:E:219:LEU:HD12	1.78	0.65
1:D:20:SER:HB3	1:D:28:ASP:OD2	1.97	0.65
1:F:228:HIS:O	6:F:512:GOL:O1	2.13	0.65
1:A:122:PRO:HB3	1:D:309:VAL:HG12	1.79	0.65
1:D:167:ASP:OD2	1:D:199:TYR:OH	2.11	0.65
1:C:283:GLU:HG3	1:C:337:LEU:HD22	1.78	0.64
1:F:121:ARG:NH1	1:F:368:GLU:OE1	2.29	0.64
1:B:157:ILE:HD13	1:B:161:LEU:HG	1.78	0.64
1:D:58:MET:HE2	1:D:62:ARG:HG3	1.78	0.64
1:F:165:LEU:O	1:F:168:ARG:HG3	1.98	0.64
1:E:60:ASP:O	1:E:64:VAL:HG23	1.98	0.63
1:F:276:LEU:HD11	1:F:339:PHE:CB	2.27	0.63
1:E:271:VAL:HA	1:E:374:VAL:HG13	1.80	0.63

Continued on next page...

CONFIDENTIAL VALIDATION REPORT

Continued from previous page...

Atom-1	Atom-2	Interatomic distance (Å)	Clash overlap (Å)
1:E:263:GLU:HG2	1:E:265:LYS:HE2	1.81	0.63
1:A:222:ALA:O	1:A:226:ASP:HB2	1.98	0.63
1:F:108:VAL:CG2	1:F:215:LEU:HD13	2.29	0.63
1:D:284:MET:O	1:D:288:THR:CG2	2.46	0.63
1:B:256:LEU:HD22	1:B:284[B]:MET:HB3	1.80	0.62
1:E:150:VAL:O	1:E:154:VAL:HG13	1.98	0.62
1:D:282:GLU:OE1	1:D:346:HIS:HE1	1.82	0.62
1:B:309:VAL:HG13	1:C:122:PRO:HB3	1.81	0.62
1:F:286:ARG:HD3	1:F:344:ASN:HD21	1.63	0.62
1:A:133:ASP:O	1:A:136:VAL:HG22	1.99	0.62
1:B:108:VAL:HG22	1:B:215:LEU:HD22	1.81	0.62
1:F:108:VAL:HG23	1:F:215:LEU:HD22	1.82	0.62
1:E:145:VAL:HA	1:E:149:ALA:HB3	1.81	0.62
2:F:501:HEM:HMB2	2:F:501:HEM:HBB2	1.81	0.62
1:B:105:ARG:NH2	1:B:355:HIS:O	2.25	0.61
1:C:103:ARG:NE	8:C:601:HOH:O	1.89	0.61
1:C:259:LEU:HD11	1:C:288:THR:HG22	1.81	0.61
1:D:326:ARG:HD3	8:D:620:HOH:O	1.99	0.61
1:B:256:LEU:HD22	1:B:284[A]:MET:HB3	1.81	0.61
1:E:207:ARG:NH2	1:E:214:ASP:OD2	2.33	0.61
1:D:183:ARG:HD3	1:D:394:GLY:HA3	1.82	0.61
1:E:42[A]:ARG:NH1	1:E:42[A]:ARG:HG2	2.14	0.61
1:B:213:GLU:HG2	1:B:213:GLU:O	2.01	0.60
1:A:130:SER:O	1:A:133:ASP:HB2	2.01	0.60
1:B:355:HIS:NE2	5:B:508:FMT:O2	2.32	0.60
1:A:220:ALA:O	1:A:223:THR:HG22	2.00	0.60
1:D:207:ARG:HD2	8:D:601:HOH:O	2.02	0.60
1:C:205:ALA:O	1:C:208:ARG:HB2	2.02	0.60
1:D:239:VAL:O	1:D:243:ILE:HG13	2.02	0.59
2:E:501:HEM:HMC2	2:E:501:HEM:HBC2	1.84	0.59
1:A:258:HIS:O	1:A:262:THR:HG23	2.02	0.59
1:A:69:ARG:CG	1:A:69:ARG:HH11	2.10	0.59
2:B:501:HEM:HMC2	2:B:501:HEM:HBC2	1.84	0.58
1:A:286:ARG:HD3	1:A:344:ASN:HD21	1.67	0.58
1:F:168:ARG:HA	1:F:171:PHE:CZ	2.38	0.58
1:C:105:ARG:NH2	1:C:355:HIS:O	2.29	0.58
1:C:392:LYS:HG3	1:C:399:GLY:O	2.04	0.58
1:D:94:LEU:HD21	3:D:502:QR8:O12	2.03	0.58
1:E:282:GLU:OE1	1:E:346:HIS:HE1	1.87	0.58
1:F:283:GLU:HA	1:F:283:GLU:OE1	2.04	0.57
1:E:23:HIS:CE1	1:E:26:ASP:OD2	2.57	0.57

Continued on next page...

CONFIDENTIAL VALIDATION REPORT

Continued from previous page...

Atom-1	Atom-2	Interatomic distance (Å)	Clash overlap (Å)
1:A:67:ASP:OD1	1:A:69:ARG:CG	2.52	0.57
1:C:256:LEU:HD12	1:C:370[A]:LEU:HD11	1.85	0.57
1:E:55:VAL:HG13	1:E:60:ASP:HB3	1.86	0.57
1:B:152:PHE:HB3	1:B:153:PRO:HD3	1.86	0.57
1:C:256:LEU:HD22	1:C:284:MET:HB3	1.86	0.57
1:F:273:ASP:HB3	1:F:276:LEU:HB2	1.86	0.57
2:F:501:HEM:HMC2	2:F:501:HEM:HBC2	1.86	0.57
1:A:384:GLU:OE1	1:A:389:LEU:HD23	2.05	0.57
1:B:223:THR:O	1:B:231[B]:LYS:NZ	2.37	0.57
1:E:337:LEU:HD11	1:E:339:PHE:CE1	2.40	0.57
1:F:252:GLN:O	1:F:256:LEU:HG	2.05	0.56
1:A:207:ARG:CD	1:A:212:THR:OG1	2.54	0.56
2:B:501:HEM:HBC2	2:B:501:HEM:CMC	2.35	0.56
1:D:101:HIS:CE1	1:D:354:HIS:ND1	2.73	0.56
1:D:207:ARG:HA	1:D:210:ALA:O	2.06	0.56
1:F:259:LEU:HD11	1:F:288:THR:HG22	1.87	0.56
2:D:501:HEM:HBC2	2:D:501:HEM:HMC2	1.87	0.56
1:A:199:TYR:CZ	1:A:203:LEU:HD11	2.41	0.56
1:C:12:ASP:N	8:C:610:HOH:O	2.39	0.56
1:E:123:ARG:HD3	1:E:159[A]:GLU:OE1	2.05	0.56
1:B:35:ARG:NH2	8:B:604:HOH:O	2.35	0.56
1:D:159:GLU:CG	4:D:503:TRS:H31	2.34	0.56
1:E:42[A]:ARG:HH11	1:E:42[A]:ARG:CG	2.18	0.56
1:E:152:PHE:HB3	1:E:153:PRO:HD3	1.88	0.56
1:E:287:TYR:CG	1:E:337:LEU:HD23	2.40	0.56
1:D:357[A]:ILE:HD11	2:D:501:HEM:CMD	2.36	0.56
1:A:252:GLN:HA	1:A:252:GLN:OE1	2.05	0.56
1:C:55:VAL:HG21	1:C:64:VAL:HG21	1.87	0.56
1:D:157:ILE:O	1:D:161:LEU:HB2	2.07	0.55
1:C:329:GLU:HG2	8:C:784:HOH:O	2.06	0.55
1:D:371:SER:O	1:D:375:ARG:HG3	2.06	0.55
4:D:503:TRS:O2	4:D:503:TRS:O1	2.20	0.55
1:F:157:ILE:HG13	1:F:161:LEU:HD22	1.87	0.55
1:F:117:VAL:O	1:F:120:MET:HG2	2.06	0.55
1:E:168:ARG:HA	1:E:171:PHE:CE2	2.42	0.55
1:E:101:HIS:HD2	8:E:616:HOH:O	1.88	0.55
1:B:67:ASP:OD1	1:B:69:ARG:HB2	2.07	0.55
1:C:346:HIS:HD2	1:C:348:ALA:H	1.55	0.55
1:F:236:ASN:HD22	1:F:236:ASN:C	2.11	0.55
1:C:185:THR:OG1	1:C:188[B]:GLU:OE1	2.23	0.54
1:C:326:ARG:HD3	8:C:677:HOH:O	2.08	0.54

Continued on next page...

CONFIDENTIAL VALIDATION REPORT

Continued from previous page...

Atom-1	Atom-2	Interatomic distance (Å)	Clash overlap (Å)
1:D:157:ILE:HG13	1:D:161:LEU:HD22	1.89	0.54
1:F:207:ARG:HD2	1:F:212:THR:OG1	2.06	0.54
1:F:226:ASP:O	8:F:601:HOH:O	2.17	0.54
1:F:108:VAL:HG22	1:F:215:LEU:HD13	1.89	0.54
1:A:150:VAL:O	1:A:154:VAL:HG23	2.08	0.54
1:D:17:TYR:CD1	1:D:18:PRO:HA	2.43	0.53
1:D:159:GLU:HG3	4:D:503:TRS:C3	2.37	0.53
1:E:101:HIS:CE1	2:E:501:HEM:O2D	2.60	0.53
1:E:346:HIS:HD2	1:E:348:ALA:H	1.54	0.53
1:E:17:TYR:HA	1:E:18:PRO:C	2.28	0.53
1:E:157:ILE:HG13	1:E:161:LEU:HD22	1.90	0.53
1:E:170:LEU:HD22	1:E:174:PHE:CZ	2.44	0.53
1:C:260:LEU:HD11	1:C:370[B]:LEU:HD21	1.89	0.53
1:D:357[A]:ILE:HD11	2:D:501:HEM:HMD2	1.90	0.53
1:D:17:TYR:O	1:D:46:PRO:HD3	2.09	0.53
1:A:140:SER:OG	1:A:406:SER:HA	2.09	0.53
1:A:283:GLU:HG3	1:A:337:LEU:HD22	1.90	0.52
1:E:375[B]:ARG:CG	1:E:375[B]:ARG:NH2	2.47	0.52
1:D:105:ARG:CZ	1:D:357[B]:ILE:HG12	2.40	0.52
1:D:174:PHE:HB3	1:D:196:PHE:CD2	2.45	0.52
1:C:152:PHE:HB3	1:C:153:PRO:HD3	1.90	0.52
1:C:127:LEU:HD11	1:C:155:ALA:HB3	1.90	0.52
1:A:145:VAL:HG21	1:A:402:ARG:HA	1.92	0.52
1:D:140:SER:O	1:D:407:TRP:CH2	2.63	0.52
1:E:39:PRO:HB3	1:E:57[B]:ARG:HG3	1.92	0.52
1:F:101:HIS:CE1	2:F:501:HEM:O2D	2.59	0.52
1:C:330:VAL:HG22	1:C:331:PHE:CE2	2.45	0.51
1:D:145:VAL:HA	1:D:149:ALA:HB3	1.92	0.51
1:B:127:LEU:HD11	1:B:155:ALA:HB3	1.92	0.51
1:D:101:HIS:CE1	2:D:501:HEM:O2D	2.56	0.51
1:E:312[B]:ARG:N	1:E:315:GLU:OE2	2.31	0.51
1:C:172:ARG:HH11	5:C:507:FMT:C	2.23	0.51
1:E:183:ARG:NH2	8:E:604:HOH:O	2.39	0.51
1:F:185:THR:HG23	1:F:188:GLU:OE1	2.11	0.51
1:D:159:GLU:HB3	4:D:503:TRS:H11	1.92	0.51
1:B:342[A]:GLU:CD	1:B:342[A]:GLU:N	2.60	0.51
1:A:330:VAL:HG22	1:A:331:PHE:CD2	2.46	0.51
1:B:30:HIS:HD2	1:B:33:GLU:OE2	1.94	0.51
1:D:199:TYR:CZ	1:D:203:LEU:HD11	2.46	0.51
1:D:145:VAL:HG21	1:D:402:ARG:HA	1.93	0.51
1:D:224:ASP:OD1	1:D:224:ASP:N	2.43	0.51

Continued on next page...

CONFIDENTIAL VALIDATION REPORT

Continued from previous page...

Atom-1	Atom-2	Interatomic distance (Å)	Clash overlap (Å)
1:F:337[A]:LEU:HD23	1:F:337[A]:LEU:C	2.31	0.51
1:F:183:ARG:HB2	1:F:394:GLY:HA3	1.93	0.51
1:A:384:GLU:OE2	1:A:402:ARG:HD2	2.11	0.51
1:F:266:ARG:NH1	1:F:337[B]:LEU:CD1	2.73	0.51
1:A:17:TYR:HA	1:A:18:PRO:C	2.31	0.50
1:B:145:VAL:HG21	1:B:402:ARG:HA	1.93	0.50
1:D:123:ARG:CZ	4:D:503:TRS:H22	2.42	0.50
1:A:259:LEU:HD11	1:A:288:THR:CG2	2.37	0.50
1:C:116[A]:ARG:HG3	1:C:116[A]:ARG:NH1	2.25	0.50
2:E:501:HEM:CMC	2:E:501:HEM:HBC2	2.41	0.50
1:E:183:ARG:NH1	1:E:395:MET:SD	2.85	0.50
1:D:19:PHE:CE2	1:D:30:HIS:HD2	2.30	0.50
1:F:280:ALA:HA	1:F:339:PHE:CD1	2.46	0.50
1:B:157:ILE:HG13	1:B:245:GLY:HA3	1.93	0.50
1:E:17:TYR:O	1:E:46:PRO:HD3	2.12	0.50
1:F:105:ARG:HD2	1:F:357:ILE:HD12	1.94	0.50
1:A:17:TYR:O	1:A:46:PRO:HD3	2.11	0.50
1:E:208:ARG:HD2	1:E:223:THR:CG2	2.42	0.50
1:B:101:HIS:HE1	2:B:501:HEM:O2D	1.95	0.50
1:B:309:VAL:HG13	1:C:122:PRO:CB	2.42	0.50
1:C:172:ARG:HD2	5:C:507:FMT:O1	2.12	0.50
1:F:391:TRP:O	1:F:393:GLN:HG2	2.12	0.50
1:A:127:LEU:HD11	1:A:155:ALA:HB3	1.93	0.49
1:B:207:ARG:HG2	1:B:212:THR:CG2	2.42	0.49
1:D:52:ALA:HA	1:D:316:PRO:HG2	1.93	0.49
1:E:252:GLN:HE22	1:E:290:LEU:HB2	1.77	0.49
1:B:105:ARG:HD3	1:B:357:ILE:HD12	1.93	0.49
1:F:236:ASN:ND2	1:F:236:ASN:O	2.43	0.49
1:A:331:PHE:CE1	1:A:345:PRO:HD2	2.48	0.49
1:A:55:VAL:HG21	1:A:64:VAL:HG21	1.94	0.49
1:D:159:GLU:CB	4:D:503:TRS:H31	2.42	0.49
1:F:14:VAL:HG11	1:F:42:ARG:HB3	1.94	0.49
1:F:252:GLN:NE2	1:F:290:LEU:HB2	2.25	0.49
1:D:104:LEU:HD22	1:D:229:LEU:HD23	1.94	0.49
1:A:207:ARG:HD3	1:A:212:THR:HG23	1.93	0.48
1:D:152:PHE:CB	1:D:153:PRO:HD3	2.41	0.48
1:A:209:ASP:N	1:A:209:ASP:OD1	2.45	0.48
1:D:333:HIS:CG	8:D:635:HOH:O	2.66	0.48
1:B:231[B]:LYS:HD2	8:B:687:HOH:O	2.12	0.48
1:E:259:LEU:HB2	1:E:284:MET:HE2	1.95	0.48
1:D:68:SER:O	1:D:68:SER:OG	2.30	0.48

Continued on next page...

CONFIDENTIAL VALIDATION REPORT

Continued from previous page...

Atom-1	Atom-2	Interatomic distance (Å)	Clash overlap (Å)
1:A:207:ARG:HD3	1:A:212:THR:CG2	2.44	0.48
1:B:383:ALA:HB3	1:B:404:ILE:HG22	1.95	0.48
1:F:32:ALA:HA	1:F:35:ARG:NH1	2.28	0.48
1:D:132:LEU:O	1:D:136:VAL:CG1	2.62	0.48
1:D:140:SER:HA	1:D:407:TRP:CH2	2.48	0.48
1:E:221:LEU:O	1:E:224:ASP:HB2	2.13	0.48
1:F:217:GLY:O	1:F:221:LEU:HD22	2.14	0.48
2:D:501:HEM:HBC2	2:D:501:HEM:CMC	2.42	0.48
1:B:68:SER:HA	6:B:529:GOL:O1	2.14	0.48
1:C:103:ARG:NH2	8:C:601:HOH:O	2.45	0.48
1:E:280:ALA:HA	1:E:339:PHE:CD1	2.49	0.48
1:E:208:ARG:NE	1:E:223:THR:HG22	2.29	0.48
1:A:73:ALA:O	1:A:76:THR:HG23	2.14	0.48
1:C:221:LEU:HA	1:C:221:LEU:HD12	1.79	0.48
1:F:180:SER:OG	1:F:189:ILE:HD11	2.13	0.48
1:F:256:LEU:HD22	1:F:284:MET:HB3	1.95	0.47
1:A:145:VAL:HA	1:A:149:ALA:HB3	1.97	0.47
1:C:42:ARG:CZ	1:C:51:THR:CG2	2.92	0.47
1:F:271:VAL:HG21	1:F:378:PRO:HB3	1.96	0.47
1:E:124:VAL:HG21	1:E:364:LEU:HD13	1.96	0.47
1:C:273:ASP:O	1:C:276:LEU:HB2	2.15	0.47
1:C:42:ARG:NE	1:C:51:THR:HG23	2.30	0.47
1:B:168:ARG:HA	1:B:171:PHE:CZ	2.49	0.47
1:D:162:GLY:HA3	1:D:214:ASP:OD2	2.15	0.47
1:A:101:HIS:HD2	8:A:624:HOH:O	1.97	0.47
1:A:276:LEU:HD11	1:A:340:HIS:CE1	2.50	0.47
1:B:17:TYR:HA	1:B:18:PRO:C	2.34	0.47
1:B:157:ILE:HD12	1:B:242:LEU:HA	1.96	0.47
1:E:259:LEU:HB2	1:E:284:MET:CE	2.44	0.47
1:E:251:ASN:HB3	1:E:398:ARG:O	2.15	0.47
1:D:230:THR:O	1:D:234:ILE:HD12	2.14	0.47
1:D:241:LEU:HD21	1:D:357[A]:ILE:HD12	1.97	0.47
1:E:263:GLU:CG	1:E:265:LYS:HE2	2.43	0.47
1:B:123:ARG:HG3	6:B:527:GOL:H11	1.97	0.47
1:D:258:HIS:NE2	1:D:262:THR:HG21	2.29	0.47
1:A:102:THR:O	1:A:106:ARG:HG2	2.15	0.47
1:D:105:ARG:HG2	1:D:357[B]:ILE:HD11	1.95	0.47
2:F:501:HEM:CMB	2:F:501:HEM:HBB2	2.45	0.47
1:F:247:GLU:O	1:F:251:ASN:ND2	2.47	0.47
1:C:101:HIS:HD2	8:C:623:HOH:O	1.97	0.47
1:A:96:GLN:HE22	5:A:512:FMT:C	2.28	0.46

Continued on next page...

CONFIDENTIAL VALIDATION REPORT

Continued from previous page...

Atom-1	Atom-2	Interatomic distance (Å)	Clash overlap (Å)
1:F:121:ARG:N	1:F:122:PRO:HD2	2.29	0.46
1:F:353:ALA:HB3	4:F:503:TRS:N	2.30	0.46
1:B:284[A]:MET:HE2	1:B:337:LEU:HD11	1.96	0.46
1:C:283:GLU:HG3	1:C:337:LEU:CD2	2.44	0.46
1:F:101:HIS:HD2	5:F:506:FMT:O2	1.97	0.46
1:A:283:GLU:HA	1:A:283:GLU:OE1	2.15	0.46
1:B:322:ALA:O	1:B:326:ARG:HG2	2.15	0.46
1:C:101:HIS:HE1	2:C:501:HEM:O2D	1.99	0.46
1:C:346:HIS:CD2	1:C:348:ALA:H	2.33	0.46
1:C:104:LEU:HD13	1:C:229:LEU:HD13	1.96	0.46
1:F:364:LEU:O	1:F:364:LEU:HD22	2.16	0.46
1:F:368:GLU:OE2	1:F:368:GLU:HA	2.15	0.46
1:F:85:PRO:HD2	8:F:616:HOH:O	2.15	0.46
1:E:152:PHE:CB	1:E:153:PRO:HD3	2.46	0.46
1:D:346:HIS:HD2	1:D:348:ALA:H	1.64	0.46
1:A:207:ARG:HG2	1:A:217:GLY:HA2	1.96	0.46
1:A:282:GLU:OE1	1:A:346:HIS:HE1	1.98	0.46
1:D:42:ARG:HG3	1:D:51:THR:OG1	2.17	0.46
1:E:346:HIS:CD2	1:E:348:ALA:H	2.33	0.46
1:D:258:HIS:CE1	1:D:262:THR:HG21	2.51	0.45
1:E:303:ASP:HA	1:E:311:VAL:O	2.16	0.45
1:C:165:LEU:HD23	1:C:165:LEU:HA	1.77	0.45
1:E:292:SER:HA	1:E:398:ARG:HE	1.80	0.45
1:F:200:MET:SD	1:F:216:LEU:HD11	2.56	0.45
1:C:246:HIS:O	1:C:250:VAL:HG23	2.17	0.45
1:D:106:ARG:HG2	1:D:106:ARG:HH11	1.82	0.45
1:D:127:LEU:HD13	1:D:152:PHE:HD1	1.82	0.45
1:A:39:PRO:HG3	1:A:57:ARG:NH2	2.31	0.45
1:D:14:VAL:HG12	1:D:43:VAL:HA	1.98	0.45
1:A:291:VAL:HA	1:A:397[A]:ILE:HD12	1.98	0.45
2:A:501:HEM:HMC2	2:A:501:HEM:HBC2	1.98	0.45
1:D:105:ARG:NE	1:D:357[B]:ILE:CD1	2.76	0.45
1:E:208:ARG:HD2	1:E:223:THR:HG22	1.98	0.45
1:E:132:LEU:HA	1:E:132:LEU:HD12	1.86	0.45
1:E:55:VAL:HG13	1:E:60:ASP:CB	2.46	0.45
1:B:282:GLU:OE1	1:B:346:HIS:HE1	1.99	0.45
1:B:159:GLU:HB3	6:B:527:GOL:H2	1.99	0.45
1:C:282:GLU:OE1	1:C:346:HIS:HE1	2.00	0.45
1:C:355:HIS:ND1	5:C:511:FMT:C	2.80	0.45
1:F:280:ALA:O	1:F:284:MET:HG3	2.17	0.45
1:A:152:PHE:CB	1:A:153:PRO:HD3	2.44	0.45

Continued on next page...

CONFIDENTIAL VALIDATION REPORT

Continued from previous page...

Atom-1	Atom-2	Interatomic distance (Å)	Clash overlap (Å)
1:C:31:TYR:CZ	1:C:320:HIS:CD2	3.05	0.44
2:D:501:HEM:HHC	2:D:501:HEM:HAB	1.74	0.44
1:A:303:ASP:OD2	8:A:601:HOH:O	2.20	0.44
1:C:89:GLU:O	5:C:522:FMT:O2	2.35	0.44
1:D:346:HIS:CD2	1:D:348:ALA:H	2.35	0.44
1:E:337:LEU:CD1	1:E:339:PHE:CE1	3.00	0.44
1:A:161:LEU:HB3	1:A:163:VAL:HG23	1.98	0.44
1:B:252[A]:GLN:OE1	1:B:252[A]:GLN:CA	2.47	0.44
1:D:104:LEU:O	1:D:107:LEU:HB2	2.17	0.44
1:D:283:GLU:HA	1:D:344:ASN:HD21	1.81	0.44
1:E:256:LEU:HD22	1:E:284:MET:CB	2.47	0.44
1:F:170:LEU:HD23	1:F:170:LEU:C	2.37	0.44
1:A:110[A]:LYS:HG3	1:A:110[A]:LYS:H	1.32	0.44
1:D:127:LEU:HD11	1:D:155:ALA:HB3	1.99	0.44
1:D:256:LEU:HD22	1:D:284:MET:CB	2.44	0.44
1:A:397[A]:ILE:HD12	1:A:397[A]:ILE:HA	1.80	0.44
1:B:215:LEU:HD23	1:B:215:LEU:HA	1.72	0.44
1:C:127:LEU:HD13	1:C:152:PHE:HD1	1.81	0.44
1:F:221:LEU:O	1:F:224:ASP:HB2	2.18	0.44
1:F:287:TYR:CD1	1:F:337[B]:LEU:HG	2.52	0.44
1:C:248:THR:O	1:C:252:GLN:HB2	2.18	0.44
1:E:207:ARG:HG2	1:E:207:ARG:H	1.58	0.44
1:F:199:TYR:CZ	1:F:203:LEU:HD11	2.53	0.44
1:A:127:LEU:HD13	1:A:152:PHE:HD1	1.82	0.44
1:A:244:ALA:HB1	2:A:501:HEM:C4C	2.53	0.44
1:B:111:ALA:HA	1:B:116[B]:ARG:HG2	1.99	0.44
1:B:46:PRO:HB2	1:B:47:TYR:CE1	2.53	0.44
1:D:151:PRO:HA	1:D:154:VAL:CG1	2.48	0.44
1:E:45:LEU:HD22	1:E:81:PRO:HB2	2.00	0.44
1:A:375:ARG:CD	1:D:312:ARG:HD3	2.48	0.44
1:B:231[B]:LYS:HE2	1:B:231[B]:LYS:N	2.32	0.44
1:B:92:GLY:HA2	1:B:236:ASN:ND2	2.33	0.44
1:D:156:VAL:HG13	4:D:503:TRS:H12	1.99	0.44
1:A:234:ILE:O	1:A:237:MET:HB3	2.17	0.43
1:B:170:LEU:HD22	1:B:174:PHE:CZ	2.52	0.43
1:C:199:TYR:CZ	1:C:203:LEU:HD11	2.53	0.43
1:D:357[A]:ILE:CD1	2:D:501:HEM:HMD2	2.48	0.43
1:C:110[A]:LYS:O	1:C:116[A]:ARG:HG3	2.17	0.43
1:E:132:LEU:O	1:E:136:VAL:HG13	2.18	0.43
1:F:161:LEU:O	1:F:216:LEU:HB2	2.19	0.43
1:A:225:ASN:N	1:A:225:ASN:OD1	2.51	0.43

Continued on next page...

CONFIDENTIAL VALIDATION REPORT

Continued from previous page...

Atom-1	Atom-2	Interatomic distance (Å)	Clash overlap (Å)
1:A:231[B]:LYS:O	1:A:235:VAL:HG23	2.18	0.43
1:A:105:ARG:HD3	1:A:357[B]:ILE:HD11	2.01	0.43
1:C:17:TYR:HA	1:C:18:PRO:C	2.38	0.43
1:C:260:LEU:CD1	1:C:370[B]:LEU:HD11	2.48	0.43
1:D:183:ARG:HD3	1:D:394:GLY:CA	2.45	0.43
1:B:337:LEU:HD13	1:B:339:PHE:CE1	2.54	0.43
1:C:258:HIS:NE2	1:C:262:THR:HG21	2.34	0.43
1:C:73:ALA:O	1:C:76:THR:HG23	2.19	0.43
1:D:168:ARG:HA	1:D:171:PHE:CZ	2.53	0.43
1:E:42[A]:ARG:HD3	1:E:42[A]:ARG:HA	1.89	0.43
1:B:287:TYR:O	1:B:326:ARG:NH1	2.52	0.43
1:C:145:VAL:HA	1:C:149:ALA:HB3	2.01	0.43
1:D:270:LEU:HD21	1:D:280:ALA:HB2	2.00	0.43
1:D:287:TYR:O	1:D:326:ARG:NH1	2.52	0.43
1:F:361:LEU:HD23	2:F:501:HEM:HBC2	1.99	0.43
1:B:231[A]:LYS:NZ	1:F:67:ASP:OD2	2.52	0.43
1:B:270:LEU:HB2	1:B:374:VAL:HG21	2.00	0.43
1:B:384:GLU:CD	1:B:402:ARG:HH11	2.21	0.43
1:B:233:GLU:OE2	6:B:530:GOL:H11	2.19	0.43
1:C:119:GLU:OE1	8:C:602:HOH:O	2.20	0.43
1:C:183:ARG:HD2	8:C:673:HOH:O	2.18	0.43
1:C:123:ARG:NH2	6:C:529:GOL:O1	2.51	0.42
1:D:280:ALA:HA	1:D:339:PHE:CD1	2.54	0.42
1:A:150:VAL:N	1:A:151:PRO:HD2	2.34	0.42
1:A:42:ARG:NH2	8:A:608:HOH:O	2.43	0.42
1:B:107:LEU:HA	1:B:107:LEU:HD12	1.86	0.42
1:C:216:LEU:HD12	1:C:219:LEU:HD12	2.01	0.42
1:E:312[A]:ARG:O	1:E:315:GLU:HB2	2.19	0.42
1:A:216:LEU:HD12	1:A:219:LEU:HD12	1.99	0.42
1:B:145:VAL:HA	1:B:149:ALA:HB3	2.01	0.42
1:C:116[B]:ARG:HD2	8:C:719:HOH:O	2.20	0.42
1:C:330:VAL:HG22	1:C:331:PHE:CD2	2.55	0.42
1:E:208:ARG:CZ	1:E:223:THR:CG2	2.98	0.42
1:F:283:GLU:CA	1:F:283:GLU:OE1	2.66	0.42
1:F:64:VAL:HA	1:F:70:PHE:CE2	2.54	0.42
1:B:247:GLU:O	1:B:251:ASN:ND2	2.50	0.42
1:C:287:TYR:O	1:C:326:ARG:NH1	2.52	0.42
1:E:57[A]:ARG:NH2	1:E:329[A]:GLU:HB2	2.35	0.42
1:E:77:ASP:OD1	1:E:78:PRO:HD2	2.20	0.42
1:F:284:MET:O	1:F:288:THR:HG23	2.20	0.42
1:F:31:TYR:CZ	1:F:320:HIS:CD2	3.08	0.42

Continued on next page...

CONFIDENTIAL VALIDATION REPORT

Continued from previous page...

Atom-1	Atom-2	Interatomic distance (Å)	Clash overlap (Å)
1:A:276:LEU:HA	1:A:276:LEU:HD23	1.90	0.42
1:D:327:ASP:HB3	1:D:330:VAL:HG13	1.99	0.42
1:F:157:ILE:HG12	1:F:245:GLY:HA3	2.01	0.42
1:A:154:VAL:HA	1:A:157:ILE:HD11	2.02	0.42
1:B:284[A]:MET:HE3	1:B:339:PHE:HZ	1.85	0.42
1:E:115:ARG:HE	1:E:115:ARG:HB2	1.45	0.42
1:A:67:ASP:OD2	1:A:69:ARG:NH1	2.53	0.41
1:D:101:HIS:CE1	1:D:354:HIS:HD1	2.37	0.41
1:F:244:ALA:HB1	2:F:501:HEM:C4C	2.55	0.41
1:F:276:LEU:CD1	1:F:339:PHE:HB3	2.48	0.41
1:F:84:PHE:HB3	1:F:396:LEU:HD11	2.01	0.41
1:F:94:LEU:HD21	3:F:502:QR8:O12	2.20	0.41
1:B:207:ARG:HD3	1:B:212:THR:OG1	2.20	0.41
1:F:55:VAL:HG13	1:F:60:ASP:HB2	2.02	0.41
1:B:328:GLU:HB3	5:B:513:FMT:O2	2.20	0.41
2:D:501:HEM:HMB2	2:D:501:HEM:HBB2	2.01	0.41
1:B:27:LEU:HA	1:B:27:LEU:HD13	1.91	0.41
1:E:384:GLU:OE1	1:E:402:ARG:NH1	2.53	0.41
1:B:358:GLY:HA3	2:B:501:HEM:C3C	2.55	0.41
1:D:99:PRO:O	1:D:102:THR:HB	2.19	0.41
1:E:327:ASP:HB3	1:E:330:VAL:CG1	2.51	0.41
1:E:132:LEU:HG	1:E:377:PHE:HE2	1.84	0.41
1:F:108:VAL:CG2	1:F:215:LEU:HD22	2.50	0.41
2:A:501:HEM:CMC	2:A:501:HEM:HBC2	2.50	0.41
1:B:191:ARG:HA	1:B:191:ARG:HD2	1.85	0.41
1:D:357[A]:ILE:HD11	2:D:501:HEM:HMD1	2.03	0.41
1:E:170:LEU:HD23	1:E:170:LEU:HA	1.87	0.41
1:F:160:LEU:HD12	1:F:160:LEU:HA	1.78	0.41
1:F:168:ARG:HA	1:F:171:PHE:CE2	2.56	0.41
1:D:106:ARG:HH11	1:D:106:ARG:CG	2.33	0.41
1:D:183:ARG:HB3	1:D:184:LEU:HD12	2.03	0.41
1:D:324:ALA:HB3	1:D:347:ILE:HD11	2.03	0.41
1:E:74:ALA:HB3	1:E:299:VAL:HB	2.02	0.41
1:F:154:VAL:HG11	1:F:168:ARG:HD2	2.03	0.41
1:F:168:ARG:NH2	1:F:168:ARG:HB3	2.36	0.41
1:F:42:ARG:HD3	1:F:53:TRP:CH2	2.56	0.41
1:A:132:LEU:HG	1:A:377:PHE:HE2	1.85	0.41
1:B:46:PRO:HB2	1:B:47:TYR:CD1	2.55	0.41
1:D:132:LEU:O	1:D:136:VAL:HG12	2.21	0.41
1:D:172:ARG:HD2	8:D:617:HOH:O	2.19	0.41
1:D:75:ALA:HA	1:D:80:THR:HG21	2.03	0.41

Continued on next page...

CONFIDENTIAL VALIDATION REPORT

Continued from previous page...

Atom-1	Atom-2	Interatomic distance (Å)	Clash overlap (Å)
1:E:252:GLN:NE2	1:E:290:LEU:HB2	2.35	0.41
1:D:252:GLN:OE1	1:D:252:GLN:HA	2.21	0.41
1:D:258:HIS:NE2	1:D:262:THR:CG2	2.84	0.41
1:B:124:VAL:HG13	1:B:152:PHE:HE1	1.86	0.41
1:B:258:HIS:NE2	1:B:262:THR:HG21	2.36	0.41
1:C:216:LEU:HA	1:C:216:LEU:HD12	1.86	0.41
1:D:161:LEU:HA	1:D:161:LEU:HD12	1.83	0.41
1:C:283:GLU:HA	1:C:283:GLU:OE1	2.21	0.40
1:D:65:LEU:HB3	1:D:351:HIS:O	2.21	0.40
1:F:377:PHE:N	1:F:378:PRO:HD3	2.36	0.40
1:A:157:ILE:HD12	1:A:157:ILE:N	2.36	0.40
1:A:174:PHE:HB3	1:A:196:PHE:CD2	2.57	0.40
1:B:86[A]:THR:HG21	1:B:189:ILE:HG22	2.03	0.40
1:D:121:ARG:N	1:D:122:PRO:CD	2.83	0.40
1:D:170:LEU:HD23	1:D:170:LEU:C	2.41	0.40
1:D:266:ARG:NE	1:D:337:LEU:HD12	2.35	0.40
1:E:107:LEU:HD12	1:E:107:LEU:HA	1.91	0.40
1:E:168:ARG:HD2	8:E:634:HOH:O	2.22	0.40
1:A:108:VAL:HG22	1:A:215[A]:LEU:HD22	2.03	0.40
1:B:170:LEU:HD23	1:B:170:LEU:HA	1.78	0.40
1:B:384:GLU:OE1	1:B:402:ARG:NH1	2.54	0.40
1:C:197:MET:O	1:C:201:ASP:HB2	2.21	0.40
1:F:287:TYR:CD2	1:F:337[B]:LEU:HD11	2.56	0.40
1:C:252:GLN:HA	1:C:252:GLN:OE1	2.20	0.40
1:D:108:VAL:HG21	1:D:241:LEU:CD1	2.51	0.40
1:E:39:PRO:CG	1:E:57[B]:ARG:CZ	2.99	0.40
1:E:57[A]:ARG:HH21	1:E:329[A]:GLU:HB2	1.86	0.40
1:F:278:PRO:O	1:F:282:GLU:HG2	2.22	0.40
1:B:270:LEU:CB	1:B:374:VAL:HG21	2.52	0.40
1:D:190:GLN:O	1:D:194:GLN:HG3	2.22	0.40
1:E:402:ARG:HD2	1:E:404:ILE:HG13	2.04	0.40
1:F:277:VAL:HG12	1:F:278:PRO:HD3	2.03	0.40

All (2) symmetry-related close contacts are listed below. The label for Atom-2 includes the symmetry operator and encoded unit-cell translations to be applied.

Atom-1	Atom-2	Interatomic distance (Å)	Clash overlap (Å)
1:C:302:GLU:OE1	1:E:205:ALA:O[4_646]	2.07	0.13
1:C:103:ARG:NH1	1:E:169:ASP:OD2[4_646]	2.09	0.11

CONFIDENTIAL VALIDATION REPORT

5.3 Torsion angles (i)

5.3.1 Protein backbone (i)

In the following table, the Percentiles column shows the percent Ramachandran outliers of the chain as a percentile score with respect to all X-ray entries followed by that with respect to entries of similar resolution.

The Analysed column shows the number of residues for which the backbone conformation was analysed, and the total number of residues.

Mol	Chain	Analysed	Favoured	Allowed	Outliers	Percentiles	
1	A	401/407 (98%)	388 (97%)	13 (3%)	0	100	100
1	B	403/407 (99%)	389 (96%)	14 (4%)	0	100	100
1	C	401/407 (98%)	389 (97%)	12 (3%)	0	100	100
1	D	394/407 (97%)	364 (92%)	30 (8%)	0	100	100
1	E	406/407 (100%)	386 (95%)	20 (5%)	0	100	100
1	F	396/407 (97%)	372 (94%)	24 (6%)	0	100	100
All	All	2401/2442 (98%)	2288 (95%)	113 (5%)	0	100	100

There are no Ramachandran outliers to report.

5.3.2 Protein sidechains (i)

In the following table, the Percentiles column shows the percent sidechain outliers of the chain as a percentile score with respect to all X-ray entries followed by that with respect to entries of similar resolution.

The Analysed column shows the number of residues for which the sidechain conformation was analysed, and the total number of residues.

Mol	Chain	Analysed	Rotameric	Outliers	Percentiles	
1	A	339/341 (99%)	294 (87%)	45 (13%)	4	3
1	B	340/341 (100%)	307 (90%)	33 (10%)	8	7
1	C	339/341 (99%)	311 (92%)	28 (8%)	11	11
1	D	332/341 (97%)	284 (86%)	48 (14%)	3	2
1	E	344/341 (101%)	295 (86%)	49 (14%)	3	2
1	F	334/341 (98%)	289 (86%)	45 (14%)	4	3
All	All	2028/2046 (99%)	1780 (88%)	248 (12%)	5	4

CONFIDENTIAL VALIDATION REPORT

All (248) residues with a non-rotameric sidechain are listed below:

Mol	Chain	Res	Type
1	A	14	VAL
1	A	21	LEU
1	A	38	GLU
1	A	42	ARG
1	A	51	THR
1	A	69	ARG
1	A	93	VAL
1	A	94	LEU
1	A	108	VAL
1	A	110[A]	LYS
1	A	110[B]	LYS
1	A	123	ARG
1	A	127	LEU
1	A	132	LEU
1	A	157	ILE
1	A	161	LEU
1	A	165	LEU
1	A	168	ARG
1	A	196	PHE
1	A	198	VAL
1	A	207	ARG
1	A	209	ASP
1	A	216	LEU
1	A	221	LEU
1	A	223	THR
1	A	225	ASN
1	A	226	ASP
1	A	228	HIS
1	A	229	LEU
1	A	239	VAL
1	A	265	LYS
1	A	288	THR
1	A	312	ARG
1	A	329	GLU
1	A	337	LEU
1	A	341	ARG
1	A	343	ARG
1	A	344	ASN
1	A	357[A]	ILE
1	A	357[B]	ILE
1	A	364	LEU
1	A	374	VAL

Continued on next page...

CONFIDENTIAL VALIDATION REPORT

Continued from previous page...

Mol	Chain	Res	Type
1	A	397[A]	ILE
1	A	397[B]	ILE
1	A	402	ARG
1	B	21	LEU
1	B	42	ARG
1	B	45	LEU
1	B	93	VAL
1	B	106	ARG
1	B	107	LEU
1	B	127	LEU
1	B	132	LEU
1	B	157	ILE
1	B	180	SER
1	B	190	GLN
1	B	196	PHE
1	B	204	VAL
1	B	208	ARG
1	B	214	ASP
1	B	216	LEU
1	B	221	LEU
1	B	256	LEU
1	B	263[A]	GLU
1	B	263[B]	GLU
1	B	276	LEU
1	B	288	THR
1	B	309	VAL
1	B	312	ARG
1	B	328	GLU
1	B	337	LEU
1	B	342[A]	GLU
1	B	342[B]	GLU
1	B	343	ARG
1	B	364	LEU
1	B	374	VAL
1	B	401	GLU
1	B	402	ARG
1	C	27	LEU
1	C	42	ARG
1	C	45	LEU
1	C	51	THR
1	C	93	VAL
1	C	104	LEU

Continued on next page...

CONFIDENTIAL VALIDATION REPORT

Continued from previous page...

Mol	Chain	Res	Type
1	C	107	LEU
1	C	108	VAL
1	C	123	ARG
1	C	127	LEU
1	C	157	ILE
1	C	168	ARG
1	C	196	PHE
1	C	201	ASP
1	C	208	ARG
1	C	209	ASP
1	C	216	LEU
1	C	221	LEU
1	C	223	THR
1	C	252	GLN
1	C	263	GLU
1	C	288	THR
1	C	302	GLU
1	C	337	LEU
1	C	343	ARG
1	C	364	LEU
1	C	374	VAL
1	C	402	ARG
1	D	20	SER
1	D	21	LEU
1	D	27	LEU
1	D	36	ARG
1	D	41	SER
1	D	42	ARG
1	D	45	LEU
1	D	57	ARG
1	D	68	SER
1	D	71	SER
1	D	86	THR
1	D	93	VAL
1	D	104	LEU
1	D	106	ARG
1	D	108	VAL
1	D	115	ARG
1	D	116	ARG
1	D	126	SER
1	D	127	LEU
1	D	132	LEU

Continued on next page...

CONFIDENTIAL VALIDATION REPORT

Continued from previous page...

Mol	Chain	Res	Type
1	D	136	VAL
1	D	140	SER
1	D	159	GLU
1	D	165	LEU
1	D	191	ARG
1	D	196	PHE
1	D	207	ARG
1	D	216	LEU
1	D	221	LEU
1	D	224	ASP
1	D	236	ASN
1	D	263	GLU
1	D	276	LEU
1	D	288	THR
1	D	292	SER
1	D	297	VAL
1	D	305	GLU
1	D	307	SER
1	D	309	VAL
1	D	312	ARG
1	D	318	VAL
1	D	330	VAL
1	D	357[A]	ILE
1	D	357[B]	ILE
1	D	364	LEU
1	D	374	VAL
1	D	401	GLU
1	D	402	ARG
1	E	21	LEU
1	E	35	ARG
1	E	42[A]	ARG
1	E	42[B]	ARG
1	E	45	LEU
1	E	49[A]	GLU
1	E	49[B]	GLU
1	E	59	SER
1	E	71	SER
1	E	89	GLU
1	E	93	VAL
1	E	103	ARG
1	E	107	LEU
1	E	108	VAL

Continued on next page...

CONFIDENTIAL VALIDATION REPORT

Continued from previous page...

Mol	Chain	Res	Type
1	E	110[A]	LYS
1	E	110[B]	LYS
1	E	115	ARG
1	E	123	ARG
1	E	132	LEU
1	E	161	LEU
1	E	196	PHE
1	E	204	VAL
1	E	207	ARG
1	E	215	LEU
1	E	216	LEU
1	E	223	THR
1	E	224	ASP
1	E	227	ASP
1	E	236	ASN
1	E	252	GLN
1	E	256	LEU
1	E	263	GLU
1	E	264	ARG
1	E	265	LYS
1	E	276	LEU
1	E	303	ASP
1	E	305[A]	GLU
1	E	305[B]	GLU
1	E	309	VAL
1	E	312[A]	ARG
1	E	312[B]	ARG
1	E	318	VAL
1	E	326	ARG
1	E	330	VAL
1	E	337	LEU
1	E	343	ARG
1	E	364	LEU
1	E	374	VAL
1	E	402	ARG
1	F	41	SER
1	F	42	ARG
1	F	44	ARG
1	F	45	LEU
1	F	68	SER
1	F	93	VAL
1	F	104	LEU

Continued on next page...

CONFIDENTIAL VALIDATION REPORT

Continued from previous page...

Mol	Chain	Res	Type
1	F	107	LEU
1	F	110	LYS
1	F	117	VAL
1	F	123	ARG
1	F	125	ARG
1	F	127	LEU
1	F	129	ASP
1	F	132	LEU
1	F	140	SER
1	F	159	GLU
1	F	161	LEU
1	F	180	SER
1	F	185	THR
1	F	190	GLN
1	F	196	PHE
1	F	207	ARG
1	F	208	ARG
1	F	214	ASP
1	F	216	LEU
1	F	221	LEU
1	F	225	ASN
1	F	236	ASN
1	F	265	LYS
1	F	271	VAL
1	F	273	ASP
1	F	276	LEU
1	F	277	VAL
1	F	309	VAL
1	F	312	ARG
1	F	328	GLU
1	F	329	GLU
1	F	330	VAL
1	F	344	ASN
1	F	364	LEU
1	F	374	VAL
1	F	386	VAL
1	F	393	GLN
1	F	402	ARG

Some sidechains can be flipped to improve hydrogen bonding and reduce clashes. All (34) such sidechains are listed below:

CONFIDENTIAL VALIDATION REPORT

Mol	Chain	Res	Type
1	A	30	HIS
1	A	96	GLN
1	A	101	HIS
1	A	236	ASN
1	A	340	HIS
1	A	344	ASN
1	A	346	HIS
1	B	30	HIS
1	B	101	HIS
1	B	236	ASN
1	B	346	HIS
1	B	360	GLN
1	B	393	GLN
1	C	101	HIS
1	C	193	GLN
1	C	194	GLN
1	C	206	GLN
1	C	346	HIS
1	D	30	HIS
1	D	101	HIS
1	D	194	GLN
1	D	228	HIS
1	D	320	HIS
1	D	346	HIS
1	E	101	HIS
1	E	194	GLN
1	E	225	ASN
1	E	236	ASN
1	E	346	HIS
1	F	96	GLN
1	F	101	HIS
1	F	206	GLN
1	F	344	ASN
1	F	346	HIS

5.3.3 RNA (i)

There are no RNA molecules in this entry.

5.4 Non-standard residues in protein, DNA, RNA chains (i)

There are no non-standard protein/DNA/RNA residues in this entry.

CONFIDENTIAL VALIDATION REPORT

5.5 Carbohydrates (i)

There are no monosaccharides in this entry.

5.6 Ligand geometry (i)

Of 118 ligands modelled in this entry, 5 are monoatomic - leaving 113 for Mogul analysis.

In the following table, the Counts columns list the number of bonds (or angles) for which Mogul statistics could be retrieved, the number of bonds (or angles) that are observed in the model and the number of bonds (or angles) that are defined in the Chemical Component Dictionary. The Link column lists molecule types, if any, to which the group is linked. The Z score for a bond length (or angle) is the number of standard deviations the observed value is removed from the expected value. A bond length (or angle) with $|Z| > 2$ is considered an outlier worth inspection. RMSZ is the root-mean-square of all Z scores of the bond lengths (or angles).

Mol	Type	Chain	Res	Link	Bond lengths			Bond angles		
					Counts	RMSZ	$\#\|Z\|>2$	Counts	RMSZ	$\#\|Z\|>2$
5	FMT	B	516	-	0,2,2	0.00	-	0,1,1	0.00	-
5	FMT	B	520	-	0,2,2	0.00	-	0,1,1	0.00	-
5	FMT	C	513	-	0,2,2	0.00	-	0,1,1	0.00	-
2	HEM	E	501	1	27,50,50	1.00	2 (7%)	17,82,82	2.13	6 (35%)
5	FMT	C	520	-	0,2,2	0.00	-	0,1,1	0.00	-
5	FMT	B	518	-	0,2,2	0.00	-	0,1,1	0.00	-
5	FMT	C	523	-	0,2,2	0.00	-	0,1,1	0.00	-
5	FMT	D	505	-	0,2,2	0.00	-	0,1,1	0.00	-
5	FMT	B	510	-	0,2,2	0.00	-	0,1,1	0.00	-
5	FMT	D	504	-	0,2,2	0.00	-	0,1,1	0.00	-
5	FMT	B	519	-	0,2,2	0.00	-	0,1,1	0.00	-
5	FMT	B	513	-	0,2,2	0.00	-	0,1,1	0.00	-
2	HEM	D	501	1	27,50,50	1.89	5 (18%)	17,82,82	1.89	5 (29%)
6	GOL	C	524	-	5,5,5	0.20	0	5,5,5	0.46	0
5	FMT	A	504	-	0,2,2	0.00	-	0,1,1	0.00	-
5	FMT	A	509	-	0,2,2	0.00	-	0,1,1	0.00	-
6	GOL	F	512	-	5,5,5	0.11	0	5,5,5	0.33	0
5	FMT	D	510	-	0,2,2	0.00	-	0,1,1	0.00	-
5	FMT	B	525	-	0,2,2	0.00	-	0,1,1	0.00	-
5	FMT	B	521	-	0,2,2	0.00	-	0,1,1	0.00	-
6	GOL	B	532	-	5,5,5	0.16	0	5,5,5	0.37	0
5	FMT	A	505	-	0,2,2	0.00	-	0,1,1	0.00	-
5	FMT	E	504	-	0,2,2	0.00	-	0,1,1	0.00	-
5	FMT	B	523	-	0,2,2	0.00	-	0,1,1	0.00	-
5	FMT	F	507	-	0,2,2	0.00	-	0,1,1	0.00	-
5	FMT	C	507	-	0,2,2	0.00	-	0,1,1	0.00	-
5	FMT	A	506	-	0,2,2	0.00	-	0,1,1	0.00	-

CONFIDENTIAL VALIDATION REPORT

Mol	Type	Chain	Res	Link	Bond lengths			Bond angles		
					Counts	RMSZ	#|Z| > 2	Counts	RMSZ	#|Z| > 2
5	FMT	F	504	-	0,2,2	0.00	-	0,1,1	0.00	-
3	QR8	B	502	-	26,26,26	1.74	5 (19%)	35,38,38	1.81	10 (28%)
5	FMT	F	511	-	0,2,2	0.00	-	0,1,1	0.00	-
5	FMT	B	505	-	0,2,2	0.00	-	0,1,1	0.00	-
5	FMT	A	513	-	0,2,2	0.00	-	0,1,1	0.00	-
5	FMT	B	506	-	0,2,2	0.00	-	0,1,1	0.00	-
6	GOL	B	528	-	5,5,5	0.13	0	5,5,5	0.40	0
5	FMT	A	514	-	0,2,2	0.00	-	0,1,1	0.00	-
5	FMT	C	511	-	0,2,2	0.00	-	0,1,1	0.00	-
5	FMT	C	509	-	0,2,2	0.00	-	0,1,1	0.00	-
3	QR8	A	502	-	26,26,26	1.49	3 (11%)	35,38,38	1.54	6 (17%)
5	FMT	B	526	-	0,2,2	0.00	-	0,1,1	0.00	-
5	FMT	C	516	-	0,2,2	0.00	-	0,1,1	0.00	-
5	FMT	F	505	-	0,2,2	0.00	-	0,1,1	0.00	-
5	FMT	E	508	-	0,2,2	0.00	-	0,1,1	0.00	-
5	FMT	C	519	-	0,2,2	0.00	-	0,1,1	0.00	-
5	FMT	E	507	-	0,2,2	0.00	-	0,1,1	0.00	-
5	FMT	C	510	-	0,2,2	0.00	-	0,1,1	0.00	-
5	FMT	A	516	-	0,2,2	0.00	-	0,1,1	0.00	-
5	FMT	C	522	-	0,2,2	0.00	-	0,1,1	0.00	-
6	GOL	C	529	-	5,5,5	0.15	0	5,5,5	0.39	0
5	FMT	C	504	-	0,2,2	0.00	-	0,1,1	0.00	-
5	FMT	B	511	-	0,2,2	0.00	-	0,1,1	0.00	-
2	HEM	F	501	1	27,50,50	0.91	2 (7%)	17,82,82	2.04	5 (29%)
5	FMT	E	505	-	0,2,2	0.00	-	0,1,1	0.00	-
3	QR8	C	502	-	26,26,26	1.52	4 (15%)	35,38,38	1.73	8 (22%)
5	FMT	C	521	-	0,2,2	0.00	-	0,1,1	0.00	-
5	FMT	B	508	-	0,2,2	0.00	-	0,1,1	0.00	-
5	FMT	D	506	-	0,2,2	0.00	-	0,1,1	0.00	-
5	FMT	E	506	-	0,2,2	0.00	-	0,1,1	0.00	-
4	TRS	F	503	-	7,7,7	0.45	0	9,9,9	0.75	0
5	FMT	C	503	-	0,2,2	0.00	-	0,1,1	0.00	-
3	QR8	F	502	-	26,26,26	1.51	3 (11%)	35,38,38	1.72	12 (34%)
6	GOL	C	525	-	5,5,5	0.11	0	5,5,5	0.23	0
5	FMT	A	510	-	0,2,2	0.00	-	0,1,1	0.00	-
5	FMT	B	503	-	0,2,2	0.00	-	0,1,1	0.00	-
6	GOL	D	512	-	5,5,5	0.08	0	5,5,5	0.22	0
2	HEM	B	501	1	27,50,50	1.90	7 (25%)	17,82,82	1.92	3 (17%)
5	FMT	E	509	-	0,2,2	0.00	-	0,1,1	0.00	-
5	FMT	F	508	-	0,2,2	0.00	-	0,1,1	0.00	-
6	GOL	A	518	-	5,5,5	0.14	0	5,5,5	0.43	0
5	FMT	B	515	-	0,2,2	0.00	-	0,1,1	0.00	-

CONFIDENTIAL VALIDATION REPORT

Mol	Type	Chain	Res	Link	Bond lengths			Bond angles		
					Counts	RMSZ	#|Z| > 2	Counts	RMSZ	#|Z| > 2
5	FMT	F	509	-	0,2,2	0.00	-	0,1,1	0.00	-
6	GOL	B	530	-	5,5,5	0.12	0	5,5,5	0.37	0
5	FMT	E	503	-	0,2,2	0.00	-	0,1,1	0.00	-
5	FMT	F	510	-	0,2,2	0.00	-	0,1,1	0.00	-
6	GOL	C	526	-	5,5,5	0.30	0	5,5,5	0.83	0
5	FMT	D	508	-	0,2,2	0.00	-	0,1,1	0.00	-
5	FMT	C	508	-	0,2,2	0.00	-	0,1,1	0.00	-
6	GOL	B	527	-	5,5,5	0.11	0	5,5,5	0.33	0
5	FMT	C	514	-	0,2,2	0.00	-	0,1,1	0.00	-
5	FMT	A	517	-	0,2,2	0.00	-	0,1,1	0.00	-
5	FMT	C	518	-	0,2,2	0.00	-	0,1,1	0.00	-
5	FMT	B	514	-	0,2,2	0.00	-	0,1,1	0.00	-
5	FMT	C	506	-	0,2,2	0.00	-	0,1,1	0.00	-
5	FMT	B	517	-	0,2,2	0.00	-	0,1,1	0.00	-
5	FMT	A	515	-	0,2,2	0.00	-	0,1,1	0.00	-
4	TRS	D	503	-	7,7,7	0.29	0	9,9,9	0.76	0
5	FMT	B	522	-	0,2,2	0.00	-	0,1,1	0.00	-
2	HEM	C	501	1	27,50,50	1.53	6 (22%)	17,82,82	1.98	5 (29%)
5	FMT	E	510	-	0,2,2	0.00	-	0,1,1	0.00	-
5	FMT	B	524	-	0,2,2	0.00	-	0,1,1	0.00	-
6	GOL	C	527	-	5,5,5	0.09	0	5,5,5	0.14	0
4	TRS	A	503	-	7,7,7	0.27	0	9,9,9	0.77	0
5	FMT	D	507	-	0,2,2	0.00	-	0,1,1	0.00	-
5	FMT	C	515	-	0,2,2	0.00	-	0,1,1	0.00	-
6	GOL	C	528	-	5,5,5	0.14	0	5,5,5	0.35	0
5	FMT	C	505	-	0,2,2	0.00	-	0,1,1	0.00	-
5	FMT	A	512	-	0,2,2	0.00	-	0,1,1	0.00	-
5	FMT	D	509	-	0,2,2	0.00	-	0,1,1	0.00	-
5	FMT	D	511	-	0,2,2	0.00	-	0,1,1	0.00	-
5	FMT	C	517	-	0,2,2	0.00	-	0,1,1	0.00	-
5	FMT	A	511	-	0,2,2	0.00	-	0,1,1	0.00	-
2	HEM	A	501	1	27,50,50	1.45	3 (11%)	17,82,82	2.02	5 (29%)
5	FMT	F	506	-	0,2,2	0.00	-	0,1,1	0.00	-
5	FMT	B	512	-	0,2,2	0.00	-	0,1,1	0.00	-
5	FMT	A	508	-	0,2,2	0.00	-	0,1,1	0.00	-
6	GOL	B	529	-	5,5,5	0.11	0	5,5,5	0.29	0
3	QR8	D	502	-	26,26,26	1.65	3 (11%)	35,38,38	1.82	8 (22%)
5	FMT	A	507	-	0,2,2	0.00	-	0,1,1	0.00	-
5	FMT	C	512	-	0,2,2	0.00	-	0,1,1	0.00	-
5	FMT	B	509	-	0,2,2	0.00	-	0,1,1	0.00	-
5	FMT	B	507	-	0,2,2	0.00	-	0,1,1	0.00	-
6	GOL	B	531	-	5,5,5	0.16	0	5,5,5	0.34	0

CONFIDENTIAL VALIDATION REPORT

Mol	Type	Chain	Res	Link	Bond lengths			Bond angles		
					Counts	RMSZ	#|Z| > 2	Counts	RMSZ	#|Z| > 2
5	FMT	B	504	-	0,2,2	0.00	-	0,1,1	0.00	-
3	QR8	E	502	-	26,26,26	1.59	3 (11%)	35,38,38	1.53	8 (22%)

In the following table, the Chirals column lists the number of chiral outliers, the number of chiral centers analysed, the number of these observed in the model and the number defined in the Chemical Component Dictionary. Similar counts are reported in the Torsion and Rings columns. '-' means no outliers of that kind were identified.

Mol	Type	Chain	Res	Link	Chirals	Torsions	Rings
6	GOL	C	527	-	-	2/4/4/4	-
3	QR8	B	502	-	-	11/48/48/48	0/1/1/1
4	TRS	F	503	-	-	6/9/9/9	-
4	TRS	A	503	-	-	9/9/9/9	-
3	QR8	F	502	-	-	17/48/48/48	0/1/1/1
2	HEM	E	501	1	-	0/6/54/54	-
6	GOL	B	528	-	-	2/4/4/4	-
6	GOL	D	512	-	-	0/4/4/4	-
2	HEM	B	501	1	-	0/6/54/54	-
3	QR8	A	502	-	-	12/48/48/48	0/1/1/1
6	GOL	C	528	-	-	2/4/4/4	-
6	GOL	A	518	-	-	3/4/4/4	-
6	GOL	B	530	-	-	2/4/4/4	-
6	GOL	C	526	-	-	3/4/4/4	-
2	HEM	D	501	1	-	0/6/54/54	-
2	HEM	A	501	1	-	0/6/54/54	-
6	GOL	C	524	-	-	2/4/4/4	-
6	GOL	B	529	-	-	4/4/4/4	-
6	GOL	B	527	-	-	0/4/4/4	-
6	GOL	C	529	-	-	2/4/4/4	-
3	QR8	D	502	-	-	13/48/48/48	0/1/1/1
6	GOL	F	512	-	-	1/4/4/4	-
2	HEM	F	501	1	-	0/6/54/54	-
6	GOL	B	532	-	-	4/4/4/4	-
3	QR8	C	502	-	-	11/48/48/48	0/1/1/1
4	TRS	D	503	-	-	9/9/9/9	-
6	GOL	C	525	-	-	2/4/4/4	-
2	HEM	C	501	1	-	0/6/54/54	-
6	GOL	B	531	-	-	2/4/4/4	-

Continued on next page...

CONFIDENTIAL VALIDATION REPORT

Continued from previous page...

Mol	Type	Chain	Res	Link	Chirals	Torsions	Rings
3	QR8	E	502	-	-	14/48/48/48	0/1/1/1

All (46) bond length outliers are listed below:

Mol	Chain	Res	Type	Atoms	Z	Observed(Å)	Ideal(Å)
2	D	501	HEM	C3B-C2B	-5.68	1.32	1.40
3	B	502	QR8	O2-C13	-5.03	1.38	1.46
2	B	501	HEM	C3B-C2B	-4.73	1.33	1.40
3	D	502	QR8	O2-C13	-4.55	1.39	1.46
3	E	502	QR8	O2-C13	-4.52	1.39	1.46
3	A	502	QR8	O2-C13	-4.50	1.39	1.46
2	D	501	HEM	C4B-NB	-4.28	1.27	1.36
3	C	502	QR8	O2-C13	-4.17	1.39	1.46
2	A	501	HEM	C3B-C2B	-3.94	1.34	1.40
3	F	502	QR8	O2-C1	3.92	1.43	1.34
3	E	502	QR8	O2-C1	3.75	1.43	1.34
3	D	502	QR8	C10-C9	-3.72	1.46	1.52
2	D	501	HEM	C1D-ND	-3.69	1.28	1.36
2	C	501	HEM	C3B-C2B	-3.69	1.35	1.40
3	F	502	QR8	O2-C13	-3.63	1.40	1.46
2	B	501	HEM	C4B-NB	-3.52	1.28	1.36
3	B	502	QR8	C10-C9	-3.48	1.47	1.52
3	D	502	QR8	O2-C1	3.41	1.42	1.34
3	A	502	QR8	O2-C1	3.40	1.42	1.34
3	C	502	QR8	O2-C1	3.35	1.42	1.34
3	B	502	QR8	O2-C1	3.30	1.42	1.34
2	B	501	HEM	C3C-C2C	-3.29	1.35	1.40
2	B	501	HEM	CMD-C2D	-3.15	1.45	1.51
3	F	502	QR8	C10-C9	-3.05	1.48	1.52
2	B	501	HEM	C1D-ND	-3.05	1.29	1.36
2	C	501	HEM	C4B-NB	-2.91	1.30	1.36
2	E	501	HEM	C4D-C3D	2.90	1.49	1.42
2	D	501	HEM	C1A-CHA	-2.62	1.33	1.41
2	A	501	HEM	C4B-NB	-2.61	1.30	1.36
2	C	501	HEM	C4D-C3D	2.53	1.48	1.42
2	A	501	HEM	C4D-C3D	2.53	1.48	1.42
3	E	502	QR8	C10-C9	-2.48	1.48	1.52
3	C	502	QR8	C10-C9	-2.47	1.48	1.52
3	A	502	QR8	C10-C9	-2.33	1.49	1.52
2	C	501	HEM	C1D-CHD	-2.31	1.34	1.41
2	B	501	HEM	C3C-CAC	-2.30	1.43	1.47
2	C	501	HEM	CMD-C2D	-2.26	1.46	1.51

Continued on next page...

CONFIDENTIAL VALIDATION REPORT

Continued from previous page...

Mol	Chain	Res	Type	Atoms	Z	Observed(Å)	Ideal(Å)
2	E	501	HEM	C4B-NB	-2.22	1.31	1.36
2	D	501	HEM	CAD-C3D	-2.19	1.48	1.52
2	C	501	HEM	C3C-C2C	-2.16	1.37	1.40
3	C	502	QR8	C7-C6	-2.15	1.50	1.54
3	B	502	QR8	C12-C11	-2.05	1.48	1.54
2	B	501	HEM	C3B-CAB	2.05	1.52	1.47
2	F	501	HEM	CMA-C3A	-2.04	1.47	1.51
3	B	502	QR8	C7-C6	-2.03	1.50	1.54
2	F	501	HEM	CAD-C3D	-2.02	1.48	1.52

All (81) bond angle outliers are listed below:

Mol	Chain	Res	Type	Atoms	Z	Observed(^{o})	Ideal(^{o})
3	B	502	QR8	C36-C13-C12	-5.05	107.08	114.39
2	E	501	HEM	CBA-CAA-C2A	4.80	121.34	112.49
3	C	502	QR8	C8-C9-C10	-4.52	111.25	119.10
2	B	501	HEM	CMB-C2B-C3B	4.51	133.12	124.68
2	F	501	HEM	CBA-CAA-C2A	4.41	120.62	112.49
2	B	501	HEM	CBD-CAD-C3D	-4.20	104.75	112.48
2	E	501	HEM	C1D-C2D-C3D	-4.09	104.15	107.00
2	A	501	HEM	CBA-CAA-C2A	4.04	119.95	112.49
2	A	501	HEM	CBD-CAD-C3D	-4.02	105.07	112.48
2	D	501	HEM	CBA-CAA-C2A	3.96	119.79	112.49
2	C	501	HEM	CBA-CAA-C2A	3.93	119.74	112.49
2	F	501	HEM	C4A-C3A-C2A	3.78	109.63	107.00
3	D	502	QR8	C2-C3-C4	-3.76	106.65	114.41
3	D	502	QR8	O2-C1-C2	3.72	119.72	111.56
2	C	501	HEM	C4A-C3A-C2A	3.66	109.55	107.00
3	D	502	QR8	C36-C13-C12	-3.50	109.32	114.39
3	F	502	QR8	O2-C1-C2	3.50	119.25	111.56
3	D	502	QR8	C11-C10-C9	-3.38	104.24	110.36
2	C	501	HEM	CMC-C2C-C3C	3.35	130.95	124.68
3	F	502	QR8	C36-C13-C12	-3.31	109.59	114.39
3	E	502	QR8	O2-C1-C2	3.28	118.76	111.56
2	F	501	HEM	CBD-CAD-C3D	-3.27	106.46	112.48
3	C	502	QR8	O11-C9-C10	3.23	125.20	120.60
3	C	502	QR8	O2-C1-C2	3.23	118.64	111.56
2	D	501	HEM	CMC-C2C-C3C	3.20	130.67	124.68
3	B	502	QR8	C8-C9-C10	-3.15	113.64	119.10
3	B	502	QR8	O2-C1-C2	3.11	118.38	111.56
3	D	502	QR8	C34-C10-C11	3.07	117.50	112.37
2	C	501	HEM	CMA-C3A-C2A	-3.06	119.17	124.94

Continued on next page...

CONFIDENTIAL VALIDATION REPORT

Continued from previous page...

Mol	Chain	Res	Type	Atoms	Z	Observed(°)	Ideal(°)
3	F	502	QR8	C11-C10-C9	-3.05	104.83	110.36
3	E	502	QR8	C36-C13-C12	-3.04	109.98	114.39
2	A	501	HEM	CMC-C2C-C3C	3.01	130.31	124.68
3	A	502	QR8	C36-C13-C12	-3.00	110.04	114.39
2	E	501	HEM	CMB-C2B-C3B	2.99	130.28	124.68
3	A	502	QR8	O2-C1-O1	-2.98	118.37	123.94
2	A	501	HEM	C4A-C3A-C2A	2.93	109.03	107.00
3	A	502	QR8	C8-C9-C10	-2.92	114.04	119.10
3	E	502	QR8	C2-C3-C4	-2.90	108.44	114.41
3	C	502	QR8	C34-C10-C11	2.88	117.17	112.37
2	D	501	HEM	CMA-C3A-C4A	-2.87	124.06	128.46
3	C	502	QR8	C2-C3-C4	-2.74	108.77	114.41
3	D	502	QR8	C8-C9-C10	-2.73	114.36	119.10
3	F	502	QR8	C10-C11-C12	-2.73	108.78	114.41
3	A	502	QR8	O2-C1-C2	2.72	117.53	111.56
3	B	502	QR8	C34-C10-C11	2.70	116.88	112.37
3	B	502	QR8	C32-C6-C5	-2.67	106.58	111.54
3	F	502	QR8	C2-C3-C4	-2.64	108.96	114.41
3	F	502	QR8	O2-C1-O1	-2.60	119.08	123.94
3	F	502	QR8	C32-C6-C5	-2.59	106.74	111.54
3	F	502	QR8	C32-C6-C7	-2.57	106.84	110.69
3	E	502	QR8	C32-C6-C5	-2.54	106.82	111.54
3	B	502	QR8	C2-C3-C4	-2.52	109.22	114.41
3	B	502	QR8	C10-C11-C12	-2.52	109.22	114.41
2	B	501	HEM	CBA-CAA-C2A	2.51	117.11	112.49
3	E	502	QR8	C8-C9-C10	-2.47	114.81	119.10
2	F	501	HEM	CMC-C2C-C3C	2.45	129.27	124.68
3	C	502	QR8	O2-C1-O1	-2.45	119.36	123.94
3	D	502	QR8	C32-C6-C5	-2.41	107.06	111.54
3	B	502	QR8	O2-C1-O1	-2.39	119.48	123.94
3	E	502	QR8	O2-C1-O1	-2.39	119.48	123.94
2	A	501	HEM	CMB-C2B-C3B	2.36	129.09	124.68
3	F	502	QR8	C7-C8-C9	2.28	116.21	110.85
2	E	501	HEM	CAA-CBA-CGA	-2.27	108.86	112.67
2	C	501	HEM	CBD-CAD-C3D	-2.26	108.31	112.48
3	E	502	QR8	C32-C6-C7	-2.23	107.34	110.69
2	D	501	HEM	C3C-C4C-NC	-2.23	106.74	110.94
3	A	502	QR8	C7-C8-C9	2.23	116.08	110.85
3	C	502	QR8	O12-C11-C10	2.22	113.98	108.82
3	F	502	QR8	C8-C9-C10	-2.20	115.29	119.10
3	F	502	QR8	C33-C8-C9	-2.19	104.19	109.44
3	A	502	QR8	C34-C10-C9	2.16	111.83	108.08

Continued on next page...

CONFIDENTIAL VALIDATION REPORT

Continued from previous page...

Mol	Chain	Res	Type	Atoms	Z	Observed(°)	Ideal(°)
3	D	502	QR8	O11-C9-C8	2.12	125.19	121.26
3	B	502	QR8	C32-C6-C7	-2.12	107.52	110.69
2	F	501	HEM	CAA-CBA-CGA	-2.10	109.14	112.67
3	C	502	QR8	C36-C13-C12	-2.10	111.36	114.39
3	B	502	QR8	O11-C9-C8	2.09	125.14	121.26
2	E	501	HEM	CBD-CAD-C3D	-2.07	108.67	112.48
3	E	502	QR8	C13-O2-C1	-2.07	114.52	117.51
2	D	501	HEM	CMB-C2B-C3B	2.07	128.54	124.68
3	F	502	QR8	C13-O2-C1	-2.04	114.55	117.51
2	E	501	HEM	CMC-C2C-C3C	2.04	128.49	124.68

There are no chirality outliers.

All (133) torsion outliers are listed below:

Mol	Chain	Res	Type	Atoms
6	C	524	GOL	C1-C2-C3-O3
6	C	524	GOL	O2-C2-C3-O3
6	B	532	GOL	C1-C2-C3-O3
6	B	532	GOL	O2-C2-C3-O3
3	B	502	QR8	C6-C7-C8-C9
3	B	502	QR8	C31-C4-C5-O7
3	B	502	QR8	C3-C4-C5-O7
6	B	528	GOL	C1-C2-C3-O3
3	A	502	QR8	C6-C7-C8-C9
3	A	502	QR8	C6-C7-C8-C33
3	A	502	QR8	C3-C4-C5-O7
6	C	529	GOL	C1-C2-C3-O3
3	C	502	QR8	C6-C7-C8-C9
3	C	502	QR8	C6-C7-C8-C33
3	C	502	QR8	C3-C4-C5-O7
4	F	503	TRS	C1-C-C2-O2
4	F	503	TRS	C1-C-C3-O3
4	F	503	TRS	N-C-C3-O3
3	F	502	QR8	C5-C6-C7-C8
3	F	502	QR8	C4-C5-C6-C7
3	F	502	QR8	C30-C2-C3-C4
6	A	518	GOL	O1-C1-C2-C3
6	B	530	GOL	O1-C1-C2-C3
4	D	503	TRS	C2-C-C1-O1
4	D	503	TRS	C3-C-C1-O1
4	D	503	TRS	N-C-C1-O1
4	D	503	TRS	N-C-C2-O2

Continued on next page...

CONFIDENTIAL VALIDATION REPORT

Continued from previous page...

Mol	Chain	Res	Type	Atoms
4	D	503	TRS	C1-C-C3-O3
4	D	503	TRS	C2-C-C3-O3
6	C	527	GOL	C1-C2-C3-O3
4	A	503	TRS	C3-C-C1-O1
4	A	503	TRS	N-C-C1-O1
4	A	503	TRS	C1-C-C2-O2
4	A	503	TRS	C3-C-C2-O2
4	A	503	TRS	N-C-C2-O2
6	B	529	GOL	O1-C1-C2-C3
3	D	502	QR8	C6-C7-C8-C9
3	D	502	QR8	C6-C7-C8-C33
3	D	502	QR8	C31-C4-C5-O7
3	D	502	QR8	C3-C4-C5-O7
6	B	531	GOL	O1-C1-C2-C3
3	E	502	QR8	C6-C7-C8-C9
3	E	502	QR8	C3-C4-C5-O7
3	E	502	QR8	C30-C2-C3-C4
3	C	502	QR8	C31-C4-C5-O7
3	E	502	QR8	C31-C4-C5-O7
6	B	528	GOL	O2-C2-C3-O3
6	A	518	GOL	O1-C1-C2-O2
3	B	502	QR8	C30-C2-C3-O3
3	A	502	QR8	C30-C2-C3-O3
3	C	502	QR8	C30-C2-C3-O3
3	F	502	QR8	C30-C2-C3-O3
3	D	502	QR8	C30-C2-C3-O3
3	E	502	QR8	C30-C2-C3-O3
3	A	502	QR8	C31-C4-C5-O7
3	B	502	QR8	C30-C2-C3-C4
3	A	502	QR8	C30-C2-C3-C4
3	C	502	QR8	C30-C2-C3-C4
3	D	502	QR8	C30-C2-C3-C4
3	F	502	QR8	C3-C4-C5-O7
3	D	502	QR8	C31-C4-C5-C6
3	B	502	QR8	C3-C4-C5-C6
3	C	502	QR8	C3-C4-C5-C6
3	D	502	QR8	C3-C4-C5-C6
6	B	532	GOL	O1-C1-C2-C3
6	C	525	GOL	C1-C2-C3-O3
6	A	518	GOL	C1-C2-C3-O3
6	C	528	GOL	C1-C2-C3-O3
6	B	529	GOL	C1-C2-C3-O3

Continued on next page...

CONFIDENTIAL VALIDATION REPORT

Continued from previous page...

Mol	Chain	Res	Type	Atoms
3	E	502	QR8	C3-C4-C5-C6
3	F	502	QR8	O7-C5-C6-C7
6	C	525	GOL	O2-C2-C3-O3
6	B	530	GOL	O1-C1-C2-O2
6	B	529	GOL	O1-C1-C2-O2
3	B	502	QR8	C6-C7-C8-C33
3	E	502	QR8	C6-C7-C8-C33
3	B	502	QR8	C31-C4-C5-C6
3	C	502	QR8	C31-C4-C5-C6
3	E	502	QR8	C31-C4-C5-C6
3	F	502	QR8	C32-C6-C7-C8
4	F	503	TRS	C3-C-C2-O2
4	D	503	TRS	C1-C-C2-O2
3	F	502	QR8	C6-C7-C8-C9
3	A	502	QR8	C3-C4-C5-C6
6	C	527	GOL	O2-C2-C3-O3
6	B	531	GOL	O1-C1-C2-O2
3	B	502	QR8	C5-C6-C7-C8
6	C	529	GOL	O2-C2-C3-O3
6	C	526	GOL	O2-C2-C3-O3
3	A	502	QR8	C31-C4-C5-C6
3	D	502	QR8	C32-C6-C7-C8
6	B	532	GOL	O1-C1-C2-O2
6	C	526	GOL	O1-C1-C2-O2
4	F	503	TRS	C2-C-C3-O3
4	D	503	TRS	N-C-C3-O3
4	A	503	TRS	C2-C-C1-O1
4	A	503	TRS	N-C-C3-O3
3	F	502	QR8	C3-C4-C5-C6
3	A	502	QR8	C1-C2-C3-C4
3	A	502	QR8	C1-C2-C3-O3
3	C	502	QR8	C1-C2-C3-C4
3	F	502	QR8	C1-C2-C3-C4
3	F	502	QR8	C1-C2-C3-O3
3	D	502	QR8	C1-C2-C3-C4
3	D	502	QR8	C1-C2-C3-O3
3	E	502	QR8	C1-C2-C3-C4
3	E	502	QR8	C1-C2-C3-O3
3	D	502	QR8	C5-C6-C7-C8
3	F	502	QR8	C31-C4-C5-O7
3	B	502	QR8	C33-C8-C9-C10
3	F	502	QR8	C33-C8-C9-C10

Continued on next page...

CONFIDENTIAL VALIDATION REPORT

Continued from previous page...

Mol	Chain	Res	Type	Atoms
3	F	502	QR8	C7-C8-C9-C10
3	B	502	QR8	C32-C6-C7-C8
3	A	502	QR8	C32-C6-C7-C8
3	C	502	QR8	C32-C6-C7-C8
4	A	503	TRS	C1-C-C3-O3
4	A	503	TRS	C2-C-C3-O3
3	F	502	QR8	C6-C7-C8-C33
3	E	502	QR8	C2-C1-O2-C13
3	F	502	QR8	O7-C5-C6-C32
6	C	528	GOL	O2-C2-C3-O3
6	B	529	GOL	O2-C2-C3-O3
3	F	502	QR8	C4-C5-C6-C32
3	E	502	QR8	C32-C6-C7-C8
6	F	512	GOL	O2-C2-C3-O3
3	A	502	QR8	C5-C6-C7-C8
3	C	502	QR8	C5-C6-C7-C8
4	F	503	TRS	N-C-C2-O2
4	D	503	TRS	C3-C-C2-O2
6	C	526	GOL	O1-C1-C2-C3
3	E	502	QR8	C9-C10-C11-O12
3	D	502	QR8	C33-C8-C9-C10
3	E	502	QR8	O1-C1-O2-C13

There are no ring outliers.

26 monomers are involved in 65 short contacts:

Mol	Chain	Res	Type	Clashes	Symm-Clashes
2	E	501	HEM	4	0
5	B	513	FMT	1	0
2	D	501	HEM	10	0
6	F	512	GOL	3	0
5	C	507	FMT	2	0
5	F	511	FMT	1	0
6	B	528	GOL	2	0
5	C	511	FMT	1	0
5	C	516	FMT	2	0
5	C	522	FMT	1	0
6	C	529	GOL	1	0
2	F	501	HEM	7	0
5	B	508	FMT	2	0
4	F	503	TRS	1	0
3	F	502	QR8	1	0

Continued on next page...

CONFIDENTIAL VALIDATION REPORT

Continued from previous page...

Mol	Chain	Res	Type	Clashes	Symm-Clashes
2	B	501	HEM	4	0
6	B	530	GOL	1	0
6	B	527	GOL	2	0
4	D	503	TRS	8	0
2	C	501	HEM	1	0
6	C	527	GOL	1	0
5	A	512	FMT	1	0
2	A	501	HEM	5	0
5	F	506	FMT	1	0
6	B	529	GOL	1	0
3	D	502	QR8	1	0

The following is a two-dimensional graphical depiction of Mogul quality analysis of bond lengths, bond angles, torsion angles, and ring geometry for all instances of the Ligand of Interest. In addition, ligands with molecular weight > 250 and outliers as shown on the validation Tables will also be included. For torsion angles, if less then 5% of the Mogul distribution of torsion angles is within 10 degrees of the torsion angle in question, then that torsion angle is considered an outlier. Any bond that is central to one or more torsion angles identified as an outlier by Mogul will be highlighted in the graph. For rings, the root-mean-square deviation (RMSD) between the ring in question and similar rings identified by Mogul is calculated over all ring torsion angles. If the average RMSD is greater than 60 degrees and the minimal RMSD between the ring in question and any Mogul-identified rings is also greater than 60 degrees, then that ring is considered an outlier. The outliers are highlighted in purple. The color gray indicates Mogul did not find sufficient equivalents in the CSD to analyse the geometry.

CONFIDENTIAL VALIDATION REPORT

Ligand HEM E 501

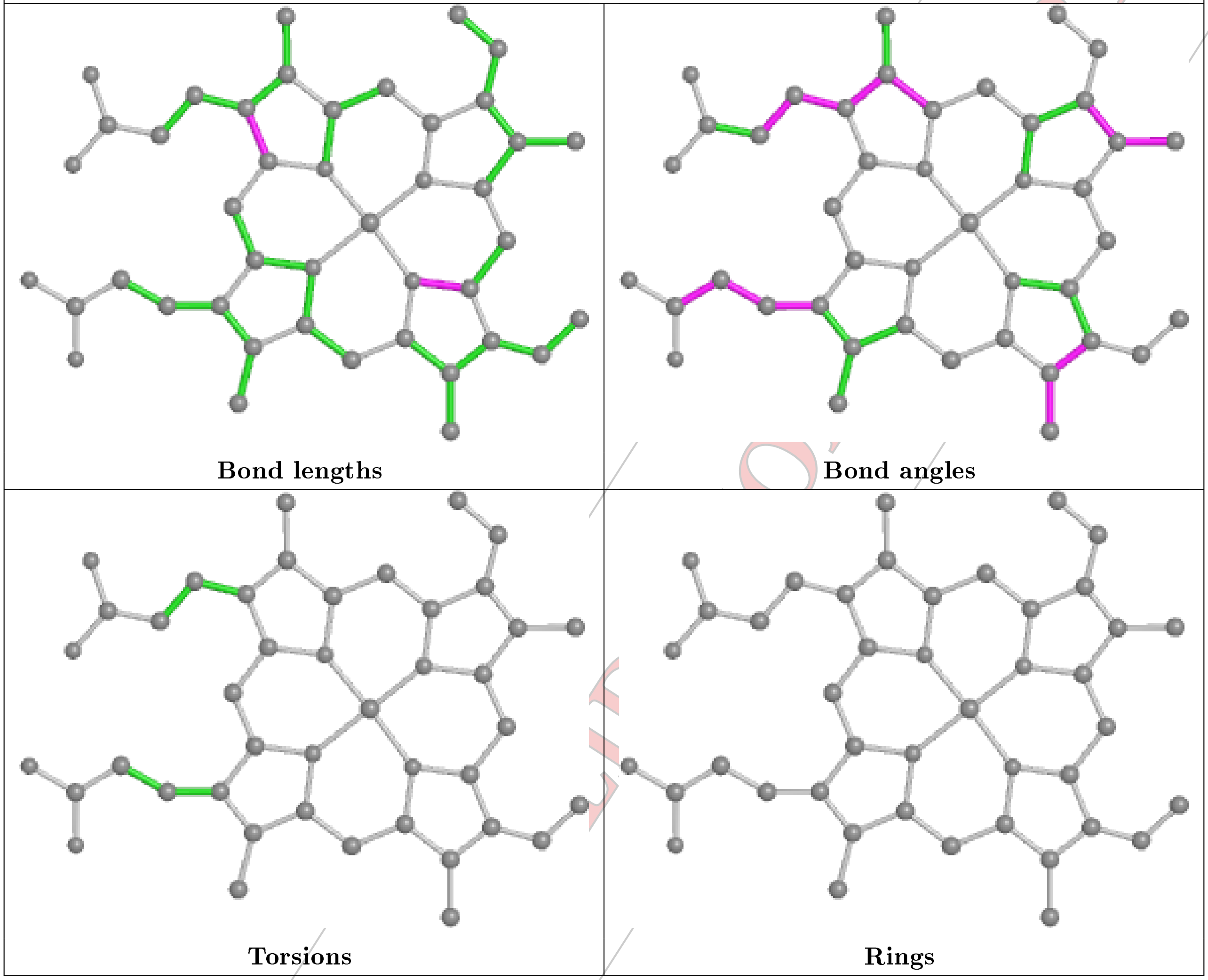

Bond lengths | Bond angles | Torsions | Rings

CONFIDENTIAL

Ligand HEM D 501

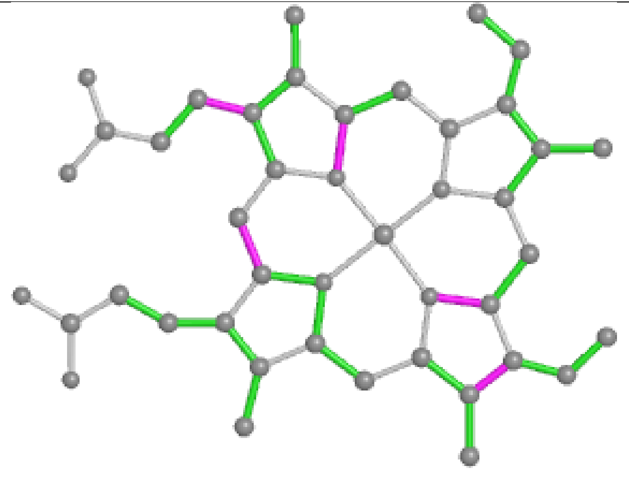

Bond lengths

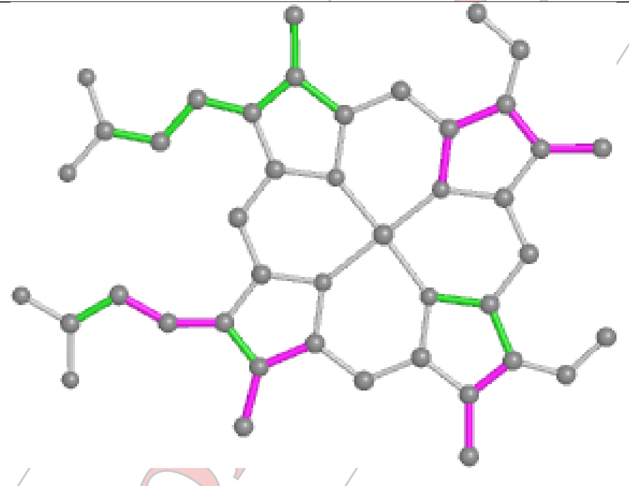

Bond angles

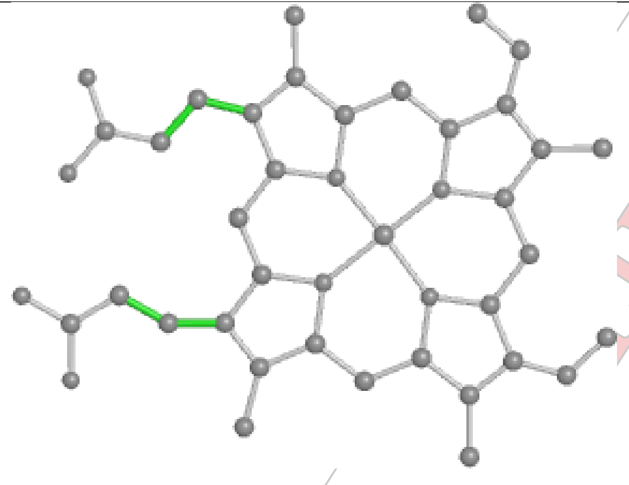

Torsions

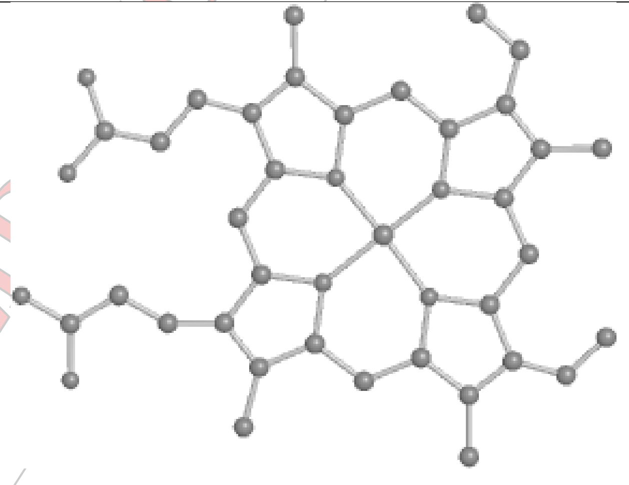

Rings

CONFIDENTIAL

Ligand QR8 B 502

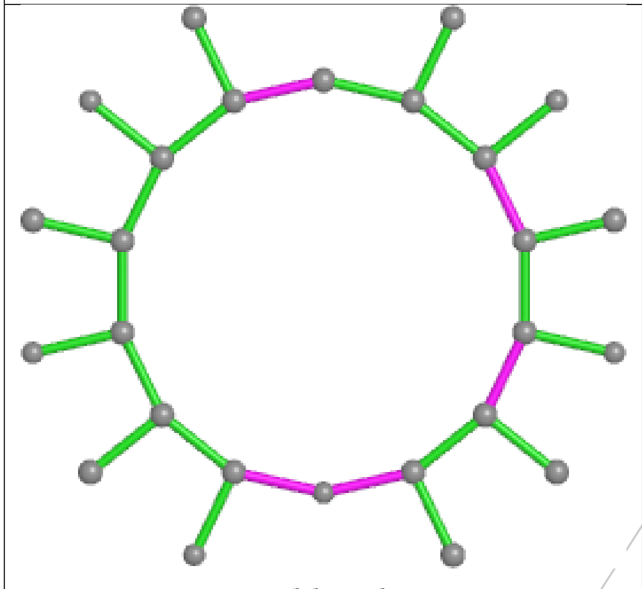

Bond lengths

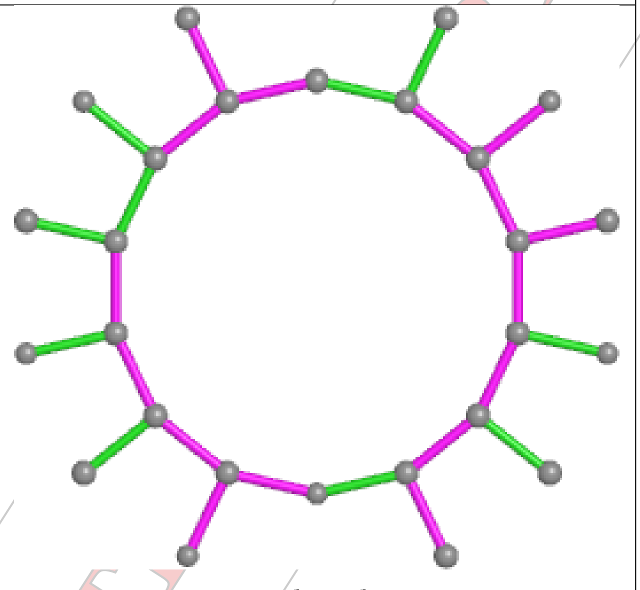

Bond angles

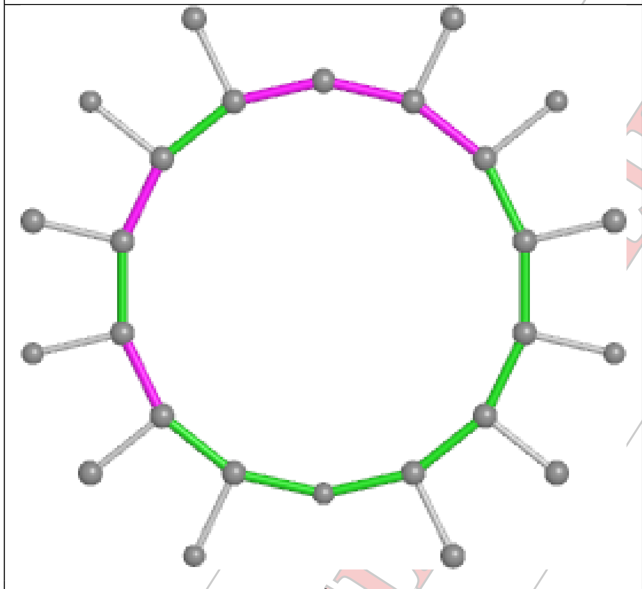

Torsions

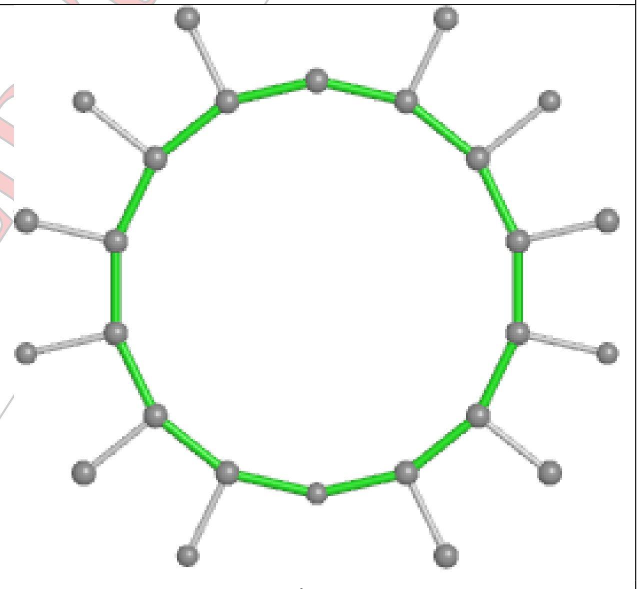

Rings

CONFIDENTIAL

Ligand QR8 A 502

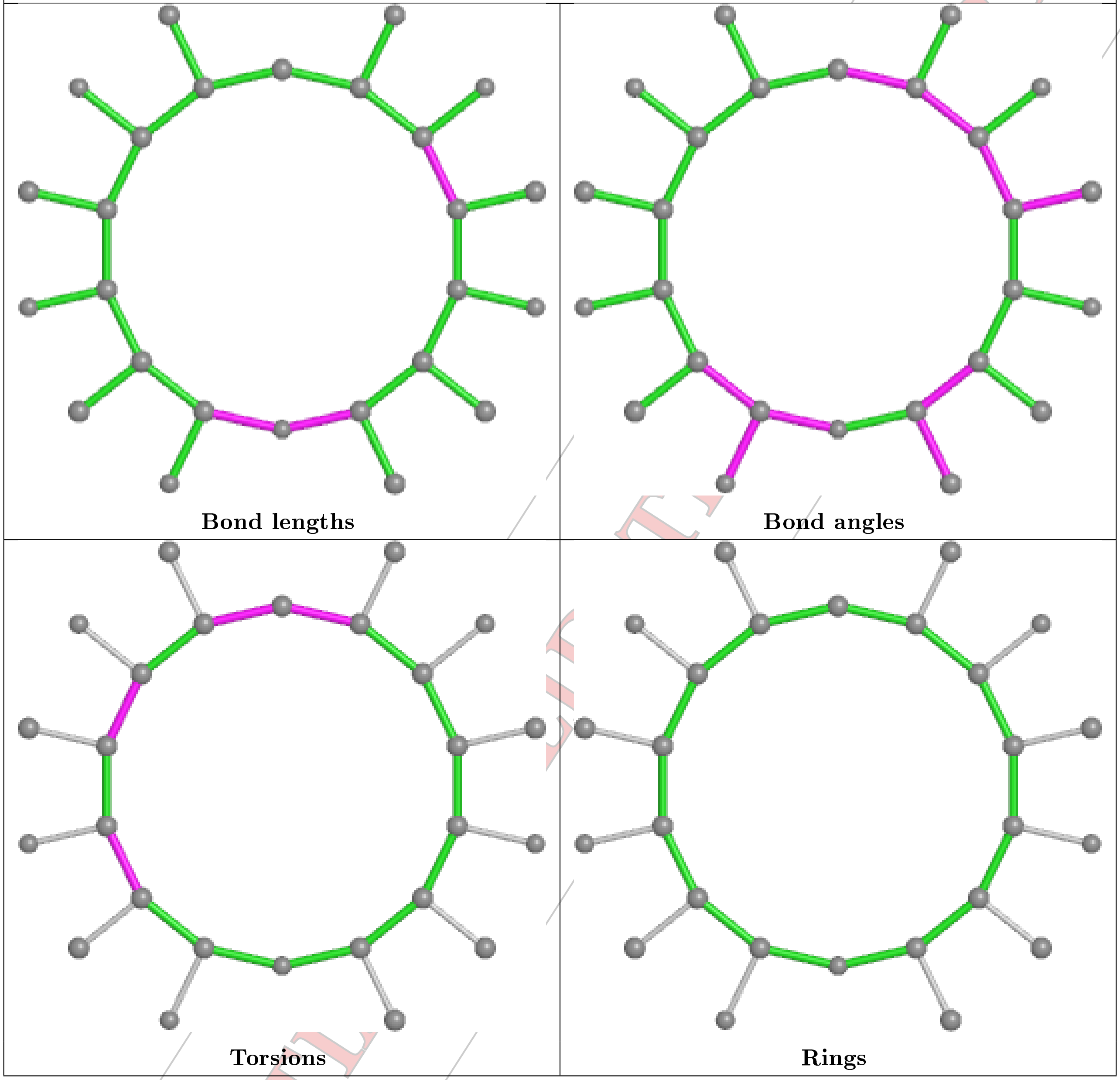

CONFIDENTIAL

Ligand HEM F 501

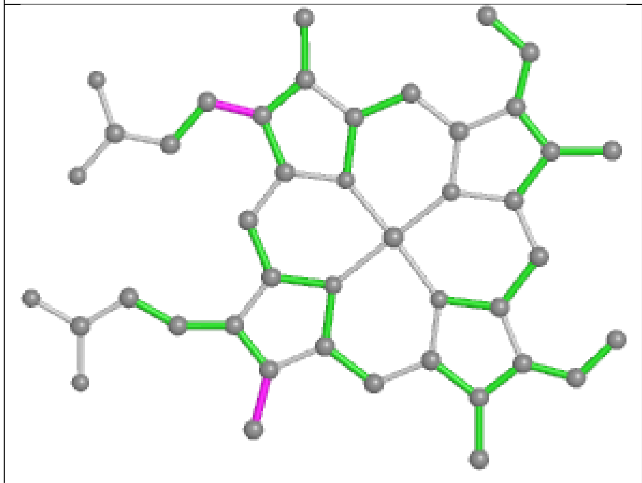

Bond lengths

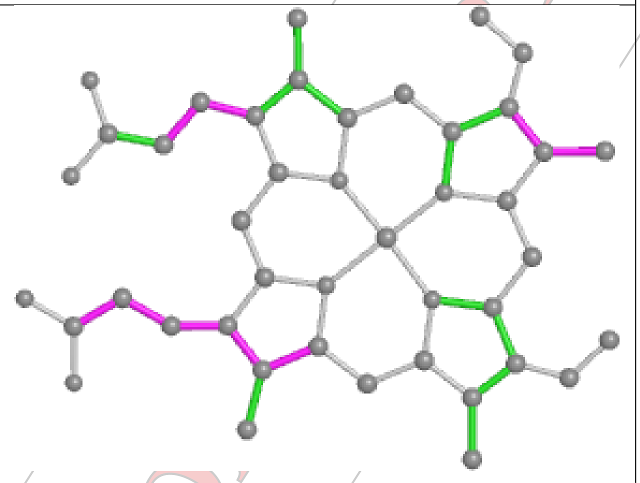

Bond angles

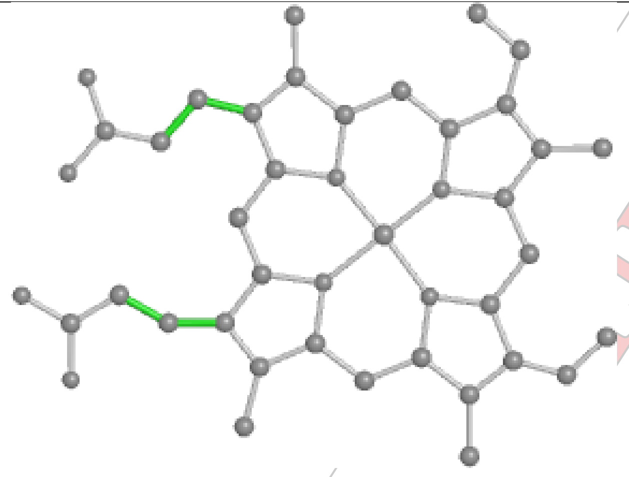

Torsions

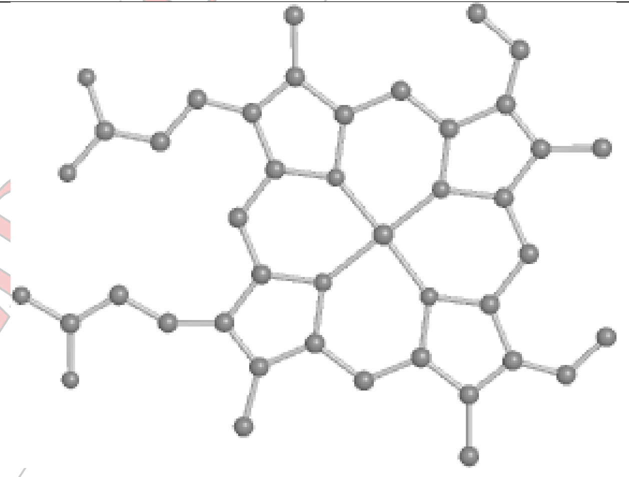

Rings

CONFIDENTIAL

Ligand QR8 C 502

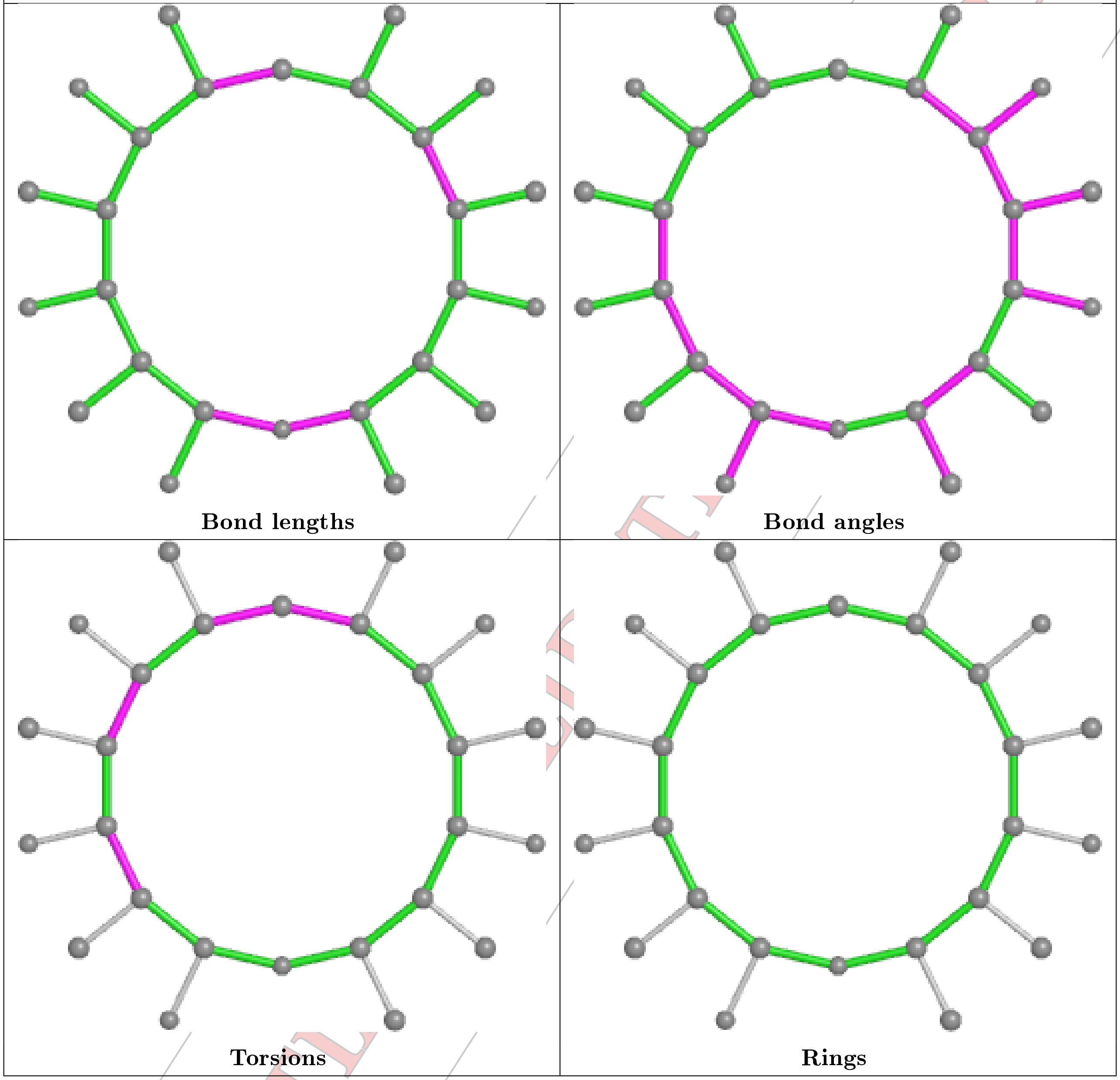

CONFIDENTIAL

Ligand QR8 F 502

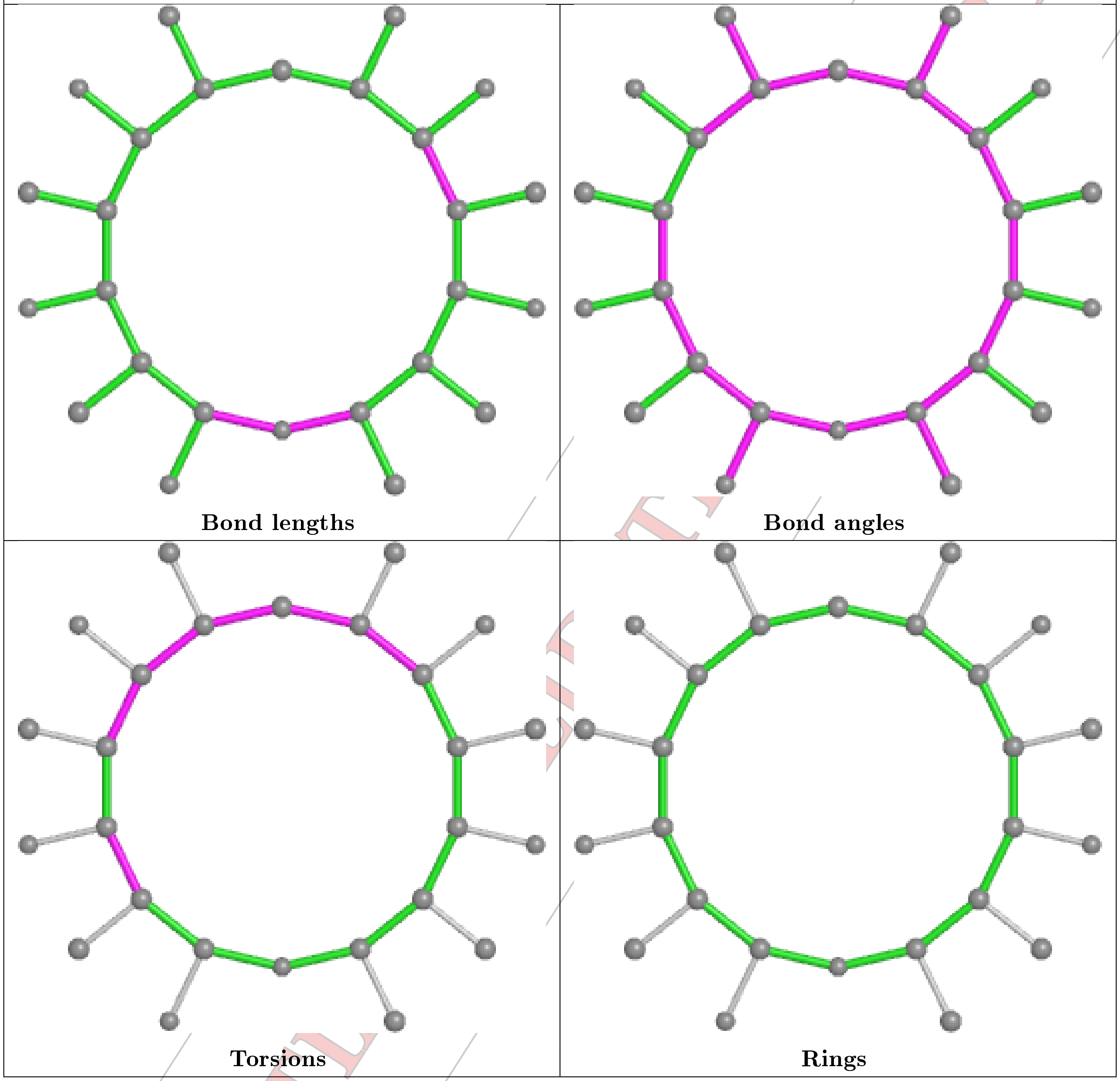

Ligand HEM B 501

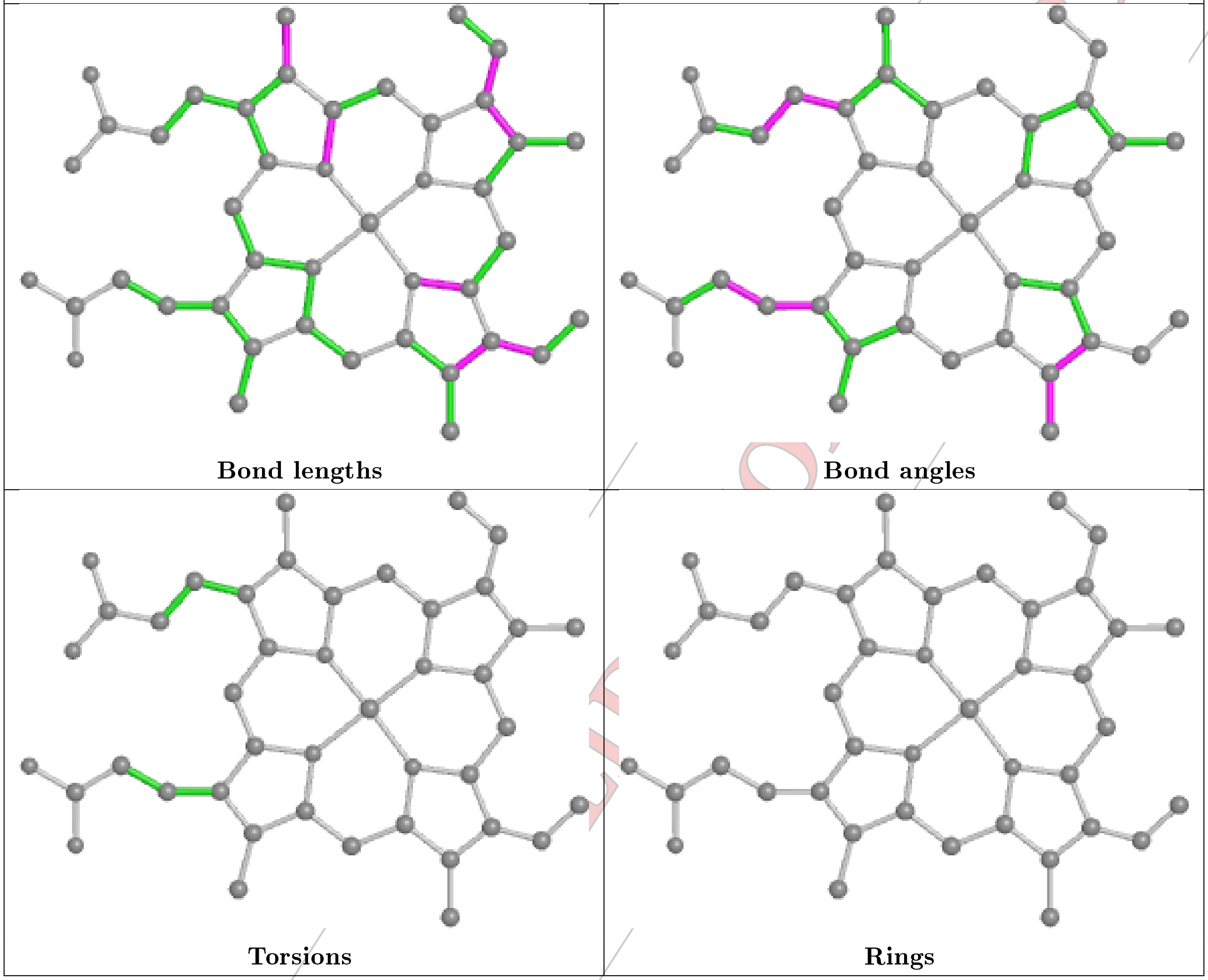

CONFIDENTIAL

Ligand HEM C 501

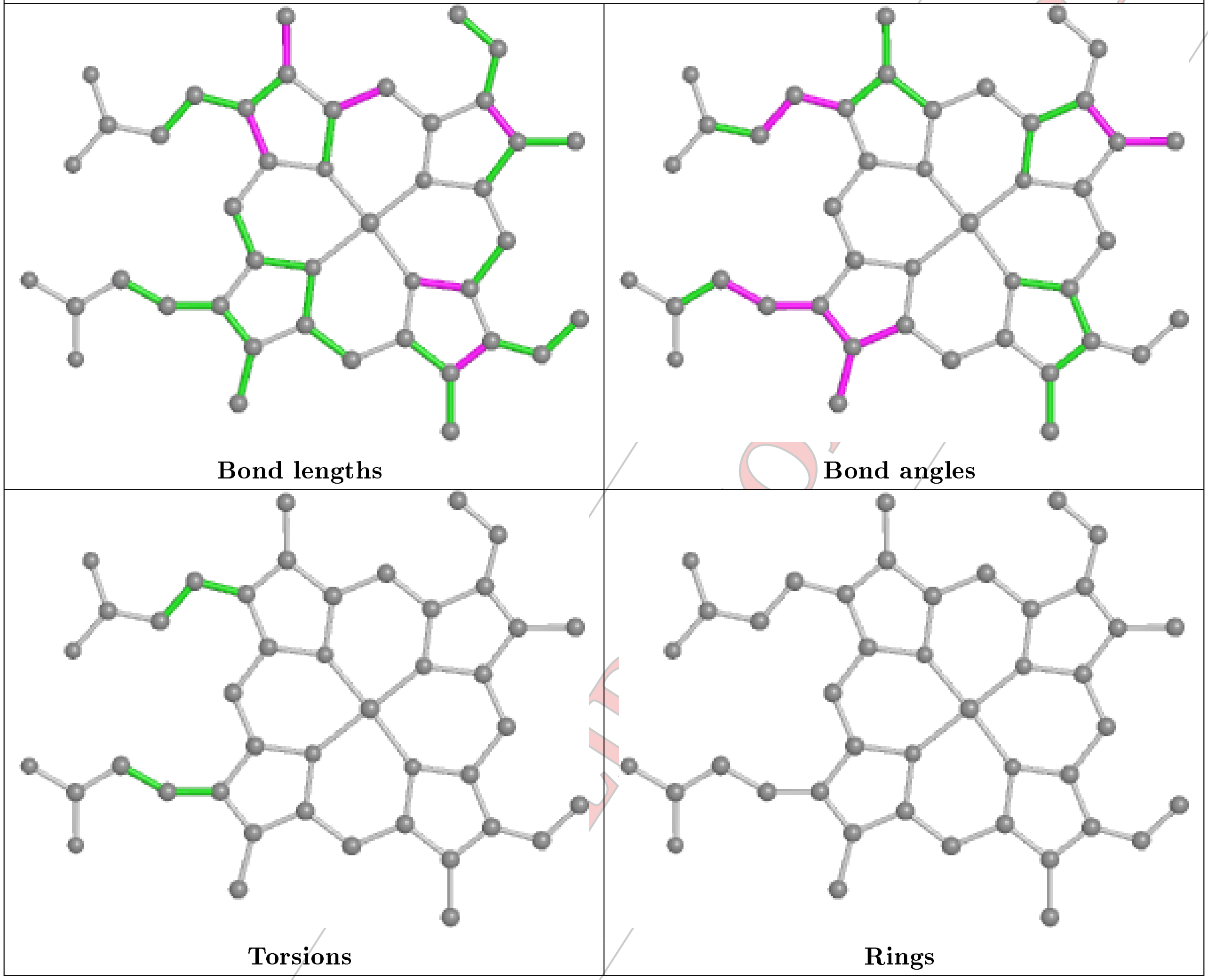

CONFIDENTIAL

Ligand HEM A 501

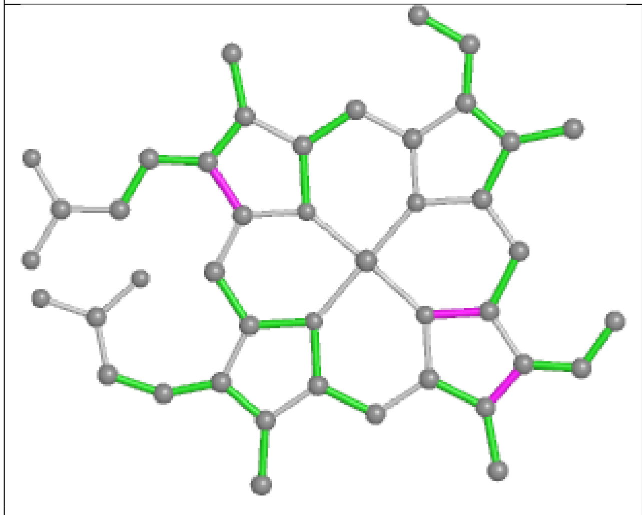

Bond lengths

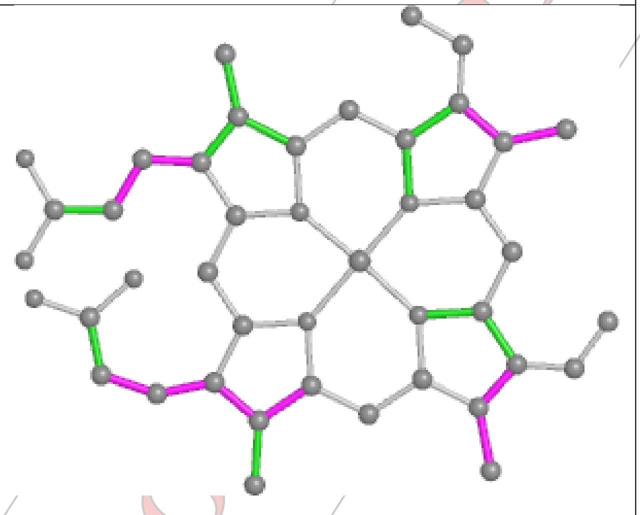

Bond angles

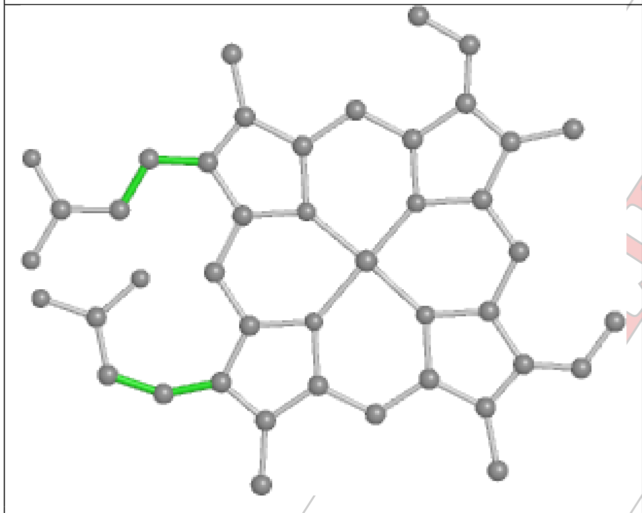

Torsions

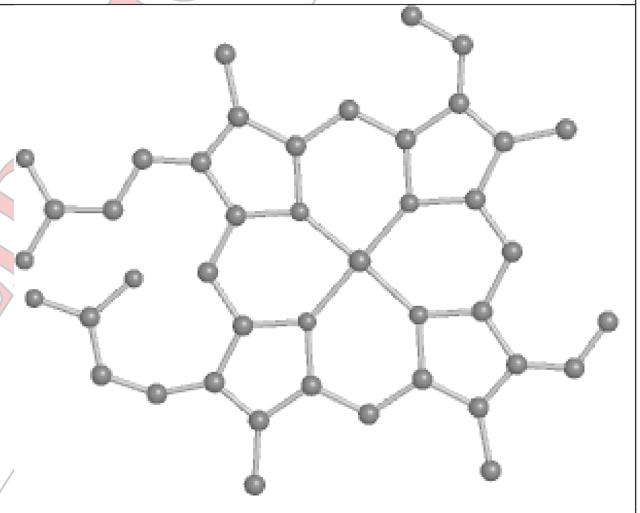

Rings

CONFIDENTIAL

Ligand QR8 D 502

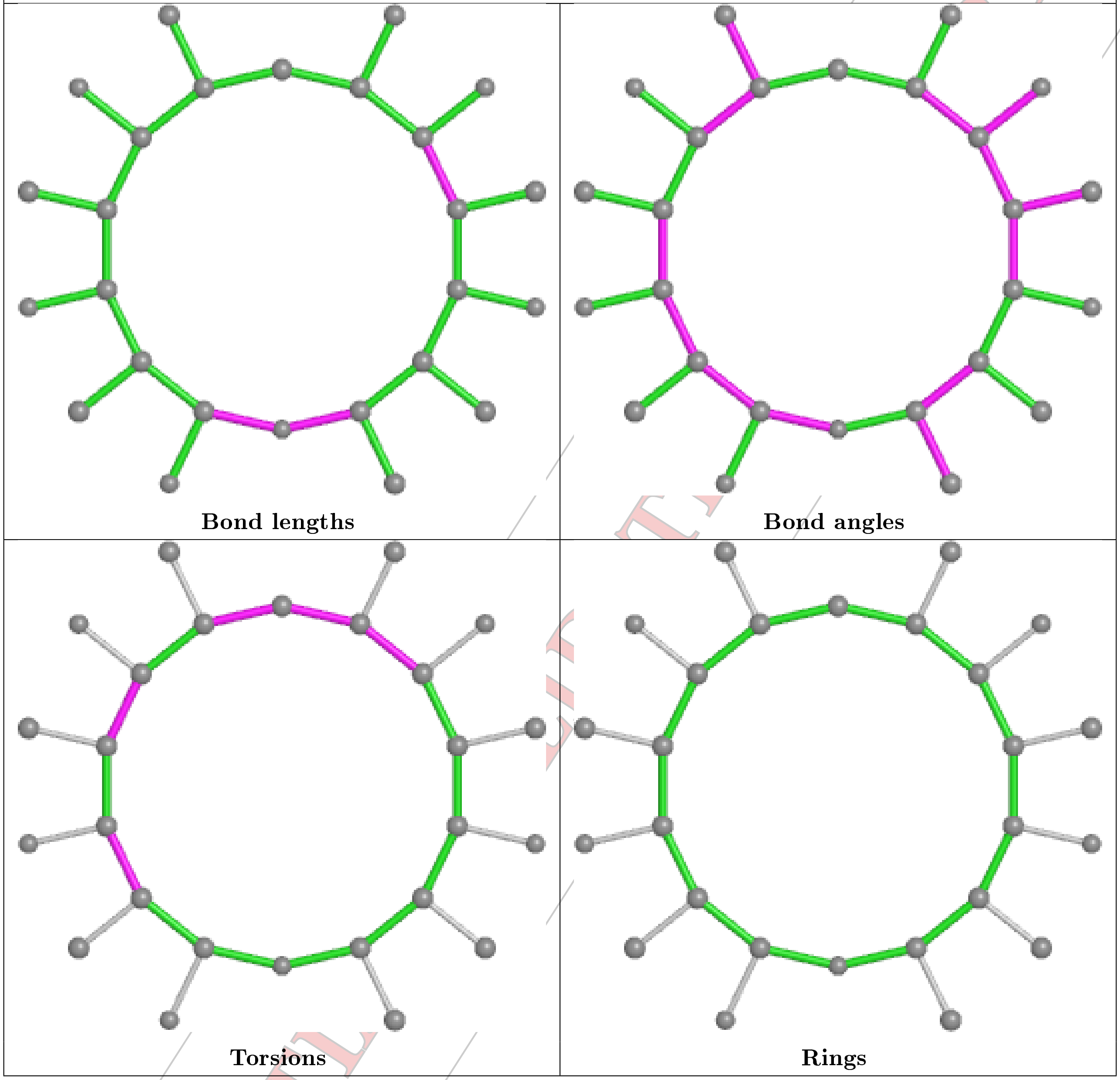

CONFIDENTIAL DRAFT

Ligand QR8 E 502

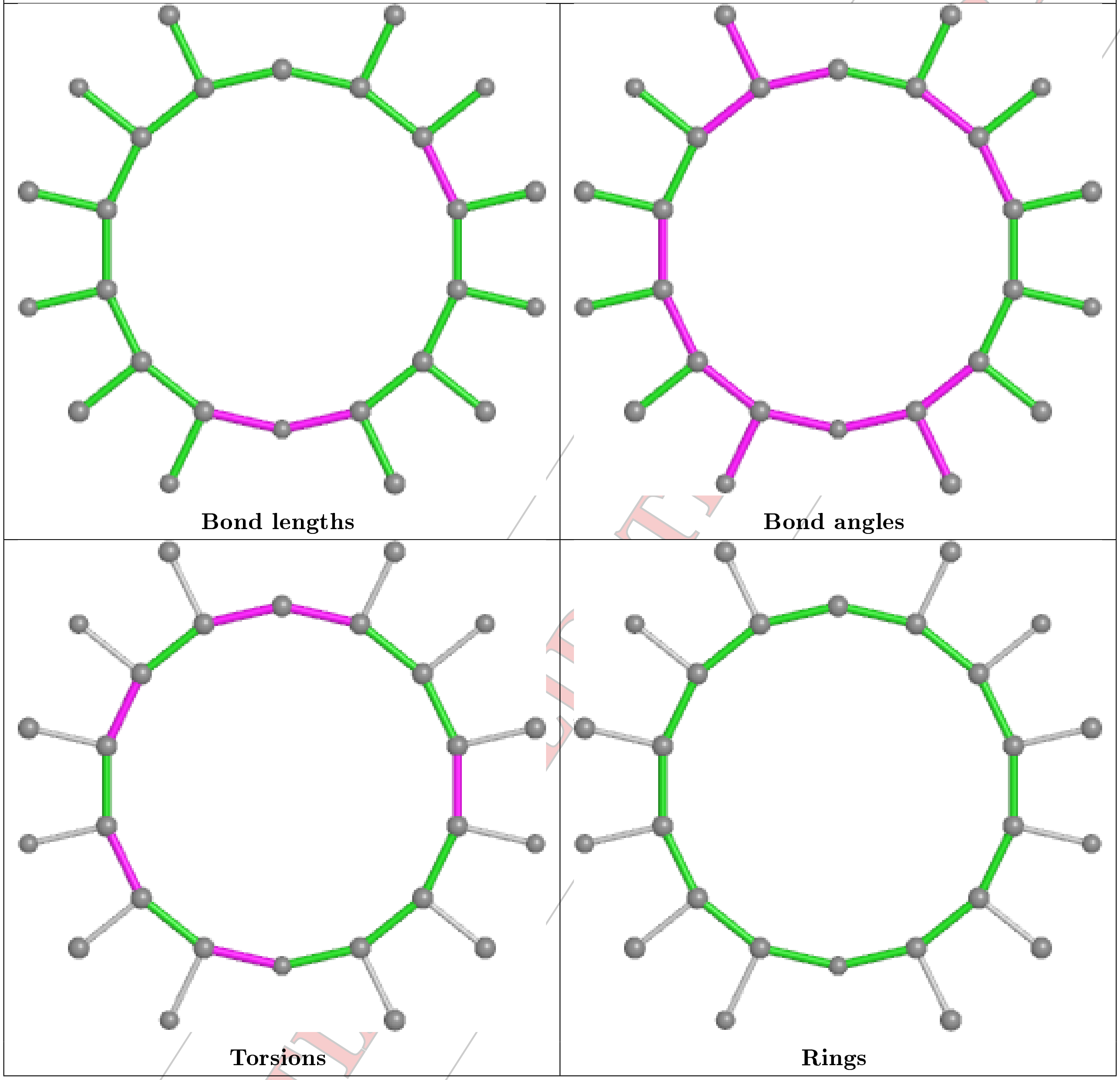

5.7 Other polymers (i)

There are no such residues in this entry.

5.8 Polymer linkage issues (i)

There are no chain breaks in this entry.

CONFIDENTIAL REPORT

6 Fit of model and data

6.1 Protein, DNA and RNA chains

In the following table, the column labelled '#RSRZ> 2' contains the number (and percentage) of RSRZ outliers, followed by percent RSRZ outliers for the chain as percentile scores relative to all X-ray entries and entries of similar resolution. The OWAB column contains the minimum, median, 95th percentile and maximum values of the occupancy-weighted average B-factor per residue. The column labelled 'Q< 0.9' lists the number of (and percentage) of residues with an average occupancy less than 0.9.

Mol	Chain	Analysed	<RSRZ>	#RSRZ>2			OWAB(Å^2)	Q<0.9
1	A	396/407 (97%)	-0.02	20 (5%)	28	26	35, 58, 87, 137	0
1	B	397/407 (97%)	-0.07	15 (3%)	40	38	32, 50, 74, 150	0
1	C	396/407 (97%)	-0.22	13 (3%)	46	44	31, 44, 64, 103	0
1	D	395/407 (97%)	0.19	30 (7%)	13	12	44, 69, 100, 147	1 (0%)
1	E	395/407 (97%)	0.43	39 (9%)	7	6	45, 70, 102, 154	0
1	F	395/407 (97%)	0.70	74 (18%)	1	1	49, 79, 124, 142	0
All	All	2374/2442 (97%)	0.17	191 (8%)	12	11	31, 62, 105, 154	1 (0%)

All (191) RSRZ outliers are listed below:

Mol	Chain	Res	Type	RSRZ
1	E	212	THR	7.6
1	E	209	ASP	7.2
1	E	343	ARG	6.4
1	E	210	ALA	6.1
1	E	42[A]	ARG	6.0
1	F	147	PHE	5.7
1	E	342[A]	GLU	5.7
1	B	211	PRO	5.4
1	E	332[A]	ASP	5.4
1	E	313	ALA	5.0
1	F	141	PRO	5.0
1	F	137	ALA	5.0
1	D	226	ASP	5.0
1	F	142	ALA	5.0
1	F	337[A]	LEU	4.9
1	A	210	ALA	4.9
1	F	261	LEU	4.8
1	F	407	TRP	4.7
1	E	13	ALA	4.7

Continued on next page...

CONFIDENTIAL VALIDATION REPORT

Continued from previous page...

Mol	Chain	Res	Type	RSRZ
1	F	379	THR	4.6
1	F	272	ALA	4.6
1	E	227	ASP	4.6
1	E	36	ARG	4.6
1	D	140	SER	4.5
1	F	380	LEU	4.5
1	F	343	ARG	4.5
1	F	37[A]	ASP	4.5
1	B	209	ASP	4.5
1	E	49[A]	GLU	4.5
1	F	386	VAL	4.4
1	D	210	ALA	4.3
1	F	138	HIS	4.2
1	F	136	VAL	4.2
1	E	305[A]	GLU	4.1
1	E	312[A]	ARG	4.0
1	E	226	ASP	4.0
1	F	328	GLU	4.0
1	F	405	VAL	4.0
1	D	209	ASP	3.9
1	F	36	ARG	3.9
1	F	140	SER	3.9
1	D	224	ASP	3.9
1	D	49	GLU	3.8
1	F	340	HIS	3.8
1	B	225	ASN	3.8
1	F	377	PHE	3.8
1	F	404	ILE	3.8
1	B	210	ALA	3.7
1	F	139	GLY	3.7
1	E	211	PRO	3.7
1	E	340	HIS	3.6
1	A	224	ASP	3.6
1	F	115	ARG	3.6
1	F	273	ASP	3.6
1	F	209	ASP	3.5
1	A	209	ASP	3.5
1	F	333	HIS	3.5
1	F	271	VAL	3.4
1	E	224	ASP	3.4
1	C	343	ARG	3.4
1	D	225	ASN	3.4

Continued on next page...

CONFIDENTIAL VALIDATION REPORT

Continued from previous page...

Mol	Chain	Res	Type	RSRZ
1	D	229	LEU	3.3
1	F	385	PRO	3.3
1	E	37	ASP	3.3
1	F	208	ARG	3.3
1	D	36	ARG	3.3
1	B	362	GLY	3.2
1	E	303	ASP	3.1
1	F	244	ALA	3.1
1	F	332	ASP	3.1
1	B	212	THR	3.1
1	F	49[A]	GLU	3.1
1	D	19	PHE	3.1
1	E	213	GLU	3.0
1	F	21	LEU	3.0
1	F	33	GLU	3.0
1	F	390	LYS	3.0
1	E	48	GLY	2.9
1	C	356	CYS	2.9
1	E	225	ASN	2.9
1	F	129	ASP	2.9
1	A	366	LEU	2.9
1	F	127	LEU	2.9
1	D	211	PRO	2.9
1	B	349	PHE	2.8
1	D	343	ARG	2.8
1	F	211	PRO	2.7
1	E	301	THR	2.7
1	F	29	PRO	2.7
1	C	291	VAL	2.7
1	F	262	THR	2.7
1	A	208	ARG	2.7
1	F	348	ALA	2.7
1	D	208	ARG	2.7
1	C	359	ALA	2.7
1	F	225	ASN	2.7
1	F	402	ARG	2.7
1	F	342	GLU	2.7
1	D	221	LEU	2.7
1	E	307	SER	2.6
1	A	225	ASN	2.6
1	E	14	VAL	2.6
1	D	227	ASP	2.6

Continued on next page...

CONFIDENTIAL VALIDATION REPORT

Continued from previous page...

Mol	Chain	Res	Type	RSRZ
1	B	366	LEU	2.6
1	D	342	GLU	2.6
1	F	248	THR	2.5
1	D	219	LEU	2.5
1	A	140	SER	2.5
1	B	224	ASP	2.5
1	E	30	HIS	2.5
1	A	379	THR	2.5
1	A	211	PRO	2.5
1	D	212	THR	2.5
1	E	285	LEU	2.5
1	C	349	PHE	2.5
1	F	336	GLU	2.5
1	B	249	SER	2.5
1	E	44[A]	ARG	2.5
1	F	329	GLU	2.5
1	F	143	ASP	2.5
1	D	332	ASP	2.4
1	A	36[A]	ARG	2.4
1	B	361	LEU	2.4
1	F	382	LEU	2.4
1	F	126	SER	2.4
1	A	356	CYS	2.4
1	C	361	LEU	2.4
1	C	362	GLY	2.4
1	D	333	HIS	2.4
1	F	339	PHE	2.4
1	F	264	ARG	2.4
1	C	357	ILE	2.4
1	F	50	GLY	2.3
1	F	388	GLY	2.3
1	D	385	PRO	2.3
1	D	249	SER	2.3
1	E	50	GLY	2.3
1	E	348	ALA	2.3
1	C	285	LEU	2.3
1	A	228	HIS	2.3
1	B	36	ARG	2.3
1	E	356	CYS	2.3
1	D	139	GLY	2.3
1	A	226	ASP	2.3
1	A	248	THR	2.3

Continued on next page...

CONFIDENTIAL VALIDATION REPORT

Continued from previous page...

Mol	Chain	Res	Type	RSRZ
1	B	252[A]	GLN	2.3
1	F	383	ALA	2.3
1	D	285	LEU	2.2
1	E	306	LEU	2.2
1	F	330	VAL	2.2
1	F	252	GLN	2.2
1	D	220	ALA	2.2
1	A	348	ALA	2.2
1	B	248	THR	2.2
1	F	387	ALA	2.2
1	C	358	GLY	2.2
1	E	329[A]	GLU	2.2
1	F	215	LEU	2.2
1	F	20	SER	2.2
1	F	349	PHE	2.2
1	F	265	LYS	2.2
1	C	364	LEU	2.2
1	F	205	ALA	2.2
1	F	356	CYS	2.2
1	A	362	GLY	2.2
1	A	136	VAL	2.1
1	D	383	ALA	2.1
1	D	329	GLU	2.1
1	F	245	GLY	2.1
1	F	94	LEU	2.1
1	E	57[A]	ARG	2.1
1	E	388	GLY	2.1
1	A	342	GLU	2.1
1	F	130	SER	2.1
1	F	270	LEU	2.1
1	F	366	LEU	2.1
1	F	30	HIS	2.1
1	D	33	GLU	2.1
1	F	123	ARG	2.1
1	D	359	ALA	2.1
1	E	223	THR	2.1
1	E	33	GLU	2.1
1	F	210	ALA	2.1
1	B	356	CYS	2.0
1	A	221	LEU	2.0
1	A	141	PRO	2.0
1	D	250	VAL	2.0

Continued on next page...

CONFIDENTIAL VALIDATION REPORT

Continued from previous page...

Mol	Chain	Res	Type	RSRZ
1	F	250	VAL	2.0
1	C	225	ASN	2.0
1	C	290	LEU	2.0
1	E	256	LEU	2.0

6.2 Non-standard residues in protein, DNA, RNA chains (i)

There are no non-standard protein/DNA/RNA residues in this entry.

6.3 Carbohydrates (i)

There are no monosaccharides in this entry.

6.4 Ligands (i)

In the following table, the Atoms column lists the number of modelled atoms in the group and the number defined in the chemical component dictionary. The B-factors column lists the minimum, median, 95th percentile and maximum values of B factors of atoms in the group. The column labelled 'Q< 0.9' lists the number of atoms with occupancy less than 0.9.

Mol	Type	Chain	Res	Atoms	RSCC	RSR	B-factors(Å^2)	Q<0.9
5	FMT	A	515	3/3	0.07	0.45	98,98,101,106	0
5	FMT	E	509	3/3	0.41	0.35	96,96,100,105	0
5	FMT	F	507	3/3	0.46	0.67	91,91,95,101	0
5	FMT	C	518	3/3	0.52	0.26	93,93,94,94	0
5	FMT	C	505	3/3	0.52	0.43	113,113,113,113	0
5	FMT	B	514	3/3	0.55	0.36	97,97,107,107	0
5	FMT	C	509	3/3	0.56	0.35	85,85,89,96	0
5	FMT	E	506	3/3	0.58	0.18	85,85,89,96	0
5	FMT	B	525	3/3	0.59	0.69	92,92,98,102	0
5	FMT	A	504	3/3	0.62	0.37	78,78,89,90	0
5	FMT	E	504	3/3	0.62	0.51	91,91,97,99	0
5	FMT	A	514	3/3	0.63	0.34	87,87,88,91	0
5	FMT	B	505	3/3	0.65	0.22	94,94,101,102	0
5	FMT	E	505	3/3	0.66	0.30	89,89,97,97	0
5	FMT	A	516	3/3	0.67	0.25	83,83,88,99	0
5	FMT	D	507	3/3	0.67	0.42	87,87,90,91	0
5	FMT	D	505	3/3	0.67	0.40	99,99,109,111	0
6	GOL	B	531	6/6	0.67	0.27	82,99,102,104	0
5	FMT	C	513	3/3	0.68	0.23	78,78,78,93	0

Continued on next page...

CONFIDENTIAL VALIDATION REPORT

Continued from previous page...

Mol	Type	Chain	Res	Atoms	RSCC	RSR	B-factors(Å^2)	Q<0.9
5	FMT	C	506	3/3	0.69	0.39	75,75,76,84	0
6	GOL	B	532	6/6	0.69	0.24	87,98,100,102	0
5	FMT	D	511	3/3	0.69	0.38	82,82,94,94	0
5	FMT	E	510	3/3	0.69	0.50	98,98,105,113	0
5	FMT	C	516	3/3	0.71	0.29	41,41,42,44	3
5	FMT	D	506	3/3	0.72	0.26	85,85,91,95	0
6	GOL	B	528	6/6	0.72	0.25	88,95,102,104	0
5	FMT	D	509	3/3	0.73	0.27	78,78,81,86	0
5	FMT	D	508	3/3	0.73	0.50	108,108,113,114	0
5	FMT	A	505	3/3	0.73	0.47	85,85,92,95	0
5	FMT	F	509	3/3	0.74	0.19	90,90,107,107	0
5	FMT	C	514	3/3	0.74	0.18	100,100,103,104	0
5	FMT	B	522	3/3	0.74	0.45	92,92,97,98	0
5	FMT	B	518	3/3	0.75	0.31	85,85,86,91	0
5	FMT	B	515	3/3	0.75	0.24	76,76,82,84	0
5	FMT	D	510	3/3	0.75	0.32	93,93,95,99	0
5	FMT	C	515	3/3	0.75	0.32	92,92,93,97	0
5	FMT	A	513	3/3	0.76	0.31	103,103,106,108	0
5	FMT	C	510	3/3	0.77	0.26	83,83,90,94	0
5	FMT	A	511	3/3	0.77	0.57	95,95,95,96	0
5	FMT	F	510	3/3	0.77	0.34	79,79,83,86	0
5	FMT	B	523	3/3	0.79	0.27	61,61,73,78	0
5	FMT	C	519	3/3	0.79	0.22	88,88,92,93	0
6	GOL	B	527	6/6	0.80	0.25	82,88,98,105	0
5	FMT	B	519	3/3	0.80	0.29	89,89,92,100	0
5	FMT	C	504	3/3	0.80	0.34	64,64,70,74	0
5	FMT	C	523	3/3	0.81	0.37	80,80,83,87	0
5	FMT	F	505	3/3	0.81	0.16	86,86,93,99	0
5	FMT	A	509	3/3	0.81	0.28	72,72,73,74	0
5	FMT	C	503	3/3	0.81	0.52	94,94,98,101	0
5	FMT	B	507	3/3	0.81	0.35	90,90,97,100	0
5	FMT	A	510	3/3	0.81	0.12	95,95,99,100	0
5	FMT	B	504	3/3	0.81	0.26	74,74,74,78	0
5	FMT	F	504	3/3	0.82	0.26	79,79,82,92	0
5	FMT	B	524	3/3	0.83	0.57	99,99,102,102	0
5	FMT	B	526	3/3	0.83	0.29	79,79,87,90	0
5	FMT	A	507	3/3	0.83	0.11	88,88,97,101	0
5	FMT	B	511	3/3	0.83	0.33	78,78,86,92	0
5	FMT	B	521	3/3	0.83	0.42	92,92,94,97	0
5	FMT	C	520	3/3	0.83	0.15	70,70,80,85	0
5	FMT	B	517	3/3	0.84	0.19	73,73,75,80	0
6	GOL	C	528	6/6	0.84	0.20	80,89,92,94	0

Continued on next page...

CONFIDENTIAL VALIDATION REPORT

Continued from previous page...

Mol	Type	Chain	Res	Atoms	RSCC	RSR	B-factors(Å^2)	Q<0.9
5	FMT	C	517	3/3	0.84	0.34	62,62,77,85	0
5	FMT	E	508	3/3	0.84	0.20	78,78,89,94	0
5	FMT	B	520	3/3	0.85	0.22	80,80,82,92	0
5	FMT	F	508	3/3	0.85	0.44	104,104,109,113	0
6	GOL	C	529	6/6	0.85	0.17	65,76,82,84	0
5	FMT	B	513	3/3	0.85	0.37	85,85,94,94	0
5	FMT	A	512	3/3	0.86	0.18	94,94,99,99	0
6	GOL	F	512	6/6	0.86	0.15	71,75,82,88	0
5	FMT	B	503	3/3	0.86	0.30	74,74,76,80	0
5	FMT	C	521	3/3	0.87	0.21	66,66,72,84	0
5	FMT	F	506	3/3	0.87	0.15	65,65,77,79	0
4	TRS	A	503	8/8	0.87	0.24	88,90,95,103	0
7	NA	F	513	1/1	0.88	0.13	64,64,64,64	0
5	FMT	B	510	3/3	0.89	0.17	71,71,73,82	0
5	FMT	A	517	3/3	0.89	0.14	69,69,80,82	0
5	FMT	C	512	3/3	0.89	0.31	72,72,85,86	0
4	TRS	F	503	8/8	0.89	0.18	63,71,75,79	0
5	FMT	C	522	3/3	0.89	0.17	70,70,82,91	0
6	GOL	B	530	6/6	0.89	0.14	76,87,92,107	0
5	FMT	B	516	3/3	0.90	0.15	66,66,78,85	0
5	FMT	A	506	3/3	0.90	0.13	79,79,82,82	0
6	GOL	C	526	6/6	0.90	0.18	42,50,62,64	0
6	GOL	C	527	6/6	0.90	0.14	67,73,75,77	0
5	FMT	E	503	3/3	0.90	0.21	85,85,90,95	0
5	FMT	B	506	3/3	0.91	0.21	81,81,96,100	0
6	GOL	D	512	6/6	0.91	0.19	90,93,95,100	0
4	TRS	D	503	8/8	0.92	0.19	76,82,84,86	0
5	FMT	E	507	3/3	0.92	0.17	73,73,79,87	0
5	FMT	F	511	3/3	0.92	0.17	90,90,95,95	0
7	NA	C	530	1/1	0.93	0.15	51,51,51,51	0
5	FMT	B	512	3/3	0.93	0.08	74,74,80,81	0
6	GOL	B	529	6/6	0.93	0.16	52,75,82,84	0
5	FMT	C	511	3/3	0.93	0.28	72,72,77,78	0
3	QR8	E	502	26/26	0.93	0.22	55,61,68,69	0
3	QR8	F	502	26/26	0.94	0.23	65,76,79,82	0
5	FMT	B	509	3/3	0.94	0.33	70,70,71,73	0
6	GOL	A	518	6/6	0.94	0.16	56,75,81,95	0
5	FMT	C	508	3/3	0.94	0.12	68,68,72,73	0
3	QR8	C	502	26/26	0.94	0.20	38,45,53,59	0
5	FMT	C	507	3/3	0.94	0.20	55,55,73,81	0
3	QR8	B	502	26/26	0.95	0.22	42,48,54,59	0
3	QR8	D	502	26/26	0.95	0.15	55,63,70,73	0

Continued on next page...

CONFIDENTIAL VALIDATION REPORT

Continued from previous page...

Mol	Type	Chain	Res	Atoms	RSCC	RSR	B-factors(Å^2)	Q<0.9
5	FMT	D	504	3/3	0.95	0.21	63,63,70,78	0
7	NA	A	519	1/1	0.96	0.28	55,55,55,55	0
7	NA	D	513	1/1	0.96	0.39	67,67,67,67	0
7	NA	B	533	1/1	0.96	0.09	52,52,52,52	0
6	GOL	C	525	6/6	0.96	0.18	68,76,80,83	0
3	QR8	A	502	26/26	0.96	0.21	47,52,60,66	0
2	HEM	F	501	43/43	0.97	0.20	59,65,76,86	0
6	GOL	C	524	6/6	0.97	0.11	50,58,61,62	0
5	FMT	B	508	3/3	0.97	0.13	56,56,56,60	0
2	HEM	E	501	43/43	0.98	0.21	44,49,56,63	0
2	HEM	D	501	43/43	0.98	0.19	41,45,54,59	0
2	HEM	C	501	43/43	0.99	0.21	28,32,38,40	0
2	HEM	B	501	43/43	0.99	0.22	30,33,39,44	0
5	FMT	A	508	3/3	0.99	0.10	52,52,54,54	0
2	HEM	A	501	43/43	0.99	0.19	32,37,42,47	0

The following is a graphical depiction of the model fit to experimental electron density of all instances of the Ligand of Interest. In addition, ligands with molecular weight > 250 and outliers as shown on the geometry validation Tables will also be included. Each fit is shown from different orientation to approximate a three-dimensional view.

CONFIDENTIAL VALIDATION REPORT

Electron density around QR8 E 502:

$2mF_o$-DF_c (at 0.7 rmsd) in gray
mF_o-DF_c (at 3 rmsd) in purple (negative)
and green (positive)

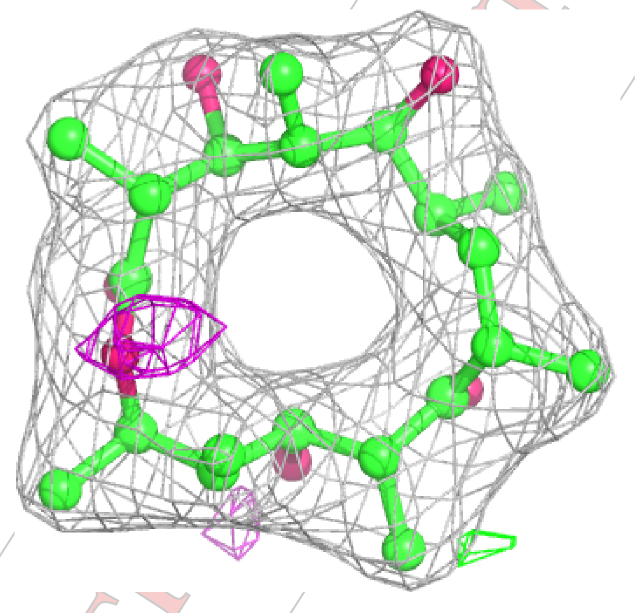

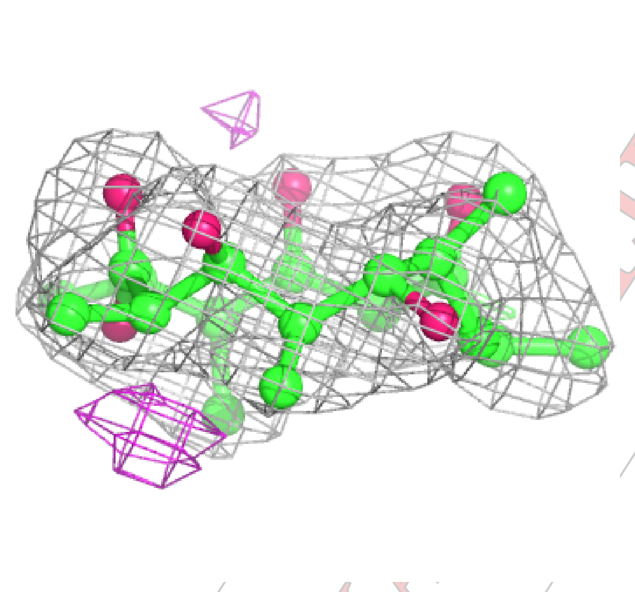

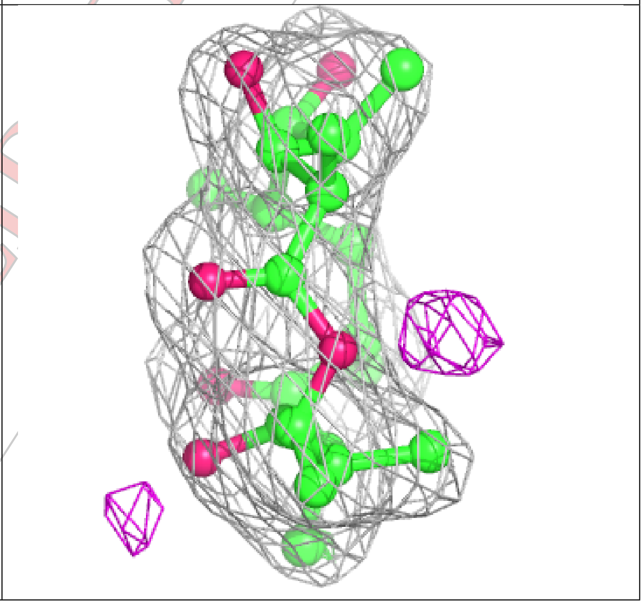

CONFIDENTIAL

Electron density around QR8 F 502:

$2mF_o$-DF_c (at 0.7 rmsd) in gray
mF_o-DF_c (at 3 rmsd) in purple (negative)
and green (positive)

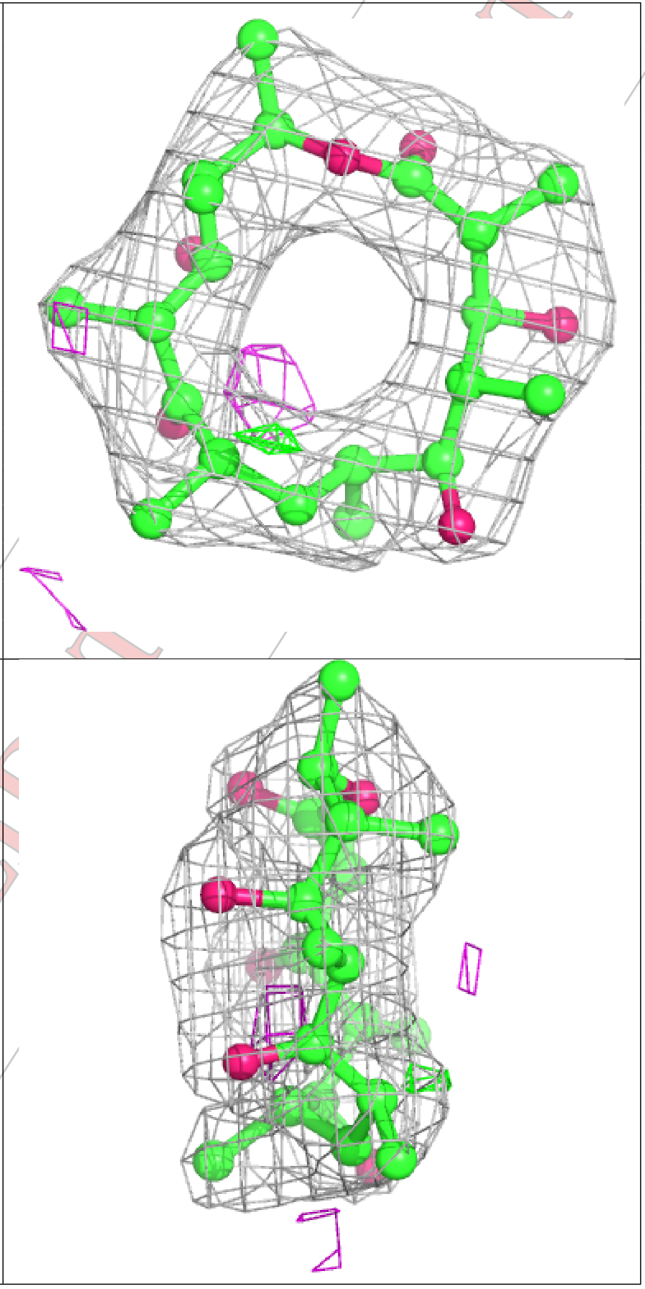

CONFIDENTIAL

Electron density around QR8 C 502:

$2mF_o$-DF_c (at 0.7 rmsd) in gray
mF_o-DF_c (at 3 rmsd) in purple (negative)
and green (positive)

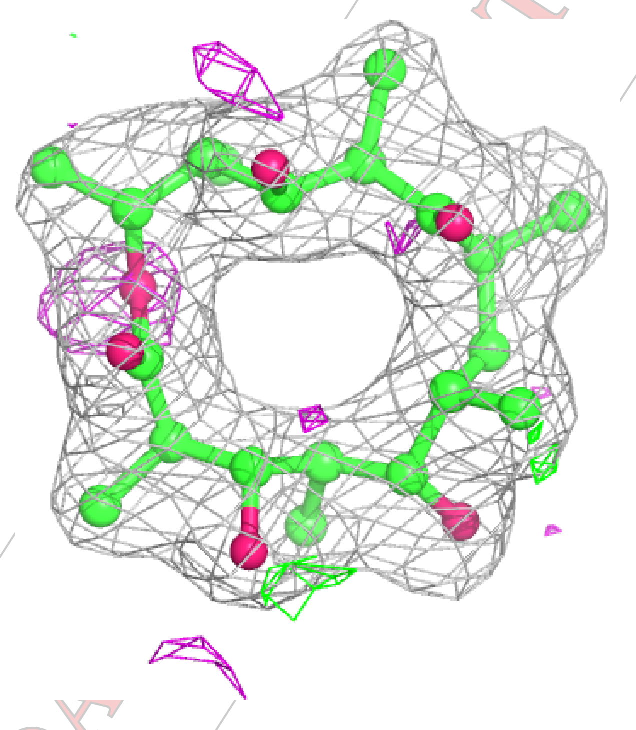

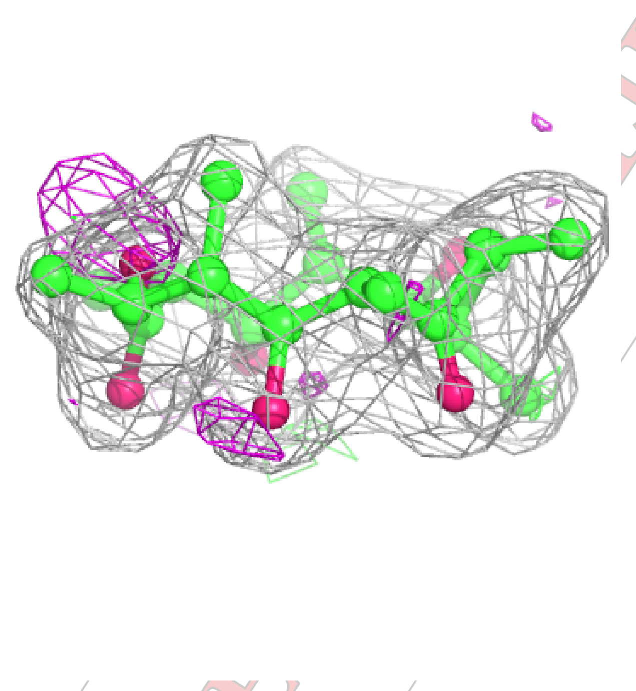

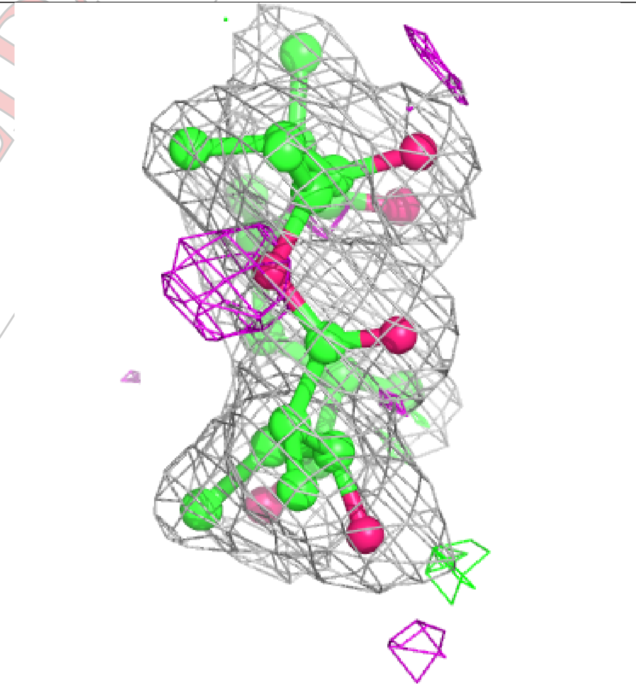

Electron density around QR8 B 502:

2mF$_o$-DF$_c$ (at 0.7 rmsd) in gray
mF$_o$-DF$_c$ (at 3 rmsd) in purple (negative)
and green (positive)

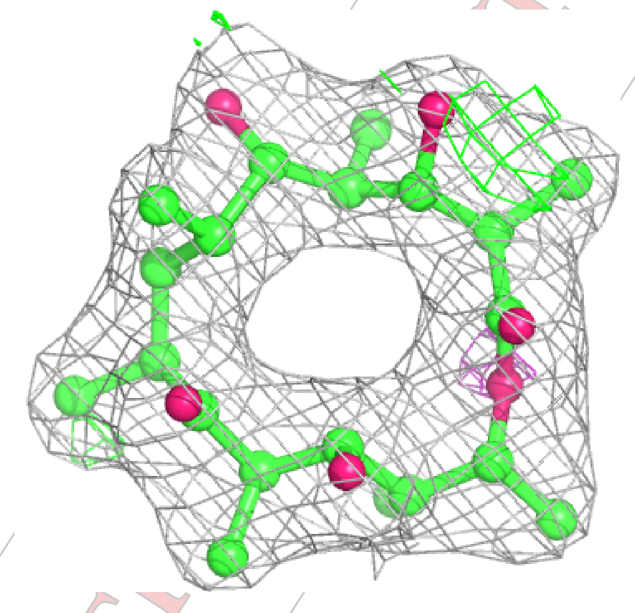

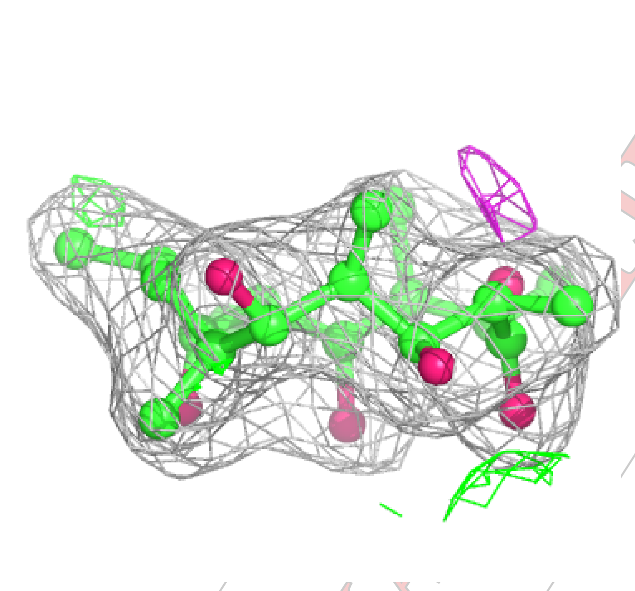

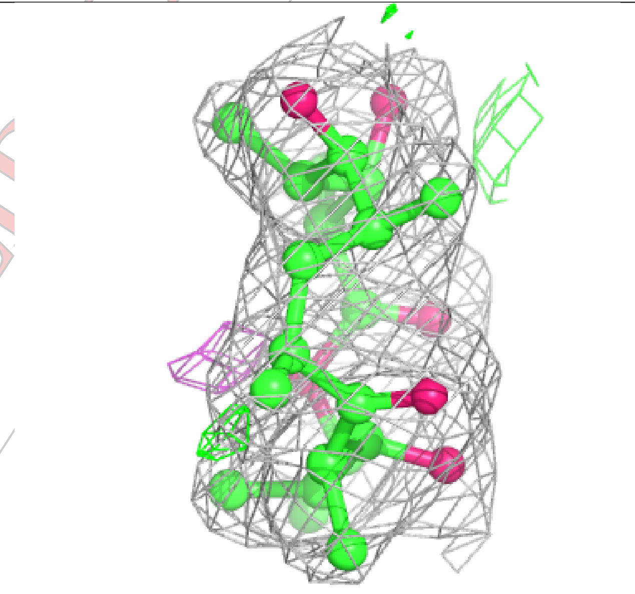

CONFIDENTIAL

Electron density around QR8 D 502:

2mF$_o$-DF$_c$ (at 0.7 rmsd) in gray
mF$_o$-DF$_c$ (at 3 rmsd) in purple (negative)
and green (positive)

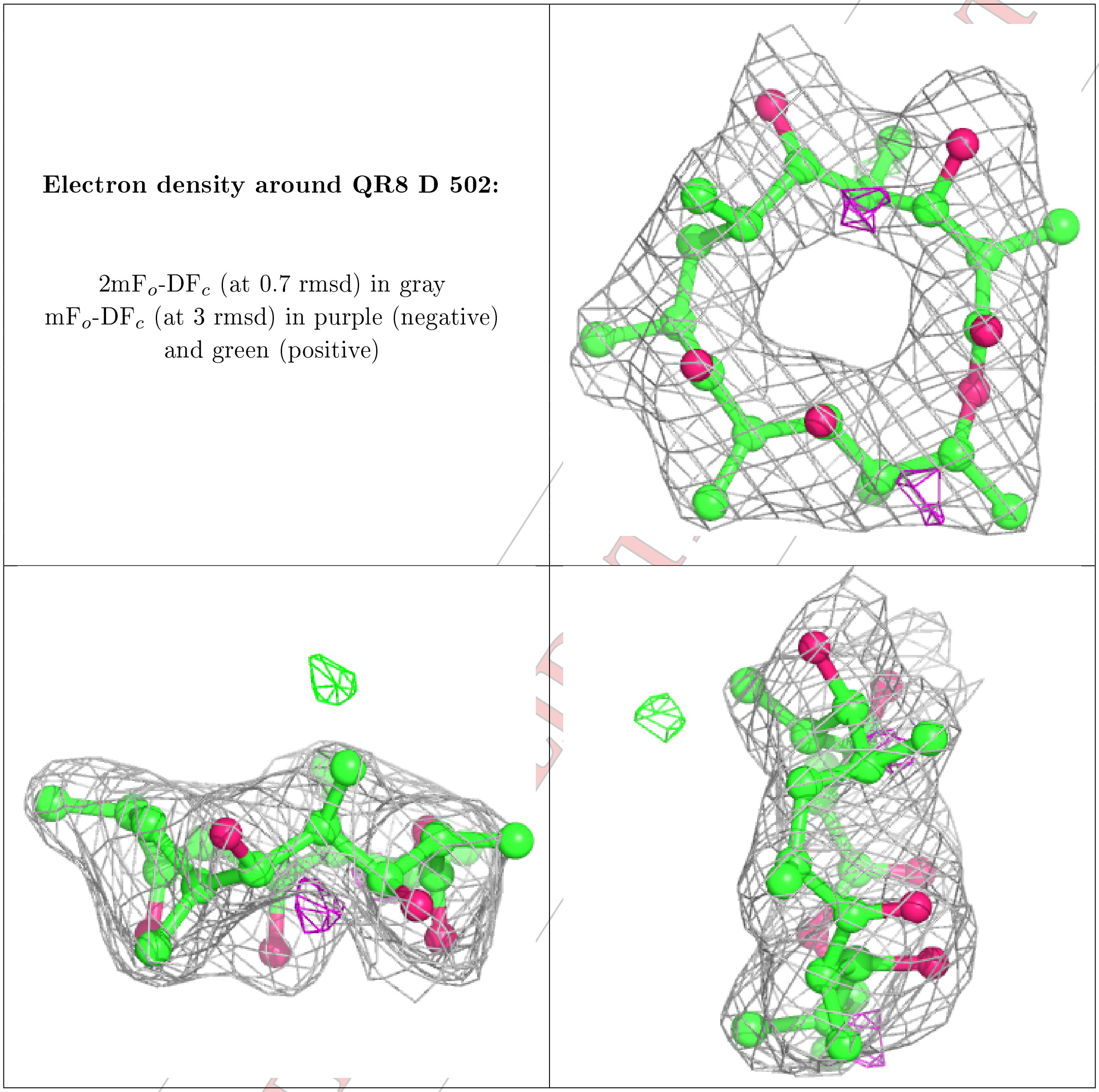

CONFIDENTIAL

Electron density around QR8 A 502:

$2mF_o$-DF_c (at 0.7 rmsd) in gray
mF_o-DF_c (at 3 rmsd) in purple (negative)
and green (positive)

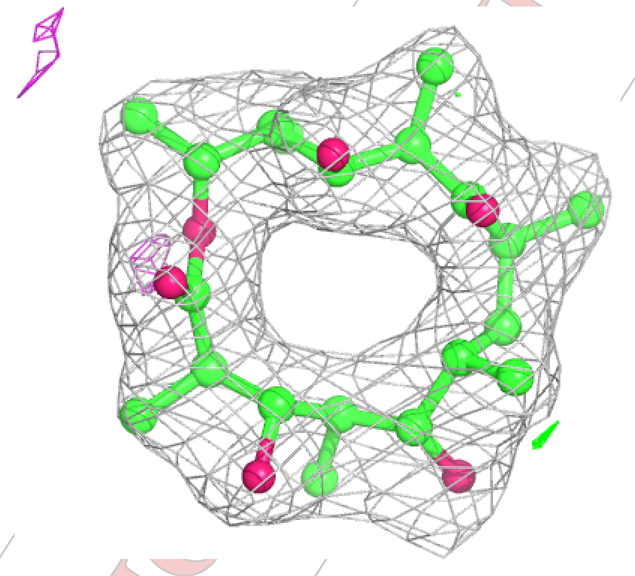

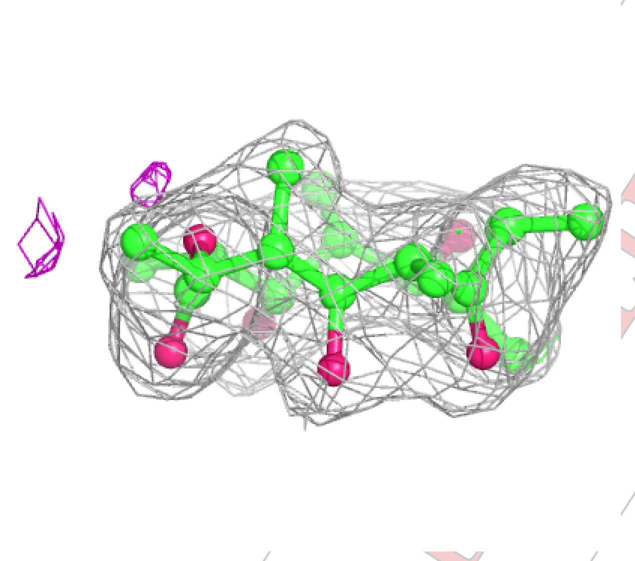

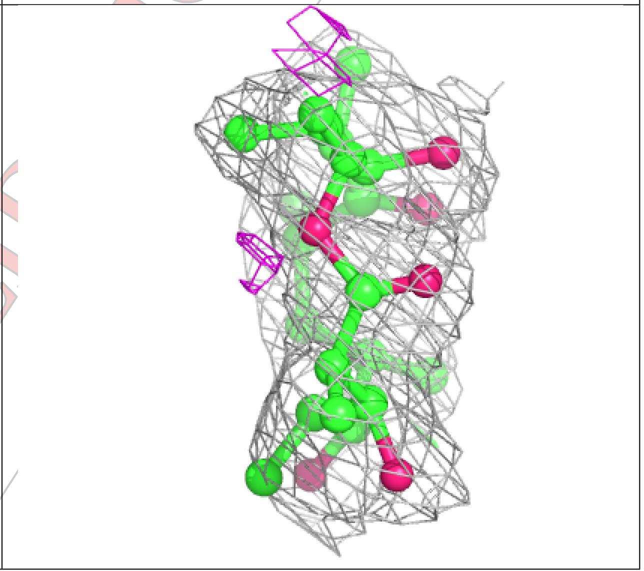

CONFIDENTIAL

Electron density around HEM F 501:

2mF$_o$-DF$_c$ (at 0.7 rmsd) in gray
mF$_o$-DF$_c$ (at 3 rmsd) in purple (negative)
and green (positive)

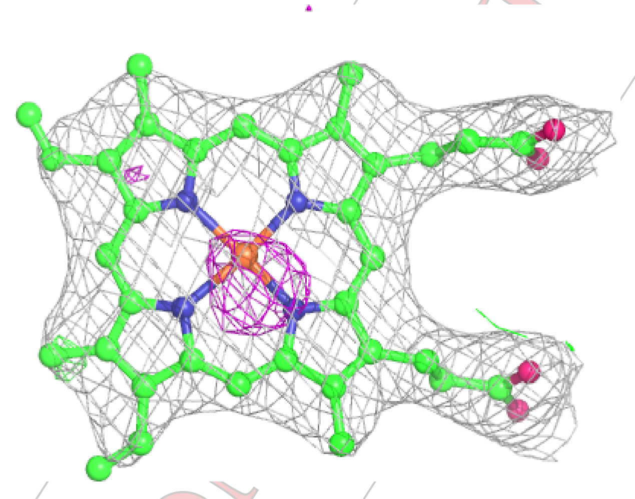

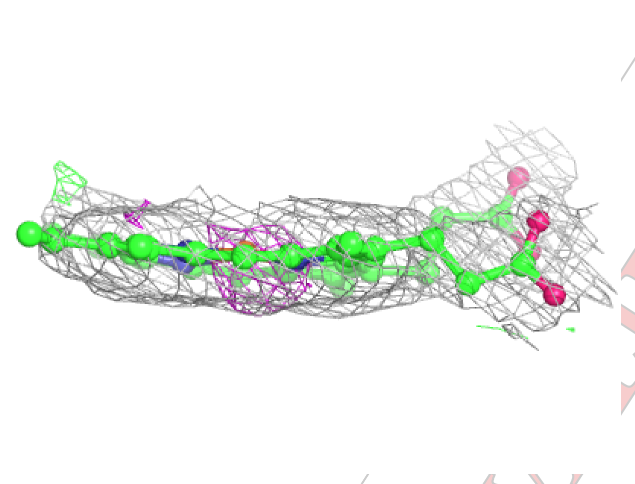

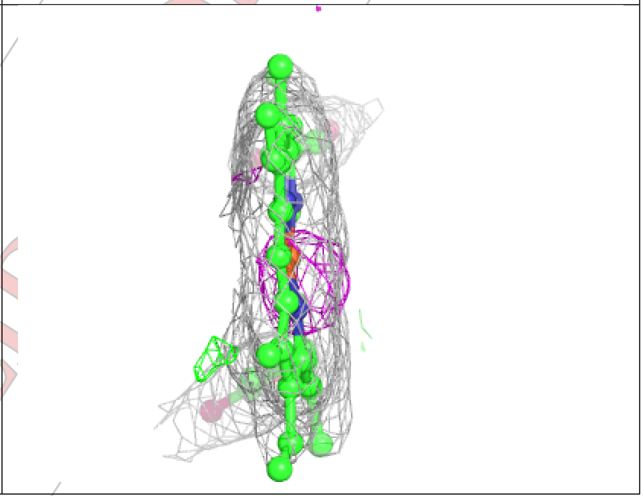

CONFIDENTIAL

Electron density around HEM E 501:

$2mF_o$-DF_c (at 0.7 rmsd) in gray
mF_o-DF_c (at 3 rmsd) in purple (negative)
and green (positive)

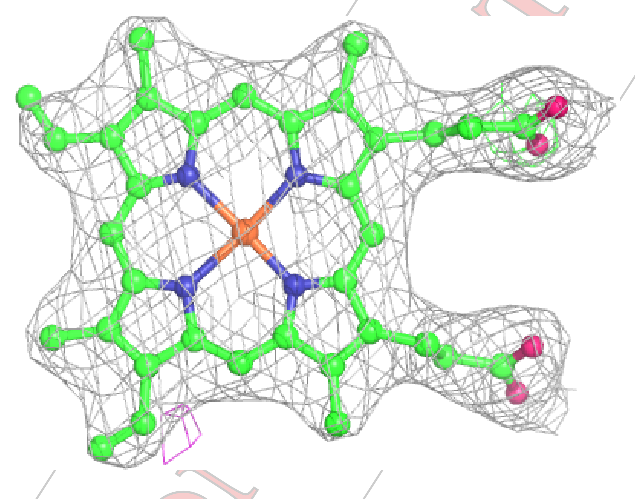

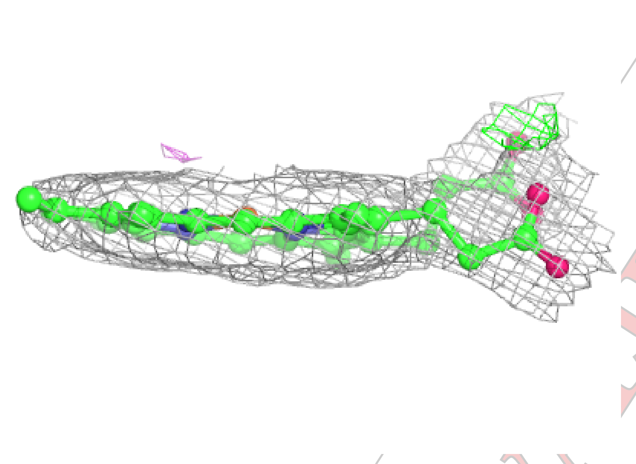

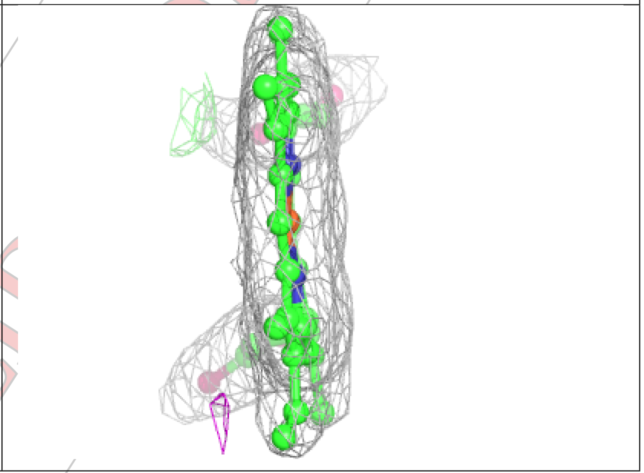

CONFIDENTIAL

Electron density around HEM D 501:

$2mF_o$-DF_c (at 0.7 rmsd) in gray
mF_o-DF_c (at 3 rmsd) in purple (negative)
and green (positive)

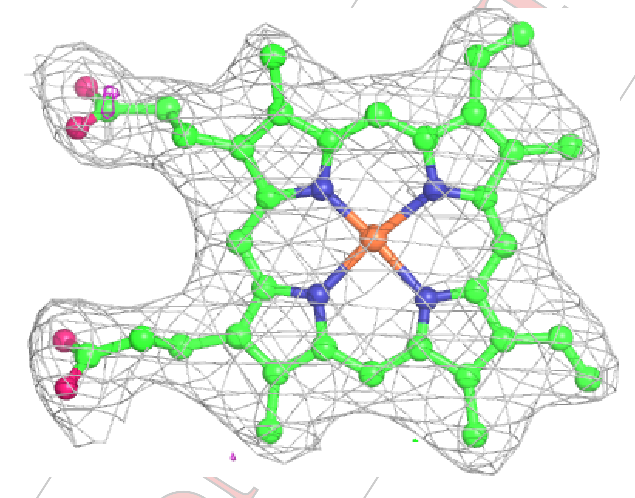

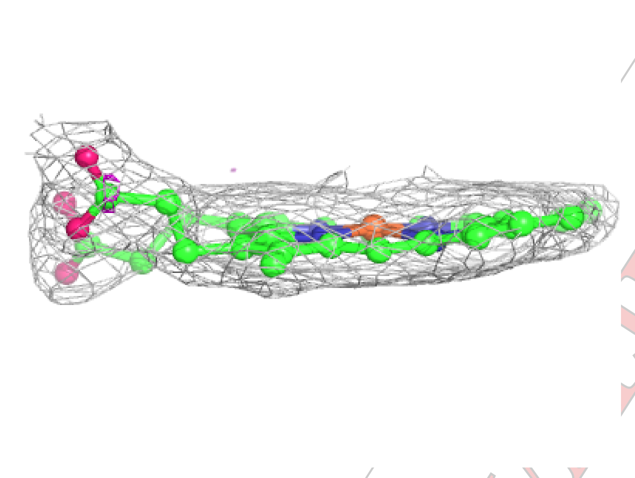

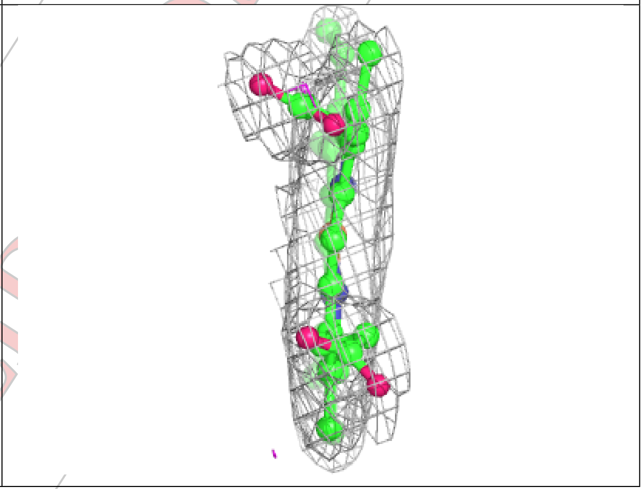

CONFIDENTIAL VALIDATION REPORT

Electron density around HEM C 501:

$2mF_o$-DF_c (at 0.7 rmsd) in gray
mF_o-DF_c (at 3 rmsd) in purple (negative)
and green (positive)

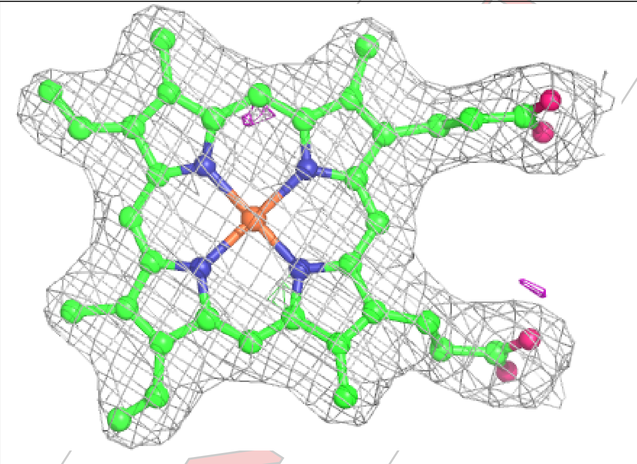

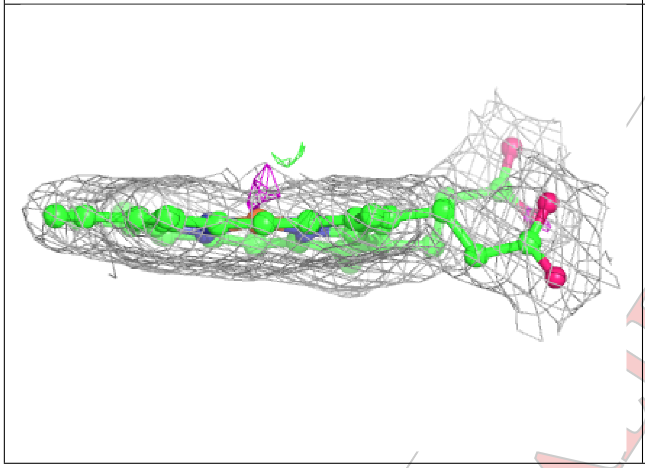

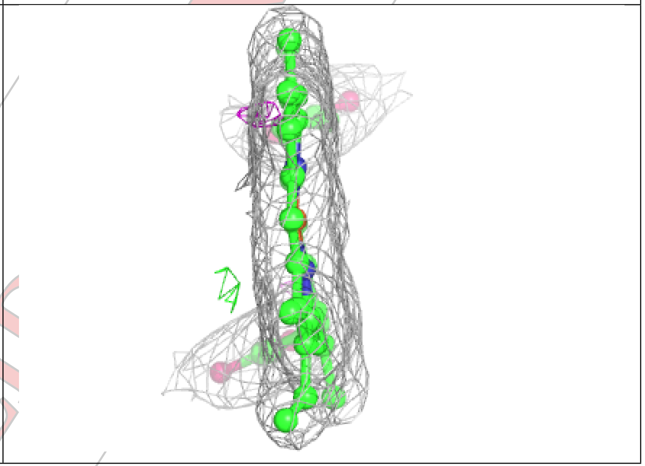

CONFIDENTIAL VALIDATION REPORT

Electron density around HEM B 501:

$2mF_o$-DF_c (at 0.7 rmsd) in gray
mF_o-DF_c (at 3 rmsd) in purple (negative)
and green (positive)

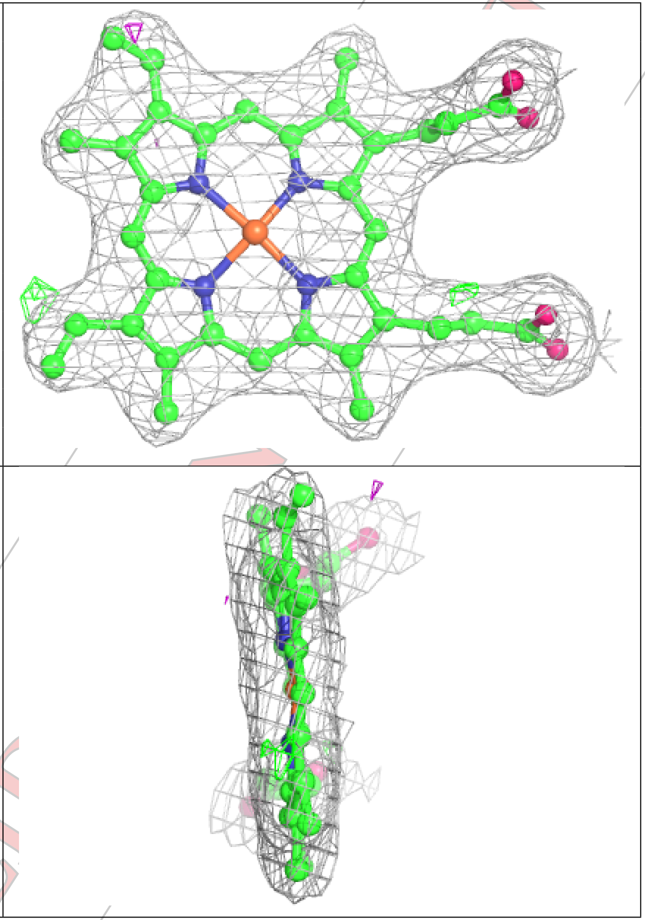

CONFIDENTIAL VALIDATION REPORT

Electron density around HEM A 501:

$2mF_o$-DF_c (at 0.7 rmsd) in gray
mF_o-DF_c (at 3 rmsd) in purple (negative)
and green (positive)

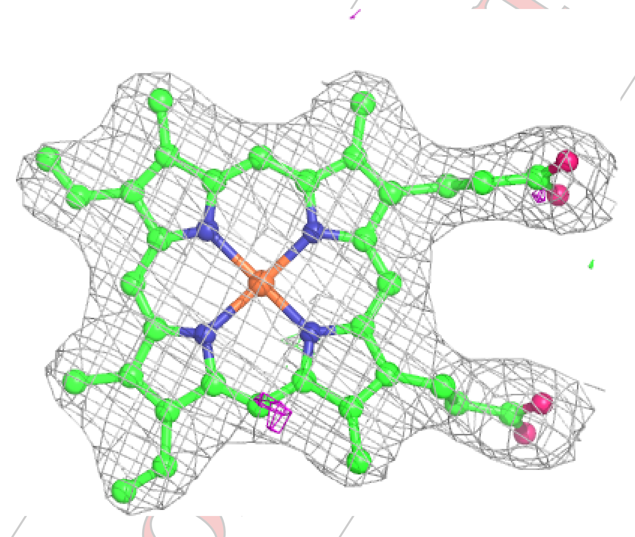

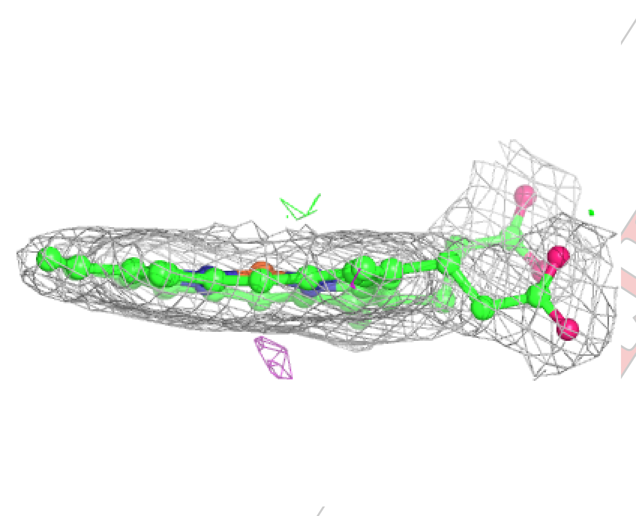

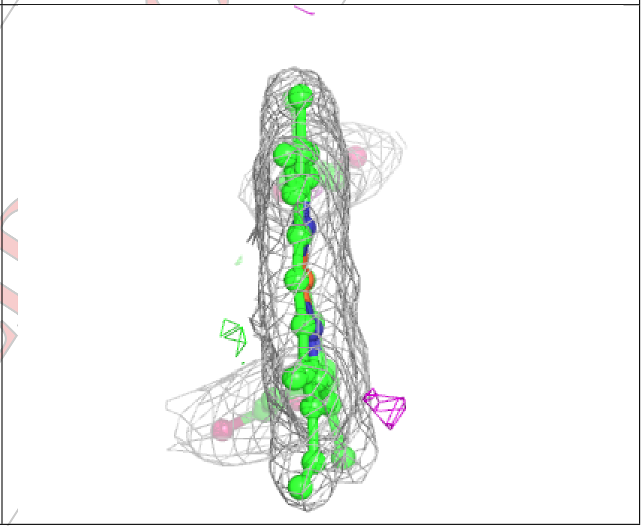

6.5 Other polymers (i)

There are no such residues in this entry.

CONFIDENTIAL
