# Supplementary material for: Dissecting the Cytochrome P450 OleP Substrate Specificity: Evidence for a Preferential Substrate
Source: Biomolecules. 2020 Oct 6;10(10):1411. doi: 10.3390/biom10101411 (PMC7600006; doi:10.3390/biom10101411)
Supplement: Supplementary file 1 [file biomolecules-10-01411-s001.zip › SupplMat_&_ValRep/Supplementary Material.pdf]

Supplementary Material

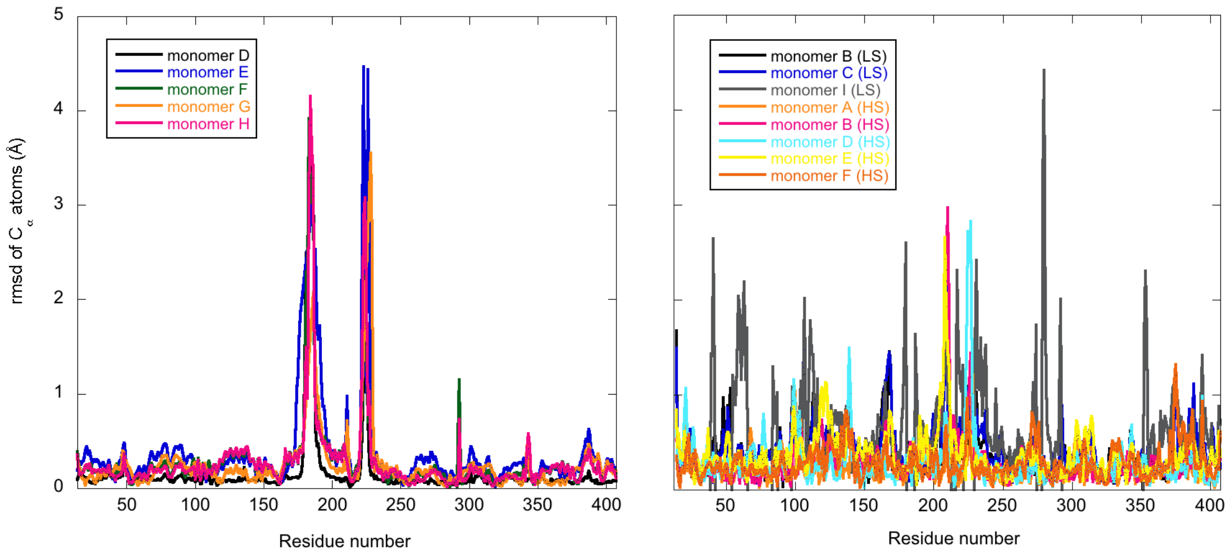

Figure S1. OleP-DEO open and closed conformers. The rmsd on Cα carbons, calculated superposing all the open monomers (left panel) and the closed ones (right panel) of OleP-DEO structures, is reported as a function of the residue number. LS= low salt conditions, HS = high salt conditions.

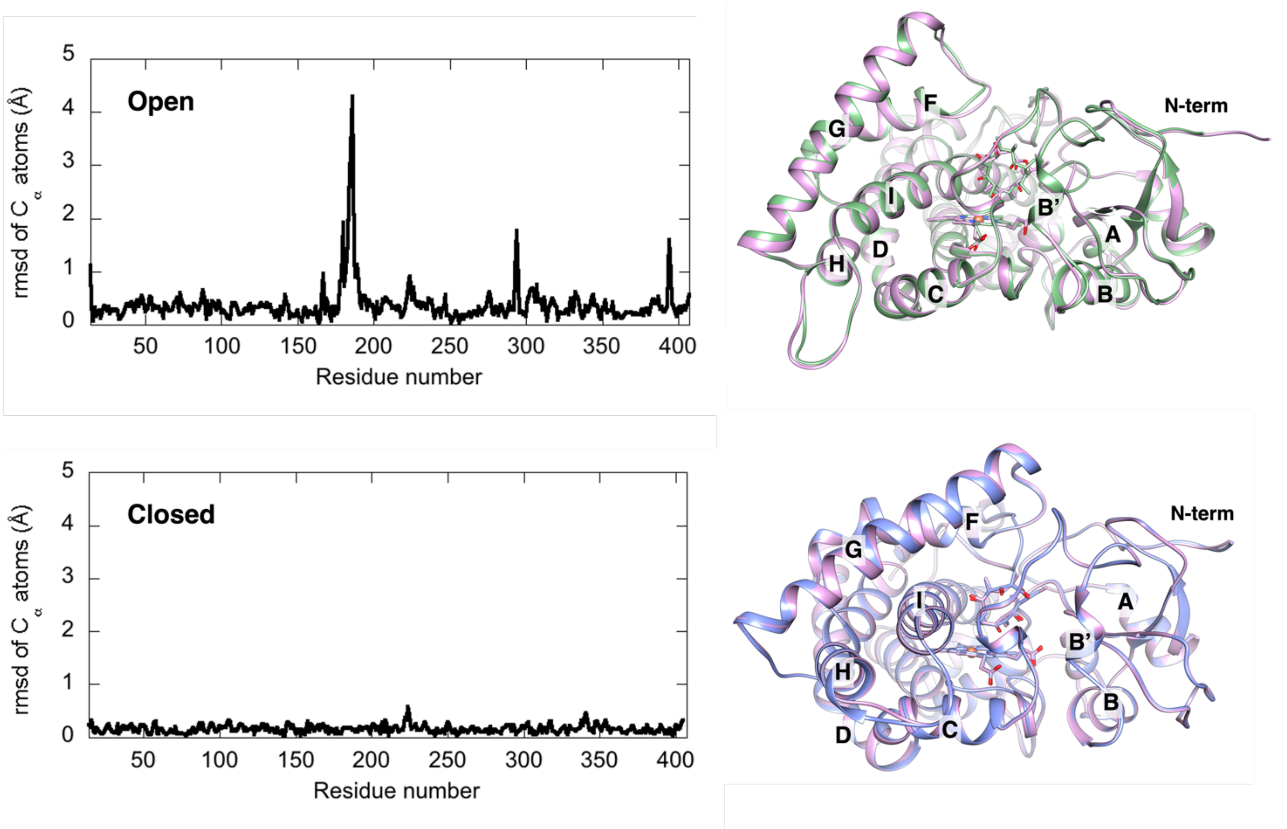

Figure S2. OleP-DEO *vs* OleP-6DEB: structural comparison. The rmsd on Cα carbons, calculated superposing the open conformers (upper panel) and the closed ones (lower panel) of OleP-DEO and OleP-6DEB structures, is reported as a function of the residue number. On the right side, secondary structure superposition of OleP-DEO *vs* OleP-6DEB in the open state (up) and in the closed state (down). OleP-DEO open and closed are in green and blue ribbons representation, respectively. OleP-6DEB open (up) and closed (down) is in pink. Secondary structural elements at the front side are labelled.

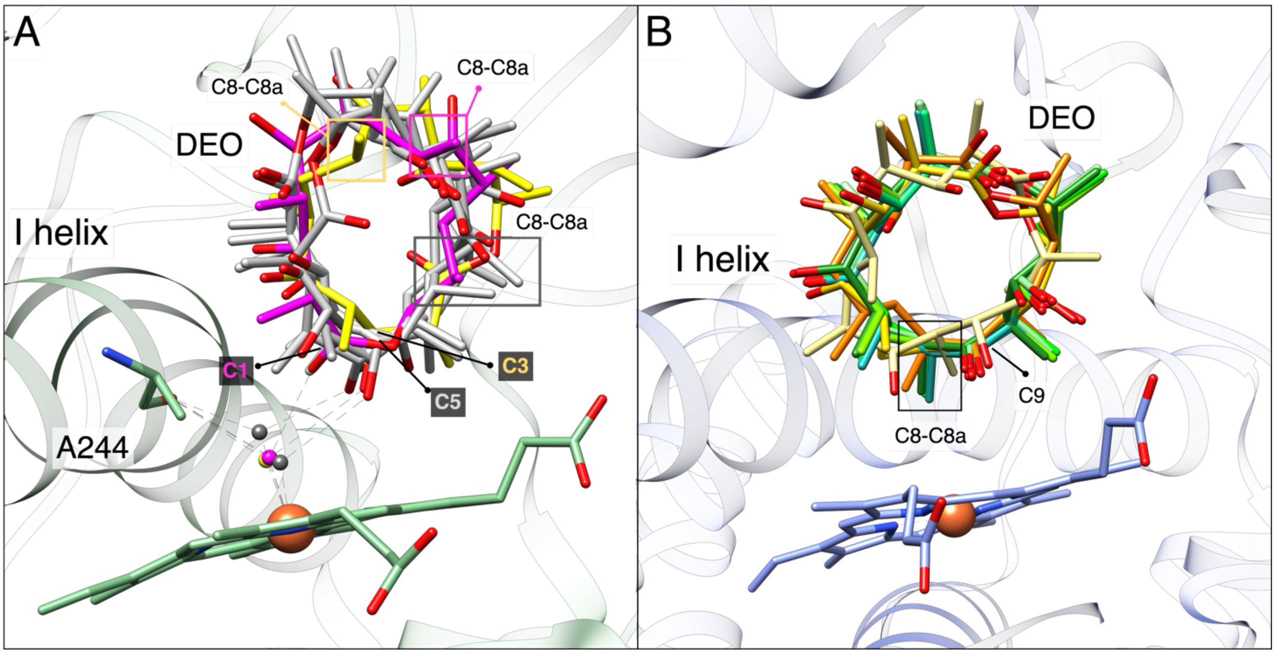

Figure S3. DEO conformations in open and closed OleP. A. Close up view on the active site of OleP-DEO in the open state. All molecules of DEO found in the open monomers of OleP are superposed and colored depending on the orientation adopted with respect to the heme iron: substrate molecules that expose the C5-OH are in grey sticks, in yellow sticks is DEO oriented with the C3-OH facing the heme, and in magenta sticks is DEO exposing the C1-carbonyl group. Colored squared boxes indicate the target bond of OleP (C8-C8a). The sixth coordinating water molecule, typical of the low spin state, is shown as sphere and colored according to the related orientation of DEO. Dashed lines represent water coordination to the heme iron and hydrogen bonds formed with the backbone carbonyl of A244 on helix I and the substrate. **B.** Close up view on the active site of OleP-DEO in the closed state. All DEO molecules found in the nine OleP closed copies, either found in low (yellow tones) and high salt (green tones) conditions, are superposed and colored depending on the monomer. Black squared box indicates the target bond, C8-C8a. C9-OH is also labelled.

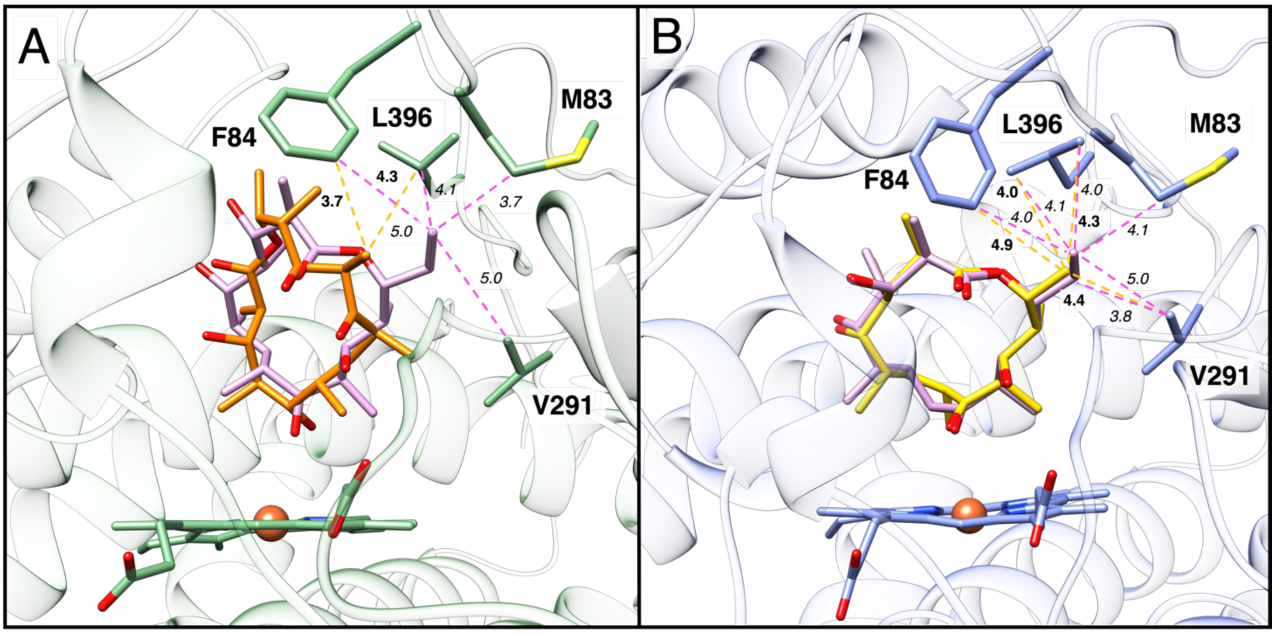

Figure S4. 6DEB *vs* DEO in OleP. Zoom on the active site of OleP in the open (green, panel A) and in the closed (blue, panel B) conformation. Residues forming the hydrophobic niche that hosts the ethyl group at C13 of 6DEB are shown and labelled. Hydrophobic contacts within 5.0 Å formed by OleP with 6DEB (pink) and DEO (orange in the open and yellow in the closed OleP) are shown as dashed lines colored in pink and orange, respectively. The distance is reported in Å (italic: distance in OleP-6DEB, regular bold: distance in OleP-DEO).

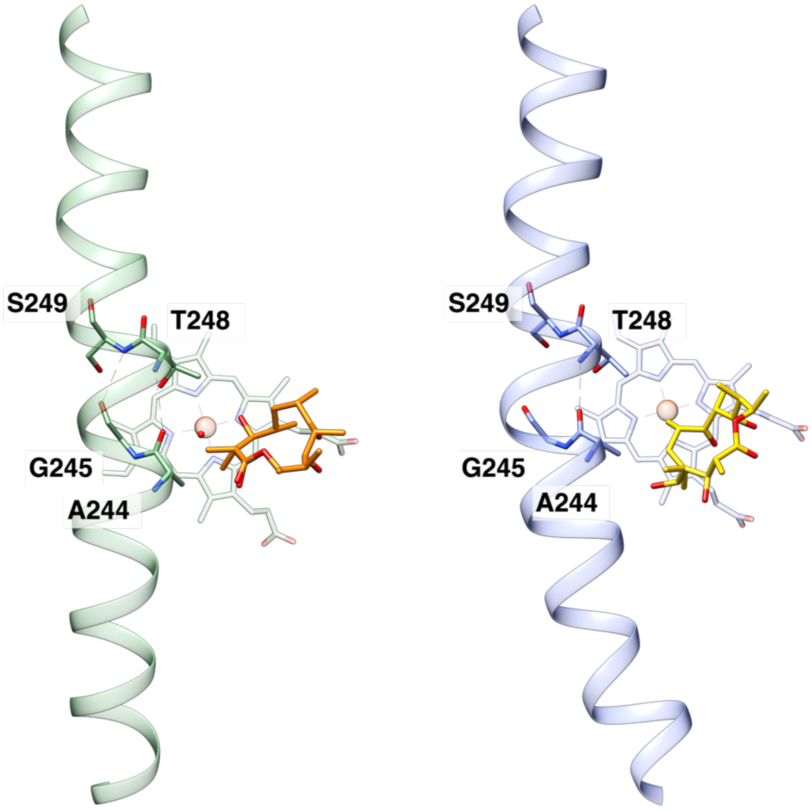

Figure S5. Helix I cleft. Zoom on the I helix of the open (green, left) and the closed (blue, right) OleP in complex with DEO. The repositioning of DEO in the active site that allows the displacement of the sixth coordinating water molecule, induces the bending of the I helix and the formation of the catalytic cleft at the level of the Ala244-Thr248 turn, typical of substrate bound P450s. Residues lining the cleft are labeled and represented as sticks. Dashed lines indicate hydrogen bonds. DEO is in orange (open conformation) and yellow (closed conformation) sticks.

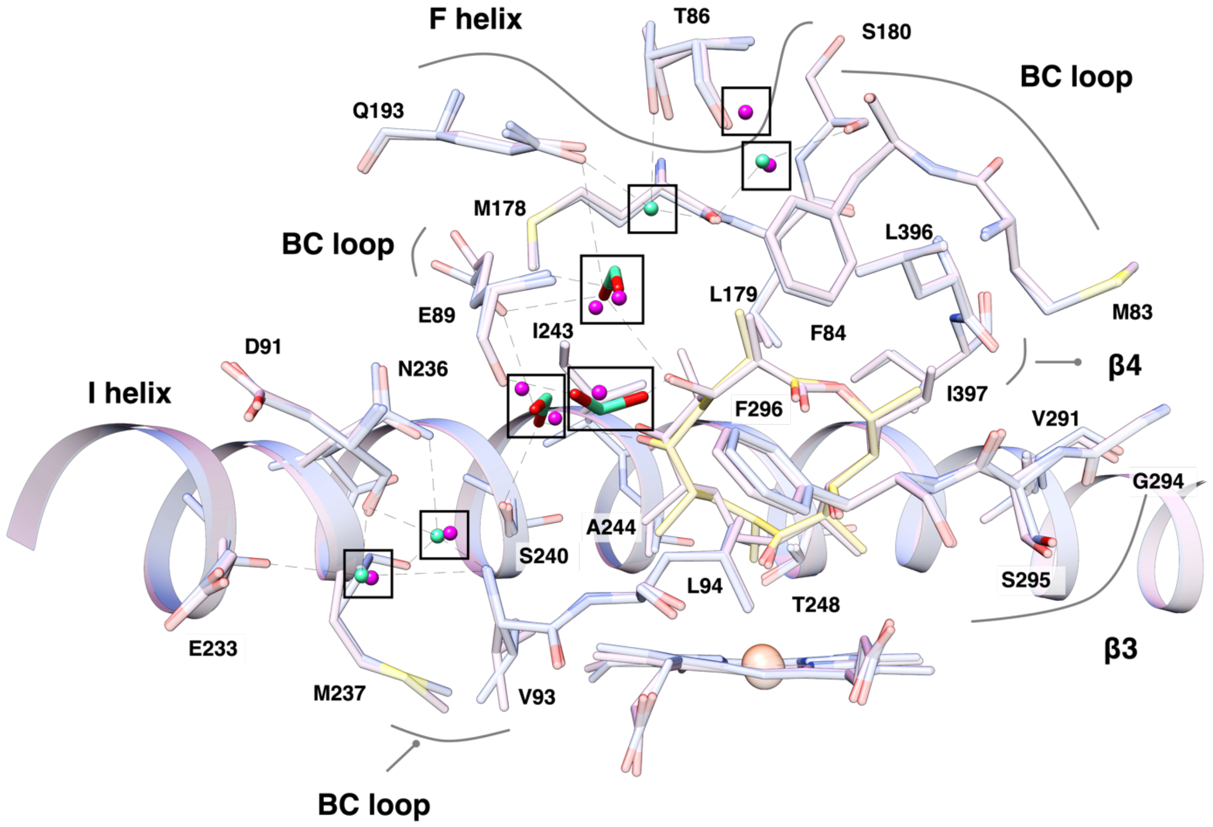

Figure S6. Solvent cavity in closed OleP-DEO and closed OleP-6DEB complexes: comparison. Close up view of the active site of closed OleP in complex with DEO (blue transparency) superposed to the active site of OleP-6DEB in closed conformation (pink transparency). Waters and formate ions that mediate interaction between OleP and DEO are represented as aquamarine spheres and sticks, respectively; waters mediating the contacts between OleP and 6DEB are shown as magenta spheres. Rectangular boxes indicate the eight positions where solvent molecules are found in the structures, six of them being conserved. Secondary structural elements and amino acids involved in OleP-substrate interactions are labeled. Dashed lines represent hydrogen bonds in OleP-DEO. Hemes are shown in transparency as blue (OleP-DEO) and pink (OleP-6DEB) sticks. DEO is represented in transparency as yellow sticks, 6DEB as pick sticks.

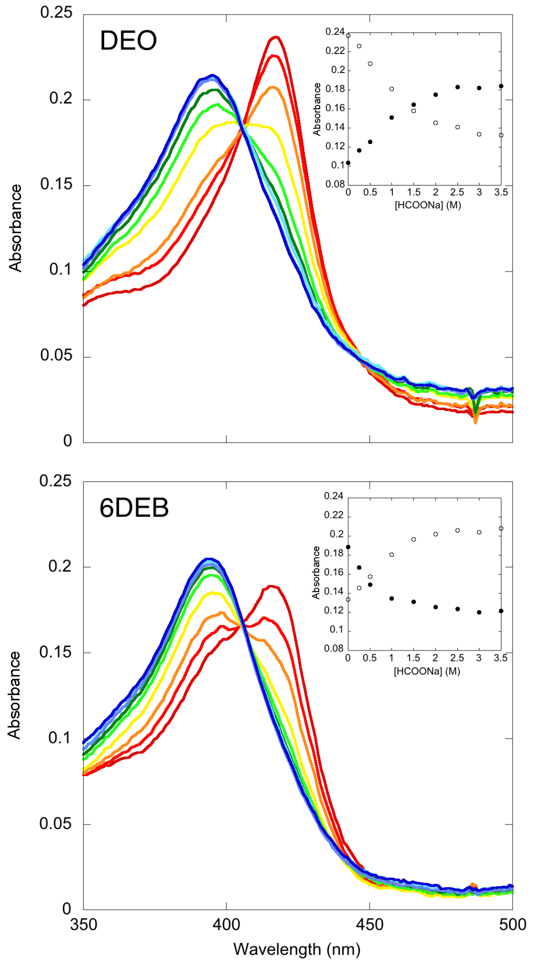

Figure S7. Effect of the ionic strength on the spin-state equilibrium in OleP bound to DEO and to 6DEB. Equilibrium binding experiments. UV-visible absorption spectra of OleP (2 μM) bound to DEO (upper panel) and 6DEB (lower panel) both at 150 μM collected upon addition of increasing concentration of sodium formate at 298 K, in 50 mM Hepes and 200 mM NaCl, pH 7.5. Rainbow colors indicate the direction of the absorption changes observed going from the lowest (absence of HCOONa, red) to the highest ([HCOONa]= 3.5 M, blue) concentration of sodium formate in solution. A clear change of the Soret peak absorption typical of mixed low/high spin-state populations (red) to a full high spin-state population (blue) is visible. Relative insets: absorbance intensities of OleP bound to DEO and to 6DEB as a function of total sodium formate concentration are reported. Data refer to the absorbance monitored at 417 nm (full dots) and at 382 nm for DEO and 388 nm for 6DEB (empty dots). The absorbance intensities display an apparent hyperbolic dependence with ionic strength, decreasing at 417 nm (low spin-state species) and increasing at 382 nm and 388 nm respectively for DEO and 6DEB (high spin-state species) with the increasing salt concentration.

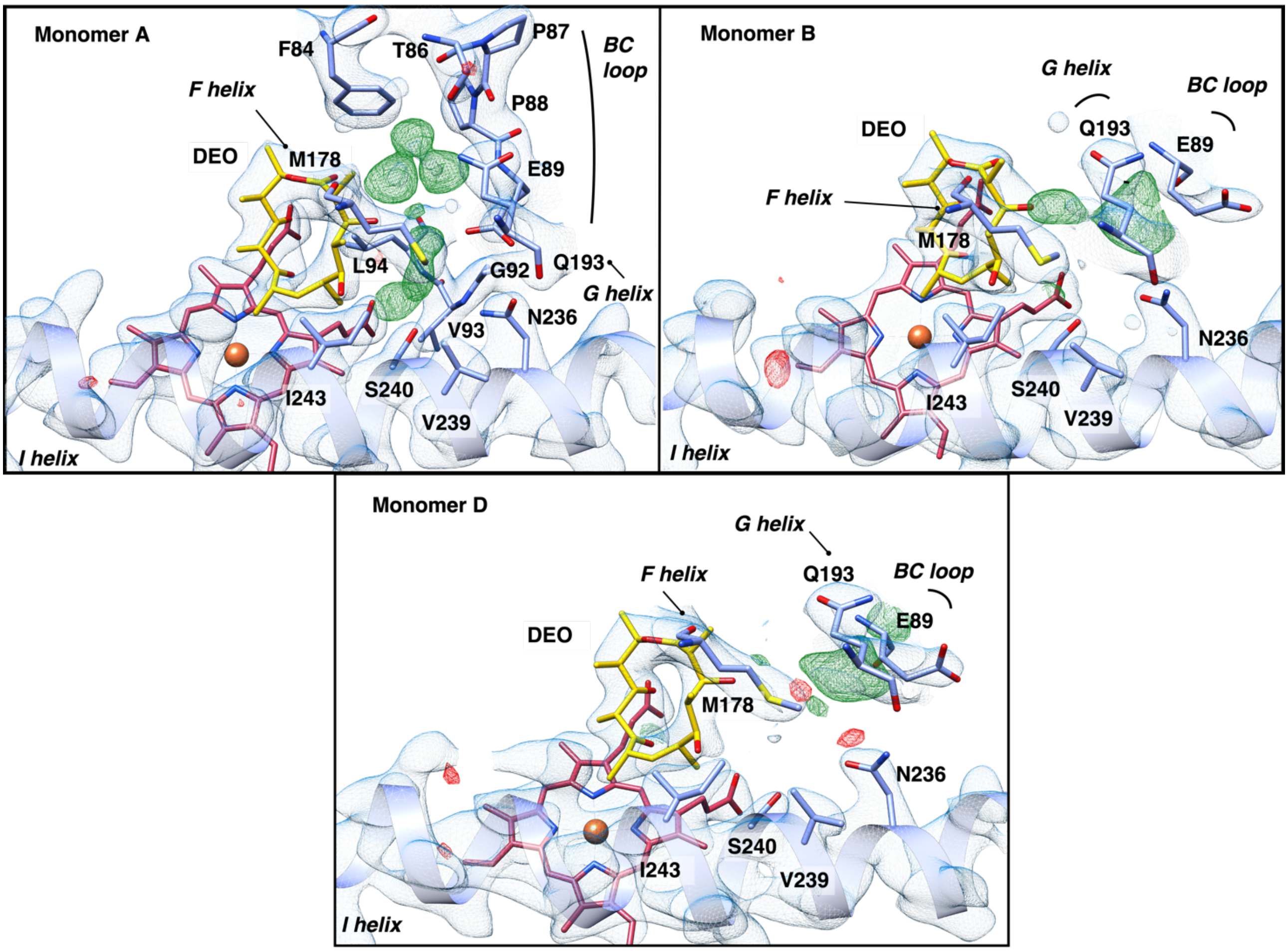

Figure S8. Structure of OleP-DEO bound to L-rhamnose: omit maps. Close up views of the active site OleP-DEO-rhamnose (monomers A, B and D, blue) showing the omit maps calculated excluding L-rhamnose from the model. In each panel, the electron density map ($2F_o–F_c$) contoured at 1 σ (blue mesh) and the (F_o-F_c) map contoured at 3 σ (positive in green, negative in red mesh) are displayed. Residues surrounding the solvent cavity are shown. Secondary structural elements and amino acids are labeled. DEO is represented as yellow sticks.

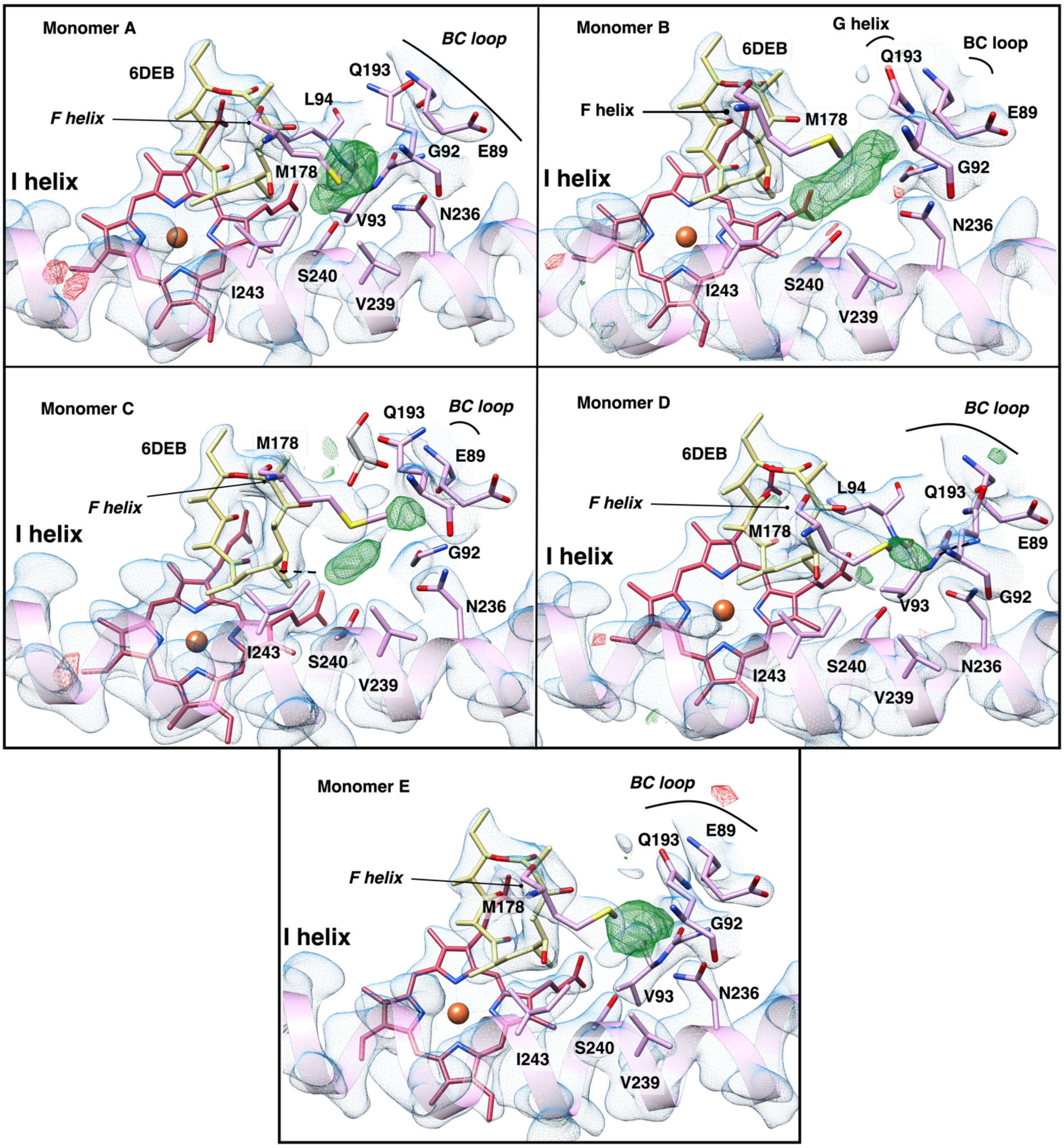

Figure S9. Structure of OleP-6DEB bound to L-rhamnose: omit maps. Close up views of the active site OleP-DEO-rhamnose (monomers A, B, C, D and E, pink) showing the omit maps calculated excluding L-rhamnose from the model. In each panel, the electron density map ($2F_o–F_c$) contoured at 1 σ (blue mesh) and the (F_o-F_c) map contoured at 3 σ (positive in green, negative in red mesh) are displayed. Residues surrounding the solvent cavity are shown. Secondary structural elements and amino acids are labeled. 6DEB is represented as khaki sticks.

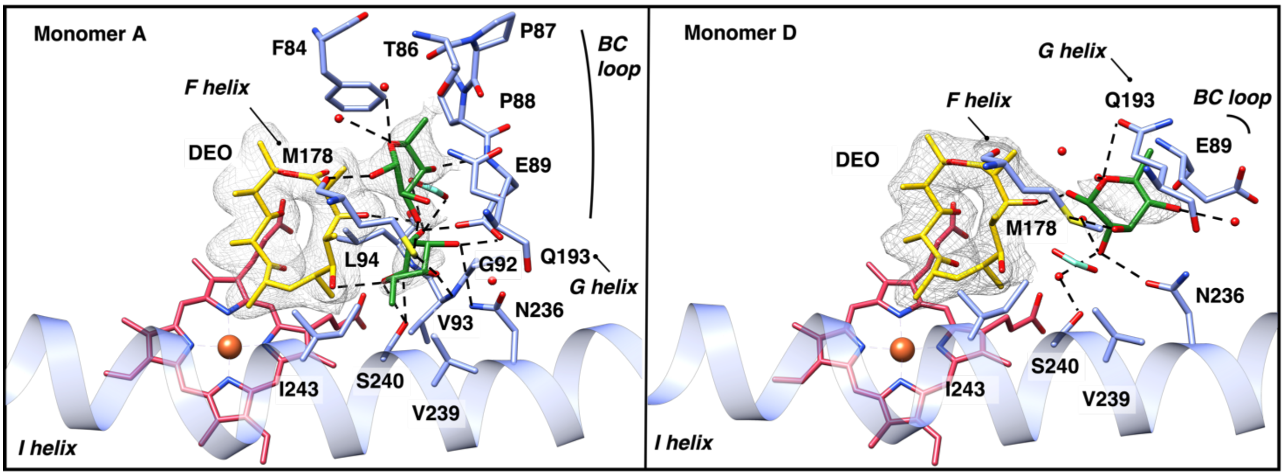

Figure S10. Structure of OleP-DEO bound to L-rhamnose. Close up views of the active site OleP-DEO-rhamnose (monomers A and D, blue). Residues and solvent molecules (waters, red spheres; formate ions, aquamarine sticks) within 5 Å from L-rhamnose are displayed. Dashed lines represent hydrogen bonds. Secondary structural elements and amino acids are labeled. In both panels, the electron density map ($2F_o$-F_c) contoured at 1 σ around L-rhamnose (green sticks) and DEO (yellow sticks) is shown in grey mesh. In monomer A, two L-rhamnose conformers occupying different positions were identified within the solvent cavity.

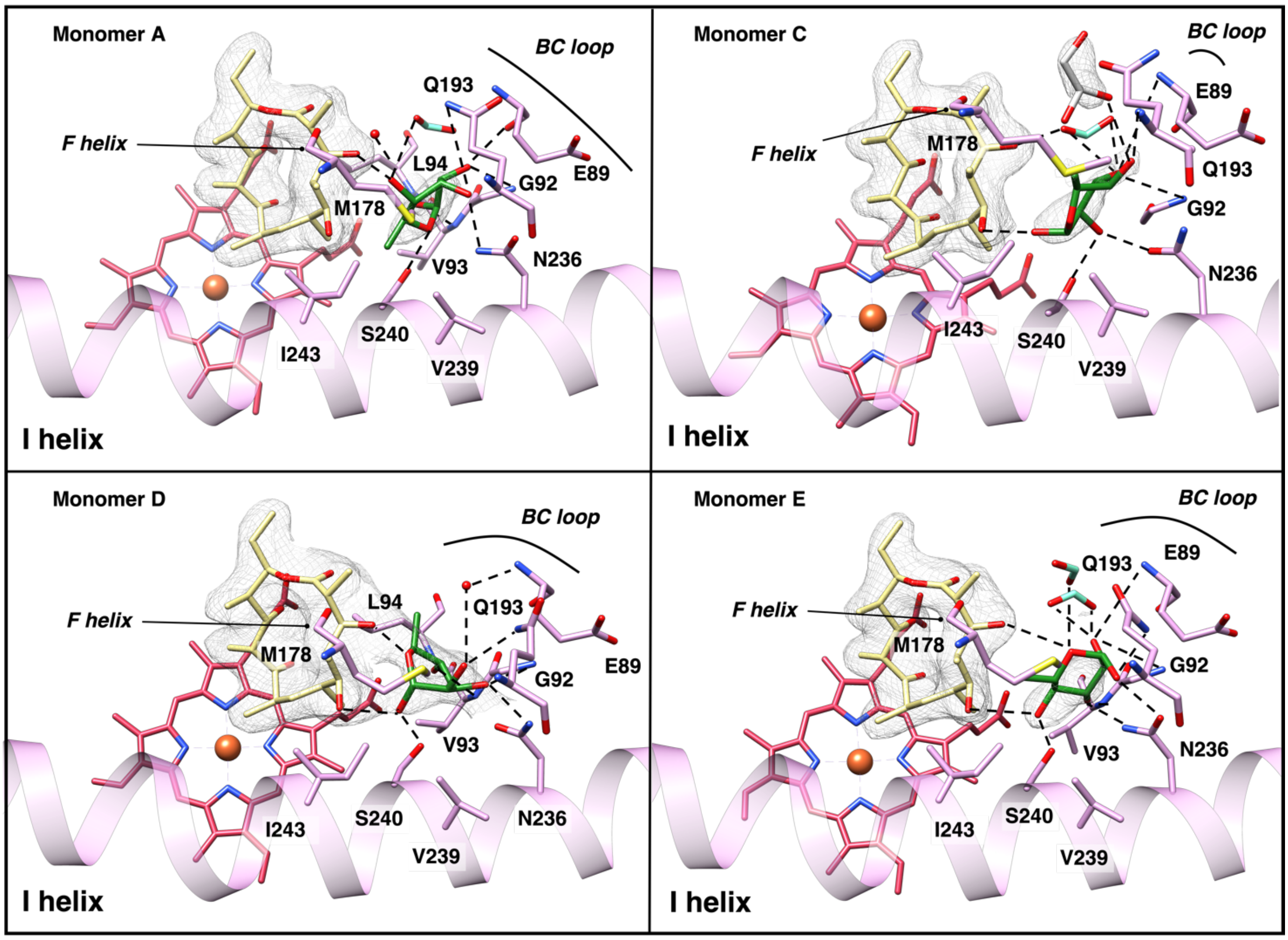

Figure S11. Structure of OleP-6DEB bound to L-rhamnose. Close up views of the active site OleP-6DEB-rhamnose (monomers A, C, D and E, pink). Residues and solvent molecules (waters, red spheres; formate ions, aquamarine sticks) within 5 Å from L-rhamnose are displayed. Dashed lines represent hydrogen bonds. Secondary structural elements and amino acids are labeled. In both panels, the electron density map ($2F_o$-F_c) contoured at 1 σ around L-rhamnose (green sticks) and 6DEB (khaki sticks) is shown in grey mesh.
